# Expression-based drug screening of neural progenitor cells from individuals with schizophrenia

**DOI:** 10.1038/s41467-018-06515-4

**Published:** 2018-10-24

**Authors:** Benjamin Readhead, Brigham J. Hartley, Brian J. Eastwood, David A. Collier, David Evans, Richard Farias, Ching He, Gabriel Hoffman, Pamela Sklar, Joel T. Dudley, Eric E. Schadt, Radoslav Savić, Kristen J. Brennand

**Affiliations:** 1 Department of Genetics and Genomic Sciences, Icahn School of Medicine at Mount Sinai, New York, NY 10029 USA; 2 Icahn Institute for Genomics and Multiscale Biology, Icahn School of Medicine at Mount Sinai, New York, NY 10029 USA; 3 Institute for Next Generation Healthcare, Icahn School of Medicine at Mount Sinai, New York, NY 10029 USA; 4 Department of Neuroscience, Icahn School of Medicine at Mount Sinai, New York, NY 10029 USA; 5 Friedman Brain Institute, Icahn School of Medicine at Mount Sinai, New York, NY 10029 USA; 6 Eli Lilly and Company Ltd, Erl Wood Manor, Surrey, UK; 7 Social, Genetic and Developmental Psychiatry Centre, Institute of Psychiatry, King’s College London, London, UK; 8 Department of Psychiatry, Icahn School of Medicine at Mount Sinai, New York, NY 10029 USA; 9 Sema4, a Mount Sinai venture, Stamford, Connecticut, USA; 10 Present Address: ASU-Banner Neurodegenerative Disease Research Center, Arizona State University, Tempe, AZ 85287-5001 USA

## Abstract

A lack of biologically relevant screening models hinders the discovery of better treatments for schizophrenia (SZ) and other neuropsychiatric disorders. Here we compare the transcriptional responses of 8 commonly used cancer cell lines (CCLs) directly with that of human induced pluripotent stem cell (hiPSC)-derived neural progenitor cells (NPCs) from 12 individuals with SZ and 12 controls across 135 drugs, generating 4320 unique drug-response transcriptional signatures. We identify those drugs that reverse post-mortem SZ-associated transcriptomic signatures, several of which also differentially regulate neuropsychiatric disease-associated genes in a cell type (hiPSC NPC vs. CCL) and/or a diagnosis (SZ vs. control)-dependent manner. Overall, we describe a proof-of-concept application of transcriptomic drug screening to hiPSC-based models, demonstrating that the drug-induced gene expression differences observed with patient-derived hiPSC NPCs are enriched for SZ biology, thereby revealing a major advantage of incorporating cell type and patient-specific platforms in drug discovery.

## Abstract (web summary)

Unbiased large scale screening of small molecules for drug discovery in psychiatric disease is technically challenging and financially costly. Here, Readhead and colleagues integrate in silico and in vitro approaches to design and conduct transcriptomic drug screening in schizophrenia patient-derived neural cells, in order to survey novel pathologies and points of intervention.

## Introduction

Schizophrenia (SZ) is a highly heritable neuropsychiatric disorder (NPD)^1^, with genetic risk reflecting a combination of highly penetrant rare mutations^2^ and common variants of small effect^3^. All currently Food and Drug Administration-approved antipsychotic drugs for the treatment of SZ share antagonist activity against the dopamine D2 receptor and predominantly address positive psychotic symptoms (for review, see ref. ^4^). Approximately one-third of SZ patients do not respond to antipsychotic medications and another third have only a partial response^5^; antipsychotic responsiveness has been hypothesized to be a heritable component of disease risk^6^. Current pharmaceutical in vitro drug discovery platforms for SZ frequently combine immortalized cell lines and simple biological readouts (such as receptor-binding properties) (reviewed^7^), but the drug discovery success rate for NPD is particularly low^8^. Consequently, drug development tends to focus on refining existing treatments toward reducing side-effect profiles^9^ and/or increasing efficacy through adherence^10^.

An improved drug-screening strategy would more faithfully recapitulate SZ biology and also integrate advances in psychiatric genetics^2,3^. Human induced pluripotent stem cell (hiPSC)-based models of SZ have identified a number of neural^11,12^, synaptic^13,14^ and molecular^15–18^ phenotypes in patient-derived hiPSC neurons, demonstrating the feasibility of a more personalized approach to drug discovery. The protracted experimental timelines to synaptic maturity combined with difficulties associated with high-content synaptic screening assays (reviewed^19,20^) have limited the adoption of hiPSC neurons to high-throughput drug screening for psychiatric disease. As an alternative, we tested whether hiPSC-derived neural progenitor cell (hiPSC NPC)-focused gene expression-based screening represented a scalable alternative approach.

**Fig. 1:**
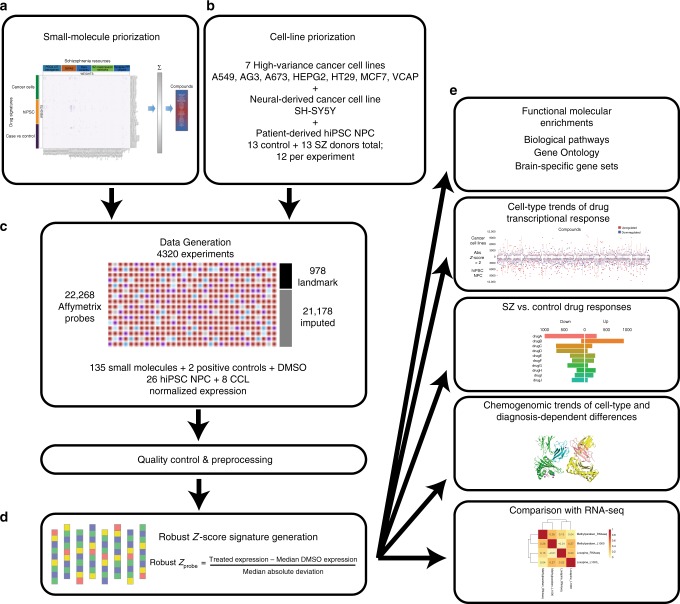
Summary schematic of experimental and analytic pipeline. **a** One hundred and thirty-five drugs were prioritized for screening based on connectivity with diverse aspects of SZ-related biology. **b** Cells used for screening comprised seven CCLs (A549, AGS, A673, HEPG2, HT29, MCF7, and VCAP) that were prioritized using LINCs datasets, one additional neural CCL (SH-SY5Y) and hiPSC NPCs from 13 SZ and 13 control individuals (12 each per drug). **c** Data were generated using the L1000 platform to yield 6650 drug-perturbation transcriptomic profiles (135 drugs tested across 26 hiPSC NPCs and 8 CCLs). After data quality control, normalized expression was converted to **d** robust *Z*-scores based on comparison with isogenic DMSO-treated experiments, and used as inputs for **e** functional molecular enrichments, cell-type-specific (hiPSC NPCs and CCLs) trends, diagnosis-dependent (SZ and control) responses, and chemogenomics analyses. Global transcriptomic responses of two drugs were tested across hiPSC NPCs from three SZ and three control individuals by RNA-seq, as part of a validation of the L1000 results

Comprehensive data-driven models can inform disease understanding and identify potential drug targets (reviewed in ref. ^21^). We applied an integrative genomics approach to predict and evaluate drug-induced perturbations in hiPSC NPCs, an easy to culture^22^ human neural cell type arguably more relevant to SZ than the transformed cancer cell lines (CCLs) historically used for drug screening. Across 135 drugs prioritized in silico, we conducted a transcriptomic screen of hiPSC NPCs from 12 SZ patients and 12 healthy controls each, as well as 8 CCLs. This head-to-head comparison of hiPSC NPCs with CCLs queried the extent to which cell-type-specific and diagnosis-dependent drug responses impacted SZ-related transcriptomic signatures and gene sets enriched for SZ biology. Drug-induced gene expression changes observed in hiPSC NPCs relative to CCLs and, to a lesser extent, in SZ hiPSC NPCs relative to control hiPSC NPCs were enriched for genes linked to SZ. Patient hiPSC-based neural screening captured molecular responses to drug perturbations in a more disease-relevant in vitro system, obtaining results that more strongly connected to SZ (Fig. 1).

## Results

### Transcriptomic-based drug-screening approaches

We applied an in silico computational drug-screening approach to select the drugs for our transcriptomic-based drug screening. From a total of 6269 small molecules (herein referred to as drugs), 135 were prioritized based on predicted or known interactions with diverse SZ-relevant biology (Supplementary Table 1). We collated SZ sets from transcriptomic, genetic, and protein-centric data [including SZ post-mortem brain gene expression^23^ (SZ networks), SZ-risk gene sets based on common^3^ and rare variants^24,25^ (SZ-risk genes), and synaptic protein PPI (protein–protein interaction) communities generated from subsetting human protein interaction data], as well as synapse-specific gene sets^26^ that were filtered on NPD-associated gene sets (Supplementary Table 2) (Synapse PPI communities). Fifty-seven drugs were prioritized through a connectivity mapping approach^27^, according to their predicted ability to differentially regulate the transcription of SZ sets. Sixteen additional drugs were chosen based on overlap of referenced (DrugBank 4.1^28^) and predicted (SEA^29^) drug-target associations with the SZ sets. A further 58 drugs were selected because they were known to target SZ-relevant genes.

In total, we compared hiPSC NPCs (12 SZ and 12 control) and CCLs (8) using 135 drugs, generating 4320 unique signatures. NPCs used in this study were previously characterized as part of two independent hiPSC cohorts of SZ and control hiPSCs^12,15^; hiPSC NPCs from 11 SZ patients and 11 controls were used across all 135 drugs, whereas 2 SZ patients and 2 controls each received half of the drugs tested. Seven CCLs were selected from the LINCS^30^ dataset, prioritized according to the fraction of transcriptomic variance captured by each cell type^31,32^. An additional CCL derived from neuroblastoma cells (SH-SY5Y) was added to ensure that a neural cell type was represented. Available clinical data for all cell lines is presented in Supplementary Table 3. Drug treatment concentrations are listed in Supplementary Table 4.

Drug-induced gene expression profiles were generated using the L1000 platform^33^ (Supplementary Figure 1 and Supplementary Data 1). Direct measures of 978 landmark probes were used to impute normalized transcriptomic expression^31,32^ (see Methods). Drug perturbation signatures for each unique drug–cell–plate combination were transformed to robust Z-scores (RZS)^34^, representing the comparison of normalized gene expression for treated wells with cell-line-matched, dimethyl sulfoxide (DMSO)-treated wells within each plate.

**Fig. 2:**
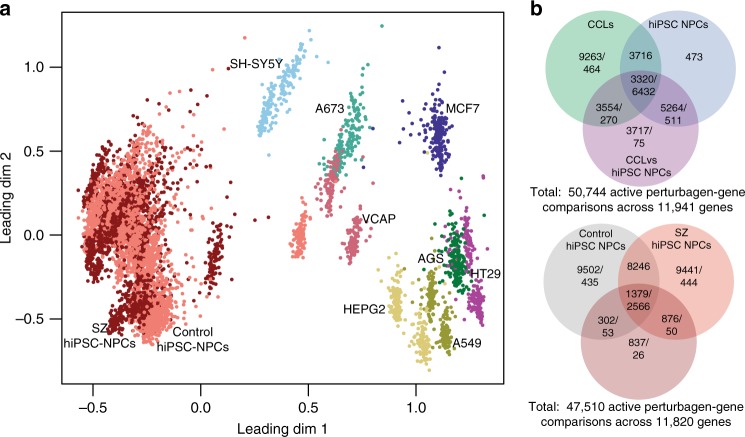
Global summary of expression trends and frequently perturbed genes in hiPSC NPCs and CCLs. **a** Multi-dimensional scaling plot of normalized expression data for all drug-induced gene expression profiles across each hiPSC NPC and CCL. Each point represents the normalized gene expression corresponding to a single experiment. **b** Venn diagrams showing drug perturbation gene differential expression results by comparison type. Drug-gene perturbagen (DGP) comparisons were defined as active (abs-RZS > 2) in one or more of the four within-cell type (CCL, hiPSC NPC, SZ hiPSC NPC, and control hiPSC NPC) comparisons and two between cell-type comparisons (CCL vs. hiPSC NPC, control vs. SZ hiPSC NPC), one row per DGP-comparison combination. Three circles are required, because a particular DGP test may independently be called as active in the CCLs, hiPSC NPCs, and/or CCL vs. hiPSC NPC tests (or control hiPSC NPCs, SZ hiPSC NPCs, and/or control vs. SZ hiPSC NPCs). The first number in each section is the number of drug perturbation gene differential expression counts, whereas the second number shows the number of genes with at least one drug–gene differential expression result. Top panel shows overlap between CCLs, hiPSC NPCs, and CCLs vs. hiPSC NPCs. Bottom panel shows overlap between control hiPSC NPCs, SZ hiPSC NPCs, and control vs. SZ hiPSC NPCs. Twenty-three percent of the 50,744 drug–gene DE had opposite results in hiPSC NPCs and CCLs, but not large enough to meet the DE threshold; 56% had DE results in one cell type but not the other; 20% had effects that were directionally the same in both cell types, but larger in magnitude in one cell type vs. the other; 0.6% had DE results in both cell types but in opposite directions. Twenty-five percent of the 47,510 DE were opposite between SZ and control hiPSC NPCs, but not large enough to meet the DE threshold; 35% represented a DE in one cell type but not the other; 41% represented effects that were directionally the same in both cell types, but larger in magnitude in one cell type than the other; none showed a DE result in opposing directions

As expected, cell type (hiPSC NPC and CCL) was a major source of variation in the gene expression data (Fig. 2a). Clustering of CCLs was more distinct; there was little to no separation between individual hiPSC NPC lines or between SZ and control hiPSC NPC group means. Variance partition analysis^35^ quantified the sources of variance across all L1000 experiments (Supplementary Figure 2a), confirming that multiple covariates, particularly technical variables (such as L1000 plate, treatment plate, and project phase) accounted for significant proportions of variation in the gene expression data. Transformation of data to RZS-based comparisons eliminated the effect of these technical drivers of variance. Although this strategy precluded our ability to draw insights about baseline differences between cell lines, it clarified the cell-type-dependent drug responses described below.

### Amelioration of post-mortem SZ transcriptomic signatures

We evaluated each drug on its ability to reverse a SZ transcriptomic signature. We used an external post-mortem, rather than a within-study-derived hiPSC-based signature, because our hiPSC NPC donor sample size was small relative to available post-mortem datasets^23^ and because the RZS transformation we used to remove technical sources of variation precluded studying baseline differences between cell lines. We used a gene-set enrichment analysis^36–38^, instead of a direct examination of relative expression levels, because the L1000 platform better examines correlational patterns of gene response than relative expression differences of inferred genes.

**Fig. 3:**
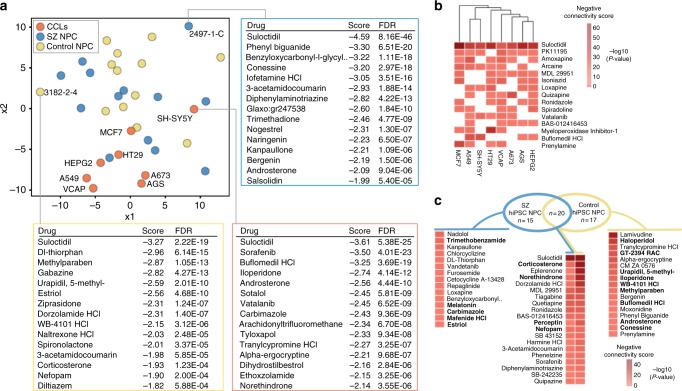
Ability of drug-induced responses to reverse a SZ-related transcriptional signature. **a** Multi-dimensional scaling plot of individual cell lines based on connectivity scores between drugs and SZ post-mortem differentially expressed (DE) genes^23^. Top 15 drugs predicted to normalize SZ post-mortem DE genes, for representative control and SZ hiPSC NPCs, as well as the neuroendocrine derived CCL, SH-SY5Y. **b** Top 15 drugs sorted according to predicted ability to normalize SZ post-mortem DE genes across multiple CCLs. **c** Summary of drugs predicted to normalize SZ post-mortem DE genes in hiPSC NPCs, based on negative connectivity with aggregated SZ and control hiPSC NPC experiments. Heatmap cells with negative connectivity score corresponding to FDR < 0.1 are indicated. Highlighted in bold are the 18 drugs (5/15 SZ-specific, 9/17 control-specific, and 4/20 non-specifically in hiPSC NPCs) that were independently identified within the top 30 drugs with SZ-specific regulation of SZ-sets in Fig. 5b

Across each hiPSC NPC and CCL, we asked to what extent the post-mortem SZ-related transcriptomic signature was modulated by drug treatment. Multi-dimensional scaling assigned each hiPSC NPC and CCL a connectivity score between the 135 drugs and the SZ post-mortem differentially expressed (DE) genes^23^ (Fig. 3a). In general, CCLs occupied the lower segment of the plot and hiPSC NPCs the upper half. The neuroblastoma cell line (SH-SY5Y) sat relatively closer to the majority of hiPSC NPCs than the other CCLs, perhaps reflecting its neural origins. For one SZ and one control hiPSC NPC (selected based on their differential connectivity), as well as SH-SY5Y, we noted the top 15 drugs predicted to normalize SZ DE genes, conveying that for some drugs (i.e., suloctidil) there was a strong and shared response, whereas for others there was not. In fact, suloctidil reversed SZ-related transcriptomic signatures across all CCL and hiPSC NPC contexts, with a high pairwise correlation between all RZS (mean pairwise correlation: 0.42).

Fifty-two of the 135 drugs (39%) ameliorated the SZ-related transcriptomic signature in hiPSC NPCs, 61% of these in a diagnosis-dependent manner (Fig. 3c). Twenty drugs ameliorated the SZ DE in both control and SZ hiPSC NPCs, 15 in SZ hiPSC NPCs only, and 17 in control hiPSC NPCs only. Many of the drugs found to reverse the SZ transcriptional signature (Fig. 3c, highlighted in bold) also produced diagnosis-dependent regulation of SZ sets (see below).

For a subset of the SZ cases in this study, clinical responsiveness to clozapine was previously evaluated^39^. Perhaps due to our small sample size, we observed only a poor stratification of patients by multi-dimensional scaling of clozapine-induced gene expression changes to known clozapine response (Supplementary Figure 3). We cannot conclude whether hiPSC NPC transcriptional response to clozapine differed between patients with clozapine-responsive and non-responsive SZ.

### Cell-type-specific transcriptomic drug responses

We compared drug-induced gene expression changes between CCLs and overall hiPSC NPC drug signatures before separately considering SZ and control hiPSC NPC drug signatures. All DE (absolute RZS ≥ 2) drug–gene results within hiPSC NPCs and CCLs were identified (Supplementary Data 1). Unexpectedly, CCLs showed diminished overall drug responsiveness, with approximately 15% fewer total drug-probe DE results (Bonferroni (BF) *p* < 0.001) (Supplementary Data 1; Fig. 2b). In total, 11,132 genes were perturbed by an average of 3.1 drugs across hiPSC NPCs, whereas 10,882 genes were perturbed by an average of 2.7 drugs in CCLs; 7288 genes showed a differential DE (absolute difference in median RZS ≥ 2) between cell types by an average of 2.2 drug perturbations (Supplementary Data 1; Fig. 2b). Of the 50,744 drug–gene DE results, 15,855 showed a differential DE result between hiPSC NPCs and CCLs.

The overall pattern of drug response between hiPSC NPCs and CCLs was distinct (Fig. 2a), but the biological pathways associated with the most frequently perturbed gene sets overlapped between CCLs and hiPSC NPCs (hiPSC NPC; Supplementary Figure 4b,d; CCL, Supplementary Figure 4a,c). Two hundred and fourteen genes were differentially perturbed in a cell type-specific manner by ≥ 5 drugs (absolute difference in RZS ≥ 2, |hiPSC NPC – CCL|, Up: 93, Down: 119, both Up and Down: 2). The top cell-type enrichment for the genes most frequently downregulated in hiPSC NPCs was an independent NPC gene signature set (Supplementary Data 1).

**Fig. 4:**
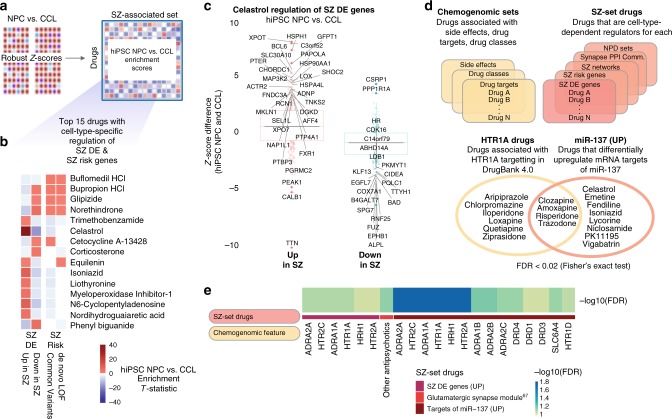
Differences in drug-induced responses between hiPSC NPCs and CCLs. **a** A gene-set enrichment approach was applied to identify drugs that regulated expression of SZ-set genes differently in profiles generated from hiPSC NPCs and CCLs. **b** Top 15 drugs with highest collective cell-type-specific perturbation of genes differentially expressed in prefrontal cortex of individuals with SZ (SZ DE), and/or genes harboring common and de novo SZ-risk-associated variants. Squares with enrichment FDR > 0.1 are shown as white. **c** Celastrol showed strong hiPSC NPC/CCL differential regulation of SZ DE genes (absolute Z-score difference > 3 labeled as solid filled circles with dark red or blue color). **d** To identify pharmacological features associated with drugs that differentially perturbed SZ sets in hiPSC NPCs compared with CCLs, we performed a chemogenomic enrichment analysis. SZ sets that were differentially perturbed by at least three drugs in the study (SZ-set drugs) were identified and enrichment testing was performed to identify over-represented drug targets, drug classes, and side effects. Drugs that differentially perturbed SZ sets between hiPSC NPCs and CCLs shared two pharmacological features: targeting of the serotonin receptor HTR1A and upregulation of the SZ-associated microRNA miR-137. **e** Chemogenomic features that were over-represented among drugs that differentially regulated various SZ sets depending on cell type (hiPSC NPC vs. CCL) context. Strong enrichment observed for drugs that differentially regulated SZ DE genes and targets of miR-137, particularly by antipsychotic drugs that targeted adrenergic, serotonergic, and dopaminergic neurotransmitter receptors (chemogenomic features). Abbreviations: SZ DE, genes differentially expressed in SZ; SZ Risk, genes harboring SZ-risk-associated loci; SZ Networks, SZ-associated coexpression networks; Synapse PPI Communities, NPD enriched synaptic protein seeded clique communities; and NPD Sets, diverse NPD-associated sets, e.g., miR-137 targets

In total, 129 drugs showed significant (false discovery rate (FDR) < 0.1) differential regulation of at least one SZ set between hiPSC NPCs and CCLs (Fig. 4, Supplementary Data 1). As many SZ sets were originally identified in the brain, we restricted the analyses to genes expressed in CCLs under vehicle-treated conditions (see Methods). We examined systematic differences in the transcriptomic profiles that were generated when profiling hiPSC NPCs compared with CCLs. Figure 4b shows the 15 drugs that induced the largest relative perturbations to SZ-risk loci genes and/or drug SZ-DE genes, dependent on cell line type. Celastrol was the drug with the strongest single cell-type-specific enrichment (NPC vs. CCL enrichment *T*-statistic: 2.79, FDR < 1.2e − 44, Fig. 4b, c, Supplementary Data 1). The observed effect included multiple HSP genes (consistent with evidence that celastrol induces HSP proteins in neurons^40^ as well as Fragile X Mental Retardation 1 (FMR1) (linked with autism spectrum disorder (ASD)^41^ and SZ^25^).

We performed a chemogenomic enrichment analysis to ask whether drugs that regulated SZ sets differently in hiPSC NPC and CCL contexts shared any pharmacological features (Fig. 4d; Methods; Supplementary Data 1). Drugs that differentially regulated each SZ set (FDR < 0.1) were grouped and compared across diverse chemogenomic annotations (see Methods). We identified two shared pharmacological features, targeting of the serotonin receptor HTR1A and upregulation of SZ-associated miR-137^3^ (Fig. 4e; Supplementary Figure 5a).

Taken together, multiple drugs induced perturbations of SZ sets differently in hiPSC NPCs and CCLs, including antipsychotics. The chemogenomic enrichments, although short of functional validation, reinforced this context dependence of drug perturbations. Overall, transcriptomic profiling of drug effects in SZ-relevant cell types better revealed disease signal.

### Diagnosis-dependent transcriptomic drug responses

SZ hiPSC NPCs showed a greater responsiveness to drug perturbations than control hiPSC NPCs (BF *p* < 0.002); both showed a greater responsiveness than CCLs (BF *p* < 0.001 all comparisons). Overall, drug-induced transcriptomic differences between SZ and control hiPSC NPCs were markedly less than observed between hiPSC NPCs and CCLs. Of the 47,510 DE results, 3394 show a differential DE result between SZ and control hiPSC NPCs.

**Fig. 5:**
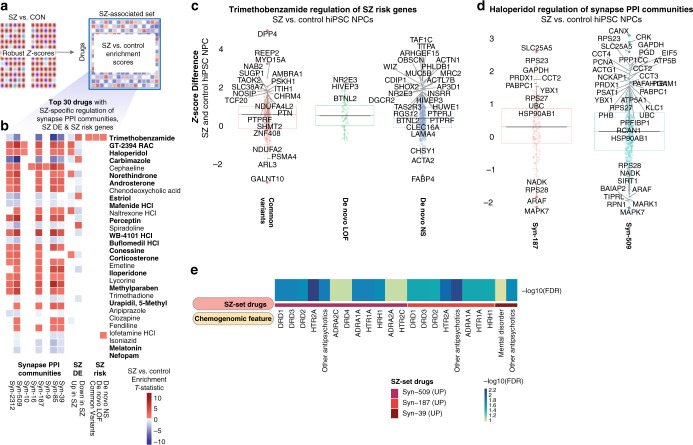
Drug-induced regulation of SZ biology varies between hiPSC NPCs derived from SZ patients compared with healthy controls. **a** A gene-set enrichment approach was applied to identify drugs that regulated expression of SZ-set genes differently in profiles generated on SZ and control hiPSC NPCs. **b** Top 30 drugs with highest collective cell-line type-specific perturbation of synaptic protein interaction clique (PPI) communities, genes differentially expressed in prefrontal cortex of individuals with SZ (SZ DE), and genes harboring common and de novo SZ-risk-associated variants. Squares with enrichment FDR > 0.1 are shown as white. Highlighted in bold are the 18/30 drugs that were independently identified as drugs that normalized SZ-related transcriptional signatures in Fig. 3c. **c** Trimethobenzamide shows strong SZ/control differential regulation of SZ-risk genes (Z-score difference > 1.5 labeled as solid filled circles with dark red, green or blue color). **d** Haloperidol shows strong SZ/control differential regulation of synaptic PPI communities (representative synaptic PPI communities, Z-score difference > 1.5 labeled as solid filled circles with dark red or blue color). **e** Strong enrichment observed for drugs that differentially regulated specific synaptic PPI communities (SZ-set drugs), particularly by antipsychotic drugs that targeted dopaminergic and serotoninergic neurotransmitter receptors (chemogenomic features)

One hundred and five drugs induced diagnosis-dependent (SZ vs. control hiPSC NPCs) differential gene expression changes (Supplementary Data 1; Fig. 5); 99 showed significant (FDR < 0.1) diagnosis-dependent differential regulation of at least one SZ set. Although glipizide was the drug with the single strongest diagnosis-specific enrichment (SZ vs. control hiPSC NPC enrichment *T*-statistic: 2.11, FDR < 1.6e − 12 (Supplementary Data 1)), trimethobenzamide showed the strongest enrichment over a range of SZ sets (synapse PPI communities, SZ DE, and SZ risk) (Fig. 5b, c, Supplementary Data 1). Critically, 18 of the top 30 drugs that were identified as having the most SZ-related transcriptional changes (Fig. 5b) also showed the best normalization of SZ-related transcriptional signatures (notably trimethobenzamide (Fig. 5c) in SZ hiPSC NPCs and haloperidol (Fig. 5d) in control hiPSC NPCs (Fig. 3c)).

To probe synaptic biology, we created synaptic PPI networks. Within these, we identified dense interacting sets of proteins (referred to as PPI clique communities), representing known pathways with higher coherence (Supplementary Table 2). Multiple drugs induced significant (FDR < 0.1) diagnosis-dependent perturbation of specific synapse PPI clique communities (Supplementary Data 1; top 30 such drugs shown in Fig. 5b), with enrichment for known antipsychotics (driven by aripiprazole, iloperidone, and risperidone) (Fig. 5e; Supplementary Figure 5b).

To confirm that these observed gene-set enrichments were not driven exclusively by imputed probes, we regenerated the SZ-set enrichments for each drug using only the landmark probe set. The enrichment *t*-statistics generated in a comparison of drug-induced responses between hiPSC NPCs and CCLs (shown in Fig. 4), and SZ and control hiPSC NPCs (shown in Fig. 5) were significantly positively correlated with statistics generated only from the landmark probe subset of the transcriptome (Corr: 0.55, Student’s *p*-value: 1.6e − 213 and Corr: 0.44, Student’s *p*-value: 9.4e − 136, respectively). Specifically the Synapse PPI Community set enrichments identified in SZ and control hiPSC NPCs (shown in Fig. 5b, d) were significantly positively correlated with landmark probe-set drug enrichment scores, most strongly for Syn-509 (Supplementary Figure 6). Taken together, these findings are supportive of the general accuracy of the enrichments generated using the full L1000 expression set.

**Fig. 6:**
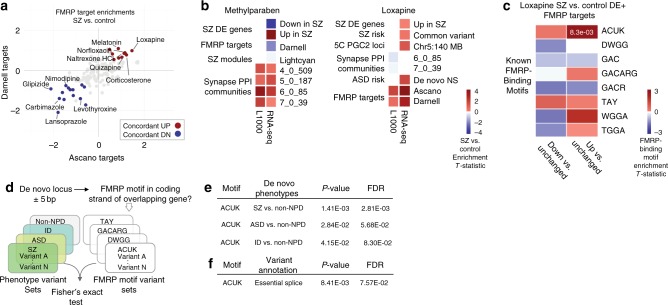
Loxapine-induced regulation of FMRP targets implicates NPD-associated binding motifs. **a** Summary of diagnosis-dependent drug-induced perturbations in *FMR1* protein (FMRP) targets from L1000 drug-screening data. Red (upregulation) and blue (downregulation) points indicate drugs that induce significant (FDR < 0.1) changes in the regulation of FMRP targets in SZ hiPSC NPCs in two FMRP target sets^42,43^. **b** SZ sets that are most differentially regulated by methylparaben and loxapine, across L1000 and RNA-seq. Squares with enrichment FDR > 0.1 are shown as white. **c** Known FMRP-binding motifs in DE genes of loxapine-treated SZ and control hiPSC NPCs. **d** FMRP targets enriched in loxapine-treated diagnosis-dependent hiPSC NPC DE genes were tested for association with SZ, ASD, and intellectual disability (ID) rare variant gene lists. **e** Enrichment for ACUK FMRP-binding motif variants identified in NPD mutations. **f** Enrichment for ACUK FMRP-binding motif variants identified in neuropsychiatric disease mutations with essential splice sites

We observed significant (FDR < 0.1) diagnosis-dependent changes (SZ vs. control hiPSC NPCs) in drug-induced perturbations in *FMR1* protein (FMRP) targets (Fig. 6a; Supplementary Figure 9a–c). Thirty-three percent (45/135) of drugs induced significant (FDR < 0.1) differential (21 increased, 24 decreased) and 13% (18/135) induced significant (FDR < 0.1) concordant (6 increased, 12 decreased) expression changes in both of two independent FMRP targets sets^42,43^. The antipsychotic loxapine, a drug that we previously reported to impact key SZ-associated cellular and molecular alterations^12^, induced the largest increase in FMRP target expression (FMRP target enrichments: NPC SZ vs. control enrichments, Ascano Targets: *T*-statistic: 0.99, FDR < 8.1e − 6, Darnell Targets: *T*-statistic: 0.99, FDR < 0.0024, Supplementary Data 1). This suggested altered regulation of FMRP targets in the context of SZ hiPSC NPCs and may point to FMRP activity as a potential point of mechanistic convergence between the drugs tested.

### FMRP targets and differential transcriptomic drug responses

To confirm the observed diagnosis-dependent impact of select drugs on FMRP targets, we employed RNA-sequencing (RNA-seq), which is a higher fidelity platform. We selected two L1000-flagged drugs with dissimilar FMRP target responses, loxapine and methylparaben, for validation across two hiPSC NPC lines from three SZ and three controls each (Methods; Supplementary Table 1; Supplementary Figure 7). As with the L1000 data, variance partition analysis demonstrated that transformation of expression to RZS from isogenic comparisons dramatically reduced variation attributed to the cell line and donor variables (Supplementary Figure 2b). Moreover, RNA-seq confirmed dissimilar FMRP target responses to these two drugs; loxapine, but not methylparaben (see Supplementary Figure 8), increased FMRP target levels in two independent FMRP targets sets^42,43^ (Fig. 6b).

FMRP acts through negative regulation of mRNA translation at polyribosomes, although roles for RNA folding, subcellular trafficking, and regulation of microRNA-mediated translational repression are also reported (reviewed^44^). We tested whether the genes driving SZ hiPSC NPC-specific differences in loxapine-induced perturbation of FMRP targets were associated with any particular binding motifs. Eight nucleotide sequence patterns associated with FMRP binding^45^ were evaluated for differences in motif density between FMRP targets differentially perturbed in the loxapine experiments (Fig. 6c). Perturbed FMRP targets were enriched for the ACUK motif (*T*-statistic: 2.48, *p*-value: 0.013, two-sample *T*-test), an enrichment driven by the upregulated targets (relative to unchanged targets) (*T*-statistic: 2.65, *p*-value: 0.0083, two-sample *T*-test).

Given the association of FMRP target genes to NPD risk^24,25^, we hypothesized that these ACUK motifs might correspond to sites of NPD-associated genetic variation. We evaluated coding and splice-associated DNA variants (collected and annotated by ref. ^25^), from individuals with SZ (918 variants total), ASD (1044 variants total), Intellectual Disability (166 variants total), and controls (591 variants total). For each variant, we identified occurrences of FMRP-binding motifs (Fig. 6d); the ACUK motif was enriched among de novo variants reported in SZ (Fisher’s exact test, FDR < 0.0028), ASD (Fisher’s exact test, FDR < 0.057), and ID (Fisher’s exact test, FDR < 0.083) (Fig. 6e), particularly at essential splice sites (Fisher’s exact test, FDR < 0.076). Therefore, although identified in the context of FMRP binding^43^, the ACUK motif may also regulate mRNA splicing (Fig. 6f) within de novo variants associated with NPD (Supplementary Figure 9d). These results highlight the FMRP-binding motif ACUK as a novel therapeutic point of intervention and mechanism of SZ and provide an example of a SZ-relevant transcriptomic-based insight that could not have emerged from a study of CCLs alone.

## Discussion

Here we provide a resource of 4320 transcriptional signatures generated from hiPSC NPCs (derived from 12 SZ cases and 12 controls) and 8 CCLs treated with 135 SZ-relevant drugs. By combining two emerging technologies, hiPSC-based models with in silico drug-screening methodologies, we established the feasibility of transcriptomic-based drug screens of patient-derived neural cells. Drug-induced perturbations were overall very similar between hiPSC NPCs and CCLs; however, when specifically considering differential drug-induced perturbations in SZ hiPSC NPCs, relative to control hiPSC NPCs and particularly CCLs, select drugs induced differential responses in subsets of genes, and those differentially impacted gene sets were enriched for SZ biology. Although many drug–gene perturbations were shared, there were important differences between cell types. While more drugs showed larger differences in drug-induced perturbations between hiPSC NPCs and CCLs than between hiPSC NPC cell line groups, surprisingly, CCLs were overall less drug responsive than either SZ or control hiPSC NPCs. Our data suggests that inclusion of patient-derived neural cell lines will enrich the results for transcriptomic responses relevant to disease processes. Importantly, we identified drugs capable of ameliorating a SZ-related transcriptional signature in hiPSC NPCs; the genes differentially impacted by many of these drugs (i.e., trimethobenzamide, loxapine) specifically enriched for SZ biology in our subsequent analyses. Our ability to independently identify a common set of 18 drugs that both reversed SZ signatures in hiPSC NPCs (Fig. 3c) and differentially regulated SZ-set genes in SZ hiPSC NPCs (Fig. 5b) supports the validity of our transcriptomic drug-screening approach. Although our SZ-signature and SZ-set analyses identified groups of genes differentially regulated by select drugs on a cell type (hiPSC NPC vs. CCL) or diagnosis (SZ vs. control)-specific basis, it was by chemogenomic analysis that we identified hypotheses as to why these differential effects occurred.

The use of hiPSC-derived NPCs and neurons has already identified novel targets for neurodegenerative disease such as ALS^46,47^, Parkinson’s disease^48^, Wolfram Syndrome^48^, and neurotoxicity^49^; mitochondrial risk effects can also be modeled and screened for with hiPSCs NPCs^50^. However, to the best of our knowledge, our work presents one of the first studies using hiPSC NPCs at this scale to support the discovery of novel biology and therapeutic targets in SZ. The next steps to further the use of hiPSC NPCs in NPD must include rigorous reduction of the sources of biological and technical variation (particularly those related to miniaturization to enable high-throughput screening) to improve the reproducibility of data and full expression profiling to increase the strength of donor transcriptional signatures^18,51^. Given the resemblance of hiPSC NPCs and neurons to fetal tissue^52–56^, it remains to be investigated how the targets or drug candidates identified through this approach could be provided clinically (e.g., preventive treatments in high-risk individuals, early intervention, or symptomatic treatment of established or chronic SZ).

Although repeated genetic studies of SZ have identified enrichment for rare mutations that impact synaptic proteins and FMRP targets^24,25,57,58^, FMRP is not yet a focus of SZ therapeutic discovery. Future work might benefit from an adaptation of an existing *FMR1-Nluc* reporter high throughput assay in hiPSC NPCs^59^. A major function of FMRP is to stall ribosomal translocation on its target mRNAs, suggesting that compounds that inhibit translation elongation might alleviate neuronal phenotypes associated with FMRP (reviewed^60^; critically, many antibiotics act by stalling the translocation of bacterial ribosomes and were predicted to reverse SZ gene signatures in our drug repurposing analyses (Supplementary Table 1). Consistent with this, the antibiotic minocycline can reverse abnormal synaptic structure and behaviors exhibited by *Fmr1*-knockout mice^61^ and *dfmr1*-null flies^62^.

Importantly, continued quality control of data and use of orthogonal assays is important when applying transcriptomic technologies; L1000 and RNA-seq median platform self-correlation across CCLs and >3000 tissues was reported to be 0.84^33,63^. Here, our RNA-seq experiments do not specifically address the fidelity of the L1000 platform, as they were conducted on independently cultured and drug-treated hiPSC NPCs (with the addition of a second independent NPC line differentiated from an independent clonal hiPSC line), using different batches of all critical reagents, reflecting both biological as well as technical effects. In addition, the L1000 platform is largely designed to query signature level changes rather than examine gene-specific differences. However, its cost-effectiveness, when coupled with a gene-set enrichment analysis approach, allowed us to profile a substantially larger number of drugs than would have otherwise been feasible using a higher fidelity platform.

In conclusion, consistent with work by Subramanian et al.^33^, this study demonstrates the feasibility of large scale drug screening using a transcriptomic readout. By projecting our transcriptomic signatures onto biologically diverse, SZ-associated genomics datasets, our approach has the potential to significantly improve the success rate of NPD drug discovery. As sequencing costs continue to drop, RNA-seq based screening^64^ should be substituted in order to capture specific drug-induced changes at the gene and isoform level. Integration of high-content imaging^65^ and proteomic^66^ readouts will further facilitate annotation of molecular and cellular response to drug perturbations. Furthermore, and along the lines of integrating multiple layers of data, network models will be necessary to capture large ensembles of gene loci and variants associated with disease. Ultimately, multiscale biology approaches that integrate pre-clinical, clinical, literature, and imaging data will be required to construct predictive disease network models and advance target and drug discovery^21^.

## Methods

### CCLs and hiPSC NPCs

CCLs used in this study were purchased from ATCC: HT29 (Catalog Number HTB38), A549 (Catalog Number CCL-185), VCaP (Catalog Number CRL-2876), MCF7 (Catalog Number HTB-22), AGS (Catalog Number CRL-1739), A673 (Catalog Number CRL-1598), HepG2 (Catalog Number HB-8065), and SH-SY5Y cells (Catalog Number CRL-2266). NPCs used in this study were obtained from two sources. For cohort #1, four patients and five control hiPSCs were reprogrammed from fibroblasts obtained from Coriell or ATCC^12,55^. For cohort #2, 13 child-onset SZ patients and 13 control hiPSCs were reprogrammed from fibroblasts obtained from NIH^15,18^. All hiPSCs were differentiated to hiPSC NPCs between passages 10 and 20 through an embyroid body-based strategy^12,15,18^; all hiPSC NPCs were used for drug screening between passages 5 and 10. The cell lines used in each experiment, as well as available technical and clinical information, are available in Supplementary Table 3.

For CCLs, fetal bovine serum (FBS, Catalog Number 16000-044, Lot Number 1551835), penicillin streptomycin (P/S, Catalog Number 15140-122, Lot Number 1523694), and culture media were from ThermoFisher Scientific, unless noted otherwise. Growth media for all cancer cells contained 10% FBS and 100 U mL^−1^ of P/S. HT29 cells grew in McCoy’s 5A (Catalog Number 16600-082, Lot Number 1459946); A549 cells grew in F-12K (Catalog Number 21127-022, Lot Number 1550730); VCaP cells grew in Dulbecco’s modified Eagle’s medium (DMEM) (Catalog Number 11995-040, Lot Number 1550730); MCF7 cells grew in EMEM (ATCC, Catalog Number 30-2003, Lot Number 61756726) containing 10 ng mL^−1^ insulin (Sigma, Catalog Number I9978-5ML, Lot Number 1374942); AGS cells grew in F-12K; A673 cells grew in DMEM; HepG2 grew in EMEM, and SH-SY5Y cells grew in 1:1 F-12K and EMEM. CCLs were expanded according to vendor’s instructions, tested for mycoplasma, and banked (within five passages) before experiments. Cell lines used in all experiments were from the same lot of a given banked passage for the cell line. Seeding cell density was adjusted to ensure 70% cell confluence for all CCLs at the initiation of drug treatment.

For hiPSC NPCs, growth media used was DMEM/F12 + GlutaMAX™ -I (ThermoFisher Scientific, Catalog Number 10565, Lot Number 1715888) containing 1 × N2 (ThermoFisher Scientific, Catalog Number 17502-048, Lot Number 1672893), 1 × B27-RA (ThermoFisher Scientific, Catalog Number 12587-010, Lot Number 1731195), and 20 ng ml^−1^ FGF2 (R&D Systems, Catalog Number 233-FB, Lot Number HKW12815061). Accutase for cell dissociation was from Innovative Cell Technologies (Catalog Number AT-104, Lot Number 5S2415A). Cyto One (USA Scientific) and Corning (Fisher Scientific) culture plasticware was used for expansion and plating of cells. hiPSC NPCs were grown on Matrigel (BD Biosciences, Catalog Number 354230, Lot Number 3018665)-coated plates in NPC media. NPC experiments were conducted on myocoplasma-free passage-matched populations, generally between passages five and seven.

After plating, all cell lines were incubated overnight before starting the drug treatments.

### Drug prioritization

We compared hiPSC NPCs (12 SZ and 12 control) and CCLs (8) using 135 small molecule perturbagens (referred to as drugs in this manuscript), generating over 4,300 unique signatures. Drugs were prioritized based on known or predicted connections with diverse SZ biology (Supplementary Table 1). We first collated (i) SZ transcriptomic, genetic, and protein-centric data [including SZ post-mortem brain gene expression^23^, (SZ DE genes), gene coexpression modules reported as differentially activated in SZ post-mortem brain tissue^67^ (SZ networks), SZ-risk gene sets based on common^3^ and rare variants^24,25^ (SZ-risk genes), and synaptic protein PPI communities generated from subsetting human protein interaction data] with (ii) synapse-specific gene sets^26^ filtered on NPD-associated gene sets (Supplementary Table 2) (Synapse PPI communities). Next, we applied two methods to select the drugs: (1) a connectivity mapping approach^27^ prioritized drugs according to their predicted ability to differentially regulate the transcription of each SZ set and (2) an enrichment analysis incorporating referenced (DrugBank 4.1^28^) and predicted (SEA^29^) drug-target associations with targets that are enriched among compounds that regulate each SZ set. We prioritized 75 drugs by method 1 and the remainder by method 2, from a total of 6269 drugs (Fig. 1).

### Drug screening

Drugs tested in this study were provided by Eli Lilly as 10 mM stocks in DMSO, in heat-sealed 96-well plates or purchased from Tocris and dissolved in DMSO (Sigma Catalog Number D5879, Lot Number SHBF7682V). HP D300 T8 cassettes for pilot experiment were from Tecan. All other automated liquid handling consumables, experiments in stages 1 and 2, were from Perkin Elmer. Concentration of each drug was selected by accounting for concentrations calculated from ChEMBL (https://www.ebi.ac.uk/chembl/) and Lilly-generated internal data, to identify for each compound, the highest affinity target available in the databases, and concentrations from The Connectivity Map (CMap)^27,33^. If the calculated concentration was lower than CMap^27^, the calculated concentration was used. If the calculated concentration exceeded that in CMap, then the CMap concentration was used. If either concentration exceeded 10 µM, the test concentration was capped at 10 µM (Supplementary Table 4).

In a pilot experiment, we performed a procedural dry run using 2 CCLs (SH-SY5Y and A549), 2 NPCs (1 SZ and 1 control), 5 pairs of agonists and antagonists (to D2R, mGluR2/3, NMDAR, 5HT2A, and Ca(V)1) at 2 concentrations each (20 test wells), and a combination of 1 concentration of antagonist in the presence of 2 concentrations of an agonist (10 test wells) (Supplementary Table 3). HP D300 digital dispenser (Integrated Screening Core at Mount Sinai) was used to administer test compounds and vehicle (DMSO) to designated wells. DMSO was normalized to 0.2%. Total of 30 test conditions, 12 vehicles, 2 positive controls (duplicate), and 1 empty well were used per cell line, per time point (6 and 18 h) (Supplementary Table 4). Eight half 96-well plates were used for 6- and eight for 18 h time point. The resulting cell lysates were assembled onto a single 384-well plate as per Genometry’s instructions for L1000 assay. Based on the strong concordance in drug signatures between the 6 and 18 h time points in the pilot analysis, particularly at the higher drug concentrations (capped at 10 μM), a 6 h treatment at the higher dose range (Supplementary Table 4) was selected for the larger study.

In stage 1 and stage 2 experiments, we used automated screening. Twenty-four hiPSC-derived NPCs and 8 CCLs were used (total: 32 cell lines (Supplementary Table 3)). Of the 24 NPC lines tested, three were replaced in phase 2 for technical reasons, resulting in 21/24 NPC lines being tested in both phases and 3/24 tested only in phase 2. All eight CCLs were tested in both phases without replacement (Fig. 1). Each 96-well plate included the following: A1 empty well, 12 vehicle wells, and 2 positive control wells (in duplicate) per cell line (i.e., per plate). The remaining 79 wells were used for test compounds: (a) phase 1 had 6/79 drugs from pilot and 73/79 phase 1 test compounds, (b) phase 2 had 6 pilot drugs re-tested, 6/73 phase 1 drugs re-tested, and 67/79 phase 2 compounds. One hundred and thirty-five unique compounds were tested (Supplementary Table 4).

High-throughput automated screening equipment included JANUS MDT (Perkin Elmer) with integrated plate stack towers, incubator, gripper arm, and a 96 pin tool (VP Scientific, P/N 70229750), which was used to add all drugs. Cell Explorer (Perkin Elmer) was used to remove media, lyse cells, and fill 384-well plates for downstream processing by Genometry. Both systems were enclosed and equipped with Hepa filters. Briefly, cells were loaded in JANUS MDT incubator. Working plates of 1000 × test drugs in DMSO were used to pin drugs into each 96-well plate containing cells. Cells were returned to the incubator for 6 h and then lysed with cell lysis buffer (Genometry; 110 µL per well) (and stored at − 80 °C). Eight 96-well cell plates were processed per day (days 1–4). At the end of experiments (day 5), 96-well plates were thawed at RT and 50 µL of lysate transferred into each of the two daughter 384-well plates (four 96-well plates were combined per one 384-well plate; yielding a total of eight 384-well plates for phase 1 and another eight plates for phase 2). One 384-well plate was analyzed by Genometry and one stored at − 80 °C as backup (Supplementary Figure 1).

### Expression profiling, pre-processing, and quality control

The drug-screening experiments were designed to minimize the impact of inter-cell line (hiPSC NPC and CCL) and inter-batch variability in confounding the detection of drug effects^27,34^. Each drug perturbation was generated alongside cell-matched, DMSO-treated controls within the same treatment plate; each cell line therefore acted as its own control, minimizing the effects of heterogeneity across individuals and clonal hiPSC populations (Supplementary Figure 1). Drug-induced gene expression profiles were generated using the L1000 platform^33^ cost-effective genome-wide microarray assay based on Luminex bead technology that directly measures the expression of 978 landmark probes, in order to impute probe expression values to the full Affymetrix (HgU133A) probe space (22,268 probes)^31,32^. The L1000 gene-expression profiling assay (Genometry, Inc.) was used to generate data; GCT files with intra-sample scaled, intra-batch quantile-normalized, and log2-transformed data were received and used for downstream analysis. The imputed probe set was converted to an expression matrix corresponding to probes mapping to unique Entrez gene identifiers. Entrez gene identifiers with multiple corresponding probes were collapsed by retaining the probe with the highest expression across drug signatures from all samples, totaling 12,500 unique Entrez gene identifiers. Overall, normalized probe expression from 22,268 (978 landmark and 21,290 inferred) probes was collapsed to an expression matrix corresponding to 12,500 unique Entrez gene identifiers, retaining probes with the highest expression across all samples^68^.

Drug signatures were generated by transforming gene expression to a RZS, which reflects comparison with cell-matched, DMSO-treated wells within each plate to minimize the effects of inter-batch variability as described in refs. ^31–33^. *Z*-score signatures for drugs that were assayed multiple times on a given cell were collapsed to the median expression value for each gene, and the following equation used to generated the score:

$${\rm Robust}\,Z_{gene} = \frac{{Treated\,{{\rm experssion}} - Median\,DMSO\,{{\rm experssion}}}}{{\rm Median\,absolute\,deviation}}$$

Drug signatures were quantified by transforming normalized gene expression to a RZS^34^ reflecting a comparison between cell-line matched, DMSO-treated wells within each plate. For the minority of drugs that were assayed multiple times on a given cell type, RZS signatures were summarized to the median value for each probe (Fig. 1).

Drug–gene perturbations were classified as DE if the absolute median RZS was ≥ 2 (Supplementary Data 1). A drug–gene perturbation was classified as differentially DE between two cell types expressed if the absolute difference in median RZS was ≥ 2. Proportions of DE drug–gene perturbations in each cell type were compared by Mantel–Haenszel test stratifying by drug–gene perturbation^69^ and *p*-values were corrected for multiple comparisons using the Bonferroni method. Each gene was scored according to the number of drugs for which it was DE.

Drug enrichments for SZ sets were generated using the R package GAGE (30) to identify individual SZ sets that are differentially perturbed between groups under comparison (i.e., NPC vs. CCL or SZ NPC vs. control NPC). For each drug, we iterated over each SZ-set type and compared the transcriptome-wide matrices of RZS between groups under consideration using an unpaired, two-sample *t*-test, as implemented in the *gage* function. Raw enrichment *p*-values were adjusted automatically by the software, using a *Q*-value to estimate the FDR.

We estimated an appropriate baseline gene expression background for the CCL using a publicly available library of CCL RNA-seq profiles^70^. This includes profiles for six of our eight CCLs (A549, A673, AGS, HEPG2, HT29, and MCF7). We identified exactly 16,000 unique genes, with at least 10 mapped reads, in at least half of the cell lines under consideration and classified these as an estimated background for subsequent gene-set enrichment testing of our profiled CCLs in the context of brain-derived SZ sets.

### Chemogenomic enrichment analysis

We grouped drugs that differentially regulate each SZ set (FDR < 0.1), and then annotated them using diverse chemogenomic data, including drug target, enzyme, transporter, and carrier information from (DrugBank 4.1^28^) structure-based drug-target predictions (SEA^29^), drug therapeutic classes (ATC^71^), and side effects (SIDER^72^ and OFFSIDES^73^). For each chemogenomic class of interest, we identified the relevant subset of compounds (for instance, side effects are only meaningful for compounds that have been used clinically), generating custom compound backgrounds for further enrichment testing. For each chemogenomic feature, we considered the unique set of compounds that were associated with each SZ set and calculated chemogenomic enrichments using Fisher’s exact text, and one-sided *p*-values (to identify over-representation of compounds) were adjusted using the Benjamini–Hochberg method^74^.

### RNA-seq validation

NPCs were treated for 6 h with equivalent volumes of DMSO, methylparaben (10 μM), or loxapine (1 μM) diluted into NPC media. Total RNA was purified using the RNeasy Plus Mini Kit (Qiagen) and eluted in water. RNA libraries were prepared for sequencing using standard Illumina protocols (Ribo-Zero) and sequenced at ISMMS using a Hi-Seq 2500 using pair-end 100 nt reads, 8 samples per lane.

RNA-seq experiments were conducted after L1000 drug screening and data analyses were completed, capturing both biological (independent cell culture and RNA purification) and technical variation (independent transcriptomic analysis) (Supplementary Figure 7). RNA-seq data were normalized using the voom function^75^, retaining genes with at least 1 count per million mapped reads, in at least 5 samples, resulting in 14,931 unique genes. L1000 data was restricted to DMSO-, methylparaben-, or loxapine-treated hiPSC NPCs derived from the ten individuals collectively included in the RNA-seq. Normalized gene expression was merged across platforms, resulting in an expression matrix comprising the 9680 unique genes present in both datasets. The mean pairwise Spearman’s correlation across platforms from samples derived from the each drug were generally high and positive (mean cross-platform Spearman’s *ρ*, DMSO = 0.54, loxapine = 0.55, and methylparaben = 0.55), although samples clustered most strongly by platform, both when considering the full set of genes (Supplementary Figure 7a) or when subsetting to the only the genes corresponding to the landmark probes (Supplementary Figure 7b) (Median *ρ*, landmark = 0.43, inferred = 0.19, all = 0.3).

## Electronic supplementary material

Supplementary Information

Description of Additional Supplementary Files

Supplementary Data 1

Supplementary Data 2

## Data Availability

All L1000 and RNA-seq data are available at the Gene Expression Omnibus (GEO) series GSE119291, L1000 data under GSE119275, and RNA-seq data under GSE119290. hiPSC lines have been deposited at the NIMH Stem Cell Center maintained by Rutgers University Cell and DNA Repository (RUCDR); CCLs were obtained from and available at ATCC.
