# Author Correction: Expression-based drug screening of neural progenitor cells from individuals with schizophrenia

**DOI:** 10.1038/s41467-018-07326-3

**Published:** 2018-11-19

**Authors:** Benjamin Readhead, Brigham J. Hartley, Brian J. Eastwood, David A. Collier, David Evans, Richard Farias, Ching He, Gabriel Hoffman, Pamela Sklar, Joel T. Dudley, Eric E. Schadt, Radoslav Savić, Kristen J. Brennand

**Affiliations:** 1 Department of Genetics and Genomic Sciences, Icahn School of Medicine at Mount Sinai, New York, NY 10029 USA; 2 Icahn Institute for Genomics and Multiscale Biology, Icahn School of Medicine at Mount Sinai, New York, NY 10029 USA; 3 Institute for Next Generation Healthcare, Icahn School of Medicine at Mount Sinai, New York, NY 10029 USA; 4 Department of Neuroscience, Icahn School of Medicine at Mount Sinai, New York, NY 10029 USA; 5 Friedman Brain Institute, Icahn School of Medicine at Mount Sinai, New York, NY 10029 USA; 6 Eli Lilly and Company Ltd, Erl Wood Manor, Surrey, UK; 7 Social, Genetic and Developmental Psychiatry Centre, Institute of Psychiatry, King’s College London, London, UK; 8 Department of Psychiatry, Icahn School of Medicine at Mount Sinai, New York, NY 10029 USA; 9 Sema4, a Mount Sinai venture, Stamford, Connecticut USA; 10 Present Address: ASU-Banner Neurodegenerative Disease Research Center, Arizona State University, Tempe, AZ 85287-5001 USA

Correction to: *Nature Communications*; 10.1038/s41467-018-06515-4; published online 24 Oct 2018

In the originally published version of this Article, the affiliation details for Eric E. Schadt and Radoslav Savic incorrectly omitted ‘Sema4, a Mount Sinai venture, Stamford, Connecticut, USA’. This has been corrected in both the PDF and HTML versions of the Article.

