# Supplementary material for: Expression-based drug screening of neural progenitor cells from individuals with schizophrenia
Source: Nat Commun. 2018 Oct 24;9:4412. doi: 10.1038/s41467-018-06515-4 (PMC6200740; doi:10.1038/s41467-018-06515-4)
Supplement: Supplementary file 1 — Supplementary Information [file 41467_2018_6515_MOESM1_ESM.pdf]

Supplementary Information

Expression-based drug screening of neural progenitor cells from individuals with schizophrenia

Readhead et al

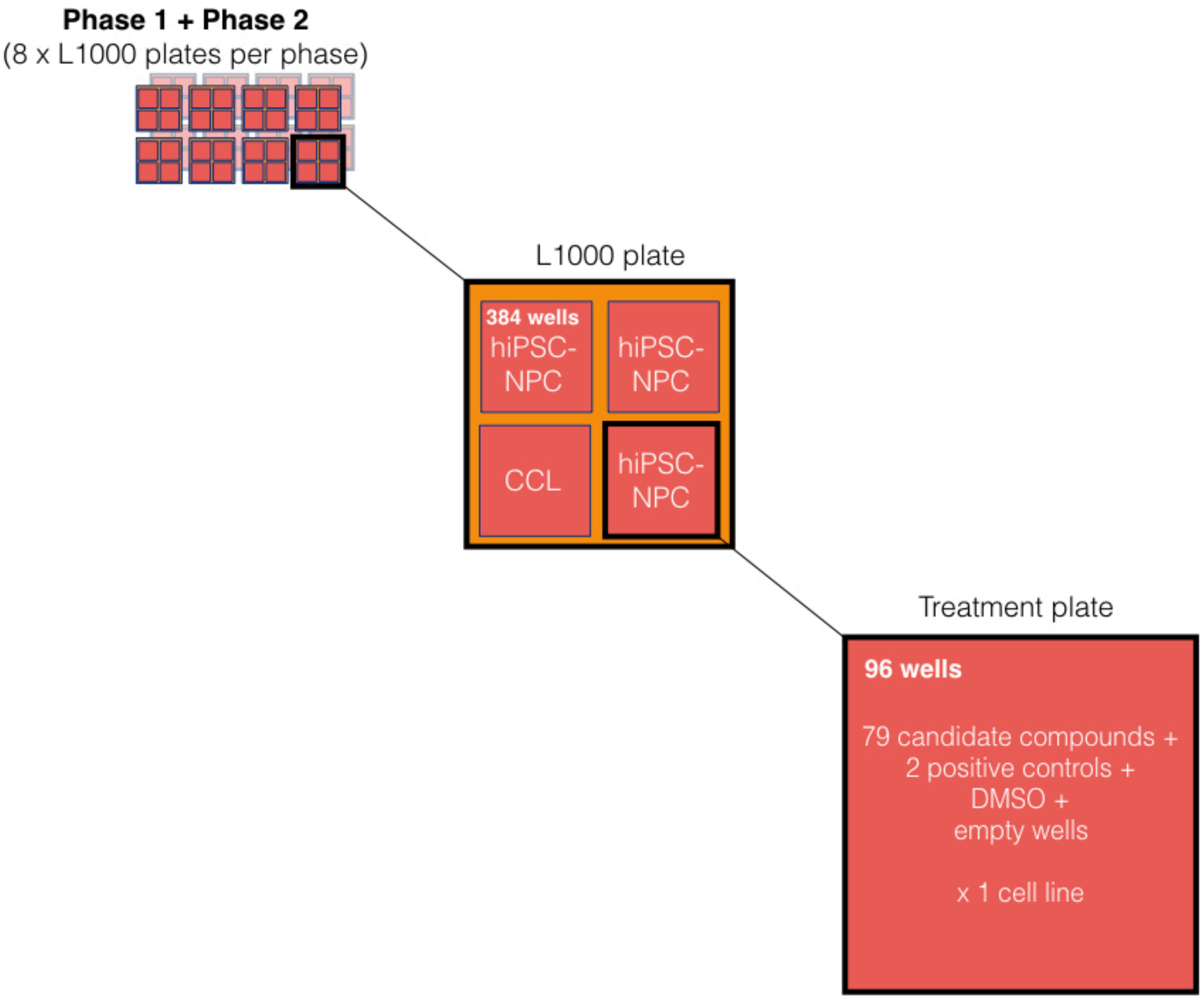

Supplementary Figure 1. Schematic of tissue culture and L1000 plate design for two phases of drug screening experiments.

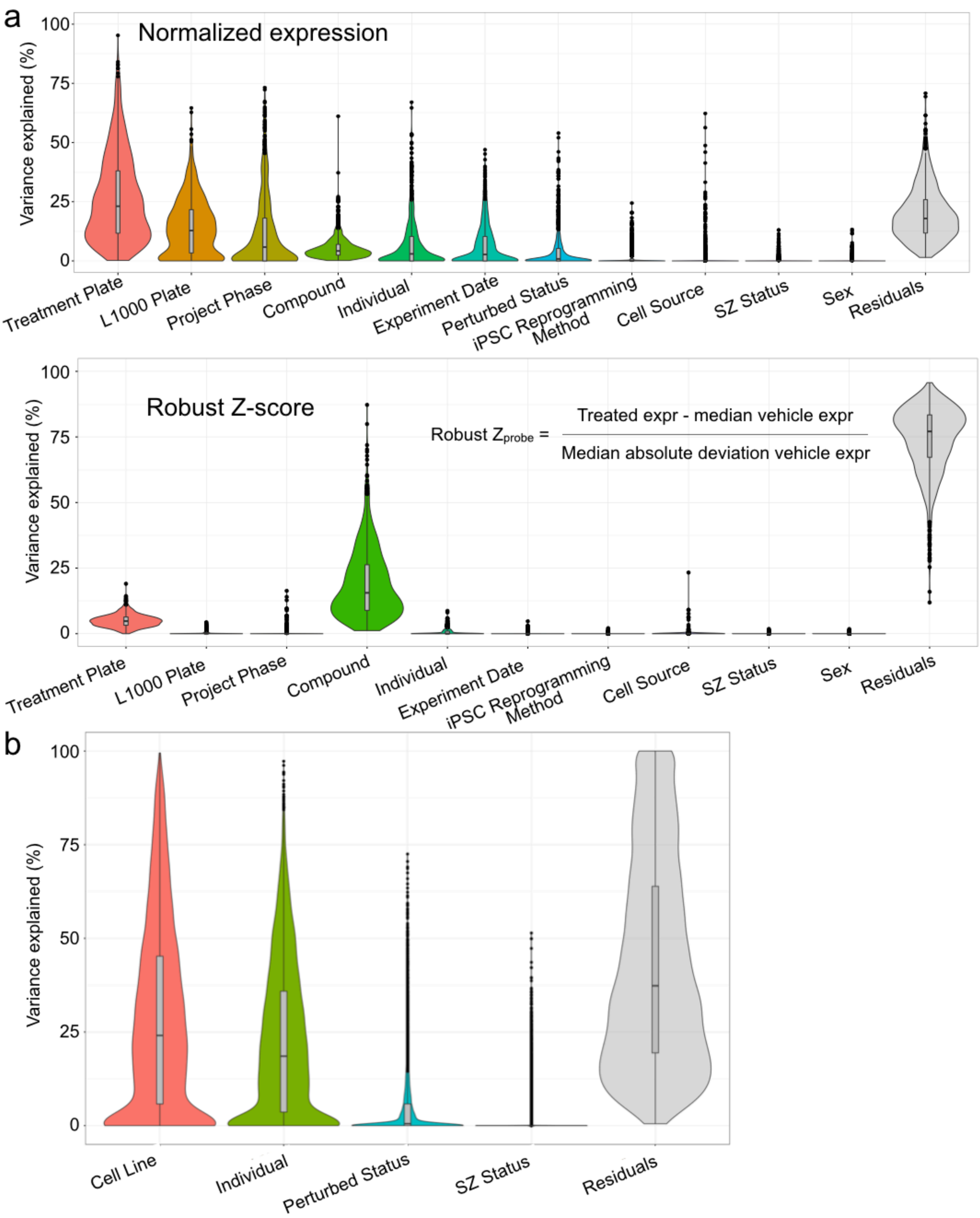

Supplementary Figure 2. Variance partition of L1000 and RNA-seq transcriptomic data. (a) Variance partition of L1000 transcriptomic data before (top, normalized gene expression) and after

(bottom, robust Z-score) normalization by comparison with isogenic within-plate DMSO experiments. (b) Variance partition RNA-seq transcriptomic data before conversion to isogenic comparisons.

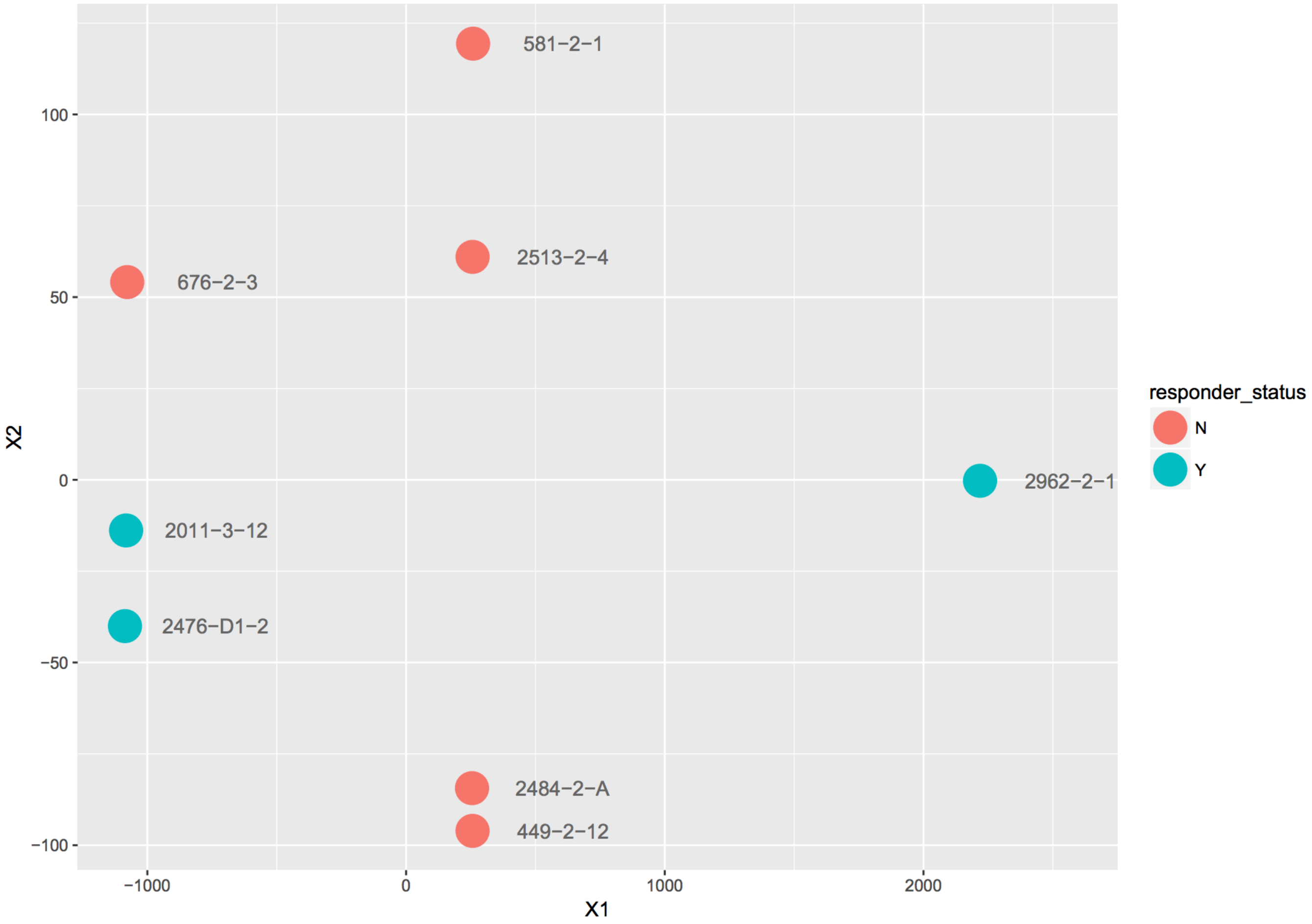

Supplementary Figure 3. Clozapine induced gene expression among hiPSC NPCs derived from individuals with childhood onset schizophrenia.

Multidimensional scaling plot of clozapine induced gene expression changes, stratified according to known clozapine response.

a **Frequently perturbed CCL genes**

	Probe	Type	Symbol	Name	# Drugs
Up	207546_at	INF	ATP4B	ATPase, H+/K+ exchanging, beta polypeptide	15
	37028_at	INF	PPP1R15	protein phosphatase 1, regulatory subunit 15A	12
	202679_at	LM	NPC1	Niemann-Pick disease, type C1	12
	204420_at	LM	FOSL1	FOS-like antigen 1	12
	213112_s_at	INF	SQSTM1	sequestosome 1	12
	206513_at	INF	AIM2	absent in melanoma 2	12
	205991_s_at	INF	PRRX1	paired related homeobox 1	11
	201466_s_at	LM	JUN	jun proto-oncogene	11
	206461_x_at	INF	MT1H	metallothionein 1H	11
	202672_s_at	INF	ATF3	activating transcription factor 3	11
	205281_s_at	INF	PIGA	phosphatidylinositol glycan anchor biosynthesis, class A	11
	203455_s_at	INF	SAT1	spermidine/spermine N1-acetyltransferase 1	11
	205791_x_at	INF	ZNF230	zinc finger protein 230	11
	209383_at	INF	DDIT3	DNA-damage-inducible transcript 3	10
	201693_s_at	LM	EGR1	early growth response 1	10
Down	218755_at	LM	KIF20A	kinesin family member 20A	11
	213599_at	INF	OIP5	Opa interacting protein 5	11
	201555_at	LM	MCM3	minichromosome maintenance complex component 3	11
	201051_at	INF	ANP32A	acidic (leucine-rich) nuclear phosphoprotein 32 family, member A	11
	203188_at	LM	B3GNT1	UDP-GlcNAc:betaGal beta-1,3-N-acetylglucosaminyltransferase 1	9
	202534_x_at	INF	DHFR	dihydrofolate reductase	9
	212320_at	INF	TUBB	tubulin, beta class I	9
	201609_x_at	INF	ICMT	isoprenylcysteine carboxyl methyltransferase	9
	203871_at	INF	SENP3	SUMO1/sentrin/SMT3 specific peptidase 3	9
	200768_s_at	LM	MAT2A	methionine adenosyltransferase II, alpha	9
	201890_at	INF	RRM2	ribonucleotide reductase M2	9
	211784_s_at	INF	SRSF1	serine/arginine-rich splicing factor 1	9
	202330_s_at	INF	UNG	uracil-DNA glycosylase	9
	202705_at	LM	CCNB2	cyclin B2	9
	201774_s_at	LM	NCAPD2	non-SMC condensin I complex, subunit D2	9

b **Frequently perturbed hiPSC NPC genes**

	Probe	Type	Symbol	Name	# Drugs
Up	202431_s_at	LM	MYC	v-myc avian myelocytomatosis viral oncogene homolog	12
	201693_s_at	LM	EGR1	early growth response 1	11
	37028_at	INF	PPP1R1	protein phosphatase 1, regulatory subunit 15A	11
	201466_s_at	LM	JUN	jun proto-oncogene	11
	202672_s_at	INF	ATF3	activating transcription factor 3	11
	202130_at	INF	RIOK3	RIO kinase 3	11
	202081_at	INF	IER2	immediate early response 2	11
	201536_at	LM	DUSP3	dual specificity phosphatase 3	10
	205249_at	INF	EGR2	early growth response 2	10
	202768_at	INF	FOSB	FBJ murine osteosarcoma viral oncogene homolog B	10
	202340_x_at	INF	NR4A1	nuclear receptor subfamily 4, group A, member 1	10
	202627_s_at	LM	SERPIN	serpin peptidase inhibitor, clade E (nexin, plasminogen activator inhibitor	10
	201531_at	LM	ZFP36	ZFP36 ring finger protein	10
	204420_at	LM	FOSL1	FOS-like antigen 1	10
	212614_at	LM	ARID5B	AT rich interactive domain 5B (MRF1-like)	10
Down	202954_at	LM	UBE2C	ubiquitin-conjugating enzyme E2C	12
	203276_at	INF	LMNB1	lamin B1	11
	201051_at	INF	ANP32A	acidic (leucine-rich) nuclear phosphoprotein 32 family, member A	11
	202743_at	LM	PIK3R3	phosphoinositide-3-kinase, regulatory subunit 3 (gamma)	11
	206102_at	INF	GINS1	GINS complex subunit 1 (Psf1 homolog)	11
	209172_s_at	INF	CENPF	centromere protein F, 350/400kDa	11
	210950_s_at	INF	FDFT1	farnesyl-diphosphate farnesyltransferase 1	10
	202580_x_at	INF	FOXM1	forkhead box M1	10
	212126_at	INF	CBX5	chromobox homolog 5	10
	202540_s_at	LM	HMGCR	3-hydroxy-3-methylglutaryl-CoA reductase	10
	201555_at	LM	MCM3	minichromosome maintenance complex component 3	10
	209218_at	INF	SQLE	squalene epoxidase	10
	213599_at	INF	OIP5	Opa interacting protein 5	9
	201791_s_at	INF	DHCR7	7-dehydrocholesterol reductase	9
	202534_x_at	INF	DHFR	dihydrofolate reductase	9

c **Frequently perturbed CCL gene sets**

Hallmark gene set enrichments

	Gene Set	FDR	Fold Change
Up	TNFA SIGNALING VIA NFKB	1.01E-48	7.81
	HYPOXIA	1.33E-08	3.39
	P53 PATHWAY	1.42E-07	3.24
	APOPTOSIS	1.54E-05	3.09
	CHOLESTEROL HOMEOSTASIS	8.95E-04	3.46
	MTORC1 SIGNALING	3.96E-03	2.05
	UV RESPONSE DN	7.24E-03	2.55
	INFLAMMATORY RESPONSE	1.42E-02	2.25
	UV RESPONSE UP	2.73E-02	2.07
	TGF BETA SIGNALING	3.69E-02	3.27
Down	E2F TARGETS	7.28E-34	9.33
	G2M CHECKPOINT	5.54E-21	6.87
	MTORC1 SIGNALING	1.35E-09	4.54
	MYC TARGETS V1	1.08E-08	4.54
	MITOTIC SPINDLE	3.11E-06	3.83
	CHOLESTEROL HOMEOSTASIS	8.95E-04	4.68
	UNFOLDED PROTEIN RESPONSE	2.77E-03	3.52
	ESTROGEN RESPONSE LATE	4.24E-03	2.84
	GLYCOLYSIS	1.27E-02	2.84
	PEROXISOME	1.28E-02	3.83

d **Frequently perturbed hiPSC NPC gene sets**

Hallmark gene set enrichments

	Gene Set	FDR	Fold Change
Up	TNFA SIGNALING VIA NFKB	1.4E-23	4.86
	HYPOXIA	2.8E-07	2.88
	APOPTOSIS	7.4E-05	2.63
	**UNFOLDED PROTEIN RESPONSE**	1.7E-03	2.63
	MTORC1 SIGNALING	3.2E-03	1.99
	UV RESPONSE UP	5.6E-03	2.28
	P53 PATHWAY	8.8E-03	1.98
	UV RESPONSE DN	5.1E-02	1.94
	**ANDROGEN RESPONSE**	5.3E-02	2.09
	TGF BETA SIGNALING	8.5E-02	2.47
Down	E2F TARGETS	1.6E-49	9.69
	G2M CHECKPOINT	7.6E-34	7.58
	MTORC1 SIGNALING	1.7E-13	4.72
	CHOLESTEROL HOMEOSTASIS	1.9E-11	8.25
	MITOTIC SPINDLE	7.0E-09	3.98
	MYC TARGETS V1	8.6E-08	3.64
	MYC TARGETS V2	3.2E-06	6.05
	ESTROGEN RESPONSE LATE	2.2E-03	2.67
	**FATTY ACID METABOLISM**	4.5E-03	2.97
	**ANDROGEN RESPONSE**	3.3E-02	2.61

Supplementary Figure 4. Drug-induced probes and genes in CCLs and hiPSC NPCs.
(a-b) Table of top 15 most frequently up- and down-regulated genes (absolute difference in robust Z-score ≥ 2) in CCLs (a) and hiPSC NPCs (b) across all drug-treatment conditions. (c-d) Biological pathways associated with gene sets that are frequently perturbed (>= 5 drugs) in CCLs (c) and hiPSC NPCs (d). Highlighted in bold are those pathways frequently perturbed in hiPSC NPCs but not CCLs.

a

Chemogenomic Type	Chemogenomic Feature	SZ-set drugs	FDR	Drugs in overlap	Fold Change
Drug Targets	ADRA2A	SZ DE genes (UP)	7.8E-02	Amoxapine, clozapine, risperidone, trazodone, Ziprasidone	4.44
	HTR2C		7.8E-02		4.44
	ADRA1A		7.8E-02		4.00
	HTR1A		7.8E-02		4.00
	HRH1		8.8E-02		3.64
	HTR2A		8.8E-02		3.64
Drug Class ATC	Other antipsychotics	Red SZ Network	7.2E-02	aripiprazole, iloperidone, risperidone	4.94
Drug Targets	ADRA2A	Targets of miR-137 (UP)	1.9E-02	Amoxapine, clozapine, risperidone, trazodone	7.90
	HTR2C		1.9E-02		7.90
	ADRA1A		1.9E-02		7.11
	HTR1A		1.9E-02		7.11
	HRH1		1.9E-02		6.46
	HTR2A		1.9E-02		6.46
	ADRA1B		6.7E-02	Amoxapine, clozapine, risperidone	6.67
	ADRA2B		6.7E-02		6.67
	ADRA2C		7.4E-02		5.93
	DRD4		7.4E-02		5.93
	DRD1		8.8E-02		5.33
	DRD3		8.8E-02		5.33
	SLC6A4		7.4E-02	Amoxapine, trazodone	11.85
	HTR1D		8.3E-02	Estriol, Naloxone HCl, Nogestrel	8.89

b

Chemogenomic Type	Chemogenomic Feature	SZ-set drugs	FDR	Drugs in overlap	Fold Change
Drug Targets	DRD1	Syn-509 (UP)	1.7E-02	Aripiprazole, haloperidol, iloperidone, risperidone	7.11
	DRD3		1.7E-02		7.11
	DRD2		1.9E-02		6.46
	HTR2A		6.2E-03	Aripiprazole, haloperidol, iloperidone, risperidone, trazodone	8.08
ATC (4)	Other antipsychotics		1.5E-02	Aripiprazole, iloperidone, risperidone	8.09
Drug Targets	ADRA2C		8.0E-02		5.93
	DRD4		8.0E-02		5.93
	ADRA1A		1.7E-02	Aripiprazole, iloperidone, risperidone, trazodone	7.11
	HTR1A		1.7E-02		7.11
	HRH1		1.9E-02		6.46
	ADRA2A		8.0E-02	Aripiprazole, risperidone, trazodone	5.93
	HTR2C		8.0E-02		5.93
	DRD1	Syn-187 (UP)	2.8E-02	Aripiprazole, haloperidol, iloperidone, risperidone	6.40
	DRD3		2.8E-02		6.40
	DRD2		3.0E-02		5.82
	HTR2A		1.2E-02	Aripiprazole, haloperidol, iloperidone, risperidone, trazodone	7.27
ATC (4)	Other antipsychotics		7.4E-03	Aripiprazole, iloperidone, risperidone	9.89
Drug Targets	ADRA1A		2.8E-02	Aripiprazole, iloperidone, risperidone, trazodone	6.40
	HTR1A		2.8E-02		6.40
	HRH1		3.0E-02		5.82
Side-effect	Mental disorder	Syn-39 (UP)	9.0E-02	Aripiprazole, haloperidol, iloperidone, naltrexone HCl, potassium estrone sulfate, risperidone, sorafenib, vigabatrin	2.65
ATC (4)	Other antipsychotics		1.9E-02	Aripiprazole, iloperidone, risperidone	7.42

Supplementary Figure 5. Drug-induced chemogenic analysis in CCLs and hiPSC NPCs.
(a-b) Chemogenic features that differ by cell type (a) or diagnosis (b), expanded from **Figure 4e and 5e**.

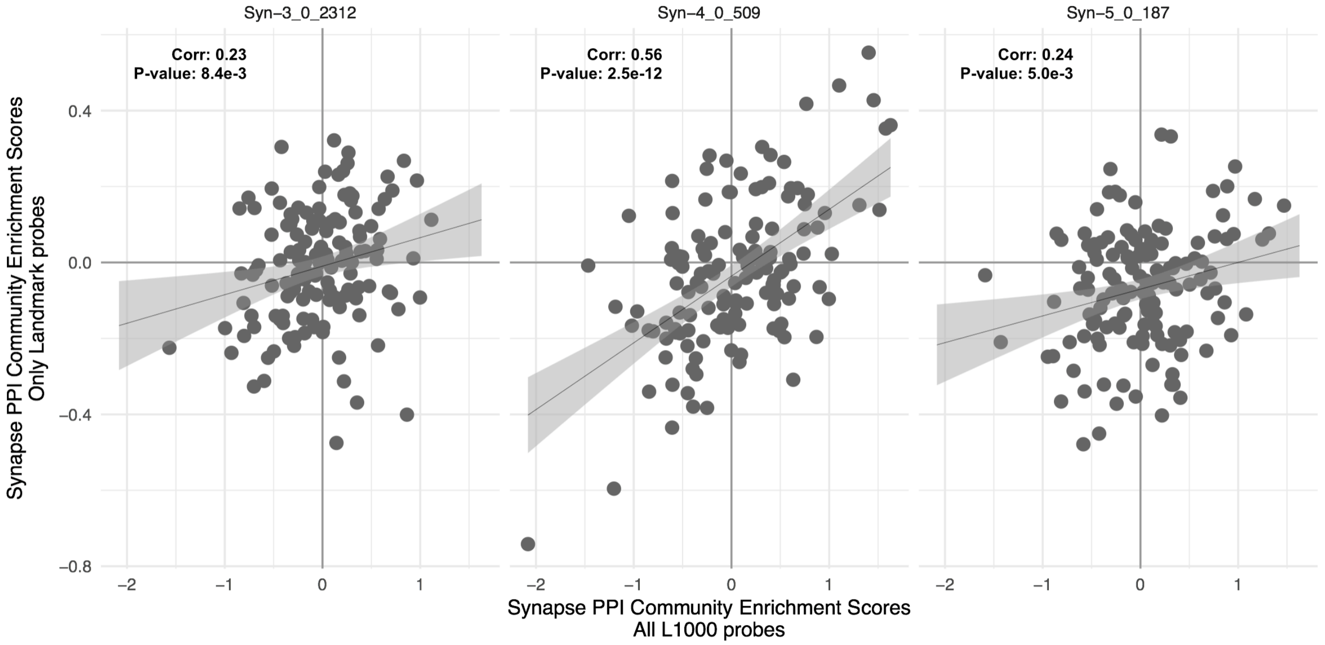

Supplementary Figure 6. Concordance of Synapse PPI Enrichment Scores between all L1000 probes and landmark probes only.

Summary of Synapse PPI Community enrichments for all drugs, calculated using all L1000 probes, compared with enrichment scores generated using only 978 landmark probes. Pearson correlation of enrichment t-statistics for all drugs assayed within the study, are shown.

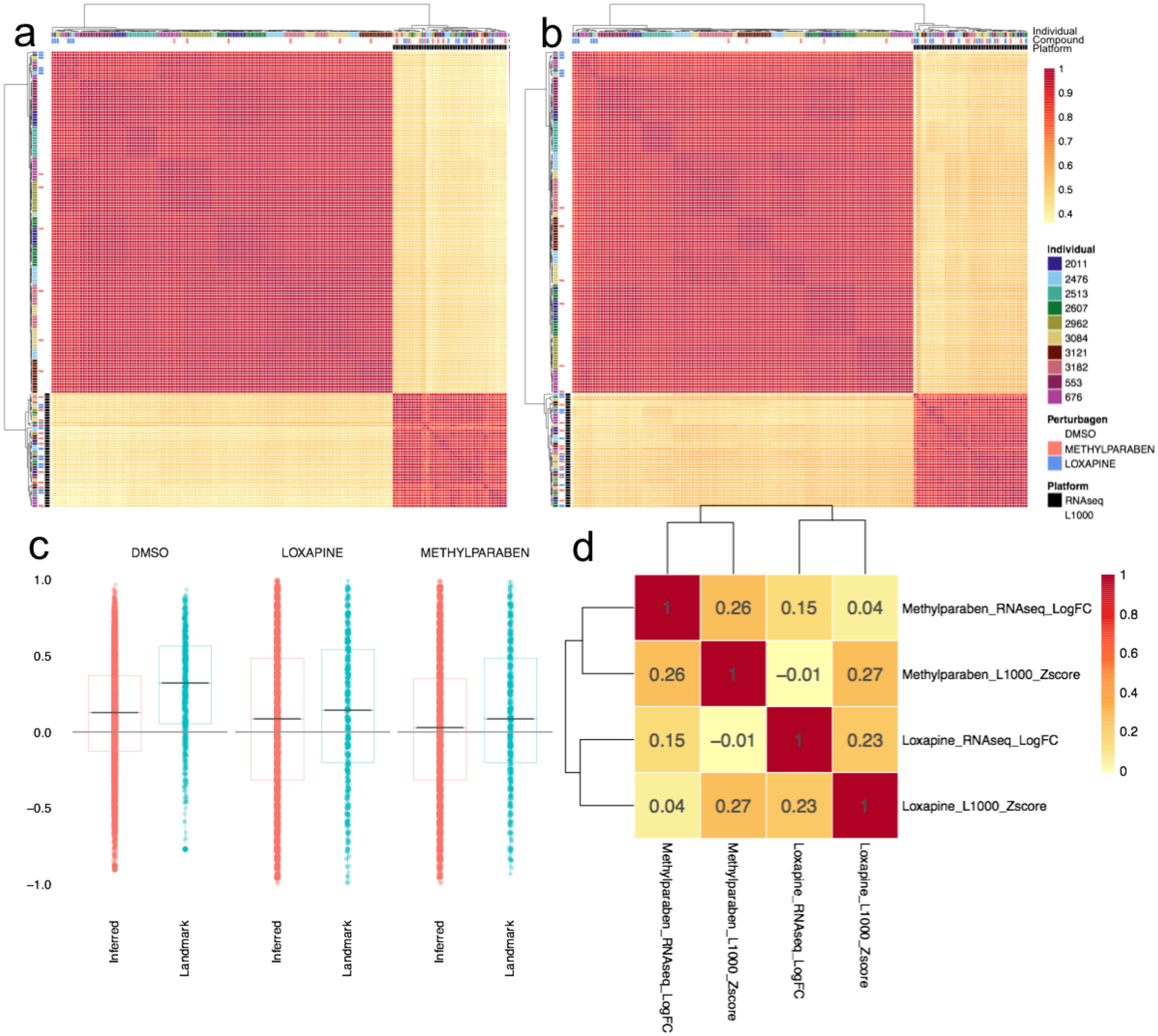

Supplementary Figure 7. Mean pairwise Spearman's correlation across L1000 and RNA-seq platforms from hiPSC NPCs treated with loxapine or methylparaben.

(a) Pearson correlation of normalized expression data from L1000 and RNA-seq for loxapine, methylparaben and DMSO (all probes). (b) Pearson correlation of normalized expression data from L1000 and RNA-seq for loxapine, methylparaben and DMSO (landmark probes only). (c) Gene-level Spearman correlation of normalized expression data from L1000 and RNA-seq for DMSO, loxapine, methylparaben and DMSO, stratified by L1000 probe-type. (d) Pairwise comparison (correlation) of loxapine and methylparaben signatures from L1000 and RNA-sequencing experiments.

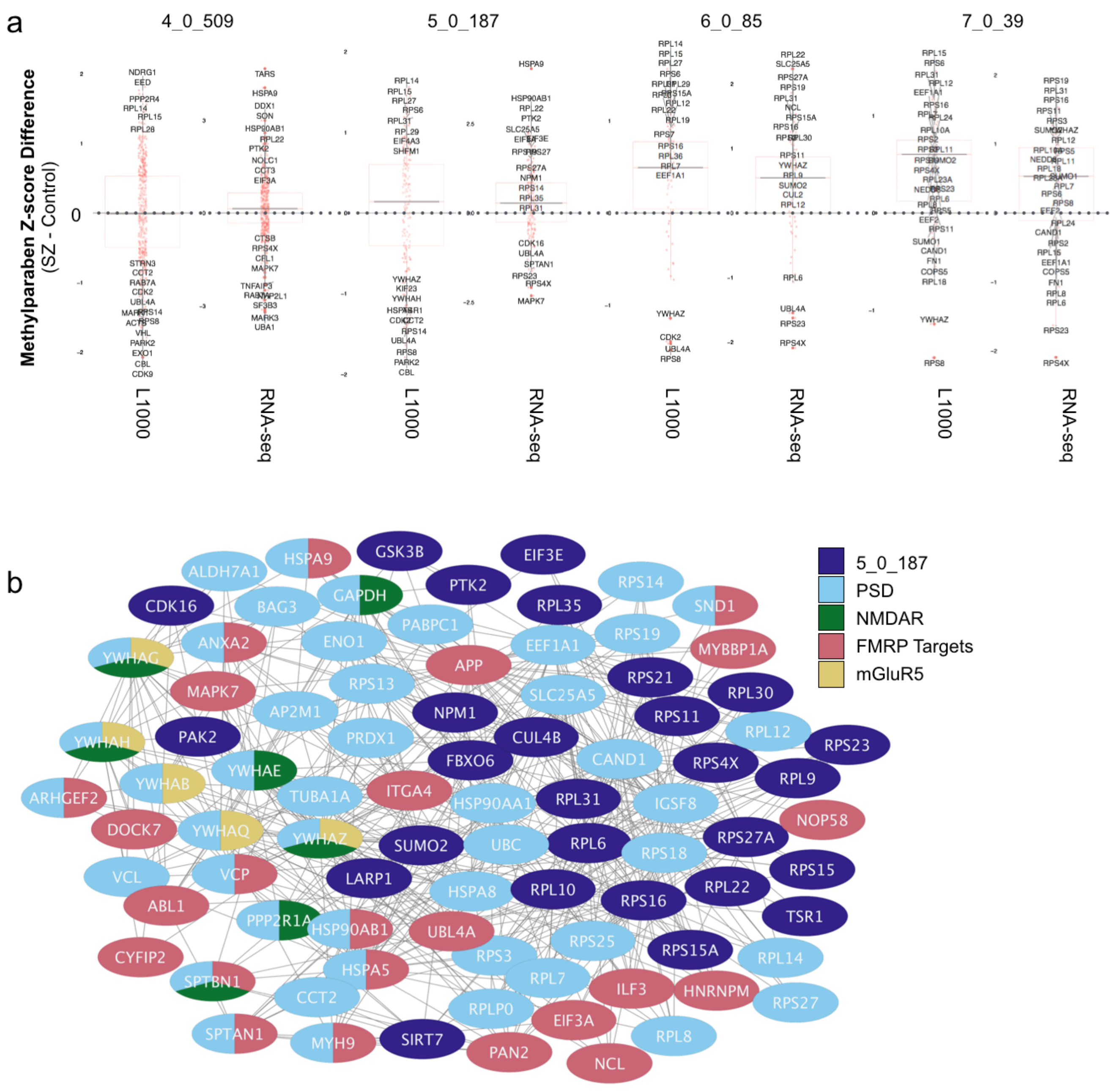

Supplementary Figure 8. Methylparaben-induced regulation of SZ-sets, particularly synapse PPI communities.

(a) Example synapse PPI communities (4_0_509, 5_0_187, 6_0_85, 7_0_39) that are most differentially regulated by methylparaben that are conserved between L1000 and RNA-seq. (Top 20 most differentially expressed genes are labelled) (b) Protein interaction network of synaptic genes perturbed by methylparaben treatment.

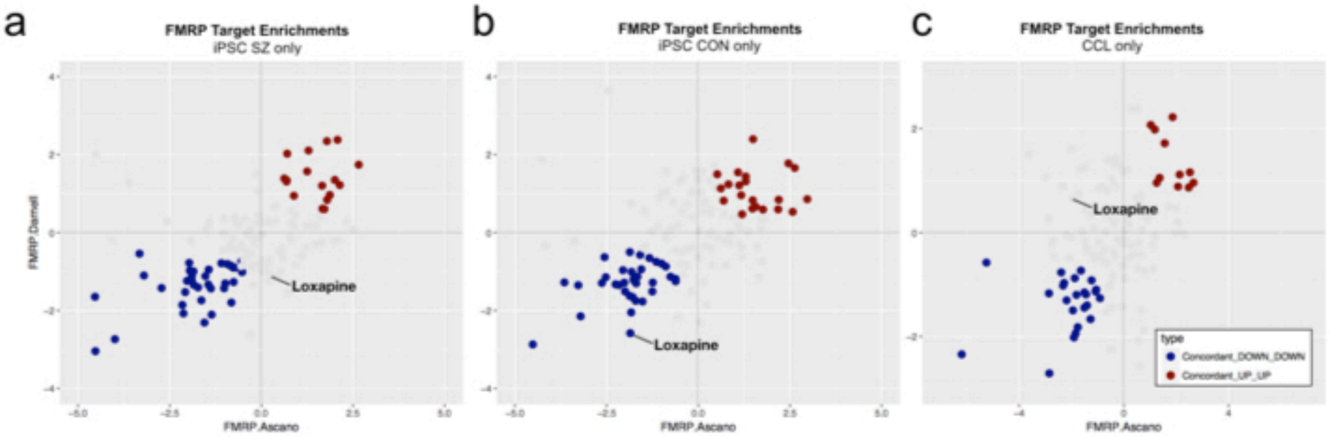

d

ACUK motifs in *de novo* NPD variants

Dx	Study	Locus	Annotation	Ref	Alt	Symbol	Strand	Forward DNA sequence
SZ	Fromer	chr11:83194295	esplice	C	T	DLG2	-	ACTTACTCAGA
ID	Rauch	chr9:130422307-130422308	esplice	AG	N	STXBP1	+	TCCACAGTCCGT
SZ	Xu	chr12:120306864	missense	A	G	CIT	-	CTTACACTTCC
ASD	Iossifov	chr12:120172026	missense	T	C	CIT	-	CAAACTGGGAT
ASD	ORoak	chr2:149225965-149225967	frameshift	GTC	N	MBD5	+	AATAAGTCTCATG
ID	Rauch	chr22:42273402-42273403	missense	GC	TT	SREBF2	+	TGGCCGCAGTGT
SZ	Xu	chr15:54586124	missense	G	T	UNC13C	+	TCAAAGTCAGA
SZ	Fromer	chr19:2117265	missense	G	C	AP3D1	-	ACTGGGTTCAG
ASD	Iossifov	chr19:2121195	missense	T	C	AP3D1	-	TGGACTGGCTG
ASD	ORoak	chr20:49510027-49510029	frameshift	CTT	N	ADNP	-	GGAGACTTTAACT

Supplementary Figure 9. Drug-induced regulation of FMRP targets.

(a-c) FMRP target enrichments in SZ hiPSC NPCs (a), control hiPSC NPCs (b) and CCLs (c). (d) ACUK FMRP binding motif variants identified in neuropsychiatric disease (NPD) mutations.

Supplementary Table 1. Final prioritization of candidate drugs. **README**

Column	Explanation
**DRUG**	Drug name
**LSN**	Drug identifier
**nPhase**	Number of phases in which drug was profiled (max 2)
**Phases**	Which phases a drug was profiled in
**Method_of_selection**	Broad approach by which drug was prioritized for profiling
**Target_of_interest_profile**	If drug was selected due to target profile, whether drug was associated with selective, or promiscuous target profile
**SZ_Sets**	If drug was selected due to association with SZ-sets, which SZ-set was implicated
**Method_of_Connections_with_SZ_Sets**	If drug was selected due to association with SZ-sets, the sort of connection upon which association was based
**ATC_level_1**	Drug class according to Anatomical and Therapeutic Chemical Classification System, Level 1
**ATC_level_2**	Drug class according to Anatomical and Therapeutic Chemical Classification System, Level 2
**ATC_level_3**	Drug class according to Anatomical and Therapeutic Chemical Classification System, Level 3
**ATC_level_4**	Drug class according to Anatomical and Therapeutic Chemical Classification System, Level 4
**ATC_level_5**	Drug class according to Anatomical and Therapeutic Chemical Classification System, Level 5
**selective**	
**promiscuous**	
**1P1**	
**LSN**	
**ATC**	
**SZ-set**	
**SZ-set libraries**	
**SCZ-denovo**	
**PGC2**	

Supplementary Table 1. Final prioritization of candidate drugs.

<table>
<tr><th>DRUG</th><th>LSN</th><th>nPhase</th><th>Phases</th><th>Method_of_selection</th><th>Target_of_interest_profile</th><th>SZ_Sets</th><th>Method_of_Connections_with_SZ_Sets</th><th>ATC_level_1</th><th>ATC_level_2</th><th>ATC_level_3</th><th>ATC_level_4</th><th>ATC_level_5</th></tr>
<tr><td>DIPHEMANIL.METILSULFATE</td><td>26209</td><td>1</td><td>P1</td><td>Connectivity_with_SZ_sets</td><td></td><td>Coexp_Module_black</td><td>topConnectivityScores</td><td>A_ALIMENTARY_TRAC</td><td>A03_DRUGS_FOR_FUN</td><td>A03A_DRUGS_FOR_FU</td><td>A03AB_Synthetic_anti</td><td>A03AB15_</td></tr>
<tr><td>LANSOPRAZOLE</td><td>520544</td><td>1</td><td>P1</td><td>Connectivity_with_SZ_sets</td><td></td><td>Coexp_Module_lightcyan</td><td>topConnectivityScores</td><td>A_ALIMENTARY_TRAC</td><td>A02_DRUGS_FOR_ACI</td><td>A02B_DRUGS_FOR_PE</td><td>A02BC_Proton_pump_</td><td>A02BC03_</td></tr>
<tr><td>GLIPIZIDE</td><td>351297</td><td>1</td><td>P2</td><td>Targets_of_interest</td><td>Selective</td><td></td><td></td><td>A_ALIMENTARY_TRAC</td><td>A10_DRUGS_USED_IN_</td><td>A10B_BLOOD_GLUCOS</td><td>A10BB_Sulfonamides,_</td><td>A10BB07_</td></tr>
<tr><td>REPAGLINIDE</td><td>2115645</td><td>1</td><td>P1</td><td>Connectivity_with_SZ_sets</td><td></td><td>Syn-3_0_2312</td><td>TopConnectivityScores</td><td>A_ALIMENTARY_TRAC</td><td>A10_DRUGS_USED_IN_</td><td>A10B_BLOOD_GLUCOS</td><td>A10BX_Other_blood_g</td><td>A10BX02_</td></tr>
<tr><td>CHENODEOXYCHOLIC.ACID</td><td>28374</td><td>2</td><td>P1|P2</td><td>Connectivity_with_SZ_sets</td><td></td><td>Coexp_Module_black</td><td>topConnectivityScores</td><td>A_ALIMENTARY_TRAC</td><td>A05_BILE_AND_LIVER_</td><td>A05A_BILE_THERAPY|</td><td>A05AA_Bile_acid_prep</td><td>A05AA01_|A05AA02_</td></tr>
<tr><td>BUDESONIDE</td><td>686163</td><td>1</td><td>P1</td><td>Connectivity_with_SZ_sets</td><td></td><td>Syn-3_0_2312</td><td>topKnown+PredictedTargetEnrichments</td><td>A_ALIMENTARY_TRAC</td><td>A07_ANTIDIARRHEALS</td><td>A07E_INTESTINAL_AN</td><td>A07EA_Corticosteroids</td><td>A07EA06_|D07AC09_|R01AD05_|R03BA02_</td></tr>
<tr><td>BAY.K8644</td><td>Pilot1.5|BAY K8644</td><td>2</td><td>P1|P2</td><td>Tool_compound</td><td></td><td></td><td></td><td>C_CARDIOVASCULAR_</td><td>C08_CALCIUM_CHANN</td><td>C08C_SELECTIVE_CALC</td><td>C08CA_Dihydropyridin</td><td>C08CA06_</td></tr>
<tr><td>BUMETANIDE</td><td>55431</td><td>1</td><td>P1</td><td>Connectivity_with_SZ_sets</td><td></td><td>Coexp_Module_black</td><td>topConnectivityScores</td><td>C_CARDIOVASCULAR_</td><td>C03_DIURETICS</td><td>C03C_HIGH-CEILING_D</td><td>C03CA_Sulfonamides,_</td><td>C03CA02_</td></tr>
<tr><td>DIHYDROERGOCRISTINE</td><td>486439</td><td>1</td><td>P1</td><td>Connectivity_with_SZ_sets</td><td></td><td>Coexp_Module_brown</td><td>topConnectivityScores</td><td>C_CARDIOVASCULAR_</td><td>C04_PERIPHERAL_VAS</td><td>C04A_PERIPHERAL_VA</td><td>C04AE_Ergot_alkaloids</td><td>C04AE04_</td></tr>
<tr><td>DILTIAZEM</td><td>486402</td><td>1</td><td>P1</td><td>Connectivity_with_SZ_sets</td><td></td><td>Syn-3_0_2312</td><td>TopConnectivityScores</td><td>C_CARDIOVASCULAR_</td><td>C08_CALCIUM_CHANN</td><td>C08D_SELECTIVE_CALC</td><td>C08DB_Benzothiazepir</td><td>C08DB01_</td></tr>
<tr><td>FENDILINE</td><td>686097</td><td>1</td><td>P1</td><td>Connectivity_with_SZ_sets</td><td></td><td>Coexp_Module_brown</td><td>topConnectivityScores</td><td>C_CARDIOVASCULAR_</td><td>C08_CALCIUM_CHANN</td><td>C08E_NON-SELECTIVE_</td><td>C08EA_Phenylalkylami</td><td>C08EA01_</td></tr>
<tr><td>FLECAINIDE</td><td>2351817</td><td>1</td><td>P1</td><td>Connectivity_with_SZ_sets</td><td></td><td>Coexp_Module_lightcyan</td><td>topConnectivityScores</td><td>C_CARDIOVASCULAR_</td><td>C01_CARDIAC_THERAP</td><td>C01B_ANTIARRHYTHM</td><td>C01BC_Antiarrhythmic</td><td>C01BC04_</td></tr>
<tr><td>FUROSEMIDE</td><td>58858</td><td>1</td><td>P1</td><td>Connectivity_with_SZ_sets</td><td></td><td>Coexp_Module_black</td><td>topConnectivityScores</td><td>C_CARDIOVASCULAR_</td><td>C03_DIURETICS</td><td>C03C_HIGH-CEILING_D</td><td>C03CA_Sulfonamides,_</td><td>C03CA01_</td></tr>
<tr><td>BUFLOMEDIL.HYDROCHLORIDE</td><td>686142</td><td>1</td><td>P2</td><td>Targets_of_interest</td><td>Selective</td><td></td><td></td><td>C_CARDIOVASCULAR_</td><td>C04_PERIPHERAL_VAS</td><td>C04A_PERIPHERAL_VA</td><td>C04AX_Other_periphe</td><td>C04AX20_</td></tr>
<tr><td>EPLERENONE</td><td>508928</td><td>1</td><td>P2</td><td>Targets_of_interest</td><td>Selective</td><td></td><td></td><td>C_CARDIOVASCULAR_</td><td>C03_DIURETICS</td><td>C03D_POTASSIUM-SPA</td><td>C03DA_Aldosterone_a</td><td>C03DA04_</td></tr>
<tr><td>MOXONIDINE</td><td>433923</td><td>1</td><td>P1</td><td>Connectivity_with_SZ_sets</td><td></td><td>Coexp_Module_lightcyan</td><td>topKnown+PredictedTargetEnrichments (NISCH)</td><td>C_CARDIOVASCULAR_</td><td>C02_ANTIHYPERTENSI</td><td>C02A_ANTIADRENERG</td><td>C02AC_Imidazoline_re</td><td>C02AC05_</td></tr>
<tr><td>NADOLOL</td><td>467746</td><td>1</td><td>P1</td><td>Connectivity_with_SZ_sets</td><td></td><td>Coexp_Module_black|Coexp_Module_brown</td><td>topConnectivityScores</td><td>C_CARDIOVASCULAR_</td><td>C07_BETA_BLOCKING_</td><td>C07A_BETA_BLOCKING</td><td>C07AA_Beta_blocking_</td><td>C07AA12_</td></tr>
<tr><td>NIMODIPINE</td><td>Pilot1.6|Nimodipine</td><td>2</td><td>P1|P2</td><td>Tool_compound</td><td></td><td></td><td></td><td>C_CARDIOVASCULAR_</td><td>C08_CALCIUM_CHANN</td><td>C08C_SELECTIVE_CALC</td><td>C08CA_Dihydropyridin</td><td>C08CA06_</td></tr>
<tr><td>PRENYLAMINE</td><td>686173</td><td>1</td><td>P1</td><td>Connectivity_with_SZ_sets</td><td></td><td>Coexp_Module_brown</td><td>topConnectivityScores</td><td>C_CARDIOVASCULAR_</td><td>C01_CARDIAC_THERAP</td><td>C01D_VASODILATORS_</td><td>C01DX_Other_vasodila</td><td>C01DX02_</td></tr>
<tr><td>SOTALOL</td><td>2330927</td><td>1</td><td>P1</td><td>Connectivity_with_SZ_sets</td><td></td><td>Coexp_Module_lightcyan</td><td>topConnectivityScores</td><td>C_CARDIOVASCULAR_</td><td>C07_BETA_BLOCKING_</td><td>C07A_BETA_BLOCKING</td><td>C07AA_Beta_blocking_</td><td>C07AA07_</td></tr>
<tr><td>SULOCTIDIL</td><td>255305</td><td>2</td><td>P1|P2</td><td>Connectivity_with_SZ_sets</td><td></td><td>Coexp_Module_brown</td><td>topConnectivityScores</td><td>C_CARDIOVASCULAR_</td><td>C04_PERIPHERAL_VAS</td><td>C04A_PERIPHERAL_VA</td><td>C04AX_Other_periphe</td><td>C04AX19_</td></tr>
<tr><td>SPIRONOLACTONE</td><td>604169</td><td>1</td><td>P2</td><td>Targets_of_interest</td><td>Selective</td><td></td><td></td><td>C_CARDIOVASCULAR_</td><td>C03_DIURETICS</td><td>C03D_POTASSIUM-SPA</td><td>C03DA_Aldosterone_a</td><td>C03DA01_</td></tr>
<tr><td>DIFLORASONE</td><td>57136</td><td>1</td><td>P1</td><td>Connectivity_with_SZ_sets</td><td></td><td>Coexp_Module_lightcyan|Coexp_Module_black</td><td>topConnectivityScores</td><td>D_DERMATOLOGICALS</td><td>D07_CORTICOSTEROID</td><td>D07A_CORTICOSTEROI</td><td>D07AC_Corticosteroids</td><td>D07AC10_</td></tr>
<tr><td>MAFENIDE.HYDROCHLORIDE</td><td>81700</td><td>1</td><td>P2</td><td>Targets_of_interest</td><td>Promiscuous</td><td></td><td></td><td>D_DERMATOLOGICALS</td><td>D06_ANTIBIOTICS_AN</td><td>D06B_CHEMOTHERAP</td><td>D06BA_Sulfonamides</td><td>D06BA03_</td></tr>
<tr><td>PODOPHYLLOTOXIN</td><td>4038</td><td>1</td><td>P2</td><td>Connectivity_with_SZ_sets|Targets_of_interest</td><td>Promiscuous</td><td>Coexp_Module_brown</td><td>topConnectivityScores</td><td>D_DERMATOLOGICALS</td><td>D06_ANTIBIOTICS_AN</td><td>D06B_CHEMOTHERAP</td><td>D06BB_Antivirals</td><td>D06BB04_</td></tr>
<tr><td>SULCONAZOLE</td><td>2402722</td><td>1</td><td>P1</td><td>Connectivity_with_SZ_sets</td><td></td><td>Syn-3_0_2312</td><td>TopConnectivityScores</td><td>D_DERMATOLOGICALS</td><td>D01_ANTIFUNGALS_F</td><td>D01A_ANTIFUNGALS_F</td><td>D01AC_Imidazole_and</td><td>D01AC09_</td></tr>
<tr><td>NOGESTREL</td><td>526576</td><td>1</td><td>P2</td><td>Targets_of_interest</td><td>Selective</td><td></td><td></td><td>G_GENITO_URINARY_</td><td>G03_SEX_HORMONES_</td><td>G03A_HORMONAL_CC</td><td>G03AC_Progestogens</td><td>G03AC08_</td></tr>
<tr><td>ESTRONE</td><td>13550</td><td>1</td><td>P2</td><td>Targets_of_interest</td><td>Promiscuous</td><td></td><td></td><td>G_GENITO_URINARY_</td><td>G03_SEX_HORMONES_</td><td>G03C_ESTROGENS|G0</td><td>G03CA_Natural_and_s</td><td>G03CA07_|G03CC04_</td></tr>
<tr><td>ESTRIOL</td><td>71211</td><td>1</td><td>P2</td><td>Targets_of_interest</td><td>Selective</td><td></td><td></td><td>G_GENITO_URINARY_</td><td>G03_SEX_HORMONES_</td><td>G03C_ESTROGENS|G0</td><td>G03CA_Natural_and_s</td><td>G03CA04_|G03CC06_</td></tr>
<tr><td>CARBIMAZOLE</td><td>61961</td><td>1</td><td>P1</td><td>Connectivity_with_SZ_sets</td><td></td><td>Coexp_Module_lightcyan</td><td>topConnectivityScores</td><td>H_SYSTEMIC_HORMO</td><td>H03_THYROID_THERA</td><td>H03B_ANTITHYROID_P</td><td>H03BB_Sulfur-containi</td><td>H03BB01_</td></tr>
<tr><td>LEVOTHYROXINE.SODIUM</td><td>2937597</td><td>1</td><td>P1</td><td>Connectivity_with_SZ_sets</td><td></td><td>Coexp_Module_lightcyan</td><td>topConnectivityScores</td><td>H_SYSTEMIC_HORMO</td><td>H03_THYROID_THERA</td><td>H03A_THYROID_PREP</td><td>H03AA_Thyroid_horm</td><td>H03AA01_</td></tr>
<tr><td>ETHIONAMIDE</td><td>438600</td><td>1</td><td>P1</td><td>Connectivity_with_SZ_sets</td><td></td><td>Coexp_Module_black</td><td>topConnectivityScores</td><td>J_ANTIINFECTIVES_FO</td><td>J04_ANTIMYCOBACTE</td><td>J04A_DRUGS_FOR_TR</td><td>J04AD_Thiocarbamide_</td><td>J04AD03_</td></tr>
<tr><td>ISONIAZID</td><td>16660</td><td>1</td><td>P1</td><td>Connectivity_with_SZ_sets</td><td></td><td>Coexp_Module_black</td><td>topConnectivityScores</td><td>J_ANTIINFECTIVES_FO</td><td>J04_ANTIMYCOBACTE</td><td>J04A_DRUGS_FOR_TR</td><td>J04AC_Hydrazides</td><td>J04AC01_</td></tr>
<tr><td>LAMIVUDINE</td><td>543742</td><td>1</td><td>P2</td><td>Targets_of_interest</td><td>Selective</td><td></td><td></td><td>J_ANTIINFECTIVES_FO</td><td>J05_ANTIVIRALS_FOR_</td><td>J05A_DIRECT_ACTING_</td><td>J05AF_Nucleoside_and</td><td>J05AF05_</td></tr>
<tr><td>NORFLOXACIN</td><td>177733</td><td>1</td><td>P2</td><td>Targets_of_interest</td><td>Promiscuous</td><td></td><td></td><td>J_ANTIINFECTIVES_FO</td><td>J01_ANTIBACTERIALS_</td><td>J01M_QUINOLONE_AN</td><td>J01MA_Fluoroquinolor</td><td>J01MA06_|S01AX12_</td></tr>
<tr><td>BOSUTINIB</td><td>3149766</td><td>1</td><td>P2</td><td>Targets_of_interest</td><td>Promiscuous</td><td></td><td></td><td>L_ANTINEOPLASTIC_A</td><td>L01_ANTINEOPLASTIC_</td><td>L01X_OTHER_ANTINE</td><td>L01XE_Protein_kinase_</td><td>L01XE14_</td></tr>
<tr><td>SORAFENIB</td><td>2133026</td><td>1</td><td>P2</td><td>Targets_of_interest</td><td>Promiscuous</td><td></td><td></td><td>L_ANTINEOPLASTIC_A</td><td>L01_ANTINEOPLASTIC_</td><td>L01X_OTHER_ANTINE</td><td>L01XE_Protein_kinase_</td><td>L01XE05_</td></tr>
<tr><td>VANDETANIB</td><td>2442691</td><td>1</td><td>P2</td><td>Targets_of_interest</td><td>Promiscuous</td><td></td><td></td><td>L_ANTINEOPLASTIC_A</td><td>L01_ANTINEOPLASTIC_</td><td>L01X_OTHER_ANTINE</td><td>L01XE_Protein_kinase_</td><td>L01XE12_</td></tr>
<tr><td>NORDIHYDROGUAIARETIC.ACID</td><td>2409</td><td>1</td><td>P1</td><td>Connectivity_with_SZ_sets</td><td></td><td>Coexp_Module_lightcyan</td><td>topConnectivityScores</td><td>L_ANTINEOPLASTIC_A</td><td>L01_ANTINEOPLASTIC_</td><td>L01X_OTHER_ANTINE</td><td>L01XX_Other_antineo</td><td>L01XX10_</td></tr>
<tr><td>VORINOSTAT</td><td>2325578</td><td>1</td><td>P1</td><td>Connectivity_with_SZ_sets</td><td></td><td>Syn-3_0_2312</td><td>topKnownTargetEnrichments</td><td>L_ANTINEOPLASTIC_A</td><td>L01_ANTINEOPLASTIC_</td><td>L01X_OTHER_ANTINE</td><td>L01XX_Other_antineo</td><td>L01XX38_</td></tr>
<tr><td>HYDROQUININE</td><td>686160</td><td>1</td><td>P1</td><td>Connectivity_with_SZ_sets</td><td></td><td>Coexp_Module_brown</td><td>topConnectivityScores</td><td>M_MUSCULO-SKELETA</td><td>M09_OTHER_DRUGS_</td><td>M09A_OTHER_DRUGS_</td><td>M09AA_Quinine_and_</td><td>M09AA01_</td></tr>
<tr><td>NIFLUMIC.ACID</td><td>62444</td><td>1</td><td>P1</td><td>Connectivity_with_SZ_sets</td><td></td><td>Syn-3_0_2312</td><td>topKnown+PredictedTargetEnrichments</td><td>M_MUSCULO-SKELETA</td><td>M01_ANTIINFLAMMAT</td><td>M01A_ANTIINFLAMM</td><td>M01AX_Other_antiinfl</td><td>M01AX02_|M02AA17_</td></tr>
<tr><td>AMOXAPINE</td><td>409924</td><td>1</td><td>P1</td><td>Connectivity_with_SZ_sets</td><td></td><td>Coexp_Module_brown</td><td>topKnownTargetEnrichments</td><td>N_NERVOUS_SYSTEM</td><td>N06_PSYCHOANALEPT</td><td>N06A_ANTIDEPRESSAN</td><td>N06AA_Non-selective_</td><td>N06AA17_</td></tr>
<tr><td>ARIPIPRAZOLE</td><td>2073076</td><td>2</td><td>P1|P2</td><td>Connectivity_with_SZ_sets</td><td></td><td>Coexp_Module_brown</td><td>topKnownTargetEnrichments</td><td>N_NERVOUS_SYSTEM</td><td>N05_PSYCHOLEPTICS</td><td>N05A_ANTIPSYCHOTIC</td><td>N05AX_Other_antipsy</td><td>N05AX12_</td></tr>
<tr><td>CHLORPROMAZINE</td><td>431837</td><td>1</td><td>P1</td><td>Connectivity_with_SZ_sets</td><td></td><td>Coexp_Module_brown</td><td>topKnownTargetEnrichments</td><td>N_NERVOUS_SYSTEM</td><td>N05_PSYCHOLEPTICS</td><td>N05A_ANTIPSYCHOTIC</td><td>N05AA_Phenothiazine</td><td>N05AA01_</td></tr>
<tr><td>CLOZAPINE</td><td>100326</td><td>1</td><td>P2</td><td>Connectivity_with_SZ_sets|Targets_of_interest</td><td>Promiscuous</td><td>Coexp_Module_lightcyan|Coexp_Module_brown</td><td>topKnownTargetEnrichments (HRH4)|topKnownTargetEnrichments</td><td>N_NERVOUS_SYSTEM</td><td>N05_PSYCHOLEPTICS</td><td>N05A_ANTIPSYCHOTIC</td><td>N05AH_Diazepines,_o</td><td>N05AH02_</td></tr>
<tr><td>MEMANTINE.HYDROCHLORIDE</td><td>50438</td><td>1</td><td>P2</td><td>Targets_of_interest</td><td>Promiscuous</td><td></td><td></td><td>N_NERVOUS_SYSTEM</td><td>N06_PSYCHOANALEPT</td><td>N06D_ANTI-DEMENTIA</td><td>N06DX_Other_anti-de</td><td>N06DX01_</td></tr>
<tr><td>RISPERIDONE</td><td>202549</td><td>1</td><td>P2</td><td>Targets_of_interest</td><td>Promiscuous</td><td></td><td></td><td>N_NERVOUS_SYSTEM</td><td>N05_PSYCHOLEPTICS</td><td>N05A_ANTIPSYCHOTIC</td><td>N05AX_Other_antipsy</td><td>N05AX08_</td></tr>
<tr><td>HALOPERIDOL</td><td>56047</td><td>1</td><td>P1</td><td>Connectivity_with_SZ_sets</td><td></td><td>Coexp_Module_black</td><td>topConnectivityScores</td><td>N_NERVOUS_SYSTEM</td><td>N05_PSYCHOLEPTICS</td><td>N05A_ANTIPSYCHOTIC</td><td>N05AD_Butyrophenon</td><td>N05AD01_</td></tr>
<tr><td>BUPROPION.HYDROCHLORIDE</td><td>486429</td><td>1</td><td>P2</td><td>Targets_of_interest</td><td>Selective</td><td></td><td></td><td>N_NERVOUS_SYSTEM</td><td>N06_PSYCHOANALEPT</td><td>N06A_ANTIDEPRESSAN</td><td>N06AX_Other_antidep</td><td>N06AX12_</td></tr>
<tr><td>LOXAPINE</td><td>2797238</td><td>1</td><td>P1</td><td>Connectivity_with_SZ_sets</td><td></td><td>Coexp_Module_brown</td><td>topKnownTargetEnrichments</td><td>N_NERVOUS_SYSTEM</td><td>N05_PSYCHOLEPTICS</td><td>N05A_ANTIPSYCHOTIC</td><td>N05AH_Diazepines,_o</td><td>N05AH01_</td></tr>
<tr><td>MELATONIN</td><td>45235</td><td>1</td><td>P1</td><td>Connectivity_with_SZ_sets</td><td></td><td>Coexp_Module_lightcyan|Coexp_Module_black</td><td>topConnectivityScores|topKnown+PredictedTargetEnrichments (MPO)</td><td>N_NERVOUS_SYSTEM</td><td>N05_PSYCHOLEPTICS</td><td>N05C_HYPNOTICS_AN</td><td>N05CH_Melatonin_rec</td><td>N05CH01_</td></tr>
<tr><td>NEFOPAM</td><td>81742</td><td>2</td><td>P1|P2</td><td>Connectivity_with_SZ_sets</td><td></td><td>Coexp_Module_lightcyan</td><td>topConnectivityScores</td><td>N_NERVOUS_SYSTEM</td><td>N02_ANALGESICS</td><td>N02B_OTHER_ANALGE</td><td>N02BG_Other_analges</td><td>N02BG06_</td></tr>
<tr><td>ILOPERIDONE</td><td>629423</td><td>1</td><td>P2</td><td>Targets_of_interest</td><td>Selective</td><td></td><td></td><td>N_NERVOUS_SYSTEM</td><td>N05_PSYCHOLEPTICS</td><td>N05A_ANTIPSYCHOTIC</td><td>N05AX_Other_antipsy</td><td>N05AX14_</td></tr>
<tr><td>PHENELZINE</td><td>113719</td><td>1</td><td>P1</td><td>Connectivity_with_SZ_sets</td><td></td><td>Coexp_Module_lightcyan|Coexp_Module_black</td><td>topConnectivityScores</td><td>N_NERVOUS_SYSTEM</td><td>N06_PSYCHOANALEPT</td><td>N06A_ANTIDEPRESSAN</td><td>N06AF_Monoamine_o</td><td>N06AF03_</td></tr>
<tr><td>PIZOTIFEN</td><td>2136329</td><td>1</td><td>P1</td><td>Connectivity_with_SZ_sets</td><td></td><td>Coexp_Module_black</td><td>topConnectivityScores</td><td>N_NERVOUS_SYSTEM</td><td>N02_ANALGESICS</td><td>N02C_ANTIMIGRAINE_</td><td>N02CX_Other_antimig</td><td>N02CX01_</td></tr>
<tr><td>PRIMIDONE</td><td>18046</td><td>1</td><td>P1</td><td>Connectivity_with_SZ_sets</td><td></td><td>Syn-3_5_12</td><td>topKnownTargetEnrichments</td><td>N_NERVOUS_SYSTEM</td><td>N03_ANTIEPILEPTICS</td><td>N03A_ANTIEPILEPTICS</td><td>N03AA_Barbiturates_a</td><td>N03AA03_</td></tr>
<tr><td>QUETIAPINE</td><td>2397678</td><td>2</td><td>P1|P2</td><td>Connectivity_with_SZ_sets</td><td></td><td>Coexp_Module_brown</td><td>topKnownTargetEnrichments</td><td>N_NERVOUS_SYSTEM</td><td>N05_PSYCHOLEPTICS</td><td>N05A_ANTIPSYCHOTIC</td><td>N05AH_Diazepines,_o</td><td>N05AH04_</td></tr>
<tr><td>MOCLOBEMIDE</td><td>603362</td><td>1</td><td>P2</td><td>Targets_of_interest</td><td>Selective</td><td></td><td></td><td>N_NERVOUS_SYSTEM</td><td>N06_PSYCHOANALEPT</td><td>N06A_ANTIDEPRESSAN</td><td>N06AG_Monoamine_o</td><td>N06AG02_</td></tr>
<tr><td>NALTREXONE.HYDROCHLORIDE</td><td>486497</td><td>1</td><td>P2</td><td>Targets_of_interest</td><td>Selective</td><td></td><td></td><td>N_NERVOUS_SYSTEM</td><td>N07_OTHER_NERVOUS</td><td>N07B_DRUGS_USED_I</td><td>N07BB_Drugs_used_in</td><td>N07BB04_</td></tr>
<tr><td>TRAZODONE</td><td>81737</td><td>1</td><td>P1</td><td>Connectivity_with_SZ_sets</td><td></td><td>Syn-3_0_2312</td><td>TopConnectivityScores</td><td>N_NERVOUS_SYSTEM</td><td>N06_PSYCHOANALEPT</td><td>N06A_ANTIDEPRESSAN</td><td>N06AX_Other_antidep</td><td>N06AX05_</td></tr>
<tr><td>TRIMETHADIONE</td><td>92223</td><td>1</td><td>P1</td><td>Connectivity_with_SZ_sets</td><td></td><td>Coexp_Module_lightcyan</td><td>topConnectivityScores</td><td>N_NERVOUS_SYSTEM</td><td>N03_ANTIEPILEPTICS</td><td>N03A_ANTIEPILEPTICS</td><td>N03AC_Oxazolidine_de</td><td>N03AC02_</td></tr>
<tr><td>TIAGABINE</td><td>279847</td><td>1</td><td>P2</td><td>Targets_of_interest</td><td>Selective</td><td></td><td></td><td>N_NERVOUS_SYSTEM</td><td>N03_ANTIEPILEPTICS</td><td>N03A_ANTIEPILEPTICS</td><td>N03AG_Fatty_acid_de</td><td>N03AG06_</td></tr>
<tr><td>TRANYLCYPROMINE.HYDROCHLORIDE</td><td>486541</td><td>1</td><td>P2</td><td>Targets_of_interest</td><td>Selective</td><td></td><td></td><td>N_NERVOUS_SYSTEM</td><td>N06_PSYCHOANALEPT</td><td>N06A_ANTIDEPRESSAN</td><td>N06AF_Monoamine_o</td><td>N06AF04_</td></tr>
<tr><td>VIGABATRIN</td><td>486547</td><td>1</td><td>P1</td><td>Connectivity_with_SZ_sets</td><td></td><td>Coexp_Module_black</td><td>topConnectivityScores</td><td>N_NERVOUS_SYSTEM</td><td>N03_ANTIEPILEPTICS</td><td>N03A_ANTIEPILEPTICS</td><td>N03AG_Fatty_acid_de</td><td>N03AG04_</td></tr>
<tr><td>ZIPRASIDONE</td><td>426345</td><td>2</td><td>P1|P2</td><td>Connectivity_with_SZ_sets</td><td></td><td>Coexp_Module_brown</td><td>topKnownTargetEnrichments</td><td>N_NERVOUS_SYSTEM</td><td>N05_PSYCHOLEPTICS</td><td>N05A_ANTIPSYCHOTIC</td><td>N05AE_Indole_derivati</td><td>N05AE04_</td></tr>
<tr><td>CHLORPROMAZINE.HYDROCHLORIDE</td><td>22389</td><td>1</td><td>P2</td><td>Targets_of_interest</td><td>Promiscuous</td><td></td><td></td><td>N_NERVOUS_SYSTEM</td><td>N05_PSYCHOLEPTICS</td><td>N05A_ANTIPSYCHOTIC</td><td>N05AA_Phenothiazine</td><td>N05AA01_</td></tr>
<tr><td>PHENAZONE</td><td>39902</td><td>1</td><td>P1</td><td>Connectivity_with_SZ_sets</td><td></td><td>Coexp_Module_black</td><td>topConnectivityScores</td><td>N_NERVOUS_SYSTEM|</td><td>N02_ANALGESICS|S02</td><td>N02B_OTHER_ANALGE</td><td>N02BB_Pyrazolones|S</td><td>N02BB01_|S02DA03_</td></tr>
<tr><td>EMETINE</td><td>281962</td><td>1</td><td>P1</td><td>Connectivity_with_SZ_sets</td><td></td><td>Coexp_Module_brown</td><td>topConnectivityScores</td><td>P_ANTIPARASITIC_PRO</td><td>P01_ANTIPROTOZOALS</td><td>P01A_AGENTS_AGAINS</td><td>P01AX_Other_agents_</td><td>P01AX02_</td></tr>
<tr><td>MEBENDAZOLE</td><td>122285</td><td>2</td><td>P1|P2</td><td>Connectivity_with_SZ_sets</td><td></td><td>Coexp_Module_brown</td><td>topConnectivityScores</td><td>P_ANTIPARASITIC_PRO</td><td>P02_ANTHELMINTICS</td><td>P02C_ANTINEMATODA</td><td>P02CA_Benzimidazole_</td><td>P02CA01_</td></tr>
<tr><td>NICLOSAMIDE</td><td>40016</td><td>2</td><td>P1|P2</td><td>Connectivity_with_SZ_sets</td><td></td><td>Coexp_Module_brown</td><td>topConnectivityScores</td><td>P_ANTIPARASITIC_PRO</td><td>P02_ANTHELMINTICS</td><td>P02D_ANTICESTODALS</td><td>P02DA_Salicylic_acid_</td><td>P02DA01_</td></tr>
<tr><td>PRESTWICK.675</td><td>126740</td><td>1</td><td>P1</td><td>Connectivity_with_SZ_sets</td><td></td><td>Coexp_Module_lightcyan|Coexp_Module_black</td><td>topConnectivityScores</td><td>P_ANTIPARASITIC_PRO</td><td>P02_ANTHELMINTICS</td><td>P02C_ANTINEMATODA</td><td>P02CA_Benzimidazole_</td><td>P02CA03_</td></tr>
<tr><td>CHLORCYCLIZINE</td><td>50383</td><td>1</td><td>P1</td><td>Connectivity_with_SZ_sets</td><td></td><td>Coexp_Module_brown</td><td>topConnectivityScores</td><td>R_RESPIRATORY_SYST</td><td>R06_ANTIHISTAMINES_</td><td>R06A_ANTIHISTAMINE</td><td>R06AE_Piperazine_der</td><td>R06AE04_</td></tr>
<tr><td>TYLOXAPOL</td><td>2879388</td><td>1</td><td>P1</td><td>Connectivity_with_SZ_sets</td><td></td><td>Syn-3_0_2312</td><td>TopConnectivityScores</td><td>R_RESPIRATORY_SYST</td><td>R05_COUGH_AND_CO</td><td>R05C_EXPECTORANTS,</td><td>R05CA_Expectorants</td><td>R05CA01_</td></tr>
<tr><td>DORZOLAMIDE.HYDROCHLORIDE</td><td>2887174</td><td>1</td><td>P2</td><td>Targets_of_interest</td><td>Promiscuous</td><td></td><td></td><td>S_SENSORY_ORGANS</td><td>S01_OPHTHALMOLOGI</td><td>S01E_ANTIGLAUCOMA</td><td>S01EC_Carbonic_anhy</td><td>S01EC03_</td></tr>
<tr><td>DICLOFENAMIDE</td><td>2402773</td><td>1</td><td>P1</td><td>Connectivity_with_SZ_sets</td><td></td><td>Coexp_Module_lightcyan|Coexp_Module_black</td><td>topConnectivityScores</td><td>S_SENSORY_ORGANS</td><td>S01_OPHTHALMOLOGI</td><td>S01E_ANTIGLAUCOMA</td><td>S01EC_Carbonic_anhy</td><td>S01EC02_</td></tr>
<tr><td>NALOXONE.HYDROCHLORIDE</td><td>486496</td><td>1</td><td>P2</td><td>Targets_of_interest</td><td>Selective</td><td></td><td></td><td>V_VARIOUS</td><td>V03_ALL_OTHER_THE</td><td>V03A_ALL_OTHER_TH</td><td>V03AB_Antidotes</td><td>V03AB15_</td></tr>
<tr><td>ALPHA.ERGOCRYPTINE</td><td>277784</td><td>1</td><td>P1</td><td>Connectivity_with_SZ_sets</td><td></td><td>Coexp_Module_lightcyan</td><td>topConnectivityScores</td><td></td><td></td><td></td><td></td><td></td></tr>
<tr><td>ANDROSTERONE</td><td>20265</td><td>1</td><td>P2</td><td>Targets_of_interest</td><td>Promiscuous</td><td></td><td></td><td></td><td></td><td></td><td></td><td></td></tr>
<tr><td>ANISOMYCIN</td><td>22948</td><td>1</td><td>P1</td><td>Connectivity_with_SZ_sets</td><td></td><td>Coexp_Module_black|Coexp_Module_brown|Syn-4_0_509|Syn-3_0_2312</td><td>topConnectivityScores|topKnownTargetEnrichments</td><td></td><td></td><td></td><td></td><td></td></tr>
<tr><td>ARACHIDONYLTRIFLUOROMETHANE</td><td>315756</td><td>1</td><td>P1</td><td>Connectivity_with_SZ_sets</td><td></td><td>Coexp_Module_black</td><td>topConnectivityScores</td><td></td><td></td><td></td><td></td><td></td></tr>
<tr><td>ARCAINE</td><td>310490</td><td>1</td><td>P1</td><td>Connectivity_with_SZ_sets</td><td></td><td>Coexp_Module_lightcyan</td><td>topConnectivityScores</td><td></td><td></td><td></td><td></td><td></td></tr>
<tr><td>BAS.012416453</td><td>2234529</td><td>1</td><td>P1</td><td>Connectivity_with_SZ_sets</td><td></td><td>Coexp_Module_lightcyan</td><td>topConnectivityScores</td><td></td><td></td><td></td><td></td><td></td></tr>
<tr><td>BMS.250904</td><td>2191400</td><td>1</td><td>P2</td><td>Targets_of_interest</td><td>Promiscuous</td><td></td><td></td><td></td><td></td><td></td><td></td><td></td></tr>
<tr><td>BERGENIN</td><td>2402706</td><td>1</td><td>P1</td><td>Connectivity_with_SZ_sets</td><td></td><td>Coexp_Module_lightcyan</td><td>topConnectivityScores</td><td></td><td></td><td></td><td></td><td></td></tr>
<tr><td>BUCLADESINE</td><td>99845</td><td>1</td><td>P1</td><td>Connectivity_with_SZ_sets</td><td></td><td>Syn-3_0_2312</td><td>topKnown+PredictedTargetEnrichments</td><td></td><td></td><td></td><td></td><td></td></tr>
<tr><td>CORTICOSTERONE</td><td>30209</td><td>1</td><td>P2</td><td>Targets_of_interest</td><td>Promiscuous</td><td></td><td></td><td></td><td></td><td></td><td></td><td></td></tr>
<tr><td>CELASTROL</td><td>2631718</td><td>2</td><td>P1|P2</td><td>Connectivity_with_SZ_sets</td><td></td><td>Coexp_Module_lightcyan</td><td>topConnectivityScores</td><td></td><td></td><td></td><td></td><td></td></tr>
<tr><td>CEPHAELINE</td><td>11796</td><td>1</td><td>P1</td><td>Connectivity_with_SZ_sets</td><td></td><td>Coexp_Module_brown</td><td>topConnectivityScores</td><td></td><td></td><td></td><td></td><td></td></tr>
<tr><td>ETHOXZOLAMIDE</td><td>509181</td><td>1</td><td>P2</td><td>Targets_of_interest</td><td>Promiscuous</td><td></td><td></td><td></td><td></td><td></td><td></td><td></td></tr>
<tr><td>GABAZINE</td><td>331565</td><td>1</td><td>P2</td><td>Targets_of_interest</td><td>Promiscuous</td><td></td><td></td><td></td><td></td><td></td><td></td><td></td></tr>
<tr><td>HARMINE.HYDROCHLORIDE</td><td>11821</td><td>1</td><td>P2</td><td>Targets_of_interest</td><td>Promiscuous</td><td></td><td></td><td></td><td></td><td></td><td></td><td></td></tr>
<tr><td>KANPAULLONE</td><td>593047</td><td>1</td><td>P2</td><td>Targets_of_interest</td><td>Promiscuous</td><td></td><td></td><td></td><td></td><td></td><td></td><td></td></tr>
<tr><td>MDL.29951</td><td>577014</td><td>1</td><td>P2</td><td>Targets_of_interest</td><td>Promiscuous</td><td></td><td></td><td></td><td></td><td></td><td></td><td></td></tr>
</table>

DL.THIORPHAN	136738	2	P1|P2	Connectivity_with_SZ_sets		Syn-3_0_2312	TopConnectivityScores
PHENELZINE.SULFATE	31562	1	P2	Targets_of_interest	Promiscuous		
POTASSIUM.ESTRONE.SULFATE	23912	1	P2	Targets_of_interest	Promiscuous		
SB.242235	2105401	1	P2	Targets_of_interest	Promiscuous		
URAPIDIL..5.METHYL.	300731	1	P2	Targets_of_interest	Promiscuous		
VATALANIB	580308	1	P2	Targets_of_interest	Promiscuous		
WB.4101.HYDROCHLORIDE	300505	1	P2	Targets_of_interest	Promiscuous		
BENZYLOXYCARBONYL.L.GLYCYL.L.PHEN	56009	1	P2	Targets_of_interest	Selective		
CETOCYCLINE...A.13428.	54929	1	P2	Targets_of_interest	Selective		
CM.ZA.0576	534501	1	P2	Targets_of_interest	Selective		
LIOTHYRONINE	36019	1	P1	Connectivity_with_SZ_sets		Syn-3_5_12	topKnown+PredictedTargetEnrichments
LY379268	Pilot1.2|LY379268	2	P1|P2	Tool_compound			
LYCORINE	589074	2	P1|P2	Connectivity_with_SZ_sets		Coexp_Module_black|Coexp_Module_brown	topConnectivityScores
CONESSINE	17759	1	P2	Targets_of_interest	Selective		
DIHYDROSTILBESTROL	5805	1	P2	Targets_of_interest	Selective		
MDL100907	Pilot1.4|MDL100907	2	P1|P2	Tool_compound			
DIPHENYLAMINOTRIAZINE	3240	1	P2	Targets_of_interest	Selective		
METHYLBENZETHONIUM.CHLORIDE	2402701	1	P1	Connectivity_with_SZ_sets		Coexp_Module_brown	topConnectivityScores
EQUILENIN	5272	1	P2	Targets_of_interest	Selective		
MYELOPEROXIDASE.INHIBITOR.I	16850	1	P1	Connectivity_with_SZ_sets		Coexp_Module_black	topKnown+PredictedTargetEnrichments (MPO)
ETIFENIN	686164	1	P2	Targets_of_interest	Selective		
GLAXO.GR247538	2814631	1	P2	Targets_of_interest	Selective		
NARINGENIN	22473	1	P1	Connectivity_with_SZ_sets		Coexp_Module_black	topConnectivityScores
GT.2394.RAC	2014221	1	P2	Targets_of_interest	Selective		
IOFETAMINE.HYDROCHLORIDE	486362	1	P2	Targets_of_interest	Selective		
PHENYL.BIGUANIDE	155313	1	P1	Connectivity_with_SZ_sets		Coexp_Module_black	topConnectivityScores
PK11195	351291	1	P1	Connectivity_with_SZ_sets		Coexp_Module_black	topKnown+PredictedTargetEnrichments (TSPO)
METHYLPARABEN	13258	1	P2	Targets_of_interest	Selective		
QUINPIROLE	Pilot1.1|quinpirole	2	P1|P2	Tool_compound			
N6.CYCLOPENTYLADENOSINE	486347	1	P2	Targets_of_interest	Selective		
RONIDAZOLE	2778926	1	P1	Connectivity_with_SZ_sets		Syn-3_0_2312	TopConnectivityScores
SALSOLIDIN	15701	2	P1|P2	Connectivity_with_SZ_sets		Coexp_Module_lightcyan	topConnectivityScores
SPIRADOLINE	2430361	1	P1	Connectivity_with_SZ_sets		Coexp_Module_lightcyan	topConnectivityScores
NORETHINDRONE	526577	1	P2	Targets_of_interest	Selective		
TANESPIMYCIN..	Positive Control|PosCon	2	P1|P2	Positive_control			
TCB.2	Pilot1.3|TCB-2	2	P1|P2	Tool_compound			
PERCEPTIN	2021964	1	P2	Targets_of_interest	Selective		
QUIPAZINE..N.METHYL...DIMALEATE	486514	1	P2	Targets_of_interest	Selective		
TRICHOSTATIN.A..	Positive Control|PosCon	2	P1|P2	Positive_control			
TRIMETHOBENZAMIDE	686087	1	P1	Connectivity_with_SZ_sets		Coexp_Module_black	topConnectivityScores
SB.43152	2070306	1	P2	Targets_of_interest	Selective		
UNC0638	3043333	1	P2	Targets_of_interest	Selective		
X3.ACETAMIDOCOUMARIN	276862	2	P1|P2	Connectivity_with_SZ_sets		Coexp_Module_black	topConnectivityScores
X4.HYDROXYPHENAZONE	8571	1	P1	Connectivity_with_SZ_sets		Coexp_Module_lightcyan	topConnectivityScores
ZARDAVERINE	2459550	1	P1	Connectivity_with_SZ_sets		Coexp_Module_brown	topConnectivityScores

Supplementary Table 2. SZ-sets collated across transcriptomic, genetic and protein-centric perspectives.

SZ_Set_Library	SZ_Set	Entrez	Symbol
SZ_Networks	Coexp_Module_black	57786	RBAK
SZ_Networks	Coexp_Module_black	147949	ZNF583
SZ_Networks	Coexp_Module_black	7559	ZNF12
SZ_Networks	Coexp_Module_black	55180	LINS
SZ_Networks	Coexp_Module_black	80321	CEP70
SZ_Networks	Coexp_Module_black	10945	KDELR1
SZ_Networks	Coexp_Module_black	91283	MSANTD3
SZ_Networks	Coexp_Module_black	4760	NEUROD1
SZ_Networks	Coexp_Module_black	4008	LMO7
SZ_Networks	Coexp_Module_black	114783	LMTK3
SZ_Networks	Coexp_Module_black	55275	VPS53
SZ_Networks	Coexp_Module_black	162655	ZNF519
SZ_Networks	Coexp_Module_black	647979	LINC00657
SZ_Networks	Coexp_Module_black	7798	LUZP1
SZ_Networks	Coexp_Module_black	10198	MPHOSPH9
SZ_Networks	Coexp_Module_black	389072	PLEKHM3
SZ_Networks	Coexp_Module_black	6546	SLC8A1
SZ_Networks	Coexp_Module_black	57719	ANO8
SZ_Networks	Coexp_Module_black	401106	LINC00884
SZ_Networks	Coexp_Module_black	2589	GALNT1
SZ_Networks	Coexp_Module_black	642987	TMEM232
SZ_Networks	Coexp_Module_black	256369	LINC00521
SZ_Networks	Coexp_Module_black	57062	DDX24
SZ_Networks	Coexp_Module_black	10956	OS9
SZ_Networks	Coexp_Module_black	10097	ACTR2
SZ_Networks	Coexp_Module_black	3190	HNRNPK
SZ_Networks	Coexp_Module_black	23019	CNOT1
SZ_Networks	Coexp_Module_black	22872	SEC31A
SZ_Networks	Coexp_Module_black	6749	SSRP1
SZ_Networks	Coexp_Module_black	10970	CKAP4
SZ_Networks	Coexp_Module_black	5886	RAD23A
SZ_Networks	Coexp_Module_black	8239	USP9X
SZ_Networks	Coexp_Module_black	10425	ARIH2
SZ_Networks	Coexp_Module_black	6601	SMARCC2
SZ_Networks	Coexp_Module_black	9520	NPEPPS
SZ_Networks	Coexp_Module_black	6240	RRM1
SZ_Networks	Coexp_Module_black	6829	SUPT5H
SZ_Networks	Coexp_Module_black	22985	ACIN1
SZ_Networks	Coexp_Module_black	9685	CLINT1
SZ_Networks	Coexp_Module_black	9049	AIP
SZ_Networks	Coexp_Module_black	8615	USO1
SZ_Networks	Coexp_Module_black	11231	SEC63
SZ_Networks	Coexp_Module_black	55186	SLC25A36
SZ_Networks	Coexp_Module_black	23064	SETX
SZ_Networks	Coexp_Module_black	9897	KIAA0196
SZ_Networks	Coexp_Module_black	224	ALDH3A2
SZ_Networks	Coexp_Module_black	25800	SLC39A6
SZ_Networks	Coexp_Module_black	10659	CELF2
SZ_Networks	Coexp_Module_black	5692	PSMB4
SZ_Networks	Coexp_Module_black	64746	ACBD3
SZ_Networks	Coexp_Module_black	5635	PRPSAP1
SZ_Networks	Coexp_Module_black	9217	VAPB
SZ_Networks	Coexp_Module_black	2776	GNAQ
SZ_Networks	Coexp_Module_black	4204	MECP2
SZ_Networks	Coexp_Module_black	6311	ATXN2

SZ_Networks	Coexp_Module_black	8295	TRRAP
SZ_Networks	Coexp_Module_black	9792	SERTAD2
SZ_Networks	Coexp_Module_black	30836	DNTTIP2
SZ_Networks	Coexp_Module_black	54838	WBP1L
SZ_Networks	Coexp_Module_black	5089	PBX2
SZ_Networks	Coexp_Module_black	22796	COG2
SZ_Networks	Coexp_Module_black	6733	SRPK2
SZ_Networks	Coexp_Module_black	1025	CDK9
SZ_Networks	Coexp_Module_black	80204	FBXO11
SZ_Networks	Coexp_Module_black	23362	PSD3
SZ_Networks	Coexp_Module_black	5685	PSMA4
SZ_Networks	Coexp_Module_black	9757	KMT2B
SZ_Networks	Coexp_Module_black	9743	ARHGAP32
SZ_Networks	Coexp_Module_black	5469	MED1
SZ_Networks	Coexp_Module_black	5537	PPP6C
SZ_Networks	Coexp_Module_black	8603	FAM193A
SZ_Networks	Coexp_Module_black	2002	ELK1
SZ_Networks	Coexp_Module_black	5433	POLR2D
SZ_Networks	Coexp_Module_black	9931	HELZ
SZ_Networks	Coexp_Module_black	1687	DFNA5
SZ_Networks	Coexp_Module_black	5332	PLCB4
SZ_Networks	Coexp_Module_black	4137	MAPT
SZ_Networks	Coexp_Module_black	9371	KIF3B
SZ_Networks	Coexp_Module_black	9923	ZBTB40
SZ_Networks	Coexp_Module_black	5074	PAWR
SZ_Networks	Coexp_Module_black	9749	PHACTR2
SZ_Networks	Coexp_Module_black	116987	AGAP1
SZ_Networks	Coexp_Module_black	10210	TOPORS
SZ_Networks	Coexp_Module_black	6018	RLF
SZ_Networks	Coexp_Module_black	1385	CREB1
SZ_Networks	Coexp_Module_black	25913	POT1
SZ_Networks	Coexp_Module_black	6668	SP2
SZ_Networks	Coexp_Module_black	10978	CLP1
SZ_Networks	Coexp_Module_black	8570	KHSRP
SZ_Networks	Coexp_Module_black	26578	OSTF1
SZ_Networks	Coexp_Module_black	5534	PPP3R1
SZ_Networks	Coexp_Module_black	7699	ZNF140
SZ_Networks	Coexp_Module_black	5170	PDPK1
SZ_Networks	Coexp_Module_black	10573	MRPL28
SZ_Networks	Coexp_Module_black	9527	GOSR1
SZ_Networks	Coexp_Module_black	66036	MTMR9
SZ_Networks	Coexp_Module_black	8411	EEA1
SZ_Networks	Coexp_Module_black	4097	MAFG
SZ_Networks	Coexp_Module_black	10020	GNE
SZ_Networks	Coexp_Module_black	5775	PTPN4
SZ_Networks	Coexp_Module_black	4520	MTF1
SZ_Networks	Coexp_Module_black	6728	SRP19
SZ_Networks	Coexp_Module_black	8975	USP13
SZ_Networks	Coexp_Module_black	10557	RPP38
SZ_Networks	Coexp_Module_black	5608	MAP2K6
SZ_Networks	Coexp_Module_black	8976	WASL
SZ_Networks	Coexp_Module_black	8715	NOL4
SZ_Networks	Coexp_Module_black	10203	CALCRL
SZ_Networks	Coexp_Module_black	1850	DUSP8
SZ_Networks	Coexp_Module_black	2892	GRIA3
SZ_Networks	Coexp_Module_black	80264	ZNF430
SZ_Networks	Coexp_Module_black	9203	ZMYM3
SZ_Networks	Coexp_Module_black	57343	ZNF304

SZ_Networks	Coexp_Module_black	9612	NCOR2
SZ_Networks	Coexp_Module_black	55109	AGGF1
SZ_Networks	Coexp_Module_black	6830	SUPT6H
SZ_Networks	Coexp_Module_black	3746	KCNC1
SZ_Networks	Coexp_Module_black	1983	EIF5
SZ_Networks	Coexp_Module_black	24148	PRPF6
SZ_Networks	Coexp_Module_black	8886	DDX18
SZ_Networks	Coexp_Module_black	8669	EIF3J
SZ_Networks	Coexp_Module_black	23705	CADM1
SZ_Networks	Coexp_Module_black	10957	PNRC1
SZ_Networks	Coexp_Module_black	9733	SART3
SZ_Networks	Coexp_Module_black	9100	USP10
SZ_Networks	Coexp_Module_black	5612	PRKRIR
SZ_Networks	Coexp_Module_black	6602	SMARCD1
SZ_Networks	Coexp_Module_black	8824	CES2
SZ_Networks	Coexp_Module_black	11100	HNRNPUL1
SZ_Networks	Coexp_Module_black	7112	TMPO
SZ_Networks	Coexp_Module_black	10098	TSPAN5
SZ_Networks	Coexp_Module_black	6314	ATXN7
SZ_Networks	Coexp_Module_black	8774	NAPG
SZ_Networks	Coexp_Module_black	51592	TRIM33
SZ_Networks	Coexp_Module_black	9146	HGS
SZ_Networks	Coexp_Module_black	10395	DLC1
SZ_Networks	Coexp_Module_black	3745	KCNB1
SZ_Networks	Coexp_Module_black	3795	KHK
SZ_Networks	Coexp_Module_black	2137	EXTL3
SZ_Networks	Coexp_Module_black	23451	SF3B1
SZ_Networks	Coexp_Module_black	396	ARHGDIA
SZ_Networks	Coexp_Module_black	2778	GNAS
SZ_Networks	Coexp_Module_black	3320	HSP90AA1
SZ_Networks	Coexp_Module_black	23214	XPO6
SZ_Networks	Coexp_Module_black	23633	KPNA6
SZ_Networks	Coexp_Module_black	57178	ZMIZ1
SZ_Networks	Coexp_Module_black	8289	ARID1A
SZ_Networks	Coexp_Module_black	55568	GALNT10
SZ_Networks	Coexp_Module_black	26058	GIGYF2
SZ_Networks	Coexp_Module_black	23512	SUZ12
SZ_Networks	Coexp_Module_black	9522	SCAMP1
SZ_Networks	Coexp_Module_black	6777	STAT5B
SZ_Networks	Coexp_Module_black	23248	RPRD2
SZ_Networks	Coexp_Module_black	57680	CHD8
SZ_Networks	Coexp_Module_black	9716	AQR
SZ_Networks	Coexp_Module_black	23001	WDFY3
SZ_Networks	Coexp_Module_black	987	LRBA
SZ_Networks	Coexp_Module_black	4684	NCAM1
SZ_Networks	Coexp_Module_black	23637	RABGAP1
SZ_Networks	Coexp_Module_black	221443	OARD1
SZ_Networks	Coexp_Module_black	56929	FEM1C
SZ_Networks	Coexp_Module_black	22930	RAB3GAP1
SZ_Networks	Coexp_Module_black	10772	SRSF10
SZ_Networks	Coexp_Module_black	3187	HNRNPH1
SZ_Networks	Coexp_Module_black	221692	PHACTR1
SZ_Networks	Coexp_Module_black	51099	ABHD5
SZ_Networks	Coexp_Module_black	146057	TTBK2
SZ_Networks	Coexp_Module_black	22902	RUFY3
SZ_Networks	Coexp_Module_black	285282	RABL3
SZ_Networks	Coexp_Module_black	11019	LIAS
SZ_Networks	Coexp_Module_black	9554	SEC22B

SZ_Networks	Coexp_Module_black	8496	PPFIBP1
SZ_Networks	Coexp_Module_black	4045	LSAMP
SZ_Networks	Coexp_Module_black	2734	GLG1
SZ_Networks	Coexp_Module_black	1954	MEGF8
SZ_Networks	Coexp_Module_black	221981	THSD7A
SZ_Networks	Coexp_Module_black	4916	NTRK3
SZ_Networks	Coexp_Module_black	55966	AJAP1
SZ_Networks	Coexp_Module_black	9538	EI24
SZ_Networks	Coexp_Module_black	5387	PMS2P3
SZ_Networks	Coexp_Module_black	23253	ANKRD12
SZ_Networks	Coexp_Module_black	10905	MAN1A2
SZ_Networks	Coexp_Module_black	5928	RBBP4
SZ_Networks	Coexp_Module_black	171392	ZNF675
SZ_Networks	Coexp_Module_black	7529	YWHAB
SZ_Networks	Coexp_Module_black	55737	VPS35
SZ_Networks	Coexp_Module_black	51566	ARMCX3
SZ_Networks	Coexp_Module_black	996	CDC27
SZ_Networks	Coexp_Module_black	55705	IPO9
SZ_Networks	Coexp_Module_black	28964	GIT1
SZ_Networks	Coexp_Module_black	55660	PRPF40A
SZ_Networks	Coexp_Module_black	64121	RRAGC
SZ_Networks	Coexp_Module_black	56683	C21orf59
SZ_Networks	Coexp_Module_black	51290	ERGIC2
SZ_Networks	Coexp_Module_black	55745	AP5M1
SZ_Networks	Coexp_Module_black	63893	UBE2O
SZ_Networks	Coexp_Module_black	5202	PFDN2
SZ_Networks	Coexp_Module_black	64062	RBM26
SZ_Networks	Coexp_Module_black	79646	PANK3
SZ_Networks	Coexp_Module_black	26015	RPAP1
SZ_Networks	Coexp_Module_black	55284	UBE2W
SZ_Networks	Coexp_Module_black	55781	RIOK2
SZ_Networks	Coexp_Module_black	55847	CISD1
SZ_Networks	Coexp_Module_black	79654	HECTD3
SZ_Networks	Coexp_Module_black	55252	ASXL2
SZ_Networks	Coexp_Module_black	55206	SBNO1
SZ_Networks	Coexp_Module_black	51318	MRPL35
SZ_Networks	Coexp_Module_black	55278	QRSL1
SZ_Networks	Coexp_Module_black	4649	MYO9A
SZ_Networks	Coexp_Module_black	54433	GAR1
SZ_Networks	Coexp_Module_black	56947	MFF
SZ_Networks	Coexp_Module_black	55279	ZNF654
SZ_Networks	Coexp_Module_black	55112	WDR60
SZ_Networks	Coexp_Module_black	64762	GAREM
SZ_Networks	Coexp_Module_black	23536	ADAT1
SZ_Networks	Coexp_Module_black	64084	CLSTN2
SZ_Networks	Coexp_Module_black	29123	ANKRD11
SZ_Networks	Coexp_Module_black	27087	B3GAT1
SZ_Networks	Coexp_Module_black	51389	RWDD1
SZ_Networks	Coexp_Module_black	51555	PEX5L
SZ_Networks	Coexp_Module_black	51134	CCDC41
SZ_Networks	Coexp_Module_black	56257	MEPCE
SZ_Networks	Coexp_Module_black	11153	FICD
SZ_Networks	Coexp_Module_black	7812	CSDE1
SZ_Networks	Coexp_Module_black	79000	AUNIP
SZ_Networks	Coexp_Module_black	79750	ZNF385D
SZ_Networks	Coexp_Module_black	56660	KCNK12
SZ_Networks	Coexp_Module_black	25769	SLC24A2
SZ_Networks	Coexp_Module_black	60560	NAA35

SZ_Networks	Coexp_Module_black	58528	RRAGD
SZ_Networks	Coexp_Module_black	55209	SETD5
SZ_Networks	Coexp_Module_black	90806	ANGEL2
SZ_Networks	Coexp_Module_black	55108	BSDC1
SZ_Networks	Coexp_Module_black	9994	CASP8AP2
SZ_Networks	Coexp_Module_black	5832	ALDH18A1
SZ_Networks	Coexp_Module_black	9967	THRAP3
SZ_Networks	Coexp_Module_black	55334	SLC39A9
SZ_Networks	Coexp_Module_black	26060	APPL1
SZ_Networks	Coexp_Module_black	65244	SPATS2
SZ_Networks	Coexp_Module_black	79718	TBL1XR1
SZ_Networks	Coexp_Module_black	55870	ASH1L
SZ_Networks	Coexp_Module_black	28966	SNX24
SZ_Networks	Coexp_Module_black	56919	DHX33
SZ_Networks	Coexp_Module_black	9406	ZRANB2
SZ_Networks	Coexp_Module_black	84187	TMEM164
SZ_Networks	Coexp_Module_black	11244	ZHX1
SZ_Networks	Coexp_Module_black	80851	SH3BP5L
SZ_Networks	Coexp_Module_black	57106	NAT14
SZ_Networks	Coexp_Module_black	84937	ZNRF1
SZ_Networks	Coexp_Module_black	84260	TCHP
SZ_Networks	Coexp_Module_black	54823	SWT1
SZ_Networks	Coexp_Module_black	5522	PPP2R2C
SZ_Networks	Coexp_Module_black	114880	OSBPL6
SZ_Networks	Coexp_Module_black	2055	CLN8
SZ_Networks	Coexp_Module_black	57533	TBC1D14
SZ_Networks	Coexp_Module_black	219771	CCNY
SZ_Networks	Coexp_Module_black	54467	ANKIB1
SZ_Networks	Coexp_Module_black	389541	LAMTOR4
SZ_Networks	Coexp_Module_black	90120	C9orf69
SZ_Networks	Coexp_Module_black	80790	CMIP
SZ_Networks	Coexp_Module_black	10640	EXOC5
SZ_Networks	Coexp_Module_black	10152	ABI2
SZ_Networks	Coexp_Module_black	55773	TBC1D23
SZ_Networks	Coexp_Module_black	25871	C3orf17
SZ_Networks	Coexp_Module_black	54439	RBM27
SZ_Networks	Coexp_Module_black	10838	ZNF275
SZ_Networks	Coexp_Module_black	57459	GATAD2B
SZ_Networks	Coexp_Module_black	246184	CDC26
SZ_Networks	Coexp_Module_black	9223	MAGI1
SZ_Networks	Coexp_Module_black	95681	CEP41
SZ_Networks	Coexp_Module_black	124808	CCDC43
SZ_Networks	Coexp_Module_black	4957	ODF2
SZ_Networks	Coexp_Module_black	114327	EFHC1
SZ_Networks	Coexp_Module_black	84440	RAB11FIP4
SZ_Networks	Coexp_Module_black	619208	FAM229B
SZ_Networks	Coexp_Module_black	26057	ANKRD17
SZ_Networks	Coexp_Module_black	83636	C19orf12
SZ_Networks	Coexp_Module_black	91748	ELMSAN1
SZ_Networks	Coexp_Module_black	5599	MAPK8
SZ_Networks	Coexp_Module_black	124245	ZC3H18
SZ_Networks	Coexp_Module_black	93627	TBCK
SZ_Networks	Coexp_Module_black	57455	REXO1
SZ_Networks	Coexp_Module_black	9175	MAP3K13
SZ_Networks	Coexp_Module_black	148867	SLC30A7
SZ_Networks	Coexp_Module_black	84897	TBRG1
SZ_Networks	Coexp_Module_black	80012	PHC3
SZ_Networks	Coexp_Module_black	254225	RNF169

SZ_Networks	Coexp_Module_black	64282	PAPD5
SZ_Networks	Coexp_Module_black	57706	DENND1A
SZ_Networks	Coexp_Module_black	254170	FBXO33
SZ_Networks	Coexp_Module_black	492311	IGIP
SZ_Networks	Coexp_Module_black	10746	MAP3K2
SZ_Networks	Coexp_Module_black	201266	SLC39A11
SZ_Networks	Coexp_Module_black	64839	FBXL17
SZ_Networks	Coexp_Module_black	143384	CACUL1
SZ_Networks	Coexp_Module_black	2017	CTTN
SZ_Networks	Coexp_Module_black	8085	KMT2D
SZ_Networks	Coexp_Module_black	50863	NTM
SZ_Networks	Coexp_Module_black	9589	WTAP
SZ_Networks	Coexp_Module_black	112939	NACC1
SZ_Networks	Coexp_Module_black	10180	RBM6
SZ_Networks	Coexp_Module_black	5500	PPP1CB
SZ_Networks	Coexp_Module_black	253260	RICTOR
SZ_Networks	Coexp_Module_black	55153	SDAD1
SZ_Networks	Coexp_Module_black	387893	SETD8
SZ_Networks	Coexp_Module_black	51616	TAF9B
SZ_Networks	Coexp_Module_black	199870	FAM76A
SZ_Networks	Coexp_Module_black	7068	THRB
SZ_Networks	Coexp_Module_black	11149	BVES
SZ_Networks	Coexp_Module_black	92482	BBIP1
SZ_Networks	Coexp_Module_black	1778	DYNC1H1
SZ_Networks	Coexp_Module_black	6497	SKI
SZ_Networks	Coexp_Module_black	54617	INO80
SZ_Networks	Coexp_Module_black	55088	C10orf118
SZ_Networks	Coexp_Module_black	5802	PTPRS
SZ_Networks	Coexp_Module_black	55604	LRRC16A
SZ_Networks	Coexp_Module_black	572558	PGM5-AS1
SZ_Networks	Coexp_Module_black	151050	KANSL1L
SZ_Networks	Coexp_Module_black	64132	XYLT2
SZ_Networks	Coexp_Module_black	140707	BRI3BP
SZ_Networks	Coexp_Module_black	57505	AARS2
SZ_Networks	Coexp_Module_black	266722	HS6ST3
SZ_Networks	Coexp_Module_black	2186	BPTF
SZ_Networks	Coexp_Module_black	387104	SOGA3
SZ_Networks	Coexp_Module_black	10168	ZNF197
SZ_Networks	Coexp_Module_black	85865	GTPBP10
SZ_Networks	Coexp_Module_black	140612	ZFP28
SZ_Networks	Coexp_Module_black	4289	MKLN1
SZ_Networks	Coexp_Module_black	645212	EIF3J-AS1
SZ_Networks	Coexp_Module_black	653121	ZBTB8A
SZ_Networks	Coexp_Module_black	139322	APOOL
SZ_Networks	Coexp_Module_black	123263	MTFMT
SZ_Networks	Coexp_Module_black	23567	ZNF346
SZ_Networks	Coexp_Module_black	55183	RIF1
SZ_Networks	Coexp_Module_black	401190	RGS7BP
SZ_Networks	Coexp_Module_black	124411	ZNF720
SZ_Networks	Coexp_Module_black	222236	NAPEPLD
SZ_Networks	Coexp_Module_black	117177	RAB3IP
SZ_Networks	Coexp_Module_black	153642	ARSK
SZ_Networks	Coexp_Module_black	5289	PIK3C3
SZ_Networks	Coexp_Module_black	84314	TMEM107
SZ_Networks	Coexp_Module_black	286205	SCAI
SZ_Networks	Coexp_Module_black	814	CAMK4
SZ_Networks	Coexp_Module_black	55680	RUFY2
SZ_Networks	Coexp_Module_black	163081	ZNF567

SZ_Networks	Coexp_Module_black	129563	DIS3L2
SZ_Networks	Coexp_Module_black	673	BRAF
SZ_Networks	Coexp_Module_black	7697	ZNF138
SZ_Networks	Coexp_Module_black	7593	MZF1
SZ_Networks	Coexp_Module_black	54662	TBC1D13
SZ_Networks	Coexp_Module_black	79567	FAM65A
SZ_Networks	Coexp_Module_black	90864	SPSB3
SZ_Networks	Coexp_Module_black	54455	FBXO42
SZ_Networks	Coexp_Module_black	57787	MARK4
SZ_Networks	Coexp_Module_red	130399	ACVR1C
SZ_Networks	Coexp_Module_red	90161	HS6ST2
SZ_Networks	Coexp_Module_red	440944	SETD5-AS1
SZ_Networks	Coexp_Module_red	2786	GNG4
SZ_Networks	Coexp_Module_red	26036	ZNF451
SZ_Networks	Coexp_Module_red	114784	CSMD2
SZ_Networks	Coexp_Module_red	283521	LINC00282
SZ_Networks	Coexp_Module_red	6991	TCTE3
SZ_Networks	Coexp_Module_red	64788	LMF1
SZ_Networks	Coexp_Module_red	6746	SSR2
SZ_Networks	Coexp_Module_red	3337	DNAJB1
SZ_Networks	Coexp_Module_red	2280	FKBP1A
SZ_Networks	Coexp_Module_red	3303	HSPA1A
SZ_Networks	Coexp_Module_red	3329	HSPD1
SZ_Networks	Coexp_Module_red	9611	NCOR1
SZ_Networks	Coexp_Module_red	2288	FKBP4
SZ_Networks	Coexp_Module_red	6386	SDCBP
SZ_Networks	Coexp_Module_red	10904	BLCAP
SZ_Networks	Coexp_Module_red	5954	RCN1
SZ_Networks	Coexp_Module_red	54386	TERF2IP
SZ_Networks	Coexp_Module_red	10625	IVNS1ABP
SZ_Networks	Coexp_Module_red	9868	TOMM70A
SZ_Networks	Coexp_Module_red	8667	EIF3H
SZ_Networks	Coexp_Module_red	8165	AKAP1
SZ_Networks	Coexp_Module_red	5514	PPP1R10
SZ_Networks	Coexp_Module_red	6626	SNRPA
SZ_Networks	Coexp_Module_red	3315	HSPB1
SZ_Networks	Coexp_Module_red	10953	TOMM34
SZ_Networks	Coexp_Module_red	80347	COASY
SZ_Networks	Coexp_Module_red	29889	GNL2
SZ_Networks	Coexp_Module_red	10422	UBAC1
SZ_Networks	Coexp_Module_red	8450	CUL4B
SZ_Networks	Coexp_Module_red	10221	TRIB1
SZ_Networks	Coexp_Module_red	26037	SIPA1L1
SZ_Networks	Coexp_Module_red	4522	MTHFD1
SZ_Networks	Coexp_Module_red	613	BCR
SZ_Networks	Coexp_Module_red	10007	GNPDA1
SZ_Networks	Coexp_Module_red	5997	RGS2
SZ_Networks	Coexp_Module_red	23640	HSPBP1
SZ_Networks	Coexp_Module_red	9663	LPIN2
SZ_Networks	Coexp_Module_red	402	ARL2
SZ_Networks	Coexp_Module_red	7873	MANF
SZ_Networks	Coexp_Module_red	9263	STK17A
SZ_Networks	Coexp_Module_red	7726	TRIM26
SZ_Networks	Coexp_Module_red	8974	P4HA2
SZ_Networks	Coexp_Module_red	9475	ROCK2
SZ_Networks	Coexp_Module_red	7750	ZMYM2
SZ_Networks	Coexp_Module_red	1627	DBN1
SZ_Networks	Coexp_Module_red	54541	DDIT4

SZ_Networks	Coexp_Module_red	9672	SDC3
SZ_Networks	Coexp_Module_red	7466	WFS1
SZ_Networks	Coexp_Module_red	5326	PLAGL2
SZ_Networks	Coexp_Module_red	3632	INPP5A
SZ_Networks	Coexp_Module_red	51363	CHST15
SZ_Networks	Coexp_Module_red	1612	DAPK1
SZ_Networks	Coexp_Module_red	10615	SPAG5
SZ_Networks	Coexp_Module_red	10244	RABEPK
SZ_Networks	Coexp_Module_red	4728	NDUFS8
SZ_Networks	Coexp_Module_red	10263	CDK2AP2
SZ_Networks	Coexp_Module_red	4850	CNOT4
SZ_Networks	Coexp_Module_red	3720	JARID2
SZ_Networks	Coexp_Module_red	22850	ADNP2
SZ_Networks	Coexp_Module_red	2119	ETV5
SZ_Networks	Coexp_Module_red	7162	TPBG
SZ_Networks	Coexp_Module_red	54726	OTUD4
SZ_Networks	Coexp_Module_red	4023	LPL
SZ_Networks	Coexp_Module_red	5092	PCBD1
SZ_Networks	Coexp_Module_red	9658	ZNF516
SZ_Networks	Coexp_Module_red	5116	PCNT
SZ_Networks	Coexp_Module_red	4350	MPG
SZ_Networks	Coexp_Module_red	9828	ARHGEF17
SZ_Networks	Coexp_Module_red	11080	DNAJB4
SZ_Networks	Coexp_Module_red	4217	MAP3K5
SZ_Networks	Coexp_Module_red	5909	RAP1GAP
SZ_Networks	Coexp_Module_red	22880	MORC2
SZ_Networks	Coexp_Module_red	9781	RNF144A
SZ_Networks	Coexp_Module_red	5090	PBX3
SZ_Networks	Coexp_Module_red	6604	SMARCD3
SZ_Networks	Coexp_Module_red	5138	PDE2A
SZ_Networks	Coexp_Module_red	10432	RBM14
SZ_Networks	Coexp_Module_red	10208	USPL1
SZ_Networks	Coexp_Module_red	10926	DBF4
SZ_Networks	Coexp_Module_red	7188	TRAF5
SZ_Networks	Coexp_Module_red	341	APOC1
SZ_Networks	Coexp_Module_red	9825	SPATA2
SZ_Networks	Coexp_Module_red	7862	BRPF1
SZ_Networks	Coexp_Module_red	115	ADCY9
SZ_Networks	Coexp_Module_red	2009	EML1
SZ_Networks	Coexp_Module_red	9254	CACNA2D2
SZ_Networks	Coexp_Module_red	2245	FGD1
SZ_Networks	Coexp_Module_red	7091	TLE4
SZ_Networks	Coexp_Module_red	6664	SOX11
SZ_Networks	Coexp_Module_red	4300	MLLT3
SZ_Networks	Coexp_Module_red	55922	NKRF
SZ_Networks	Coexp_Module_red	3815	KIT
SZ_Networks	Coexp_Module_red	3682	ITGAE
SZ_Networks	Coexp_Module_red	5915	RARB
SZ_Networks	Coexp_Module_red	10090	UST
SZ_Networks	Coexp_Module_red	41	ASIC1
SZ_Networks	Coexp_Module_red	4330	MN1
SZ_Networks	Coexp_Module_red	744	MPPED2
SZ_Networks	Coexp_Module_red	3208	HPCA
SZ_Networks	Coexp_Module_red	5502	PPP1R1A
SZ_Networks	Coexp_Module_red	10570	DPYSL4
SZ_Networks	Coexp_Module_red	22824	HSPA4L
SZ_Networks	Coexp_Module_red	5121	PCP4
SZ_Networks	Coexp_Module_red	793	CALB1

SZ_Networks	Coexp_Module_red	27253	PCDH17
SZ_Networks	Coexp_Module_red	1730	DIAPH2
SZ_Networks	Coexp_Module_red	5587	PRKD1
SZ_Networks	Coexp_Module_red	5649	RELN
SZ_Networks	Coexp_Module_red	6258	RXRG
SZ_Networks	Coexp_Module_red	9365	KL
SZ_Networks	Coexp_Module_red	956	ENTPD3
SZ_Networks	Coexp_Module_red	2774	GNAL
SZ_Networks	Coexp_Module_red	8853	ASAP2
SZ_Networks	Coexp_Module_red	3642	INSM1
SZ_Networks	Coexp_Module_red	8787	RGS9
SZ_Networks	Coexp_Module_red	6863	TAC1
SZ_Networks	Coexp_Module_red	6496	SIX3
SZ_Networks	Coexp_Module_red	3759	KCNJ2
SZ_Networks	Coexp_Module_red	5021	OXTR
SZ_Networks	Coexp_Module_red	10808	HSPH1
SZ_Networks	Coexp_Module_red	3751	KCND2
SZ_Networks	Coexp_Module_red	1009	CDH11
SZ_Networks	Coexp_Module_red	2842	GPR19
SZ_Networks	Coexp_Module_red	3358	HTR2C
SZ_Networks	Coexp_Module_red	4212	MEIS2
SZ_Networks	Coexp_Module_red	5033	P4HA1
SZ_Networks	Coexp_Module_red	3360	HTR4
SZ_Networks	Coexp_Module_red	22981	NINL
SZ_Networks	Coexp_Module_red	9921	RNF10
SZ_Networks	Coexp_Module_red	10296	MAEA
SZ_Networks	Coexp_Module_red	9519	TBPL1
SZ_Networks	Coexp_Module_red	87	ACTN1
SZ_Networks	Coexp_Module_red	23344	ESYT1
SZ_Networks	Coexp_Module_red	7150	TOP1
SZ_Networks	Coexp_Module_red	5447	POR
SZ_Networks	Coexp_Module_red	10262	SF3B4
SZ_Networks	Coexp_Module_red	55651	NHP2
SZ_Networks	Coexp_Module_red	1729	DIAPH1
SZ_Networks	Coexp_Module_red	688	KLF5
SZ_Networks	Coexp_Module_red	8408	ULK1
SZ_Networks	Coexp_Module_red	2887	GRB10
SZ_Networks	Coexp_Module_red	23345	SYNE1
SZ_Networks	Coexp_Module_red	23154	NCDN
SZ_Networks	Coexp_Module_red	10391	CORO2B
SZ_Networks	Coexp_Module_red	9750	FAM65B
SZ_Networks	Coexp_Module_red	54674	LRRN3
SZ_Networks	Coexp_Module_red	8815	BANF1
SZ_Networks	Coexp_Module_red	8648	NCOA1
SZ_Networks	Coexp_Module_red	6328	SCN3A
SZ_Networks	Coexp_Module_red	10899	JTB
SZ_Networks	Coexp_Module_red	2172	FABP6
SZ_Networks	Coexp_Module_red	27101	CACYBP
SZ_Networks	Coexp_Module_red	23089	PEG10
SZ_Networks	Coexp_Module_red	23028	KDM1A
SZ_Networks	Coexp_Module_red	271	AMPD2
SZ_Networks	Coexp_Module_red	51029	DESI2
SZ_Networks	Coexp_Module_red	439921	MXRA7
SZ_Networks	Coexp_Module_red	10252	SPRY1
SZ_Networks	Coexp_Module_red	114882	OSBPL8
SZ_Networks	Coexp_Module_red	26053	AUTS2
SZ_Networks	Coexp_Module_red	23180	RFTN1
SZ_Networks	Coexp_Module_red	23174	ZCCHC14

SZ_Networks	Coexp_Module_red	22907	DHX30
SZ_Networks	Coexp_Module_red	55818	KDM3A
SZ_Networks	Coexp_Module_red	23195	MDN1
SZ_Networks	Coexp_Module_red	23210	JMJD6
SZ_Networks	Coexp_Module_red	23271	CAMSAP2
SZ_Networks	Coexp_Module_red	221061	FAM171A1
SZ_Networks	Coexp_Module_red	4665	NAB2
SZ_Networks	Coexp_Module_red	23268	DNMBP
SZ_Networks	Coexp_Module_red	6942	TCF20
SZ_Networks	Coexp_Module_red	5986	RFNG
SZ_Networks	Coexp_Module_red	3265	HRAS
SZ_Networks	Coexp_Module_red	23286	WWC1
SZ_Networks	Coexp_Module_red	7074	TIAM1
SZ_Networks	Coexp_Module_red	144699	FBXL14
SZ_Networks	Coexp_Module_red	6091	ROBO1
SZ_Networks	Coexp_Module_red	9881	TRANK1
SZ_Networks	Coexp_Module_red	57146	TMEM159
SZ_Networks	Coexp_Module_red	26011	TENM4
SZ_Networks	Coexp_Module_red	25907	TMEM158
SZ_Networks	Coexp_Module_red	23023	TMCC1
SZ_Networks	Coexp_Module_red	26098	C10orf137
SZ_Networks	Coexp_Module_red	4862	NPAS2
SZ_Networks	Coexp_Module_red	8153	RND2
SZ_Networks	Coexp_Module_red	5179	PENK
SZ_Networks	Coexp_Module_red	51491	NOP16
SZ_Networks	Coexp_Module_red	9260	PDLIM7
SZ_Networks	Coexp_Module_red	2138	EYA1
SZ_Networks	Coexp_Module_red	168544	ZNF467
SZ_Networks	Coexp_Module_red	157627	LINC00599
SZ_Networks	Coexp_Module_red	9508	ADAMTS3
SZ_Networks	Coexp_Module_red	54874	FNBP1L
SZ_Networks	Coexp_Module_red	63982	ANO3
SZ_Networks	Coexp_Module_red	10474	TADA3
SZ_Networks	Coexp_Module_red	9077	DIRAS3
SZ_Networks	Coexp_Module_red	23085	ERC1
SZ_Networks	Coexp_Module_red	91752	ZNF804A
SZ_Networks	Coexp_Module_red	771	CA12
SZ_Networks	Coexp_Module_red	22987	SV2C
SZ_Networks	Coexp_Module_red	25876	SPEF1
SZ_Networks	Coexp_Module_red	1813	DRD2
SZ_Networks	Coexp_Module_red	23178	PASK
SZ_Networks	Coexp_Module_red	51108	METTL9
SZ_Networks	Coexp_Module_red	9531	BAG3
SZ_Networks	Coexp_Module_red	55505	NOP10
SZ_Networks	Coexp_Module_red	22822	PHLDA1
SZ_Networks	Coexp_Module_red	5763	PTMS
SZ_Networks	Coexp_Module_red	29099	COMMD9
SZ_Networks	Coexp_Module_red	51447	IP6K2
SZ_Networks	Coexp_Module_red	2872	MKNK2
SZ_Networks	Coexp_Module_red	4088	SMAD3
SZ_Networks	Coexp_Module_red	57162	PELI1
SZ_Networks	Coexp_Module_red	54539	NDUFB11
SZ_Networks	Coexp_Module_red	23589	CARHSP1
SZ_Networks	Coexp_Module_red	54994	GID8
SZ_Networks	Coexp_Module_red	65981	CAPRIN2
SZ_Networks	Coexp_Module_red	79960	PHF17
SZ_Networks	Coexp_Module_red	29098	RANGRF
SZ_Networks	Coexp_Module_red	4696	NDUFA3

SZ_Networks	Coexp_Module_red	26973	CHORDC1
SZ_Networks	Coexp_Module_red	51278	IER5
SZ_Networks	Coexp_Module_red	80324	PUS1
SZ_Networks	Coexp_Module_red	55167	MSL2
SZ_Networks	Coexp_Module_red	5583	PRKCH
SZ_Networks	Coexp_Module_red	54997	TESC
SZ_Networks	Coexp_Module_red	10885	WDR3
SZ_Networks	Coexp_Module_red	55178	RNMTL1
SZ_Networks	Coexp_Module_red	79675	FASTKD1
SZ_Networks	Coexp_Module_red	60526	C2orf43
SZ_Networks	Coexp_Module_red	57419	SLC24A3
SZ_Networks	Coexp_Module_red	79039	DDX54
SZ_Networks	Coexp_Module_red	54980	C2orf42
SZ_Networks	Coexp_Module_red	11275	KLHL2
SZ_Networks	Coexp_Module_red	79589	RNF128
SZ_Networks	Coexp_Module_red	54839	LRRC49
SZ_Networks	Coexp_Module_red	79612	NAA16
SZ_Networks	Coexp_Module_red	55076	TMEM45A
SZ_Networks	Coexp_Module_red	55188	RIC8B
SZ_Networks	Coexp_Module_red	24141	LAMP5
SZ_Networks	Coexp_Module_red	26511	CHIC2
SZ_Networks	Coexp_Module_red	9627	SNCAIP
SZ_Networks	Coexp_Module_red	80758	PRR7
SZ_Networks	Coexp_Module_red	79776	ZFHX4
SZ_Networks	Coexp_Module_red	7561	ZNF14
SZ_Networks	Coexp_Module_red	54971	BANP
SZ_Networks	Coexp_Module_red	51334	PRR16
SZ_Networks	Coexp_Module_red	79772	MCTP1
SZ_Networks	Coexp_Module_red	26507	CNNM1
SZ_Networks	Coexp_Module_red	55791	LRIF1
SZ_Networks	Coexp_Module_red	54112	GPR88
SZ_Networks	Coexp_Module_red	10858	CYP46A1
SZ_Networks	Coexp_Module_red	10777	ARPP21
SZ_Networks	Coexp_Module_red	51078	THAP4
SZ_Networks	Coexp_Module_red	9348	NDST3
SZ_Networks	Coexp_Module_red	84187	TMEM164
SZ_Networks	Coexp_Module_red	51347	TAOK3
SZ_Networks	Coexp_Module_red	63920	C5orf54
SZ_Networks	Coexp_Module_red	112398	EGLN2
SZ_Networks	Coexp_Module_red	26249	KLHL3
SZ_Networks	Coexp_Module_red	60468	BACH2
SZ_Networks	Coexp_Module_red	81558	FAM117A
SZ_Networks	Coexp_Module_red	83480	PUS3
SZ_Networks	Coexp_Module_red	55769	ZNF83
SZ_Networks	Coexp_Module_red	4121	MAN1A1
SZ_Networks	Coexp_Module_red	55603	FAM46A
SZ_Networks	Coexp_Module_red	5128	CDK17
SZ_Networks	Coexp_Module_red	56937	PMEPA1
SZ_Networks	Coexp_Module_red	54836	BSPRY
SZ_Networks	Coexp_Module_red	64776	C11orf1
SZ_Networks	Coexp_Module_red	10678	B3GNT2
SZ_Networks	Coexp_Module_red	55654	TMEM127
SZ_Networks	Coexp_Module_red	64919	BCL11B
SZ_Networks	Coexp_Module_red	51602	NOP58
SZ_Networks	Coexp_Module_red	57189	KIAA1147
SZ_Networks	Coexp_Module_red	83605	CCM2
SZ_Networks	Coexp_Module_red	9069	CLDN12
SZ_Networks	Coexp_Module_red	10194	TSHZ1

SZ_Networks	Coexp_Module_red	51061	TXNDC11
SZ_Networks	Coexp_Module_red	65977	PLEKHA3
SZ_Networks	Coexp_Module_red	84062	DTNBP1
SZ_Networks	Coexp_Module_red	91574	C12orf65
SZ_Networks	Coexp_Module_red	93611	FBXO44
SZ_Networks	Coexp_Module_red	23551	RASD2
SZ_Networks	Coexp_Module_red	30819	KCNIP2
SZ_Networks	Coexp_Module_red	83698	CALN1
SZ_Networks	Coexp_Module_red	56181	MTFR1L
SZ_Networks	Coexp_Module_red	64979	MRPL36
SZ_Networks	Coexp_Module_red	91151	TIGD7
SZ_Networks	Coexp_Module_red	9616	RNF7
SZ_Networks	Coexp_Module_red	84299	MIEN1
SZ_Networks	Coexp_Module_red	92181	UBTD2
SZ_Networks	Coexp_Module_red	80315	CPEB4
SZ_Networks	Coexp_Module_red	27086	FOXP1
SZ_Networks	Coexp_Module_red	1021	CDK6
SZ_Networks	Coexp_Module_red	2035	EPB41
SZ_Networks	Coexp_Module_red	55466	DNAJA4
SZ_Networks	Coexp_Module_red	85364	ZCCHC3
SZ_Networks	Coexp_Module_red	7402	UTRN
SZ_Networks	Coexp_Module_red	84152	PPP1R1B
SZ_Networks	Coexp_Module_red	7871	SLMAP
SZ_Networks	Coexp_Module_red	57038	RARS2
SZ_Networks	Coexp_Module_red	11057	ABHD2
SZ_Networks	Coexp_Module_red	80314	EPC1
SZ_Networks	Coexp_Module_red	162989	DEDD2
SZ_Networks	Coexp_Module_red	55802	DCP1A
SZ_Networks	Coexp_Module_red	85379	KIAA1671
SZ_Networks	Coexp_Module_red	51434	ANAPC7
SZ_Networks	Coexp_Module_red	57685	CACHD1
SZ_Networks	Coexp_Module_red	150275	CCDC117
SZ_Networks	Coexp_Module_red	8470	SORBS2
SZ_Networks	Coexp_Module_red	11320	MGAT4A
SZ_Networks	Coexp_Module_red	80818	ZNF436
SZ_Networks	Coexp_Module_red	80144	FRAS1
SZ_Networks	Coexp_Module_red	221504	ZBTB9
SZ_Networks	Coexp_Module_red	2140	EYA3
SZ_Networks	Coexp_Module_red	51312	SLC25A37
SZ_Networks	Coexp_Module_red	112752	IFT43
SZ_Networks	Coexp_Module_red	57688	ZSWIM6
SZ_Networks	Coexp_Module_red	93624	TADA2B
SZ_Networks	Coexp_Module_red	284252	KCTD1
SZ_Networks	Coexp_Module_red	122953	JDP2
SZ_Networks	Coexp_Module_red	4084	MXD1
SZ_Networks	Coexp_Module_red	57555	NLGN2
SZ_Networks	Coexp_Module_red	80311	KLHL15
SZ_Networks	Coexp_Module_red	50515	CHST11
SZ_Networks	Coexp_Module_red	120534	ARL14EP
SZ_Networks	Coexp_Module_red	222194	RSBN1L
SZ_Networks	Coexp_Module_red	57687	VAT1L
SZ_Networks	Coexp_Module_red	81559	TRIM11
SZ_Networks	Coexp_Module_red	25925	ZNF521
SZ_Networks	Coexp_Module_red	79817	MOB3B
SZ_Networks	Coexp_Module_red	139818	DOCK11
SZ_Networks	Coexp_Module_red	57633	LRRN1
SZ_Networks	Coexp_Module_red	57168	ASPHD2
SZ_Networks	Coexp_Module_red	441212	RP9P

SZ_Networks	Coexp_Module_red	201164	PLD6
SZ_Networks	Coexp_Module_red	80110	ZNF614
SZ_Networks	Coexp_Module_red	145173	B3GALTL
SZ_Networks	Coexp_Module_red	169714	QSOX2
SZ_Networks	Coexp_Module_red	119391	GSTO2
SZ_Networks	Coexp_Module_red	348801	LNP1
SZ_Networks	Coexp_Module_red	57699	CPNE5
SZ_Networks	Coexp_Module_red	84858	ZNF503
SZ_Networks	Coexp_Module_red	440193	CCDC88C
SZ_Networks	Coexp_Module_red	2982	GUCY1A3
SZ_Networks	Coexp_Module_red	22979	EFR3B
SZ_Networks	Coexp_Module_red	148523	C1orf51
SZ_Networks	Coexp_Module_red	100499467	LINC00673
SZ_Networks	Coexp_Module_red	203522	DDX26B
SZ_Networks	Coexp_Module_red	113263	GLCCI1
SZ_Networks	Coexp_Module_red	50937	CDON
SZ_Networks	Coexp_Module_red	91833	WDR20
SZ_Networks	Coexp_Module_red	2674	GFRA1
SZ_Networks	Coexp_Module_red	100131997	FAM27E3
SZ_Networks	Coexp_Module_red	5618	PRLR
SZ_Networks	Coexp_Module_red	57484	RNF150
SZ_Networks	Coexp_Module_red	57631	LRCH2
SZ_Networks	Coexp_Module_red	7770	ZNF227
SZ_Networks	Coexp_Module_red	90293	KLHL13
SZ_Networks	Coexp_Module_red	170394	PWWP2B
SZ_Networks	Coexp_Module_red	152485	ZNF827
SZ_Networks	Coexp_Module_red	115265	DDIT4L
SZ_Networks	Coexp_Module_red	349565	NMNAT3
SZ_Networks	Coexp_Module_red	9678	PHF14
SZ_Networks	Coexp_Module_red	404217	CTXN1
SZ_Networks	Coexp_Module_red	154743	C7orf60
SZ_Networks	Coexp_Module_red	111	ADCY5
SZ_Networks	Coexp_Module_red	114815	SORCS1
SZ_Networks	Coexp_Module_red	375748	ERCC6L2
SZ_Networks	Coexp_Module_red	389362	PSMG4
SZ_Networks	Coexp_Module_red	92092	ZC3HAV1L
SZ_Networks	Coexp_Module_red	57575	PCDH10
SZ_Networks	Coexp_Module_red	90362	FAM110B
SZ_Networks	Coexp_Module_red	2788	GNG7
SZ_Networks	Coexp_Module_red	1602	DACH1
SZ_Networks	Coexp_Module_red	84688	C9orf24
SZ_Networks	Coexp_Module_red	196294	IMMP1L
SZ_Networks	Coexp_Module_red	9671	WSCD2
SZ_Networks	Coexp_Module_red	80325	ABTB1
SZ_Networks	Coexp_Module_red	23765	IL17RA
SZ_Networks	Coexp_Module_red	9099	USP2
SZ_Networks	Coexp_Module_red	84456	L3MBTL3
SZ_Networks	Coexp_Module_red	26207	PITPNC1
SZ_Networks	Coexp_Module_red	124925	SEZ6
SZ_Networks	Coexp_Module_red	55552	ZNF823
SZ_Networks	Coexp_Module_red	283174	MIR4697HG
SZ_Networks	Coexp_Module_red	4986	OPRK1
SZ_Networks	Coexp_Module_red	374887	YJEFN3
SZ_Networks	Coexp_Module_red	89765	RSPH1
SZ_Networks	Coexp_Module_red	27115	PDE7B
SZ_Networks	Coexp_Module_red	55707	NECAP2
SZ_Networks	Coexp_Module_red	134548	SOWAHA
SZ_Networks	Coexp_Module_red	51046	ST8SIA3

SZ_Networks	Coexp_Module_red	132204	SYNPR
SZ_Networks	Coexp_Module_red	6654	SOS1
SZ_Networks	Coexp_Module_red	56256	SERTAD4
SZ_Networks	Coexp_Module_red	10717	AP4B1
SZ_Networks	Coexp_Module_red	57464	STRIP2
SZ_Networks	Coexp_Module_red	90075	ZNF30
SZ_Networks	Coexp_Module_red	57512	GPR158
SZ_Networks	Coexp_Module_red	83875	BCO2
SZ_Networks	Coexp_Module_red	79925	SPEF2
SZ_Networks	Coexp_Module_red	54890	ALKBH5
SZ_Networks	Coexp_Module_red	57186	RALGAPA2
SZ_Networks	Coexp_Module_red	140733	MACROD2
SZ_Networks	Coexp_Module_red	84750	FUT10
SZ_Networks	Coexp_Module_red	124961	ZFP3
SZ_Networks	Coexp_Module_red	120114	FAT3
SZ_Networks	Coexp_Module_red	6330	SCN4B
SZ_Networks	Coexp_Module_red	84867	PTPN5
SZ_Networks	Coexp_Module_red	646113	LINC00643
SZ_Networks	Coexp_Module_red	441054	C4orf47
SZ_Networks	Coexp_Module_red	197259	MLKL
SZ_Networks	Coexp_Module_red	79056	PRRG4
SZ_Networks	Coexp_Module_red	282996	RBM20
SZ_Networks	Coexp_Module_red	347730	LRRTM1
SZ_Networks	Coexp_Module_red	4842	NOS1
SZ_Networks	Coexp_Module_red	147947	ZNF542
SZ_Networks	Coexp_Module_red	441061	MARCH11
SZ_Networks	Coexp_Module_red	386618	KCTD4
SZ_Networks	Coexp_Module_red	114788	CSMD3
SZ_Networks	Coexp_Module_red	84623	KIRREL3
SZ_Networks	Coexp_Module_red	222537	HS3ST5
SZ_Networks	Coexp_Module_red	140679	SLC32A1
SZ_Networks	Coexp_Module_red	654790	PCP4L1
SZ_Networks	Coexp_Module_red	149297	FAM78B
SZ_Networks	Coexp_Module_red	284370	ZNF615
SZ_Networks	Coexp_Module_red	94122	SYTL5
SZ_Networks	Coexp_Module_red	1750	DLX6
SZ_Networks	Coexp_Module_red	148281	SYT6
SZ_Networks	Coexp_Module_red	642938	FAM196A
SZ_Networks	Coexp_Module_red	85397	RGS8
SZ_Networks	Coexp_Module_red	54555	DDX49
SZ_Networks	Coexp_Module_red	7067	THRA
SZ_Networks	Coexp_Module_red	10636	RGS14
SZ_Networks	Coexp_Module_red	23462	HEY1
SZ_Networks	Coexp_Module_red	79903	NAA60
SZ_Networks	Coexp_Module_red	84861	KLHL22
SZ_Networks	Coexp_Module_red	54976	C20orf27
SZ_Networks	Coexp_Module_red	65987	KCTD14
SZ_Networks	Coexp_Module_red	138162	C9orf116
SZ_Networks	Coexp_Module_brown	23170	TTLL12
SZ_Networks	Coexp_Module_brown	54899	PXK
SZ_Networks	Coexp_Module_brown	172	AFG3L1P
SZ_Networks	Coexp_Module_brown	135295	SRSF12
SZ_Networks	Coexp_Module_brown	123591	C15orf27
SZ_Networks	Coexp_Module_brown	158471	PRUNE2
SZ_Networks	Coexp_Module_brown	57524	CASKIN1
SZ_Networks	Coexp_Module_brown	131096	KCNH8
SZ_Networks	Coexp_Module_brown	222950	NYAP1
SZ_Networks	Coexp_Module_brown	163049	ZNF791

SZ_Networks	Coexp_Module_brown	114792	KLHL32
SZ_Networks	Coexp_Module_brown	352954	GATS
SZ_Networks	Coexp_Module_brown	56650	CLDND1
SZ_Networks	Coexp_Module_brown	55293	UEVLD
SZ_Networks	Coexp_Module_brown	1762	DMWD
SZ_Networks	Coexp_Module_brown	220965	FAM13C
SZ_Networks	Coexp_Module_brown	118442	GPR62
SZ_Networks	Coexp_Module_brown	54933	RHBDL2
SZ_Networks	Coexp_Module_brown	2894	GRID1
SZ_Networks	Coexp_Module_brown	56924	PAK6
SZ_Networks	Coexp_Module_brown	57018	CCNL1
SZ_Networks	Coexp_Module_brown	201191	SAMD14
SZ_Networks	Coexp_Module_brown	4670	HNRNPM
SZ_Networks	Coexp_Module_brown	84445	LZTS2
SZ_Networks	Coexp_Module_brown	1604	CD55
SZ_Networks	Coexp_Module_brown	64399	HHIP
SZ_Networks	Coexp_Module_brown	118611	C10orf90
SZ_Networks	Coexp_Module_brown	158228	FAM201A
SZ_Networks	Coexp_Module_brown	150356	CHADL
SZ_Networks	Coexp_Module_brown	6461	SHB
SZ_Networks	Coexp_Module_brown	387486	LINC00320
SZ_Networks	Coexp_Module_brown	494470	RNF165
SZ_Networks	Coexp_Module_brown	100129196	MATN1-AS1
SZ_Networks	Coexp_Module_brown	80313	LRRC27
SZ_Networks	Coexp_Module_brown	114794	ELFN2
SZ_Networks	Coexp_Module_brown	54819	ZCCHC10
SZ_Networks	Coexp_Module_brown	85449	KIAA1755
SZ_Networks	Coexp_Module_brown	162998	OR7D2
SZ_Networks	Coexp_Module_brown	283999	TMEM235
SZ_Networks	Coexp_Module_brown	11116	FGFR1OP
SZ_Networks	Coexp_Module_brown	1603	DAD1
SZ_Networks	Coexp_Module_brown	7705	ZNF146
SZ_Networks	Coexp_Module_brown	23020	SNRNP200
SZ_Networks	Coexp_Module_brown	2987	GUK1
SZ_Networks	Coexp_Module_brown	2339	FNTA
SZ_Networks	Coexp_Module_brown	3192	HNRNPU
SZ_Networks	Coexp_Module_brown	10397	NDRG1
SZ_Networks	Coexp_Module_brown	65108	MARCKSL1
SZ_Networks	Coexp_Module_brown	2934	GSN
SZ_Networks	Coexp_Module_brown	1808	DPYSL2
SZ_Networks	Coexp_Module_brown	4144	MAT2A
SZ_Networks	Coexp_Module_brown	5695	PSMB7
SZ_Networks	Coexp_Module_brown	8682	PEA15
SZ_Networks	Coexp_Module_brown	8826	IQGAP1
SZ_Networks	Coexp_Module_brown	9802	DAZAP2
SZ_Networks	Coexp_Module_brown	539	ATP5O
SZ_Networks	Coexp_Module_brown	6319	SCD
SZ_Networks	Coexp_Module_brown	5908	RAP1B
SZ_Networks	Coexp_Module_brown	9798	IST1
SZ_Networks	Coexp_Module_brown	1974	EIF4A2
SZ_Networks	Coexp_Module_brown	1912	PHC2
SZ_Networks	Coexp_Module_brown	1337	COX6A1
SZ_Networks	Coexp_Module_brown	7913	DEK
SZ_Networks	Coexp_Module_brown	5479	PPIB
SZ_Networks	Coexp_Module_brown	928	CD9
SZ_Networks	Coexp_Module_brown	23451	SF3B1
SZ_Networks	Coexp_Module_brown	8396	PIP4K2B
SZ_Networks	Coexp_Module_brown	1350	COX7C

SZ_Networks	Coexp_Module_brown	1965	EIF2S1
SZ_Networks	Coexp_Module_brown	9698	PUM1
SZ_Networks	Coexp_Module_brown	25793	FBXO7
SZ_Networks	Coexp_Module_brown	262	AMD1
SZ_Networks	Coexp_Module_brown	5111	PCNA
SZ_Networks	Coexp_Module_brown	5358	PLS3
SZ_Networks	Coexp_Module_brown	6625	SNRNP70
SZ_Networks	Coexp_Module_brown	10250	SRRM1
SZ_Networks	Coexp_Module_brown	9167	COX7A2L
SZ_Networks	Coexp_Module_brown	5869	RAB5B
SZ_Networks	Coexp_Module_brown	79602	ADIPOR2
SZ_Networks	Coexp_Module_brown	2319	FLOT2
SZ_Networks	Coexp_Module_brown	1315	COPB1
SZ_Networks	Coexp_Module_brown	9898	UBAP2L
SZ_Networks	Coexp_Module_brown	4077	NBR1
SZ_Networks	Coexp_Module_brown	10181	RBM5
SZ_Networks	Coexp_Module_brown	6449	SGTA
SZ_Networks	Coexp_Module_brown	156	ADRBK1
SZ_Networks	Coexp_Module_brown	8314	BAP1
SZ_Networks	Coexp_Module_brown	6414	SEPP1
SZ_Networks	Coexp_Module_brown	1340	COX6B1
SZ_Networks	Coexp_Module_brown	6009	RHEB
SZ_Networks	Coexp_Module_brown	6888	TALDO1
SZ_Networks	Coexp_Module_brown	6723	SRM
SZ_Networks	Coexp_Module_brown	9296	ATP6V1F
SZ_Networks	Coexp_Module_brown	6117	RPA1
SZ_Networks	Coexp_Module_brown	3916	LAMP1
SZ_Networks	Coexp_Module_brown	25932	CLIC4
SZ_Networks	Coexp_Module_brown	27089	UQCRQ
SZ_Networks	Coexp_Module_brown	22938	SNW1
SZ_Networks	Coexp_Module_brown	10212	DDX39A
SZ_Networks	Coexp_Module_brown	9352	TXNL1
SZ_Networks	Coexp_Module_brown	1347	COX7A2
SZ_Networks	Coexp_Module_brown	10935	PRDX3
SZ_Networks	Coexp_Module_brown	1967	EIF2B1
SZ_Networks	Coexp_Module_brown	3371	TNC
SZ_Networks	Coexp_Module_brown	2181	ACSL3
SZ_Networks	Coexp_Module_brown	740	MRPL49
SZ_Networks	Coexp_Module_brown	6446	SGK1
SZ_Networks	Coexp_Module_brown	1345	COX6C
SZ_Networks	Coexp_Module_brown	55884	WSB2
SZ_Networks	Coexp_Module_brown	3073	HEXA
SZ_Networks	Coexp_Module_brown	9918	NCAPD2
SZ_Networks	Coexp_Module_brown	11342	RNF13
SZ_Networks	Coexp_Module_brown	6035	RNASE1
SZ_Networks	Coexp_Module_brown	1717	DHCR7
SZ_Networks	Coexp_Module_brown	5564	PRKAB1
SZ_Networks	Coexp_Module_brown	3988	LIPA
SZ_Networks	Coexp_Module_brown	51035	UBXN1
SZ_Networks	Coexp_Module_brown	84722	PSRC1
SZ_Networks	Coexp_Module_brown	214	ALCAM
SZ_Networks	Coexp_Module_brown	2180	ACSL1
SZ_Networks	Coexp_Module_brown	4678	NASP
SZ_Networks	Coexp_Module_brown	1389	CREBL2
SZ_Networks	Coexp_Module_brown	4700	NDUFA6
SZ_Networks	Coexp_Module_brown	439	ASNA1
SZ_Networks	Coexp_Module_brown	4122	MAN2A2
SZ_Networks	Coexp_Module_brown	9821	RB1CC1

SZ_Networks	Coexp_Module_brown	6422	SFRP1
SZ_Networks	Coexp_Module_brown	26064	RAI14
SZ_Networks	Coexp_Module_brown	9414	TJP2
SZ_Networks	Coexp_Module_brown	10975	UQCR11
SZ_Networks	Coexp_Module_brown	23568	ARL2BP
SZ_Networks	Coexp_Module_brown	54623	PAF1
SZ_Networks	Coexp_Module_brown	5184	PEPD
SZ_Networks	Coexp_Module_brown	1349	COX7B
SZ_Networks	Coexp_Module_brown	5977	DPF2
SZ_Networks	Coexp_Module_brown	392	ARHGAP1
SZ_Networks	Coexp_Module_brown	8021	NUP214
SZ_Networks	Coexp_Module_brown	7716	VEZF1
SZ_Networks	Coexp_Module_brown	5108	PCM1
SZ_Networks	Coexp_Module_brown	8985	PLOD3
SZ_Networks	Coexp_Module_brown	50999	TMED5
SZ_Networks	Coexp_Module_brown	8897	MTMR3
SZ_Networks	Coexp_Module_brown	4047	LSS
SZ_Networks	Coexp_Module_brown	9129	PRPF3
SZ_Networks	Coexp_Module_brown	1785	DNM2
SZ_Networks	Coexp_Module_brown	522	ATP5J
SZ_Networks	Coexp_Module_brown	11243	PMF1
SZ_Networks	Coexp_Module_brown	5906	RAP1A
SZ_Networks	Coexp_Module_brown	6695	SPOCK1
SZ_Networks	Coexp_Module_brown	7398	USP1
SZ_Networks	Coexp_Module_brown	9817	KEAP1
SZ_Networks	Coexp_Module_brown	3321	IGSF3
SZ_Networks	Coexp_Module_brown	7994	KAT6A
SZ_Networks	Coexp_Module_brown	3300	DNAJB2
SZ_Networks	Coexp_Module_brown	26097	CHTOP
SZ_Networks	Coexp_Module_brown	8658	TNKS
SZ_Networks	Coexp_Module_brown	7298	TYMS
SZ_Networks	Coexp_Module_brown	9282	MED14
SZ_Networks	Coexp_Module_brown	7844	RNF103
SZ_Networks	Coexp_Module_brown	403	ARL3
SZ_Networks	Coexp_Module_brown	7456	WIPF1
SZ_Networks	Coexp_Module_brown	2958	GTF2A2
SZ_Networks	Coexp_Module_brown	4864	NPC1
SZ_Networks	Coexp_Module_brown	29890	RBM15B
SZ_Networks	Coexp_Module_brown	1327	COX4I1
SZ_Networks	Coexp_Module_brown	10140	TOB1
SZ_Networks	Coexp_Module_brown	8349	HIST2H2BE
SZ_Networks	Coexp_Module_brown	25804	LSM4
SZ_Networks	Coexp_Module_brown	6575	SLC20A2
SZ_Networks	Coexp_Module_brown	25920	NELFB
SZ_Networks	Coexp_Module_brown	836	CASP3
SZ_Networks	Coexp_Module_brown	2200	FBN1
SZ_Networks	Coexp_Module_brown	6433	SFSWAP
SZ_Networks	Coexp_Module_brown	3628	INPP1
SZ_Networks	Coexp_Module_brown	10617	STAMBP
SZ_Networks	Coexp_Module_brown	2548	GAA
SZ_Networks	Coexp_Module_brown	10614	HEXIM1
SZ_Networks	Coexp_Module_brown	196	AHR
SZ_Networks	Coexp_Module_brown	7307	U2AF1
SZ_Networks	Coexp_Module_brown	9909	DENND4B
SZ_Networks	Coexp_Module_brown	9267	CYTH1
SZ_Networks	Coexp_Module_brown	5519	PPP2R1B
SZ_Networks	Coexp_Module_brown	9053	MAP7
SZ_Networks	Coexp_Module_brown	2956	MSH6

SZ_Networks	Coexp_Module_brown	2729	GCLC
SZ_Networks	Coexp_Module_brown	9551	ATP5J2
SZ_Networks	Coexp_Module_brown	23303	KIF13B
SZ_Networks	Coexp_Module_brown	51421	AMOTL2
SZ_Networks	Coexp_Module_brown	56951	C5orf15
SZ_Networks	Coexp_Module_brown	7390	UROS
SZ_Networks	Coexp_Module_brown	5796	PTPRK
SZ_Networks	Coexp_Module_brown	9656	MDC1
SZ_Networks	Coexp_Module_brown	7869	SEMA3B
SZ_Networks	Coexp_Module_brown	7058	THBS2
SZ_Networks	Coexp_Module_brown	9425	CDYL
SZ_Networks	Coexp_Module_brown	2235	FECH
SZ_Networks	Coexp_Module_brown	9924	PAN2
SZ_Networks	Coexp_Module_brown	4891	SLC11A2
SZ_Networks	Coexp_Module_brown	3800	KIF5C
SZ_Networks	Coexp_Module_brown	8520	HAT1
SZ_Networks	Coexp_Module_brown	4130	MAP1A
SZ_Networks	Coexp_Module_brown	10428	CFDP1
SZ_Networks	Coexp_Module_brown	57020	C16orf62
SZ_Networks	Coexp_Module_brown	9770	RASSF2
SZ_Networks	Coexp_Module_brown	10058	ABCB6
SZ_Networks	Coexp_Module_brown	4552	MTRR
SZ_Networks	Coexp_Module_brown	1855	DVL1
SZ_Networks	Coexp_Module_brown	22828	SCAF8
SZ_Networks	Coexp_Module_brown	11334	TUSC2
SZ_Networks	Coexp_Module_brown	22838	RNF44
SZ_Networks	Coexp_Module_brown	9710	KIAA0355
SZ_Networks	Coexp_Module_brown	6814	STXBP3
SZ_Networks	Coexp_Module_brown	8440	NCK2
SZ_Networks	Coexp_Module_brown	858	CAV2
SZ_Networks	Coexp_Module_brown	3416	IDE
SZ_Networks	Coexp_Module_brown	5529	PPP2R5E
SZ_Networks	Coexp_Module_brown	9776	ATG13
SZ_Networks	Coexp_Module_brown	4709	NDUFB3
SZ_Networks	Coexp_Module_brown	51585	PCF11
SZ_Networks	Coexp_Module_brown	1606	DGKA
SZ_Networks	Coexp_Module_brown	7018	TF
SZ_Networks	Coexp_Module_brown	5947	RBP1
SZ_Networks	Coexp_Module_brown	5663	PSEN1
SZ_Networks	Coexp_Module_brown	5372	PMM1
SZ_Networks	Coexp_Module_brown	324	APC
SZ_Networks	Coexp_Module_brown	9670	IPO13
SZ_Networks	Coexp_Module_brown	11183	MAP4K5
SZ_Networks	Coexp_Module_brown	9232	PTTG1
SZ_Networks	Coexp_Module_brown	9638	FEZ1
SZ_Networks	Coexp_Module_brown	6878	TAF6
SZ_Networks	Coexp_Module_brown	4711	NDUFB5
SZ_Networks	Coexp_Module_brown	2263	FGFR2
SZ_Networks	Coexp_Module_brown	10150	MBNL2
SZ_Networks	Coexp_Module_brown	9765	ZFYVE16
SZ_Networks	Coexp_Module_brown	22909	FAN1
SZ_Networks	Coexp_Module_brown	5311	PKD2
SZ_Networks	Coexp_Module_brown	5142	PDE4B
SZ_Networks	Coexp_Module_brown	6484	ST3GAL4
SZ_Networks	Coexp_Module_brown	25801	GCA
SZ_Networks	Coexp_Module_brown	10165	SLC25A13
SZ_Networks	Coexp_Module_brown	9553	MRPL33
SZ_Networks	Coexp_Module_brown	605	BCL7A

SZ_Networks	Coexp_Module_brown	1839	HBEGF
SZ_Networks	Coexp_Module_brown	5998	RGS3
SZ_Networks	Coexp_Module_brown	22864	R3HDM2
SZ_Networks	Coexp_Module_brown	22924	MAPRE3
SZ_Networks	Coexp_Module_brown	64854	USP46
SZ_Networks	Coexp_Module_brown	23334	SZT2
SZ_Networks	Coexp_Module_brown	9922	IQSEC1
SZ_Networks	Coexp_Module_brown	9727	RAB11FIP3
SZ_Networks	Coexp_Module_brown	1876	E2F6
SZ_Networks	Coexp_Module_brown	2530	FUT8
SZ_Networks	Coexp_Module_brown	7403	KDM6A
SZ_Networks	Coexp_Module_brown	9319	TRIP13
SZ_Networks	Coexp_Module_brown	4211	MEIS1
SZ_Networks	Coexp_Module_brown	745	MYRF
SZ_Networks	Coexp_Module_brown	1938	EEF2
SZ_Networks	Coexp_Module_brown	55556	ENOSF1
SZ_Networks	Coexp_Module_brown	22875	ENPP4
SZ_Networks	Coexp_Module_brown	1371	CPOX
SZ_Networks	Coexp_Module_brown	1318	SLC31A2
SZ_Networks	Coexp_Module_brown	8310	ACOX3
SZ_Networks	Coexp_Module_brown	1119	CHKA
SZ_Networks	Coexp_Module_brown	9895	TECPR2
SZ_Networks	Coexp_Module_brown	11322	TMC6
SZ_Networks	Coexp_Module_brown	175	AGA
SZ_Networks	Coexp_Module_brown	1307	COL16A1
SZ_Networks	Coexp_Module_brown	4669	NAGLU
SZ_Networks	Coexp_Module_brown	2976	GTF3C2
SZ_Networks	Coexp_Module_brown	2869	GRK5
SZ_Networks	Coexp_Module_brown	24139	EML2
SZ_Networks	Coexp_Module_brown	23046	KIF21B
SZ_Networks	Coexp_Module_brown	79095	C9orf16
SZ_Networks	Coexp_Module_brown	5287	PIK3C2B
SZ_Networks	Coexp_Module_brown	5144	PDE4D
SZ_Networks	Coexp_Module_brown	8479	HIRIP3
SZ_Networks	Coexp_Module_brown	51090	PLLP
SZ_Networks	Coexp_Module_brown	22873	DZIP1
SZ_Networks	Coexp_Module_brown	8493	PPM1D
SZ_Networks	Coexp_Module_brown	22863	ATG14
SZ_Networks	Coexp_Module_brown	3897	L1CAM
SZ_Networks	Coexp_Module_brown	11007	CCDC85B
SZ_Networks	Coexp_Module_brown	10495	ENOX2
SZ_Networks	Coexp_Module_brown	9738	CCP110
SZ_Networks	Coexp_Module_brown	9771	RAPGEF5
SZ_Networks	Coexp_Module_brown	11135	CDC42EP1
SZ_Networks	Coexp_Module_brown	10351	ABCA8
SZ_Networks	Coexp_Module_brown	5653	KLK6
SZ_Networks	Coexp_Module_brown	2123	EVI2A
SZ_Networks	Coexp_Module_brown	4118	MAL
SZ_Networks	Coexp_Module_brown	5422	POLA1
SZ_Networks	Coexp_Module_brown	27030	MLH3
SZ_Networks	Coexp_Module_brown	5576	PRKAR2A
SZ_Networks	Coexp_Module_brown	1641	DCX
SZ_Networks	Coexp_Module_brown	4832	NME3
SZ_Networks	Coexp_Module_brown	3249	HPN
SZ_Networks	Coexp_Module_brown	2705	GJB1
SZ_Networks	Coexp_Module_brown	79090	TRAPPC6A
SZ_Networks	Coexp_Module_brown	7039	TGFA
SZ_Networks	Coexp_Module_brown	10124	ARL4A

SZ_Networks	Coexp_Module_brown	9134	CCNE2
SZ_Networks	Coexp_Module_brown	23542	MAPK8IP2
SZ_Networks	Coexp_Module_brown	9751	SNPH
SZ_Networks	Coexp_Module_brown	6712	SPTBN2
SZ_Networks	Coexp_Module_brown	7746	ZSCAN9
SZ_Networks	Coexp_Module_brown	50855	PARD6A
SZ_Networks	Coexp_Module_brown	580	BARD1
SZ_Networks	Coexp_Module_brown	2593	GAMT
SZ_Networks	Coexp_Module_brown	8540	AGPS
SZ_Networks	Coexp_Module_brown	3290	HSD11B1
SZ_Networks	Coexp_Module_brown	10420	TESK2
SZ_Networks	Coexp_Module_brown	1404	HAPLN1
SZ_Networks	Coexp_Module_brown	8534	CHST1
SZ_Networks	Coexp_Module_brown	51375	SNX7
SZ_Networks	Coexp_Module_brown	3269	HRH1
SZ_Networks	Coexp_Module_brown	10439	OLFM1
SZ_Networks	Coexp_Module_brown	5638	PRRG1
SZ_Networks	Coexp_Module_brown	1392	CRH
SZ_Networks	Coexp_Module_brown	6457	SH3GL3
SZ_Networks	Coexp_Module_brown	11069	RAPGEF4
SZ_Networks	Coexp_Module_brown	4036	LRP2
SZ_Networks	Coexp_Module_brown	79171	RBM42
SZ_Networks	Coexp_Module_brown	25780	RASGRP3
SZ_Networks	Coexp_Module_brown	9256	BZRAP1
SZ_Networks	Coexp_Module_brown	9832	JAKMIP2
SZ_Networks	Coexp_Module_brown	10224	ZNF443
SZ_Networks	Coexp_Module_brown	5334	PLCL1
SZ_Networks	Coexp_Module_brown	5365	PLXNB3
SZ_Networks	Coexp_Module_brown	9625	AATK
SZ_Networks	Coexp_Module_brown	7130	TNFAIP6
SZ_Networks	Coexp_Module_brown	443	ASPA
SZ_Networks	Coexp_Module_brown	7644	ZNF91
SZ_Networks	Coexp_Module_brown	9705	ST18
SZ_Networks	Coexp_Module_brown	9699	RIMS2
SZ_Networks	Coexp_Module_brown	5298	PI4KB
SZ_Networks	Coexp_Module_brown	116986	AGAP2
SZ_Networks	Coexp_Module_brown	8633	UNC5C
SZ_Networks	Coexp_Module_brown	3780	KCNN1
SZ_Networks	Coexp_Module_brown	4145	MATK
SZ_Networks	Coexp_Module_brown	53358	SHC3
SZ_Networks	Coexp_Module_brown	7545	ZIC1
SZ_Networks	Coexp_Module_brown	9745	ZNF536
SZ_Networks	Coexp_Module_brown	23639	LRRC6
SZ_Networks	Coexp_Module_brown	4051	CYP4F3
SZ_Networks	Coexp_Module_brown	7432	VIP
SZ_Networks	Coexp_Module_brown	10693	CCT6B
SZ_Networks	Coexp_Module_brown	40	ASIC2
SZ_Networks	Coexp_Module_brown	9142	TMEM257
SZ_Networks	Coexp_Module_brown	23443	SLC35A3
SZ_Networks	Coexp_Module_brown	4707	NDUFB1
SZ_Networks	Coexp_Module_brown	10013	HDAC6
SZ_Networks	Coexp_Module_brown	10633	RASL10A
SZ_Networks	Coexp_Module_brown	28513	CDH19
SZ_Networks	Coexp_Module_brown	4821	NKX2-2
SZ_Networks	Coexp_Module_brown	2555	GABRA2
SZ_Networks	Coexp_Module_brown	8402	SLC25A11
SZ_Networks	Coexp_Module_brown	5027	P2RX7
SZ_Networks	Coexp_Module_brown	4974	OMG

SZ_Networks	Coexp_Module_brown	5296	PIK3R2
SZ_Networks	Coexp_Module_brown	5046	PCSK6
SZ_Networks	Coexp_Module_brown	5394	EXOSC10
SZ_Networks	Coexp_Module_brown	8525	DGKZ
SZ_Networks	Coexp_Module_brown	7439	BEST1
SZ_Networks	Coexp_Module_brown	3094	HINT1
SZ_Networks	Coexp_Module_brown	55643	BTBD2
SZ_Networks	Coexp_Module_brown	272	AMPD3
SZ_Networks	Coexp_Module_brown	863	CBFA2T3
SZ_Networks	Coexp_Module_brown	81552	VOPP1
SZ_Networks	Coexp_Module_brown	5662	PSD
SZ_Networks	Coexp_Module_brown	83475	DOHH
SZ_Networks	Coexp_Module_brown	3991	LIPE
SZ_Networks	Coexp_Module_brown	238	ALK
SZ_Networks	Coexp_Module_brown	7368	UGT8
SZ_Networks	Coexp_Module_brown	3631	INPP4A
SZ_Networks	Coexp_Module_brown	2317	FLNB
SZ_Networks	Coexp_Module_brown	142	PARP1
SZ_Networks	Coexp_Module_brown	2222	FDFT1
SZ_Networks	Coexp_Module_brown	10109	ARPC2
SZ_Networks	Coexp_Module_brown	10521	DDX17
SZ_Networks	Coexp_Module_brown	10632	ATP5L
SZ_Networks	Coexp_Module_brown	23385	NCSTN
SZ_Networks	Coexp_Module_brown	517	ATP5G2
SZ_Networks	Coexp_Module_brown	4179	CD46
SZ_Networks	Coexp_Module_brown	1107	CHD3
SZ_Networks	Coexp_Module_brown	5747	PTK2
SZ_Networks	Coexp_Module_brown	5127	CDK16
SZ_Networks	Coexp_Module_brown	26003	GORASP2
SZ_Networks	Coexp_Module_brown	1267	CNP
SZ_Networks	Coexp_Module_brown	1397	CRIP2
SZ_Networks	Coexp_Module_brown	3189	HNRNPH3
SZ_Networks	Coexp_Module_brown	10006	ABI1
SZ_Networks	Coexp_Module_brown	10938	EHD1
SZ_Networks	Coexp_Module_brown	10539	GLRX3
SZ_Networks	Coexp_Module_brown	5981	RFC1
SZ_Networks	Coexp_Module_brown	4162	MCAM
SZ_Networks	Coexp_Module_brown	7866	IFRD2
SZ_Networks	Coexp_Module_brown	1490	CTGF
SZ_Networks	Coexp_Module_brown	7846	TUBA1A
SZ_Networks	Coexp_Module_brown	5860	QDPR
SZ_Networks	Coexp_Module_brown	25915	NDUFAF3
SZ_Networks	Coexp_Module_brown	79143	MBOAT7
SZ_Networks	Coexp_Module_brown	873	CBR1
SZ_Networks	Coexp_Module_brown	7936	NELFE
SZ_Networks	Coexp_Module_brown	22870	PPP6R1
SZ_Networks	Coexp_Module_brown	56339	METTL3
SZ_Networks	Coexp_Module_brown	2186	BPTF
SZ_Networks	Coexp_Module_brown	760	CA2
SZ_Networks	Coexp_Module_brown	563	AZGP1
SZ_Networks	Coexp_Module_brown	5822	PWP2
SZ_Networks	Coexp_Module_brown	23309	SIN3B
SZ_Networks	Coexp_Module_brown	57060	PCBP4
SZ_Networks	Coexp_Module_brown	9412	MED21
SZ_Networks	Coexp_Module_brown	7508	XPC
SZ_Networks	Coexp_Module_brown	10057	ABCC5
SZ_Networks	Coexp_Module_brown	10522	DEAF1
SZ_Networks	Coexp_Module_brown	8704	B4GALT2

SZ_Networks	Coexp_Module_brown	9044	BTAF1
SZ_Networks	Coexp_Module_brown	9181	ARHGEF2
SZ_Networks	Coexp_Module_brown	10553	HTATIP2
SZ_Networks	Coexp_Module_brown	333	APLP1
SZ_Networks	Coexp_Module_brown	2823	GPM6A
SZ_Networks	Coexp_Module_brown	25936	NSL1
SZ_Networks	Coexp_Module_brown	1798	DPAGT1
SZ_Networks	Coexp_Module_brown	11236	RNF139
SZ_Networks	Coexp_Module_brown	6873	TAF2
SZ_Networks	Coexp_Module_brown	8612	PPAP2C
SZ_Networks	Coexp_Module_brown	11145	PLA2G16
SZ_Networks	Coexp_Module_brown	1501	CTNND2
SZ_Networks	Coexp_Module_brown	2861	GPR37
SZ_Networks	Coexp_Module_brown	5523	PPP2R3A
SZ_Networks	Coexp_Module_brown	6002	RGS12
SZ_Networks	Coexp_Module_brown	5584	PRKCI
SZ_Networks	Coexp_Module_brown	868	CBLB
SZ_Networks	Coexp_Module_brown	10026	PIGK
SZ_Networks	Coexp_Module_brown	7541	ZBTB14
SZ_Networks	Coexp_Module_brown	770	CA11
SZ_Networks	Coexp_Module_brown	4613	MYCN
SZ_Networks	Coexp_Module_brown	1632	ECI1
SZ_Networks	Coexp_Module_brown	1628	DBP
SZ_Networks	Coexp_Module_brown	8605	PLA2G4C
SZ_Networks	Coexp_Module_brown	6663	SOX10
SZ_Networks	Coexp_Module_brown	6623	SNCG
SZ_Networks	Coexp_Module_brown	23127	COLGALT2
SZ_Networks	Coexp_Module_brown	22827	PUF60
SZ_Networks	Coexp_Module_brown	729991	MEF2BNB
SZ_Networks	Coexp_Module_brown	5588	PRKCQ
SZ_Networks	Coexp_Module_brown	23008	KLHDC10
SZ_Networks	Coexp_Module_brown	7108	TM7SF2
SZ_Networks	Coexp_Module_brown	5376	PMP22
SZ_Networks	Coexp_Module_brown	9717	SEC14L5
SZ_Networks	Coexp_Module_brown	2280	FKBP1A
SZ_Networks	Coexp_Module_brown	5354	PLP1
SZ_Networks	Coexp_Module_brown	274	BIN1
SZ_Networks	Coexp_Module_brown	9228	DLGAP2
SZ_Networks	Coexp_Module_brown	9536	PTGES
SZ_Networks	Coexp_Module_brown	6619	SNAPC3
SZ_Networks	Coexp_Module_brown	26118	WSB1
SZ_Networks	Coexp_Module_brown	8724	SNX3
SZ_Networks	Coexp_Module_brown	5414	SEPT4
SZ_Networks	Coexp_Module_brown	63941	NECAB3
SZ_Networks	Coexp_Module_brown	26056	RAB11FIP5
SZ_Networks	Coexp_Module_brown	825	CAPN3
SZ_Networks	Coexp_Module_brown	11343	MGLL
SZ_Networks	Coexp_Module_brown	7461	CLIP2
SZ_Networks	Coexp_Module_brown	8646	CHRD
SZ_Networks	Coexp_Module_brown	6579	SLCO1A2
SZ_Networks	Coexp_Module_brown	3306	HSPA2
SZ_Networks	Coexp_Module_brown	775	CACNA1C
SZ_Networks	Coexp_Module_brown	64963	MRPS11
SZ_Networks	Coexp_Module_brown	1781	DYNC1I2
SZ_Networks	Coexp_Module_brown	3516	RBPJ
SZ_Networks	Coexp_Module_brown	3021	H3F3B
SZ_Networks	Coexp_Module_brown	5411	PNN
SZ_Networks	Coexp_Module_brown	23350	U2SURP

SZ_Networks	Coexp_Module_brown	26133	TRPC4AP
SZ_Networks	Coexp_Module_brown	23203	PMPCA
SZ_Networks	Coexp_Module_brown	2907	GRINA
SZ_Networks	Coexp_Module_brown	57509	MTUS1
SZ_Networks	Coexp_Module_brown	857	CAV1
SZ_Networks	Coexp_Module_brown	90861	HN1L
SZ_Networks	Coexp_Module_brown	23187	PHLDB1
SZ_Networks	Coexp_Module_brown	25777	SUN2
SZ_Networks	Coexp_Module_brown	31	ACACA
SZ_Networks	Coexp_Module_brown	25963	TMEM87A
SZ_Networks	Coexp_Module_brown	23198	PSME4
SZ_Networks	Coexp_Module_brown	3423	IDS
SZ_Networks	Coexp_Module_brown	23165	NUP205
SZ_Networks	Coexp_Module_brown	92140	MTDH
SZ_Networks	Coexp_Module_brown	667	DST
SZ_Networks	Coexp_Module_brown	8888	MCM3AP
SZ_Networks	Coexp_Module_brown	23048	FNBP1
SZ_Networks	Coexp_Module_brown	91782	CHMP7
SZ_Networks	Coexp_Module_brown	22998	LIMCH1
SZ_Networks	Coexp_Module_brown	4642	MYO1D
SZ_Networks	Coexp_Module_brown	10972	TMED10
SZ_Networks	Coexp_Module_brown	5962	RDX
SZ_Networks	Coexp_Module_brown	399665	FAM102A
SZ_Networks	Coexp_Module_brown	200734	SPRED2
SZ_Networks	Coexp_Module_brown	171546	SPTSSA
SZ_Networks	Coexp_Module_brown	8301	PICALM
SZ_Networks	Coexp_Module_brown	23396	PIP5K1C
SZ_Networks	Coexp_Module_brown	7324	UBE2E1
SZ_Networks	Coexp_Module_brown	5151	PDE8A
SZ_Networks	Coexp_Module_brown	23348	DOCK9
SZ_Networks	Coexp_Module_brown	9557	CHD1L
SZ_Networks	Coexp_Module_brown	55023	PHIP
SZ_Networks	Coexp_Module_brown	285527	FRYL
SZ_Networks	Coexp_Module_brown	23258	DENND5A
SZ_Networks	Coexp_Module_brown	23052	ENDOD1
SZ_Networks	Coexp_Module_brown	831	CAST
SZ_Networks	Coexp_Module_brown	10042	HMGXB4
SZ_Networks	Coexp_Module_brown	201562	PTPLB
SZ_Networks	Coexp_Module_brown	7551	ZNF3
SZ_Networks	Coexp_Module_brown	5096	PCCB
SZ_Networks	Coexp_Module_brown	151011	SEPT10
SZ_Networks	Coexp_Module_brown	57553	MICAL3
SZ_Networks	Coexp_Module_brown	23239	PHLPP1
SZ_Networks	Coexp_Module_brown	23336	SYNM
SZ_Networks	Coexp_Module_brown	2760	GM2A
SZ_Networks	Coexp_Module_brown	57591	MKL1
SZ_Networks	Coexp_Module_brown	23332	CLASP1
SZ_Networks	Coexp_Module_brown	23041	MON2
SZ_Networks	Coexp_Module_brown	20	ABCA2
SZ_Networks	Coexp_Module_brown	23241	PACS2
SZ_Networks	Coexp_Module_brown	11113	CIT
SZ_Networks	Coexp_Module_brown	256987	SERINC5
SZ_Networks	Coexp_Module_brown	83700	JAM3
SZ_Networks	Coexp_Module_brown	26030	PLEKHG3
SZ_Networks	Coexp_Module_brown	22976	PAXIP1
SZ_Networks	Coexp_Module_brown	8871	SYNJ2
SZ_Networks	Coexp_Module_brown	8495	PPFIBP2
SZ_Networks	Coexp_Module_brown	84909	C9orf3

SZ_Networks	Coexp_Module_brown	124222	PAQR4
SZ_Networks	Coexp_Module_brown	26009	ZZZ3
SZ_Networks	Coexp_Module_brown	23097	CDK19
SZ_Networks	Coexp_Module_brown	23283	CSTF2T
SZ_Networks	Coexp_Module_brown	137886	UBXN2B
SZ_Networks	Coexp_Module_brown	23452	ANGPTL2
SZ_Networks	Coexp_Module_brown	5362	PLXNA2
SZ_Networks	Coexp_Module_brown	23387	SIK3
SZ_Networks	Coexp_Module_brown	23150	FRMD4B
SZ_Networks	Coexp_Module_brown	123803	NTAN1
SZ_Networks	Coexp_Module_brown	23503	ZFYVE26
SZ_Networks	Coexp_Module_brown	23373	CRTC1
SZ_Networks	Coexp_Module_brown	815	CAMK2A
SZ_Networks	Coexp_Module_brown	1287	COL4A5
SZ_Networks	Coexp_Module_brown	4752	NEK3
SZ_Networks	Coexp_Module_brown	653784	MZT2A
SZ_Networks	Coexp_Module_brown	23113	CUL9
SZ_Networks	Coexp_Module_brown	30850	CDR2L
SZ_Networks	Coexp_Module_brown	22941	SHANK2
SZ_Networks	Coexp_Module_brown	57150	SMIM8
SZ_Networks	Coexp_Module_brown	57212	TP73-AS1
SZ_Networks	Coexp_Module_brown	1028	CDKN1C
SZ_Networks	Coexp_Module_brown	6642	SNX1
SZ_Networks	Coexp_Module_brown	11155	LDB3
SZ_Networks	Coexp_Module_brown	1124	CHN2
SZ_Networks	Coexp_Module_brown	23114	NFASC
SZ_Networks	Coexp_Module_brown	23398	PPWD1
SZ_Networks	Coexp_Module_brown	10147	SUGP2
SZ_Networks	Coexp_Module_brown	402055	SRRD
SZ_Networks	Coexp_Module_brown	151230	KLHL23
SZ_Networks	Coexp_Module_brown	116984	ARAP2
SZ_Networks	Coexp_Module_brown	1298	COL9A2
SZ_Networks	Coexp_Module_brown	23148	NACAD
SZ_Networks	Coexp_Module_brown	10645	CAMKK2
SZ_Networks	Coexp_Module_brown	10215	OLIG2
SZ_Networks	Coexp_Module_brown	440073	IQSEC3
SZ_Networks	Coexp_Module_brown	79789	CLMN
SZ_Networks	Coexp_Module_brown	260294	NSUN5P2
SZ_Networks	Coexp_Module_brown	79912	PYROXD1
SZ_Networks	Coexp_Module_brown	8549	LGR5
SZ_Networks	Coexp_Module_brown	29893	PSMC3IP
SZ_Networks	Coexp_Module_brown	26038	CHD5
SZ_Networks	Coexp_Module_brown	57188	ADAMTSL3
SZ_Networks	Coexp_Module_brown	23359	FAM189A1
SZ_Networks	Coexp_Module_brown	81550	TDRD3
SZ_Networks	Coexp_Module_brown	5789	PTPRD
SZ_Networks	Coexp_Module_brown	8880	FUBP1
SZ_Networks	Coexp_Module_brown	25924	MYRIP
SZ_Networks	Coexp_Module_brown	8632	DNAH17
SZ_Networks	Coexp_Module_brown	30	ACAA1
SZ_Networks	Coexp_Module_brown	65110	UPF3A
SZ_Networks	Coexp_Module_brown	9782	MATR3
SZ_Networks	Coexp_Module_brown	6293	VPS52
SZ_Networks	Coexp_Module_brown	2897	GRIK1
SZ_Networks	Coexp_Module_brown	4340	MOG
SZ_Networks	Coexp_Module_brown	7586	ZKSCAN1
SZ_Networks	Coexp_Module_brown	226	ALDOA
SZ_Networks	Coexp_Module_brown	57798	GATAD1

SZ_Networks	Coexp_Module_brown	23007	PLCH1
SZ_Networks	Coexp_Module_brown	27352	SGSM3
SZ_Networks	Coexp_Module_brown	4650	MYO9B
SZ_Networks	Coexp_Module_brown	23109	DDN
SZ_Networks	Coexp_Module_brown	5129	CDK18
SZ_Networks	Coexp_Module_brown	23096	IQSEC2
SZ_Networks	Coexp_Module_brown	9725	TMEM63A
SZ_Networks	Coexp_Module_brown	9380	GRHPR
SZ_Networks	Coexp_Module_brown	102	ADAM10
SZ_Networks	Coexp_Module_brown	80028	FBXL18
SZ_Networks	Coexp_Module_brown	2971	GTF3A
SZ_Networks	Coexp_Module_brown	4808	NHLH2
SZ_Networks	Coexp_Module_brown	6543	SLC8A2
SZ_Networks	Coexp_Module_brown	643314	KIAA0754
SZ_Networks	Coexp_Module_brown	2346	FOLH1
SZ_Networks	Coexp_Module_brown	2558	GABRA5
SZ_Networks	Coexp_Module_brown	4946	OAZ1
SZ_Networks	Coexp_Module_brown	4099	MAG
SZ_Networks	Coexp_Module_brown	85452	KIAA1751
SZ_Networks	Coexp_Module_brown	23313	KIAA0930
SZ_Networks	Coexp_Module_brown	166	AES
SZ_Networks	Coexp_Module_brown	10015	PDCD6IP
SZ_Networks	Coexp_Module_brown	55748	CNDP2
SZ_Networks	Coexp_Module_brown	4697	NDUFA4
SZ_Networks	Coexp_Module_brown	64397	ZNF106
SZ_Networks	Coexp_Module_brown	51506	UFC1
SZ_Networks	Coexp_Module_brown	79109	MAPKAP1
SZ_Networks	Coexp_Module_brown	51125	GOLGA7
SZ_Networks	Coexp_Module_brown	79811	SLTM
SZ_Networks	Coexp_Module_brown	51466	EVL
SZ_Networks	Coexp_Module_brown	5859	QARS
SZ_Networks	Coexp_Module_brown	55819	RNF130
SZ_Networks	Coexp_Module_brown	51144	HSD17B12
SZ_Networks	Coexp_Module_brown	60313	GPBP1L1
SZ_Networks	Coexp_Module_brown	51133	KCTD3
SZ_Networks	Coexp_Module_brown	23621	BACE1
SZ_Networks	Coexp_Module_brown	27018	NGFRAP1
SZ_Networks	Coexp_Module_brown	29115	SAP30BP
SZ_Networks	Coexp_Module_brown	2287	FKBP3
SZ_Networks	Coexp_Module_brown	8566	PDXK
SZ_Networks	Coexp_Module_brown	51660	MPC1
SZ_Networks	Coexp_Module_brown	51024	FIS1
SZ_Networks	Coexp_Module_brown	4201	MEA1
SZ_Networks	Coexp_Module_brown	25980	AAR2
SZ_Networks	Coexp_Module_brown	55852	TEX2
SZ_Networks	Coexp_Module_brown	56882	CDC42SE1
SZ_Networks	Coexp_Module_brown	4702	NDUFA8
SZ_Networks	Coexp_Module_brown	29796	UQCR10
SZ_Networks	Coexp_Module_brown	4708	NDUFB2
SZ_Networks	Coexp_Module_brown	29880	ALG5
SZ_Networks	Coexp_Module_brown	80148	PQLC1
SZ_Networks	Coexp_Module_brown	746	TMEM258
SZ_Networks	Coexp_Module_brown	4710	NDUFB4
SZ_Networks	Coexp_Module_brown	9950	GOLGA5
SZ_Networks	Coexp_Module_brown	51128	SAR1B
SZ_Networks	Coexp_Module_brown	53371	NUP54
SZ_Networks	Coexp_Module_brown	58486	ZBED5
SZ_Networks	Coexp_Module_brown	64786	TBC1D15

SZ_Networks	Coexp_Module_brown	51642	MRPL48
SZ_Networks	Coexp_Module_brown	60492	CCDC90B
SZ_Networks	Coexp_Module_brown	55450	CAMK2N1
SZ_Networks	Coexp_Module_brown	51809	GALNT7
SZ_Networks	Coexp_Module_brown	26520	TIMM9
SZ_Networks	Coexp_Module_brown	9209	LRRFIP2
SZ_Networks	Coexp_Module_brown	64766	S100PBP
SZ_Networks	Coexp_Module_brown	10179	RBM7
SZ_Networks	Coexp_Module_brown	29937	NENF
SZ_Networks	Coexp_Module_brown	54820	NDE1
SZ_Networks	Coexp_Module_brown	51542	VPS54
SZ_Networks	Coexp_Module_brown	26585	GREM1
SZ_Networks	Coexp_Module_brown	51067	YARS2
SZ_Networks	Coexp_Module_brown	8462	KLF11
SZ_Networks	Coexp_Module_brown	79726	WDR59
SZ_Networks	Coexp_Module_brown	94104	PAXBP1
SZ_Networks	Coexp_Module_brown	55032	SLC35A5
SZ_Networks	Coexp_Module_brown	55665	URGCP
SZ_Networks	Coexp_Module_brown	23400	ATP13A2
SZ_Networks	Coexp_Module_brown	55313	CPPED1
SZ_Networks	Coexp_Module_brown	64784	CRTC3
SZ_Networks	Coexp_Module_brown	55323	LARP6
SZ_Networks	Coexp_Module_brown	54872	PIGG
SZ_Networks	Coexp_Module_brown	55144	LRRC8D
SZ_Networks	Coexp_Module_brown	23555	TSPAN15
SZ_Networks	Coexp_Module_brown	51074	APIP
SZ_Networks	Coexp_Module_brown	51110	LACTB2
SZ_Networks	Coexp_Module_brown	64856	VWA1
SZ_Networks	Coexp_Module_brown	51444	RNF138
SZ_Networks	Coexp_Module_brown	79707	NOL9
SZ_Networks	Coexp_Module_brown	57122	NUP107
SZ_Networks	Coexp_Module_brown	57763	ANKRA2
SZ_Networks	Coexp_Module_brown	54494	C11orf71
SZ_Networks	Coexp_Module_brown	55227	LRRC1
SZ_Networks	Coexp_Module_brown	51121	RPL26L1
SZ_Networks	Coexp_Module_brown	54972	TMEM132A
SZ_Networks	Coexp_Module_brown	56940	DUSP22
SZ_Networks	Coexp_Module_brown	64798	DEPTOR
SZ_Networks	Coexp_Module_brown	51673	TPPP3
SZ_Networks	Coexp_Module_brown	64837	KLC2
SZ_Networks	Coexp_Module_brown	4771	NF2
SZ_Networks	Coexp_Module_brown	54925	ZSCAN32
SZ_Networks	Coexp_Module_brown	80227	PAAF1
SZ_Networks	Coexp_Module_brown	51076	CUTC
SZ_Networks	Coexp_Module_brown	55084	SOBP
SZ_Networks	Coexp_Module_brown	29844	TFPT
SZ_Networks	Coexp_Module_brown	79668	PARP8
SZ_Networks	Coexp_Module_brown	79710	MORC4
SZ_Networks	Coexp_Module_brown	55258	THNSL2
SZ_Networks	Coexp_Module_brown	8270	LAGE3
SZ_Networks	Coexp_Module_brown	79731	NARS2
SZ_Networks	Coexp_Module_brown	28232	SLCO3A1
SZ_Networks	Coexp_Module_brown	55619	DOCK10
SZ_Networks	Coexp_Module_brown	11201	POLI
SZ_Networks	Coexp_Module_brown	56475	RPRM
SZ_Networks	Coexp_Module_brown	79899	PRR5L
SZ_Networks	Coexp_Module_brown	79152	FA2H
SZ_Networks	Coexp_Module_brown	56913	C1GALT1

SZ_Networks	Coexp_Module_brown	54826	GIN1
SZ_Networks	Coexp_Module_brown	56980	PRDM10
SZ_Networks	Coexp_Module_brown	25837	RAB26
SZ_Networks	Coexp_Module_brown	79686	LINC00341
SZ_Networks	Coexp_Module_brown	55614	KIF16B
SZ_Networks	Coexp_Module_brown	4815	NINJ2
SZ_Networks	Coexp_Module_brown	79970	ZNF767
SZ_Networks	Coexp_Module_brown	254394	MCM9
SZ_Networks	Coexp_Module_brown	9499	MYOT
SZ_Networks	Coexp_Module_brown	64130	LIN7B
SZ_Networks	Coexp_Module_brown	28957	MRPS28
SZ_Networks	Coexp_Module_brown	79800	CARF
SZ_Networks	Coexp_Module_brown	26468	LHX6
SZ_Networks	Coexp_Module_brown	55096	EBLN2
SZ_Networks	Coexp_Module_brown	11226	GALNT6
SZ_Networks	Coexp_Module_brown	79905	TMC7
SZ_Networks	Coexp_Module_brown	22802	CLCA4
SZ_Networks	Coexp_Module_brown	80235	PIGZ
SZ_Networks	Coexp_Module_brown	79722	ANKRD55
SZ_Networks	Coexp_Module_brown	60484	HAPLN2
SZ_Networks	Coexp_Module_brown	51700	CYB5R2
SZ_Networks	Coexp_Module_brown	54537	FAM35A
SZ_Networks	Coexp_Module_brown	57084	SLC17A6
SZ_Networks	Coexp_Module_brown	22933	SIRT2
SZ_Networks	Coexp_Module_brown	55205	ZNF532
SZ_Networks	Coexp_Module_brown	55179	FAIM
SZ_Networks	Coexp_Module_brown	105	ADARB2
SZ_Networks	Coexp_Module_brown	11141	IL1RAPL1
SZ_Networks	Coexp_Module_brown	10826	FAXDC2
SZ_Networks	Coexp_Module_brown	57596	BEGAIN
SZ_Networks	Coexp_Module_brown	56936	CCDC177
SZ_Networks	Coexp_Module_brown	26355	FAM162A
SZ_Networks	Coexp_Module_brown	81873	ARPC5L
SZ_Networks	Coexp_Module_brown	81609	SNX27
SZ_Networks	Coexp_Module_brown	26012	NSMF
SZ_Networks	Coexp_Module_brown	51742	ARID4B
SZ_Networks	Coexp_Module_brown	81563	C1orf21
SZ_Networks	Coexp_Module_brown	6138	RPL15
SZ_Networks	Coexp_Module_brown	64422	ATG3
SZ_Networks	Coexp_Module_brown	27335	EIF3K
SZ_Networks	Coexp_Module_brown	8675	STX16
SZ_Networks	Coexp_Module_brown	79365	BHLHE41
SZ_Networks	Coexp_Module_brown	83638	C11orf68
SZ_Networks	Coexp_Module_brown	79003	MIS12
SZ_Networks	Coexp_Module_brown	11221	DUSP10
SZ_Networks	Coexp_Module_brown	79929	MAP6D1
SZ_Networks	Coexp_Module_brown	54434	SSH1
SZ_Networks	Coexp_Module_brown	26119	LDLRAP1
SZ_Networks	Coexp_Module_brown	9367	RAB9A
SZ_Networks	Coexp_Module_brown	54934	KANSL2
SZ_Networks	Coexp_Module_brown	220972	MARCH8
SZ_Networks	Coexp_Module_brown	9314	KLF4
SZ_Networks	Coexp_Module_brown	23232	TBC1D12
SZ_Networks	Coexp_Module_brown	25861	DFNB31
SZ_Networks	Coexp_Module_brown	158747	MOSPD2
SZ_Networks	Coexp_Module_brown	10443	N4BP2L2
SZ_Networks	Coexp_Module_brown	84900	RNFT2
SZ_Networks	Coexp_Module_brown	51218	GLRX5

SZ_Networks	Coexp_Module_brown	5165	PDK3
SZ_Networks	Coexp_Module_brown	55357	TBC1D2
SZ_Networks	Coexp_Module_brown	29956	CERS2
SZ_Networks	Coexp_Module_brown	79007	DBNDD1
SZ_Networks	Coexp_Module_brown	55454	CSGALNACT2
SZ_Networks	Coexp_Module_brown	23677	SH3BP4
SZ_Networks	Coexp_Module_brown	23585	TMEM50A
SZ_Networks	Coexp_Module_brown	23603	CORO1C
SZ_Networks	Coexp_Module_brown	27131	SNX5
SZ_Networks	Coexp_Module_brown	51114	ZDHHC9
SZ_Networks	Coexp_Module_brown	79892	MCMBP
SZ_Networks	Coexp_Module_brown	9547	CXCL14
SZ_Networks	Coexp_Module_brown	50861	STMN3
SZ_Networks	Coexp_Module_brown	23413	NCS1
SZ_Networks	Coexp_Module_brown	84146	ZNF644
SZ_Networks	Coexp_Module_brown	79956	ERMP1
SZ_Networks	Coexp_Module_brown	54443	ANLN
SZ_Networks	Coexp_Module_brown	54801	HAUS6
SZ_Networks	Coexp_Module_brown	55331	ACER3
SZ_Networks	Coexp_Module_brown	55151	TMEM38B
SZ_Networks	Coexp_Module_brown	64792	RABL5
SZ_Networks	Coexp_Module_brown	79644	SRD5A3
SZ_Networks	Coexp_Module_brown	55248	TMEM206
SZ_Networks	Coexp_Module_brown	56896	DPYSL5
SZ_Networks	Coexp_Module_brown	56952	PRTFDC1
SZ_Networks	Coexp_Module_brown	9462	RASAL2
SZ_Networks	Coexp_Module_brown	57690	TNRC6C
SZ_Networks	Coexp_Module_brown	27345	KCNMB4
SZ_Networks	Coexp_Module_brown	28982	FLVCR1
SZ_Networks	Coexp_Module_brown	10888	GPR83
SZ_Networks	Coexp_Module_brown	55004	LAMTOR1
SZ_Networks	Coexp_Module_brown	51248	PDZD11
SZ_Networks	Coexp_Module_brown	54461	FBXW5
SZ_Networks	Coexp_Module_brown	83641	FAM107B
SZ_Networks	Coexp_Module_brown	83543	AIF1L
SZ_Networks	Coexp_Module_brown	54888	NSUN2
SZ_Networks	Coexp_Module_brown	55862	ECHDC1
SZ_Networks	Coexp_Module_brown	25962	KIAA1429
SZ_Networks	Coexp_Module_brown	58480	RHOU
SZ_Networks	Coexp_Module_brown	26022	TMEM98
SZ_Networks	Coexp_Module_brown	221294	NT5DC1
SZ_Networks	Coexp_Module_brown	83719	YPEL3
SZ_Networks	Coexp_Module_brown	63027	SLC22A23
SZ_Networks	Coexp_Module_brown	55829	VIMP
SZ_Networks	Coexp_Module_brown	84287	ZDHHC16
SZ_Networks	Coexp_Module_brown	55967	NDUFA12
SZ_Networks	Coexp_Module_brown	55975	KLHL7
SZ_Networks	Coexp_Module_brown	84640	USP38
SZ_Networks	Coexp_Module_brown	84233	TMEM126A
SZ_Networks	Coexp_Module_brown	666	BOK
SZ_Networks	Coexp_Module_brown	55964	SEPT3
SZ_Networks	Coexp_Module_brown	84261	FBXW9
SZ_Networks	Coexp_Module_brown	93183	PIGM
SZ_Networks	Coexp_Module_brown	83690	CRISPLD1
SZ_Networks	Coexp_Module_brown	66000	TMEM108
SZ_Networks	Coexp_Module_brown	57595	PDZD4
SZ_Networks	Coexp_Module_brown	54880	BCOR
SZ_Networks	Coexp_Module_brown	57467	HHATL

SZ_Networks	Coexp_Module_brown	84288	EFCAB2
SZ_Networks	Coexp_Module_brown	84735	CNDP1
SZ_Networks	Coexp_Module_brown	10655	DMRT2
SZ_Networks	Coexp_Module_brown	79992	AGPAT4-IT1
SZ_Networks	Coexp_Module_brown	94015	TTYH2
SZ_Networks	Coexp_Module_brown	55335	NIPSNAP3B
SZ_Networks	Coexp_Module_brown	3798	KIF5A
SZ_Networks	Coexp_Module_brown	28514	DLL1
SZ_Networks	Coexp_Module_brown	11124	FAF1
SZ_Networks	Coexp_Module_brown	27166	PRELID1
SZ_Networks	Coexp_Module_brown	140803	TRPM6
SZ_Networks	Coexp_Module_brown	84303	CHCHD6
SZ_Networks	Coexp_Module_brown	84315	MON1A
SZ_Networks	Coexp_Module_brown	56893	UBQLN4
SZ_Networks	Coexp_Module_brown	57731	SPTBN4
SZ_Networks	Coexp_Module_brown	50809	HP1BP3
SZ_Networks	Coexp_Module_brown	57448	BIRC6
SZ_Networks	Coexp_Module_brown	121665	SPPL3
SZ_Networks	Coexp_Module_brown	1073	CFL2
SZ_Networks	Coexp_Module_brown	197370	NSMCE1
SZ_Networks	Coexp_Module_brown	114823	LENG8
SZ_Networks	Coexp_Module_brown	23184	MESDC2
SZ_Networks	Coexp_Module_brown	4301	MLLT4
SZ_Networks	Coexp_Module_brown	347734	SLC35B2
SZ_Networks	Coexp_Module_brown	83986	ITFG3
SZ_Networks	Coexp_Module_brown	57584	ARHGAP21
SZ_Networks	Coexp_Module_brown	55677	IWS1
SZ_Networks	Coexp_Module_brown	51148	CERCAM
SZ_Networks	Coexp_Module_brown	26277	TINF2
SZ_Networks	Coexp_Module_brown	88455	ANKRD13A
SZ_Networks	Coexp_Module_brown	149951	COMMD7
SZ_Networks	Coexp_Module_brown	6272	SORT1
SZ_Networks	Coexp_Module_brown	84896	ATAD1
SZ_Networks	Coexp_Module_brown	84188	FAR1
SZ_Networks	Coexp_Module_brown	440574	MINOS1
SZ_Networks	Coexp_Module_brown	57609	DIP2B
SZ_Networks	Coexp_Module_brown	51082	POLR1D
SZ_Networks	Coexp_Module_brown	64951	MRPS24
SZ_Networks	Coexp_Module_brown	950	SCARB2
SZ_Networks	Coexp_Module_brown	121260	SLC15A4
SZ_Networks	Coexp_Module_brown	56927	GPR108
SZ_Networks	Coexp_Module_brown	9517	SPTLC2
SZ_Networks	Coexp_Module_brown	5814	PURB
SZ_Networks	Coexp_Module_brown	221184	CPNE2
SZ_Networks	Coexp_Module_brown	26224	FBXL3
SZ_Networks	Coexp_Module_brown	6242	RTKN
SZ_Networks	Coexp_Module_brown	152503	SH3D19
SZ_Networks	Coexp_Module_brown	64645	HIAT1
SZ_Networks	Coexp_Module_brown	128338	DRAM2
SZ_Networks	Coexp_Module_brown	55766	H2AFJ
SZ_Networks	Coexp_Module_brown	22834	ZNF652
SZ_Networks	Coexp_Module_brown	10085	EDIL3
SZ_Networks	Coexp_Module_brown	127829	ARL8A
SZ_Networks	Coexp_Module_brown	54492	NEURL1B
SZ_Networks	Coexp_Module_brown	5728	PTEN
SZ_Networks	Coexp_Module_brown	91461	PKDCC
SZ_Networks	Coexp_Module_brown	90135	BTBD6
SZ_Networks	Coexp_Module_brown	51621	KLF13

SZ_Networks	Coexp_Module_brown	84340	GFM2
SZ_Networks	Coexp_Module_brown	116461	TSEN15
SZ_Networks	Coexp_Module_brown	84833	USMG5
SZ_Networks	Coexp_Module_brown	84316	LSMD1
SZ_Networks	Coexp_Module_brown	9117	SEC22C
SZ_Networks	Coexp_Module_brown	85013	TMEM128
SZ_Networks	Coexp_Module_brown	127687	C1orf122
SZ_Networks	Coexp_Module_brown	64773	PCED1A
SZ_Networks	Coexp_Module_brown	4133	MAP2
SZ_Networks	Coexp_Module_brown	641638	SNHG6
SZ_Networks	Coexp_Module_brown	54998	AURKAIP1
SZ_Networks	Coexp_Module_brown	255967	PAN3
SZ_Networks	Coexp_Module_brown	221178	SPATA13
SZ_Networks	Coexp_Module_brown	201965	RWDD4
SZ_Networks	Coexp_Module_brown	57210	SLC45A4
SZ_Networks	Coexp_Module_brown	122060	SLAIN1
SZ_Networks	Coexp_Module_brown	92017	SNX29
SZ_Networks	Coexp_Module_brown	57556	SEMA6A
SZ_Networks	Coexp_Module_brown	84899	TMTC4
SZ_Networks	Coexp_Module_brown	57666	FBRSL1
SZ_Networks	Coexp_Module_brown	90799	CEP95
SZ_Networks	Coexp_Module_brown	57475	PLEKHH1
SZ_Networks	Coexp_Module_brown	4040	LRP6
SZ_Networks	Coexp_Module_brown	221035	REEP3
SZ_Networks	Coexp_Module_brown	91689	SMDT1
SZ_Networks	Coexp_Module_brown	124512	METTL23
SZ_Networks	Coexp_Module_brown	54893	MTMR10
SZ_Networks	Coexp_Module_brown	54464	XRN1
SZ_Networks	Coexp_Module_brown	10814	CPLX2
SZ_Networks	Coexp_Module_brown	128218	TMEM125
SZ_Networks	Coexp_Module_brown	6558	SLC12A2
SZ_Networks	Coexp_Module_brown	7008	TEF
SZ_Networks	Coexp_Module_brown	56655	POLE4
SZ_Networks	Coexp_Module_brown	2342	FNTB
SZ_Networks	Coexp_Module_brown	115286	SLC25A26
SZ_Networks	Coexp_Module_brown	57185	NIPAL3
SZ_Networks	Coexp_Module_brown	80018	NAA25
SZ_Networks	Coexp_Module_brown	128869	PIGU
SZ_Networks	Coexp_Module_brown	84334	APOPT1
SZ_Networks	Coexp_Module_brown	340371	NRBP2
SZ_Networks	Coexp_Module_brown	169200	TMEM64
SZ_Networks	Coexp_Module_brown	2549	GAB1
SZ_Networks	Coexp_Module_brown	55917	CTTNBP2NL
SZ_Networks	Coexp_Module_brown	835	CASP2
SZ_Networks	Coexp_Module_brown	80176	SPSB1
SZ_Networks	Coexp_Module_brown	57466	SCAF4
SZ_Networks	Coexp_Module_brown	64398	MPP5
SZ_Networks	Coexp_Module_brown	81790	RNF170
SZ_Networks	Coexp_Module_brown	50862	RNF141
SZ_Networks	Coexp_Module_brown	54149	C21orf91
SZ_Networks	Coexp_Module_brown	6443	SGCB
SZ_Networks	Coexp_Module_brown	9946	CRYZL1
SZ_Networks	Coexp_Module_brown	51228	GLTP
SZ_Networks	Coexp_Module_brown	2065	ERBB3
SZ_Networks	Coexp_Module_brown	401548	SNX30
SZ_Networks	Coexp_Module_brown	64960	MRPS15
SZ_Networks	Coexp_Module_brown	122622	ADSSL1
SZ_Networks	Coexp_Module_brown	130872	AHSA2

SZ_Networks	Coexp_Module_brown	121053	C12orf45
SZ_Networks	Coexp_Module_brown	3092	HIP1
SZ_Networks	Coexp_Module_brown	51529	ANAPC11
SZ_Networks	Coexp_Module_brown	90522	YIF1B
SZ_Networks	Coexp_Module_brown	84915	FAM222A
SZ_Networks	Coexp_Module_brown	57458	TMCC3
SZ_Networks	Coexp_Module_brown	80031	SEMA6D
SZ_Networks	Coexp_Module_brown	84186	ZCCHC7
SZ_Networks	Coexp_Module_brown	4124	MAN2A1
SZ_Networks	Coexp_Module_brown	388228	SBK1
SZ_Networks	Coexp_Module_brown	1609	DGKQ
SZ_Networks	Coexp_Module_brown	26528	DAZAP1
SZ_Networks	Coexp_Module_brown	5337	PLD1
SZ_Networks	Coexp_Module_brown	57636	ARHGAP23
SZ_Networks	Coexp_Module_brown	3344	FOXN2
SZ_Networks	Coexp_Module_brown	129642	MBOAT2
SZ_Networks	Coexp_Module_brown	5208	PFKFB2
SZ_Networks	Coexp_Module_brown	143684	FAM76B
SZ_Networks	Coexp_Module_brown	91404	SESTD1
SZ_Networks	Coexp_Module_brown	7566	ZNF18
SZ_Networks	Coexp_Module_brown	90990	KIFC2
SZ_Networks	Coexp_Module_brown	283267	LINC00294
SZ_Networks	Coexp_Module_brown	55858	TMEM165
SZ_Networks	Coexp_Module_brown	130502	TTC32
SZ_Networks	Coexp_Module_brown	53944	CSNK1G1
SZ_Networks	Coexp_Module_brown	30812	SOX8
SZ_Networks	Coexp_Module_brown	84502	JPH4
SZ_Networks	Coexp_Module_brown	132864	CPEB2
SZ_Networks	Coexp_Module_brown	128434	VSTM2L
SZ_Networks	Coexp_Module_brown	11102	RPP14
SZ_Networks	Coexp_Module_brown	94274	PPP1R14A
SZ_Networks	Coexp_Module_brown	283635	FAM177A1
SZ_Networks	Coexp_Module_brown	65124	SOWAHC
SZ_Networks	Coexp_Module_brown	25914	RTTN
SZ_Networks	Coexp_Module_brown	57519	STARD9
SZ_Networks	Coexp_Module_brown	643988	C1orf233
SZ_Networks	Coexp_Module_brown	64975	MRPL41
SZ_Networks	Coexp_Module_brown	59271	EVA1C
SZ_Networks	Coexp_Module_brown	6872	TAF1
SZ_Networks	Coexp_Module_brown	338657	CCDC84
SZ_Networks	Coexp_Module_brown	57482	KIAA1211
SZ_Networks	Coexp_Module_brown	134359	POC5
SZ_Networks	Coexp_Module_brown	388962	BOLA3
SZ_Networks	Coexp_Module_brown	1954	MEGF8
SZ_Networks	Coexp_Module_brown	140685	ZBTB46
SZ_Networks	Coexp_Module_brown	159195	USP54
SZ_Networks	Coexp_Module_brown	4784	NFIX
SZ_Networks	Coexp_Module_brown	286343	LURAP1L
SZ_Networks	Coexp_Module_brown	221545	C6orf136
SZ_Networks	Coexp_Module_brown	23383	MAU2
SZ_Networks	Coexp_Module_brown	154043	CNKSR3
SZ_Networks	Coexp_Module_brown	51104	ABHD17B
SZ_Networks	Coexp_Module_brown	84749	USP30
SZ_Networks	Coexp_Module_brown	55051	NRDE2
SZ_Networks	Coexp_Module_brown	123283	TARSL2
SZ_Networks	Coexp_Module_brown	146330	FBXL16
SZ_Networks	Coexp_Module_brown	140730	RIMS4
SZ_Networks	Coexp_Module_brown	91419	XRCC6BP1

SZ_Networks	Coexp_Module_brown	219844	HYLS1
SZ_Networks	Coexp_Module_brown	2770	GNAI1
SZ_Networks	Coexp_Module_brown	389337	ARHGEF37
SZ_Networks	Coexp_Module_brown	90390	MED30
SZ_Networks	Coexp_Module_brown	643836	ZFP62
SZ_Networks	Coexp_Module_brown	132720	C4orf32
SZ_Networks	Coexp_Module_brown	85358	SHANK3
SZ_Networks	Coexp_Module_brown	83786	FRMD8
SZ_Networks	Coexp_Module_brown	79183	TTPAL
SZ_Networks	Coexp_Module_brown	91683	SYT12
SZ_Networks	Coexp_Module_brown	143903	LAYN
SZ_Networks	Coexp_Module_brown	29116	MYLIP
SZ_Networks	Coexp_Module_brown	91584	PLXNA4
SZ_Networks	Coexp_Module_brown	2067	ERCC1
SZ_Networks	Coexp_Module_brown	339263	C17orf51
SZ_Networks	Coexp_Module_brown	196500	PIANP
SZ_Networks	Coexp_Module_brown	115548	FCHO2
SZ_Networks	Coexp_Module_brown	147906	DACT3
SZ_Networks	Coexp_Module_brown	54620	FBXL19
SZ_Networks	Coexp_Module_brown	55857	PLK1S1
SZ_Networks	Coexp_Module_brown	91614	DEPDC7
SZ_Networks	Coexp_Module_brown	285464	CRIPAK
SZ_Networks	Coexp_Module_brown	92558	CCDC64
SZ_Networks	Coexp_Module_brown	1600	DAB1
SZ_Networks	Coexp_Module_brown	5010	CLDN11
SZ_Networks	Coexp_Module_brown	114825	PWWP2A
SZ_Networks	Coexp_Module_brown	118812	MORN4
SZ_Networks	Coexp_Module_brown	170371	C10orf128
SZ_Networks	Coexp_Module_brown	152404	IGSF11
SZ_Networks	Coexp_Module_brown	84327	ZBED3
SZ_Networks	Coexp_Module_brown	3749	KCNC4
SZ_Networks	Coexp_Module_brown	344558	SH3RF3
SZ_Networks	Coexp_Module_brown	23446	SLC44A1
SZ_Networks	Coexp_Module_brown	55356	SLC22A15
SZ_Networks	Coexp_Module_brown	91133	L3MBTL4
SZ_Networks	Coexp_Module_brown	401022	HOXD-AS1
SZ_Networks	Coexp_Module_brown	10096	ACTR3
SZ_Networks	Coexp_Module_brown	55314	TMEM144
SZ_Networks	Coexp_Module_brown	5629	PROX1
SZ_Networks	Coexp_Module_brown	10505	SEMA4F
SZ_Networks	Coexp_Module_brown	56895	AGPAT4
SZ_Networks	Coexp_Module_brown	85414	SLC45A3
SZ_Networks	Coexp_Module_brown	113510	HELQ
SZ_Networks	Coexp_Module_brown	374875	HSD11B1L
SZ_Networks	Coexp_Module_brown	143879	KBTBD3
SZ_Networks	Coexp_Module_brown	23528	ZNF281
SZ_Networks	Coexp_Module_brown	10507	SEMA4D
SZ_Networks	Coexp_Module_brown	80312	TET1
SZ_Networks	Coexp_Module_brown	84307	ZNF397
SZ_Networks	Coexp_Module_brown	144811	LACC1
SZ_Networks	Coexp_Module_brown	117584	RFFL
SZ_Networks	Coexp_Module_brown	57571	CARNS1
SZ_Networks	Coexp_Module_brown	90673	PPP1R3E
SZ_Networks	Coexp_Module_brown	441094	NR2F1-AS1
SZ_Networks	Coexp_Module_brown	84190	METTL25
SZ_Networks	Coexp_Module_brown	57558	USP35
SZ_Networks	Coexp_Module_brown	400242	DICER1-AS1
SZ_Networks	Coexp_Module_brown	22874	PLEKHA6

SZ_Networks	Coexp_Module_brown	170463	SSBP4
SZ_Networks	Coexp_Module_brown	57338	JPH3
SZ_Networks	Coexp_Module_brown	89801	PPP1R3F
SZ_Networks	Coexp_Module_brown	2909	ARHGAP35
SZ_Networks	Coexp_Module_brown	148418	SAMD13
SZ_Networks	Coexp_Module_brown	79939	SLC35E1
SZ_Networks	Coexp_Module_brown	138639	PTPDC1
SZ_Networks	Coexp_Module_brown	2562	GABRB3
SZ_Networks	Coexp_Module_brown	57507	ZNF608
SZ_Networks	Coexp_Module_brown	55095	SAMD4B
SZ_Networks	Coexp_Module_brown	118738	ZNF488
SZ_Networks	Coexp_Module_brown	133121	ENPP6
SZ_Networks	Coexp_Module_brown	388662	SLC6A17
SZ_Networks	Coexp_Module_brown	2055	CLN8
SZ_Networks	Coexp_Module_brown	768206	PRCD
SZ_Networks	Coexp_Module_brown	84545	MRPL43
SZ_Networks	Coexp_Module_brown	64172	OSGEPL1
SZ_Networks	Coexp_Module_brown	170692	ADAMTS18
SZ_Networks	Coexp_Module_brown	6900	CNTN2
SZ_Networks	Coexp_Module_brown	51735	RAPGEF6
SZ_Networks	Coexp_Module_brown	145270	PRIMA1
SZ_Networks	Coexp_Module_brown	389084	C2orf82
SZ_Networks	Coexp_Module_brown	80005	DOCK5
SZ_Networks	Coexp_Module_brown	342035	GLDN
SZ_Networks	Coexp_Module_brown	55471	NDUFAF7
SZ_Networks	Coexp_Module_brown	53637	S1PR5
SZ_Networks	Coexp_Module_brown	388135	C15orf59
SZ_Networks	Coexp_Module_brown	387914	SHISA2
SZ_Networks	Coexp_Module_brown	220108	FAM124A
SZ_Networks	Coexp_Module_brown	94032	CAMK2N2
SZ_Networks	Coexp_Module_brown	23250	ATP11A
SZ_Networks	Coexp_Module_brown	10361	NPM2
SZ_Networks	Coexp_Module_brown	117283	IP6K3
SZ_Networks	Coexp_Module_brown	64147	KIF9
SZ_Networks	Coexp_Module_brown	11046	SLC35D2
SZ_Networks	Coexp_Module_brown	23544	SEZ6L
SZ_Networks	Coexp_Module_brown	127281	FAM213B
SZ_Networks	Coexp_Module_brown	51068	NMD3
SZ_Networks	Coexp_Module_brown	347689	SOX2-OT
SZ_Networks	Coexp_Module_brown	57471	ERMN
SZ_Networks	Coexp_Module_brown	220164	DOK6
SZ_Networks	Coexp_Module_brown	439949	PRKCQ-AS1
SZ_Networks	Coexp_Module_brown	85445	CNTNAP4
SZ_Networks	Coexp_Module_brown	340351	AGBL3
SZ_Networks	Coexp_Module_brown	57622	LRFN1
SZ_Networks	Coexp_Module_brown	55968	NSFL1C
SZ_Networks	Coexp_Module_brown	158038	LINGO2
SZ_Networks	Coexp_Module_brown	284615	ANKRD34A
SZ_Networks	Coexp_Module_brown	26297	SERGEF
SZ_Networks	Coexp_Module_brown	282973	JAKMIP3
SZ_Networks	Coexp_Module_brown	9328	GTF3C5
SZ_Networks	Coexp_Module_brown	55593	OTUD5
SZ_Networks	Coexp_Module_brown	160	AP2A1
SZ_Networks	Coexp_Module_brown	55902	ACSS2
SZ_Networks	Coexp_Module_brown	54832	VPS13C
SZ_Networks	Coexp_Module_brown	161742	SPRED1
SZ_Networks	Coexp_Module_brown	201725	C4orf46
SZ_Networks	Coexp_Module_brown	222223	KIAA1324L

SZ_Networks	Coexp_Module_brown	339983	NAT8L
SZ_Networks	Coexp_Module_brown	50859	SPOCK3
SZ_Networks	Coexp_Module_brown	27031	NPHP3
SZ_Networks	Coexp_Module_brown	219287	AMER2
SZ_Networks	Coexp_Module_brown	286827	TRIM59
SZ_Networks	Coexp_Module_brown	344595	LINC00883
SZ_Networks	Coexp_Module_brown	160335	TMTC2
SZ_Networks	Coexp_Module_brown	119392	SFR1
SZ_Networks	Coexp_Module_brown	84530	SRRM4
SZ_Networks	Coexp_Module_brown	121601	ANO4
SZ_Networks	Coexp_Module_brown	3996	LLGL1
SZ_Networks	Coexp_Module_brown	115584	SLC5A11
SZ_Networks	Coexp_Module_brown	114786	XKR4
SZ_Networks	Coexp_Module_brown	285800	PRR18
SZ_Networks	Coexp_Module_brown	643911	CRNDE
SZ_Networks	Coexp_Module_brown	203190	LGI3
SZ_Networks	Coexp_Module_brown	338328	GPIHBP1
SZ_Networks	Coexp_Module_brown	116362	RBP7
SZ_Networks	Coexp_Module_brown	8110	DPF3
SZ_Networks	Coexp_Module_brown	284323	ZNF780A
SZ_Networks	Coexp_Module_brown	5137	PDE1C
SZ_Networks	Coexp_Module_brown	134549	SHROOM1
SZ_Networks	Coexp_Module_brown	84083	ZRANB3
SZ_Networks	Coexp_Module_brown	93377	OPALIN
SZ_Networks	Coexp_Module_brown	254251	LCORL
SZ_Networks	Coexp_Module_brown	100505929	C21orf37
SZ_Networks	Coexp_Module_brown	154215	NKAIN2
SZ_Networks	Coexp_Module_brown	1745	DLX1
SZ_Networks	Coexp_Module_brown	151647	FAM19A4
SZ_Networks	Coexp_Module_brown	777	CACNA1E
SZ_Networks	Coexp_Module_brown	145788	C15orf65
SZ_Networks	Coexp_Module_brown	345630	FBLL1
SZ_Networks	Coexp_Module_brown	253650	ANKRD18A
SZ_Networks	Coexp_Module_brown	1378	CR1
SZ_Networks	Coexp_Module_brown	133022	TRAM1L1
SZ_Networks	Coexp_Module_brown	100128252	ZNF667-AS1
SZ_Networks	Coexp_Module_brown	10634	GAS2L1
SZ_Networks	Coexp_Module_brown	23625	FAM89B
SZ_Networks	Coexp_Module_brown	6253	RTN2
SZ_Networks	Coexp_Module_brown	203069	R3HCC1
SZ_Networks	Coexp_Module_brown	9905	SGSM2
SZ_Networks	Coexp_Module_brown	4681	NBL1
SZ_Networks	Coexp_Module_brown	933	CD22
SZ_Networks	Coexp_Module_brown	6305	SBF1
SZ_Networks	Coexp_Module_brown	26051	PPP1R16B
SZ_Networks	Coexp_Module_brown	150726	FBXO41
SZ_Networks	Coexp_Module_brown	57406	ABHD6
SZ_Networks	Coexp_Module_brown	54985	HCFC1R1
SZ_Networks	Coexp_Module_brown	55101	ATP5SL
SZ_Networks	Coexp_Module_brown	54910	SEMA4C
SZ_Networks	Coexp_Module_brown	93643	TJAP1
SZ_Networks	Coexp_Module_brown	112869	CCDC101
SZ_Networks	Coexp_Module_brown	7942	TFEB
SZ_Networks	Coexp_Module_brown	26088	GGA1
SZ_Networks	Coexp_Module_brown	196743	PAOX
SZ_Networks	Coexp_Module_brown	89853	MVB12B
SZ_Networks	Coexp_Module_brown	79701	OGFOD3
SZ_Networks	Coexp_Module_brown	83637	ZMIZ2

SZ_Networks	Coexp_Module_brown	85377	MICALL1
SZ_Networks	Coexp_Module_brown	64834	ELOVL1
SZ_Networks	Coexp_Module_brown	55223	TRIM62
SZ_Networks	Coexp_Module_brown	5526	PPP2R5B
SZ_Networks	Coexp_Module_brown	57175	CORO1B
SZ_Networks	Coexp_Module_brown	79583	TMEM231
SZ_Networks	Coexp_Module_brown	79874	RABEP2
SZ_Networks	Coexp_Module_darkorange	7494	XBP1
SZ_Networks	Coexp_Module_darkorange	813	CALU
SZ_Networks	Coexp_Module_darkorange	8531	YBX3
SZ_Networks	Coexp_Module_darkorange	9775	EIF4A3
SZ_Networks	Coexp_Module_darkorange	7538	ZFP36
SZ_Networks	Coexp_Module_darkorange	4860	PNP
SZ_Networks	Coexp_Module_darkorange	3615	IMPDH2
SZ_Networks	Coexp_Module_darkorange	2171	FABP5
SZ_Networks	Coexp_Module_darkorange	4147	MATN2
SZ_Networks	Coexp_Module_darkorange	5209	PFKFB3
SZ_Networks	Coexp_Module_darkorange	22918	CD93
SZ_Networks	Coexp_Module_darkorange	604	BCL6
SZ_Networks	Coexp_Module_darkorange	1647	GADD45A
SZ_Networks	Coexp_Module_darkorange	8460	TPST1
SZ_Networks	Coexp_Module_darkorange	1017	CDK2
SZ_Networks	Coexp_Module_darkorange	2152	F3
SZ_Networks	Coexp_Module_darkorange	2669	GEM
SZ_Networks	Coexp_Module_darkorange	5277	PIGA
SZ_Networks	Coexp_Module_darkorange	5966	REL
SZ_Networks	Coexp_Module_darkorange	5606	MAP2K3
SZ_Networks	Coexp_Module_darkorange	11167	FSTL1
SZ_Networks	Coexp_Module_darkorange	60481	ELOVL5
SZ_Networks	Coexp_Module_darkorange	1407	CRY1
SZ_Networks	Coexp_Module_darkorange	3082	HGF
SZ_Networks	Coexp_Module_darkorange	4013	VWA5A
SZ_Networks	Coexp_Module_darkorange	4928	NUP98
SZ_Networks	Coexp_Module_darkorange	26018	LRIG1
SZ_Networks	Coexp_Module_darkorange	1973	EIF4A1
SZ_Networks	Coexp_Module_darkorange	23516	SLC39A14
SZ_Networks	Coexp_Module_darkorange	3987	LIMS1
SZ_Networks	Coexp_Module_darkorange	1992	SERPINB1
SZ_Networks	Coexp_Module_darkorange	6432	SRSF7
SZ_Networks	Coexp_Module_darkorange	6464	SHC1
SZ_Networks	Coexp_Module_darkorange	3995	FADS3
SZ_Networks	Coexp_Module_darkorange	339479	FAM5C
SZ_Networks	Coexp_Module_darkorange	10135	NAMPT
SZ_Networks	Coexp_Module_darkorange	79042	TSEN34
SZ_Networks	Coexp_Module_darkorange	6322	SCML1
SZ_Networks	Coexp_Module_darkorange	51129	ANGPTL4
SZ_Networks	Coexp_Module_darkorange	54915	YTHDF1
SZ_Networks	Coexp_Module_darkorange	25825	BACE2
SZ_Networks	Coexp_Module_darkorange	10783	NEK6
SZ_Networks	Coexp_Module_darkorange	64332	NFKBIZ
SZ_Networks	Coexp_Module_darkorange	200916	RPL22L1
SZ_Networks	Coexp_Module_darkorange	284207	METRNL
SZ_Networks	Coexp_Module_darkorange	56975	FAM20C
SZ_Networks	Coexp_Module_darkorange	195827	AAED1
SZ_Networks	Coexp_Module_darkorange	51050	PI15
SZ_Networks	Coexp_Module_darkorange	283131	NEAT1
SZ_Networks	Coexp_Module_lightcyan	377	ARF3
SZ_Networks	Coexp_Module_lightcyan	8933	FAM127A

SZ_Networks	Coexp_Module_lightcyan	5590	PRKCZ
SZ_Networks	Coexp_Module_lightcyan	3064	HTT
SZ_Networks	Coexp_Module_lightcyan	1400	CRMP1
SZ_Networks	Coexp_Module_lightcyan	140885	SIRPA
SZ_Networks	Coexp_Module_lightcyan	9915	ARNT2
SZ_Networks	Coexp_Module_lightcyan	5799	PTPRN2
SZ_Networks	Coexp_Module_lightcyan	23229	ARHGEF9
SZ_Networks	Coexp_Module_lightcyan	8514	KCNAB2
SZ_Networks	Coexp_Module_lightcyan	9797	TATDN2
SZ_Networks	Coexp_Module_lightcyan	4900	NRGN
SZ_Networks	Coexp_Module_lightcyan	1020	CDK5
SZ_Networks	Coexp_Module_lightcyan	23641	LDOC1
SZ_Networks	Coexp_Module_lightcyan	2039	DMTN
SZ_Networks	Coexp_Module_lightcyan	9764	KIAA0513
SZ_Networks	Coexp_Module_lightcyan	8927	BSN
SZ_Networks	Coexp_Module_lightcyan	5218	CDK14
SZ_Networks	Coexp_Module_lightcyan	9796	PHYHIP
SZ_Networks	Coexp_Module_lightcyan	3785	KCNQ2
SZ_Networks	Coexp_Module_lightcyan	8941	CDK5R2
SZ_Networks	Coexp_Module_lightcyan	3706	ITPKA
SZ_Networks	Coexp_Module_lightcyan	10900	RUNDC3A
SZ_Networks	Coexp_Module_lightcyan	23221	RHOBTB2
SZ_Networks	Coexp_Module_lightcyan	25864	ABHD14A
SZ_Networks	Coexp_Module_lightcyan	53335	BCL11A
SZ_Networks	Coexp_Module_lightcyan	84726	PRRC2B
SZ_Networks	Coexp_Module_lightcyan	2036	EPB41L1
SZ_Networks	Coexp_Module_lightcyan	25999	CLIP3
SZ_Networks	Coexp_Module_lightcyan	4628	MYH10
SZ_Networks	Coexp_Module_lightcyan	9807	IP6K1
SZ_Networks	Coexp_Module_lightcyan	57476	GRAMD1B
SZ_Networks	Coexp_Module_lightcyan	5965	RECQL
SZ_Networks	Coexp_Module_lightcyan	23031	MAST3
SZ_Networks	Coexp_Module_lightcyan	460	ASTN1
SZ_Networks	Coexp_Module_lightcyan	155066	ATP6V0E2
SZ_Networks	Coexp_Module_lightcyan	441151	TMEM151B
SZ_Networks	Coexp_Module_lightcyan	23316	CUX2
SZ_Networks	Coexp_Module_lightcyan	8497	PPFIA4
SZ_Networks	Coexp_Module_lightcyan	1759	DNM1
SZ_Networks	Coexp_Module_lightcyan	64771	C6orf106
SZ_Networks	Coexp_Module_lightcyan	79762	C1orf115
SZ_Networks	Coexp_Module_lightcyan	27344	PCSK1N
SZ_Networks	Coexp_Module_lightcyan	25789	TMEM59L
SZ_Networks	Coexp_Module_lightcyan	79187	FSD1
SZ_Networks	Coexp_Module_lightcyan	8506	CNTNAP1
SZ_Networks	Coexp_Module_lightcyan	1008	CDH10
SZ_Networks	Coexp_Module_lightcyan	5361	PLXNA1
SZ_Networks	Coexp_Module_lightcyan	6853	SYN1
SZ_Networks	Coexp_Module_lightcyan	54472	TOLLIP
SZ_Networks	Coexp_Module_lightcyan	80700	UBXN6
SZ_Networks	Coexp_Module_lightcyan	10815	CPLX1
SZ_Networks	Coexp_Module_lightcyan	64101	LRRC4
SZ_Networks	Coexp_Module_lightcyan	84258	SYT3
SZ_Networks	Coexp_Module_lightcyan	400916	CHCHD10
SZ_Networks	Coexp_Module_lightcyan	115024	NT5C3B
SZ_Networks	Coexp_Module_lightcyan	55844	PPP2R2D
SZ_Networks	Coexp_Module_lightcyan	100287932	TIMM23
SZ_Networks	Coexp_Module_lightcyan	4254	KITLG
SZ_Networks	Coexp_Module_lightcyan	57463	AMIGO1

SZ_Networks	Coexp_Module_lightcyan	285362	SUMF1
SZ_Networks	Coexp_Module_lightcyan	57504	MTA3
SZ_Networks	Coexp_Module_lightcyan	387921	NHLRC3
SZ_Networks	Coexp_Module_lightcyan	85300	ATCAY
SZ_Networks	Coexp_Module_lightcyan	126282	TNFAIP8L1
SZ_Networks	Coexp_Module_lightcyan	23025	UNC13A
SZ_Networks	Coexp_Module_lightcyan	387923	SERP2
SZ_Networks	Coexp_Module_lightcyan	3778	KCNMA1
SZ_Networks	Coexp_Module_lightcyan	1804	DPP6
SZ_Networks	Coexp_Module_lightcyan	7275	TUB
SZ_Networks	Coexp_Module_lightcyan	1740	DLG2
SZ_Networks	Coexp_Module_lightcyan	146713	RBFOX3
SZ_Networks	Coexp_Module_lightcyan	60680	CELF5
SZ_Networks	Coexp_Module_lightcyan	112755	STX1B
SZ_Networks	Coexp_Module_lightcyan	57689	LRRC4C
SZ_Networks	Coexp_Module_lightcyan	11346	SYNPO
SZ_Networks	Coexp_Module_lightcyan	784	CACNB3
SZ_Networks	Coexp_Module_lightcyan	55611	OTUB1
SZ_Networks	Coexp_Module_lightcyan	376267	RAB15
SZ_Networks	Coexp_Module_midnightblue	6547	SLC8A3
SZ_Networks	Coexp_Module_midnightblue	4478	MSN
SZ_Networks	Coexp_Module_midnightblue	8407	TAGLN2
SZ_Networks	Coexp_Module_midnightblue	79073	TMEM109
SZ_Networks	Coexp_Module_midnightblue	678	ZFP36L2
SZ_Networks	Coexp_Module_midnightblue	3487	IGFBP4
SZ_Networks	Coexp_Module_midnightblue	26509	MYOF
SZ_Networks	Coexp_Module_midnightblue	3597	IL13RA1
SZ_Networks	Coexp_Module_midnightblue	25937	WWTR1
SZ_Networks	Coexp_Module_midnightblue	3985	LIMK2
SZ_Networks	Coexp_Module_midnightblue	3554	IL1R1
SZ_Networks	Coexp_Module_midnightblue	22856	CHSY1
SZ_Networks	Coexp_Module_midnightblue	11309	SLCO2B1
SZ_Networks	Coexp_Module_midnightblue	9411	ARHGAP29
SZ_Networks	Coexp_Module_midnightblue	1052	CEBPD
SZ_Networks	Coexp_Module_midnightblue	571	BACH1
SZ_Networks	Coexp_Module_midnightblue	4005	LMO2
SZ_Networks	Coexp_Module_midnightblue	3087	HHEX
SZ_Networks	Coexp_Module_midnightblue	1439	CSF2RB
SZ_Networks	Coexp_Module_midnightblue	3570	IL6R
SZ_Networks	Coexp_Module_midnightblue	5627	PROS1
SZ_Networks	Coexp_Module_midnightblue	4904	YBX1
SZ_Networks	Coexp_Module_midnightblue	9124	PDLIM1
SZ_Networks	Coexp_Module_midnightblue	7037	TFRC
SZ_Networks	Coexp_Module_midnightblue	7048	TGFBR2
SZ_Networks	Coexp_Module_midnightblue	5175	PECAM1
SZ_Networks	Coexp_Module_midnightblue	6774	STAT3
SZ_Networks	Coexp_Module_midnightblue	182	JAG1
SZ_Networks	Coexp_Module_midnightblue	11067	C10orf10
SZ_Networks	Coexp_Module_midnightblue	84617	TUBB6
SZ_Networks	Coexp_Module_midnightblue	8795	TNFRSF10B
SZ_Networks	Coexp_Module_midnightblue	4071	TM4SF1
SZ_Networks	Coexp_Module_midnightblue	5873	RAB27A
SZ_Networks	Coexp_Module_midnightblue	30844	EHD4
SZ_Networks	Coexp_Module_midnightblue	3588	IL10RB
SZ_Networks	Coexp_Module_midnightblue	302	ANXA2
SZ_Networks	Coexp_Module_midnightblue	3119	HLA-DQB1
SZ_Networks	Coexp_Module_midnightblue	4627	MYH9
SZ_Networks	Coexp_Module_midnightblue	1284	COL4A2

SZ_Networks	Coexp_Module_midnightblue	1282	COL4A1
SZ_Networks	Coexp_Module_midnightblue	115207	KCTD12
SZ_Networks	Coexp_Module_midnightblue	8829	NRP1
SZ_Networks	Coexp_Module_midnightblue	23034	SAMD4A
SZ_Networks	Coexp_Module_midnightblue	221749	PXDC1
SZ_Networks	Coexp_Module_midnightblue	22898	DENND3
SZ_Networks	Coexp_Module_midnightblue	5771	PTPN2
SZ_Networks	Coexp_Module_midnightblue	7035	TFPI
SZ_Networks	Coexp_Module_midnightblue	9413	FAM189A2
SZ_Networks	Coexp_Module_midnightblue	2335	FN1
SZ_Networks	Coexp_Module_midnightblue	54918	CMTM6
SZ_Networks	Coexp_Module_midnightblue	116496	FAM129A
SZ_Networks	Coexp_Module_midnightblue	53827	FXYD5
SZ_Networks	Coexp_Module_midnightblue	55332	DRAM1
SZ_Networks	Coexp_Module_midnightblue	51279	C1RL
SZ_Networks	Coexp_Module_midnightblue	79651	RHBDF2
SZ_Networks	Coexp_Module_midnightblue	51365	PLA1A
SZ_Networks	Coexp_Module_midnightblue	9630	GNA14
SZ_Networks	Coexp_Module_midnightblue	83716	CRISPLD2
SZ_Networks	Coexp_Module_midnightblue	2004	ELK3
SZ_Networks	Coexp_Module_midnightblue	55616	ASAP3
SZ_Networks	Coexp_Module_midnightblue	58191	CXCL16
SZ_Networks	Coexp_Module_midnightblue	51435	SCARA3
SZ_Networks	Coexp_Module_midnightblue	23406	COTL1
SZ_Networks	Coexp_Module_midnightblue	7046	TGFBR1
SZ_Networks	Coexp_Module_midnightblue	196527	ANO6
SZ_Networks	Coexp_Module_midnightblue	152137	CCDC50
SZ_Networks	Coexp_Module_midnightblue	2120	ETV6
SZ_Networks	Coexp_Module_midnightblue	91107	TRIM47
SZ_Networks	Coexp_Module_midnightblue	768211	RELL1
SZ_Networks	Coexp_Module_midnightblue	83937	RASSF4
SZ_Networks	Coexp_Module_midnightblue	79993	ELOVL7
SZ_Networks	Coexp_Module_midnightblue	121457	IKBIP
SZ_Networks	Coexp_Module_midnightblue	3718	JAK3
SZ_Networks	Coexp_Module_midnightblue	54947	LPCAT2
SZ_Networks	Coexp_Module_midnightblue	140576	S100A16
SZ_Networks	Coexp_Module_midnightblue	155038	GIMAP8
Synapse_PPI_Communities	Syn-7_0_39	1915	EEF1A1
Synapse_PPI_Communities	Syn-7_0_39	6128	RPL6
Synapse_PPI_Communities	Syn-7_0_39	6124	RPL4
Synapse_PPI_Communities	Syn-7_0_39	1938	EEF2
Synapse_PPI_Communities	Syn-7_0_39	6217	RPS16
Synapse_PPI_Communities	Syn-7_0_39	55832	CAND1
Synapse_PPI_Communities	Syn-7_0_39	140801	RPL10L
Synapse_PPI_Communities	Syn-7_0_39	6189	RPS3A
Synapse_PPI_Communities	Syn-7_0_39	7534	YWHAZ
Synapse_PPI_Communities	Syn-7_0_39	4738	NEDD8
Synapse_PPI_Communities	Syn-7_0_39	6152	RPL24
Synapse_PPI_Communities	Syn-7_0_39	6137	RPL13
Synapse_PPI_Communities	Syn-7_0_39	6205	RPS11
Synapse_PPI_Communities	Syn-7_0_39	6138	RPL15
Synapse_PPI_Communities	Syn-7_0_39	6193	RPS5
Synapse_PPI_Communities	Syn-7_0_39	6134	RPL10
Synapse_PPI_Communities	Syn-7_0_39	6132	RPL8
Synapse_PPI_Communities	Syn-7_0_39	6223	RPS19
Synapse_PPI_Communities	Syn-7_0_39	6130	RPL7A
Synapse_PPI_Communities	Syn-7_0_39	6135	RPL11
Synapse_PPI_Communities	Syn-7_0_39	6129	RPL7

Synapse_PPI_Communities	Syn-7_0_39	6224	RPS20
Synapse_PPI_Communities	Syn-7_0_39	6136	RPL12
Synapse_PPI_Communities	Syn-7_0_39	6141	RPL18
Synapse_PPI_Communities	Syn-7_0_39	6160	RPL31
Synapse_PPI_Communities	Syn-7_0_39	6144	RPL21
Synapse_PPI_Communities	Syn-7_0_39	6187	RPS2
Synapse_PPI_Communities	Syn-7_0_39	6228	RPS23
Synapse_PPI_Communities	Syn-7_0_39	4736	RPL10A
Synapse_PPI_Communities	Syn-7_0_39	6191	RPS4X
Synapse_PPI_Communities	Syn-7_0_39	6202	RPS8
Synapse_PPI_Communities	Syn-7_0_39	6194	RPS6
Synapse_PPI_Communities	Syn-7_0_39	6147	RPL23A
Synapse_PPI_Communities	Syn-7_0_39	6125	RPL5
Synapse_PPI_Communities	Syn-7_0_39	6188	RPS3
Synapse_PPI_Communities	Syn-7_0_39	6613	SUMO2
Synapse_PPI_Communities	Syn-7_0_39	7341	SUMO1
Synapse_PPI_Communities	Syn-7_0_39	10987	COPS5
Synapse_PPI_Communities	Syn-7_0_39	2335	FN1
Synapse_PPI_Communities	Syn-6_1_9	7534	YWHAZ
Synapse_PPI_Communities	Syn-6_1_9	7532	YWHAG
Synapse_PPI_Communities	Syn-6_1_9	7529	YWHAB
Synapse_PPI_Communities	Syn-6_1_9	7533	YWHAH
Synapse_PPI_Communities	Syn-6_1_9	9181	ARHGEF2
Synapse_PPI_Communities	Syn-6_1_9	7415	VCP
Synapse_PPI_Communities	Syn-6_1_9	2885	GRB2
Synapse_PPI_Communities	Syn-6_1_9	8452	CUL3
Synapse_PPI_Communities	Syn-6_1_9	2335	FN1
Synapse_PPI_Communities	Syn-6_0_85	1915	EEF1A1
Synapse_PPI_Communities	Syn-6_0_85	6128	RPL6
Synapse_PPI_Communities	Syn-6_0_85	6124	RPL4
Synapse_PPI_Communities	Syn-6_0_85	1938	EEF2
Synapse_PPI_Communities	Syn-6_0_85	6217	RPS16
Synapse_PPI_Communities	Syn-6_0_85	55832	CAND1
Synapse_PPI_Communities	Syn-6_0_85	140801	RPL10L
Synapse_PPI_Communities	Syn-6_0_85	6189	RPS3A
Synapse_PPI_Communities	Syn-6_0_85	7534	YWHAZ
Synapse_PPI_Communities	Syn-6_0_85	4738	NEDD8
Synapse_PPI_Communities	Syn-6_0_85	6152	RPL24
Synapse_PPI_Communities	Syn-6_0_85	6137	RPL13
Synapse_PPI_Communities	Syn-6_0_85	6205	RPS11
Synapse_PPI_Communities	Syn-6_0_85	6138	RPL15
Synapse_PPI_Communities	Syn-6_0_85	6193	RPS5
Synapse_PPI_Communities	Syn-6_0_85	6134	RPL10
Synapse_PPI_Communities	Syn-6_0_85	6132	RPL8
Synapse_PPI_Communities	Syn-6_0_85	6223	RPS19
Synapse_PPI_Communities	Syn-6_0_85	6130	RPL7A
Synapse_PPI_Communities	Syn-6_0_85	6135	RPL11
Synapse_PPI_Communities	Syn-6_0_85	6129	RPL7
Synapse_PPI_Communities	Syn-6_0_85	6224	RPS20
Synapse_PPI_Communities	Syn-6_0_85	6136	RPL12
Synapse_PPI_Communities	Syn-6_0_85	6141	RPL18
Synapse_PPI_Communities	Syn-6_0_85	6160	RPL31
Synapse_PPI_Communities	Syn-6_0_85	6144	RPL21
Synapse_PPI_Communities	Syn-6_0_85	6187	RPS2
Synapse_PPI_Communities	Syn-6_0_85	6228	RPS23
Synapse_PPI_Communities	Syn-6_0_85	4736	RPL10A
Synapse_PPI_Communities	Syn-6_0_85	6191	RPS4X
Synapse_PPI_Communities	Syn-6_0_85	6202	RPS8

Synapse_PPI_Communities	Syn-6_0_85	6194	RPS6
Synapse_PPI_Communities	Syn-6_0_85	6147	RPL23A
Synapse_PPI_Communities	Syn-6_0_85	6125	RPL5
Synapse_PPI_Communities	Syn-6_0_85	6188	RPS3
Synapse_PPI_Communities	Syn-6_0_85	6613	SUMO2
Synapse_PPI_Communities	Syn-6_0_85	7341	SUMO1
Synapse_PPI_Communities	Syn-6_0_85	10987	COPS5
Synapse_PPI_Communities	Syn-6_0_85	2335	FN1
Synapse_PPI_Communities	Syn-6_0_85	6218	RPS17
Synapse_PPI_Communities	Syn-6_0_85	6203	RPS9
Synapse_PPI_Communities	Syn-6_0_85	93185	IGSF8
Synapse_PPI_Communities	Syn-6_0_85	6155	RPL27
Synapse_PPI_Communities	Syn-6_0_85	6122	RPL3
Synapse_PPI_Communities	Syn-6_0_85	25873	RPL36
Synapse_PPI_Communities	Syn-6_0_85	6233	RPS27A
Synapse_PPI_Communities	Syn-6_0_85	6168	RPL37A
Synapse_PPI_Communities	Syn-6_0_85	292	SLC25A5
Synapse_PPI_Communities	Syn-6_0_85	6142	RPL18A
Synapse_PPI_Communities	Syn-6_0_85	6234	RPS28
Synapse_PPI_Communities	Syn-6_0_85	9045	RPL14
Synapse_PPI_Communities	Syn-6_0_85	3608	ILF2
Synapse_PPI_Communities	Syn-6_0_85	10528	NOP56
Synapse_PPI_Communities	Syn-6_0_85	6201	RPS7
Synapse_PPI_Communities	Syn-6_0_85	6230	RPS25
Synapse_PPI_Communities	Syn-6_0_85	6146	RPL22
Synapse_PPI_Communities	Syn-6_0_85	4691	NCL
Synapse_PPI_Communities	Syn-6_0_85	6139	RPL17
Synapse_PPI_Communities	Syn-6_0_85	6133	RPL9
Synapse_PPI_Communities	Syn-6_0_85	6159	RPL29
Synapse_PPI_Communities	Syn-6_0_85	6156	RPL30
Synapse_PPI_Communities	Syn-6_0_85	6229	RPS24
Synapse_PPI_Communities	Syn-6_0_85	6207	RPS13
Synapse_PPI_Communities	Syn-6_0_85	9349	RPL23
Synapse_PPI_Communities	Syn-6_0_85	220717	RPLP0P6
Synapse_PPI_Communities	Syn-6_0_85	6143	RPL19
Synapse_PPI_Communities	Syn-6_0_85	6176	RPLP1
Synapse_PPI_Communities	Syn-6_0_85	6175	RPLP0
Synapse_PPI_Communities	Syn-6_0_85	3609	ILF3
Synapse_PPI_Communities	Syn-6_0_85	3320	HSP90AA1
Synapse_PPI_Communities	Syn-6_0_85	6210	RPS15A
Synapse_PPI_Communities	Syn-6_0_85	6045	RNF2
Synapse_PPI_Communities	Syn-6_0_85	26270	FBXO6
Synapse_PPI_Communities	Syn-6_0_85	1017	CDK2
Synapse_PPI_Communities	Syn-6_0_85	8266	UBL4A
Synapse_PPI_Communities	Syn-6_0_85	8452	CUL3
Synapse_PPI_Communities	Syn-6_0_85	8454	CUL1
Synapse_PPI_Communities	Syn-6_0_85	8065	CUL5
Synapse_PPI_Communities	Syn-6_0_85	409	ARRB2
Synapse_PPI_Communities	Syn-6_0_85	23435	TARDBP
Synapse_PPI_Communities	Syn-6_0_85	3921	RPSA
Synapse_PPI_Communities	Syn-6_0_85	6780	STAU1
Synapse_PPI_Communities	Syn-6_0_85	8453	CUL2
Synapse_PPI_Communities	Syn-6_0_85	7316	UBC
Synapse_PPI_Communities	Syn-6_0_85	975	CD81
Synapse_PPI_Communities	Syn-5_9_5	7534	YWHAZ
Synapse_PPI_Communities	Syn-5_9_5	7532	YWHAG
Synapse_PPI_Communities	Syn-5_9_5	3799	KIF5B
Synapse_PPI_Communities	Syn-5_9_5	7529	YWHAB

Synapse_PPI_Communities	Syn-5_9_5	89953	KLC4
Synapse_PPI_Communities	Syn-5_8_9	529	ATP6V1E1
Synapse_PPI_Communities	Syn-5_8_9	525	ATP6V1B1
Synapse_PPI_Communities	Syn-5_8_9	7316	UBC
Synapse_PPI_Communities	Syn-5_8_9	535	ATP6V0A1
Synapse_PPI_Communities	Syn-5_8_9	51382	ATP6V1D
Synapse_PPI_Communities	Syn-5_8_9	526	ATP6V1B2
Synapse_PPI_Communities	Syn-5_8_9	9296	ATP6V1F
Synapse_PPI_Communities	Syn-5_8_9	51606	ATP6V1H
Synapse_PPI_Communities	Syn-5_8_9	64924	SLC30A5
Synapse_PPI_Communities	Syn-5_0_187	1915	EEF1A1
Synapse_PPI_Communities	Syn-5_0_187	6128	RPL6
Synapse_PPI_Communities	Syn-5_0_187	6124	RPL4
Synapse_PPI_Communities	Syn-5_0_187	1938	EEF2
Synapse_PPI_Communities	Syn-5_0_187	6217	RPS16
Synapse_PPI_Communities	Syn-5_0_187	55832	CAND1
Synapse_PPI_Communities	Syn-5_0_187	140801	RPL10L
Synapse_PPI_Communities	Syn-5_0_187	6189	RPS3A
Synapse_PPI_Communities	Syn-5_0_187	7534	YWHAZ
Synapse_PPI_Communities	Syn-5_0_187	4738	NEDD8
Synapse_PPI_Communities	Syn-5_0_187	6152	RPL24
Synapse_PPI_Communities	Syn-5_0_187	6137	RPL13
Synapse_PPI_Communities	Syn-5_0_187	6205	RPS11
Synapse_PPI_Communities	Syn-5_0_187	6138	RPL15
Synapse_PPI_Communities	Syn-5_0_187	6193	RPS5
Synapse_PPI_Communities	Syn-5_0_187	6134	RPL10
Synapse_PPI_Communities	Syn-5_0_187	6132	RPL8
Synapse_PPI_Communities	Syn-5_0_187	6223	RPS19
Synapse_PPI_Communities	Syn-5_0_187	6130	RPL7A
Synapse_PPI_Communities	Syn-5_0_187	6135	RPL11
Synapse_PPI_Communities	Syn-5_0_187	6129	RPL7
Synapse_PPI_Communities	Syn-5_0_187	6224	RPS20
Synapse_PPI_Communities	Syn-5_0_187	6136	RPL12
Synapse_PPI_Communities	Syn-5_0_187	6141	RPL18
Synapse_PPI_Communities	Syn-5_0_187	6160	RPL31
Synapse_PPI_Communities	Syn-5_0_187	6144	RPL21
Synapse_PPI_Communities	Syn-5_0_187	6187	RPS2
Synapse_PPI_Communities	Syn-5_0_187	6228	RPS23
Synapse_PPI_Communities	Syn-5_0_187	4736	RPL10A
Synapse_PPI_Communities	Syn-5_0_187	6191	RPS4X
Synapse_PPI_Communities	Syn-5_0_187	6202	RPS8
Synapse_PPI_Communities	Syn-5_0_187	6194	RPS6
Synapse_PPI_Communities	Syn-5_0_187	6147	RPL23A
Synapse_PPI_Communities	Syn-5_0_187	6125	RPL5
Synapse_PPI_Communities	Syn-5_0_187	6188	RPS3
Synapse_PPI_Communities	Syn-5_0_187	6613	SUMO2
Synapse_PPI_Communities	Syn-5_0_187	7341	SUMO1
Synapse_PPI_Communities	Syn-5_0_187	10987	COPS5
Synapse_PPI_Communities	Syn-5_0_187	2335	FN1
Synapse_PPI_Communities	Syn-5_0_187	6218	RPS17
Synapse_PPI_Communities	Syn-5_0_187	6203	RPS9
Synapse_PPI_Communities	Syn-5_0_187	93185	IGSF8
Synapse_PPI_Communities	Syn-5_0_187	6155	RPL27
Synapse_PPI_Communities	Syn-5_0_187	6122	RPL3
Synapse_PPI_Communities	Syn-5_0_187	25873	RPL36
Synapse_PPI_Communities	Syn-5_0_187	6233	RPS27A
Synapse_PPI_Communities	Syn-5_0_187	6168	RPL37A
Synapse_PPI_Communities	Syn-5_0_187	292	SLC25A5

Synapse_PPI_Communities	Syn-5_0_187	6142	RPL18A
Synapse_PPI_Communities	Syn-5_0_187	7532	YWHAG
Synapse_PPI_Communities	Syn-5_0_187	7529	YWHAB
Synapse_PPI_Communities	Syn-5_0_187	7533	YWHAH
Synapse_PPI_Communities	Syn-5_0_187	9181	ARHGEF2
Synapse_PPI_Communities	Syn-5_0_187	7415	VCP
Synapse_PPI_Communities	Syn-5_0_187	6234	RPS28
Synapse_PPI_Communities	Syn-5_0_187	9045	RPL14
Synapse_PPI_Communities	Syn-5_0_187	3608	ILF2
Synapse_PPI_Communities	Syn-5_0_187	10528	NOP56
Synapse_PPI_Communities	Syn-5_0_187	6201	RPS7
Synapse_PPI_Communities	Syn-5_0_187	6230	RPS25
Synapse_PPI_Communities	Syn-5_0_187	6146	RPL22
Synapse_PPI_Communities	Syn-5_0_187	4691	NCL
Synapse_PPI_Communities	Syn-5_0_187	6139	RPL17
Synapse_PPI_Communities	Syn-5_0_187	6133	RPL9
Synapse_PPI_Communities	Syn-5_0_187	6159	RPL29
Synapse_PPI_Communities	Syn-5_0_187	6156	RPL30
Synapse_PPI_Communities	Syn-5_0_187	6229	RPS24
Synapse_PPI_Communities	Syn-5_0_187	6207	RPS13
Synapse_PPI_Communities	Syn-5_0_187	9349	RPL23
Synapse_PPI_Communities	Syn-5_0_187	220717	RPLP0P6
Synapse_PPI_Communities	Syn-5_0_187	6143	RPL19
Synapse_PPI_Communities	Syn-5_0_187	6176	RPLP1
Synapse_PPI_Communities	Syn-5_0_187	6175	RPLP0
Synapse_PPI_Communities	Syn-5_0_187	3609	ILF3
Synapse_PPI_Communities	Syn-5_0_187	3320	HSP90AA1
Synapse_PPI_Communities	Syn-5_0_187	6210	RPS15A
Synapse_PPI_Communities	Syn-5_0_187	2885	GRB2
Synapse_PPI_Communities	Syn-5_0_187	8452	CUL3
Synapse_PPI_Communities	Syn-5_0_187	6045	RNF2
Synapse_PPI_Communities	Syn-5_0_187	10971	YWHAQ
Synapse_PPI_Communities	Syn-5_0_187	2597	GAPDH
Synapse_PPI_Communities	Syn-5_0_187	203068	TUBB
Synapse_PPI_Communities	Syn-5_0_187	26270	FBXO6
Synapse_PPI_Communities	Syn-5_0_187	1017	CDK2
Synapse_PPI_Communities	Syn-5_0_187	3312	HSPA8
Synapse_PPI_Communities	Syn-5_0_187	8266	UBL4A
Synapse_PPI_Communities	Syn-5_0_187	8454	CUL1
Synapse_PPI_Communities	Syn-5_0_187	8065	CUL5
Synapse_PPI_Communities	Syn-5_0_187	409	ARRB2
Synapse_PPI_Communities	Syn-5_0_187	23435	TARDBP
Synapse_PPI_Communities	Syn-5_0_187	3921	RPSA
Synapse_PPI_Communities	Syn-5_0_187	6780	STAU1
Synapse_PPI_Communities	Syn-5_0_187	8453	CUL2
Synapse_PPI_Communities	Syn-5_0_187	7316	UBC
Synapse_PPI_Communities	Syn-5_0_187	975	CD81
Synapse_PPI_Communities	Syn-5_0_187	6222	RPS18
Synapse_PPI_Communities	Syn-5_0_187	55720	TSR1
Synapse_PPI_Communities	Syn-5_0_187	51065	RPS27L
Synapse_PPI_Communities	Syn-5_0_187	65220	NADK
Synapse_PPI_Communities	Syn-5_0_187	501	ALDH7A1
Synapse_PPI_Communities	Syn-5_0_187	26999	CYFIP2
Synapse_PPI_Communities	Syn-5_0_187	9493	KIF23
Synapse_PPI_Communities	Syn-5_0_187	51602	NOP58
Synapse_PPI_Communities	Syn-5_0_187	11224	RPL35
Synapse_PPI_Communities	Syn-5_0_187	7979	SHFM1
Synapse_PPI_Communities	Syn-5_0_187	10514	MYBBP1A

Synapse_PPI_Communities	Syn-5_0_187	9770	RASSF2
Synapse_PPI_Communities	Syn-5_0_187	7846	TUBA1A
Synapse_PPI_Communities	Syn-5_0_187	27430	MAT2B
Synapse_PPI_Communities	Syn-5_0_187	3646	EIF3E
Synapse_PPI_Communities	Syn-5_0_187	85440	DOCK7
Synapse_PPI_Communities	Syn-5_0_187	27044	SND1
Synapse_PPI_Communities	Syn-5_0_187	5127	CDK16
Synapse_PPI_Communities	Syn-5_0_187	369	ARAF
Synapse_PPI_Communities	Syn-5_0_187	65125	WNK1
Synapse_PPI_Communities	Syn-5_0_187	6232	RPS27
Synapse_PPI_Communities	Syn-5_0_187	9101	USP8
Synapse_PPI_Communities	Syn-5_0_187	8661	EIF3A
Synapse_PPI_Communities	Syn-5_0_187	6208	RPS14
Synapse_PPI_Communities	Syn-5_0_187	23327	NEDD4L
Synapse_PPI_Communities	Syn-5_0_187	6209	RPS15
Synapse_PPI_Communities	Syn-5_0_187	4486	MST1R
Synapse_PPI_Communities	Syn-5_0_187	10133	OPTN
Synapse_PPI_Communities	Syn-5_0_187	10238	DCAF7
Synapse_PPI_Communities	Syn-5_0_187	6157	RPL27A
Synapse_PPI_Communities	Syn-5_0_187	2539	G6PD
Synapse_PPI_Communities	Syn-5_0_187	6927	HNF1A
Synapse_PPI_Communities	Syn-5_0_187	6231	RPS26
Synapse_PPI_Communities	Syn-5_0_187	4670	HNRNPM
Synapse_PPI_Communities	Syn-5_0_187	7531	YWHAE
Synapse_PPI_Communities	Syn-5_0_187	351	APP
Synapse_PPI_Communities	Syn-5_0_187	6227	RPS21
Synapse_PPI_Communities	Syn-5_0_187	5747	PTK2
Synapse_PPI_Communities	Syn-5_0_187	23367	LARP1
Synapse_PPI_Communities	Syn-5_0_187	1029	CDKN2A
Synapse_PPI_Communities	Syn-5_0_187	3303	HSPA1A
Synapse_PPI_Communities	Syn-5_0_187	5062	PAK2
Synapse_PPI_Communities	Syn-5_0_187	4904	YBX1
Synapse_PPI_Communities	Syn-5_0_187	3313	HSPA9
Synapse_PPI_Communities	Syn-5_0_187	5580	PRKCD
Synapse_PPI_Communities	Syn-5_0_187	10014	HDAC5
Synapse_PPI_Communities	Syn-5_0_187	10399	GNB2L1
Synapse_PPI_Communities	Syn-5_0_187	7414	VCL
Synapse_PPI_Communities	Syn-5_0_187	26986	PABPC1
Synapse_PPI_Communities	Syn-5_0_187	3309	HSPA5
Synapse_PPI_Communities	Syn-5_0_187	80086	TUBA4B
Synapse_PPI_Communities	Syn-5_0_187	1173	AP2M1
Synapse_PPI_Communities	Syn-5_0_187	84790	TUBA1C
Synapse_PPI_Communities	Syn-5_0_187	25	ABL1
Synapse_PPI_Communities	Syn-5_0_187	3383	ICAM1
Synapse_PPI_Communities	Syn-5_0_187	2932	GSK3B
Synapse_PPI_Communities	Syn-5_0_187	6622	SNCA
Synapse_PPI_Communities	Syn-5_0_187	5598	MAPK7
Synapse_PPI_Communities	Syn-5_0_187	6711	SPTBN1
Synapse_PPI_Communities	Syn-5_0_187	6709	SPTAN1
Synapse_PPI_Communities	Syn-5_0_187	867	CBL
Synapse_PPI_Communities	Syn-5_0_187	9531	BAG3
Synapse_PPI_Communities	Syn-5_0_187	4627	MYH9
Synapse_PPI_Communities	Syn-5_0_187	84959	UBASH3B
Synapse_PPI_Communities	Syn-5_0_187	9657	IQCB1
Synapse_PPI_Communities	Syn-5_0_187	1994	ELAVL1
Synapse_PPI_Communities	Syn-5_0_187	408	ARRB1
Synapse_PPI_Communities	Syn-5_0_187	2099	ESR1
Synapse_PPI_Communities	Syn-5_0_187	9924	PAN2

Synapse_PPI_Communities	Syn-5_0_187	54165	DCUN1D1
Synapse_PPI_Communities	Syn-5_0_187	1457	CSNK2A1
Synapse_PPI_Communities	Syn-5_0_187	9775	EIF4A3
Synapse_PPI_Communities	Syn-5_0_187	6464	SHC1
Synapse_PPI_Communities	Syn-5_0_187	1386	ATF2
Synapse_PPI_Communities	Syn-5_0_187	10537	UBD
Synapse_PPI_Communities	Syn-5_0_187	5052	PRDX1
Synapse_PPI_Communities	Syn-5_0_187	10576	CCT2
Synapse_PPI_Communities	Syn-5_0_187	5684	PSMA3
Synapse_PPI_Communities	Syn-5_0_187	5518	PPP2R1A
Synapse_PPI_Communities	Syn-5_0_187	4193	MDM2
Synapse_PPI_Communities	Syn-5_0_187	302	ANXA2
Synapse_PPI_Communities	Syn-5_0_187	5071	PARK2
Synapse_PPI_Communities	Syn-5_0_187	7412	VCAM1
Synapse_PPI_Communities	Syn-5_0_187	3326	HSP90AB1
Synapse_PPI_Communities	Syn-5_0_187	51547	SIRT7
Synapse_PPI_Communities	Syn-5_0_187	8450	CUL4B
Synapse_PPI_Communities	Syn-5_0_187	4869	NPM1
Synapse_PPI_Communities	Syn-5_0_187	3676	ITGA4
Synapse_PPI_Communities	Syn-5_0_187	154	ADRB2
Synapse_PPI_Communities	Syn-5_0_187	57154	SMURF1
Synapse_PPI_Communities	Syn-5_0_187	2023	ENO1
Synapse_PPI_Communities	Syn-5_0_187	1956	EGFR
Synapse_PPI_Communities	Syn-4_9_13	4905	NSF
Synapse_PPI_Communities	Syn-4_9_13	8417	STX7
Synapse_PPI_Communities	Syn-4_9_13	10228	STX6
Synapse_PPI_Communities	Syn-4_9_13	7316	UBC
Synapse_PPI_Communities	Syn-4_9_13	8773	SNAP23
Synapse_PPI_Communities	Syn-4_9_13	8775	NAPA
Synapse_PPI_Communities	Syn-4_9_13	6811	STX5
Synapse_PPI_Communities	Syn-4_9_13	6616	SNAP25
Synapse_PPI_Communities	Syn-4_9_13	6810	STX4
Synapse_PPI_Communities	Syn-4_9_13	9342	SNAP29
Synapse_PPI_Communities	Syn-4_9_13	6845	VAMP7
Synapse_PPI_Communities	Syn-4_9_13	23673	STX12
Synapse_PPI_Communities	Syn-4_9_13	4899	NRF1
Synapse_PPI_Communities	Syn-4_32_5	161	AP2A2
Synapse_PPI_Communities	Syn-4_32_5	160	AP2A1
Synapse_PPI_Communities	Syn-4_32_5	273	AMPH
Synapse_PPI_Communities	Syn-4_32_5	274	BIN1
Synapse_PPI_Communities	Syn-4_32_5	7316	UBC
Synapse_PPI_Communities	Syn-4_3_16	529	ATP6V1E1
Synapse_PPI_Communities	Syn-4_3_16	525	ATP6V1B1
Synapse_PPI_Communities	Syn-4_3_16	7316	UBC
Synapse_PPI_Communities	Syn-4_3_16	535	ATP6V0A1
Synapse_PPI_Communities	Syn-4_3_16	51382	ATP6V1D
Synapse_PPI_Communities	Syn-4_3_16	526	ATP6V1B2
Synapse_PPI_Communities	Syn-4_3_16	9296	ATP6V1F
Synapse_PPI_Communities	Syn-4_3_16	51606	ATP6V1H
Synapse_PPI_Communities	Syn-4_3_16	64924	SLC30A5
Synapse_PPI_Communities	Syn-4_3_16	10312	TCIRG1
Synapse_PPI_Communities	Syn-4_3_16	528	ATP6V1C1
Synapse_PPI_Communities	Syn-4_3_16	6222	RPS18
Synapse_PPI_Communities	Syn-4_3_16	6209	RPS15
Synapse_PPI_Communities	Syn-4_3_16	6844	VAMP2
Synapse_PPI_Communities	Syn-4_3_16	1244	ABCC2
Synapse_PPI_Communities	Syn-4_3_16	8301	PICALM
Synapse_PPI_Communities	Syn-4_23_10	51678	MPP6

Synapse_PPI_Communities	Syn-4_23_10	3768	KCNJ12
Synapse_PPI_Communities	Syn-4_23_10	8825	LIN7A
Synapse_PPI_Communities	Syn-4_23_10	7316	UBC
Synapse_PPI_Communities	Syn-4_23_10	1742	DLG4
Synapse_PPI_Communities	Syn-4_23_10	1740	DLG2
Synapse_PPI_Communities	Syn-4_23_10	2977	GUCY1A2
Synapse_PPI_Communities	Syn-4_23_10	140735	DYNLL2
Synapse_PPI_Communities	Syn-4_23_10	8655	DYNLL1
Synapse_PPI_Communities	Syn-4_23_10	4130	MAP1A
Synapse_PPI_Communities	Syn-4_0_509	1915	EEF1A1
Synapse_PPI_Communities	Syn-4_0_509	6128	RPL6
Synapse_PPI_Communities	Syn-4_0_509	6124	RPL4
Synapse_PPI_Communities	Syn-4_0_509	1938	EEF2
Synapse_PPI_Communities	Syn-4_0_509	6217	RPS16
Synapse_PPI_Communities	Syn-4_0_509	55832	CAND1
Synapse_PPI_Communities	Syn-4_0_509	140801	RPL10L
Synapse_PPI_Communities	Syn-4_0_509	6189	RPS3A
Synapse_PPI_Communities	Syn-4_0_509	7534	YWHAZ
Synapse_PPI_Communities	Syn-4_0_509	4738	NEDD8
Synapse_PPI_Communities	Syn-4_0_509	6152	RPL24
Synapse_PPI_Communities	Syn-4_0_509	6137	RPL13
Synapse_PPI_Communities	Syn-4_0_509	6205	RPS11
Synapse_PPI_Communities	Syn-4_0_509	6138	RPL15
Synapse_PPI_Communities	Syn-4_0_509	6193	RPS5
Synapse_PPI_Communities	Syn-4_0_509	6134	RPL10
Synapse_PPI_Communities	Syn-4_0_509	6132	RPL8
Synapse_PPI_Communities	Syn-4_0_509	6223	RPS19
Synapse_PPI_Communities	Syn-4_0_509	6130	RPL7A
Synapse_PPI_Communities	Syn-4_0_509	6135	RPL11
Synapse_PPI_Communities	Syn-4_0_509	6129	RPL7
Synapse_PPI_Communities	Syn-4_0_509	6224	RPS20
Synapse_PPI_Communities	Syn-4_0_509	6136	RPL12
Synapse_PPI_Communities	Syn-4_0_509	6141	RPL18
Synapse_PPI_Communities	Syn-4_0_509	6160	RPL31
Synapse_PPI_Communities	Syn-4_0_509	6144	RPL21
Synapse_PPI_Communities	Syn-4_0_509	6187	RPS2
Synapse_PPI_Communities	Syn-4_0_509	6228	RPS23
Synapse_PPI_Communities	Syn-4_0_509	4736	RPL10A
Synapse_PPI_Communities	Syn-4_0_509	6191	RPS4X
Synapse_PPI_Communities	Syn-4_0_509	6202	RPS8
Synapse_PPI_Communities	Syn-4_0_509	6194	RPS6
Synapse_PPI_Communities	Syn-4_0_509	6147	RPL23A
Synapse_PPI_Communities	Syn-4_0_509	6125	RPL5
Synapse_PPI_Communities	Syn-4_0_509	6188	RPS3
Synapse_PPI_Communities	Syn-4_0_509	6613	SUMO2
Synapse_PPI_Communities	Syn-4_0_509	7341	SUMO1
Synapse_PPI_Communities	Syn-4_0_509	10987	COPS5
Synapse_PPI_Communities	Syn-4_0_509	2335	FN1
Synapse_PPI_Communities	Syn-4_0_509	6218	RPS17
Synapse_PPI_Communities	Syn-4_0_509	6203	RPS9
Synapse_PPI_Communities	Syn-4_0_509	93185	IGSF8
Synapse_PPI_Communities	Syn-4_0_509	6155	RPL27
Synapse_PPI_Communities	Syn-4_0_509	6122	RPL3
Synapse_PPI_Communities	Syn-4_0_509	25873	RPL36
Synapse_PPI_Communities	Syn-4_0_509	6233	RPS27A
Synapse_PPI_Communities	Syn-4_0_509	6168	RPL37A
Synapse_PPI_Communities	Syn-4_0_509	292	SLC25A5
Synapse_PPI_Communities	Syn-4_0_509	6142	RPL18A

Synapse_PPI_Communities	Syn-4_0_509	7532	YWHAG
Synapse_PPI_Communities	Syn-4_0_509	7529	YWHAB
Synapse_PPI_Communities	Syn-4_0_509	7533	YWHAH
Synapse_PPI_Communities	Syn-4_0_509	9181	ARHGEF2
Synapse_PPI_Communities	Syn-4_0_509	7415	VCP
Synapse_PPI_Communities	Syn-4_0_509	6234	RPS28
Synapse_PPI_Communities	Syn-4_0_509	9045	RPL14
Synapse_PPI_Communities	Syn-4_0_509	3608	ILF2
Synapse_PPI_Communities	Syn-4_0_509	10528	NOP56
Synapse_PPI_Communities	Syn-4_0_509	6201	RPS7
Synapse_PPI_Communities	Syn-4_0_509	6230	RPS25
Synapse_PPI_Communities	Syn-4_0_509	6146	RPL22
Synapse_PPI_Communities	Syn-4_0_509	4691	NCL
Synapse_PPI_Communities	Syn-4_0_509	6139	RPL17
Synapse_PPI_Communities	Syn-4_0_509	6133	RPL9
Synapse_PPI_Communities	Syn-4_0_509	6159	RPL29
Synapse_PPI_Communities	Syn-4_0_509	6156	RPL30
Synapse_PPI_Communities	Syn-4_0_509	6229	RPS24
Synapse_PPI_Communities	Syn-4_0_509	6207	RPS13
Synapse_PPI_Communities	Syn-4_0_509	9349	RPL23
Synapse_PPI_Communities	Syn-4_0_509	220717	RPLP0P6
Synapse_PPI_Communities	Syn-4_0_509	6143	RPL19
Synapse_PPI_Communities	Syn-4_0_509	7203	CCT3
Synapse_PPI_Communities	Syn-4_0_509	10574	CCT7
Synapse_PPI_Communities	Syn-4_0_509	5516	PPP2CB
Synapse_PPI_Communities	Syn-4_0_509	10694	CCT8
Synapse_PPI_Communities	Syn-4_0_509	10014	HDAC5
Synapse_PPI_Communities	Syn-4_0_509	10575	CCT4
Synapse_PPI_Communities	Syn-4_0_509	6950	TCP1
Synapse_PPI_Communities	Syn-4_0_509	3676	ITGA4
Synapse_PPI_Communities	Syn-4_0_509	6176	RPLP1
Synapse_PPI_Communities	Syn-4_0_509	6175	RPLP0
Synapse_PPI_Communities	Syn-4_0_509	3609	ILF3
Synapse_PPI_Communities	Syn-4_0_509	3320	HSP90AA1
Synapse_PPI_Communities	Syn-4_0_509	6210	RPS15A
Synapse_PPI_Communities	Syn-4_0_509	2885	GRB2
Synapse_PPI_Communities	Syn-4_0_509	8452	CUL3
Synapse_PPI_Communities	Syn-4_0_509	6045	RNF2
Synapse_PPI_Communities	Syn-4_0_509	10971	YWHAQ
Synapse_PPI_Communities	Syn-4_0_509	2597	GAPDH
Synapse_PPI_Communities	Syn-4_0_509	203068	TUBB
Synapse_PPI_Communities	Syn-4_0_509	26270	FBXO6
Synapse_PPI_Communities	Syn-4_0_509	1017	CDK2
Synapse_PPI_Communities	Syn-4_0_509	3312	HSPA8
Synapse_PPI_Communities	Syn-4_0_509	8266	UBL4A
Synapse_PPI_Communities	Syn-4_0_509	8454	CUL1
Synapse_PPI_Communities	Syn-4_0_509	8065	CUL5
Synapse_PPI_Communities	Syn-4_0_509	409	ARRB2
Synapse_PPI_Communities	Syn-4_0_509	23435	TARDBP
Synapse_PPI_Communities	Syn-4_0_509	3921	RPSA
Synapse_PPI_Communities	Syn-4_0_509	6780	STAU1
Synapse_PPI_Communities	Syn-4_0_509	8453	CUL2
Synapse_PPI_Communities	Syn-4_0_509	7316	UBC
Synapse_PPI_Communities	Syn-4_0_509	975	CD81
Synapse_PPI_Communities	Syn-4_0_509	6222	RPS18
Synapse_PPI_Communities	Syn-4_0_509	55720	TSR1
Synapse_PPI_Communities	Syn-4_0_509	51065	RPS27L
Synapse_PPI_Communities	Syn-4_0_509	65220	NADK

Synapse_PPI_Communities	Syn-4_0_509	501	ALDH7A1
Synapse_PPI_Communities	Syn-4_0_509	26999	CYFIP2
Synapse_PPI_Communities	Syn-4_0_509	9493	KIF23
Synapse_PPI_Communities	Syn-4_0_509	811	CALR
Synapse_PPI_Communities	Syn-4_0_509	7184	HSP90B1
Synapse_PPI_Communities	Syn-4_0_509	5034	P4HB
Synapse_PPI_Communities	Syn-4_0_509	23193	GANAB
Synapse_PPI_Communities	Syn-4_0_509	5589	PRKCSH
Synapse_PPI_Communities	Syn-4_0_509	51602	NOP58
Synapse_PPI_Communities	Syn-4_0_509	11224	RPL35
Synapse_PPI_Communities	Syn-4_0_509	7979	SHFM1
Synapse_PPI_Communities	Syn-4_0_509	10514	MYBBP1A
Synapse_PPI_Communities	Syn-4_0_509	9770	RASSF2
Synapse_PPI_Communities	Syn-4_0_509	7846	TUBA1A
Synapse_PPI_Communities	Syn-4_0_509	27430	MAT2B
Synapse_PPI_Communities	Syn-4_0_509	83992	CTTNBP2
Synapse_PPI_Communities	Syn-4_0_509	3646	EIF3E
Synapse_PPI_Communities	Syn-4_0_509	85440	DOCK7
Synapse_PPI_Communities	Syn-4_0_509	27044	SND1
Synapse_PPI_Communities	Syn-4_0_509	29109	FHOD1
Synapse_PPI_Communities	Syn-4_0_509	60	ACTB
Synapse_PPI_Communities	Syn-4_0_509	71	ACTG1
Synapse_PPI_Communities	Syn-4_0_509	5127	CDK16
Synapse_PPI_Communities	Syn-4_0_509	369	ARAF
Synapse_PPI_Communities	Syn-4_0_509	65125	WNK1
Synapse_PPI_Communities	Syn-4_0_509	6232	RPS27
Synapse_PPI_Communities	Syn-4_0_509	9101	USP8
Synapse_PPI_Communities	Syn-4_0_509	5230	PGK1
Synapse_PPI_Communities	Syn-4_0_509	2023	ENO1
Synapse_PPI_Communities	Syn-4_0_509	7167	TPI1
Synapse_PPI_Communities	Syn-4_0_509	8661	EIF3A
Synapse_PPI_Communities	Syn-4_0_509	10576	CCT2
Synapse_PPI_Communities	Syn-4_0_509	5520	PPP2R2A
Synapse_PPI_Communities	Syn-4_0_509	6801	STRN
Synapse_PPI_Communities	Syn-4_0_509	908	CCT6A
Synapse_PPI_Communities	Syn-4_0_509	23450	SF3B3
Synapse_PPI_Communities	Syn-4_0_509	6208	RPS14
Synapse_PPI_Communities	Syn-4_0_509	23327	NEDD4L
Synapse_PPI_Communities	Syn-4_0_509	6209	RPS15
Synapse_PPI_Communities	Syn-4_0_509	4486	MST1R
Synapse_PPI_Communities	Syn-4_0_509	10133	OPTN
Synapse_PPI_Communities	Syn-4_0_509	10238	DCAF7
Synapse_PPI_Communities	Syn-4_0_509	6157	RPL27A
Synapse_PPI_Communities	Syn-4_0_509	2539	G6PD
Synapse_PPI_Communities	Syn-4_0_509	6927	HNF1A
Synapse_PPI_Communities	Syn-4_0_509	6231	RPS26
Synapse_PPI_Communities	Syn-4_0_509	5524	PPP2R4
Synapse_PPI_Communities	Syn-4_0_509	29966	STRN3
Synapse_PPI_Communities	Syn-4_0_509	5531	PPP4C
Synapse_PPI_Communities	Syn-4_0_509	3799	KIF5B
Synapse_PPI_Communities	Syn-4_0_509	89953	KLC4
Synapse_PPI_Communities	Syn-4_0_509	22948	CCT5
Synapse_PPI_Communities	Syn-4_0_509	4670	HNRNPM
Synapse_PPI_Communities	Syn-4_0_509	7531	YWHAE
Synapse_PPI_Communities	Syn-4_0_509	351	APP
Synapse_PPI_Communities	Syn-4_0_509	6227	RPS21
Synapse_PPI_Communities	Syn-4_0_509	5747	PTK2
Synapse_PPI_Communities	Syn-4_0_509	23367	LARP1

Synapse_PPI_Communities	Syn-4_0_509	1029	CDKN2A
Synapse_PPI_Communities	Syn-4_0_509	8428	STK24
Synapse_PPI_Communities	Syn-4_0_509	5521	PPP2R2B
Synapse_PPI_Communities	Syn-4_0_509	1499	CTNNB1
Synapse_PPI_Communities	Syn-4_0_509	7345	UCHL1
Synapse_PPI_Communities	Syn-4_0_509	1956	EGFR
Synapse_PPI_Communities	Syn-4_0_509	3303	HSPA1A
Synapse_PPI_Communities	Syn-4_0_509	5062	PAK2
Synapse_PPI_Communities	Syn-4_0_509	135138	PACRG
Synapse_PPI_Communities	Syn-4_0_509	4904	YBX1
Synapse_PPI_Communities	Syn-4_0_509	3313	HSPA9
Synapse_PPI_Communities	Syn-4_0_509	5580	PRKCD
Synapse_PPI_Communities	Syn-4_0_509	10399	GNB2L1
Synapse_PPI_Communities	Syn-4_0_509	7414	VCL
Synapse_PPI_Communities	Syn-4_0_509	26986	PABPC1
Synapse_PPI_Communities	Syn-4_0_509	3309	HSPA5
Synapse_PPI_Communities	Syn-4_0_509	80086	TUBA4B
Synapse_PPI_Communities	Syn-4_0_509	1173	AP2M1
Synapse_PPI_Communities	Syn-4_0_509	84790	TUBA1C
Synapse_PPI_Communities	Syn-4_0_509	5515	PPP2CA
Synapse_PPI_Communities	Syn-4_0_509	25	ABL1
Synapse_PPI_Communities	Syn-4_0_509	3383	ICAM1
Synapse_PPI_Communities	Syn-4_0_509	3476	IGBP1
Synapse_PPI_Communities	Syn-4_0_509	2932	GSK3B
Synapse_PPI_Communities	Syn-4_0_509	6622	SNCA
Synapse_PPI_Communities	Syn-4_0_509	4193	MDM2
Synapse_PPI_Communities	Syn-4_0_509	5598	MAPK7
Synapse_PPI_Communities	Syn-4_0_509	6711	SPTBN1
Synapse_PPI_Communities	Syn-4_0_509	6709	SPTAN1
Synapse_PPI_Communities	Syn-4_0_509	867	CBL
Synapse_PPI_Communities	Syn-4_0_509	9531	BAG3
Synapse_PPI_Communities	Syn-4_0_509	4627	MYH9
Synapse_PPI_Communities	Syn-4_0_509	84959	UBASH3B
Synapse_PPI_Communities	Syn-4_0_509	1386	ATF2
Synapse_PPI_Communities	Syn-4_0_509	3014	H2AFX
Synapse_PPI_Communities	Syn-4_0_509	5684	PSMA3
Synapse_PPI_Communities	Syn-4_0_509	9657	IQCB1
Synapse_PPI_Communities	Syn-4_0_509	1994	ELAVL1
Synapse_PPI_Communities	Syn-4_0_509	408	ARRB1
Synapse_PPI_Communities	Syn-4_0_509	2099	ESR1
Synapse_PPI_Communities	Syn-4_0_509	9924	PAN2
Synapse_PPI_Communities	Syn-4_0_509	54165	DCUN1D1
Synapse_PPI_Communities	Syn-4_0_509	1457	CSNK2A1
Synapse_PPI_Communities	Syn-4_0_509	9775	EIF4A3
Synapse_PPI_Communities	Syn-4_0_509	1072	CFL1
Synapse_PPI_Communities	Syn-4_0_509	6464	SHC1
Synapse_PPI_Communities	Syn-4_0_509	10537	UBD
Synapse_PPI_Communities	Syn-4_0_509	708	C1QBP
Synapse_PPI_Communities	Syn-4_0_509	5052	PRDX1
Synapse_PPI_Communities	Syn-4_0_509	5518	PPP2R1A
Synapse_PPI_Communities	Syn-4_0_509	302	ANXA2
Synapse_PPI_Communities	Syn-4_0_509	5071	PARK2
Synapse_PPI_Communities	Syn-4_0_509	7412	VCAM1
Synapse_PPI_Communities	Syn-4_0_509	3326	HSP90AB1
Synapse_PPI_Communities	Syn-4_0_509	51547	SIRT7
Synapse_PPI_Communities	Syn-4_0_509	8450	CUL4B
Synapse_PPI_Communities	Syn-4_0_509	4869	NPM1
Synapse_PPI_Communities	Syn-4_0_509	154	ADRB2

Synapse_PPI_Communities	Syn-4_0_509	4843	NOS2
Synapse_PPI_Communities	Syn-4_0_509	57154	SMURF1
Synapse_PPI_Communities	Syn-4_0_509	59342	SCPEP1
Synapse_PPI_Communities	Syn-4_0_509	7317	UBA1
Synapse_PPI_Communities	Syn-4_0_509	16	AARS
Synapse_PPI_Communities	Syn-4_0_509	1827	RCAN1
Synapse_PPI_Communities	Syn-4_0_509	29127	RACGAP1
Synapse_PPI_Communities	Syn-4_0_509	55103	RALGPS2
Synapse_PPI_Communities	Syn-4_0_509	8729	GBF1
Synapse_PPI_Communities	Syn-4_0_509	728689	EIF3CL
Synapse_PPI_Communities	Syn-4_0_509	3308	HSPA4
Synapse_PPI_Communities	Syn-4_0_509	9555	H2AFY
Synapse_PPI_Communities	Syn-4_0_509	84365	MKI67IP
Synapse_PPI_Communities	Syn-4_0_509	6158	RPL28
Synapse_PPI_Communities	Syn-4_0_509	26354	GNL3
Synapse_PPI_Communities	Syn-4_0_509	5936	RBM4
Synapse_PPI_Communities	Syn-4_0_509	6500	SKP1
Synapse_PPI_Communities	Syn-4_0_509	26273	FBXO3
Synapse_PPI_Communities	Syn-4_0_509	10487	CAP1
Synapse_PPI_Communities	Syn-4_0_509	2194	FASN
Synapse_PPI_Communities	Syn-4_0_509	7170	TPM3
Synapse_PPI_Communities	Syn-4_0_509	6235	RPS29
Synapse_PPI_Communities	Syn-4_0_509	2091	FBL
Synapse_PPI_Communities	Syn-4_0_509	1508	CTSB
Synapse_PPI_Communities	Syn-4_0_509	4809	NHP2L1
Synapse_PPI_Communities	Syn-4_0_509	8664	EIF3D
Synapse_PPI_Communities	Syn-4_0_509	84206	MEX3B
Synapse_PPI_Communities	Syn-4_0_509	93144	RPS10P5
Synapse_PPI_Communities	Syn-4_0_509	9921	RNF10
Synapse_PPI_Communities	Syn-4_0_509	55646	LYAR
Synapse_PPI_Communities	Syn-4_0_509	4139	MARK1
Synapse_PPI_Communities	Syn-4_0_509	6737	TRIM21
Synapse_PPI_Communities	Syn-4_0_509	9156	EXO1
Synapse_PPI_Communities	Syn-4_0_509	1999	ELF3
Synapse_PPI_Communities	Syn-4_0_509	4839	NOP2
Synapse_PPI_Communities	Syn-4_0_509	23332	CLASP1
Synapse_PPI_Communities	Syn-4_0_509	80153	EDC3
Synapse_PPI_Communities	Syn-4_0_509	55095	SAMD4B
Synapse_PPI_Communities	Syn-4_0_509	26064	RAI14
Synapse_PPI_Communities	Syn-4_0_509	51386	EIF3L
Synapse_PPI_Communities	Syn-4_0_509	3018	HIST1H2BB
Synapse_PPI_Communities	Syn-4_0_509	29968	PSAT1
Synapse_PPI_Communities	Syn-4_0_509	6184	RPN1
Synapse_PPI_Communities	Syn-4_0_509	6651	SON
Synapse_PPI_Communities	Syn-4_0_509	4113	MAGEB2
Synapse_PPI_Communities	Syn-4_0_509	23020	SNRNP200
Synapse_PPI_Communities	Syn-4_0_509	3106	HLA-B
Synapse_PPI_Communities	Syn-4_0_509	7410	VAV2
Synapse_PPI_Communities	Syn-4_0_509	9414	TJP2
Synapse_PPI_Communities	Syn-4_0_509	4799	NFX1
Synapse_PPI_Communities	Syn-4_0_509	6897	TARS
Synapse_PPI_Communities	Syn-4_0_509	4629	MYH11
Synapse_PPI_Communities	Syn-4_0_509	10693	CCT6B
Synapse_PPI_Communities	Syn-4_0_509	54606	DDX56
Synapse_PPI_Communities	Syn-4_0_509	64092	SAMSN1
Synapse_PPI_Communities	Syn-4_0_509	5687	PSMA6
Synapse_PPI_Communities	Syn-4_0_509	4174	MCM5
Synapse_PPI_Communities	Syn-4_0_509	6154	RPL26

Synapse_PPI_Communities	Syn-4_0_509	7278	TUBA3C
Synapse_PPI_Communities	Syn-4_0_509	22954	TRIM32
Synapse_PPI_Communities	Syn-4_0_509	3310	HSPA6
Synapse_PPI_Communities	Syn-4_0_509	4140	MARK3
Synapse_PPI_Communities	Syn-4_0_509	5585	PKN1
Synapse_PPI_Communities	Syn-4_0_509	84305	WIBG
Synapse_PPI_Communities	Syn-4_0_509	23313	KIAA0930
Synapse_PPI_Communities	Syn-4_0_509	22827	PUF60
Synapse_PPI_Communities	Syn-4_0_509	9978	RBX1
Synapse_PPI_Communities	Syn-4_0_509	5998	RGS3
Synapse_PPI_Communities	Syn-4_0_509	55699	IARS2
Synapse_PPI_Communities	Syn-4_0_509	5049	PAFAH1B2
Synapse_PPI_Communities	Syn-4_0_509	2937	GSS
Synapse_PPI_Communities	Syn-4_0_509	4673	NAP1L1
Synapse_PPI_Communities	Syn-4_0_509	7001	PRDX2
Synapse_PPI_Communities	Syn-4_0_509	261726	TIPRL
Synapse_PPI_Communities	Syn-4_0_509	3621	ING1
Synapse_PPI_Communities	Syn-4_0_509	28960	DCPS
Synapse_PPI_Communities	Syn-4_0_509	821	CANX
Synapse_PPI_Communities	Syn-4_0_509	7248	TSC1
Synapse_PPI_Communities	Syn-4_0_509	1676	DFFA
Synapse_PPI_Communities	Syn-4_0_509	5128	CDK17
Synapse_PPI_Communities	Syn-4_0_509	10979	FERMT2
Synapse_PPI_Communities	Syn-4_0_509	9967	THRAP3
Synapse_PPI_Communities	Syn-4_0_509	26227	PHGDH
Synapse_PPI_Communities	Syn-4_0_509	5686	PSMA5
Synapse_PPI_Communities	Syn-4_0_509	55054	ATG16L1
Synapse_PPI_Communities	Syn-4_0_509	10458	BAIAP2
Synapse_PPI_Communities	Syn-4_0_509	6733	SRPK2
Synapse_PPI_Communities	Syn-4_0_509	7280	TUBB2A
Synapse_PPI_Communities	Syn-4_0_509	11344	TWF2
Synapse_PPI_Communities	Syn-4_0_509	3098	HK1
Synapse_PPI_Communities	Syn-4_0_509	5710	PSMD4
Synapse_PPI_Communities	Syn-4_0_509	10298	PAK4
Synapse_PPI_Communities	Syn-4_0_509	9343	EFTUD2
Synapse_PPI_Communities	Syn-4_0_509	55844	PPP2R2D
Synapse_PPI_Communities	Syn-4_0_509	7965	AIMP2
Synapse_PPI_Communities	Syn-4_0_509	6161	RPL32
Synapse_PPI_Communities	Syn-4_0_509	26259	FBXW8
Synapse_PPI_Communities	Syn-4_0_509	7128	TNFAIP3
Synapse_PPI_Communities	Syn-4_0_509	80335	WDR82
Synapse_PPI_Communities	Syn-4_0_509	10915	TCERG1
Synapse_PPI_Communities	Syn-4_0_509	56061	UBFD1
Synapse_PPI_Communities	Syn-4_0_509	2773	GNAI3
Synapse_PPI_Communities	Syn-4_0_509	6612	SUMO3
Synapse_PPI_Communities	Syn-4_0_509	9368	SLC9A3R1
Synapse_PPI_Communities	Syn-4_0_509	10413	YAP1
Synapse_PPI_Communities	Syn-4_0_509	1778	DYNC1H1
Synapse_PPI_Communities	Syn-4_0_509	2316	FLNA
Synapse_PPI_Communities	Syn-4_0_509	840	CASP7
Synapse_PPI_Communities	Syn-4_0_509	1213	CLTC
Synapse_PPI_Communities	Syn-4_0_509	30011	SH3KBP1
Synapse_PPI_Communities	Syn-4_0_509	8867	SYNJ1
Synapse_PPI_Communities	Syn-4_0_509	7337	UBE3A
Synapse_PPI_Communities	Syn-4_0_509	5522	PPP2R2C
Synapse_PPI_Communities	Syn-4_0_509	5058	PAK1
Synapse_PPI_Communities	Syn-4_0_509	9221	NOLC1
Synapse_PPI_Communities	Syn-4_0_509	3191	HNRNPL

Synapse_PPI_Communities	Syn-4_0_509	8471	IRS4
Synapse_PPI_Communities	Syn-4_0_509	8496	PPFIBP1
Synapse_PPI_Communities	Syn-4_0_509	2869	GRK5
Synapse_PPI_Communities	Syn-4_0_509	1175	AP2S1
Synapse_PPI_Communities	Syn-4_0_509	4301	MLLT4
Synapse_PPI_Communities	Syn-4_0_509	1975	EIF4B
Synapse_PPI_Communities	Syn-4_0_509	695	BTK
Synapse_PPI_Communities	Syn-4_0_509	6206	RPS12
Synapse_PPI_Communities	Syn-4_0_509	5289	PIK3C3
Synapse_PPI_Communities	Syn-4_0_509	1665	DHX15
Synapse_PPI_Communities	Syn-4_0_509	10155	TRIM28
Synapse_PPI_Communities	Syn-4_0_509	1653	DDX1
Synapse_PPI_Communities	Syn-4_0_509	64837	KLC2
Synapse_PPI_Communities	Syn-4_0_509	8161	COIL
Synapse_PPI_Communities	Syn-4_0_509	1203	CLN5
Synapse_PPI_Communities	Syn-4_0_509	3998	LMAN1
Synapse_PPI_Communities	Syn-4_0_509	2197	FAU
Synapse_PPI_Communities	Syn-4_0_509	8533	COPS3
Synapse_PPI_Communities	Syn-4_0_509	6204	RPS10
Synapse_PPI_Communities	Syn-4_0_509	3875	KRT18
Synapse_PPI_Communities	Syn-4_0_509	1027	CDKN1B
Synapse_PPI_Communities	Syn-4_0_509	5621	PRNP
Synapse_PPI_Communities	Syn-4_0_509	23411	SIRT1
Synapse_PPI_Communities	Syn-4_0_509	10130	PDIA6
Synapse_PPI_Communities	Syn-4_0_509	871	SERPINH1
Synapse_PPI_Communities	Syn-4_0_509	984	CDK11B
Synapse_PPI_Communities	Syn-4_0_509	5226	PGD
Synapse_PPI_Communities	Syn-4_0_509	3939	LDHA
Synapse_PPI_Communities	Syn-4_0_509	51631	LUC7L2
Synapse_PPI_Communities	Syn-4_0_509	26135	SERBP1
Synapse_PPI_Communities	Syn-4_0_509	4214	MAP3K1
Synapse_PPI_Communities	Syn-4_0_509	6742	SSBP1
Synapse_PPI_Communities	Syn-4_0_509	817	CAMK2D
Synapse_PPI_Communities	Syn-4_0_509	64326	RFWD2
Synapse_PPI_Communities	Syn-4_0_509	8517	IKBKG
Synapse_PPI_Communities	Syn-4_0_509	81567	TXNDC5
Synapse_PPI_Communities	Syn-4_0_509	5894	RAF1
Synapse_PPI_Communities	Syn-4_0_509	3305	HSPA1L
Synapse_PPI_Communities	Syn-4_0_509	653333	FAM86B2
Synapse_PPI_Communities	Syn-4_0_509	11198	SUPT16H
Synapse_PPI_Communities	Syn-4_0_509	5886	RAD23A
Synapse_PPI_Communities	Syn-4_0_509	2010	EMD
Synapse_PPI_Communities	Syn-4_0_509	1973	EIF4A1
Synapse_PPI_Communities	Syn-4_0_509	1983	EIF5
Synapse_PPI_Communities	Syn-4_0_509	11345	GABARAPL2
Synapse_PPI_Communities	Syn-4_0_509	5584	PRKCI
Synapse_PPI_Communities	Syn-4_0_509	142	PARP1
Synapse_PPI_Communities	Syn-4_0_509	3304	HSPA1B
Synapse_PPI_Communities	Syn-4_0_509	6428	SRSF3
Synapse_PPI_Communities	Syn-4_0_509	7334	UBE2N
Synapse_PPI_Communities	Syn-4_0_509	1207	CLNS1A
Synapse_PPI_Communities	Syn-4_0_509	25843	MOB4
Synapse_PPI_Communities	Syn-4_0_509	3800	KIF5C
Synapse_PPI_Communities	Syn-4_0_509	3692	EIF6
Synapse_PPI_Communities	Syn-4_0_509	6513	SLC2A1
Synapse_PPI_Communities	Syn-4_0_509	2923	PDIA3
Synapse_PPI_Communities	Syn-4_0_509	23095	KIF1B
Synapse_PPI_Communities	Syn-4_0_509	4628	MYH10

Synapse_PPI_Communities	Syn-4_0_509	8766	RAB11A
Synapse_PPI_Communities	Syn-4_0_509	220988	HNRNPA3
Synapse_PPI_Communities	Syn-4_0_509	6468	FBXW4
Synapse_PPI_Communities	Syn-4_0_509	92342	METTL18
Synapse_PPI_Communities	Syn-4_0_509	5223	PGAM1
Synapse_PPI_Communities	Syn-4_0_509	4088	SMAD3
Synapse_PPI_Communities	Syn-4_0_509	55611	OTUB1
Synapse_PPI_Communities	Syn-4_0_509	4851	NOTCH1
Synapse_PPI_Communities	Syn-4_0_509	3831	KLC1
Synapse_PPI_Communities	Syn-4_0_509	3945	LDHB
Synapse_PPI_Communities	Syn-4_0_509	1025	CDK9
Synapse_PPI_Communities	Syn-4_0_509	10787	NCKAP1
Synapse_PPI_Communities	Syn-4_0_509	1432	MAPK14
Synapse_PPI_Communities	Syn-4_0_509	1655	DDX5
Synapse_PPI_Communities	Syn-4_0_509	51762	RAB8B
Synapse_PPI_Communities	Syn-4_0_509	3187	HNRNPH1
Synapse_PPI_Communities	Syn-4_0_509	5296	PIK3R2
Synapse_PPI_Communities	Syn-4_0_509	3192	HNRNPU
Synapse_PPI_Communities	Syn-4_0_509	10376	TUBA1B
Synapse_PPI_Communities	Syn-4_0_509	10381	TUBB3
Synapse_PPI_Communities	Syn-4_0_509	5604	MAP2K1
Synapse_PPI_Communities	Syn-4_0_509	5591	PRKDC
Synapse_PPI_Communities	Syn-4_0_509	5111	PCNA
Synapse_PPI_Communities	Syn-4_0_509	1660	DHX9
Synapse_PPI_Communities	Syn-4_0_509	5478	PPIA
Synapse_PPI_Communities	Syn-4_0_509	3178	HNRNPA1
Synapse_PPI_Communities	Syn-4_0_509	826	CAPNS1
Synapse_PPI_Communities	Syn-4_0_509	5048	PAFAH1B1
Synapse_PPI_Communities	Syn-4_0_509	5590	PRKCZ
Synapse_PPI_Communities	Syn-4_0_509	5683	PSMA2
Synapse_PPI_Communities	Syn-4_0_509	23524	SRRM2
Synapse_PPI_Communities	Syn-4_0_509	8826	IQGAP1
Synapse_PPI_Communities	Syn-4_0_509	1488	CTBP2
Synapse_PPI_Communities	Syn-4_0_509	1937	EEF1G
Synapse_PPI_Communities	Syn-4_0_509	2631	GBAS
Synapse_PPI_Communities	Syn-4_0_509	7431	VIM
Synapse_PPI_Communities	Syn-4_0_509	10383	TUBB4B
Synapse_PPI_Communities	Syn-4_0_509	1500	CTNND1
Synapse_PPI_Communities	Syn-4_0_509	1495	CTNNA1
Synapse_PPI_Communities	Syn-4_0_509	5663	PSEN1
Synapse_PPI_Communities	Syn-4_0_509	207	AKT1
Synapse_PPI_Communities	Syn-4_0_509	3064	HTT
Synapse_PPI_Communities	Syn-4_0_509	1639	DCTN1
Synapse_PPI_Communities	Syn-4_0_509	10540	DCTN2
Synapse_PPI_Communities	Syn-4_0_509	3329	HSPD1
Synapse_PPI_Communities	Syn-4_0_509	999	CDH1
Synapse_PPI_Communities	Syn-4_0_509	6714	SRC
Synapse_PPI_Communities	Syn-4_0_509	7052	TGM2
Synapse_PPI_Communities	Syn-4_0_509	51377	UCHL5
Synapse_PPI_Communities	Syn-4_0_509	5536	PPP5C
Synapse_PPI_Communities	Syn-4_0_509	10963	STIP1
Synapse_PPI_Communities	Syn-4_0_509	9146	HGS
Synapse_PPI_Communities	Syn-4_0_509	4137	MAPT
Synapse_PPI_Communities	Syn-4_0_509	120892	LRRK2
Synapse_PPI_Communities	Syn-4_0_509	1020	CDK5
Synapse_PPI_Communities	Syn-4_0_509	8841	HDAC3
Synapse_PPI_Communities	Syn-4_0_509	506	ATP5B
Synapse_PPI_Communities	Syn-4_0_509	5245	PHB

Synapse_PPI_Communities	Syn-4_0_509	7157	TP53
Synapse_PPI_Communities	Syn-4_0_509	3190	HNRNPK
Synapse_PPI_Communities	Syn-4_0_509	1398	CRK
Synapse_PPI_Communities	Syn-4_0_509	51552	RAB14
Synapse_PPI_Communities	Syn-4_0_509	51741	WWOX
Synapse_PPI_Communities	Syn-4_0_509	988	CDC5L
Synapse_PPI_Communities	Syn-4_0_509	920	CD4
Synapse_PPI_Communities	Syn-4_0_509	8451	CUL4A
Synapse_PPI_Communities	Syn-4_0_509	5195	PEX14
Synapse_PPI_Communities	Syn-4_0_509	3184	HNRNPD
Synapse_PPI_Communities	Syn-4_0_509	1460	CSNK2B
Synapse_PPI_Communities	Syn-4_0_509	1026	CDKN1A
Synapse_PPI_Communities	Syn-4_0_509	26146	TRAF3IP1
Synapse_PPI_Communities	Syn-4_0_509	11034	DSTN
Synapse_PPI_Communities	Syn-4_0_509	7879	RAB7A
Synapse_PPI_Communities	Syn-4_0_509	5479	PPIB
Synapse_PPI_Communities	Syn-4_0_509	387082	SUMO4
Synapse_PPI_Communities	Syn-4_0_509	1627	DBN1
Synapse_PPI_Communities	Syn-4_0_509	4116	MAGOH
Synapse_PPI_Communities	Syn-4_0_509	5501	PPP1CC
Synapse_PPI_Communities	Syn-4_0_509	7086	TKT
Synapse_PPI_Communities	Syn-4_0_509	498	ATP5A1
Synapse_PPI_Communities	Syn-4_0_509	5879	RAC1
Synapse_PPI_Communities	Syn-4_0_509	5893	RAD52
Synapse_PPI_Communities	Syn-4_0_509	56257	MEPCE
Synapse_PPI_Communities	Syn-4_0_509	5216	PFN1
Synapse_PPI_Communities	Syn-4_0_509	3611	ILK
Synapse_PPI_Communities	Syn-4_0_509	10397	NDRG1
Synapse_PPI_Communities	Syn-4_0_509	6117	RPA1
Synapse_PPI_Communities	Syn-4_0_509	310	ANXA7
Synapse_PPI_Communities	Syn-4_0_509	301	ANXA1
Synapse_PPI_Communities	Syn-4_0_509	25895	METTL21B
Synapse_PPI_Communities	Syn-4_0_509	160	AP2A1
Synapse_PPI_Communities	Syn-4_0_509	161	AP2A2
Synapse_PPI_Communities	Syn-4_0_509	3301	DNAJA1
Synapse_PPI_Communities	Syn-4_0_509	7295	TXN
Synapse_PPI_Communities	Syn-4_0_509	10980	COPS6
Synapse_PPI_Communities	Syn-4_0_509	7428	VHL
Synapse_PPI_Communities	Syn-4_0_509	26260	FBXO25
Synapse_PPI_Communities	Syn-4_0_509	8726	EED
Synapse_PPI_Communities	Syn-4_0_509	5885	RAD21
Synapse_PPI_Communities	Syn-4_0_509	6119	RPA3
Synapse_PPI_Communities	Syn-4_0_509	4899	NRF1
Synapse_PPI_Communities	Syn-4_0_509	801	CALM1
Synapse_PPI_Communities	Syn-4_0_509	4609	MYC
Synapse_PPI_Communities	Syn-4_0_509	9636	ISG15
Synapse_PPI_Communities	Syn-4_0_509	6118	RPA2
Synapse_PPI_Communities	Syn-4_0_509	3837	KPNB1
Synapse_PPI_Communities	Syn-3_5_12	2891	GRIA2
Synapse_PPI_Communities	Syn-3_5_12	2901	GRIK5
Synapse_PPI_Communities	Syn-3_5_12	2895	GRID2
Synapse_PPI_Communities	Syn-3_5_12	6386	SDCBP
Synapse_PPI_Communities	Syn-3_5_12	2890	GRIA1
Synapse_PPI_Communities	Syn-3_5_12	2893	GRIA4
Synapse_PPI_Communities	Syn-3_5_12	10369	CACNG2
Synapse_PPI_Communities	Syn-3_5_12	2898	GRIK2
Synapse_PPI_Communities	Syn-3_5_12	1742	DLG4
Synapse_PPI_Communities	Syn-3_5_12	1741	DLG3

Synapse_PPI_Communities	Syn-3_5_12	23426	GRIP1
Synapse_PPI_Communities	Syn-3_5_12	9463	PICK1
Synapse_PPI_Communities	Syn-3_24_3	3897	L1CAM
Synapse_PPI_Communities	Syn-3_24_3	6900	CNTN2
Synapse_PPI_Communities	Syn-3_24_3	1463	NCAN
Synapse_PPI_Communities	Syn-3_1_3	6854	SYN2
Synapse_PPI_Communities	Syn-3_1_3	8224	SYN3
Synapse_PPI_Communities	Syn-3_1_3	9722	NOS1AP
Synapse_PPI_Communities	Syn-3_0_2312	1915	EEF1A1
Synapse_PPI_Communities	Syn-3_0_2312	6128	RPL6
Synapse_PPI_Communities	Syn-3_0_2312	6124	RPL4
Synapse_PPI_Communities	Syn-3_0_2312	1938	EEF2
Synapse_PPI_Communities	Syn-3_0_2312	6217	RPS16
Synapse_PPI_Communities	Syn-3_0_2312	55832	CAND1
Synapse_PPI_Communities	Syn-3_0_2312	140801	RPL10L
Synapse_PPI_Communities	Syn-3_0_2312	6189	RPS3A
Synapse_PPI_Communities	Syn-3_0_2312	7534	YWHAZ
Synapse_PPI_Communities	Syn-3_0_2312	4738	NEDD8
Synapse_PPI_Communities	Syn-3_0_2312	6152	RPL24
Synapse_PPI_Communities	Syn-3_0_2312	6137	RPL13
Synapse_PPI_Communities	Syn-3_0_2312	6205	RPS11
Synapse_PPI_Communities	Syn-3_0_2312	6138	RPL15
Synapse_PPI_Communities	Syn-3_0_2312	6193	RPS5
Synapse_PPI_Communities	Syn-3_0_2312	6134	RPL10
Synapse_PPI_Communities	Syn-3_0_2312	6132	RPL8
Synapse_PPI_Communities	Syn-3_0_2312	6223	RPS19
Synapse_PPI_Communities	Syn-3_0_2312	6130	RPL7A
Synapse_PPI_Communities	Syn-3_0_2312	6135	RPL11
Synapse_PPI_Communities	Syn-3_0_2312	6129	RPL7
Synapse_PPI_Communities	Syn-3_0_2312	6224	RPS20
Synapse_PPI_Communities	Syn-3_0_2312	6136	RPL12
Synapse_PPI_Communities	Syn-3_0_2312	6141	RPL18
Synapse_PPI_Communities	Syn-3_0_2312	6160	RPL31
Synapse_PPI_Communities	Syn-3_0_2312	6144	RPL21
Synapse_PPI_Communities	Syn-3_0_2312	6187	RPS2
Synapse_PPI_Communities	Syn-3_0_2312	6228	RPS23
Synapse_PPI_Communities	Syn-3_0_2312	4736	RPL10A
Synapse_PPI_Communities	Syn-3_0_2312	6191	RPS4X
Synapse_PPI_Communities	Syn-3_0_2312	6202	RPS8
Synapse_PPI_Communities	Syn-3_0_2312	6194	RPS6
Synapse_PPI_Communities	Syn-3_0_2312	6147	RPL23A
Synapse_PPI_Communities	Syn-3_0_2312	6125	RPL5
Synapse_PPI_Communities	Syn-3_0_2312	6188	RPS3
Synapse_PPI_Communities	Syn-3_0_2312	6613	SUMO2
Synapse_PPI_Communities	Syn-3_0_2312	7341	SUMO1
Synapse_PPI_Communities	Syn-3_0_2312	10987	COPS5
Synapse_PPI_Communities	Syn-3_0_2312	2335	FN1
Synapse_PPI_Communities	Syn-3_0_2312	6218	RPS17
Synapse_PPI_Communities	Syn-3_0_2312	6203	RPS9
Synapse_PPI_Communities	Syn-3_0_2312	93185	IGSF8
Synapse_PPI_Communities	Syn-3_0_2312	6155	RPL27
Synapse_PPI_Communities	Syn-3_0_2312	6122	RPL3
Synapse_PPI_Communities	Syn-3_0_2312	25873	RPL36
Synapse_PPI_Communities	Syn-3_0_2312	6233	RPS27A
Synapse_PPI_Communities	Syn-3_0_2312	6168	RPL37A
Synapse_PPI_Communities	Syn-3_0_2312	292	SLC25A5
Synapse_PPI_Communities	Syn-3_0_2312	6142	RPL18A
Synapse_PPI_Communities	Syn-3_0_2312	7532	YWHAG

Synapse_PPI_Communities	Syn-3_0_2312	7529	YWHAB
Synapse_PPI_Communities	Syn-3_0_2312	7533	YWHAH
Synapse_PPI_Communities	Syn-3_0_2312	9181	ARHGEF2
Synapse_PPI_Communities	Syn-3_0_2312	7415	VCP
Synapse_PPI_Communities	Syn-3_0_2312	6234	RPS28
Synapse_PPI_Communities	Syn-3_0_2312	9045	RPL14
Synapse_PPI_Communities	Syn-3_0_2312	3608	ILF2
Synapse_PPI_Communities	Syn-3_0_2312	10528	NOP56
Synapse_PPI_Communities	Syn-3_0_2312	6201	RPS7
Synapse_PPI_Communities	Syn-3_0_2312	6230	RPS25
Synapse_PPI_Communities	Syn-3_0_2312	6146	RPL22
Synapse_PPI_Communities	Syn-3_0_2312	4691	NCL
Synapse_PPI_Communities	Syn-3_0_2312	6139	RPL17
Synapse_PPI_Communities	Syn-3_0_2312	6133	RPL9
Synapse_PPI_Communities	Syn-3_0_2312	6159	RPL29
Synapse_PPI_Communities	Syn-3_0_2312	6156	RPL30
Synapse_PPI_Communities	Syn-3_0_2312	6229	RPS24
Synapse_PPI_Communities	Syn-3_0_2312	6207	RPS13
Synapse_PPI_Communities	Syn-3_0_2312	9349	RPL23
Synapse_PPI_Communities	Syn-3_0_2312	220717	RPLP0P6
Synapse_PPI_Communities	Syn-3_0_2312	6143	RPL19
Synapse_PPI_Communities	Syn-3_0_2312	7203	CCT3
Synapse_PPI_Communities	Syn-3_0_2312	10574	CCT7
Synapse_PPI_Communities	Syn-3_0_2312	5516	PPP2CB
Synapse_PPI_Communities	Syn-3_0_2312	10694	CCT8
Synapse_PPI_Communities	Syn-3_0_2312	10014	HDAC5
Synapse_PPI_Communities	Syn-3_0_2312	10575	CCT4
Synapse_PPI_Communities	Syn-3_0_2312	6950	TCP1
Synapse_PPI_Communities	Syn-3_0_2312	3676	ITGA4
Synapse_PPI_Communities	Syn-3_0_2312	6176	RPLP1
Synapse_PPI_Communities	Syn-3_0_2312	6175	RPLP0
Synapse_PPI_Communities	Syn-3_0_2312	3609	ILF3
Synapse_PPI_Communities	Syn-3_0_2312	3320	HSP90AA1
Synapse_PPI_Communities	Syn-3_0_2312	6210	RPS15A
Synapse_PPI_Communities	Syn-3_0_2312	2885	GRB2
Synapse_PPI_Communities	Syn-3_0_2312	8452	CUL3
Synapse_PPI_Communities	Syn-3_0_2312	6045	RNF2
Synapse_PPI_Communities	Syn-3_0_2312	10971	YWHAQ
Synapse_PPI_Communities	Syn-3_0_2312	2597	GAPDH
Synapse_PPI_Communities	Syn-3_0_2312	203068	TUBB
Synapse_PPI_Communities	Syn-3_0_2312	26270	FBXO6
Synapse_PPI_Communities	Syn-3_0_2312	1017	CDK2
Synapse_PPI_Communities	Syn-3_0_2312	3312	HSPA8
Synapse_PPI_Communities	Syn-3_0_2312	8266	UBL4A
Synapse_PPI_Communities	Syn-3_0_2312	8454	CUL1
Synapse_PPI_Communities	Syn-3_0_2312	8065	CUL5
Synapse_PPI_Communities	Syn-3_0_2312	409	ARRB2
Synapse_PPI_Communities	Syn-3_0_2312	23435	TARDBP
Synapse_PPI_Communities	Syn-3_0_2312	3921	RPSA
Synapse_PPI_Communities	Syn-3_0_2312	6780	STAU1
Synapse_PPI_Communities	Syn-3_0_2312	8453	CUL2
Synapse_PPI_Communities	Syn-3_0_2312	7316	UBC
Synapse_PPI_Communities	Syn-3_0_2312	975	CD81
Synapse_PPI_Communities	Syn-3_0_2312	6222	RPS18
Synapse_PPI_Communities	Syn-3_0_2312	55720	TSR1
Synapse_PPI_Communities	Syn-3_0_2312	51065	RPS27L
Synapse_PPI_Communities	Syn-3_0_2312	65220	NADK
Synapse_PPI_Communities	Syn-3_0_2312	501	ALDH7A1

Synapse_PPI_Communities	Syn-3_0_2312	26999	CYFIP2
Synapse_PPI_Communities	Syn-3_0_2312	9493	KIF23
Synapse_PPI_Communities	Syn-3_0_2312	811	CALR
Synapse_PPI_Communities	Syn-3_0_2312	7184	HSP90B1
Synapse_PPI_Communities	Syn-3_0_2312	5034	P4HB
Synapse_PPI_Communities	Syn-3_0_2312	23193	GANAB
Synapse_PPI_Communities	Syn-3_0_2312	5589	PRKCSH
Synapse_PPI_Communities	Syn-3_0_2312	51602	NOP58
Synapse_PPI_Communities	Syn-3_0_2312	11224	RPL35
Synapse_PPI_Communities	Syn-3_0_2312	7979	SHFM1
Synapse_PPI_Communities	Syn-3_0_2312	10514	MYBBP1A
Synapse_PPI_Communities	Syn-3_0_2312	9770	RASSF2
Synapse_PPI_Communities	Syn-3_0_2312	7846	TUBA1A
Synapse_PPI_Communities	Syn-3_0_2312	27430	MAT2B
Synapse_PPI_Communities	Syn-3_0_2312	83992	CTTNBP2
Synapse_PPI_Communities	Syn-3_0_2312	3646	EIF3E
Synapse_PPI_Communities	Syn-3_0_2312	85440	DOCK7
Synapse_PPI_Communities	Syn-3_0_2312	27044	SND1
Synapse_PPI_Communities	Syn-3_0_2312	29109	FHOD1
Synapse_PPI_Communities	Syn-3_0_2312	60	ACTB
Synapse_PPI_Communities	Syn-3_0_2312	71	ACTG1
Synapse_PPI_Communities	Syn-3_0_2312	5127	CDK16
Synapse_PPI_Communities	Syn-3_0_2312	369	ARAF
Synapse_PPI_Communities	Syn-3_0_2312	65125	WNK1
Synapse_PPI_Communities	Syn-3_0_2312	6232	RPS27
Synapse_PPI_Communities	Syn-3_0_2312	9101	USP8
Synapse_PPI_Communities	Syn-3_0_2312	5230	PGK1
Synapse_PPI_Communities	Syn-3_0_2312	2023	ENO1
Synapse_PPI_Communities	Syn-3_0_2312	7167	TPI1
Synapse_PPI_Communities	Syn-3_0_2312	8661	EIF3A
Synapse_PPI_Communities	Syn-3_0_2312	10093	ARPC4
Synapse_PPI_Communities	Syn-3_0_2312	51100	SH3GLB1
Synapse_PPI_Communities	Syn-3_0_2312	10097	ACTR2
Synapse_PPI_Communities	Syn-3_0_2312	10552	ARPC1A
Synapse_PPI_Communities	Syn-3_0_2312	51002	TPRKB
Synapse_PPI_Communities	Syn-3_0_2312	10096	ACTR3
Synapse_PPI_Communities	Syn-3_0_2312	5566	PRKACA
Synapse_PPI_Communities	Syn-3_0_2312	80755	AARSD1
Synapse_PPI_Communities	Syn-3_0_2312	10576	CCT2
Synapse_PPI_Communities	Syn-3_0_2312	5520	PPP2R2A
Synapse_PPI_Communities	Syn-3_0_2312	6801	STRN
Synapse_PPI_Communities	Syn-3_0_2312	908	CCT6A
Synapse_PPI_Communities	Syn-3_0_2312	23450	SF3B3
Synapse_PPI_Communities	Syn-3_0_2312	7165	TPD52L2
Synapse_PPI_Communities	Syn-3_0_2312	6208	RPS14
Synapse_PPI_Communities	Syn-3_0_2312	23327	NEDD4L
Synapse_PPI_Communities	Syn-3_0_2312	2771	GNAI2
Synapse_PPI_Communities	Syn-3_0_2312	2099	ESR1
Synapse_PPI_Communities	Syn-3_0_2312	2770	GNAI1
Synapse_PPI_Communities	Syn-3_0_2312	2773	GNAI3
Synapse_PPI_Communities	Syn-3_0_2312	59345	GNB4
Synapse_PPI_Communities	Syn-3_0_2312	4543	MTNR1A
Synapse_PPI_Communities	Syn-3_0_2312	154	ADRB2
Synapse_PPI_Communities	Syn-3_0_2312	6209	RPS15
Synapse_PPI_Communities	Syn-3_0_2312	529	ATP6V1E1
Synapse_PPI_Communities	Syn-3_0_2312	525	ATP6V1B1
Synapse_PPI_Communities	Syn-3_0_2312	535	ATP6V0A1
Synapse_PPI_Communities	Syn-3_0_2312	51382	ATP6V1D

Synapse_PPI_Communities	Syn-3_0_2312	526	ATP6V1B2
Synapse_PPI_Communities	Syn-3_0_2312	9296	ATP6V1F
Synapse_PPI_Communities	Syn-3_0_2312	51606	ATP6V1H
Synapse_PPI_Communities	Syn-3_0_2312	4486	MST1R
Synapse_PPI_Communities	Syn-3_0_2312	10133	OPTN
Synapse_PPI_Communities	Syn-3_0_2312	10238	DCAF7
Synapse_PPI_Communities	Syn-3_0_2312	6157	RPL27A
Synapse_PPI_Communities	Syn-3_0_2312	2539	G6PD
Synapse_PPI_Communities	Syn-3_0_2312	64924	SLC30A5
Synapse_PPI_Communities	Syn-3_0_2312	6927	HNF1A
Synapse_PPI_Communities	Syn-3_0_2312	6231	RPS26
Synapse_PPI_Communities	Syn-3_0_2312	6455	SH3GL1
Synapse_PPI_Communities	Syn-3_0_2312	10109	ARPC2
Synapse_PPI_Communities	Syn-3_0_2312	813	CALU
Synapse_PPI_Communities	Syn-3_0_2312	5524	PPP2R4
Synapse_PPI_Communities	Syn-3_0_2312	29966	STRN3
Synapse_PPI_Communities	Syn-3_0_2312	5531	PPP4C
Synapse_PPI_Communities	Syn-3_0_2312	3799	KIF5B
Synapse_PPI_Communities	Syn-3_0_2312	89953	KLC4
Synapse_PPI_Communities	Syn-3_0_2312	22948	CCT5
Synapse_PPI_Communities	Syn-3_0_2312	10094	ARPC3
Synapse_PPI_Communities	Syn-3_0_2312	4670	HNRNPM
Synapse_PPI_Communities	Syn-3_0_2312	7531	YWHAE
Synapse_PPI_Communities	Syn-3_0_2312	351	APP
Synapse_PPI_Communities	Syn-3_0_2312	10092	ARPC5
Synapse_PPI_Communities	Syn-3_0_2312	2783	GNB2
Synapse_PPI_Communities	Syn-3_0_2312	6227	RPS21
Synapse_PPI_Communities	Syn-3_0_2312	5747	PTK2
Synapse_PPI_Communities	Syn-3_0_2312	23367	LARP1
Synapse_PPI_Communities	Syn-3_0_2312	1029	CDKN2A
Synapse_PPI_Communities	Syn-3_0_2312	8428	STK24
Synapse_PPI_Communities	Syn-3_0_2312	5521	PPP2R2B
Synapse_PPI_Communities	Syn-3_0_2312	1499	CTNNB1
Synapse_PPI_Communities	Syn-3_0_2312	7345	UCHL1
Synapse_PPI_Communities	Syn-3_0_2312	1956	EGFR
Synapse_PPI_Communities	Syn-3_0_2312	3303	HSPA1A
Synapse_PPI_Communities	Syn-3_0_2312	5062	PAK2
Synapse_PPI_Communities	Syn-3_0_2312	135138	PACRG
Synapse_PPI_Communities	Syn-3_0_2312	4904	YBX1
Synapse_PPI_Communities	Syn-3_0_2312	3313	HSPA9
Synapse_PPI_Communities	Syn-3_0_2312	5580	PRKCD
Synapse_PPI_Communities	Syn-3_0_2312	10399	GNB2L1
Synapse_PPI_Communities	Syn-3_0_2312	7414	VCL
Synapse_PPI_Communities	Syn-3_0_2312	26986	PABPC1
Synapse_PPI_Communities	Syn-3_0_2312	3309	HSPA5
Synapse_PPI_Communities	Syn-3_0_2312	80086	TUBA4B
Synapse_PPI_Communities	Syn-3_0_2312	1173	AP2M1
Synapse_PPI_Communities	Syn-3_0_2312	84790	TUBA1C
Synapse_PPI_Communities	Syn-3_0_2312	5515	PPP2CA
Synapse_PPI_Communities	Syn-3_0_2312	25	ABL1
Synapse_PPI_Communities	Syn-3_0_2312	3383	ICAM1
Synapse_PPI_Communities	Syn-3_0_2312	3476	IGBP1
Synapse_PPI_Communities	Syn-3_0_2312	2932	GSK3B
Synapse_PPI_Communities	Syn-3_0_2312	6622	SNCA
Synapse_PPI_Communities	Syn-3_0_2312	4193	MDM2
Synapse_PPI_Communities	Syn-3_0_2312	5598	MAPK7
Synapse_PPI_Communities	Syn-3_0_2312	6711	SPTBN1
Synapse_PPI_Communities	Syn-3_0_2312	6709	SPTAN1

Synapse_PPI_Communities	Syn-3_0_2312	867	CBL
Synapse_PPI_Communities	Syn-3_0_2312	9531	BAG3
Synapse_PPI_Communities	Syn-3_0_2312	4627	MYH9
Synapse_PPI_Communities	Syn-3_0_2312	84959	UBASH3B
Synapse_PPI_Communities	Syn-3_0_2312	1386	ATF2
Synapse_PPI_Communities	Syn-3_0_2312	3014	H2AFX
Synapse_PPI_Communities	Syn-3_0_2312	5684	PSMA3
Synapse_PPI_Communities	Syn-3_0_2312	9657	IQCB1
Synapse_PPI_Communities	Syn-3_0_2312	1994	ELAVL1
Synapse_PPI_Communities	Syn-3_0_2312	408	ARRB1
Synapse_PPI_Communities	Syn-3_0_2312	9924	PAN2
Synapse_PPI_Communities	Syn-3_0_2312	54165	DCUN1D1
Synapse_PPI_Communities	Syn-3_0_2312	1457	CSNK2A1
Synapse_PPI_Communities	Syn-3_0_2312	9775	EIF4A3
Synapse_PPI_Communities	Syn-3_0_2312	1072	CFL1
Synapse_PPI_Communities	Syn-3_0_2312	6464	SHC1
Synapse_PPI_Communities	Syn-3_0_2312	10537	UBD
Synapse_PPI_Communities	Syn-3_0_2312	708	C1QBP
Synapse_PPI_Communities	Syn-3_0_2312	5052	PRDX1
Synapse_PPI_Communities	Syn-3_0_2312	5518	PPP2R1A
Synapse_PPI_Communities	Syn-3_0_2312	302	ANXA2
Synapse_PPI_Communities	Syn-3_0_2312	5071	PARK2
Synapse_PPI_Communities	Syn-3_0_2312	7412	VCAM1
Synapse_PPI_Communities	Syn-3_0_2312	3326	HSP90AB1
Synapse_PPI_Communities	Syn-3_0_2312	51547	SIRT7
Synapse_PPI_Communities	Syn-3_0_2312	8450	CUL4B
Synapse_PPI_Communities	Syn-3_0_2312	4869	NPM1
Synapse_PPI_Communities	Syn-3_0_2312	4843	NOS2
Synapse_PPI_Communities	Syn-3_0_2312	57154	SMURF1
Synapse_PPI_Communities	Syn-3_0_2312	59342	SCPEP1
Synapse_PPI_Communities	Syn-3_0_2312	7317	UBA1
Synapse_PPI_Communities	Syn-3_0_2312	16	AARS
Synapse_PPI_Communities	Syn-3_0_2312	1827	RCAN1
Synapse_PPI_Communities	Syn-3_0_2312	29127	RACGAP1
Synapse_PPI_Communities	Syn-3_0_2312	55103	RALGPS2
Synapse_PPI_Communities	Syn-3_0_2312	8729	GBF1
Synapse_PPI_Communities	Syn-3_0_2312	8674	VAMP4
Synapse_PPI_Communities	Syn-3_0_2312	55690	PACS1
Synapse_PPI_Communities	Syn-3_0_2312	728689	EIF3CL
Synapse_PPI_Communities	Syn-3_0_2312	3308	HSPA4
Synapse_PPI_Communities	Syn-3_0_2312	9555	H2AFY
Synapse_PPI_Communities	Syn-3_0_2312	84365	MKI67IP
Synapse_PPI_Communities	Syn-3_0_2312	6158	RPL28
Synapse_PPI_Communities	Syn-3_0_2312	26354	GNL3
Synapse_PPI_Communities	Syn-3_0_2312	5936	RBM4
Synapse_PPI_Communities	Syn-3_0_2312	6500	SKP1
Synapse_PPI_Communities	Syn-3_0_2312	26273	FBXO3
Synapse_PPI_Communities	Syn-3_0_2312	10487	CAP1
Synapse_PPI_Communities	Syn-3_0_2312	2194	FASN
Synapse_PPI_Communities	Syn-3_0_2312	7170	TPM3
Synapse_PPI_Communities	Syn-3_0_2312	6235	RPS29
Synapse_PPI_Communities	Syn-3_0_2312	2091	FBL
Synapse_PPI_Communities	Syn-3_0_2312	1508	CTSB
Synapse_PPI_Communities	Syn-3_0_2312	9456	HOMER1
Synapse_PPI_Communities	Syn-3_0_2312	6262	RYR2
Synapse_PPI_Communities	Syn-3_0_2312	6261	RYR1
Synapse_PPI_Communities	Syn-3_0_2312	4809	NHP2L1
Synapse_PPI_Communities	Syn-3_0_2312	5861	RAB1A

Synapse_PPI_Communities	Syn-3_0_2312	2923	PDIA3
Synapse_PPI_Communities	Syn-3_0_2312	136319	MTPN
Synapse_PPI_Communities	Syn-3_0_2312	523	ATP6V1A
Synapse_PPI_Communities	Syn-3_0_2312	8664	EIF3D
Synapse_PPI_Communities	Syn-3_0_2312	84206	MEX3B
Synapse_PPI_Communities	Syn-3_0_2312	93144	RPS10P5
Synapse_PPI_Communities	Syn-3_0_2312	9921	RNF10
Synapse_PPI_Communities	Syn-3_0_2312	9368	SLC9A3R1
Synapse_PPI_Communities	Syn-3_0_2312	9520	NPEPPS
Synapse_PPI_Communities	Syn-3_0_2312	26007	DAK
Synapse_PPI_Communities	Syn-3_0_2312	55646	LYAR
Synapse_PPI_Communities	Syn-3_0_2312	4139	MARK1
Synapse_PPI_Communities	Syn-3_0_2312	6737	TRIM21
Synapse_PPI_Communities	Syn-3_0_2312	9156	EXO1
Synapse_PPI_Communities	Syn-3_0_2312	10287	RGS19
Synapse_PPI_Communities	Syn-3_0_2312	1999	ELF3
Synapse_PPI_Communities	Syn-3_0_2312	4839	NOP2
Synapse_PPI_Communities	Syn-3_0_2312	23332	CLASP1
Synapse_PPI_Communities	Syn-3_0_2312	80153	EDC3
Synapse_PPI_Communities	Syn-3_0_2312	55095	SAMD4B
Synapse_PPI_Communities	Syn-3_0_2312	26064	RAI14
Synapse_PPI_Communities	Syn-3_0_2312	51386	EIF3L
Synapse_PPI_Communities	Syn-3_0_2312	3018	HIST1H2BB
Synapse_PPI_Communities	Syn-3_0_2312	7280	TUBB2A
Synapse_PPI_Communities	Syn-3_0_2312	55968	NSFL1C
Synapse_PPI_Communities	Syn-3_0_2312	3098	HK1
Synapse_PPI_Communities	Syn-3_0_2312	9373	PLAA
Synapse_PPI_Communities	Syn-3_0_2312	29968	PSAT1
Synapse_PPI_Communities	Syn-3_0_2312	1268	CNR1
Synapse_PPI_Communities	Syn-3_0_2312	6184	RPN1
Synapse_PPI_Communities	Syn-3_0_2312	6651	SON
Synapse_PPI_Communities	Syn-3_0_2312	4905	NSF
Synapse_PPI_Communities	Syn-3_0_2312	8417	STX7
Synapse_PPI_Communities	Syn-3_0_2312	10228	STX6
Synapse_PPI_Communities	Syn-3_0_2312	8773	SNAP23
Synapse_PPI_Communities	Syn-3_0_2312	4113	MAGEB2
Synapse_PPI_Communities	Syn-3_0_2312	23020	SNRNP200
Synapse_PPI_Communities	Syn-3_0_2312	3106	HLA-B
Synapse_PPI_Communities	Syn-3_0_2312	9522	SCAMP1
Synapse_PPI_Communities	Syn-3_0_2312	10066	SCAMP2
Synapse_PPI_Communities	Syn-3_0_2312	7410	VAV2
Synapse_PPI_Communities	Syn-3_0_2312	9414	TJP2
Synapse_PPI_Communities	Syn-3_0_2312	54974	THG1L
Synapse_PPI_Communities	Syn-3_0_2312	64407	RGS18
Synapse_PPI_Communities	Syn-3_0_2312	3760	KCNJ3
Synapse_PPI_Communities	Syn-3_0_2312	2782	GNB1
Synapse_PPI_Communities	Syn-3_0_2312	4799	NFX1
Synapse_PPI_Communities	Syn-3_0_2312	6897	TARS
Synapse_PPI_Communities	Syn-3_0_2312	11345	GABARAPL2
Synapse_PPI_Communities	Syn-3_0_2312	2631	GBAS
Synapse_PPI_Communities	Syn-3_0_2312	8508	NIPSNAP1
Synapse_PPI_Communities	Syn-3_0_2312	23710	GABARAPL1
Synapse_PPI_Communities	Syn-3_0_2312	4629	MYH11
Synapse_PPI_Communities	Syn-3_0_2312	4924	NUCB1
Synapse_PPI_Communities	Syn-3_0_2312	10693	CCT6B
Synapse_PPI_Communities	Syn-3_0_2312	54606	DDX56
Synapse_PPI_Communities	Syn-3_0_2312	64092	SAMSN1
Synapse_PPI_Communities	Syn-3_0_2312	5687	PSMA6

Synapse_PPI_Communities	Syn-3_0_2312	2017	CTTN
Synapse_PPI_Communities	Syn-3_0_2312	7456	WIPF1
Synapse_PPI_Communities	Syn-3_0_2312	2664	GDI1
Synapse_PPI_Communities	Syn-3_0_2312	9805	SCRN1
Synapse_PPI_Communities	Syn-3_0_2312	90933	TRIM41
Synapse_PPI_Communities	Syn-3_0_2312	4174	MCM5
Synapse_PPI_Communities	Syn-3_0_2312	6154	RPL26
Synapse_PPI_Communities	Syn-3_0_2312	7278	TUBA3C
Synapse_PPI_Communities	Syn-3_0_2312	5664	PSEN2
Synapse_PPI_Communities	Syn-3_0_2312	54536	EXOC6
Synapse_PPI_Communities	Syn-3_0_2312	4846	NOS3
Synapse_PPI_Communities	Syn-3_0_2312	22954	TRIM32
Synapse_PPI_Communities	Syn-3_0_2312	23385	NCSTN
Synapse_PPI_Communities	Syn-3_0_2312	3550	IK
Synapse_PPI_Communities	Syn-3_0_2312	26227	PHGDH
Synapse_PPI_Communities	Syn-3_0_2312	3310	HSPA6
Synapse_PPI_Communities	Syn-3_0_2312	4140	MARK3
Synapse_PPI_Communities	Syn-3_0_2312	1152	CKB
Synapse_PPI_Communities	Syn-3_0_2312	54187	NANS
Synapse_PPI_Communities	Syn-3_0_2312	5585	PKN1
Synapse_PPI_Communities	Syn-3_0_2312	84305	WIBG
Synapse_PPI_Communities	Syn-3_0_2312	309	ANXA6
Synapse_PPI_Communities	Syn-3_0_2312	3417	IDH1
Synapse_PPI_Communities	Syn-3_0_2312	7328	UBE2H
Synapse_PPI_Communities	Syn-3_0_2312	2805	GOT1
Synapse_PPI_Communities	Syn-3_0_2312	10312	TCIRG1
Synapse_PPI_Communities	Syn-3_0_2312	57180	ACTR3B
Synapse_PPI_Communities	Syn-3_0_2312	23313	KIAA0930
Synapse_PPI_Communities	Syn-3_0_2312	22827	PUF60
Synapse_PPI_Communities	Syn-3_0_2312	9978	RBX1
Synapse_PPI_Communities	Syn-3_0_2312	5998	RGS3
Synapse_PPI_Communities	Syn-3_0_2312	26059	ERC2
Synapse_PPI_Communities	Syn-3_0_2312	28964	GIT1
Synapse_PPI_Communities	Syn-3_0_2312	8541	PPFIA3
Synapse_PPI_Communities	Syn-3_0_2312	5879	RAC1
Synapse_PPI_Communities	Syn-3_0_2312	998	CDC42
Synapse_PPI_Communities	Syn-3_0_2312	8506	CNTNAP1
Synapse_PPI_Communities	Syn-3_0_2312	4899	NRF1
Synapse_PPI_Communities	Syn-3_0_2312	10053	AP1M2
Synapse_PPI_Communities	Syn-3_0_2312	9545	RAB3D
Synapse_PPI_Communities	Syn-3_0_2312	22983	MAST1
Synapse_PPI_Communities	Syn-3_0_2312	55699	IARS2
Synapse_PPI_Communities	Syn-3_0_2312	5864	RAB3A
Synapse_PPI_Communities	Syn-3_0_2312	51295	ECSIT
Synapse_PPI_Communities	Syn-3_0_2312	7001	PRDX2
Synapse_PPI_Communities	Syn-3_0_2312	5910	RAP1GDS1
Synapse_PPI_Communities	Syn-3_0_2312	5049	PAFAH1B2
Synapse_PPI_Communities	Syn-3_0_2312	2937	GSS
Synapse_PPI_Communities	Syn-3_0_2312	8499	PPFIA2
Synapse_PPI_Communities	Syn-3_0_2312	4507	MTAP
Synapse_PPI_Communities	Syn-3_0_2312	118	ADD1
Synapse_PPI_Communities	Syn-3_0_2312	6710	SPTB
Synapse_PPI_Communities	Syn-3_0_2312	4673	NAP1L1
Synapse_PPI_Communities	Syn-3_0_2312	261726	TIPRL
Synapse_PPI_Communities	Syn-3_0_2312	3621	ING1
Synapse_PPI_Communities	Syn-3_0_2312	28960	DCPS
Synapse_PPI_Communities	Syn-3_0_2312	6004	RGS16
Synapse_PPI_Communities	Syn-3_0_2312	821	CANX

Synapse_PPI_Communities	Syn-3_0_2312	79068	FTO
Synapse_PPI_Communities	Syn-3_0_2312	7248	TSC1
Synapse_PPI_Communities	Syn-3_0_2312	1676	DFFA
Synapse_PPI_Communities	Syn-3_0_2312	5128	CDK17
Synapse_PPI_Communities	Syn-3_0_2312	10979	FERMT2
Synapse_PPI_Communities	Syn-3_0_2312	9967	THRAP3
Synapse_PPI_Communities	Syn-3_0_2312	5686	PSMA5
Synapse_PPI_Communities	Syn-3_0_2312	51678	MPP6
Synapse_PPI_Communities	Syn-3_0_2312	3768	KCNJ12
Synapse_PPI_Communities	Syn-3_0_2312	8825	LIN7A
Synapse_PPI_Communities	Syn-3_0_2312	10497	UNC13B
Synapse_PPI_Communities	Syn-3_0_2312	6844	VAMP2
Synapse_PPI_Communities	Syn-3_0_2312	112755	STX1B
Synapse_PPI_Communities	Syn-3_0_2312	824	CAPN2
Synapse_PPI_Communities	Syn-3_0_2312	8490	RGS5
Synapse_PPI_Communities	Syn-3_0_2312	2778	GNAS
Synapse_PPI_Communities	Syn-3_0_2312	6915	TBXA2R
Synapse_PPI_Communities	Syn-3_0_2312	2767	GNA11
Synapse_PPI_Communities	Syn-3_0_2312	55054	ATG16L1
Synapse_PPI_Communities	Syn-3_0_2312	11034	DSTN
Synapse_PPI_Communities	Syn-3_0_2312	55611	OTUB1
Synapse_PPI_Communities	Syn-3_0_2312	4093	SMAD9
Synapse_PPI_Communities	Syn-3_0_2312	5663	PSEN1
Synapse_PPI_Communities	Syn-3_0_2312	2	A2M
Synapse_PPI_Communities	Syn-3_0_2312	10458	BAIAP2
Synapse_PPI_Communities	Syn-3_0_2312	6733	SRPK2
Synapse_PPI_Communities	Syn-3_0_2312	55970	GNG12
Synapse_PPI_Communities	Syn-3_0_2312	10211	FLOT1
Synapse_PPI_Communities	Syn-3_0_2312	2319	FLOT2
Synapse_PPI_Communities	Syn-3_0_2312	60491	NIF3L1
Synapse_PPI_Communities	Syn-3_0_2312	11344	TWF2
Synapse_PPI_Communities	Syn-3_0_2312	8573	CASK
Synapse_PPI_Communities	Syn-3_0_2312	493	ATP2B4
Synapse_PPI_Communities	Syn-3_0_2312	55914	ERBB2IP
Synapse_PPI_Communities	Syn-3_0_2312	140545	RNF32
Synapse_PPI_Communities	Syn-3_0_2312	348	APOE
Synapse_PPI_Communities	Syn-3_0_2312	5710	PSMD4
Synapse_PPI_Communities	Syn-3_0_2312	1740	DLG2
Synapse_PPI_Communities	Syn-3_0_2312	2903	GRIN2A
Synapse_PPI_Communities	Syn-3_0_2312	9229	DLGAP1
Synapse_PPI_Communities	Syn-3_0_2312	10298	PAK4
Synapse_PPI_Communities	Syn-3_0_2312	9343	EFTUD2
Synapse_PPI_Communities	Syn-3_0_2312	55844	PPP2R2D
Synapse_PPI_Communities	Syn-3_0_2312	11336	EXOC3
Synapse_PPI_Communities	Syn-3_0_2312	55770	EXOC2
Synapse_PPI_Communities	Syn-3_0_2312	4644	MYO5A
Synapse_PPI_Communities	Syn-3_0_2312	55748	CNDP2
Synapse_PPI_Communities	Syn-3_0_2312	7965	AIMP2
Synapse_PPI_Communities	Syn-3_0_2312	6161	RPL32
Synapse_PPI_Communities	Syn-3_0_2312	26259	FBXW8
Synapse_PPI_Communities	Syn-3_0_2312	81873	ARPC5L
Synapse_PPI_Communities	Syn-3_0_2312	7128	TNFAIP3
Synapse_PPI_Communities	Syn-3_0_2312	80335	WDR82
Synapse_PPI_Communities	Syn-3_0_2312	10095	ARPC1B
Synapse_PPI_Communities	Syn-3_0_2312	10915	TCERG1
Synapse_PPI_Communities	Syn-3_0_2312	56061	UBFD1
Synapse_PPI_Communities	Syn-3_0_2312	6612	SUMO3
Synapse_PPI_Communities	Syn-3_0_2312	1742	DLG4

Synapse_PPI_Communities	Syn-3_0_2312	23265	EXOC7
Synapse_PPI_Communities	Syn-3_0_2312	10640	EXOC5
Synapse_PPI_Communities	Syn-3_0_2312	5868	RAB5A
Synapse_PPI_Communities	Syn-3_0_2312	5867	RAB4A
Synapse_PPI_Communities	Syn-3_0_2312	79874	RABEP2
Synapse_PPI_Communities	Syn-3_0_2312	10413	YAP1
Synapse_PPI_Communities	Syn-3_0_2312	1778	DYNC1H1
Synapse_PPI_Communities	Syn-3_0_2312	2316	FLNA
Synapse_PPI_Communities	Syn-3_0_2312	840	CASP7
Synapse_PPI_Communities	Syn-3_0_2312	1213	CLTC
Synapse_PPI_Communities	Syn-3_0_2312	30011	SH3KBP1
Synapse_PPI_Communities	Syn-3_0_2312	8867	SYNJ1
Synapse_PPI_Communities	Syn-3_0_2312	7337	UBE3A
Synapse_PPI_Communities	Syn-3_0_2312	5522	PPP2R2C
Synapse_PPI_Communities	Syn-3_0_2312	5058	PAK1
Synapse_PPI_Communities	Syn-3_0_2312	161	AP2A2
Synapse_PPI_Communities	Syn-3_0_2312	160	AP2A1
Synapse_PPI_Communities	Syn-3_0_2312	273	AMPH
Synapse_PPI_Communities	Syn-3_0_2312	274	BIN1
Synapse_PPI_Communities	Syn-3_0_2312	9221	NOLC1
Synapse_PPI_Communities	Syn-3_0_2312	3191	HNRNPL
Synapse_PPI_Communities	Syn-3_0_2312	8471	IRS4
Synapse_PPI_Communities	Syn-3_0_2312	8496	PPFIBP1
Synapse_PPI_Communities	Syn-3_0_2312	2869	GRK5
Synapse_PPI_Communities	Syn-3_0_2312	1175	AP2S1
Synapse_PPI_Communities	Syn-3_0_2312	4301	MLLT4
Synapse_PPI_Communities	Syn-3_0_2312	8775	NAPA
Synapse_PPI_Communities	Syn-3_0_2312	6811	STX5
Synapse_PPI_Communities	Syn-3_0_2312	6616	SNAP25
Synapse_PPI_Communities	Syn-3_0_2312	2977	GUCY1A2
Synapse_PPI_Communities	Syn-3_0_2312	1975	EIF4B
Synapse_PPI_Communities	Syn-3_0_2312	140735	DYNLL2
Synapse_PPI_Communities	Syn-3_0_2312	6810	STX4
Synapse_PPI_Communities	Syn-3_0_2312	9342	SNAP29
Synapse_PPI_Communities	Syn-3_0_2312	6845	VAMP7
Synapse_PPI_Communities	Syn-3_0_2312	23673	STX12
Synapse_PPI_Communities	Syn-3_0_2312	5567	PRKACB
Synapse_PPI_Communities	Syn-3_0_2312	9601	PDIA4
Synapse_PPI_Communities	Syn-3_0_2312	695	BTK
Synapse_PPI_Communities	Syn-3_0_2312	2037	EPB41L2
Synapse_PPI_Communities	Syn-3_0_2312	149371	EXOC8
Synapse_PPI_Communities	Syn-3_0_2312	6206	RPS12
Synapse_PPI_Communities	Syn-3_0_2312	10541	ANP32B
Synapse_PPI_Communities	Syn-3_0_2312	5289	PIK3C3
Synapse_PPI_Communities	Syn-3_0_2312	1665	DHX15
Synapse_PPI_Communities	Syn-3_0_2312	10155	TRIM28
Synapse_PPI_Communities	Syn-3_0_2312	5594	MAPK1
Synapse_PPI_Communities	Syn-3_0_2312	1653	DDX1
Synapse_PPI_Communities	Syn-3_0_2312	64837	KLC2
Synapse_PPI_Communities	Syn-3_0_2312	8161	COIL
Synapse_PPI_Communities	Syn-3_0_2312	1203	CLN5
Synapse_PPI_Communities	Syn-3_0_2312	528	ATP6V1C1
Synapse_PPI_Communities	Syn-3_0_2312	3998	LMAN1
Synapse_PPI_Communities	Syn-3_0_2312	2197	FAU
Synapse_PPI_Communities	Syn-3_0_2312	8533	COPS3
Synapse_PPI_Communities	Syn-3_0_2312	6204	RPS10
Synapse_PPI_Communities	Syn-3_0_2312	8655	DYNLL1
Synapse_PPI_Communities	Syn-3_0_2312	4130	MAP1A

Synapse_PPI_Communities	Syn-3_0_2312	9341	VAMP3
Synapse_PPI_Communities	Syn-3_0_2312	2054	STX2
Synapse_PPI_Communities	Syn-3_0_2312	8673	VAMP8
Synapse_PPI_Communities	Syn-3_0_2312	6857	SYT1
Synapse_PPI_Communities	Syn-3_0_2312	6809	STX3
Synapse_PPI_Communities	Syn-3_0_2312	495	ATP4A
Synapse_PPI_Communities	Syn-3_0_2312	2768	GNA12
Synapse_PPI_Communities	Syn-3_0_2312	5579	PRKCB
Synapse_PPI_Communities	Syn-3_0_2312	3875	KRT18
Synapse_PPI_Communities	Syn-3_0_2312	1027	CDKN1B
Synapse_PPI_Communities	Syn-3_0_2312	5621	PRNP
Synapse_PPI_Communities	Syn-3_0_2312	23411	SIRT1
Synapse_PPI_Communities	Syn-3_0_2312	10130	PDIA6
Synapse_PPI_Communities	Syn-3_0_2312	871	SERPINH1
Synapse_PPI_Communities	Syn-3_0_2312	2665	GDI2
Synapse_PPI_Communities	Syn-3_0_2312	984	CDK11B
Synapse_PPI_Communities	Syn-3_0_2312	5226	PGD
Synapse_PPI_Communities	Syn-3_0_2312	3939	LDHA
Synapse_PPI_Communities	Syn-3_0_2312	51631	LUC7L2
Synapse_PPI_Communities	Syn-3_0_2312	26135	SERBP1
Synapse_PPI_Communities	Syn-3_0_2312	4214	MAP3K1
Synapse_PPI_Communities	Syn-3_0_2312	6742	SSBP1
Synapse_PPI_Communities	Syn-3_0_2312	817	CAMK2D
Synapse_PPI_Communities	Syn-3_0_2312	64326	RFWD2
Synapse_PPI_Communities	Syn-3_0_2312	8517	IKBKG
Synapse_PPI_Communities	Syn-3_0_2312	81567	TXNDC5
Synapse_PPI_Communities	Syn-3_0_2312	5894	RAF1
Synapse_PPI_Communities	Syn-3_0_2312	3305	HSPA1L
Synapse_PPI_Communities	Syn-3_0_2312	653333	FAM86B2
Synapse_PPI_Communities	Syn-3_0_2312	11198	SUPT16H
Synapse_PPI_Communities	Syn-3_0_2312	54888	NSUN2
Synapse_PPI_Communities	Syn-3_0_2312	5886	RAD23A
Synapse_PPI_Communities	Syn-3_0_2312	2010	EMD
Synapse_PPI_Communities	Syn-3_0_2312	1973	EIF4A1
Synapse_PPI_Communities	Syn-3_0_2312	801	CALM1
Synapse_PPI_Communities	Syn-3_0_2312	4609	MYC
Synapse_PPI_Communities	Syn-3_0_2312	818	CAMK2G
Synapse_PPI_Communities	Syn-3_0_2312	51035	UBXN1
Synapse_PPI_Communities	Syn-3_0_2312	1983	EIF5
Synapse_PPI_Communities	Syn-3_0_2312	5584	PRKCI
Synapse_PPI_Communities	Syn-3_0_2312	142	PARP1
Synapse_PPI_Communities	Syn-3_0_2312	3304	HSPA1B
Synapse_PPI_Communities	Syn-3_0_2312	6428	SRSF3
Synapse_PPI_Communities	Syn-3_0_2312	1244	ABCC2
Synapse_PPI_Communities	Syn-3_0_2312	8301	PICALM
Synapse_PPI_Communities	Syn-3_0_2312	1780	DYNC1I1
Synapse_PPI_Communities	Syn-3_0_2312	10381	TUBB3
Synapse_PPI_Communities	Syn-3_0_2312	310	ANXA7
Synapse_PPI_Communities	Syn-3_0_2312	7334	UBE2N
Synapse_PPI_Communities	Syn-3_0_2312	1207	CLNS1A
Synapse_PPI_Communities	Syn-3_0_2312	25843	MOB4
Synapse_PPI_Communities	Syn-3_0_2312	3800	KIF5C
Synapse_PPI_Communities	Syn-3_0_2312	3692	EIF6
Synapse_PPI_Communities	Syn-3_0_2312	6513	SLC2A1
Synapse_PPI_Communities	Syn-3_0_2312	1434	CSE1L
Synapse_PPI_Communities	Syn-3_0_2312	23095	KIF1B
Synapse_PPI_Communities	Syn-3_0_2312	8766	RAB11A
Synapse_PPI_Communities	Syn-3_0_2312	9230	RAB11B

Synapse_PPI_Communities	Syn-3_0_2312	4628	MYH10
Synapse_PPI_Communities	Syn-3_0_2312	7879	RAB7A
Synapse_PPI_Communities	Syn-3_0_2312	220988	HNRNPA3
Synapse_PPI_Communities	Syn-3_0_2312	6468	FBXW4
Synapse_PPI_Communities	Syn-3_0_2312	6426	SRSF1
Synapse_PPI_Communities	Syn-3_0_2312	3848	KRT1
Synapse_PPI_Communities	Syn-3_0_2312	92342	METTL18
Synapse_PPI_Communities	Syn-3_0_2312	5223	PGAM1
Synapse_PPI_Communities	Syn-3_0_2312	4088	SMAD3
Synapse_PPI_Communities	Syn-3_0_2312	4851	NOTCH1
Synapse_PPI_Communities	Syn-3_0_2312	3831	KLC1
Synapse_PPI_Communities	Syn-3_0_2312	7416	VDAC1
Synapse_PPI_Communities	Syn-3_0_2312	5581	PRKCE
Synapse_PPI_Communities	Syn-3_0_2312	3945	LDHB
Synapse_PPI_Communities	Syn-3_0_2312	1025	CDK9
Synapse_PPI_Communities	Syn-3_0_2312	10787	NCKAP1
Synapse_PPI_Communities	Syn-3_0_2312	3192	HNRNPU
Synapse_PPI_Communities	Syn-3_0_2312	3920	LAMP2
Synapse_PPI_Communities	Syn-3_0_2312	7284	TUFM
Synapse_PPI_Communities	Syn-3_0_2312	3728	JUP
Synapse_PPI_Communities	Syn-3_0_2312	1000	CDH2
Synapse_PPI_Communities	Syn-3_0_2312	51762	RAB8B
Synapse_PPI_Communities	Syn-3_0_2312	1432	MAPK14
Synapse_PPI_Communities	Syn-3_0_2312	1655	DDX5
Synapse_PPI_Communities	Syn-3_0_2312	3187	HNRNPH1
Synapse_PPI_Communities	Syn-3_0_2312	5296	PIK3R2
Synapse_PPI_Communities	Syn-3_0_2312	81876	RAB1B
Synapse_PPI_Communities	Syn-3_0_2312	10376	TUBA1B
Synapse_PPI_Communities	Syn-3_0_2312	4218	RAB8A
Synapse_PPI_Communities	Syn-3_0_2312	5604	MAP2K1
Synapse_PPI_Communities	Syn-3_0_2312	9218	VAPA
Synapse_PPI_Communities	Syn-3_0_2312	7419	VDAC3
Synapse_PPI_Communities	Syn-3_0_2312	5591	PRKDC
Synapse_PPI_Communities	Syn-3_0_2312	8878	SQSTM1
Synapse_PPI_Communities	Syn-3_0_2312	5111	PCNA
Synapse_PPI_Communities	Syn-3_0_2312	1660	DHX9
Synapse_PPI_Communities	Syn-3_0_2312	5478	PPIA
Synapse_PPI_Communities	Syn-3_0_2312	3178	HNRNPA1
Synapse_PPI_Communities	Syn-3_0_2312	826	CAPNS1
Synapse_PPI_Communities	Syn-3_0_2312	5048	PAFAH1B1
Synapse_PPI_Communities	Syn-3_0_2312	5590	PRKCZ
Synapse_PPI_Communities	Syn-3_0_2312	5683	PSMA2
Synapse_PPI_Communities	Syn-3_0_2312	10857	PGRMC1
Synapse_PPI_Communities	Syn-3_0_2312	10134	BCAP31
Synapse_PPI_Communities	Syn-3_0_2312	6117	RPA1
Synapse_PPI_Communities	Syn-3_0_2312	6118	RPA2
Synapse_PPI_Communities	Syn-3_0_2312	6119	RPA3
Synapse_PPI_Communities	Syn-3_0_2312	23524	SRRM2
Synapse_PPI_Communities	Syn-3_0_2312	8826	IQGAP1
Synapse_PPI_Communities	Syn-3_0_2312	1488	CTBP2
Synapse_PPI_Communities	Syn-3_0_2312	1937	EEF1G
Synapse_PPI_Communities	Syn-3_0_2312	7431	VIM
Synapse_PPI_Communities	Syn-3_0_2312	10383	TUBB4B
Synapse_PPI_Communities	Syn-3_0_2312	1500	CTNND1
Synapse_PPI_Communities	Syn-3_0_2312	1495	CTNNA1
Synapse_PPI_Communities	Syn-3_0_2312	207	AKT1
Synapse_PPI_Communities	Syn-3_0_2312	3064	HTT
Synapse_PPI_Communities	Syn-3_0_2312	1639	DCTN1

Synapse_PPI_Communities	Syn-3_0_2312	10540	DCTN2
Synapse_PPI_Communities	Syn-3_0_2312	3329	HSPD1
Synapse_PPI_Communities	Syn-3_0_2312	999	CDH1
Synapse_PPI_Communities	Syn-3_0_2312	6714	SRC
Synapse_PPI_Communities	Syn-3_0_2312	1400	CRMP1
Synapse_PPI_Communities	Syn-3_0_2312	7052	TGM2
Synapse_PPI_Communities	Syn-3_0_2312	51377	UCHL5
Synapse_PPI_Communities	Syn-3_0_2312	5536	PPP5C
Synapse_PPI_Communities	Syn-3_0_2312	10963	STIP1
Synapse_PPI_Communities	Syn-3_0_2312	5595	MAPK3
Synapse_PPI_Communities	Syn-3_0_2312	857	CAV1
Synapse_PPI_Communities	Syn-3_0_2312	9146	HGS
Synapse_PPI_Communities	Syn-3_0_2312	4137	MAPT
Synapse_PPI_Communities	Syn-3_0_2312	120892	LRRK2
Synapse_PPI_Communities	Syn-3_0_2312	1020	CDK5
Synapse_PPI_Communities	Syn-3_0_2312	8841	HDAC3
Synapse_PPI_Communities	Syn-3_0_2312	506	ATP5B
Synapse_PPI_Communities	Syn-3_0_2312	5245	PHB
Synapse_PPI_Communities	Syn-3_0_2312	7157	TP53
Synapse_PPI_Communities	Syn-3_0_2312	3190	HNRNPK
Synapse_PPI_Communities	Syn-3_0_2312	1398	CRK
Synapse_PPI_Communities	Syn-3_0_2312	51552	RAB14
Synapse_PPI_Communities	Syn-3_0_2312	51741	WWOX
Synapse_PPI_Communities	Syn-3_0_2312	988	CDC5L
Synapse_PPI_Communities	Syn-3_0_2312	920	CD4
Synapse_PPI_Communities	Syn-3_0_2312	8451	CUL4A
Synapse_PPI_Communities	Syn-3_0_2312	5195	PEX14
Synapse_PPI_Communities	Syn-3_0_2312	3184	HNRNPD
Synapse_PPI_Communities	Syn-3_0_2312	1460	CSNK2B
Synapse_PPI_Communities	Syn-3_0_2312	1026	CDKN1A
Synapse_PPI_Communities	Syn-3_0_2312	26146	TRAF3IP1
Synapse_PPI_Communities	Syn-3_0_2312	5479	PPIB
Synapse_PPI_Communities	Syn-3_0_2312	387082	SUMO4
Synapse_PPI_Communities	Syn-3_0_2312	1627	DBN1
Synapse_PPI_Communities	Syn-3_0_2312	4116	MAGOH
Synapse_PPI_Communities	Syn-3_0_2312	5501	PPP1CC
Synapse_PPI_Communities	Syn-3_0_2312	7086	TKT
Synapse_PPI_Communities	Syn-3_0_2312	498	ATP5A1
Synapse_PPI_Communities	Syn-3_0_2312	5893	RAD52
Synapse_PPI_Communities	Syn-3_0_2312	56257	MEPCE
Synapse_PPI_Communities	Syn-3_0_2312	5216	PFN1
Synapse_PPI_Communities	Syn-3_0_2312	3611	ILK
Synapse_PPI_Communities	Syn-3_0_2312	10397	NDRG1
Synapse_PPI_Communities	Syn-3_0_2312	301	ANXA1
Synapse_PPI_Communities	Syn-3_0_2312	25895	METTL21B
Synapse_PPI_Communities	Syn-3_0_2312	3301	DNAJA1
Synapse_PPI_Communities	Syn-3_0_2312	87	ACTN1
Synapse_PPI_Communities	Syn-3_0_2312	7295	TXN
Synapse_PPI_Communities	Syn-3_0_2312	10980	COPS6
Synapse_PPI_Communities	Syn-3_0_2312	7428	VHL
Synapse_PPI_Communities	Syn-3_0_2312	26260	FBXO25
Synapse_PPI_Communities	Syn-3_0_2312	8726	EED
Synapse_PPI_Communities	Syn-3_0_2312	5885	RAD21
Synapse_PPI_Communities	Syn-3_0_2312	9636	ISG15
Synapse_PPI_Communities	Syn-3_0_2312	3837	KPNB1
Synapse_PPI_Communities	Syn-3_0_2312	54434	SSH1
Synapse_PPI_Communities	Syn-3_0_2312	229	ALDOB
Synapse_PPI_Communities	Syn-3_0_2312	111	ADCY5

Synapse_PPI_Communities	Syn-3_0_2312	108	ADCY2
Synapse_PPI_Communities	Syn-3_0_2312	387	RHOA
Synapse_PPI_Communities	Syn-3_0_2312	7430	EZR
Synapse_PPI_Communities	Syn-3_0_2312	1609	DGKQ
Synapse_PPI_Communities	Syn-3_0_2312	10890	RAB10
Synapse_PPI_Communities	Syn-3_0_2312	94120	SYTL3
Synapse_PPI_Communities	Syn-3_0_2312	8899	PRPF4B
Synapse_PPI_Communities	Syn-3_0_2312	53916	RAB4B
Synapse_PPI_Communities	Syn-3_0_2312	55208	DCUN1D2
Synapse_PPI_Communities	Syn-3_0_2312	23542	MAPK8IP2
Synapse_PPI_Communities	Syn-3_0_2312	2257	FGF12
Synapse_PPI_Communities	Syn-3_0_2312	396	ARHGDIA
Synapse_PPI_Communities	Syn-3_0_2312	1967	EIF2B1
Synapse_PPI_Communities	Syn-3_0_2312	23474	ETHE1
Synapse_PPI_Communities	Syn-3_0_2312	256364	EML3
Synapse_PPI_Communities	Syn-3_0_2312	391634	HSP90AB2P
Synapse_PPI_Communities	Syn-3_0_2312	900	CCNG1
Synapse_PPI_Communities	Syn-3_0_2312	50852	TRAT1
Synapse_PPI_Communities	Syn-3_0_2312	51013	EXOSC1
Synapse_PPI_Communities	Syn-3_0_2312	84545	MRPL43
Synapse_PPI_Communities	Syn-3_0_2312	10165	SLC25A13
Synapse_PPI_Communities	Syn-3_0_2312	4035	LRP1
Synapse_PPI_Communities	Syn-3_0_2312	2157	F8
Synapse_PPI_Communities	Syn-3_0_2312	83451	ABHD11
Synapse_PPI_Communities	Syn-3_0_2312	3884	KRT33B
Synapse_PPI_Communities	Syn-3_0_2312	134492	NUDCD2
Synapse_PPI_Communities	Syn-3_0_2312	10526	IPO8
Synapse_PPI_Communities	Syn-3_0_2312	1562	CYP2C18
Synapse_PPI_Communities	Syn-3_0_2312	8078	USP5
Synapse_PPI_Communities	Syn-3_0_2312	9943	OXSR1
Synapse_PPI_Communities	Syn-3_0_2312	4831	NME2
Synapse_PPI_Communities	Syn-3_0_2312	5437	POLR2H
Synapse_PPI_Communities	Syn-3_0_2312	79711	IPO4
Synapse_PPI_Communities	Syn-3_0_2312	56990	CDC42SE2
Synapse_PPI_Communities	Syn-3_0_2312	2868	GRK4
Synapse_PPI_Communities	Syn-3_0_2312	3845	KRAS
Synapse_PPI_Communities	Syn-3_0_2312	8036	SHOC2
Synapse_PPI_Communities	Syn-3_0_2312	5592	PRKG1
Synapse_PPI_Communities	Syn-3_0_2312	7038	TG
Synapse_PPI_Communities	Syn-3_0_2312	55361	PI4K2A
Synapse_PPI_Communities	Syn-3_0_2312	1013	CDH15
Synapse_PPI_Communities	Syn-3_0_2312	56945	MRPS22
Synapse_PPI_Communities	Syn-3_0_2312	677	ZFP36L1
Synapse_PPI_Communities	Syn-3_0_2312	915	CD3D
Synapse_PPI_Communities	Syn-3_0_2312	25831	HECTD1
Synapse_PPI_Communities	Syn-3_0_2312	64708	COPS7B
Synapse_PPI_Communities	Syn-3_0_2312	777	CACNA1E
Synapse_PPI_Communities	Syn-3_0_2312	3500	IGHG1
Synapse_PPI_Communities	Syn-3_0_2312	2972	BRF1
Synapse_PPI_Communities	Syn-3_0_2312	1785	DNM2
Synapse_PPI_Communities	Syn-3_0_2312	79694	MANEA
Synapse_PPI_Communities	Syn-3_0_2312	79980	DSN1
Synapse_PPI_Communities	Syn-3_0_2312	9912	ARHGAP44
Synapse_PPI_Communities	Syn-3_0_2312	80852	GRIP2
Synapse_PPI_Communities	Syn-3_0_2312	95	ACY1
Synapse_PPI_Communities	Syn-3_0_2312	66005	CHID1
Synapse_PPI_Communities	Syn-3_0_2312	573	BAG1
Synapse_PPI_Communities	Syn-3_0_2312	8546	AP3B1

Synapse_PPI_Communities	Syn-3_0_2312	8943	AP3D1
Synapse_PPI_Communities	Syn-3_0_2312	10239	AP3S2
Synapse_PPI_Communities	Syn-3_0_2312	26292	MYCBP
Synapse_PPI_Communities	Syn-3_0_2312	26576	SRPK3
Synapse_PPI_Communities	Syn-3_0_2312	1119	CHKA
Synapse_PPI_Communities	Syn-3_0_2312	55037	PTCD3
Synapse_PPI_Communities	Syn-3_0_2312	5106	PCK2
Synapse_PPI_Communities	Syn-3_0_2312	50	ACO2
Synapse_PPI_Communities	Syn-3_0_2312	8907	AP1M1
Synapse_PPI_Communities	Syn-3_0_2312	162	AP1B1
Synapse_PPI_Communities	Syn-3_0_2312	8905	AP1S2
Synapse_PPI_Communities	Syn-3_0_2312	1174	AP1S1
Synapse_PPI_Communities	Syn-3_0_2312	64848	YTHDC2
Synapse_PPI_Communities	Syn-3_0_2312	51649	MRPS23
Synapse_PPI_Communities	Syn-3_0_2312	7019	TFAM
Synapse_PPI_Communities	Syn-3_0_2312	4643	MYO1E
Synapse_PPI_Communities	Syn-3_0_2312	5211	PFKL
Synapse_PPI_Communities	Syn-3_0_2312	51530	ZC3HC1
Synapse_PPI_Communities	Syn-3_0_2312	8976	WASL
Synapse_PPI_Communities	Syn-3_0_2312	27257	LSM1
Synapse_PPI_Communities	Syn-3_0_2312	60681	FKBP10
Synapse_PPI_Communities	Syn-3_0_2312	23168	RTF1
Synapse_PPI_Communities	Syn-3_0_2312	109	ADCY3
Synapse_PPI_Communities	Syn-3_0_2312	10295	BCKDK
Synapse_PPI_Communities	Syn-3_0_2312	28957	MRPS28
Synapse_PPI_Communities	Syn-3_0_2312	64432	MRPS25
Synapse_PPI_Communities	Syn-3_0_2312	79921	TCEAL4
Synapse_PPI_Communities	Syn-3_0_2312	23234	DNAJC9
Synapse_PPI_Communities	Syn-3_0_2312	10728	PTGES3
Synapse_PPI_Communities	Syn-3_0_2312	8601	RGS20
Synapse_PPI_Communities	Syn-3_0_2312	7381	UQCRB
Synapse_PPI_Communities	Syn-3_0_2312	6470	SHMT1
Synapse_PPI_Communities	Syn-3_0_2312	135644	TRIM40
Synapse_PPI_Communities	Syn-3_0_2312	5432	POLR2C
Synapse_PPI_Communities	Syn-3_0_2312	8339	HIST1H2BG
Synapse_PPI_Communities	Syn-3_0_2312	317772	HIST2H2AB
Synapse_PPI_Communities	Syn-3_0_2312	3013	HIST1H2AD
Synapse_PPI_Communities	Syn-3_0_2312	100505503	RPS17L
Synapse_PPI_Communities	Syn-3_0_2312	93380	MMGT1
Synapse_PPI_Communities	Syn-3_0_2312	5888	RAD51
Synapse_PPI_Communities	Syn-3_0_2312	3265	HRAS
Synapse_PPI_Communities	Syn-3_0_2312	8315	BRAP
Synapse_PPI_Communities	Syn-3_0_2312	10059	DNM1L
Synapse_PPI_Communities	Syn-3_0_2312	84262	PSMG3
Synapse_PPI_Communities	Syn-3_0_2312	219988	PATL1
Synapse_PPI_Communities	Syn-3_0_2312	81704	DOCK8
Synapse_PPI_Communities	Syn-3_0_2312	55336	FBXL8
Synapse_PPI_Communities	Syn-3_0_2312	2036	EPB41L1
Synapse_PPI_Communities	Syn-3_0_2312	5691	PSMB3
Synapse_PPI_Communities	Syn-3_0_2312	4733	DRG1
Synapse_PPI_Communities	Syn-3_0_2312	26097	CHTOP
Synapse_PPI_Communities	Syn-3_0_2312	9020	MAP3K14
Synapse_PPI_Communities	Syn-3_0_2312	5908	RAP1B
Synapse_PPI_Communities	Syn-3_0_2312	338382	RAB7B
Synapse_PPI_Communities	Syn-3_0_2312	1264	CNN1
Synapse_PPI_Communities	Syn-3_0_2312	6605	SMARCE1
Synapse_PPI_Communities	Syn-3_0_2312	56965	PARP6
Synapse_PPI_Communities	Syn-3_0_2312	23190	UBXN4

Synapse_PPI_Communities	Syn-3_0_2312	9344	TAOK2
Synapse_PPI_Communities	Syn-3_0_2312	10815	CPLX1
Synapse_PPI_Communities	Syn-3_0_2312	57446	NDRG3
Synapse_PPI_Communities	Syn-3_0_2312	5530	PPP3CA
Synapse_PPI_Communities	Syn-3_0_2312	4898	NRD1
Synapse_PPI_Communities	Syn-3_0_2312	6945	MLX
Synapse_PPI_Communities	Syn-3_0_2312	5356	PLRG1
Synapse_PPI_Communities	Syn-3_0_2312	4157	MC1R
Synapse_PPI_Communities	Syn-3_0_2312	10313	RTN3
Synapse_PPI_Communities	Syn-3_0_2312	57142	RTN4
Synapse_PPI_Communities	Syn-3_0_2312	10087	COL4A3BP
Synapse_PPI_Communities	Syn-3_0_2312	25923	ATL3
Synapse_PPI_Communities	Syn-3_0_2312	54541	DDIT4
Synapse_PPI_Communities	Syn-3_0_2312	6167	RPL37
Synapse_PPI_Communities	Syn-3_0_2312	23647	ARFIP2
Synapse_PPI_Communities	Syn-3_0_2312	23421	ITGB3BP
Synapse_PPI_Communities	Syn-3_0_2312	8623	ASMTL
Synapse_PPI_Communities	Syn-3_0_2312	80273	GRPEL1
Synapse_PPI_Communities	Syn-3_0_2312	29925	GMPPB
Synapse_PPI_Communities	Syn-3_0_2312	7388	UQCRH
Synapse_PPI_Communities	Syn-3_0_2312	6440	SFTPC
Synapse_PPI_Communities	Syn-3_0_2312	7030	TFE3
Synapse_PPI_Communities	Syn-3_0_2312	92856	IMP4
Synapse_PPI_Communities	Syn-3_0_2312	205	AK4
Synapse_PPI_Communities	Syn-3_0_2312	22826	DNAJC8
Synapse_PPI_Communities	Syn-3_0_2312	5345	SERPINF2
Synapse_PPI_Communities	Syn-3_0_2312	4709	NDUFB3
Synapse_PPI_Communities	Syn-3_0_2312	831	CAST
Synapse_PPI_Communities	Syn-3_0_2312	6456	SH3GL2
Synapse_PPI_Communities	Syn-3_0_2312	79364	ZXDC
Synapse_PPI_Communities	Syn-3_0_2312	11040	PIM2
Synapse_PPI_Communities	Syn-3_0_2312	27236	ARFIP1
Synapse_PPI_Communities	Syn-3_0_2312	10598	AHSA1
Synapse_PPI_Communities	Syn-3_0_2312	23612	PHLDA3
Synapse_PPI_Communities	Syn-3_0_2312	9135	RABEP1
Synapse_PPI_Communities	Syn-3_0_2312	55860	ACTR10
Synapse_PPI_Communities	Syn-3_0_2312	26284	ERAL1
Synapse_PPI_Communities	Syn-3_0_2312	5576	PRKAR2A
Synapse_PPI_Communities	Syn-3_0_2312	10564	ARFGEF2
Synapse_PPI_Communities	Syn-3_0_2312	9820	CUL7
Synapse_PPI_Communities	Syn-3_0_2312	5832	ALDH18A1
Synapse_PPI_Communities	Syn-3_0_2312	55561	CDC42BPG
Synapse_PPI_Communities	Syn-3_0_2312	2618	GART
Synapse_PPI_Communities	Syn-3_0_2312	201176	ARHGAP27
Synapse_PPI_Communities	Syn-3_0_2312	3853	KRT6A
Synapse_PPI_Communities	Syn-3_0_2312	10939	AFG3L2
Synapse_PPI_Communities	Syn-3_0_2312	9776	ATG13
Synapse_PPI_Communities	Syn-3_0_2312	10069	RWDD2B
Synapse_PPI_Communities	Syn-3_0_2312	5287	PIK3C2B
Synapse_PPI_Communities	Syn-3_0_2312	22930	RAB3GAP1
Synapse_PPI_Communities	Syn-3_0_2312	25782	RAB3GAP2
Synapse_PPI_Communities	Syn-3_0_2312	26073	POLDIP2
Synapse_PPI_Communities	Syn-3_0_2312	23191	CYFIP1
Synapse_PPI_Communities	Syn-3_0_2312	5238	PGM3
Synapse_PPI_Communities	Syn-3_0_2312	259	AMBP
Synapse_PPI_Communities	Syn-3_0_2312	8725	URI1
Synapse_PPI_Communities	Syn-3_0_2312	4793	NFKBIB
Synapse_PPI_Communities	Syn-3_0_2312	319101	KRT73

Synapse_PPI_Communities	Syn-3_0_2312	10248	POP7
Synapse_PPI_Communities	Syn-3_0_2312	11159	RABL2A
Synapse_PPI_Communities	Syn-3_0_2312	79930	DOK3
Synapse_PPI_Communities	Syn-3_0_2312	26263	FBXO22
Synapse_PPI_Communities	Syn-3_0_2312	54455	FBXO42
Synapse_PPI_Communities	Syn-3_0_2312	80028	FBXL18
Synapse_PPI_Communities	Syn-3_0_2312	54461	FBXW5
Synapse_PPI_Communities	Syn-3_0_2312	80204	FBXO11
Synapse_PPI_Communities	Syn-3_0_2312	90293	KLHL13
Synapse_PPI_Communities	Syn-3_0_2312	55692	LUC7L
Synapse_PPI_Communities	Syn-3_0_2312	2683	B4GALT1
Synapse_PPI_Communities	Syn-3_0_2312	50937	CDON
Synapse_PPI_Communities	Syn-3_0_2312	23395	LARS2
Synapse_PPI_Communities	Syn-3_0_2312	11269	DDX19B
Synapse_PPI_Communities	Syn-3_0_2312	1977	EIF4E
Synapse_PPI_Communities	Syn-3_0_2312	135114	HINT3
Synapse_PPI_Communities	Syn-3_0_2312	1436	CSF1R
Synapse_PPI_Communities	Syn-3_0_2312	80823	BHLHB9
Synapse_PPI_Communities	Syn-3_0_2312	51004	COQ6
Synapse_PPI_Communities	Syn-3_0_2312	10110	SGK2
Synapse_PPI_Communities	Syn-3_0_2312	2324	FLT4
Synapse_PPI_Communities	Syn-3_0_2312	79169	C1orf35
Synapse_PPI_Communities	Syn-3_0_2312	9448	MAP4K4
Synapse_PPI_Communities	Syn-3_0_2312	23678	SGK3
Synapse_PPI_Communities	Syn-3_0_2312	79858	NEK11
Synapse_PPI_Communities	Syn-3_0_2312	9615	GDA
Synapse_PPI_Communities	Syn-3_0_2312	2744	GLS
Synapse_PPI_Communities	Syn-3_0_2312	2799	GNS
Synapse_PPI_Communities	Syn-3_0_2312	3957	LGALS2
Synapse_PPI_Communities	Syn-3_0_2312	57508	INTS2
Synapse_PPI_Communities	Syn-3_0_2312	81545	FBXO38
Synapse_PPI_Communities	Syn-3_0_2312	5184	PEPD
Synapse_PPI_Communities	Syn-3_0_2312	26224	FBXL3
Synapse_PPI_Communities	Syn-3_0_2312	55308	DDX19A
Synapse_PPI_Communities	Syn-3_0_2312	9391	CIAO1
Synapse_PPI_Communities	Syn-3_0_2312	122704	MRPL52
Synapse_PPI_Communities	Syn-3_0_2312	6279	S100A8
Synapse_PPI_Communities	Syn-3_0_2312	51127	TRIM17
Synapse_PPI_Communities	Syn-3_0_2312	8209	C21orf33
Synapse_PPI_Communities	Syn-3_0_2312	23034	SAMD4A
Synapse_PPI_Communities	Syn-3_0_2312	115290	FBXO17
Synapse_PPI_Communities	Syn-3_0_2312	126433	FBXO27
Synapse_PPI_Communities	Syn-3_0_2312	9462	RASAL2
Synapse_PPI_Communities	Syn-3_0_2312	84861	KLHL22
Synapse_PPI_Communities	Syn-3_0_2312	114792	KLHL32
Synapse_PPI_Communities	Syn-3_0_2312	122786	FRMD6
Synapse_PPI_Communities	Syn-3_0_2312	83732	RIOK1
Synapse_PPI_Communities	Syn-3_0_2312	57679	ALS2
Synapse_PPI_Communities	Syn-3_0_2312	26272	FBXO4
Synapse_PPI_Communities	Syn-3_0_2312	55213	RCBTB1
Synapse_PPI_Communities	Syn-3_0_2312	9306	SOCS6
Synapse_PPI_Communities	Syn-3_0_2312	10436	EMG1
Synapse_PPI_Communities	Syn-3_0_2312	54778	RNF111
Synapse_PPI_Communities	Syn-3_0_2312	64951	MRPS24
Synapse_PPI_Communities	Syn-3_0_2312	57645	POGK
Synapse_PPI_Communities	Syn-3_0_2312	56144	PCDHA4
Synapse_PPI_Communities	Syn-3_0_2312	51056	LAP3
Synapse_PPI_Communities	Syn-3_0_2312	50945	TBX22

Synapse_PPI_Communities	Syn-3_0_2312	56987	BBX
Synapse_PPI_Communities	Syn-3_0_2312	23532	PRAME
Synapse_PPI_Communities	Syn-3_0_2312	51078	THAP4
Synapse_PPI_Communities	Syn-3_0_2312	5935	RBM3
Synapse_PPI_Communities	Syn-3_0_2312	81788	NUAK2
Synapse_PPI_Communities	Syn-3_0_2312	84172	POLR1B
Synapse_PPI_Communities	Syn-3_0_2312	55655	NLRP2
Synapse_PPI_Communities	Syn-3_0_2312	79012	CAMKV
Synapse_PPI_Communities	Syn-3_0_2312	5429	POLH
Synapse_PPI_Communities	Syn-3_0_2312	5753	PTK6
Synapse_PPI_Communities	Syn-3_0_2312	1163	CKS1B
Synapse_PPI_Communities	Syn-3_0_2312	11315	PARK7
Synapse_PPI_Communities	Syn-3_0_2312	6620	SNCB
Synapse_PPI_Communities	Syn-3_0_2312	1263	PLK3
Synapse_PPI_Communities	Syn-3_0_2312	11333	PDAP1
Synapse_PPI_Communities	Syn-3_0_2312	1612	DAPK1
Synapse_PPI_Communities	Syn-3_0_2312	103910	MYL12B
Synapse_PPI_Communities	Syn-3_0_2312	84950	PRPF38A
Synapse_PPI_Communities	Syn-3_0_2312	27248	ERLEC1
Synapse_PPI_Communities	Syn-3_0_2312	4830	NME1
Synapse_PPI_Communities	Syn-3_0_2312	2224	FDPS
Synapse_PPI_Communities	Syn-3_0_2312	23221	RHOBTB2
Synapse_PPI_Communities	Syn-3_0_2312	2902	GRIN1
Synapse_PPI_Communities	Syn-3_0_2312	116444	GRIN3B
Synapse_PPI_Communities	Syn-3_0_2312	4593	MUSK
Synapse_PPI_Communities	Syn-3_0_2312	60489	APOBEC3G
Synapse_PPI_Communities	Syn-3_0_2312	55781	RIOK2
Synapse_PPI_Communities	Syn-3_0_2312	10549	PRDX4
Synapse_PPI_Communities	Syn-3_0_2312	7352	UCP3
Synapse_PPI_Communities	Syn-3_0_2312	11279	KLF8
Synapse_PPI_Communities	Syn-3_0_2312	282974	STK32C
Synapse_PPI_Communities	Syn-3_0_2312	152503	SH3D19
Synapse_PPI_Communities	Syn-3_0_2312	1739	DLG1
Synapse_PPI_Communities	Syn-3_0_2312	3759	KCNJ2
Synapse_PPI_Communities	Syn-3_0_2312	65268	WNK2
Synapse_PPI_Communities	Syn-3_0_2312	64784	CRTC3
Synapse_PPI_Communities	Syn-3_0_2312	55835	CENPJ
Synapse_PPI_Communities	Syn-3_0_2312	10672	GNA13
Synapse_PPI_Communities	Syn-3_0_2312	10566	AKAP3
Synapse_PPI_Communities	Syn-3_0_2312	140809	SRXN1
Synapse_PPI_Communities	Syn-3_0_2312	11346	SYNPO
Synapse_PPI_Communities	Syn-3_0_2312	4313	MMP2
Synapse_PPI_Communities	Syn-3_0_2312	4023	LPL
Synapse_PPI_Communities	Syn-3_0_2312	10198	MPHOSPH9
Synapse_PPI_Communities	Syn-3_0_2312	8888	MCM3AP
Synapse_PPI_Communities	Syn-3_0_2312	8853	ASAP2
Synapse_PPI_Communities	Syn-3_0_2312	6760	SS18
Synapse_PPI_Communities	Syn-3_0_2312	81608	FIP1L1
Synapse_PPI_Communities	Syn-3_0_2312	5774	PTPN3
Synapse_PPI_Communities	Syn-3_0_2312	63943	FKBPL
Synapse_PPI_Communities	Syn-3_0_2312	2308	FOXO1
Synapse_PPI_Communities	Syn-3_0_2312	8449	DHX16
Synapse_PPI_Communities	Syn-3_0_2312	4833	NME4
Synapse_PPI_Communities	Syn-3_0_2312	196441	ZFC3H1
Synapse_PPI_Communities	Syn-3_0_2312	10742	RAI2
Synapse_PPI_Communities	Syn-3_0_2312	79982	DNAJB14
Synapse_PPI_Communities	Syn-3_0_2312	730211	HSP90AA5P
Synapse_PPI_Communities	Syn-3_0_2312	23786	BCL2L13

Synapse_PPI_Communities	Syn-3_0_2312	57018	CCNL1
Synapse_PPI_Communities	Syn-3_0_2312	912	CD1D
Synapse_PPI_Communities	Syn-3_0_2312	5582	PRKCG
Synapse_PPI_Communities	Syn-3_0_2312	2557	GABRA4
Synapse_PPI_Communities	Syn-3_0_2312	7166	TPH1
Synapse_PPI_Communities	Syn-3_0_2312	25864	ABHD14A
Synapse_PPI_Communities	Syn-3_0_2312	7818	DAP3
Synapse_PPI_Communities	Syn-3_0_2312	2870	GRK6
Synapse_PPI_Communities	Syn-3_0_2312	6011	GRK1
Synapse_PPI_Communities	Syn-3_0_2312	114034	TOE1
Synapse_PPI_Communities	Syn-3_0_2312	51021	MRPS16
Synapse_PPI_Communities	Syn-3_0_2312	9915	ARNT2
Synapse_PPI_Communities	Syn-3_0_2312	1007	CDH9
Synapse_PPI_Communities	Syn-3_0_2312	728	C5AR1
Synapse_PPI_Communities	Syn-3_0_2312	56905	C15orf39
Synapse_PPI_Communities	Syn-3_0_2312	55929	DMAP1
Synapse_PPI_Communities	Syn-3_0_2312	23621	BACE1
Synapse_PPI_Communities	Syn-3_0_2312	350	APOH
Synapse_PPI_Communities	Syn-3_0_2312	80311	KLHL15
Synapse_PPI_Communities	Syn-3_0_2312	22882	ZHX2
Synapse_PPI_Communities	Syn-3_0_2312	51191	HERC5
Synapse_PPI_Communities	Syn-3_0_2312	10325	RRAGB
Synapse_PPI_Communities	Syn-3_0_2312	51503	CWC15
Synapse_PPI_Communities	Syn-3_0_2312	84916	CIRH1A
Synapse_PPI_Communities	Syn-3_0_2312	94274	PPP1R14A
Synapse_PPI_Communities	Syn-3_0_2312	1454	CSNK1E
Synapse_PPI_Communities	Syn-3_0_2312	11104	KATNA1
Synapse_PPI_Communities	Syn-3_0_2312	5578	PRKCA
Synapse_PPI_Communities	Syn-3_0_2312	23513	SCRIB
Synapse_PPI_Communities	Syn-3_0_2312	51322	WAC
Synapse_PPI_Communities	Syn-3_0_2312	790	CAD
Synapse_PPI_Communities	Syn-3_0_2312	4133	MAP2
Synapse_PPI_Communities	Syn-3_0_2312	5719	PSMD13
Synapse_PPI_Communities	Syn-3_0_2312	23243	ANKRD28
Synapse_PPI_Communities	Syn-3_0_2312	3148	HMGB2
Synapse_PPI_Communities	Syn-3_0_2312	8507	ENC1
Synapse_PPI_Communities	Syn-3_0_2312	23071	ERP44
Synapse_PPI_Communities	Syn-3_0_2312	6732	SRPK1
Synapse_PPI_Communities	Syn-3_0_2312	782	CACNB1
Synapse_PPI_Communities	Syn-3_0_2312	84106	PRAM1
Synapse_PPI_Communities	Syn-3_0_2312	2193	FARSA
Synapse_PPI_Communities	Syn-3_0_2312	147841	SPC24
Synapse_PPI_Communities	Syn-3_0_2312	2181	ACSL3
Synapse_PPI_Communities	Syn-3_0_2312	2182	ACSL4
Synapse_PPI_Communities	Syn-3_0_2312	10972	TMED10
Synapse_PPI_Communities	Syn-3_0_2312	140461	ASB8
Synapse_PPI_Communities	Syn-3_0_2312	11168	PSIP1
Synapse_PPI_Communities	Syn-3_0_2312	8407	TAGLN2
Synapse_PPI_Communities	Syn-3_0_2312	10286	BCAS2
Synapse_PPI_Communities	Syn-3_0_2312	10049	DNAJB6
Synapse_PPI_Communities	Syn-3_0_2312	57606	SLAIN2
Synapse_PPI_Communities	Syn-3_0_2312	65108	MARCKSL1
Synapse_PPI_Communities	Syn-3_0_2312	26589	MRPL46
Synapse_PPI_Communities	Syn-3_0_2312	56922	MCCC1
Synapse_PPI_Communities	Syn-3_0_2312	3306	HSPA2
Synapse_PPI_Communities	Syn-3_0_2312	2026	ENO2
Synapse_PPI_Communities	Syn-3_0_2312	4860	PNP
Synapse_PPI_Communities	Syn-3_0_2312	10254	STAM2

Synapse_PPI_Communities	Syn-3_0_2312	22950	SLC4A1AP
Synapse_PPI_Communities	Syn-3_0_2312	5756	TWF1
Synapse_PPI_Communities	Syn-3_0_2312	54534	MRPL50
Synapse_PPI_Communities	Syn-3_0_2312	85451	UNK
Synapse_PPI_Communities	Syn-3_0_2312	26276	VPS33B
Synapse_PPI_Communities	Syn-3_0_2312	51699	VPS29
Synapse_PPI_Communities	Syn-3_0_2312	55705	IPO9
Synapse_PPI_Communities	Syn-3_0_2312	9948	WDR1
Synapse_PPI_Communities	Syn-3_0_2312	3093	UBE2K
Synapse_PPI_Communities	Syn-3_0_2312	56658	TRIM39
Synapse_PPI_Communities	Syn-3_0_2312	9939	RBM8A
Synapse_PPI_Communities	Syn-3_0_2312	11316	COPE
Synapse_PPI_Communities	Syn-3_0_2312	9701	PPP6R2
Synapse_PPI_Communities	Syn-3_0_2312	1155	TBCB
Synapse_PPI_Communities	Syn-3_0_2312	4791	NFKB2
Synapse_PPI_Communities	Syn-3_0_2312	22858	ICK
Synapse_PPI_Communities	Syn-3_0_2312	29899	GPSM2
Synapse_PPI_Communities	Syn-3_0_2312	79657	RPAP3
Synapse_PPI_Communities	Syn-3_0_2312	92591	ASB16
Synapse_PPI_Communities	Syn-3_0_2312	5514	PPP1R10
Synapse_PPI_Communities	Syn-3_0_2312	5693	PSMB5
Synapse_PPI_Communities	Syn-3_0_2312	6907	TBL1X
Synapse_PPI_Communities	Syn-3_0_2312	91653	BOC
Synapse_PPI_Communities	Syn-3_0_2312	4773	NFATC2
Synapse_PPI_Communities	Syn-3_0_2312	2047	EPHB1
Synapse_PPI_Communities	Syn-3_0_2312	10762	NUP50
Synapse_PPI_Communities	Syn-3_0_2312	57658	CALCOCO1
Synapse_PPI_Communities	Syn-3_0_2312	5607	MAP2K5
Synapse_PPI_Communities	Syn-3_0_2312	80279	CDK5RAP3
Synapse_PPI_Communities	Syn-3_0_2312	4645	MYO5B
Synapse_PPI_Communities	Syn-3_0_2312	25827	FBXL2
Synapse_PPI_Communities	Syn-3_0_2312	8139	GAN
Synapse_PPI_Communities	Syn-3_0_2312	378884	NHLRC1
Synapse_PPI_Communities	Syn-3_0_2312	84759	PCGF1
Synapse_PPI_Communities	Syn-3_0_2312	3001	GZMA
Synapse_PPI_Communities	Syn-3_0_2312	10062	NR1H3
Synapse_PPI_Communities	Syn-3_0_2312	55643	BTBD2
Synapse_PPI_Communities	Syn-3_0_2312	1164	CKS2
Synapse_PPI_Communities	Syn-3_0_2312	5588	PRKCQ
Synapse_PPI_Communities	Syn-3_0_2312	57172	CAMK1G
Synapse_PPI_Communities	Syn-3_0_2312	8030	CCDC6
Synapse_PPI_Communities	Syn-3_0_2312	23705	CADM1
Synapse_PPI_Communities	Syn-3_0_2312	23136	EPB41L3
Synapse_PPI_Communities	Syn-3_0_2312	534	ATP6V1G2
Synapse_PPI_Communities	Syn-3_0_2312	1073	CFL2
Synapse_PPI_Communities	Syn-3_0_2312	9363	RAB33A
Synapse_PPI_Communities	Syn-3_0_2312	79666	PLEKHF2
Synapse_PPI_Communities	Syn-3_0_2312	11135	CDC42EP1
Synapse_PPI_Communities	Syn-3_0_2312	7050	TGIF1
Synapse_PPI_Communities	Syn-3_0_2312	991	CDC20
Synapse_PPI_Communities	Syn-3_0_2312	57596	BEGAIN
Synapse_PPI_Communities	Syn-3_0_2312	10474	TADA3
Synapse_PPI_Communities	Syn-3_0_2312	285527	FRYL
Synapse_PPI_Communities	Syn-3_0_2312	200186	CRTC2
Synapse_PPI_Communities	Syn-3_0_2312	23164	MPRIP
Synapse_PPI_Communities	Syn-3_0_2312	5298	PI4KB
Synapse_PPI_Communities	Syn-3_0_2312	9470	EIF4E2
Synapse_PPI_Communities	Syn-3_0_2312	10128	LRPPRC

Synapse_PPI_Communities	Syn-3_0_2312	3891	KRT85
Synapse_PPI_Communities	Syn-3_0_2312	23294	ANKS1A
Synapse_PPI_Communities	Syn-3_0_2312	55161	TMEM33
Synapse_PPI_Communities	Syn-3_0_2312	57530	CGN
Synapse_PPI_Communities	Syn-3_0_2312	8795	TNFRSF10B
Synapse_PPI_Communities	Syn-3_0_2312	843	CASP10
Synapse_PPI_Communities	Syn-3_0_2312	1001	CDH3
Synapse_PPI_Communities	Syn-3_0_2312	91833	WDR20
Synapse_PPI_Communities	Syn-3_0_2312	22927	HABP4
Synapse_PPI_Communities	Syn-3_0_2312	4637	MYL6
Synapse_PPI_Communities	Syn-3_0_2312	79784	MYH14
Synapse_PPI_Communities	Syn-3_0_2312	10398	MYL9
Synapse_PPI_Communities	Syn-3_0_2312	116986	AGAP2
Synapse_PPI_Communities	Syn-3_0_2312	54910	SEMA4C
Synapse_PPI_Communities	Syn-3_0_2312	10567	RABAC1
Synapse_PPI_Communities	Syn-3_0_2312	79890	RIN3
Synapse_PPI_Communities	Syn-3_0_2312	5869	RAB5B
Synapse_PPI_Communities	Syn-3_0_2312	54212	SNTG1
Synapse_PPI_Communities	Syn-3_0_2312	6814	STXBP3
Synapse_PPI_Communities	Syn-3_0_2312	26985	AP3M1
Synapse_PPI_Communities	Syn-3_0_2312	9371	KIF3B
Synapse_PPI_Communities	Syn-3_0_2312	815	CAMK2A
Synapse_PPI_Communities	Syn-3_0_2312	3746	KCNC1
Synapse_PPI_Communities	Syn-3_0_2312	2596	GAP43
Synapse_PPI_Communities	Syn-3_0_2312	842	CASP9
Synapse_PPI_Communities	Syn-3_0_2312	9465	AKAP7
Synapse_PPI_Communities	Syn-3_0_2312	9472	AKAP6
Synapse_PPI_Communities	Syn-3_0_2312	933	CD22
Synapse_PPI_Communities	Syn-3_0_2312	2247	FGF2
Synapse_PPI_Communities	Syn-3_0_2312	28954	REM1
Synapse_PPI_Communities	Syn-3_0_2312	64130	LIN7B
Synapse_PPI_Communities	Syn-3_0_2312	3752	KCND3
Synapse_PPI_Communities	Syn-3_0_2312	55294	FBXW7
Synapse_PPI_Communities	Syn-3_0_2312	2788	GNG7
Synapse_PPI_Communities	Syn-3_0_2312	2784	GNB3
Synapse_PPI_Communities	Syn-3_0_2312	2246	FGF1
Synapse_PPI_Communities	Syn-3_0_2312	6284	S100A13
Synapse_PPI_Communities	Syn-3_0_2312	3915	LAMC1
Synapse_PPI_Communities	Syn-3_0_2312	3150	HMGN1
Synapse_PPI_Communities	Syn-3_0_2312	3762	KCNJ5
Synapse_PPI_Communities	Syn-3_0_2312	1815	DRD4
Synapse_PPI_Communities	Syn-3_0_2312	11337	GABARAP
Synapse_PPI_Communities	Syn-3_0_2312	2079	ERH
Synapse_PPI_Communities	Syn-3_0_2312	2776	GNAQ
Synapse_PPI_Communities	Syn-3_0_2312	2917	GRM7
Synapse_PPI_Communities	Syn-3_0_2312	5159	PDGFRB
Synapse_PPI_Communities	Syn-3_0_2312	4744	NEFH
Synapse_PPI_Communities	Syn-3_0_2312	4747	NEFL
Synapse_PPI_Communities	Syn-3_0_2312	5327	PLAT
Synapse_PPI_Communities	Syn-3_0_2312	11184	MAP4K1
Synapse_PPI_Communities	Syn-3_0_2312	1813	DRD2
Synapse_PPI_Communities	Syn-3_0_2312	56882	CDC42SE1
Synapse_PPI_Communities	Syn-3_0_2312	6285	S100B
Synapse_PPI_Communities	Syn-3_0_2312	10527	IPO7
Synapse_PPI_Communities	Syn-3_0_2312	1901	S1PR1
Synapse_PPI_Communities	Syn-3_0_2312	23770	FKBP8
Synapse_PPI_Communities	Syn-3_0_2312	9900	SV2A
Synapse_PPI_Communities	Syn-3_0_2312	10140	TOB1

Synapse_PPI_Communities	Syn-3_0_2312	5979	RET
Synapse_PPI_Communities	Syn-3_0_2312	143187	VTI1A
Synapse_PPI_Communities	Syn-3_0_2312	8675	STX16
Synapse_PPI_Communities	Syn-3_0_2312	4983	OPHN1
Synapse_PPI_Communities	Syn-3_0_2312	3107	HLA-C
Synapse_PPI_Communities	Syn-3_0_2312	10912	GADD45G
Synapse_PPI_Communities	Syn-3_0_2312	4916	NTRK3
Synapse_PPI_Communities	Syn-3_0_2312	23022	PALLD
Synapse_PPI_Communities	Syn-3_0_2312	2322	FLT3
Synapse_PPI_Communities	Syn-3_0_2312	92799	SHKBP1
Synapse_PPI_Communities	Syn-3_0_2312	7253	TSHR
Synapse_PPI_Communities	Syn-3_0_2312	6093	ROCK1
Synapse_PPI_Communities	Syn-3_0_2312	3985	LIMK2
Synapse_PPI_Communities	Syn-3_0_2312	80333	KCNIP4
Synapse_PPI_Communities	Syn-3_0_2312	5602	MAPK10
Synapse_PPI_Communities	Syn-3_0_2312	65975	STK33
Synapse_PPI_Communities	Syn-3_0_2312	2065	ERBB3
Synapse_PPI_Communities	Syn-3_0_2312	9419	CRIPT
Synapse_PPI_Communities	Syn-3_0_2312	115557	ARHGEF25
Synapse_PPI_Communities	Syn-3_0_2312	79039	DDX54
Synapse_PPI_Communities	Syn-3_0_2312	2120	ETV6
Synapse_PPI_Communities	Syn-3_0_2312	7336	UBE2V2
Synapse_PPI_Communities	Syn-3_0_2312	9736	USP34
Synapse_PPI_Communities	Syn-3_0_2312	9126	SMC3
Synapse_PPI_Communities	Syn-3_0_2312	85377	MICALL1
Synapse_PPI_Communities	Syn-3_0_2312	55159	RFWD3
Synapse_PPI_Communities	Syn-3_0_2312	9546	APBA3
Synapse_PPI_Communities	Syn-3_0_2312	10010	TANK
Synapse_PPI_Communities	Syn-3_0_2312	9817	KEAP1
Synapse_PPI_Communities	Syn-3_0_2312	10135	NAMPT
Synapse_PPI_Communities	Syn-3_0_2312	3553	IL1B
Synapse_PPI_Communities	Syn-3_0_2312	3097	HIVEP2
Synapse_PPI_Communities	Syn-3_0_2312	3937	LCP2
Synapse_PPI_Communities	Syn-3_0_2312	6744	SSFA2
Synapse_PPI_Communities	Syn-3_0_2312	7812	CSDE1
Synapse_PPI_Communities	Syn-3_0_2312	9750	FAM65B
Synapse_PPI_Communities	Syn-3_0_2312	25937	WWTR1
Synapse_PPI_Communities	Syn-3_0_2312	51306	FAM13B
Synapse_PPI_Communities	Syn-3_0_2312	51307	FAM53C
Synapse_PPI_Communities	Syn-3_0_2312	55167	MSL2
Synapse_PPI_Communities	Syn-3_0_2312	55778	ZNF839
Synapse_PPI_Communities	Syn-3_0_2312	84952	CGNL1
Synapse_PPI_Communities	Syn-3_0_2312	100462977	MTRNR2L1
Synapse_PPI_Communities	Syn-3_0_2312	7351	UCP2
Synapse_PPI_Communities	Syn-3_0_2312	467	ATF3
Synapse_PPI_Communities	Syn-3_0_2312	55596	ZCCHC8
Synapse_PPI_Communities	Syn-3_0_2312	8330	HIST1H2AK
Synapse_PPI_Communities	Syn-3_0_2312	4356	MPP3
Synapse_PPI_Communities	Syn-3_0_2312	114609	TIRAP
Synapse_PPI_Communities	Syn-3_0_2312	64802	NMNAT1
Synapse_PPI_Communities	Syn-3_0_2312	85015	USP45
Synapse_PPI_Communities	Syn-3_0_2312	5695	PSMB7
Synapse_PPI_Communities	Syn-3_0_2312	51599	LSR
Synapse_PPI_Communities	Syn-3_0_2312	79084	WDR77
Synapse_PPI_Communities	Syn-3_0_2312	29896	TRA2A
Synapse_PPI_Communities	Syn-3_0_2312	5743	PTGS2
Synapse_PPI_Communities	Syn-3_0_2312	124739	USP43
Synapse_PPI_Communities	Syn-3_0_2312	23216	TBC1D1

Synapse_PPI_Communities	Syn-3_0_2312	26268	FBXO9
Synapse_PPI_Communities	Syn-3_0_2312	89796	NAV1
Synapse_PPI_Communities	Syn-3_0_2312	4772	NFATC1
Synapse_PPI_Communities	Syn-3_0_2312	2961	GTF2E2
Synapse_PPI_Communities	Syn-3_0_2312	23759	PPIL2
Synapse_PPI_Communities	Syn-3_0_2312	5335	PLCG1
Synapse_PPI_Communities	Syn-3_0_2312	5834	PYGB
Synapse_PPI_Communities	Syn-3_0_2312	55148	UBR7
Synapse_PPI_Communities	Syn-3_0_2312	197	AHSG
Synapse_PPI_Communities	Syn-3_0_2312	326	AIRE
Synapse_PPI_Communities	Syn-3_0_2312	10749	KIF1C
Synapse_PPI_Communities	Syn-3_0_2312	83933	HDAC10
Synapse_PPI_Communities	Syn-3_0_2312	5265	SERPINA1
Synapse_PPI_Communities	Syn-3_0_2312	6242	RTKN
Synapse_PPI_Communities	Syn-3_0_2312	27230	SERP1
Synapse_PPI_Communities	Syn-3_0_2312	7704	ZBTB16
Synapse_PPI_Communities	Syn-3_0_2312	26230	TIAM2
Synapse_PPI_Communities	Syn-3_0_2312	51042	ZNF593
Synapse_PPI_Communities	Syn-3_0_2312	3507	IGHM
Synapse_PPI_Communities	Syn-3_0_2312	199731	CADM4
Synapse_PPI_Communities	Syn-3_0_2312	440	ASNS
Synapse_PPI_Communities	Syn-3_0_2312	7905	REEP5
Synapse_PPI_Communities	Syn-3_0_2312	26002	MOXD1
Synapse_PPI_Communities	Syn-3_0_2312	2806	GOT2
Synapse_PPI_Communities	Syn-3_0_2312	23649	POLA2
Synapse_PPI_Communities	Syn-3_0_2312	9189	ZBED1
Synapse_PPI_Communities	Syn-3_0_2312	8833	GMPS
Synapse_PPI_Communities	Syn-3_0_2312	3416	IDE
Synapse_PPI_Communities	Syn-3_0_2312	9818	NUPL1
Synapse_PPI_Communities	Syn-3_0_2312	23476	BRD4
Synapse_PPI_Communities	Syn-3_0_2312	92483	LDHAL6B
Synapse_PPI_Communities	Syn-3_0_2312	5292	PIM1
Synapse_PPI_Communities	Syn-3_0_2312	4144	MAT2A
Synapse_PPI_Communities	Syn-3_0_2312	9099	USP2
Synapse_PPI_Communities	Syn-3_0_2312	9644	SH3PXD2A
Synapse_PPI_Communities	Syn-3_0_2312	22872	SEC31A
Synapse_PPI_Communities	Syn-3_0_2312	654364	NME1-NME2
Synapse_PPI_Communities	Syn-3_0_2312	10422	UBAC1
Synapse_PPI_Communities	Syn-3_0_2312	10808	HSPH1
Synapse_PPI_Communities	Syn-3_0_2312	5496	PPM1G
Synapse_PPI_Communities	Syn-3_0_2312	10146	G3BP1
Synapse_PPI_Communities	Syn-3_0_2312	9584	RBM39
Synapse_PPI_Communities	Syn-3_0_2312	10929	SRSF8
Synapse_PPI_Communities	Syn-3_0_2312	6164	RPL34
Synapse_PPI_Communities	Syn-3_0_2312	3615	IMPDH2
Synapse_PPI_Communities	Syn-3_0_2312	23061	TBC1D9B
Synapse_PPI_Communities	Syn-3_0_2312	1808	DPYSL2
Synapse_PPI_Communities	Syn-3_0_2312	54517	PUS7
Synapse_PPI_Communities	Syn-3_0_2312	56899	ANKS1B
Synapse_PPI_Communities	Syn-3_0_2312	5931	RBBP7
Synapse_PPI_Communities	Syn-3_0_2312	58490	RPRD1B
Synapse_PPI_Communities	Syn-3_0_2312	94239	H2AFV
Synapse_PPI_Communities	Syn-3_0_2312	255626	HIST1H2BA
Synapse_PPI_Communities	Syn-3_0_2312	51540	SCLY
Synapse_PPI_Communities	Syn-3_0_2312	23075	SWAP70
Synapse_PPI_Communities	Syn-3_0_2312	65993	MRPS34
Synapse_PPI_Communities	Syn-3_0_2312	9128	PRPF4
Synapse_PPI_Communities	Syn-3_0_2312	6904	TBCD

Synapse_PPI_Communities	Syn-3_0_2312	51081	MRPS7
Synapse_PPI_Communities	Syn-3_0_2312	3836	KPNA1
Synapse_PPI_Communities	Syn-3_0_2312	84309	NUDT16L1
Synapse_PPI_Communities	Syn-3_0_2312	23645	PPP1R15A
Synapse_PPI_Communities	Syn-3_0_2312	22909	FAN1
Synapse_PPI_Communities	Syn-3_0_2312	10138	YAF2
Synapse_PPI_Communities	Syn-3_0_2312	5813	PURA
Synapse_PPI_Communities	Syn-3_0_2312	27332	ZNF638
Synapse_PPI_Communities	Syn-3_0_2312	64949	MRPS26
Synapse_PPI_Communities	Syn-3_0_2312	56342	PPAN
Synapse_PPI_Communities	Syn-3_0_2312	23107	MRPS27
Synapse_PPI_Communities	Syn-3_0_2312	9801	MRPL19
Synapse_PPI_Communities	Syn-3_0_2312	7307	U2AF1
Synapse_PPI_Communities	Syn-3_0_2312	26090	ABHD12
Synapse_PPI_Communities	Syn-3_0_2312	9184	BUB3
Synapse_PPI_Communities	Syn-3_0_2312	80185	TTI2
Synapse_PPI_Communities	Syn-3_0_2312	10016	PDCD6
Synapse_PPI_Communities	Syn-3_0_2312	6182	MRPL12
Synapse_PPI_Communities	Syn-3_0_2312	8106	PABPN1
Synapse_PPI_Communities	Syn-3_0_2312	3959	LGALS3BP
Synapse_PPI_Communities	Syn-3_0_2312	5328	PLAU
Synapse_PPI_Communities	Syn-3_0_2312	56829	ZC3HAV1
Synapse_PPI_Communities	Syn-3_0_2312	8566	PDXK
Synapse_PPI_Communities	Syn-3_0_2312	2926	GRSF1
Synapse_PPI_Communities	Syn-3_0_2312	6432	SRSF7
Synapse_PPI_Communities	Syn-3_0_2312	3311	HSPA7
Synapse_PPI_Communities	Syn-3_0_2312	79077	DCTPP1
Synapse_PPI_Communities	Syn-3_0_2312	78994	PRR14
Synapse_PPI_Communities	Syn-3_0_2312	55707	NECAP2
Synapse_PPI_Communities	Syn-3_0_2312	54431	DNAJC10
Synapse_PPI_Communities	Syn-3_0_2312	6812	STXBP1
Synapse_PPI_Communities	Syn-3_0_2312	94121	SYTL4
Synapse_PPI_Communities	Syn-3_0_2312	5874	RAB27B
Synapse_PPI_Communities	Syn-3_0_2312	9495	AKAP5
Synapse_PPI_Communities	Syn-3_0_2312	2562	GABRB3
Synapse_PPI_Communities	Syn-3_0_2312	2775	GNAO1
Synapse_PPI_Communities	Syn-3_0_2312	4985	OPRD1
Synapse_PPI_Communities	Syn-3_0_2312	4988	OPRM1
Synapse_PPI_Communities	Syn-3_0_2312	84675	TRIM55
Synapse_PPI_Communities	Syn-3_0_2312	9564	BCAR1
Synapse_PPI_Communities	Syn-3_0_2312	2983	GUCY1B3
Synapse_PPI_Communities	Syn-3_0_2312	3738	KCNA3
Synapse_PPI_Communities	Syn-3_0_2312	3736	KCNA1
Synapse_PPI_Communities	Syn-3_0_2312	3737	KCNA2
Synapse_PPI_Communities	Syn-3_0_2312	3739	KCNA4
Synapse_PPI_Communities	Syn-3_0_2312	10928	RALBP1
Synapse_PPI_Communities	Syn-3_0_2312	1677	DFFB
Synapse_PPI_Communities	Syn-3_0_2312	3956	LGALS1
Synapse_PPI_Communities	Syn-3_0_2312	5792	PTPRF
Synapse_PPI_Communities	Syn-3_0_2312	9454	HOMER3
Synapse_PPI_Communities	Syn-3_0_2312	4216	MAP3K4
Synapse_PPI_Communities	Syn-3_0_2312	23291	FBXW11
Synapse_PPI_Communities	Syn-3_0_2312	5682	PSMA1
Synapse_PPI_Communities	Syn-3_0_2312	221092	HNRNPUL2
Synapse_PPI_Communities	Syn-3_0_2312	10043	TOM1
Synapse_PPI_Communities	Syn-3_0_2312	83940	TATDN1
Synapse_PPI_Communities	Syn-3_0_2312	51366	UBR5
Synapse_PPI_Communities	Syn-3_0_2312	84676	TRIM63

Synapse_PPI_Communities	Syn-3_0_2312	7094	TLN1
Synapse_PPI_Communities	Syn-3_0_2312	7141	TNP1
Synapse_PPI_Communities	Syn-3_0_2312	6050	RNH1
Synapse_PPI_Communities	Syn-3_0_2312	10498	CARM1
Synapse_PPI_Communities	Syn-3_0_2312	9530	BAG4
Synapse_PPI_Communities	Syn-3_0_2312	51003	MED31
Synapse_PPI_Communities	Syn-3_0_2312	8676	STX11
Synapse_PPI_Communities	Syn-3_0_2312	8683	SRSF9
Synapse_PPI_Communities	Syn-3_0_2312	537	ATP6AP1
Synapse_PPI_Communities	Syn-3_0_2312	29789	OLA1
Synapse_PPI_Communities	Syn-3_0_2312	83983	TSSK6
Synapse_PPI_Communities	Syn-3_0_2312	55621	TRMT1
Synapse_PPI_Communities	Syn-3_0_2312	27072	VPS41
Synapse_PPI_Communities	Syn-3_0_2312	5495	PPM1B
Synapse_PPI_Communities	Syn-3_0_2312	4082	MARCKS
Synapse_PPI_Communities	Syn-3_0_2312	29978	UBQLN2
Synapse_PPI_Communities	Syn-3_0_2312	84617	TUBB6
Synapse_PPI_Communities	Syn-3_0_2312	6597	SMARCA4
Synapse_PPI_Communities	Syn-3_0_2312	8350	HIST1H3A
Synapse_PPI_Communities	Syn-3_0_2312	6091	ROBO1
Synapse_PPI_Communities	Syn-3_0_2312	6238	RRBP1
Synapse_PPI_Communities	Syn-3_0_2312	4701	NDUFA7
Synapse_PPI_Communities	Syn-3_0_2312	90678	LRSAM1
Synapse_PPI_Communities	Syn-3_0_2312	171024	SYNPO2
Synapse_PPI_Communities	Syn-3_0_2312	10988	METAP2
Synapse_PPI_Communities	Syn-3_0_2312	153769	SH3RF2
Synapse_PPI_Communities	Syn-3_0_2312	9892	SNAP91
Synapse_PPI_Communities	Syn-3_0_2312	2060	EPS15
Synapse_PPI_Communities	Syn-3_0_2312	51116	MRPS2
Synapse_PPI_Communities	Syn-3_0_2312	50944	SHANK1
Synapse_PPI_Communities	Syn-3_0_2312	85358	SHANK3
Synapse_PPI_Communities	Syn-3_0_2312	26156	RSL1D1
Synapse_PPI_Communities	Syn-3_0_2312	320	APBA1
Synapse_PPI_Communities	Syn-3_0_2312	55327	LIN7C
Synapse_PPI_Communities	Syn-3_0_2312	5534	PPP3R1
Synapse_PPI_Communities	Syn-3_0_2312	7187	TRAF3
Synapse_PPI_Communities	Syn-3_0_2312	7518	XRCC4
Synapse_PPI_Communities	Syn-3_0_2312	6708	SPTA1
Synapse_PPI_Communities	Syn-3_0_2312	64965	MRPS9
Synapse_PPI_Communities	Syn-3_0_2312	10262	SF3B4
Synapse_PPI_Communities	Syn-3_0_2312	1965	EIF2S1
Synapse_PPI_Communities	Syn-3_0_2312	80349	WDR61
Synapse_PPI_Communities	Syn-3_0_2312	1487	CTBP1
Synapse_PPI_Communities	Syn-3_0_2312	8467	SMARCA5
Synapse_PPI_Communities	Syn-3_0_2312	9672	SDC3
Synapse_PPI_Communities	Syn-3_0_2312	3149	HMGB3
Synapse_PPI_Communities	Syn-3_0_2312	5929	RBBP5
Synapse_PPI_Communities	Syn-3_0_2312	11222	MRPL3
Synapse_PPI_Communities	Syn-3_0_2312	29761	USP25
Synapse_PPI_Communities	Syn-3_0_2312	3030	HADHA
Synapse_PPI_Communities	Syn-3_0_2312	10196	PRMT3
Synapse_PPI_Communities	Syn-3_0_2312	9527	GOSR1
Synapse_PPI_Communities	Syn-3_0_2312	3761	KCNJ4
Synapse_PPI_Communities	Syn-3_0_2312	57510	XPO5
Synapse_PPI_Communities	Syn-3_0_2312	51520	LARS
Synapse_PPI_Communities	Syn-3_0_2312	7453	WARS
Synapse_PPI_Communities	Syn-3_0_2312	2975	GTF3C1
Synapse_PPI_Communities	Syn-3_0_2312	11235	PDCD10

Synapse_PPI_Communities	Syn-3_0_2312	22803	XRN2
Synapse_PPI_Communities	Syn-3_0_2312	6532	SLC6A4
Synapse_PPI_Communities	Syn-3_0_2312	7535	ZAP70
Synapse_PPI_Communities	Syn-3_0_2312	27330	RPS6KA6
Synapse_PPI_Communities	Syn-3_0_2312	4155	MBP
Synapse_PPI_Communities	Syn-3_0_2312	23236	PLCB1
Synapse_PPI_Communities	Syn-3_0_2312	3782	KCNN3
Synapse_PPI_Communities	Syn-3_0_2312	55234	SMU1
Synapse_PPI_Communities	Syn-3_0_2312	3840	KPNA4
Synapse_PPI_Communities	Syn-3_0_2312	572	BAD
Synapse_PPI_Communities	Syn-3_0_2312	3655	ITGA6
Synapse_PPI_Communities	Syn-3_0_2312	2289	FKBP5
Synapse_PPI_Communities	Syn-3_0_2312	6502	SKP2
Synapse_PPI_Communities	Syn-3_0_2312	5500	PPP1CB
Synapse_PPI_Communities	Syn-3_0_2312	3054	HCFC1
Synapse_PPI_Communities	Syn-3_0_2312	3949	LDLR
Synapse_PPI_Communities	Syn-3_0_2312	8764	TNFRSF14
Synapse_PPI_Communities	Syn-3_0_2312	80705	TSGA10
Synapse_PPI_Communities	Syn-3_0_2312	56924	PAK6
Synapse_PPI_Communities	Syn-3_0_2312	11021	RAB35
Synapse_PPI_Communities	Syn-3_0_2312	9519	TBPL1
Synapse_PPI_Communities	Syn-3_0_2312	51119	SBDS
Synapse_PPI_Communities	Syn-3_0_2312	5795	PTPRJ
Synapse_PPI_Communities	Syn-3_0_2312	10946	SF3A3
Synapse_PPI_Communities	Syn-3_0_2312	8570	KHSRP
Synapse_PPI_Communities	Syn-3_0_2312	7538	ZFP36
Synapse_PPI_Communities	Syn-3_0_2312	4008	LMO7
Synapse_PPI_Communities	Syn-3_0_2312	1861	TOR1A
Synapse_PPI_Communities	Syn-3_0_2312	3868	KRT16
Synapse_PPI_Communities	Syn-3_0_2312	7123	CLEC3B
Synapse_PPI_Communities	Syn-3_0_2312	311	ANXA11
Synapse_PPI_Communities	Syn-3_0_2312	6813	STXBP2
Synapse_PPI_Communities	Syn-3_0_2312	306	ANXA3
Synapse_PPI_Communities	Syn-3_0_2312	10439	OLFM1
Synapse_PPI_Communities	Syn-3_0_2312	4300	MLLT3
Synapse_PPI_Communities	Syn-3_0_2312	7048	TGFBR2
Synapse_PPI_Communities	Syn-3_0_2312	2629	GBA
Synapse_PPI_Communities	Syn-3_0_2312	435	ASL
Synapse_PPI_Communities	Syn-3_0_2312	3702	ITK
Synapse_PPI_Communities	Syn-3_0_2312	4306	NR3C2
Synapse_PPI_Communities	Syn-3_0_2312	51141	INSIG2
Synapse_PPI_Communities	Syn-3_0_2312	79885	HDAC11
Synapse_PPI_Communities	Syn-3_0_2312	80342	TRAF3IP3
Synapse_PPI_Communities	Syn-3_0_2312	23576	DDAH1
Synapse_PPI_Communities	Syn-3_0_2312	23210	JMJD6
Synapse_PPI_Communities	Syn-3_0_2312	6300	MAPK12
Synapse_PPI_Communities	Syn-3_0_2312	50809	HP1BP3
Synapse_PPI_Communities	Syn-3_0_2312	84254	CAMKK1
Synapse_PPI_Communities	Syn-3_0_2312	51202	DDX47
Synapse_PPI_Communities	Syn-3_0_2312	81624	DIAPH3
Synapse_PPI_Communities	Syn-3_0_2312	51150	SDF4
Synapse_PPI_Communities	Syn-3_0_2312	3055	HCK
Synapse_PPI_Communities	Syn-3_0_2312	9689	BZW1
Synapse_PPI_Communities	Syn-3_0_2312	112858	TP53RK
Synapse_PPI_Communities	Syn-3_0_2312	10868	USP20
Synapse_PPI_Communities	Syn-3_0_2312	2935	GSPT1
Synapse_PPI_Communities	Syn-3_0_2312	373156	GSTK1
Synapse_PPI_Communities	Syn-3_0_2312	117246	FTSJ3

Synapse_PPI_Communities	Syn-3_0_2312	6993	DYNLT1
Synapse_PPI_Communities	Syn-3_0_2312	1783	DYNC1LI2
Synapse_PPI_Communities	Syn-3_0_2312	2475	MTOR
Synapse_PPI_Communities	Syn-3_0_2312	1981	EIF4G1
Synapse_PPI_Communities	Syn-3_0_2312	23380	SRGAP2
Synapse_PPI_Communities	Syn-3_0_2312	10769	PLK2
Synapse_PPI_Communities	Syn-3_0_2312	54477	PLEKHA5
Synapse_PPI_Communities	Syn-3_0_2312	23049	SMG1
Synapse_PPI_Communities	Syn-3_0_2312	51073	MRPL4
Synapse_PPI_Communities	Syn-3_0_2312	11073	TOPBP1
Synapse_PPI_Communities	Syn-3_0_2312	682	BSG
Synapse_PPI_Communities	Syn-3_0_2312	51665	ASB1
Synapse_PPI_Communities	Syn-3_0_2312	6919	TCEA2
Synapse_PPI_Communities	Syn-3_0_2312	10713	USP39
Synapse_PPI_Communities	Syn-3_0_2312	1114	CHGB
Synapse_PPI_Communities	Syn-3_0_2312	159	ADSS
Synapse_PPI_Communities	Syn-3_0_2312	83464	APH1B
Synapse_PPI_Communities	Syn-3_0_2312	25855	BRMS1
Synapse_PPI_Communities	Syn-3_0_2312	11244	ZHX1
Synapse_PPI_Communities	Syn-3_0_2312	54919	HEATR2
Synapse_PPI_Communities	Syn-3_0_2312	4597	MVD
Synapse_PPI_Communities	Syn-3_0_2312	11065	UBE2C
Synapse_PPI_Communities	Syn-3_0_2312	3251	HPRT1
Synapse_PPI_Communities	Syn-3_0_2312	10923	SUB1
Synapse_PPI_Communities	Syn-3_0_2312	10570	DPYSL4
Synapse_PPI_Communities	Syn-3_0_2312	5800	PTPRO
Synapse_PPI_Communities	Syn-3_0_2312	51676	ASB2
Synapse_PPI_Communities	Syn-3_0_2312	10260	DENND4A
Synapse_PPI_Communities	Syn-3_0_2312	5985	RFC5
Synapse_PPI_Communities	Syn-3_0_2312	1198	CLK3
Synapse_PPI_Communities	Syn-3_0_2312	51130	ASB3
Synapse_PPI_Communities	Syn-3_0_2312	2261	FGFR3
Synapse_PPI_Communities	Syn-3_0_2312	7433	VIPR1
Synapse_PPI_Communities	Syn-3_0_2312	10956	OS9
Synapse_PPI_Communities	Syn-3_0_2312	140459	ASB6
Synapse_PPI_Communities	Syn-3_0_2312	57698	KIAA1598
Synapse_PPI_Communities	Syn-3_0_2312	8195	MKKS
Synapse_PPI_Communities	Syn-3_0_2312	11329	STK38
Synapse_PPI_Communities	Syn-3_0_2312	83481	EPPK1
Synapse_PPI_Communities	Syn-3_0_2312	5445	PON2
Synapse_PPI_Communities	Syn-3_0_2312	4281	MID1
Synapse_PPI_Communities	Syn-3_0_2312	54487	DGCR8
Synapse_PPI_Communities	Syn-3_0_2312	8850	KAT2B
Synapse_PPI_Communities	Syn-3_0_2312	64785	GINS3
Synapse_PPI_Communities	Syn-3_0_2312	56254	RNF20
Synapse_PPI_Communities	Syn-3_0_2312	79577	CDC73
Synapse_PPI_Communities	Syn-3_0_2312	26578	OSTF1
Synapse_PPI_Communities	Syn-3_0_2312	27020	NPTN
Synapse_PPI_Communities	Syn-3_0_2312	51651	PTRH2
Synapse_PPI_Communities	Syn-3_0_2312	11331	PHB2
Synapse_PPI_Communities	Syn-3_0_2312	5586	PKN2
Synapse_PPI_Communities	Syn-3_0_2312	829	CAPZA1
Synapse_PPI_Communities	Syn-3_0_2312	5877	RABIF
Synapse_PPI_Communities	Syn-3_0_2312	376267	RAB15
Synapse_PPI_Communities	Syn-3_0_2312	5306	PITPNA
Synapse_PPI_Communities	Syn-3_0_2312	9908	G3BP2
Synapse_PPI_Communities	Syn-3_0_2312	10289	EIF1B
Synapse_PPI_Communities	Syn-3_0_2312	8883	NAE1

Synapse_PPI_Communities	Syn-3_0_2312	259249	MRGPRX1
Synapse_PPI_Communities	Syn-3_0_2312	9098	USP6
Synapse_PPI_Communities	Syn-3_0_2312	5139	PDE3A
Synapse_PPI_Communities	Syn-3_0_2312	10525	HYOU1
Synapse_PPI_Communities	Syn-3_0_2312	54850	FBXL12
Synapse_PPI_Communities	Syn-3_0_2312	642	BLMH
Synapse_PPI_Communities	Syn-3_0_2312	54843	SYTL2
Synapse_PPI_Communities	Syn-3_0_2312	55794	DDX28
Synapse_PPI_Communities	Syn-3_0_2312	10197	PSME3
Synapse_PPI_Communities	Syn-3_0_2312	23708	GSPT2
Synapse_PPI_Communities	Syn-3_0_2312	8148	TAF15
Synapse_PPI_Communities	Syn-3_0_2312	3910	LAMA4
Synapse_PPI_Communities	Syn-3_0_2312	11137	PWP1
Synapse_PPI_Communities	Syn-3_0_2312	24138	IFIT5
Synapse_PPI_Communities	Syn-3_0_2312	2274	FHL2
Synapse_PPI_Communities	Syn-3_0_2312	6871	TADA2A
Synapse_PPI_Communities	Syn-3_0_2312	3267	AGFG1
Synapse_PPI_Communities	Syn-3_0_2312	51776	ZAK
Synapse_PPI_Communities	Syn-3_0_2312	51491	NOP16
Synapse_PPI_Communities	Syn-3_0_2312	55379	LRRC59
Synapse_PPI_Communities	Syn-3_0_2312	26031	OSBPL3
Synapse_PPI_Communities	Syn-3_0_2312	1558	CYP2C8
Synapse_PPI_Communities	Syn-3_0_2312	64969	MRPS5
Synapse_PPI_Communities	Syn-3_0_2312	4358	MPV17
Synapse_PPI_Communities	Syn-3_0_2312	6643	SNX2
Synapse_PPI_Communities	Syn-3_0_2312	6185	RPN2
Synapse_PPI_Communities	Syn-3_0_2312	30845	EHD3
Synapse_PPI_Communities	Syn-3_0_2312	8660	IRS2
Synapse_PPI_Communities	Syn-3_0_2312	7779	SLC30A1
Synapse_PPI_Communities	Syn-3_0_2312	4615	MYD88
Synapse_PPI_Communities	Syn-3_0_2312	11311	VPS45
Synapse_PPI_Communities	Syn-3_0_2312	51808	PHAX
Synapse_PPI_Communities	Syn-3_0_2312	10594	PRPF8
Synapse_PPI_Communities	Syn-3_0_2312	660	BMX
Synapse_PPI_Communities	Syn-3_0_2312	2064	ERBB2
Synapse_PPI_Communities	Syn-3_0_2312	11212	PROSC
Synapse_PPI_Communities	Syn-3_0_2312	57732	ZFYVE28
Synapse_PPI_Communities	Syn-3_0_2312	286	ANK1
Synapse_PPI_Communities	Syn-3_0_2312	50488	MINK1
Synapse_PPI_Communities	Syn-3_0_2312	5976	UPF1
Synapse_PPI_Communities	Syn-3_0_2312	8880	FUBP1
Synapse_PPI_Communities	Syn-3_0_2312	6790	AURKA
Synapse_PPI_Communities	Syn-3_0_2312	54840	APTX
Synapse_PPI_Communities	Syn-3_0_2312	10746	MAP3K2
Synapse_PPI_Communities	Syn-3_0_2312	5434	POLR2E
Synapse_PPI_Communities	Syn-3_0_2312	8165	AKAP1
Synapse_PPI_Communities	Syn-3_0_2312	7124	TNF
Synapse_PPI_Communities	Syn-3_0_2312	6836	SURF4
Synapse_PPI_Communities	Syn-3_0_2312	11171	STRAP
Synapse_PPI_Communities	Syn-3_0_2312	51566	ARMCX3
Synapse_PPI_Communities	Syn-3_0_2312	6642	SNX1
Synapse_PPI_Communities	Syn-3_0_2312	9212	AURKB
Synapse_PPI_Communities	Syn-3_0_2312	407	ARR3
Synapse_PPI_Communities	Syn-3_0_2312	3784	KCNQ1
Synapse_PPI_Communities	Syn-3_0_2312	7273	TTN
Synapse_PPI_Communities	Syn-3_0_2312	5982	RFC2
Synapse_PPI_Communities	Syn-3_0_2312	6648	SOD2
Synapse_PPI_Communities	Syn-3_0_2312	6301	SARS

Synapse_PPI_Communities	Syn-3_0_2312	51593	SRRT
Synapse_PPI_Communities	Syn-3_0_2312	148867	SLC30A7
Synapse_PPI_Communities	Syn-3_0_2312	3007	HIST1H1D
Synapse_PPI_Communities	Syn-3_0_2312	10992	SF3B2
Synapse_PPI_Communities	Syn-3_0_2312	29767	TMOD2
Synapse_PPI_Communities	Syn-3_0_2312	4780	NFE2L2
Synapse_PPI_Communities	Syn-3_0_2312	5833	PCYT2
Synapse_PPI_Communities	Syn-3_0_2312	2670	GFAP
Synapse_PPI_Communities	Syn-3_0_2312	6804	STX1A
Synapse_PPI_Communities	Syn-3_0_2312	6855	SYP
Synapse_PPI_Communities	Syn-3_0_2312	56902	PNO1
Synapse_PPI_Communities	Syn-3_0_2312	6599	SMARCC1
Synapse_PPI_Communities	Syn-3_0_2312	5527	PPP2R5C
Synapse_PPI_Communities	Syn-3_0_2312	27316	RBMX
Synapse_PPI_Communities	Syn-3_0_2312	23184	MESDC2
Synapse_PPI_Communities	Syn-3_0_2312	10425	ARIH2
Synapse_PPI_Communities	Syn-3_0_2312	800	CALD1
Synapse_PPI_Communities	Syn-3_0_2312	29115	SAP30BP
Synapse_PPI_Communities	Syn-3_0_2312	355	FAS
Synapse_PPI_Communities	Syn-3_0_2312	5110	PCMT1
Synapse_PPI_Communities	Syn-3_0_2312	3336	HSPE1
Synapse_PPI_Communities	Syn-3_0_2312	689	BTF3
Synapse_PPI_Communities	Syn-3_0_2312	10724	MGEA5
Synapse_PPI_Communities	Syn-3_0_2312	10533	ATG7
Synapse_PPI_Communities	Syn-3_0_2312	10008	KCNE3
Synapse_PPI_Communities	Syn-3_0_2312	5720	PSME1
Synapse_PPI_Communities	Syn-3_0_2312	23139	MAST2
Synapse_PPI_Communities	Syn-3_0_2312	7070	THY1
Synapse_PPI_Communities	Syn-3_0_2312	84324	SARNP
Synapse_PPI_Communities	Syn-3_0_2312	4190	MDH1
Synapse_PPI_Communities	Syn-3_0_2312	6389	SDHA
Synapse_PPI_Communities	Syn-3_0_2312	5481	PPID
Synapse_PPI_Communities	Syn-3_0_2312	1429	CRYZ
Synapse_PPI_Communities	Syn-3_0_2312	140576	S100A16
Synapse_PPI_Communities	Syn-3_0_2312	55658	RNF126
Synapse_PPI_Communities	Syn-3_0_2312	8237	USP11
Synapse_PPI_Communities	Syn-3_0_2312	3280	HES1
Synapse_PPI_Communities	Syn-3_0_2312	47	ACLY
Synapse_PPI_Communities	Syn-3_0_2312	4149	MAX
Synapse_PPI_Communities	Syn-3_0_2312	1741	DLG3
Synapse_PPI_Communities	Syn-3_0_2312	57555	NLGN2
Synapse_PPI_Communities	Syn-3_0_2312	54413	NLGN3
Synapse_PPI_Communities	Syn-3_0_2312	2318	FLNC
Synapse_PPI_Communities	Syn-3_0_2312	2309	FOXO3
Synapse_PPI_Communities	Syn-3_0_2312	509	ATP5C1
Synapse_PPI_Communities	Syn-3_0_2312	472	ATM
Synapse_PPI_Communities	Syn-3_0_2312	1978	EIF4EBP1
Synapse_PPI_Communities	Syn-3_0_2312	8536	CAMK1
Synapse_PPI_Communities	Syn-3_0_2312	10645	CAMKK2
Synapse_PPI_Communities	Syn-3_0_2312	2034	EPAS1
Synapse_PPI_Communities	Syn-3_0_2312	26047	CNTNAP2
Synapse_PPI_Communities	Syn-3_0_2312	23499	MACF1
Synapse_PPI_Communities	Syn-3_0_2312	6294	SAFB
Synapse_PPI_Communities	Syn-3_0_2312	5793	PTPRG
Synapse_PPI_Communities	Syn-3_0_2312	8500	PPFIA1
Synapse_PPI_Communities	Syn-3_0_2312	55227	LRRC1
Synapse_PPI_Communities	Syn-3_0_2312	10419	PRMT5
Synapse_PPI_Communities	Syn-3_0_2312	5610	EIF2AK2

Synapse_PPI_Communities	Syn-3_0_2312	8870	IER3
Synapse_PPI_Communities	Syn-3_0_2312	5272	SERPINB9
Synapse_PPI_Communities	Syn-3_0_2312	6829	SUPT5H
Synapse_PPI_Communities	Syn-3_0_2312	57223	SMEK2
Synapse_PPI_Communities	Syn-3_0_2312	55207	ARL8B
Synapse_PPI_Communities	Syn-3_0_2312	1394	CRHR1
Synapse_PPI_Communities	Syn-3_0_2312	25814	ATXN10
Synapse_PPI_Communities	Syn-3_0_2312	7450	VWF
Synapse_PPI_Communities	Syn-3_0_2312	55291	PPP6R3
Synapse_PPI_Communities	Syn-3_0_2312	6453	ITSN1
Synapse_PPI_Communities	Syn-3_0_2312	8871	SYNJ2
Synapse_PPI_Communities	Syn-3_0_2312	6434	TRA2B
Synapse_PPI_Communities	Syn-3_0_2312	5603	MAPK13
Synapse_PPI_Communities	Syn-3_0_2312	3667	IRS1
Synapse_PPI_Communities	Syn-3_0_2312	9611	NCOR1
Synapse_PPI_Communities	Syn-3_0_2312	8851	CDK5R1
Synapse_PPI_Communities	Syn-3_0_2312	10369	CACNG2
Synapse_PPI_Communities	Syn-3_0_2312	57473	ZNF512B
Synapse_PPI_Communities	Syn-3_0_2312	27429	HTRA2
Synapse_PPI_Communities	Syn-3_0_2312	5144	PDE4D
Synapse_PPI_Communities	Syn-3_0_2312	1647	GADD45A
Synapse_PPI_Communities	Syn-3_0_2312	7468	WHSC1
Synapse_PPI_Communities	Syn-3_0_2312	3297	HSF1
Synapse_PPI_Communities	Syn-3_0_2312	22824	HSPA4L
Synapse_PPI_Communities	Syn-3_0_2312	995	CDC25C
Synapse_PPI_Communities	Syn-3_0_2312	55737	VPS35
Synapse_PPI_Communities	Syn-3_0_2312	8892	EIF2B2
Synapse_PPI_Communities	Syn-3_0_2312	1399	CRKL
Synapse_PPI_Communities	Syn-3_0_2312	1121	CHM
Synapse_PPI_Communities	Syn-3_0_2312	5870	RAB6A
Synapse_PPI_Communities	Syn-3_0_2312	11200	CHEK2
Synapse_PPI_Communities	Syn-3_0_2312	7448	VTN
Synapse_PPI_Communities	Syn-3_0_2312	1759	DNM1
Synapse_PPI_Communities	Syn-3_0_2312	29763	PACSIN3
Synapse_PPI_Communities	Syn-3_0_2312	3872	KRT17
Synapse_PPI_Communities	Syn-3_0_2312	816	CAMK2B
Synapse_PPI_Communities	Syn-3_0_2312	226	ALDOA
Synapse_PPI_Communities	Syn-3_0_2312	5865	RAB3B
Synapse_PPI_Communities	Syn-3_0_2312	22841	RAB11FIP2
Synapse_PPI_Communities	Syn-3_0_2312	6720	SREBF1
Synapse_PPI_Communities	Syn-3_0_2312	10040	TOM1L1
Synapse_PPI_Communities	Syn-3_0_2312	6712	SPTBN2
Synapse_PPI_Communities	Syn-3_0_2312	1387	CREBBP
Synapse_PPI_Communities	Syn-3_0_2312	4688	NCF2
Synapse_PPI_Communities	Syn-3_0_2312	6383	SDC2
Synapse_PPI_Communities	Syn-3_0_2312	3861	KRT14
Synapse_PPI_Communities	Syn-3_0_2312	8202	NCOA3
Synapse_PPI_Communities	Syn-3_0_2312	6626	SNRPA
Synapse_PPI_Communities	Syn-3_0_2312	51474	LIMA1
Synapse_PPI_Communities	Syn-3_0_2312	26043	UBXN7
Synapse_PPI_Communities	Syn-3_0_2312	6559	SLC12A3
Synapse_PPI_Communities	Syn-3_0_2312	581	BAX
Synapse_PPI_Communities	Syn-3_0_2312	58	ACTA1
Synapse_PPI_Communities	Syn-3_0_2312	23406	COTL1
Synapse_PPI_Communities	Syn-3_0_2312	10910	SUGT1
Synapse_PPI_Communities	Syn-3_0_2312	3575	IL7R
Synapse_PPI_Communities	Syn-3_0_2312	117189	GRDX
Synapse_PPI_Communities	Syn-3_0_2312	51143	DYNC1LI1

Synapse_PPI_Communities	Syn-3_0_2312	25824	PRDX5
Synapse_PPI_Communities	Syn-3_0_2312	81605	URM1
Synapse_PPI_Communities	Syn-3_0_2312	4217	MAP3K5
Synapse_PPI_Communities	Syn-3_0_2312	7322	UBE2D2
Synapse_PPI_Communities	Syn-3_0_2312	29927	SEC61A1
Synapse_PPI_Communities	Syn-3_0_2312	833	CARS
Synapse_PPI_Communities	Syn-3_0_2312	5451	POU2F1
Synapse_PPI_Communities	Syn-3_0_2312	3708	ITPR1
Synapse_PPI_Communities	Syn-3_0_2312	330	BIRC3
Synapse_PPI_Communities	Syn-3_0_2312	9554	SEC22B
Synapse_PPI_Communities	Syn-3_0_2312	51534	VTA1
Synapse_PPI_Communities	Syn-3_0_2312	5707	PSMD1
Synapse_PPI_Communities	Syn-3_0_2312	7249	TSC2
Synapse_PPI_Communities	Syn-3_0_2312	2648	KAT2A
Synapse_PPI_Communities	Syn-3_0_2312	3983	ABLIM1
Synapse_PPI_Communities	Syn-3_0_2312	50619	DEF6
Synapse_PPI_Communities	Syn-3_0_2312	3843	IPO5
Synapse_PPI_Communities	Syn-3_0_2312	7018	TF
Synapse_PPI_Communities	Syn-3_0_2312	23039	XPO7
Synapse_PPI_Communities	Syn-3_0_2312	9361	LONP1
Synapse_PPI_Communities	Syn-3_0_2312	9513	FXR2
Synapse_PPI_Communities	Syn-3_0_2312	7013	TERF1
Synapse_PPI_Communities	Syn-3_0_2312	5780	PTPN9
Synapse_PPI_Communities	Syn-3_0_2312	7158	TP53BP1
Synapse_PPI_Communities	Syn-3_0_2312	23256	SCFD1
Synapse_PPI_Communities	Syn-3_0_2312	53917	RAB24
Synapse_PPI_Communities	Syn-3_0_2312	57179	KIAA1191
Synapse_PPI_Communities	Syn-3_0_2312	5094	PCBP2
Synapse_PPI_Communities	Syn-3_0_2312	5290	PIK3CA
Synapse_PPI_Communities	Syn-3_0_2312	1447	CSN2
Synapse_PPI_Communities	Syn-3_0_2312	9482	STX8
Synapse_PPI_Communities	Syn-3_0_2312	10490	VTI1B
Synapse_PPI_Communities	Syn-3_0_2312	7311	UBA52
Synapse_PPI_Communities	Syn-3_0_2312	9113	LATS1
Synapse_PPI_Communities	Syn-3_0_2312	5573	PRKAR1A
Synapse_PPI_Communities	Syn-3_0_2312	898	CCNE1
Synapse_PPI_Communities	Syn-3_0_2312	9092	SART1
Synapse_PPI_Communities	Syn-3_0_2312	10036	CHAF1A
Synapse_PPI_Communities	Syn-3_0_2312	8666	EIF3G
Synapse_PPI_Communities	Syn-3_0_2312	9295	SRSF11
Synapse_PPI_Communities	Syn-3_0_2312	477	ATP1A2
Synapse_PPI_Communities	Syn-3_0_2312	81565	NDEL1
Synapse_PPI_Communities	Syn-3_0_2312	11252	PACSIN2
Synapse_PPI_Communities	Syn-3_0_2312	6633	SNRPD2
Synapse_PPI_Communities	Syn-3_0_2312	3189	HNRNPH3
Synapse_PPI_Communities	Syn-3_0_2312	26088	GGA1
Synapse_PPI_Communities	Syn-3_0_2312	891	CCNB1
Synapse_PPI_Communities	Syn-3_0_2312	8874	ARHGEF7
Synapse_PPI_Communities	Syn-3_0_2312	4751	NEK2
Synapse_PPI_Communities	Syn-3_0_2312	2550	GABBR1
Synapse_PPI_Communities	Syn-3_0_2312	5999	RGS4
Synapse_PPI_Communities	Syn-3_0_2312	10989	IMMT
Synapse_PPI_Communities	Syn-3_0_2312	2280	FKBP1A
Synapse_PPI_Communities	Syn-3_0_2312	7528	YY1
Synapse_PPI_Communities	Syn-3_0_2312	3125	HLA-DRB3
Synapse_PPI_Communities	Syn-3_0_2312	2560	GABRB1
Synapse_PPI_Communities	Syn-3_0_2312	25898	RCHY1
Synapse_PPI_Communities	Syn-3_0_2312	345651	ACTBL2

Synapse_PPI_Communities	Syn-3_0_2312	3725	JUN
Synapse_PPI_Communities	Syn-3_0_2312	25977	NECAP1
Synapse_PPI_Communities	Syn-3_0_2312	832	CAPZB
Synapse_PPI_Communities	Syn-3_0_2312	9061	PAPSS1
Synapse_PPI_Communities	Syn-3_0_2312	3005	H1F0
Synapse_PPI_Communities	Syn-3_0_2312	80331	DNAJC5
Synapse_PPI_Communities	Syn-3_0_2312	57122	NUP107
Synapse_PPI_Communities	Syn-3_0_2312	10949	HNRNPA0
Synapse_PPI_Communities	Syn-3_0_2312	7168	TPM1
Synapse_PPI_Communities	Syn-3_0_2312	5917	RARS
Synapse_PPI_Communities	Syn-3_0_2312	5007	OSBP
Synapse_PPI_Communities	Syn-3_0_2312	3315	HSPB1
Synapse_PPI_Communities	Syn-3_0_2312	1021	CDK6
Synapse_PPI_Communities	Syn-3_0_2312	375790	AGRN
Synapse_PPI_Communities	Syn-3_0_2312	439	ASNA1
Synapse_PPI_Communities	Syn-3_0_2312	9374	PPT2
Synapse_PPI_Communities	Syn-3_0_2312	545	ATR
Synapse_PPI_Communities	Syn-3_0_2312	10521	DDX17
Synapse_PPI_Communities	Syn-3_0_2312	373509	USP50
Synapse_PPI_Communities	Syn-3_0_2312	1018	CDK3
Synapse_PPI_Communities	Syn-3_0_2312	29904	EEF2K
Synapse_PPI_Communities	Syn-3_0_2312	5214	PFKP
Synapse_PPI_Communities	Syn-3_0_2312	1762	DMWD
Synapse_PPI_Communities	Syn-3_0_2312	55577	NAGK
Synapse_PPI_Communities	Syn-3_0_2312	147746	HIPK4
Synapse_PPI_Communities	Syn-3_0_2312	196541	METTL21C
Synapse_PPI_Communities	Syn-3_0_2312	10494	STK25
Synapse_PPI_Communities	Syn-3_0_2312	440574	MINOS1
Synapse_PPI_Communities	Syn-3_0_2312	5705	PSMC5
Synapse_PPI_Communities	Syn-3_0_2312	8445	DYRK2
Synapse_PPI_Communities	Syn-3_0_2312	7314	UBB
Synapse_PPI_Communities	Syn-3_0_2312	10486	CAP2
Synapse_PPI_Communities	Syn-3_0_2312	4173	MCM4
Synapse_PPI_Communities	Syn-3_0_2312	8554	PIAS1
Synapse_PPI_Communities	Syn-3_0_2312	84936	ZFYVE19
Synapse_PPI_Communities	Syn-3_0_2312	29937	NENF
Synapse_PPI_Communities	Syn-3_0_2312	8335	HIST1H2AB
Synapse_PPI_Communities	Syn-3_0_2312	6854	SYN2
Synapse_PPI_Communities	Syn-3_0_2312	4259	MGST3
Synapse_PPI_Communities	Syn-3_0_2312	23077	MYCBP2
Synapse_PPI_Communities	Syn-3_0_2312	2931	GSK3A
Synapse_PPI_Communities	Syn-3_0_2312	9774	BCLAF1
Synapse_PPI_Communities	Syn-3_0_2312	11228	RASSF8
Synapse_PPI_Communities	Syn-3_0_2312	11098	PRSS23
Synapse_PPI_Communities	Syn-3_0_2312	2639	GCDH
Synapse_PPI_Communities	Syn-3_0_2312	5577	PRKAR2B
Synapse_PPI_Communities	Syn-3_0_2312	8615	USO1
Synapse_PPI_Communities	Syn-3_0_2312	50855	PARD6A
Synapse_PPI_Communities	Syn-3_0_2312	4363	ABCC1
Synapse_PPI_Communities	Syn-3_0_2312	775	CACNA1C
Synapse_PPI_Communities	Syn-3_0_2312	10206	TRIM13
Synapse_PPI_Communities	Syn-3_0_2312	1604	CD55
Synapse_PPI_Communities	Syn-3_0_2312	6430	SRSF5
Synapse_PPI_Communities	Syn-3_0_2312	9972	NUP153
Synapse_PPI_Communities	Syn-3_0_2312	51574	LARP7
Synapse_PPI_Communities	Syn-3_0_2312	6727	SRP14
Synapse_PPI_Communities	Syn-3_0_2312	9372	ZFYVE9
Synapse_PPI_Communities	Syn-3_0_2312	9188	DDX21

Synapse_PPI_Communities	Syn-3_0_2312	2353	FOS
Synapse_PPI_Communities	Syn-3_0_2312	8994	LIMD1
Synapse_PPI_Communities	Syn-3_0_2312	5533	PPP3CC
Synapse_PPI_Communities	Syn-3_0_2312	6249	CLIP1
Synapse_PPI_Communities	Syn-3_0_2312	51663	ZFR
Synapse_PPI_Communities	Syn-3_0_2312	51015	ISOC1
Synapse_PPI_Communities	Syn-3_0_2312	5739	PTGIR
Synapse_PPI_Communities	Syn-3_0_2312	1314	COPA
Synapse_PPI_Communities	Syn-3_0_2312	3127	HLA-DRB5
Synapse_PPI_Communities	Syn-3_0_2312	11215	AKAP11
Synapse_PPI_Communities	Syn-3_0_2312	51564	HDAC7
Synapse_PPI_Communities	Syn-3_0_2312	3852	KRT5
Synapse_PPI_Communities	Syn-3_0_2312	79598	CEP97
Synapse_PPI_Communities	Syn-3_0_2312	23118	TAB2
Synapse_PPI_Communities	Syn-3_0_2312	1191	CLU
Synapse_PPI_Communities	Syn-3_0_2312	9093	DNAJA3
Synapse_PPI_Communities	Syn-3_0_2312	51726	DNAJB11
Synapse_PPI_Communities	Syn-3_0_2312	27101	CACYBP
Synapse_PPI_Communities	Syn-3_0_2312	60485	SAV1
Synapse_PPI_Communities	Syn-3_0_2312	10236	HNRNPR
Synapse_PPI_Communities	Syn-3_0_2312	3126	HLA-DRB4
Synapse_PPI_Communities	Syn-3_0_2312	23534	TNPO3
Synapse_PPI_Communities	Syn-3_0_2312	5692	PSMB4
Synapse_PPI_Communities	Syn-3_0_2312	9873	FCHSD2
Synapse_PPI_Communities	Syn-3_0_2312	11338	U2AF2
Synapse_PPI_Communities	Syn-3_0_2312	4189	DNAJB9
Synapse_PPI_Communities	Syn-3_0_2312	90627	STARD13
Synapse_PPI_Communities	Syn-3_0_2312	84078	KBTBD7
Synapse_PPI_Communities	Syn-3_0_2312	26128	KIAA1279
Synapse_PPI_Communities	Syn-3_0_2312	83660	TLN2
Synapse_PPI_Communities	Syn-3_0_2312	6598	SMARCB1
Synapse_PPI_Communities	Syn-3_0_2312	3047	HBG1
Synapse_PPI_Communities	Syn-3_0_2312	6635	SNRPE
Synapse_PPI_Communities	Syn-3_0_2312	11031	RAB31
Synapse_PPI_Communities	Syn-3_0_2312	55573	CDV3
Synapse_PPI_Communities	Syn-3_0_2312	8473	OGT
Synapse_PPI_Communities	Syn-3_0_2312	823	CAPN1
Synapse_PPI_Communities	Syn-3_0_2312	8626	TP63
Synapse_PPI_Communities	Syn-3_0_2312	5829	PXN
Synapse_PPI_Communities	Syn-3_0_2312	4790	NFKB1
Synapse_PPI_Communities	Syn-3_0_2312	4131	MAP1B
Synapse_PPI_Communities	Syn-3_0_2312	65018	PINK1
Synapse_PPI_Communities	Syn-3_0_2312	10627	MYL12A
Synapse_PPI_Communities	Syn-3_0_2312	7298	TYMS
Synapse_PPI_Communities	Syn-3_0_2312	4926	NUMA1
Synapse_PPI_Communities	Syn-3_0_2312	80700	UBXN6
Synapse_PPI_Communities	Syn-3_0_2312	3146	HMGB1
Synapse_PPI_Communities	Syn-3_0_2312	64750	SMURF2
Synapse_PPI_Communities	Syn-3_0_2312	5962	RDX
Synapse_PPI_Communities	Syn-3_0_2312	10284	SAP18
Synapse_PPI_Communities	Syn-3_0_2312	1892	ECHS1
Synapse_PPI_Communities	Syn-3_0_2312	2271	FH
Synapse_PPI_Communities	Syn-3_0_2312	3984	LIMK1
Synapse_PPI_Communities	Syn-3_0_2312	64175	LEPRE1
Synapse_PPI_Communities	Syn-3_0_2312	23603	CORO1C
Synapse_PPI_Communities	Syn-3_0_2312	55869	HDAC8
Synapse_PPI_Communities	Syn-3_0_2312	7385	UQCRC2
Synapse_PPI_Communities	Syn-3_0_2312	5717	PSMD11

Synapse_PPI_Communities	Syn-3_0_2312	8535	CBX4
Synapse_PPI_Communities	Syn-3_0_2312	254013	METTL20
Synapse_PPI_Communities	Syn-3_0_2312	151194	METTL21A
Synapse_PPI_Communities	Syn-3_0_2312	3897	L1CAM
Synapse_PPI_Communities	Syn-3_0_2312	64786	TBC1D15
Synapse_PPI_Communities	Syn-3_0_2312	3337	DNAJB1
Synapse_PPI_Communities	Syn-3_0_2312	3551	IKBKB
Synapse_PPI_Communities	Syn-3_0_2312	85365	ALG2
Synapse_PPI_Communities	Syn-3_0_2312	3916	LAMP1
Synapse_PPI_Communities	Syn-3_0_2312	2185	PTK2B
Synapse_PPI_Communities	Syn-3_0_2312	7150	TOP1
Synapse_PPI_Communities	Syn-3_0_2312	29760	BLNK
Synapse_PPI_Communities	Syn-3_0_2312	830	CAPZA2
Synapse_PPI_Communities	Syn-3_0_2312	3849	KRT2
Synapse_PPI_Communities	Syn-3_0_2312	841	CASP8
Synapse_PPI_Communities	Syn-3_0_2312	2908	NR3C1
Synapse_PPI_Communities	Syn-3_0_2312	23410	SIRT3
Synapse_PPI_Communities	Syn-3_0_2312	6520	SLC3A2
Synapse_PPI_Communities	Syn-3_0_2312	8140	SLC7A5
Synapse_PPI_Communities	Syn-3_0_2312	55366	LGR4
Synapse_PPI_Communities	Syn-3_0_2312	6767	ST13
Synapse_PPI_Communities	Syn-3_0_2312	6446	SGK1
Synapse_PPI_Communities	Syn-3_0_2312	7015	TERT
Synapse_PPI_Communities	Syn-3_0_2312	356	FASLG
Synapse_PPI_Communities	Syn-3_0_2312	31	ACACA
Synapse_PPI_Communities	Syn-3_0_2312	27131	SNX5
Synapse_PPI_Communities	Syn-3_0_2312	8774	NAPG
Synapse_PPI_Communities	Syn-3_0_2312	4343	MOV10
Synapse_PPI_Communities	Syn-3_0_2312	5786	PTPRA
Synapse_PPI_Communities	Syn-3_0_2312	5019	OXCT1
Synapse_PPI_Communities	Syn-3_0_2312	5802	PTPRS
Synapse_PPI_Communities	Syn-3_0_2312	1984	EIF5A
Synapse_PPI_Communities	Syn-3_0_2312	2861	GPR37
Synapse_PPI_Communities	Syn-3_0_2312	7114	TMSB4X
Synapse_PPI_Communities	Syn-3_0_2312	1130	LYST
Synapse_PPI_Communities	Syn-3_0_2312	3689	ITGB2
Synapse_PPI_Communities	Syn-3_0_2312	5198	PFAS
Synapse_PPI_Communities	Syn-3_0_2312	7511	XPNPEP1
Synapse_PPI_Communities	Syn-3_0_2312	5701	PSMC2
Synapse_PPI_Communities	Syn-3_0_2312	10015	PDCD6IP
Synapse_PPI_Communities	Syn-3_0_2312	1786	DNMT1
Synapse_PPI_Communities	Syn-3_0_2312	6667	SP1
Synapse_PPI_Communities	Syn-3_0_2312	23400	ATP13A2
Synapse_PPI_Communities	Syn-3_0_2312	3660	IRF2
Synapse_PPI_Communities	Syn-3_0_2312	84557	MAP1LC3A
Synapse_PPI_Communities	Syn-3_0_2312	4194	MDM4
Synapse_PPI_Communities	Syn-3_0_2312	54926	UBE2R2
Synapse_PPI_Communities	Syn-3_0_2312	5898	RALA
Synapse_PPI_Communities	Syn-3_0_2312	5912	RAP2B
Synapse_PPI_Communities	Syn-3_0_2312	7706	TRIM25
Synapse_PPI_Communities	Syn-3_0_2312	22931	RAB18
Synapse_PPI_Communities	Syn-3_0_2312	64857	PLEKHG2
Synapse_PPI_Communities	Syn-3_0_2312	23607	CD2AP
Synapse_PPI_Communities	Syn-3_0_2312	3123	HLA-DRB1
Synapse_PPI_Communities	Syn-3_0_2312	338	APOB
Synapse_PPI_Communities	Syn-3_0_2312	1453	CSNK1D
Synapse_PPI_Communities	Syn-3_0_2312	6721	SREBF2
Synapse_PPI_Communities	Syn-3_0_2312	134957	STXBP5

Synapse_PPI_Communities	Syn-3_0_2312	5688	PSMA7
Synapse_PPI_Communities	Syn-3_0_2312	4176	MCM7
Synapse_PPI_Communities	Syn-3_0_2312	5162	PDHB
Synapse_PPI_Communities	Syn-3_0_2312	6601	SMARCC2
Synapse_PPI_Communities	Syn-3_0_2312	4168	MCF2
Synapse_PPI_Communities	Syn-3_0_2312	293	SLC25A6
Synapse_PPI_Communities	Syn-3_0_2312	5347	PLK1
Synapse_PPI_Communities	Syn-3_0_2312	84708	LNX1
Synapse_PPI_Communities	Syn-3_0_2312	6477	SIAH1
Synapse_PPI_Communities	Syn-3_0_2312	3122	HLA-DRA
Synapse_PPI_Communities	Syn-3_0_2312	7276	TTR
Synapse_PPI_Communities	Syn-3_0_2312	5685	PSMA4
Synapse_PPI_Communities	Syn-3_0_2312	2035	EPB41
Synapse_PPI_Communities	Syn-3_0_2312	197131	UBR1
Synapse_PPI_Communities	Syn-3_0_2312	11160	ERLIN2
Synapse_PPI_Communities	Syn-3_0_2312	5862	RAB2A
Synapse_PPI_Communities	Syn-3_0_2312	112464	PRKCDBP
Synapse_PPI_Communities	Syn-3_0_2312	10131	TRAP1
Synapse_PPI_Communities	Syn-3_0_2312	613	BCR
Synapse_PPI_Communities	Syn-3_0_2312	4234	METTL1
Synapse_PPI_Communities	Syn-3_0_2312	142686	ASB14
Synapse_PPI_Communities	Syn-3_0_2312	4086	SMAD1
Synapse_PPI_Communities	Syn-3_0_2312	1147	CHUK
Synapse_PPI_Communities	Syn-3_0_2312	2956	MSH6
Synapse_PPI_Communities	Syn-3_0_2312	7073	TIAL1
Synapse_PPI_Communities	Syn-3_0_2312	5562	PRKAA1
Synapse_PPI_Communities	Syn-3_0_2312	10018	BCL2L11
Synapse_PPI_Communities	Syn-3_0_2312	7132	TNFRSF1A
Synapse_PPI_Communities	Syn-3_0_2312	4355	MPP2
Synapse_PPI_Communities	Syn-3_0_2312	4436	MSH2
Synapse_PPI_Communities	Syn-3_0_2312	5873	RAB27A
Synapse_PPI_Communities	Syn-3_0_2312	347733	TUBB2B
Synapse_PPI_Communities	Syn-3_0_2312	79091	METTL22
Synapse_PPI_Communities	Syn-3_0_2312	5108	PCM1
Synapse_PPI_Communities	Syn-3_0_2312	5430	POLR2A
Synapse_PPI_Communities	Syn-3_0_2312	7409	VAV1
Synapse_PPI_Communities	Syn-3_0_2312	554	AVPR2
Synapse_PPI_Communities	Syn-3_0_2312	54331	GNG2
Synapse_PPI_Communities	Syn-3_0_2312	5050	PAFAH1B3
Synapse_PPI_Communities	Syn-3_0_2312	3654	IRAK1
Synapse_PPI_Communities	Syn-3_0_2312	7408	VASP
Synapse_PPI_Communities	Syn-3_0_2312	4522	MTHFD1
Synapse_PPI_Communities	Syn-3_0_2312	5860	QDPR
Synapse_PPI_Communities	Syn-3_0_2312	5250	SLC25A3
Synapse_PPI_Communities	Syn-3_0_2312	3159	HMGA1
Synapse_PPI_Communities	Syn-3_0_2312	88	ACTN2
Synapse_PPI_Communities	Syn-3_0_2312	2904	GRIN2B
Synapse_PPI_Communities	Syn-3_0_2312	3717	JAK2
Synapse_PPI_Communities	Syn-3_0_2312	9997	SCO2
Synapse_PPI_Communities	Syn-3_0_2312	333	APLP1
Synapse_PPI_Communities	Syn-3_0_2312	5504	PPP1R2
Synapse_PPI_Communities	Syn-3_0_2312	10075	HUWE1
Synapse_PPI_Communities	Syn-3_0_2312	6472	SHMT2
Synapse_PPI_Communities	Syn-3_0_2312	9533	POLR1C
Synapse_PPI_Communities	Syn-3_0_2312	7283	TUBG1
Synapse_PPI_Communities	Syn-3_0_2312	23640	HSPBP1
Synapse_PPI_Communities	Syn-3_0_2312	5606	MAP2K3
Synapse_PPI_Communities	Syn-3_0_2312	27250	PDCD4

Synapse_PPI_Communities	Syn-3_0_2312	6632	SNRPD1
Synapse_PPI_Communities	Syn-3_0_2312	7407	VARS
Synapse_PPI_Communities	Syn-3_0_2312	23677	SH3BP4
Synapse_PPI_Communities	Syn-3_0_2312	27338	UBE2S
Synapse_PPI_Communities	Syn-3_0_2312	2066	ERBB4
Synapse_PPI_Communities	Syn-3_0_2312	6853	SYN1
Synapse_PPI_Communities	Syn-3_0_2312	5921	RASA1
Synapse_PPI_Communities	Syn-3_0_2312	2175	FANCA
Synapse_PPI_Communities	Syn-3_0_2312	1855	DVL1
Synapse_PPI_Communities	Syn-3_0_2312	4092	SMAD7
Synapse_PPI_Communities	Syn-3_0_2312	1459	CSNK2A2
Synapse_PPI_Communities	Syn-3_0_2312	7159	TP53BP2
Synapse_PPI_Communities	Syn-3_0_2312	27339	PRPF19
Synapse_PPI_Communities	Syn-3_0_2312	10625	IVNS1ABP
Synapse_PPI_Communities	Syn-3_0_2312	9643	MORF4L2
Synapse_PPI_Communities	Syn-3_0_2312	4311	MME
Synapse_PPI_Communities	Syn-3_0_2312	51138	COPS4
Synapse_PPI_Communities	Syn-3_0_2312	253260	RICTOR
Synapse_PPI_Communities	Syn-3_0_2312	10768	AHCYL1
Synapse_PPI_Communities	Syn-3_0_2312	3925	STMN1
Synapse_PPI_Communities	Syn-3_0_2312	983	CDK1
Synapse_PPI_Communities	Syn-3_0_2312	471	ATIC
Synapse_PPI_Communities	Syn-3_0_2312	6850	SYK
Synapse_PPI_Communities	Syn-3_0_2312	25942	SIN3A
Synapse_PPI_Communities	Syn-3_0_2312	6843	VAMP1
Synapse_PPI_Communities	Syn-3_0_2312	9217	VAPB
Synapse_PPI_Communities	Syn-3_0_2312	5204	PFDN5
Synapse_PPI_Communities	Syn-3_0_2312	4026	LPP
Synapse_PPI_Communities	Syn-3_0_2312	2033	EP300
Synapse_PPI_Communities	Syn-3_0_2312	8945	BTRC
Synapse_PPI_Communities	Syn-3_0_2312	1917	EEF1A2
Synapse_PPI_Communities	Syn-3_0_2312	9352	TXNL1
Synapse_PPI_Communities	Syn-3_0_2312	11124	FAF1
Synapse_PPI_Communities	Syn-3_0_2312	83658	DYNLRB1
Synapse_PPI_Communities	Syn-3_0_2312	9685	CLINT1
Synapse_PPI_Communities	Syn-3_0_2312	490	ATP2B1
Synapse_PPI_Communities	Syn-3_0_2312	9759	HDAC4
Synapse_PPI_Communities	Syn-3_0_2312	1019	CDK4
Synapse_PPI_Communities	Syn-3_0_2312	7153	TOP2A
Synapse_PPI_Communities	Syn-3_0_2312	7335	UBE2V1
Synapse_PPI_Communities	Syn-3_0_2312	6342	SCP2
Synapse_PPI_Communities	Syn-3_0_2312	163	AP2B1
Synapse_PPI_Communities	Syn-3_0_2312	29924	EPN1
Synapse_PPI_Communities	Syn-3_0_2312	2100	ESR2
Synapse_PPI_Communities	Syn-3_0_2312	4171	MCM2
Synapse_PPI_Communities	Syn-3_0_2312	10681	GNB5
Synapse_PPI_Communities	Syn-3_0_2312	6197	RPS6KA3
Synapse_PPI_Communities	Syn-3_0_2312	3185	HNRNPF
Synapse_PPI_Communities	Syn-3_0_2312	6741	SSB
Synapse_PPI_Communities	Syn-3_0_2312	84687	PPP1R9B
Synapse_PPI_Communities	Syn-3_0_2312	491	ATP2B2
Synapse_PPI_Communities	Syn-3_0_2312	3183	HNRNPC
Synapse_PPI_Communities	Syn-3_0_2312	5911	RAP2A
Synapse_PPI_Communities	Syn-3_0_2312	4734	NEDD4
Synapse_PPI_Communities	Syn-3_0_2312	9588	PRDX6
Synapse_PPI_Communities	Syn-3_0_2312	7064	THOP1
Synapse_PPI_Communities	Syn-3_0_2312	752	FMNL1
Synapse_PPI_Communities	Syn-3_0_2312	10813	UTP14A

Synapse_PPI_Communities	Syn-3_0_2312	7881	KCNAB1
Synapse_PPI_Communities	Syn-3_0_2312	150	ADRA2A
Synapse_PPI_Communities	Syn-3_0_2312	56288	PARD3
Synapse_PPI_Communities	Syn-3_0_2312	4294	MAP3K10
Synapse_PPI_Communities	Syn-3_0_2312	5708	PSMD2
Synapse_PPI_Communities	Syn-3_0_2312	488	ATP2A2
Synapse_PPI_Communities	Syn-3_0_2312	140458	ASB5
Synapse_PPI_Communities	Syn-3_0_2312	6531	SLC6A3
Synapse_PPI_Communities	Syn-3_0_2312	9463	PICK1
Synapse_PPI_Communities	Syn-3_0_2312	5339	PLEC
Synapse_PPI_Communities	Syn-3_0_2312	3735	KARS
Synapse_PPI_Communities	Syn-3_0_2312	81631	MAP1LC3B
Synapse_PPI_Communities	Syn-3_0_2312	1622	DBI
Synapse_PPI_Communities	Syn-3_0_2312	57794	SUGP1
Synapse_PPI_Communities	Syn-3_0_2312	10067	SCAMP3
Synapse_PPI_Communities	Syn-3_0_2312	7536	SF1
Synapse_PPI_Communities	Syn-3_0_2312	6749	SSRP1
Synapse_PPI_Communities	Syn-3_0_2312	637	BID
Synapse_PPI_Communities	Syn-3_0_2312	6009	RHEB
Synapse_PPI_Communities	Syn-3_0_2312	3838	KPNA2
Synapse_PPI_Communities	Syn-3_0_2312	10273	STUB1
Synapse_PPI_Communities	Syn-3_0_2312	22933	SIRT2
Synapse_PPI_Communities	Syn-3_0_2312	9475	ROCK2
Synapse_PPI_Communities	Syn-3_0_2312	10921	RNPS1
Synapse_PPI_Communities	Syn-3_0_2312	23170	TTLL12
Synapse_PPI_Communities	Syn-3_0_2312	213	ALB
Synapse_PPI_Communities	Syn-3_0_2312	9149	DYRK1B
Synapse_PPI_Communities	Syn-3_0_2312	23408	SIRT5
Synapse_PPI_Communities	Syn-3_0_2312	116540	MRPL53
Synapse_PPI_Communities	Syn-3_0_2312	5528	PPP2R5D
Synapse_PPI_Communities	Syn-3_0_2312	10250	SRRM1
Synapse_PPI_Communities	Syn-3_0_2312	3858	KRT10
Synapse_PPI_Communities	Syn-3_0_2312	2043	EPHA4
Synapse_PPI_Communities	Syn-3_0_2312	4915	NTRK2
Synapse_PPI_Communities	Syn-3_0_2312	3857	KRT9
Synapse_PPI_Communities	Syn-3_0_2312	4296	MAP3K11
Synapse_PPI_Communities	Syn-3_0_2312	84552	PARD6G
Synapse_PPI_Communities	Syn-3_0_2312	2011	MARK2
Synapse_PPI_Communities	Syn-3_0_2312	673	BRAF
Synapse_PPI_Communities	Syn-3_0_2312	1969	EPHA2
Synapse_PPI_Communities	Syn-3_0_2312	2495	FTH1
Synapse_PPI_Communities	Syn-3_0_2312	2029	ENSA
Synapse_PPI_Communities	Syn-3_0_2312	25913	POT1
Synapse_PPI_Communities	Syn-3_0_2312	65057	ACD
Synapse_PPI_Communities	Syn-3_0_2312	11214	AKAP13
Synapse_PPI_Communities	Syn-3_0_2312	5575	PRKAR1B
Synapse_PPI_Communities	Syn-3_0_2312	84817	TXNDC17
Synapse_PPI_Communities	Syn-3_0_2312	7171	TPM4
Synapse_PPI_Communities	Syn-3_0_2312	8936	WASF1
Synapse_PPI_Communities	Syn-3_0_2312	7791	ZYX
Synapse_PPI_Communities	Syn-3_0_2312	6462	SHBG
Synapse_PPI_Communities	Syn-3_0_2312	3741	KCNA5
Synapse_PPI_Communities	Syn-3_0_2312	8663	EIF3C
Synapse_PPI_Communities	Syn-3_0_2312	22941	SHANK2
Synapse_PPI_Communities	Syn-3_0_2312	22839	DLGAP4
Synapse_PPI_Communities	Syn-3_0_2312	6427	SRSF2
Synapse_PPI_Communities	Syn-3_0_2312	154796	AMOT
Synapse_PPI_Communities	Syn-3_0_2312	8411	EEA1

Synapse_PPI_Communities	Syn-3_0_2312	5337	PLD1
Synapse_PPI_Communities	Syn-3_0_2312	51517	NCKIPSD
Synapse_PPI_Communities	Syn-3_0_2312	8667	EIF3H
Synapse_PPI_Communities	Syn-3_0_2312	51765	MST4
Synapse_PPI_Communities	Syn-3_0_2312	1022	CDK7
Synapse_PPI_Communities	Syn-3_0_2312	156	ADRBK1
Synapse_PPI_Communities	Syn-3_0_2312	5903	RANBP2
Synapse_PPI_Communities	Syn-3_0_2312	1856	DVL2
Synapse_PPI_Communities	Syn-3_0_2312	58512	DLGAP3
Synapse_PPI_Communities	Syn-3_0_2312	1729	DIAPH1
Synapse_PPI_Communities	Syn-3_0_2312	164	AP1G1
Synapse_PPI_Communities	Syn-3_0_2312	1153	CIRBP
Synapse_PPI_Communities	Syn-3_0_2312	11091	WDR5
Synapse_PPI_Communities	Syn-3_0_2312	11321	GPN1
Synapse_PPI_Communities	Syn-3_0_2312	5906	RAP1A
Synapse_PPI_Communities	Syn-3_0_2312	4684	NCAM1
Synapse_PPI_Communities	Syn-3_0_2312	83737	ITCH
Synapse_PPI_Communities	Syn-3_0_2312	8470	SORBS2
Synapse_PPI_Communities	Syn-3_0_2312	29979	UBQLN1
Synapse_PPI_Communities	Syn-3_0_2312	5217	PFN2
Synapse_PPI_Communities	Syn-3_0_2312	2521	FUS
Synapse_PPI_Communities	Syn-3_0_2312	57062	DDX24
Synapse_PPI_Communities	Syn-3_0_2312	25939	SAMHD1
Synapse_PPI_Communities	Syn-3_0_2312	2241	FER
Synapse_PPI_Communities	Syn-3_0_2312	10772	SRSF10
Synapse_PPI_Communities	Syn-3_0_2312	7251	TSG101
Synapse_PPI_Communities	Syn-3_0_2312	6198	RPS6KB1
Synapse_PPI_Communities	Syn-3_0_2312	2617	GARS
Synapse_PPI_Communities	Syn-3_0_2312	8882	ZNF259
Synapse_PPI_Communities	Syn-3_0_2312	367	AR
Synapse_PPI_Communities	Syn-3_0_2312	60412	EXOC4
Synapse_PPI_Communities	Syn-3_0_2312	6169	RPL38
Synapse_PPI_Communities	Syn-3_0_2312	23451	SF3B1
Synapse_PPI_Communities	Syn-3_0_2312	7074	TIAM1
Synapse_PPI_Communities	Syn-3_0_2312	10048	RANBP9
Synapse_PPI_Communities	Syn-3_0_2312	6611	SMS
Synapse_PPI_Communities	Syn-3_0_2312	5757	PTMA
Synapse_PPI_Communities	Syn-3_0_2312	6625	SNRNP70
Synapse_PPI_Communities	Syn-3_0_2312	4172	MCM3
Synapse_PPI_Communities	Syn-3_0_2312	2534	FYN
Synapse_PPI_Communities	Syn-3_0_2312	9732	DOCK4
Synapse_PPI_Communities	Syn-3_0_2312	29888	STRN4
Synapse_PPI_Communities	Syn-3_0_2312	1933	EEF1B2
Synapse_PPI_Communities	Syn-3_0_2312	2810	SFN
Synapse_PPI_Communities	Syn-3_0_2312	26994	RNF11
Synapse_PPI_Communities	Syn-3_0_2312	10580	SORBS1
Synapse_PPI_Communities	Syn-3_0_2312	60626	RIC8A
Synapse_PPI_Communities	Syn-3_0_2312	56904	SH3GLB2
Synapse_PPI_Communities	Syn-3_0_2312	79876	UBA5
Synapse_PPI_Communities	Syn-3_0_2312	27342	RABGEF1
Synapse_PPI_Communities	Syn-3_0_2312	10534	SSSCA1
Synapse_PPI_Communities	Syn-3_0_2312	50618	ITSN2
Synapse_PPI_Communities	Syn-3_0_2312	6181	RPLP2
Synapse_PPI_Communities	Syn-3_0_2312	2058	EPRS
Synapse_PPI_Communities	Syn-3_0_2312	10006	ABI1
Synapse_PPI_Communities	Syn-3_0_2312	8522	GAS7
Synapse_PPI_Communities	Syn-3_0_2312	8502	PKP4
Synapse_PPI_Communities	Syn-3_0_2312	1003	CDH5

Synapse_PPI_Communities	Syn-3_0_2312	1832	DSP
Synapse_PPI_Communities	Syn-3_0_2312	6418	SET
Synapse_PPI_Communities	Syn-3_0_2312	24137	KIF4A
Synapse_PPI_Communities	Syn-3_0_2312	10657	KHDRBS1
Synapse_PPI_Communities	Syn-3_0_2312	1104	RCC1
Synapse_PPI_Communities	Syn-3_0_2312	3480	IGF1R
Synapse_PPI_Communities	Syn-3_0_2312	64210	MMS19
Synapse_PPI_Communities	Syn-3_0_2312	3006	HIST1H1C
Synapse_PPI_Communities	Syn-3_0_2312	9641	IKBKE
Synapse_PPI_Communities	Syn-3_0_2312	5129	CDK18
Synapse_PPI_Communities	Syn-3_0_2312	9252	RPS6KA5
Synapse_PPI_Communities	Syn-3_0_2312	1859	DYRK1A
Synapse_PPI_Communities	Syn-3_0_2312	8290	HIST3H3
Synapse_PPI_Communities	Syn-3_0_2312	6647	SOD1
Synapse_PPI_Communities	Syn-3_0_2312	6745	SSR1
Synapse_PPI_Communities	Syn-3_0_2312	10933	MORF4L1
Synapse_PPI_Communities	Syn-3_0_2312	124512	METTL23
Synapse_PPI_Communities	Syn-3_0_2312	55763	EXOC1
Synapse_PPI_Communities	Syn-3_0_2312	27185	DISC1
Synapse_PPI_Communities	Syn-3_0_2312	23028	KDM1A
Synapse_PPI_Communities	Syn-3_0_2312	1465	CSRP1
Synapse_PPI_Communities	Syn-3_0_2312	55114	ARHGAP17
Synapse_PPI_Communities	Syn-3_0_2312	4287	ATXN3
Synapse_PPI_Communities	Syn-3_0_2312	23154	NCDN
Synapse_PPI_Communities	Syn-3_0_2312	11140	CDC37
Synapse_PPI_Communities	Syn-3_0_2312	5460	POU5F1
Synapse_PPI_Communities	Syn-3_0_2312	6772	STAT1
Synapse_PPI_Communities	Syn-3_0_2312	7514	XPO1
Synapse_PPI_Communities	Syn-3_0_2312	9632	SEC24C
Synapse_PPI_Communities	Syn-3_0_2312	322	APBB1
Synapse_PPI_Communities	Syn-3_0_2312	5928	RBBP4
Synapse_PPI_Communities	Syn-3_0_2312	56893	UBQLN4
Synapse_PPI_Communities	Syn-3_0_2312	8665	EIF3F
Synapse_PPI_Communities	Syn-3_0_2312	7265	TTC1
Synapse_PPI_Communities	Syn-3_0_2312	5997	RGS2
Synapse_PPI_Communities	Syn-3_0_2312	23512	SUZ12
Synapse_PPI_Communities	Syn-3_0_2312	191	AHCY
Synapse_PPI_Communities	Syn-3_0_2312	5970	RELA
Synapse_PPI_Communities	Syn-3_0_2312	6236	RRAD
Synapse_PPI_Communities	Syn-3_0_2312	3856	KRT8
Synapse_PPI_Communities	Syn-3_0_2312	5213	PFKM
Synapse_PPI_Communities	Syn-3_0_2312	5093	PCBP1
Synapse_PPI_Communities	Syn-3_0_2312	29993	PACSIN1
Synapse_PPI_Communities	Syn-3_0_2312	57787	MARK4
Synapse_PPI_Communities	Syn-3_0_2312	5037	PEBP1
Synapse_PPI_Communities	Syn-3_0_2312	56647	BCCIP
Synapse_PPI_Communities	Syn-3_0_2312	3066	HDAC2
Synapse_PPI_Communities	Syn-3_0_2312	4763	NF1
Synapse_PPI_Communities	Syn-3_0_2312	7417	VDAC2
Synapse_PPI_Communities	Syn-3_0_2312	25813	SAMM50
Synapse_PPI_Communities	Syn-3_0_2312	993	CDC25A
Synapse_PPI_Communities	Syn-3_0_2312	57118	CAMK1D
Synapse_PPI_Communities	Syn-3_0_2312	331	XIAP
Synapse_PPI_Communities	Syn-3_0_2312	4089	SMAD4
Synapse_PPI_Communities	Syn-3_0_2312	291	SLC25A4
Synapse_PPI_Communities	Syn-3_0_2312	5295	PIK3R1
Synapse_PPI_Communities	Syn-3_0_2312	5499	PPP1CA
Synapse_PPI_Communities	Syn-3_0_2312	57379	AICDA

Synapse_PPI_Communities	Syn-3_0_2312	5300	PIN1
Synapse_PPI_Communities	Syn-3_0_2312	11161	C14orf1
Synapse_PPI_Communities	Syn-3_0_2312	4163	MCC
Synapse_PPI_Communities	Syn-3_0_2312	267	AMFR
Synapse_PPI_Communities	Syn-3_0_2312	23085	ERC1
Synapse_PPI_Communities	Syn-3_0_2312	11100	HNRNPUL1
Synapse_PPI_Communities	Syn-3_0_2312	23111	SPG20
Synapse_PPI_Communities	Syn-3_0_2312	9656	MDC1
Synapse_PPI_Communities	Syn-3_0_2312	6517	SLC2A4
Synapse_PPI_Communities	Syn-3_0_2312	10484	SEC23A
Synapse_PPI_Communities	Syn-3_0_2312	9114	ATP6V0D1
Synapse_PPI_Communities	Syn-3_0_2312	6259	RYK
Synapse_PPI_Communities	Syn-3_0_2312	4653	MYOC
Synapse_PPI_Communities	Syn-3_0_2312	3636	INPPL1
Synapse_PPI_Communities	Syn-3_0_2312	6774	STAT3
Synapse_PPI_Communities	Syn-3_0_2312	127247	ASB17
Synapse_PPI_Communities	Syn-3_0_2312	10409	BASP1
Synapse_PPI_Communities	Syn-3_0_2312	6310	ATXN1
Synapse_PPI_Communities	Syn-3_0_2312	3688	ITGB1
Synapse_PPI_Communities	Syn-3_0_2312	7082	TJP1
Synapse_PPI_Communities	Syn-3_0_2312	7525	YES1
Synapse_PPI_Communities	Syn-3_0_2312	5905	RANGAP1
Synapse_PPI_Communities	Syn-3_0_2312	8649	LAMTOR3
Synapse_PPI_Communities	Syn-3_0_2312	2547	XRCC6
Synapse_PPI_Communities	Syn-3_0_2312	23392	KIAA0368
Synapse_PPI_Communities	Syn-3_0_2312	7083	TK1
Synapse_PPI_Communities	Syn-3_0_2312	6606	SMN1
Synapse_PPI_Communities	Syn-3_0_2312	1601	DAB2
Synapse_PPI_Communities	Syn-3_0_2312	23122	CLASP2
Synapse_PPI_Communities	Syn-3_0_2312	5859	QARS
Synapse_PPI_Communities	Syn-3_0_2312	2078	ERG
Synapse_PPI_Communities	Syn-3_0_2312	648	BMI1
Synapse_PPI_Communities	Syn-3_0_2312	55738	ARFGAP1
Synapse_PPI_Communities	Syn-3_0_2312	4292	MLH1
Synapse_PPI_Communities	Syn-3_0_2312	2146	EZH2
Synapse_PPI_Communities	Syn-3_0_2312	10454	TAB1
Synapse_PPI_Communities	Syn-3_0_2312	5599	MAPK8
Synapse_PPI_Communities	Syn-3_0_2312	22976	PAXIP1
Synapse_PPI_Communities	Syn-3_0_2312	5597	MAPK6
Synapse_PPI_Communities	Syn-3_0_2312	7321	UBE2D1
Synapse_PPI_Communities	Syn-3_0_2312	6624	FSCN1
Synapse_PPI_Communities	Syn-3_0_2312	6696	SPP1
Synapse_PPI_Communities	Syn-3_0_2312	10524	KAT5
Synapse_PPI_Communities	Syn-3_0_2312	10856	RUVBL2
Synapse_PPI_Communities	Syn-3_0_2312	5105	PCK1
Synapse_PPI_Communities	Syn-3_0_2312	140462	ASB9
Synapse_PPI_Communities	Syn-3_0_2312	7520	XRCC5
Synapse_PPI_Communities	Syn-3_0_2312	10492	SYNCRIP
Synapse_PPI_Communities	Syn-3_0_2312	1654	DDX3X
Synapse_PPI_Communities	Syn-3_0_2312	1107	CHD3
Synapse_PPI_Communities	Syn-3_0_2312	1936	EEF1D
Synapse_PPI_Communities	Syn-3_0_2312	773	CACNA1A
Synapse_PPI_Communities	Syn-3_0_2312	6929	TCF3
Synapse_PPI_Communities	Syn-3_0_2312	1080	CFTR
Synapse_PPI_Communities	Syn-3_0_2312	10013	HDAC6
Synapse_PPI_Communities	Syn-3_0_2312	7189	TRAF6
Synapse_PPI_Communities	Syn-3_0_2312	4087	SMAD2
Synapse_PPI_Communities	Syn-3_0_2312	3181	HNRNPA2B1

Synapse_PPI_Communities	Syn-3_0_2312	4000	LMNA
Synapse_PPI_Communities	Syn-3_0_2312	6840	SVIL
Synapse_PPI_Communities	Syn-3_0_2312	55660	PRPF40A
Synapse_PPI_Communities	Syn-3_0_2312	59	ACTA2
Synapse_PPI_Communities	Syn-3_0_2312	3895	KTN1
Synapse_PPI_Communities	Syn-3_0_2312	3065	HDAC1
Synapse_PPI_Communities	Syn-3_0_2312	7277	TUBA4A
Synapse_PPI_Communities	Syn-3_0_2312	3396	ICT1
Synapse_PPI_Communities	Syn-3_0_2312	4282	MIF
Synapse_PPI_Communities	Syn-3_0_2312	79823	CAMKMT
Synapse_PPI_Communities	Syn-3_0_2312	805	CALM2
Synapse_PPI_Communities	Syn-3_0_2312	808	CALM3
Synapse_PPI_Communities	Syn-3_0_2312	672	BRCA1
Synapse_PPI_Communities	Syn-3_0_2312	8607	RUVBL1
Synapse_PPI_Communities	Syn-3_0_2312	3981	LIG4
Synapse_PPI_Communities	Syn-3_0_2312	1894	ECT2
Synapse_PPI_Communities	Syn-3_0_2312	6195	RPS6KA1
Synapse_PPI_Communities	Syn-3_0_2312	4478	MSN
Synapse_PPI_Communities	Syn-3_0_2312	1201	CLN3
Synapse_PPI_Communities	Syn-3_0_2312	3295	HSD17B4
Synapse_PPI_Communities	Syn-3_0_2312	6657	SOX2
Synapse_PPI_Communities	Syn-3_0_2312	5878	RAB5C
Synapse_PPI_Communities	Syn-3_0_2312	2950	GSTP1
Synapse_PPI_Communities	Syn-3_0_2312	57520	HECW2
Synapse_PPI_Communities	Syn-3_0_2312	4255	MGMT
Synapse_PPI_Communities	Syn-3_0_2312	324	APC
Synapse_PPI_Communities	Syn-3_0_2312	378	ARF4
Synapse_PPI_Communities	Syn-3_0_2312	476	ATP1A1
Synapse_PPI_Communities	Syn-3_0_2312	26277	TINF2
Synapse_PPI_Communities	Syn-3_0_2312	7014	TERF2
Synapse_PPI_Communities	Syn-3_0_2312	308	ANXA5
SZ_Risk	PGC2	1806	DPYD
SZ_Risk	PGC2	406928	MIR137
SZ_Risk	PGC2	403	ARL3
SZ_Risk	PGC2	57412	AS3MT
SZ_Risk	PGC2	119032	C10orf32
SZ_Risk	PGC2	54805	CNNM2
SZ_Risk	PGC2	1586	CYP17A1
SZ_Risk	PGC2	9118	INA
SZ_Risk	PGC2	22978	NT5C2
SZ_Risk	PGC2	84108	PCGF6
SZ_Risk	PGC2	22984	PDCD11
SZ_Risk	PGC2	118980	SFXN2
SZ_Risk	PGC2	6877	TAF5
SZ_Risk	PGC2	81603	TRIM8
SZ_Risk	PGC2	84833	USMG5
SZ_Risk	PGC2	54838	WBP1L
SZ_Risk	PGC2	775	CACNA1C
SZ_Risk	PGC2	203062	TSNARE1
SZ_Risk	PGC2	64116	SLC39A8
SZ_Risk	PGC2	8379	MAD1L1
SZ_Risk	PGC2	57688	ZSWIM6
SZ_Risk	PGC2	23457	ABCB9
SZ_Risk	PGC2	51329	ARL6IP4
SZ_Risk	PGC2	91574	C12orf65
SZ_Risk	PGC2	8099	CDK2AP1
SZ_Risk	PGC2	10198	MPHOSPH9
SZ_Risk	PGC2	79676	OGFOD2

SZ_Risk	PGC2	57605	PITPNM2
SZ_Risk	PGC2	196383	RILPL2
SZ_Risk	PGC2	55206	SBNO1
SZ_Risk	PGC2	387893	SETD8
SZ_Risk	PGC2	79568	C2orf47
SZ_Risk	PGC2	205327	C2orf69
SZ_Risk	PGC2	129450	TYW5
SZ_Risk	PGC2	2242	FES
SZ_Risk	PGC2	5045	FURIN
SZ_Risk	PGC2	4122	MAN2A2
SZ_Risk	PGC2	9881	TRANK1
SZ_Risk	PGC2	84334	APOPT1
SZ_Risk	PGC2	9529	BAG5
SZ_Risk	PGC2	1152	CKB
SZ_Risk	PGC2	3831	KLC1
SZ_Risk	PGC2	23368	PPP1R13B
SZ_Risk	PGC2	115708	TRMT61A
SZ_Risk	PGC2	7517	XRCC3
SZ_Risk	PGC2	79038	ZFYVE21
SZ_Risk	PGC2	1136	CHRNA3
SZ_Risk	PGC2	1138	CHRNA5
SZ_Risk	PGC2	1143	CHRNB4
SZ_Risk	PGC2	3658	IREB2
SZ_Risk	PGC2	5685	PSMA4
SZ_Risk	PGC2	83943	IMMP2L
SZ_Risk	PGC2	399979	SNX19
SZ_Risk	PGC2	91752	ZNF804A
SZ_Risk	PGC2	22866	CNKSR2
SZ_Risk	PGC2	783	CACNB2
SZ_Risk	PGC2	4035	LRP1
SZ_Risk	PGC2	4640	MYO1A
SZ_Risk	PGC2	4665	NAB2
SZ_Risk	PGC2	56901	NDUFA4L2
SZ_Risk	PGC2	11247	NXPH4
SZ_Risk	PGC2	22864	R3HDM2
SZ_Risk	PGC2	6472	SHMT2
SZ_Risk	PGC2	246329	STAC3
SZ_Risk	PGC2	6778	STAT6
SZ_Risk	PGC2	6866	TAC3
SZ_Risk	PGC2	23306	TMEM194A
SZ_Risk	PGC2	127255	LRRIQ3
SZ_Risk	PGC2	389084	C2orf82
SZ_Risk	PGC2	80303	EFHD1
SZ_Risk	PGC2	26058	GIGYF2
SZ_Risk	PGC2	3769	KCNJ13
SZ_Risk	PGC2	25791	NGEF
SZ_Risk	PGC2	90952	ESAM
SZ_Risk	PGC2	79684	MSANTD2
SZ_Risk	PGC2	4900	NRGN
SZ_Risk	PGC2	23584	VSIG2
SZ_Risk	PGC2	6925	TCF4
SZ_Risk	PGC2	55626	AMBRA1
SZ_Risk	PGC2	392	ARHGAP1
SZ_Risk	PGC2	9776	ATG13
SZ_Risk	PGC2	1132	CHRM4
SZ_Risk	PGC2	9793	CKAP5
SZ_Risk	PGC2	90993	CREB3L1
SZ_Risk	PGC2	8525	DGKZ

SZ_Risk	PGC2	2147	F2
SZ_Risk	PGC2	283254	HARBI1
SZ_Risk	PGC2	4192	MDK
SZ_Risk	PGC2	79797	ZNF408
SZ_Risk	PGC2	339829	CCDC39
SZ_Risk	PGC2	131118	DNAJC19
SZ_Risk	PGC2	8087	FXR1
SZ_Risk	PGC2	79913	ACTR5
SZ_Risk	PGC2	26051	PPP1R16B
SZ_Risk	PGC2	140679	SLC32A1
SZ_Risk	PGC2	55120	FANCL
SZ_Risk	PGC2	7444	VRK2
SZ_Risk	PGC2	57188	ADAMTSL3
SZ_Risk	PGC2	643707	GOLGA6L4
SZ_Risk	PGC2	54993	ZSCAN2
SZ_Risk	PGC2	91526	ANKRD44
SZ_Risk	PGC2	66037	BOLL
SZ_Risk	PGC2	80219	COQ10B
SZ_Risk	PGC2	3329	HSPD1
SZ_Risk	PGC2	3336	HSPE1
SZ_Risk	PGC2	92935	MARS2
SZ_Risk	PGC2	5334	PLCL1
SZ_Risk	PGC2	130132	RFTN2
SZ_Risk	PGC2	23451	SF3B1
SZ_Risk	PGC2	150356	CHADL
SZ_Risk	PGC2	2033	EP300
SZ_Risk	PGC2	83746	L3MBTL2
SZ_Risk	PGC2	5905	RANGAP1
SZ_Risk	PGC2	27012	KCNV1
SZ_Risk	PGC2	152330	CNTN4
SZ_Risk	PGC2	1813	DRD2
SZ_Risk	PGC2	22997	IGSF9B
SZ_Risk	PGC2	55830	GLT8D1
SZ_Risk	PGC2	26354	GNL3
SZ_Risk	PGC2	3697	ITIH1
SZ_Risk	PGC2	3699	ITIH3
SZ_Risk	PGC2	3700	ITIH4
SZ_Risk	PGC2	389125	MUSTN1
SZ_Risk	PGC2	6787	NEK4
SZ_Risk	PGC2	11188	NISCH
SZ_Risk	PGC2	64943	NT5DC2
SZ_Risk	PGC2	55193	PBRM1
SZ_Risk	PGC2	440957	SMIM4
SZ_Risk	PGC2	28972	SPCS1
SZ_Risk	PGC2	23166	STAB1
SZ_Risk	PGC2	375346	TMEM110
SZ_Risk	PGC2	100526772	TMEM110-MUSTN1
SZ_Risk	PGC2	226	ALDOA
SZ_Risk	PGC2	253982	ASPHD1
SZ_Risk	PGC2	146378	C16orf92
SZ_Risk	PGC2	8448	DOC2A
SZ_Risk	PGC2	83723	FAM57B
SZ_Risk	PGC2	79153	GDPD3
SZ_Risk	PGC2	8479	HIRIP3
SZ_Risk	PGC2	283899	INO80E
SZ_Risk	PGC2	253980	KCTD13
SZ_Risk	PGC2	5595	MAPK3
SZ_Risk	PGC2	5531	PPP4C

SZ_Risk	PGC2	26470	SEZ6L2
SZ_Risk	PGC2	9344	TAOK2
SZ_Risk	PGC2	6911	TBX6
SZ_Risk	PGC2	124446	TMEM219
SZ_Risk	PGC2	83719	YPEL3
SZ_Risk	PGC2	8911	CACNA1I
SZ_Risk	PGC2	55167	MSL2
SZ_Risk	PGC2	4690	NCK1
SZ_Risk	PGC2	5096	PCCB
SZ_Risk	PGC2	5523	PPP2R3A
SZ_Risk	PGC2	80723	SLC35G2
SZ_Risk	PGC2	10274	STAG1
SZ_Risk	PGC2	2890	GRIA1
SZ_Risk	PGC2	64219	PJA1
SZ_Risk	PGC2	9905	SGSM2
SZ_Risk	PGC2	23293	SMG6
SZ_Risk	PGC2	63826	SRR
SZ_Risk	PGC2	55720	TSR1
SZ_Risk	PGC2	2913	GRM3
SZ_Risk	PGC2	9682	KDM4A
SZ_Risk	PGC2	5792	PTPRF
SZ_Risk	PGC2	148113	CILP2
SZ_Risk	PGC2	54815	GATAD2A
SZ_Risk	PGC2	404037	HAPLN4
SZ_Risk	PGC2	23383	MAU2
SZ_Risk	PGC2	1463	NCAN
SZ_Risk	PGC2	51079	NDUFA13
SZ_Risk	PGC2	80714	PBX4
SZ_Risk	PGC2	57794	SUGP1
SZ_Risk	PGC2	53345	TM6SF2
SZ_Risk	PGC2	83983	TSSK6
SZ_Risk	PGC2	81611	ANP32E
SZ_Risk	PGC2	51107	APH1A
SZ_Risk	PGC2	148523	C1orf51
SZ_Risk	PGC2	79630	C1orf54
SZ_Risk	PGC2	23632	CA14
SZ_Risk	PGC2	56957	OTUD7B
SZ_Risk	PGC2	51177	PLEKHO1
SZ_Risk	PGC2	11311	VPS45
SZ_Risk	PGC2	9892	SNAP91
SZ_Risk	PGC2	9651	PLCH2
SZ_Risk	PGC2	2072	ERCC4
SZ_Risk	PGC2	54517	PUS7
SZ_Risk	PGC2	6733	SRPK2
SZ_Risk	PGC2	473	RERE
SZ_Risk	PGC2	50651	SLC45A1
SZ_Risk	PGC2	488	ATP2A2
SZ_Risk	PGC2	54969	C4orf27
SZ_Risk	PGC2	1182	CLCN3
SZ_Risk	PGC2	4750	NEK1
SZ_Risk	PGC2	10690	FUT9
SZ_Risk	PGC2	79019	CENPM
SZ_Risk	PGC2	1565	CYP2D6
SZ_Risk	PGC2	150368	FAM109B
SZ_Risk	PGC2	4668	NAGA
SZ_Risk	PGC2	4700	NDUFA6
SZ_Risk	PGC2	55964	SEPT3
SZ_Risk	PGC2	440829	SHISA8

SZ_Risk	PGC2	91689	SMDT1
SZ_Risk	PGC2	6721	SREBF2
SZ_Risk	PGC2	6942	TCF20
SZ_Risk	PGC2	115650	TNFRSF13C
SZ_Risk	PGC2	164684	WBP2NL
SZ_Risk	PGC2	643376	BTBD18
SZ_Risk	PGC2	280636	C11orf31
SZ_Risk	PGC2	10978	CLP1
SZ_Risk	PGC2	1500	CTNND1
SZ_Risk	PGC2	219541	MED19
SZ_Risk	PGC2	710	SERPING1
SZ_Risk	PGC2	51075	TMX2
SZ_Risk	PGC2	219539	YPEL4
SZ_Risk	PGC2	25921	ZDHHC5
SZ_Risk	PGC2	338645	LUZP2
SZ_Risk	PGC2	9162	DGKI
SZ_Risk	PGC2	5764	PTN
SZ_Risk	PGC2	7088	TLE1
SZ_Risk	PGC2	10000	AKT3
SZ_Risk	PGC2	10806	SDCCAG8
SZ_Risk	PGC2	100131244	ANKRD63
SZ_Risk	PGC2	56924	PAK6
SZ_Risk	PGC2	5330	PLCB2
SZ_Risk	PGC2	9745	ZNF536
SZ_Risk	PGC2	4208	MEF2C
SZ_Risk	PGC2	9779	TBC1D5
SZ_Risk	PGC2	995	CDC25C
SZ_Risk	PGC2	1495	CTNNA1
SZ_Risk	PGC2	1958	EGR1
SZ_Risk	PGC2	2107	ETF1
SZ_Risk	PGC2	51307	FAM53C
SZ_Risk	PGC2	2676	GFRA3
SZ_Risk	PGC2	3313	HSPA9
SZ_Risk	PGC2	51780	KDM3B
SZ_Risk	PGC2	51308	REEP2
SZ_Risk	PGC2	64919	BCL11B
SZ_Risk	PGC2	9628	RGS6
SZ_Risk	PGC2	348980	HCN1
SZ_Risk	PGC2	767	CA8
SZ_Risk	PGC2	56603	CYP26B1
SZ_Risk	PGC2	57476	GRAMD1B
SZ_Risk	PGC2	23314	SATB2
SZ_Risk	PGC2	64002	PCGEM1
SZ_Risk	PGC2	2823	GPM6A
SZ_Risk	PGC2	64478	CSMD1
SZ_Risk	PGC2	8452	CUL3
SZ_Risk	PGC2	4325	MMP16
SZ_Risk	PGC2	2903	GRIN2A
SZ_Risk	PGC2	5587	PRKD1
SZ_Risk	PGC2	6314	ATXN7
SZ_Risk	PGC2	132200	C3orf49
SZ_Risk	PGC2	9861	PSMD6
SZ_Risk	PGC2	80145	THOC7
SZ_Risk	PGC2	65057	ACD
SZ_Risk	PGC2	388284	C16orf86
SZ_Risk	PGC2	80152	CENPT
SZ_Risk	PGC2	1506	CTRL
SZ_Risk	PGC2	55794	DDX28

SZ_Risk	PGC2	64174	DPEP2
SZ_Risk	PGC2	64180	DPEP3
SZ_Risk	PGC2	23644	EDC4
SZ_Risk	PGC2	84080	ENKD1
SZ_Risk	PGC2	80004	ESRP2
SZ_Risk	PGC2	81577	GFOD2
SZ_Risk	PGC2	3931	LCAT
SZ_Risk	PGC2	4775	NFATC3
SZ_Risk	PGC2	123904	NRN1L
SZ_Risk	PGC2	10204	NUTF2
SZ_Risk	PGC2	50855	PARD6A
SZ_Risk	PGC2	23659	PLA2G15
SZ_Risk	PGC2	5681	PSKH1
SZ_Risk	PGC2	5699	PSMB10
SZ_Risk	PGC2	57610	RANBP10
SZ_Risk	PGC2	6560	SLC12A4
SZ_Risk	PGC2	9057	SLC7A6
SZ_Risk	PGC2	84138	SLC7A6OS
SZ_Risk	PGC2	57215	THAP11
SZ_Risk	PGC2	55815	TSNAXIP1
SZ_Risk	PGC2	26122	EPC2
SZ_Risk	PGC2	91647	ATPAF2
SZ_Risk	PGC2	1819	DRG2
SZ_Risk	PGC2	79018	GID4
SZ_Risk	PGC2	83450	LRRC48
SZ_Risk	PGC2	51168	MYO15A
SZ_Risk	PGC2	10743	RAI1
SZ_Risk	PGC2	6720	SREBF1
SZ_Risk	PGC2	146691	TOM1L2
SZ_Risk	PGC2	7090	TLE3
SZ_Risk	PGC2	23019	CNOT1
SZ_Risk	PGC2	55238	SLC38A7
SZ_Risk	PGC2	1191	CLU
SZ_Risk	PGC2	2053	EPHX2
SZ_Risk	PGC2	57502	NLGN4X
SZ_Risk	PGC2	22999	RIMS1
SZ_Risk	PGC2	1687	DFNA5
SZ_Risk	PGC2	51678	MPP6
SZ_Risk	PGC2	26031	OSBPL3
SZ_Risk	PGC2	4124	MAN2A1
SZ_Risk	PGC2	100616252	MIR548AJ2
SZ_Risk	PGC2	55568	GALNT10
SZ_Risk	PGC2	399947	C11orf87
SZ_Risk	PGC2	83857	TMTC1
SZ_Risk	PGC2	5420	PODXL
SZ_Risk	PGC2	100128537	C1orf132
SZ_Risk	PGC2	4179	CD46
SZ_Risk	PGC2	1379	CR1L
SZ_Risk	PGC2	3745	KCNB1
SZ_Risk	PGC2	5740	PTGIS
SZ_Risk	PGC2	256021	C12orf79
SZ_Risk	PGC2	1803	DPP4
SZ_Risk	PGC2	57282	SLC4A10
SZ_Risk	PGC2	51070	NOSIP
SZ_Risk	PGC2	57479	PRR12
SZ_Risk	PGC2	5639	PRRG2
SZ_Risk	PGC2	57333	RCN3
SZ_Risk	PGC2	6237	RRAS

SZ_Risk	PGC2	58506	SCAF1
SZ_Risk	PGC2	374470	C12orf42
SZ_Risk	PGC2	929	CD14
SZ_Risk	PGC2	373863	DND1
SZ_Risk	PGC2	3035	HARS
SZ_Risk	PGC2	23438	HARS2
SZ_Risk	PGC2	3550	IK
SZ_Risk	PGC2	4695	NDUFA2
SZ_Risk	PGC2	56147	PCDHA1
SZ_Risk	PGC2	56139	PCDHA10
SZ_Risk	PGC2	56146	PCDHA2
SZ_Risk	PGC2	56145	PCDHA3
SZ_Risk	PGC2	56144	PCDHA4
SZ_Risk	PGC2	56143	PCDHA5
SZ_Risk	PGC2	56142	PCDHA6
SZ_Risk	PGC2	56141	PCDHA7
SZ_Risk	PGC2	56140	PCDHA8
SZ_Risk	PGC2	9752	PCDHA9
SZ_Risk	PGC2	55374	TMCO6
SZ_Risk	PGC2	54853	WDR55
SZ_Risk	PGC2	153527	ZMAT2
SZ_Risk	PGC2_permissive	80317	ZKSCAN3
SZ_Risk	PGC2_permissive	9753	ZSCAN12
SZ_Risk	PGC2_permissive	222696	ZSCAN23
SZ_Risk	PGC2_permissive	2880	GPX5
SZ_Risk	PGC2_permissive	114821	SCAND3
SZ_Risk	PGC2_permissive	54838	WBP1L
SZ_Risk	PGC2_permissive	1586	CYP17A1
SZ_Risk	PGC2_permissive	119032	C10orf32
SZ_Risk	PGC2_permissive	100528007	C10orf32-AS3MT
SZ_Risk	PGC2_permissive	57412	AS3MT
SZ_Risk	PGC2_permissive	54805	CNNM2
SZ_Risk	PGC2_permissive	22978	NT5C2
SZ_Risk	PGC2_permissive	775	CACNA1C
SZ_Risk	PGC2_permissive	100874234	CACNA1C-AS4
SZ_Risk	PGC2_permissive	100874370	CACNA1C-IT3
SZ_Risk	PGC2_permissive	1806	DPYD
SZ_Risk	PGC2_permissive	400765	MIR137HG
SZ_Risk	PGC2_permissive	100616452	MIR2682
SZ_Risk	PGC2_permissive	406928	MIR137
SZ_Risk	PGC2_permissive	205327	C2orf69
SZ_Risk	PGC2_permissive	129450	TYW5
SZ_Risk	PGC2_permissive	79568	C2orf47
SZ_Risk	PGC2_permissive	23457	ABCB9
SZ_Risk	PGC2_permissive	79676	OGFOD2
SZ_Risk	PGC2_permissive	51329	ARL6IP4
SZ_Risk	PGC2_permissive	57605	PITPNM2
SZ_Risk	PGC2_permissive	100422931	MIR4304
SZ_Risk	PGC2_permissive	100507091	LOC100507091
SZ_Risk	PGC2_permissive	10198	MPHOSPH9
SZ_Risk	PGC2_permissive	91574	C12orf65
SZ_Risk	PGC2_permissive	8099	CDK2AP1
SZ_Risk	PGC2_permissive	55206	SBNO1
SZ_Risk	PGC2_permissive	387893	SETD8
SZ_Risk	PGC2_permissive	196383	RILPL2
SZ_Risk	PGC2_permissive	8379	MAD1L1
SZ_Risk	PGC2_permissive	100616160	MIR4655
SZ_Risk	PGC2_permissive	1152	CKB

SZ_Risk	PGC2_permissive	115708	TRMT61A
SZ_Risk	PGC2_permissive	9529	BAG5
SZ_Risk	PGC2_permissive	84334	APOPT1
SZ_Risk	PGC2_permissive	3831	KLC1
SZ_Risk	PGC2_permissive	7517	XRCC3
SZ_Risk	PGC2_permissive	79038	ZFYVE21
SZ_Risk	PGC2_permissive	23368	PPP1R13B
SZ_Risk	PGC2_permissive	57688	ZSWIM6
SZ_Risk	PGC2_permissive	619434	LINC00051
SZ_Risk	PGC2_permissive	203062	TSNARE1
SZ_Risk	PGC2_permissive	83943	IMMP2L
SZ_Risk	PGC2_permissive	6925	TCF4
SZ_Risk	PGC2_permissive	64116	SLC39A8
SZ_Risk	PGC2_permissive	3658	IREB2
SZ_Risk	PGC2_permissive	5685	PSMA4
SZ_Risk	PGC2_permissive	1138	CHRNA5
SZ_Risk	PGC2_permissive	1136	CHRNA3
SZ_Risk	PGC2_permissive	1143	CHRNB4
SZ_Risk	PGC2_permissive	5045	FURIN
SZ_Risk	PGC2_permissive	2242	FES
SZ_Risk	PGC2_permissive	4122	MAN2A2
SZ_Risk	PGC2_permissive	399979	SNX19
SZ_Risk	PGC2_permissive	80303	EFHD1
SZ_Risk	PGC2_permissive	26058	GIGYF2
SZ_Risk	PGC2_permissive	3769	KCNJ13
SZ_Risk	PGC2_permissive	389084	C2orf82
SZ_Risk	PGC2_permissive	25791	NGEF
SZ_Risk	PGC2_permissive	9881	TRANK1
SZ_Risk	PGC2_permissive	4900	NRGN
SZ_Risk	PGC2_permissive	23584	VSIG2
SZ_Risk	PGC2_permissive	90952	ESAM
SZ_Risk	PGC2_permissive	79684	MSANTD2
SZ_Risk	PGC2_permissive	4035	LRP1
SZ_Risk	PGC2_permissive	100302201	MIR1228
SZ_Risk	PGC2_permissive	11247	NXPH4
SZ_Risk	PGC2_permissive	6472	SHMT2
SZ_Risk	PGC2_permissive	56901	NDUFA4L2
SZ_Risk	PGC2_permissive	246329	STAC3
SZ_Risk	PGC2_permissive	22864	R3HDM2
SZ_Risk	PGC2_permissive	100302237	MIR1281
SZ_Risk	PGC2_permissive	2033	EP300
SZ_Risk	PGC2_permissive	83746	L3MBTL2
SZ_Risk	PGC2_permissive	150356	CHADL
SZ_Risk	PGC2_permissive	5905	RANGAP1
SZ_Risk	PGC2_permissive	91752	ZNF804A
SZ_Risk	PGC2_permissive	22997	IGSF9B
SZ_Risk	PGC2_permissive	91526	ANKRD44
SZ_Risk	PGC2_permissive	23451	SF3B1
SZ_Risk	PGC2_permissive	80219	COQ10B
SZ_Risk	PGC2_permissive	3329	HSPD1
SZ_Risk	PGC2_permissive	3336	HSPE1
SZ_Risk	PGC2_permissive	100529241	HSPE1-MOB4
SZ_Risk	PGC2_permissive	25843	MOB4
SZ_Risk	PGC2_permissive	130132	RFTN2
SZ_Risk	PGC2_permissive	92935	MARS2
SZ_Risk	PGC2_permissive	66037	BOLL
SZ_Risk	PGC2_permissive	5334	PLCL1
SZ_Risk	PGC2_permissive	90993	CREB3L1

SZ_Risk	PGC2_permissive	8525	DGKZ
SZ_Risk	PGC2_permissive	100616368	MIR4688
SZ_Risk	PGC2_permissive	4192	MDK
SZ_Risk	PGC2_permissive	1132	CHRM4
SZ_Risk	PGC2_permissive	55626	AMBRA1
SZ_Risk	PGC2_permissive	100422827	MIR3160-1
SZ_Risk	PGC2_permissive	100422825	MIR3160-2
SZ_Risk	PGC2_permissive	283254	HARBI1
SZ_Risk	PGC2_permissive	9776	ATG13
SZ_Risk	PGC2_permissive	392	ARHGAP1
SZ_Risk	PGC2_permissive	79797	ZNF408
SZ_Risk	PGC2_permissive	2147	F2
SZ_Risk	PGC2_permissive	9793	CKAP5
SZ_Risk	PGC2_permissive	8911	CACNA1I
SZ_Risk	PGC2_permissive	783	CACNB2
SZ_Risk	PGC2_permissive	11188	NISCH
SZ_Risk	PGC2_permissive	23166	STAB1
SZ_Risk	PGC2_permissive	64943	NT5DC2
SZ_Risk	PGC2_permissive	55193	PBRM1
SZ_Risk	PGC2_permissive	26354	GNL3
SZ_Risk	PGC2_permissive	692089	SNORD19
SZ_Risk	PGC2_permissive	100113381	SNORD19B
SZ_Risk	PGC2_permissive	692109	SNORD69
SZ_Risk	PGC2_permissive	55830	GLT8D1
SZ_Risk	PGC2_permissive	28972	SPCS1
SZ_Risk	PGC2_permissive	6787	NEK4
SZ_Risk	PGC2_permissive	3697	ITIH1
SZ_Risk	PGC2_permissive	3699	ITIH3
SZ_Risk	PGC2_permissive	3700	ITIH4
SZ_Risk	PGC2_permissive	389125	MUSTN1
SZ_Risk	PGC2_permissive	100526772	TMEM110-MUSTN1
SZ_Risk	PGC2_permissive	375346	TMEM110
SZ_Risk	PGC2_permissive	152330	CNTN4
SZ_Risk	PGC2_permissive	5523	PPP2R3A
SZ_Risk	PGC2_permissive	55167	MSL2
SZ_Risk	PGC2_permissive	5096	PCCB
SZ_Risk	PGC2_permissive	10274	STAG1
SZ_Risk	PGC2_permissive	80723	SLC35G2
SZ_Risk	PGC2_permissive	4690	NCK1
SZ_Risk	PGC2_permissive	8087	FXR1
SZ_Risk	PGC2_permissive	131118	DNAJC19
SZ_Risk	PGC2_permissive	140679	SLC32A1
SZ_Risk	PGC2_permissive	79913	ACTR5
SZ_Risk	PGC2_permissive	26051	PPP1R16B
SZ_Risk	PGC2_permissive	81603	TRIM8
SZ_Risk	PGC2_permissive	403	ARL3
SZ_Risk	PGC2_permissive	57188	ADAMTSL3
SZ_Risk	PGC2_permissive	100505679	LOC100505679
SZ_Risk	PGC2_permissive	440300	LOC440300
SZ_Risk	PGC2_permissive	388152	LOC388152
SZ_Risk	PGC2_permissive	643707	GOLGA6L4
SZ_Risk	PGC2_permissive	440299	DNM1P41
SZ_Risk	PGC2_permissive	374650	GOLGA6L5
SZ_Risk	PGC2_permissive	388165	UBE2Q2P1
SZ_Risk	PGC2_permissive	54993	ZSCAN2
SZ_Risk	PGC2_permissive	2913	GRM3
SZ_Risk	PGC2_permissive	26470	SEZ6L2
SZ_Risk	PGC2_permissive	253982	ASPHD1

SZ_Risk	PGC2_permissive	253980	KCTD13
SZ_Risk	PGC2_permissive	124446	TMEM219
SZ_Risk	PGC2_permissive	9344	TAOK2
SZ_Risk	PGC2_permissive	8479	HIRIP3
SZ_Risk	PGC2_permissive	283899	INO80E
SZ_Risk	PGC2_permissive	8448	DOC2A
SZ_Risk	PGC2_permissive	146378	C16orf92
SZ_Risk	PGC2_permissive	83723	FAM57B
SZ_Risk	PGC2_permissive	226	ALDOA
SZ_Risk	PGC2_permissive	5531	PPP4C
SZ_Risk	PGC2_permissive	6911	TBX6
SZ_Risk	PGC2_permissive	83719	YPEL3
SZ_Risk	PGC2_permissive	79153	GDPD3
SZ_Risk	PGC2_permissive	5595	MAPK3
SZ_Risk	PGC2_permissive	1813	DRD2
SZ_Risk	PGC2_permissive	100422855	MIR4301
SZ_Risk	PGC2_permissive	5792	PTPRF
SZ_Risk	PGC2_permissive	9682	KDM4A
SZ_Risk	PGC2_permissive	488	ATP2A2
SZ_Risk	PGC2_permissive	9892	SNAP91
SZ_Risk	PGC2_permissive	56957	OTUD7B
SZ_Risk	PGC2_permissive	11311	VPS45
SZ_Risk	PGC2_permissive	51177	PLEKHO1
SZ_Risk	PGC2_permissive	81611	ANP32E
SZ_Risk	PGC2_permissive	23632	CA14
SZ_Risk	PGC2_permissive	51107	APH1A
SZ_Risk	PGC2_permissive	79630	C1orf54
SZ_Risk	PGC2_permissive	148523	C1orf51
SZ_Risk	PGC2_permissive	23293	SMG6
SZ_Risk	PGC2_permissive	63826	SRR
SZ_Risk	PGC2_permissive	55720	TSR1
SZ_Risk	PGC2_permissive	692208	SNORD91B
SZ_Risk	PGC2_permissive	692207	SNORD91A
SZ_Risk	PGC2_permissive	55964	SEPT3
SZ_Risk	PGC2_permissive	164684	WBP2NL
SZ_Risk	PGC2_permissive	4668	NAGA
SZ_Risk	PGC2_permissive	150368	FAM109B
SZ_Risk	PGC2_permissive	4700	NDUFA6
SZ_Risk	PGC2_permissive	1565	CYP2D6
SZ_Risk	PGC2_permissive	1564	CYP2D7P1
SZ_Risk	PGC2_permissive	6942	TCF20
SZ_Risk	PGC2_permissive	388906	LOC388906
SZ_Risk	PGC2_permissive	9628	RGS6
SZ_Risk	PGC2_permissive	57476	GRAMD1B
SZ_Risk	PGC2_permissive	50651	SLC45A1
SZ_Risk	PGC2_permissive	473	RERE
SZ_Risk	PGC2_permissive	6733	SRPK2
SZ_Risk	PGC2_permissive	9745	ZNF536
SZ_Risk	PGC2_permissive	9651	PLCH2
SZ_Risk	PGC2_permissive	10806	SDCCAG8
SZ_Risk	PGC2_permissive	2107	ETF1
SZ_Risk	PGC2_permissive	3313	HSPA9
SZ_Risk	PGC2_permissive	26785	SNORD63
SZ_Risk	PGC2_permissive	2903	GRIN2A
SZ_Risk	PGC2_permissive	10743	RAI1
SZ_Risk	PGC2_permissive	6720	SREBF1
SZ_Risk	PGC2_permissive	693120	MIR33B
SZ_Risk	PGC2_permissive	146691	TOM1L2

SZ_Risk	PGC2_permissive	83450	LRRC48
SZ_Risk	PGC2_permissive	91647	ATPAF2
SZ_Risk	PGC2_permissive	79018	GID4
SZ_Risk	PGC2_permissive	1819	DRG2
SZ_Risk	PGC2_permissive	51168	MYO15A
SZ_Risk	PGC2_permissive	1463	NCAN
SZ_Risk	PGC2_permissive	404037	HAPLN4
SZ_Risk	PGC2_permissive	53345	TM6SF2
SZ_Risk	PGC2_permissive	57794	SUGP1
SZ_Risk	PGC2_permissive	23383	MAU2
SZ_Risk	PGC2_permissive	54815	GATAD2A
SZ_Risk	PGC2_permissive	83983	TSSK6
SZ_Risk	PGC2_permissive	51079	NDUFA13
SZ_Risk	PGC2_permissive	374887	YJEFN3
SZ_Risk	PGC2_permissive	148113	CILP2
SZ_Risk	PGC2_permissive	80714	PBX4
SZ_Risk	PGC2_permissive	5764	PTN
SZ_Risk	PGC2_permissive	9162	DGKI
SZ_Risk	PGC2_permissive	7444	VRK2
SZ_Risk	PGC2_permissive	55120	FANCL
SZ_Risk	PGC2_permissive	4750	NEK1
SZ_Risk	PGC2_permissive	1182	CLCN3
SZ_Risk	PGC2_permissive	54969	C4orf27
SZ_Risk	PGC2_permissive	100616290	MIR4529
SZ_Risk	PGC2_permissive	8452	CUL3
SZ_Risk	PGC2_permissive	2823	GPM6A
SZ_Risk	PGC2_permissive	23019	CNOT1
SZ_Risk	PGC2_permissive	55238	SLC38A7
SZ_Risk	PGC2_permissive	79144	PPDPF
SZ_Risk	PGC2_permissive	5753	PTK6
SZ_Risk	PGC2_permissive	6725	SRMS
SZ_Risk	PGC2_permissive	100616252	MIR548AJ2
SZ_Risk	PGC2_permissive	348980	HCN1
SZ_Risk	PGC2_permissive	64919	BCL11B
SZ_Risk	PGC2_permissive	729020	LOC729020
SZ_Risk	PGC2_permissive	9118	INA
SZ_Risk	PGC2_permissive	84108	PCGF6
SZ_Risk	PGC2_permissive	6877	TAF5
SZ_Risk	PGC2_permissive	84833	USMG5
SZ_Risk	PGC2_permissive	100302174	MIR1307
SZ_Risk	PGC2_permissive	22984	PDCD11
SZ_Risk	PGC2_permissive	10690	FUT9
SZ_Risk	PGC2_permissive	100616343	MIR4677
SZ_Risk	PGC2_permissive	4325	MMP16
SZ_Risk	PGC2_permissive	2053	EPHX2
SZ_Risk	PGC2_permissive	1191	CLU
SZ_Risk	PGC2_permissive	286042	FAM86B3P
SZ_Risk	PGC2_permissive	157285	SGK223
SZ_Risk	PGC2_permissive	2676	GFRA3
SZ_Risk	PGC2_permissive	995	CDC25C
SZ_Risk	PGC2_permissive	51307	FAM53C
SZ_Risk	PGC2_permissive	51780	KDM3B
SZ_Risk	PGC2_permissive	132200	C3orf49
SZ_Risk	PGC2_permissive	80145	THOC7
SZ_Risk	PGC2_permissive	6314	ATXN7
SZ_Risk	PGC2_permissive	100507062	PSMD6-AS2
SZ_Risk	PGC2_permissive	9861	PSMD6
SZ_Risk	PGC2_permissive	26122	EPC2

SZ_Risk	PGC2_permissive	64478	CSMD1
SZ_Risk	PGC2_permissive	10000	AKT3
SZ_Risk	PGC2_permissive	146206	RLTPR
SZ_Risk	PGC2_permissive	65057	ACD
SZ_Risk	PGC2_permissive	50855	PARD6A
SZ_Risk	PGC2_permissive	388284	C16orf86
SZ_Risk	PGC2_permissive	81577	GFOD2
SZ_Risk	PGC2_permissive	57610	RANBP10
SZ_Risk	PGC2_permissive	55815	TSNAXIP1
SZ_Risk	PGC2_permissive	80152	CENPT
SZ_Risk	PGC2_permissive	57215	THAP11
SZ_Risk	PGC2_permissive	10204	NUTF2
SZ_Risk	PGC2_permissive	23644	EDC4
SZ_Risk	PGC2_permissive	123904	NRN1L
SZ_Risk	PGC2_permissive	5681	PSKH1
SZ_Risk	PGC2_permissive	1506	CTRL
SZ_Risk	PGC2_permissive	5699	PSMB10
SZ_Risk	PGC2_permissive	3931	LCAT
SZ_Risk	PGC2_permissive	6560	SLC12A4
SZ_Risk	PGC2_permissive	64180	DPEP3
SZ_Risk	PGC2_permissive	64174	DPEP2
SZ_Risk	PGC2_permissive	55794	DDX28
SZ_Risk	PGC2_permissive	4775	NFATC3
SZ_Risk	PGC2_permissive	80004	ESRP2
SZ_Risk	PGC2_permissive	23659	PLA2G15
SZ_Risk	PGC2_permissive	9057	SLC7A6
SZ_Risk	PGC2_permissive	2309	FOXO3
SZ_Risk	PGC2_permissive	23314	SATB2
SZ_Risk	PGC2_permissive	100505474	LOC100505474
SZ_Risk	PGC2_permissive	55568	GALNT10
SZ_Risk	PGC2_permissive	100616347	MIR548AI
SZ_Risk	PGC2_permissive	5587	PRKD1
SZ_Risk	PGC2_permissive	2272	FHIT
SZ_Risk	PGC2_permissive	57282	SLC4A10
SZ_Risk	PGC2_permissive	1803	DPP4
SZ_Risk	PGC2_permissive	55552	ZNF823
SZ_Risk	PGC2_permissive	56924	PAK6
SZ_Risk	PGC2_permissive	100131244	ANKRD63
SZ_Risk	PGC2_permissive	5330	PLCB2
SZ_Risk	PGC2_permissive	6721	SREBF2
SZ_Risk	PGC2_permissive	407039	MIR33A
SZ_Risk	PGC2_permissive	440829	SHISA8
SZ_Risk	PGC2_permissive	115650	TNFRSF13C
SZ_Risk	PGC2_permissive	79019	CENPM
SZ_Risk	PGC2_permissive	339674	LINC00634
SZ_Risk	PGC2_permissive	9779	TBC1D5
SZ_Risk	PGC2_permissive	64506	CPEB1
SZ_Risk	PGC2_permissive	283692	LOC283692
SZ_Risk	PGC2_permissive	8120	AP3B2
SZ_Risk	PGC2_permissive	338963	LOC338963
SZ_Risk	PGC2_permissive	219790	RTKN2
SZ_Risk	PGC2_permissive	710	SERPING1
SZ_Risk	PGC2_permissive	406919	MIR130A
SZ_Risk	PGC2_permissive	219539	YPEL4
SZ_Risk	PGC2_permissive	10978	CLP1
SZ_Risk	PGC2_permissive	25921	ZDHHC5
SZ_Risk	PGC2_permissive	219541	MED19
SZ_Risk	PGC2_permissive	51075	TMX2

SZ_Risk	PGC2_permissive	100528016	TMX2-CTNND1
SZ_Risk	PGC2_permissive	643376	BTBD18
SZ_Risk	PGC2_permissive	1500	CTNND1
SZ_Risk	PGC2_permissive	22999	RIMS1
SZ_Risk	PGC2_permissive	83857	TMTC1
SZ_Risk	PGC2_permissive	90342	FER1L5
SZ_Risk	PGC2_permissive	81562	LMAN2L
SZ_Risk	PGC2_permissive	26504	CNNM4
SZ_Risk	PGC2_permissive	100422928	MIR3127
SZ_Risk	PGC2_permissive	26505	CNNM3
SZ_Risk	PGC2_permissive	200539	ANKRD23
SZ_Risk	PGC2_permissive	51239	ANKRD39
SZ_Risk	PGC2_permissive	54910	SEMA4C
SZ_Risk	PGC2_permissive	51252	FAM178B
SZ_Risk	PGC2_permissive	344558	SH3RF3
SZ_Risk	PGC2_permissive	151011	SEPT10
SZ_Risk	PGC2_permissive	65124	SOWAHC
SZ_Risk	PGC2_permissive	1054	CEBPG
SZ_Risk	PGC2_permissive	5184	PEPD
SZ_Risk	PGC2_permissive	7840	ALMS1
SZ_Risk	PGC2_permissive	9027	NAT8
SZ_Risk	PGC2_permissive	200420	ALMS1P
SZ_Risk	PGC2_permissive	645323	LINC00461
SZ_Risk	PGC2_permissive	407047	MIR9-2
SZ_Risk	PGC2_permissive	4208	MEF2C
SZ_Risk	PGC2_permissive	389549	FEZF1
SZ_Risk	PGC2_permissive	154860	FEZF1-AS1
SZ_Risk	PGC2_permissive	93664	CADPS2
SZ_Risk	PGC2_permissive	9553	MRPL33
SZ_Risk	PGC2_permissive	64080	RBKS
SZ_Risk	PGC2_permissive	100302650	BRE-AS1
SZ_Risk	PGC2_permissive	9577	BRE
SZ_Risk	PGC2_permissive	100422965	MIR4263
SZ_Risk	PGC2_permissive	4124	MAN2A1
SZ_Risk	PGC2_permissive	100289673	LOC100289673
SZ_Risk	PGC2_permissive	29123	ANKRD11
SZ_Risk	PGC2_permissive	6687	SPG7
SZ_Risk	PGC2_permissive	6137	RPL13
SZ_Risk	PGC2_permissive	606500	SNORD68
SZ_Risk	PGC2_permissive	51201	ZDHHC2
SZ_Risk	PGC2_permissive	3745	KCNB1
SZ_Risk	PGC2_permissive	5740	PTGIS
SZ_Risk	PGC2_permissive	27086	FOXP1
SZ_Risk	PGC2_permissive	56603	CYP26B1
SZ_Risk	PGC2_permissive	22979	EFR3B
SZ_Risk	PGC2_permissive	222223	KIAA1324L
SZ_Risk	PGC2_permissive	9988	DMTF1
SZ_Risk	PGC2_permissive	11257	TP53TG1
SZ_Risk	PGC2_permissive	55714	TENM3
SZ_Risk	PGC2_permissive	100873932	DPYD-AS1
SZ_Risk	PGC2_permissive	5521	PPP2R2B
SZ_Risk	PGC2_permissive	100874361	PPP2R2B-IT1
SZ_Risk	PGC2_permissive	280655	IGBP1P1
SZ_Risk	PGC2_permissive	6729	SRP54
SZ_Risk	PGC2_permissive	283635	FAM177A1
SZ_Risk	PGC2_permissive	55012	PPP2R3C
SZ_Risk	PGC2_permissive	9692	KIAA0391
SZ_Risk	PGC2_permissive	5687	PSMA6

SZ_Risk	PGC2_permissive	2016	EMX1
SZ_Risk	PGC2_permissive	94097	SFXN5
SZ_Risk	PGC2_permissive	22990	PCNX
SZ_Risk	PGC2_permissive	1379	CR1L
SZ_Risk	PGC2_permissive	4179	CD46
SZ_Risk	PGC2_permissive	407026	MIR29C
SZ_Risk	PGC2_permissive	407025	MIR29B2
SZ_Risk	PGC2_permissive	148696	LOC148696
SZ_Risk	PGC2_permissive	90025	UBE3D
SZ_Risk	PGC2_permissive	23033	DOPEY1
SZ_Risk	PGC2_permissive	5238	PGM3
SZ_Risk	PGC2_permissive	112611	RWDD2A
SZ_Risk	PGC2_permissive	399947	C11orf87
SZ_Risk	PGC2_permissive	225689	MAPK15
SZ_Risk	PGC2_permissive	286077	FAM83H
SZ_Risk	PGC2_permissive	100616318	MIR4664
SZ_Risk	PGC2_permissive	23513	SCRIB
SZ_Risk	PGC2_permissive	23081	KDM4C
SZ_Risk	PGC2_permissive	2185	PTK2B
SZ_Risk	PGC2_permissive	1135	CHRNA2
SZ_Risk	PGC2_permissive	4985	OPRD1
SZ_Risk	PGC2_permissive	5592	PRKG1
SZ_Risk	PGC2_permissive	6866	TAC3
SZ_Risk	PGC2_permissive	4640	MYO1A
SZ_Risk	PGC2_permissive	23306	TMEM194A
SZ_Risk	PGC2_permissive	4665	NAB2
SZ_Risk	PGC2_permissive	6778	STAT6
SZ_Risk	PGC2_permissive	51070	NOSIP
SZ_Risk	PGC2_permissive	5639	PRRG2
SZ_Risk	PGC2_permissive	57479	PRR12
SZ_Risk	PGC2_permissive	6237	RRAS
SZ_Risk	PGC2_permissive	58506	SCAF1
SZ_Risk	PGC2_permissive	7088	TLE1
SZ_Risk	PGC2_permissive	5520	PPP2R2A
SZ_Risk	PGC2_permissive	665	BNIP3L
SZ_Risk	PGC2_permissive	6770	STAR
SZ_Risk	PGC2_permissive	27257	LSM1
SZ_Risk	PGC2_permissive	9530	BAG4
SZ_Risk	PGC2_permissive	23259	DDHD2
SZ_Risk	PGC2_permissive	84513	PPAPDC1B
SZ_Risk	PGC2_permissive	54904	WHSC1L1
SZ_Risk	PGC2_permissive	137994	LETM2
SZ_Risk	PGC2_permissive	2260	FGFR1
SZ_Risk	PGC2_permissive	440594	FLJ31662
SZ_Risk	PGC2_permissive	11278	KLF12
SZ_Risk	PGC2_permissive	441389	FLJ35282
SZ_Risk	PGC2_permissive	27303	RBMS3
SZ_Risk	PGC2_permissive	23362	PSD3
SZ_Risk	PGC2_permissive	26084	ARHGEF26
SZ_Risk	PGC2_permissive	54715	RBFOX1
SZ_Risk	PGC2_permissive	100529144	CORO7-PAM16
SZ_Risk	PGC2_permissive	79585	CORO7
SZ_Risk	PGC2_permissive	114990	VASN
SZ_Risk	PGC2_permissive	9093	DNAJA3
SZ_Risk	PGC2_permissive	57407	NMRAL1
SZ_Risk	PGC2_permissive	3163	HMOX2
SZ_Risk	PGC2_permissive	342346	C16orf96
SZ_Risk	PGC2_permissive	4482	MSRA

SZ_Risk	PGC2_permissive	5142	PDE4B
SZ_Risk	PGC2_permissive	6457	SH3GL3
SZ_Risk	PGC2_permissive	53335	BCL11A
SZ_Risk	PGC2_permissive	27095	TRAPPC3
SZ_Risk	PGC2_permissive	55700	MAP7D1
SZ_Risk	PGC2_permissive	23774	BRD1
SZ_Risk	PGC2_permissive	90834	LOC90834
SZ_Risk	PGC2_permissive	9889	ZBED4
SZ_Risk	PGC2_permissive	79087	ALG12
SZ_Risk	PGC2_permissive	79174	CRELD2
SZ_Risk	PGC2_permissive	22871	NLGN1
SZ_Risk	PGC2_permissive	4916	NTRK3
SZ_Risk	PGC2_permissive	100874179	LINC00457
SZ_Risk	PGC2_permissive	9472	AKAP6
SZ_Risk	PGC2_permissive	8715	NOL4
SZ_Risk	PGC2_permissive	51678	MPP6
SZ_Risk	PGC2_permissive	1687	DFNA5
SZ_Risk	PGC2_permissive	26031	OSBPL3
SZ_Risk	PGC2_permissive	929	CD14
SZ_Risk	PGC2_permissive	55374	TMCO6
SZ_Risk	PGC2_permissive	4695	NDUFA2
SZ_Risk	PGC2_permissive	3550	IK
SZ_Risk	PGC2_permissive	100500820	MIR3655
SZ_Risk	PGC2_permissive	54853	WDR55
SZ_Risk	PGC2_permissive	373863	DND1
SZ_Risk	PGC2_permissive	3035	HARS
SZ_Risk	PGC2_permissive	23438	HARS2
SZ_Risk	PGC2_permissive	153527	ZMAT2
SZ_Risk	PGC2_permissive	56664	VTRNA1-1
SZ_Risk	PGC2_permissive	56663	VTRNA1-2
SZ_Risk	PGC2_permissive	56662	VTRNA1-3
SZ_Risk	PGC2_permissive	56147	PCDHA1
SZ_Risk	PGC2_permissive	56146	PCDHA2
SZ_Risk	PGC2_permissive	56145	PCDHA3
SZ_Risk	PGC2_permissive	56144	PCDHA4
SZ_Risk	PGC2_permissive	56143	PCDHA5
SZ_Risk	PGC2_permissive	56142	PCDHA6
SZ_Risk	PGC2_permissive	56141	PCDHA7
SZ_Risk	PGC2_permissive	56140	PCDHA8
SZ_Risk	PGC2_permissive	9752	PCDHA9
SZ_Risk	PGC2_permissive	56139	PCDHA10
SZ_Risk	PGC2_permissive	100113403	LINC00577
SZ_Risk	PGC2_permissive	389421	LIN28B
SZ_Risk	PGC2_permissive	133418	EMB
SZ_Risk	PGC2_permissive	29896	TRA2A
SZ_Risk	PGC2_permissive	1197	CLK2P
SZ_Risk	PGC2_permissive	90693	CCDC126
SZ_Risk	PGC2_permissive	25896	INTS7
SZ_Risk	PGC2_permissive	353189	SLCO4C1
SZ_Risk	PGC2_permissive	133482	SLCO6A1
SZ_Risk	PGC2_permissive	26056	RAB11FIP5
SZ_Risk	PGC2_permissive	196385	DNAH10
SZ_Risk	PGC2_permissive	80212	CCDC92
SZ_Risk	PGC2_permissive	144348	ZNF664
SZ_Risk	PGC2_permissive	100533183	ZNF664-FAM101A
SZ_Risk	PGC2_permissive	1081	CGA
SZ_Risk	PGC2_permissive	23036	ZNF292
SZ_Risk	PGC2_permissive	375519	GJB7

SZ_Risk	PGC2_permissive	206412	C6orf163
SZ_Risk	PGC2_permissive	63914	C6orf164
SZ_Risk	PGC2_permissive	154313	C6orf165
SZ_Risk	PGC2_permissive	10559	SLC35A1
SZ_Risk	PGC2_permissive	6252	RTN1
SZ_Risk	PGC2_permissive	84059	GPR98
SZ_Risk	PGC2_permissive	26010	SPATS2L
SZ_Risk	PGC2_permissive	79886	CAAP1
SZ_Risk	PGC2_permissive	9373	PLAA
SZ_Risk	PGC2_permissive	140803	TRPM6
SZ_Risk	PGC2_permissive	7086	TKT
SZ_Risk	PGC2_permissive	55802	DCP1A
SZ_Risk	PGC2_permissive	776	CACNA1D
SZ_Risk	PGC2_permissive	3990	LIPC
SZ_Risk	PGC2_permissive	83698	CALN1
SZ_Risk	PGC2_permissive	9568	GABBR2
SZ_Risk	PGC2_permissive	80309	SPHKAP
SZ_Risk	PGC2_permissive	100128239	LOC100128239
SZ_Risk	PGC2_permissive	5243	ABCB1
SZ_Risk	PGC2_permissive	154661	RUNDC3B
SZ_Risk	PGC2_permissive	55972	SLC25A40
SZ_Risk	PGC2_permissive	10926	DBF4
SZ_Risk	PGC2_permissive	53616	ADAM22
SZ_Risk	PGC2_permissive	1385	CREB1
SZ_Risk	PGC2_permissive	151194	METTL21A
SZ_Risk	PGC2_permissive	100874128	LINC00333
SZ_Risk	PGC2_permissive	114818	KLHL29
SZ_Risk	PGC2_permissive	100616119	MIR4697
SZ_Risk	PGC2_permissive	6671	SP4
SZ_Risk	PGC2_permissive	57186	RALGAPA2
SZ_Risk	PGC2_permissive	10082	GPC6
SZ_Risk	PGC2_permissive	100873973	GPC6-AS2
SZ_Risk	PGC2_permissive	29881	NPC1L1
SZ_Risk	PGC2_permissive	54606	DDX56
SZ_Risk	PGC2_permissive	54970	TTC12
SZ_Risk	PGC2_permissive	255239	ANKK1
SZ_Risk	PGC2_permissive	10454	TAB1
SZ_Risk	PGC2_permissive	100506472	LOC100506472
SZ_Risk	PGC2_permissive	4248	MGAT3
SZ_Risk	PGC2_permissive	2917	GRM7
SZ_Risk	PGC2_permissive	10750	GRAP
SZ_Risk	PGC2_permissive	400581	GRAPL
SZ_Risk	PGC2_permissive	22905	EPN2
SZ_Risk	PGC2_permissive	100874309	EPN2-IT1
SZ_Risk	PGC2_permissive	100874018	EPN2-AS1
SZ_Risk	PGC2_permissive	27077	B9D1
SZ_Risk	PGC2_permissive	100302256	MIR1180
SZ_Risk	PGC2_permissive	5598	MAPK7
SZ_Risk	PGC2_permissive	4239	MFAP4
SZ_Risk	PGC2_permissive	7732	RNF112
SZ_Risk	PGC2_permissive	2202	EFEMP1
SZ_Risk	PGC2_permissive	406999	MIR217
SZ_Risk	PGC2_permissive	406998	MIR216A
SZ_Risk	PGC2_permissive	3624	INHBA
SZ_Risk	PGC2_permissive	285954	INHBA-AS1
SZ_Risk	PGC2_permissive	4978	OPCML
SZ_Risk	PGC2_permissive	285370	LINC00606
SZ_Risk	PGC2_permissive	6900	CNTN2

SZ_Risk	PGC2_permissive	388730	TMEM81
SZ_Risk	PGC2_permissive	5929	RBBP5
SZ_Risk	PGC2_permissive	25778	DSTYK
SZ_Risk	PGC2_permissive	9911	TMCC2
SZ_Risk	PGC2_permissive	2904	GRIN2B
SZ_Risk	PGC2_permissive	79840	NHEJ1
SZ_Risk	PGC2_permissive	151295	SLC23A3
SZ_Risk	PGC2_permissive	27013	CNPPD1
SZ_Risk	PGC2_permissive	79137	FAM134A
SZ_Risk	PGC2_permissive	130617	ZFAND2B
SZ_Risk	PGC2_permissive	10058	ABCB6
SZ_Risk	PGC2_permissive	84630	TTBK1
SZ_Risk	PGC2_permissive	10864	SLC22A7
SZ_Risk	PGC2_permissive	401262	CRIP3
SZ_Risk	PGC2_permissive	24149	ZNF318
SZ_Risk	PGC2_permissive	89845	ABCC10
SZ_Risk	PGC2_permissive	65989	DLK2
SZ_Risk	PGC2_permissive	11344	TWF2
SZ_Risk	PGC2_permissive	132160	PPM1M
SZ_Risk	PGC2_permissive	80335	WDR82
SZ_Risk	PGC2_permissive	406890	MIRLET7G
SZ_Risk	PGC2_permissive	132158	GLYCTK
SZ_Risk	PGC2_permissive	406925	MIR135A1
SZ_Risk	PGC2_permissive	25981	DNAH1
SZ_Risk	PGC2_permissive	8314	BAP1
SZ_Risk	PGC2_permissive	51533	PHF7
SZ_Risk	PGC2_permissive	169792	GLIS3
SZ_Risk	PGC2_permissive	84850	GLIS3-AS1
SZ_Risk	PGC2_permissive	5629	PROX1
SZ_Risk	PGC2_permissive	11216	AKAP10
SZ_Risk	PGC2_permissive	92521	SPECC1
SZ_Risk	PGC2_permissive	284194	LGALS9B
SZ_Risk	PGC2_permissive	26251	KCNG2
SZ_Risk	PGC2_permissive	80148	PQLC1
SZ_Risk	PGC2_permissive	2788	GNG7
SZ_Risk	PGC2_permissive	148252	DIRAS1
SZ_Risk	PGC2_permissive	29985	SLC39A3
SZ_Risk	PGC2_permissive	83700	JAM3
SZ_Risk	PGC2_permissive	57521	RPTOR
SZ_Risk	PGC2_permissive	27087	B3GAT1
SZ_Risk	PGC2_permissive	283177	LOC283177
SZ_Risk	PGC2_permissive	6660	SOX5
SZ_Risk	PGC2_permissive	254531	LPCAT4
SZ_Risk	PGC2_permissive	23015	GOLGA8A
SZ_Risk	PGC2_permissive	79166	LILRP2
SZ_Risk	PGC2_permissive	115653	KIR3DL3
SZ_Risk	PGC2_permissive	221078	NSUN6
SZ_Risk	PGC2_permissive	4842	NOS1
SZ_Risk	PGC2_permissive	64131	XYLT1
SZ_Risk	PGC2_permissive	5526	PPP2R5B
SZ_Risk	PGC2_permissive	170589	GPHA2
SZ_Risk	PGC2_permissive	283129	C11orf85
SZ_Risk	PGC2_permissive	56899	ANKS1B
SZ_Risk	PGC2_permissive	9839	ZEB2
SZ_Risk	PGC2_permissive	8546	AP3B1
SZ_Risk	PGC2_permissive	144097	C11orf84
SZ_Risk	PGC2_permissive	2011	MARK2
SZ_Risk	PGC2_permissive	6315	ATXN8OS

SZ_Risk	PGC2_permissive	80114	BICC1
SZ_Risk	PGC2_permissive	5980	REV3L
SZ_Risk	PGC2_permissive	643749	TRAF3IP2-AS1
SZ_Risk	PGC2_permissive	10758	TRAF3IP2
SZ_Risk	PGC2_permissive	729993	SHISA9
SZ_Risk	PGC2_permissive	51523	CXXC5
SZ_Risk	PGC2_permissive	23237	ARC
SZ_Risk	PGC2_permissive	8629	JRK
SZ_Risk	PGC2_permissive	8000	PSCA
SZ_Risk	PGC2_permissive	165721	DNAJB8
SZ_Risk	PGC2_permissive	285224	DNAJB8-AS1
SZ_Risk	PGC2_permissive	2624	GATA2
SZ_Risk	PGC2_permissive	1917	EEF1A2
SZ_Risk	PGC2_permissive	3344	FOXN2
SZ_Risk	PGC2_permissive	129285	PPP1R21
SZ_Risk	PGC2_permissive	1800	DPEP1
SZ_Risk	PGC2_permissive	5119	CHMP1A
SZ_Risk	PGC2_permissive	440279	UNC13C
SZ_Risk	PGC2_permissive	285084	LOC285084
SZ_Risk	PGC2_permissive	100131390	SP9
SZ_Risk	PGC2_permissive	9541	CIR1
SZ_Risk	PGC2_permissive	79634	SCRN3
SZ_Risk	PGC2_permissive	9863	MAGI2
SZ_Risk	PGC2_permissive	650794	MIPEPP3
SZ_Risk	PGC2_permissive	253832	ZDHHC20
SZ_Risk	PGC2_permissive	23264	ZC3H7B
SZ_Risk	PGC2_permissive	7008	TEF
SZ_Risk	PGC2_permissive	10766	TOB2
SZ_Risk	PGC2_permissive	84844	PHF5A
SZ_Risk	PGC2_permissive	50	ACO2
SZ_Risk	PGC2_permissive	171568	POLR3H
SZ_Risk	PGC2_permissive	27254	CSDC2
SZ_Risk	PGC2_permissive	5372	PMM1
SZ_Risk	PGC2_permissive	57511	COG6
SZ_Risk	PGC2_permissive	2590	GALNT2
SZ_Risk	PGC2_permissive	254102	EHBP1L1
SZ_Risk	PGC2_permissive	10089	KCNK7
SZ_Risk	PGC2_permissive	4296	MAP3K11
SZ_Risk	PGC2_permissive	399909	PCNXL3
SZ_Risk	PGC2_permissive	100616292	MIR4690
SZ_Risk	PGC2_permissive	6494	SIPA1
SZ_Risk	PGC2_permissive	100616284	MIR4489
SZ_Risk	PGC2_permissive	5970	RELA
SZ_Risk	PGC2_permissive	10524	KAT5
SZ_Risk	PGC2_permissive	84153	RNASEH2C
SZ_Risk	PGC2_permissive	8997	KALRN
SZ_Risk	PGC2_permissive	65217	PCDH15
SZ_Risk	PGC2_permissive	121256	TMEM132D
SZ_Risk	PGC2_permissive	1781	DYNC1I2
SZ_Risk	PGC2_permissive	8604	SLC25A12
SZ_Risk	PGC2_permissive	8520	HAT1
SZ_Risk	PGC2_permissive	254042	METAP1D
SZ_Risk	PGC2_permissive	100128977	MAPT-AS1
SZ_Risk	PGC2_permissive	162540	SPPL2C
SZ_Risk	PGC2_permissive	4137	MAPT
SZ_Risk	PGC2_permissive	100130148	MAPT-IT1
SZ_Risk	PGC2_permissive	54662	TBC1D13
SZ_Risk	PGC2_permissive	2021	ENDOG

SZ_Risk	PGC2_permissive	51490	C9orf114
SZ_Risk	PGC2_permissive	883	CCBL1
SZ_Risk	PGC2_permissive	56262	LRRC8A
SZ_Risk	PGC2_permissive	22821	RASA3
SZ_Risk	PGC2_permissive	80059	LRRTM4
SZ_Risk	PGC2_permissive	7068	THRB
SZ_Risk	PGC2_permissive	151126	ZNF385B
SZ_Risk	PGC2_permissive	654502	IQCJ
SZ_Risk	PGC2_permissive	100505385	IQCJ-SCHIP1
SZ_Risk	PGC2_permissive	29970	SCHIP1
SZ_Risk	PGC2_permissive	388336	SHISA6
SZ_Risk	PGC2_permissive	150381	LOC150381
SZ_Risk	PGC2_permissive	400931	MIRLET7BHG
SZ_Risk	PGC2_permissive	100500828	MIR3619
SZ_Risk	PGC2_permissive	1316	KLF6
SZ_Risk	PGC2_permissive	54674	LRRN3
SZ_Risk	PGC2_permissive	5579	PRKCB
SZ_Risk	PGC2_permissive	152006	RNF38
SZ_Risk	PGC2_permissive	1643	DDB2
SZ_Risk	PGC2_permissive	53	ACP2
SZ_Risk	PGC2_permissive	10062	NR1H3
SZ_Risk	PGC2_permissive	8567	MADD
SZ_Risk	PGC2_permissive	285671	RNF180
SZ_Risk	PGC2_permissive	100507421	TMEM178B
SZ_Risk	PGC2_permissive	51571	FAM49B
SZ_Risk	PGC2_permissive	100847051	MIR5194
SZ_Risk	PGC2_permissive	50807	ASAP1
SZ_Risk	PGC2_permissive	29065	ASAP1-IT1
SZ_Risk	PGC2_permissive	794	CALB2
SZ_Risk	PGC2_permissive	8945	BTRC
SZ_Risk	PGC2_permissive	27343	POLL
SZ_Risk	PGC2_permissive	25911	DPCD
SZ_Risk	PGC2_permissive	100422900	MIR3158-1
SZ_Risk	PGC2_permissive	100423033	MIR3158-2
SZ_Risk	PGC2_permissive	6468	FBXW4
SZ_Risk	PGC2_permissive	10579	TACC2
SZ_Risk	PGC2_permissive	11281	POU6F2
SZ_Risk	PGC2_permissive	57606	SLAIN2
SZ_Risk	PGC2_permissive	201780	SLC10A4
SZ_Risk	PGC2_permissive	326340	ZAR1
SZ_Risk	PGC2_permissive	285527	FRYL
SZ_Risk	PGC2_permissive	57520	HECW2
SZ_Risk	PGC2_permissive	107	ADCY1
SZ_Risk	PGC2_permissive	56648	EIF5A2
SZ_Risk	PGC2_permissive	54808	DYM
SZ_Risk	PGC2_permissive	100616420	MIR4744
SZ_Risk	PGC2_permissive	100129027	LOC100129027
SZ_Risk	PGC2_permissive	744	MPPED2
SZ_Risk	PGC2_permissive	10613	ERLIN1
SZ_Risk	PGC2_permissive	1147	CHUK
SZ_Risk	PGC2_permissive	55280	CWF19L1
SZ_Risk	PGC2_permissive	677800	SNORA12
SZ_Risk	PGC2_permissive	282991	BLOC1S2
SZ_Risk	PGC2_permissive	9033	PKD2L1
SZ_Risk	PGC2_permissive	22986	SORCS3
SZ_Risk	PGC2_permissive	4784	NFIX
SZ_Risk	PGC2_permissive	65009	NDRG4
SZ_Risk	PGC2_permissive	79918	SETD6

SZ_Risk	PGC2_permissive	677827	SNORA46
SZ_Risk	PGC2_permissive	677830	SNORA50
SZ_Risk	PGC2_permissive	391365	SULT6B1
SZ_Risk	PGC2_permissive	100505876	LOC100505876
SZ_Risk	PGC2_permissive	10153	CEBPZ
SZ_Risk	PGC2_permissive	23683	PRKD3
SZ_Risk	PGC2_permissive	25797	QPCT
SZ_Risk	PGC2_permissive	6345	SRL
SZ_Risk	PGC2_permissive	149465	WDR65
SZ_Risk	PGC2_permissive	128218	TMEM125
SZ_Risk	PGC2_permissive	57591	MKL1
SZ_Risk	PGC2_permissive	2847	MCHR1
SZ_Risk	PGC2_permissive	1983	EIF5
SZ_Risk	PGC2_permissive	677811	SNORA28
SZ_Risk	PGC2_permissive	491	ATP2B2
SZ_Risk	PGC2_permissive	100126334	MIR885
SZ_Risk	PGC2_permissive	55765	C1orf106
SZ_Risk	PGC2_permissive	23046	KIF21B
SZ_Risk	PGC2_permissive	779	CACNA1S
SZ_Risk	PGC2_permissive	64839	FBXL17
SZ_Risk	PGC2_permissive	9912	ARHGAP44
SZ_Risk	PGC2_permissive	60528	ELAC2
SZ_Risk	PGC2_permissive	257194	NEGR1
SZ_Risk	PGC2_permissive	64400	AKTIP
SZ_Risk	PGC2_permissive	23322	RPGRIP1L
SZ_Risk	PGC2_permissive	79068	FTO
SZ_Risk	PGC2_permissive	51460	SFMBT1
SZ_Risk	PGC2_permissive	407051	MIR9-3
SZ_Risk	PGC2_permissive	375057	C1orf95
SZ_Risk	PGC2_permissive	100526767	RNF103-CHMP3
SZ_Risk	PGC2_permissive	64795	RMND5A
SZ_Risk	PGC2_permissive	133022	TRAM1L1
SZ_Risk	PGC2_permissive	25902	MTHFD1L
SZ_Risk	PGC2_permissive	51043	ZBTB7B
SZ_Risk	PGC2_permissive	127579	DCST2
SZ_Risk	PGC2_permissive	149095	DCST1
SZ_Risk	PGC2_permissive	7844	RNF103
SZ_Risk	PGC2_permissive	925	CD8A
SZ_Risk	PGC2_permissive	162494	RHBDL3
SZ_Risk	PGC2_permissive	64149	C17orf75
SZ_Risk	PGC2_permissive	693217	MIR632
SZ_Risk	PGC2_permissive	7756	ZNF207
SZ_Risk	PGC2_permissive	5717	PSMD11
SZ_Risk	PGC2_permissive	8851	CDK5R1
SZ_Risk	PGC2_permissive	4642	MYO1D
SZ_Risk	PGC2_permissive	10100	TSPAN2
SZ_Risk	PGC2_permissive	64582	GPR135
SZ_Risk	PGC2_permissive	112849	L3HYPDH
SZ_Risk	PGC2_permissive	51528	JKAMP
SZ_Risk	PGC2_permissive	729665	CCDC175
SZ_Risk	PGC2_permissive	100847084	MIR548AP
SZ_Risk	PGC2_permissive	343450	KCNT2
SZ_Risk	PGC2_permissive	7150	TOP1
SZ_Risk	PGC2_permissive	5335	PLCG1
SZ_Risk	PGC2_permissive	23051	ZHX3
SZ_Risk	PGC2_permissive	64900	LPIN3
SZ_Risk	PGC2_permissive	9148	NEURL
SZ_Risk	PGC2_permissive	4481	MSR1

SZ_Risk	PGC2_permissive	79570	NKAIN1
SZ_Risk	PGC2_permissive	9410	SNRNP40
SZ_Risk	PGC2_permissive	51538	ZCCHC17
SZ_Risk	PGC2_permissive	2170	FABP3
SZ_Risk	PGC2_permissive	347735	SERINC2
SZ_Risk	PGC2_permissive	7531	YWHAE
SZ_Risk	PGC2_permissive	94134	ARHGAP12
SZ_Risk	PGC2_permissive	3799	KIF5B
SZ_Risk	PGC2_permissive	406917	MIR129-1
SZ_Risk	PGC2_permissive	3952	LEP
SZ_Risk	PGC2_permissive	266722	HS6ST3
SZ_Risk	PGC2_permissive	3191	HNRNPL
SZ_Risk	PGC2_permissive	126432	RINL
SZ_Risk	PGC2_permissive	9856	KIAA0319
SZ_Risk	PGC2_permissive	51567	TDP2
SZ_Risk	PGC2_permissive	55856	ACOT13
SZ_Risk	PGC2_permissive	81688	C6orf62
SZ_Risk	PGC2_permissive	55561	CDC42BPG
SZ_Risk	PGC2_permissive	10938	EHD1
SZ_Risk	PGC2_permissive	406967	MIR192
SZ_Risk	PGC2_permissive	406970	MIR194-2
SZ_Risk	PGC2_permissive	23130	ATG2A
SZ_Risk	PGC2_permissive	116071	BATF2
SZ_Risk	PGC2_permissive	402	ARL2
SZ_Risk	PGC2_permissive	100528018	ARL2-SNX15
SZ_Risk	PGC2_permissive	29907	SNX15
SZ_Risk	PGC2_permissive	64062	RBM26
SZ_Risk	PGC2_permissive	100505538	RBM26-AS1
SZ_Risk	PGC2_permissive	100874208	NDFIP2-AS1
SZ_Risk	PGC2_permissive	54602	NDFIP2
SZ_Risk	PGC2_permissive	6249	CLIP1
SZ_Risk	PGC2_permissive	100507066	LOC100507066
SZ_Risk	PGC2_permissive	55596	ZCCHC8
SZ_Risk	PGC2_permissive	5287	PIK3C2B
SZ_Risk	PGC2_permissive	4194	MDM4
SZ_Risk	PGC2_permissive	10446	LRRN2
SZ_Risk	PGC2_permissive	1838	DTNB
SZ_Risk	PGC2_permissive	474	ATOH1
SZ_Risk	PGC2_permissive	283446	MYO1H
SZ_Risk	PGC2_permissive	83892	KCTD10
SZ_Risk	PGC2_permissive	89910	UBE3B
SZ_Risk	PGC2_permissive	326625	MMAB
SZ_Risk	PGC2_permissive	4598	MVK
SZ_Risk	PGC2_permissive	196513	DCP1B
SZ_Risk	PGC2_permissive	158293	FAM120AOS
SZ_Risk	PGC2_permissive	23196	FAM120A
SZ_Risk	PGC2_permissive	5253	PHF2
SZ_Risk	PGC2_permissive	421	ARVCF
SZ_Risk	PGC2_permissive	406961	MIR185
SZ_Risk	PGC2_permissive	54487	DGCR8
SZ_Risk	PGC2_permissive	100500860	MIR3618
SZ_Risk	PGC2_permissive	100302197	MIR1306
SZ_Risk	PGC2_permissive	27037	TRMT2A
SZ_Risk	PGC2_permissive	5902	RANBP1
SZ_Risk	PGC2_permissive	29801	ZDHHC8
SZ_Risk	PGC2_permissive	388849	LOC388849
SZ_Risk	PGC2_permissive	729987	LOC729987
SZ_Risk	PGC2_permissive	51065	RPS27L

SZ_Risk	PGC2_permissive	51762	RAB8B
SZ_Risk	PGC2_permissive	100506710	LOC100506710
SZ_Risk	PGC2_permissive	84186	ZCCHC7
SZ_Risk	PGC2_permissive	344022	NOTO
SZ_Risk	PGC2_permissive	10322	SMYD5
SZ_Risk	PGC2_permissive	84279	PRADC1
SZ_Risk	PGC2_permissive	10574	CCT7
SZ_Risk	PGC2_permissive	9764	KIAA0513
SZ_Risk	PGC2_permissive	339145	FAM92B
SZ_Risk	PGC2_permissive	1630	DCC
SZ_Risk	PGC2_permissive	57172	CAMK1G
SZ_Risk	PGC2_permissive	6602	SMARCD1
SZ_Risk	PGC2_permissive	2819	GPD1
SZ_Risk	PGC2_permissive	84987	COX14
SZ_Risk	PGC2_permissive	91012	CERS5
SZ_Risk	PGC2_permissive	51474	LIMA1
SZ_Risk	PGC2_permissive	100302220	MIR1293
SZ_Risk	PGC2_permissive	121006	FAM186A
SZ_Risk	PGC2_permissive	113251	LARP4
SZ_Risk	PGC2_permissive	57609	DIP2B
SZ_Risk	PGC2_permissive	6016	RIT1
SZ_Risk	PGC2_permissive	22889	KIAA0907
SZ_Risk	PGC2_permissive	677823	SNORA42
SZ_Risk	PGC2_permissive	677771	SCARNA4
SZ_Risk	PGC2_permissive	4126	MANBA
SZ_Risk	PGC2_permissive	7323	UBE2D3
SZ_Risk	PGC2_permissive	493856	CISD2
SZ_Risk	PGC2_permissive	150159	SLC9B1
SZ_Risk	PGC2_permissive	133308	SLC9B2
SZ_Risk	PGC2_permissive	222255	ATXN7L1
SZ_Risk	PGC2_permissive	26137	ZBTB20
SZ_Risk	PGC2_permissive	116154	PHACTR3
SZ_Risk	PGC2_permissive	387885	CCDC42B
SZ_Risk	PGC2_permissive	79039	DDX54
SZ_Risk	PGC2_permissive	84934	C12orf52
SZ_Risk	PGC2_permissive	115811	IQCD
SZ_Risk	PGC2_permissive	53373	TPCN1
SZ_Risk	PGC2_permissive	9071	CLDN10
SZ_Risk	PGC2_permissive	100874194	CLDN10-AS1
SZ_Risk	PGC2_permissive	54629	FAM63B
SZ_Risk	PGC2_permissive	79811	SLTM
SZ_Risk	PGC2_permissive	85439	STON2
SZ_Risk	PGC2_permissive	403341	ZBTB34
SZ_Risk	PGC2_permissive	9649	RALGPS1
SZ_Risk	PGC2_permissive	23452	ANGPTL2
SZ_Risk	PGC2_permissive	22891	ZNF365
SZ_Risk	PGC2_permissive	64843	ISL2
SZ_Risk	PGC2_permissive	49855	SCAPER
SZ_Risk	PGC2_permissive	63978	PRDM14
SZ_Risk	PGC2_permissive	80727	TTYH3
SZ_Risk	PGC2_permissive	155185	AMZ1
SZ_Risk	PGC2_permissive	11252	PACSIN2
SZ_Risk	PGC2_permissive	25809	TTLL1
SZ_Risk	PGC2_permissive	442319	ZNF727
SZ_Risk	PGC2_permissive	463	ZFHX3
SZ_Risk	PGC2_permissive	148479	PHF13
SZ_Risk	PGC2_permissive	90326	THAP3
SZ_Risk	PGC2_permissive	55735	DNAJC11

SZ_Risk	PGC2_permissive	5101	PCDH9
SZ_Risk	PGC2_permissive	100874086	PCDH9-AS3
SZ_Risk	scz-denovo-lof	122773	KLHDC1
SZ_Risk	scz-denovo-lof	1770	DNAH9
SZ_Risk	scz-denovo-lof	3320	HSP90AA1
SZ_Risk	scz-denovo-lof	23648	SSBP3
SZ_Risk	scz-denovo-lof	9213	XPR1
SZ_Risk	scz-denovo-lof	23331	TTC28
SZ_Risk	scz-denovo-lof	84775	ZNF607
SZ_Risk	scz-denovo-lof	90313	TP53I13
SZ_Risk	scz-denovo-lof	9588	PRDX6
SZ_Risk	scz-denovo-lof	6326	SCN2A
SZ_Risk	scz-denovo-lof	84166	NLRC5
SZ_Risk	scz-denovo-lof	92667	MGME1
SZ_Risk	scz-denovo-lof	9874	TLK1
SZ_Risk	scz-denovo-lof	259173	ALS2CL
SZ_Risk	scz-denovo-lof	4035	LRP1
SZ_Risk	scz-denovo-lof	23259	DDHD2
SZ_Risk	scz-denovo-lof	1180	CLCN1
SZ_Risk	scz-denovo-lof	405753	DUOXA2
SZ_Risk	scz-denovo-lof	6935	ZEB1
SZ_Risk	scz-denovo-lof	51533	PHF7
SZ_Risk	scz-denovo-lof	90952	ESAM
SZ_Risk	scz-denovo-lof	55269	PSPC1
SZ_Risk	scz-denovo-lof	91746	YTHDC1
SZ_Risk	scz-denovo-lof	4674	NAP1L2
SZ_Risk	scz-denovo-lof	10002	NR2E3
SZ_Risk	scz-denovo-lof	339451	KLHL17
SZ_Risk	scz-denovo-lof	55628	ZNF407
SZ_Risk	scz-denovo-lof	3008	HIST1H1E
SZ_Risk	scz-denovo-lof	1969	EPHA2
SZ_Risk	scz-denovo-lof	4343	MOV10
SZ_Risk	scz-denovo-lof	56244	BTNL2
SZ_Risk	scz-denovo-lof	25921	ZDHHC5
SZ_Risk	scz-denovo-lof	84705	GTPBP3
SZ_Risk	scz-denovo-lof	25836	NIPBL
SZ_Risk	scz-denovo-lof	6884	TAF13
SZ_Risk	scz-denovo-lof	3908	LAMA2
SZ_Risk	scz-denovo-lof	7862	BRPF1
SZ_Risk	scz-denovo-lof	23334	SZT2
SZ_Risk	scz-denovo-lof	586	BCAT1
SZ_Risk	scz-denovo-lof	3312	HSPA8
SZ_Risk	scz-denovo-lof	5067	CNTN3
SZ_Risk	scz-denovo-lof	147657	ZNF480
SZ_Risk	scz-denovo-lof	23126	POGZ
SZ_Risk	scz-denovo-lof	9140	ATG12
SZ_Risk	scz-denovo-lof	221955	DAGLB
SZ_Risk	scz-denovo-lof	8831	SYNGAP1
SZ_Risk	scz-denovo-lof	59269	HIVEP3
SZ_Risk	scz-denovo-lof	51517	NCKIPSD
SZ_Risk	scz-denovo-lof	10659	CELF2
SZ_Risk	scz-denovo-lof	1740	DLG2
SZ_Risk	scz-denovo-lof	1806	DPYD
SZ_Risk	scz-denovo-lof	9816	URB2
SZ_Risk	scz-denovo-lof	84629	TNRC18
SZ_Risk	scz-denovo-lof	4282	MIF
SZ_Risk	scz-denovo-lof	4288	MKI67
SZ_Risk	scz-denovo-lof	8242	KDM5C

SZ_Risk	scz-denovo-lof	57185	NIPAL3
SZ_Risk	scz-denovo-lof	11260	XPOT
SZ_Risk	scz-denovo-lof	2532	DARC
SZ_Risk	scz-denovo-lof	5916	RARG
SZ_Risk	scz-denovo-lof	5287	PIK3C2B
SZ_Risk	scz-denovo-lof	9821	RB1CC1
SZ_Risk	scz-denovo-lof	57787	MARK4
SZ_Risk	scz-denovo-lof	199953	TMEM201
SZ_Risk	scz-denovo-lof	23376	UFL1
SZ_Risk	scz-denovo-lof	50624	CUZD1
SZ_Risk	scz-denovo-lof	6453	ITSN1
SZ_Risk	scz-denovo-lof	3836	KPNA1
SZ_Risk	scz-denovo-lof	10771	ZMYND11
SZ_Risk	scz-denovo-lof	339829	CCDC39
SZ_Risk	scz-denovo-lof	7840	ALMS1
SZ_Risk	scz-denovo-lof	23266	LPHN2
SZ_Risk	scz-denovo-lof	344838	PAQR9
SZ_Risk	scz-denovo-lof	1833	EPYC
SZ_Risk	scz-denovo-lof	56134	PCDHAC2
SZ_Risk	scz-denovo-lof	50944	SHANK1
SZ_Risk	scz-denovo-lof	55620	STAP2
SZ_Risk	scz-denovo-lof	7200	TRH
SZ_Risk	scz-denovo-lof	83593	RASSF5
SZ_Risk	scz-denovo-lof	25962	KIAA1429
SZ_Risk	scz-denovo-lof	54832	VPS13C
SZ_Risk	scz-denovo-lof	6601	SMARCC2
SZ_Risk	scz-denovo-lof	4703	NEB
SZ_Risk	scz-denovo-lof	7916	PRRC2A
SZ_Risk	scz-denovo-lof	53371	NUP54
SZ_Risk	scz-denovo-lof	57644	MYH7B
SZ_Risk	scz-denovo-nonsyn	170960	ZNF721
SZ_Risk	scz-denovo-nonsyn	7201	TRHR
SZ_Risk	scz-denovo-nonsyn	8658	TNKS
SZ_Risk	scz-denovo-nonsyn	23566	LPAR3
SZ_Risk	scz-denovo-nonsyn	83879	CDCA7
SZ_Risk	scz-denovo-nonsyn	220965	FAM13C
SZ_Risk	scz-denovo-nonsyn	166378	SPATA5
SZ_Risk	scz-denovo-nonsyn	122525	C14orf28
SZ_Risk	scz-denovo-nonsyn	25793	FBXO7
SZ_Risk	scz-denovo-nonsyn	90952	ESAM
SZ_Risk	scz-denovo-nonsyn	50831	TAS2R3
SZ_Risk	scz-denovo-nonsyn	3645	INSRR
SZ_Risk	scz-denovo-nonsyn	140453	MUC17
SZ_Risk	scz-denovo-nonsyn	929	CD14
SZ_Risk	scz-denovo-nonsyn	8969	HIST1H2AG
SZ_Risk	scz-denovo-nonsyn	7011	TEP1
SZ_Risk	scz-denovo-nonsyn	376940	ZC3H6
SZ_Risk	scz-denovo-nonsyn	80184	CEP290
SZ_Risk	scz-denovo-nonsyn	1775	DNASE1L2
SZ_Risk	scz-denovo-nonsyn	727897	MUC5B
SZ_Risk	scz-denovo-nonsyn	8503	PIK3R3
SZ_Risk	scz-denovo-nonsyn	259173	ALS2CL
SZ_Risk	scz-denovo-nonsyn	10880	ACTL7B
SZ_Risk	scz-denovo-nonsyn	23376	UFL1
SZ_Risk	scz-denovo-nonsyn	7275	TUB
SZ_Risk	scz-denovo-nonsyn	1756	DMD
SZ_Risk	scz-denovo-nonsyn	3356	HTR2A
SZ_Risk	scz-denovo-nonsyn	338657	CCDC84

SZ_Risk	scz-denovo-nonsyn	116369	SLC26A8
SZ_Risk	scz-denovo-nonsyn	57003	CCDC47
SZ_Risk	scz-denovo-nonsyn	10771	ZMYND11
SZ_Risk	scz-denovo-nonsyn	51517	NCKIPSD
SZ_Risk	scz-denovo-nonsyn	5991	RFX3
SZ_Risk	scz-denovo-nonsyn	22827	PUF60
SZ_Risk	scz-denovo-nonsyn	85315	PAQR8
SZ_Risk	scz-denovo-nonsyn	344838	PAQR9
SZ_Risk	scz-denovo-nonsyn	200845	KCTD6
SZ_Risk	scz-denovo-nonsyn	5140	PDE3B
SZ_Risk	scz-denovo-nonsyn	8294	HIST1H4I
SZ_Risk	scz-denovo-nonsyn	3938	LCT
SZ_Risk	scz-denovo-nonsyn	389384	C6orf222
SZ_Risk	scz-denovo-nonsyn	779	CACNA1S
SZ_Risk	scz-denovo-nonsyn	205717	KIAA2018
SZ_Risk	scz-denovo-nonsyn	7534	YWHAZ
SZ_Risk	scz-denovo-nonsyn	84441	MAML2
SZ_Risk	scz-denovo-nonsyn	3632	INPP5A
SZ_Risk	scz-denovo-nonsyn	84674	CARD6
SZ_Risk	scz-denovo-nonsyn	23339	VPS39
SZ_Risk	scz-denovo-nonsyn	8295	TRRAP
SZ_Risk	scz-denovo-nonsyn	284756	C20orf197
SZ_Risk	scz-denovo-nonsyn	284217	LAMA1
SZ_Risk	scz-denovo-nonsyn	3312	HSPA8
SZ_Risk	scz-denovo-nonsyn	3908	LAMA2
SZ_Risk	scz-denovo-nonsyn	91949	COG7
SZ_Risk	scz-denovo-nonsyn	3910	LAMA4
SZ_Risk	scz-denovo-nonsyn	57475	PLEKHH1
SZ_Risk	scz-denovo-nonsyn	57492	ARID1B
SZ_Risk	scz-denovo-nonsyn	84678	KDM2B
SZ_Risk	scz-denovo-nonsyn	9368	SLC9A3R1
SZ_Risk	scz-denovo-nonsyn	5670	PSG2
SZ_Risk	scz-denovo-nonsyn	283848	CES4A
SZ_Risk	scz-denovo-nonsyn	26953	RANBP6
SZ_Risk	scz-denovo-nonsyn	7268	TTC4
SZ_Risk	scz-denovo-nonsyn	121457	IKBIP
SZ_Risk	scz-denovo-nonsyn	84447	SYVN1
SZ_Risk	scz-denovo-nonsyn	59269	HIVEP3
SZ_Risk	scz-denovo-nonsyn	56889	TM9SF3
SZ_Risk	scz-denovo-nonsyn	90627	STARD13
SZ_Risk	scz-denovo-nonsyn	3667	IRS1
SZ_Risk	scz-denovo-nonsyn	2015	EMR1
SZ_Risk	scz-denovo-nonsyn	84658	EMR3
SZ_Risk	scz-denovo-nonsyn	54801	HAUS6
SZ_Risk	scz-denovo-nonsyn	54209	TREM2
SZ_Risk	scz-denovo-nonsyn	93323	HAUS8
SZ_Risk	scz-denovo-nonsyn	63943	FKBPL
SZ_Risk	scz-denovo-nonsyn	4343	MOV10
SZ_Risk	scz-denovo-nonsyn	2694	GIF
SZ_Risk	scz-denovo-nonsyn	51332	SPTBN5
SZ_Risk	scz-denovo-nonsyn	4288	MKI67
SZ_Risk	scz-denovo-nonsyn	87	ACTN1
SZ_Risk	scz-denovo-nonsyn	57533	TBC1D14
SZ_Risk	scz-denovo-nonsyn	339501	PRSS38
SZ_Risk	scz-denovo-nonsyn	84166	NLRC5
SZ_Risk	scz-denovo-nonsyn	23274	CLEC16A
SZ_Risk	scz-denovo-nonsyn	7692	ZNF133
SZ_Risk	scz-denovo-nonsyn	219537	SMTNL1

SZ_Risk	scz-denovo-nonsyn	9805	SCRN1
SZ_Risk	scz-denovo-nonsyn	4926	NUMA1
SZ_Risk	scz-denovo-nonsyn	58525	WIZ
SZ_Risk	scz-denovo-nonsyn	50624	CUZD1
SZ_Risk	scz-denovo-nonsyn	23334	SZT2
SZ_Risk	scz-denovo-nonsyn	131177	FAM3D
SZ_Risk	scz-denovo-nonsyn	79036	KXD1
SZ_Risk	scz-denovo-nonsyn	5916	RARG
SZ_Risk	scz-denovo-nonsyn	8204	NRIP1
SZ_Risk	scz-denovo-nonsyn	8434	RECK
SZ_Risk	scz-denovo-nonsyn	25921	ZDHHC5
SZ_Risk	scz-denovo-nonsyn	29904	EEF2K
SZ_Risk	scz-denovo-nonsyn	214	ALCAM
SZ_Risk	scz-denovo-nonsyn	84647	PLA2G12B
SZ_Risk	scz-denovo-nonsyn	48	ACO1
SZ_Risk	scz-denovo-nonsyn	2033	EP300
SZ_Risk	scz-denovo-nonsyn	29115	SAP30BP
SZ_Risk	scz-denovo-nonsyn	54932	EXD3
SZ_Risk	scz-denovo-nonsyn	643414	LIPK
SZ_Risk	scz-denovo-nonsyn	4931	NVL
SZ_Risk	scz-denovo-nonsyn	83449	PMFBP1
SZ_Risk	scz-denovo-nonsyn	641372	ACOT6
SZ_Risk	scz-denovo-nonsyn	56479	KCNQ5
SZ_Risk	scz-denovo-nonsyn	84162	KIAA1109
SZ_Risk	scz-denovo-nonsyn	10142	AKAP9
SZ_Risk	scz-denovo-nonsyn	2185	PTK2B
SZ_Risk	scz-denovo-nonsyn	9343	EFTUD2
SZ_Risk	scz-denovo-nonsyn	1826	DSCAM
SZ_Risk	scz-denovo-nonsyn	55717	WDR11
SZ_Risk	scz-denovo-nonsyn	54659	UGT1A3
SZ_Risk	scz-denovo-nonsyn	8943	AP3D1
SZ_Risk	scz-denovo-nonsyn	9588	PRDX6
SZ_Risk	scz-denovo-nonsyn	7402	UTRN
SZ_Risk	scz-denovo-nonsyn	53371	NUP54
SZ_Risk	scz-denovo-nonsyn	726	CAPN5
SZ_Risk	scz-denovo-nonsyn	9829	DNAJC6
SZ_Risk	scz-denovo-nonsyn	10753	CAPN9
SZ_Risk	scz-denovo-nonsyn	25831	HECTD1
SZ_Risk	scz-denovo-nonsyn	23076	RRP1B
SZ_Risk	scz-denovo-nonsyn	6474	SHOX2
SZ_Risk	scz-denovo-nonsyn	8940	TOP3B
SZ_Risk	scz-denovo-nonsyn	9967	THRAP3
SZ_Risk	scz-denovo-nonsyn	57448	BIRC6
SZ_Risk	scz-denovo-nonsyn	85301	COL27A1
SZ_Risk	scz-denovo-nonsyn	5067	CNTN3
SZ_Risk	scz-denovo-nonsyn	57020	C16orf62
SZ_Risk	scz-denovo-nonsyn	2215	FCGR3B
SZ_Risk	scz-denovo-nonsyn	255275	MYADML2
SZ_Risk	scz-denovo-nonsyn	94239	H2AFV
SZ_Risk	scz-denovo-nonsyn	85461	TANC1
SZ_Risk	scz-denovo-nonsyn	26115	TANC2
SZ_Risk	scz-denovo-nonsyn	64744	SMAP2
SZ_Risk	scz-denovo-nonsyn	51433	ANAPC5
SZ_Risk	scz-denovo-nonsyn	81930	KIF18A
SZ_Risk	scz-denovo-nonsyn	6884	TAF13
SZ_Risk	scz-denovo-nonsyn	9667	SAFB2
SZ_Risk	scz-denovo-nonsyn	4092	SMAD7
SZ_Risk	scz-denovo-nonsyn	2100	ESR2

SZ_Risk	scz-denovo-nonsyn	3866	KRT15
SZ_Risk	scz-denovo-nonsyn	727936	GXYLT2
SZ_Risk	scz-denovo-nonsyn	821	CANX
SZ_Risk	scz-denovo-nonsyn	4282	MIF
SZ_Risk	scz-denovo-nonsyn	9013	TAF1C
SZ_Risk	scz-denovo-nonsyn	53405	CLIC5
SZ_Risk	scz-denovo-nonsyn	284695	ZNF326
SZ_Risk	scz-denovo-nonsyn	6915	TBXA2R
SZ_Risk	scz-denovo-nonsyn	26509	MYOF
SZ_Risk	scz-denovo-nonsyn	211	ALAS1
SZ_Risk	scz-denovo-nonsyn	147920	IGFL2
SZ_Risk	scz-denovo-nonsyn	9821	RB1CC1
SZ_Risk	scz-denovo-nonsyn	7057	THBS1
SZ_Risk	scz-denovo-nonsyn	57787	MARK4
SZ_Risk	scz-denovo-nonsyn	26960	NBEA
SZ_Risk	scz-denovo-nonsyn	54084	TSPEAR
SZ_Risk	scz-denovo-nonsyn	10274	STAG1
SZ_Risk	scz-denovo-nonsyn	51574	LARP7
SZ_Risk	scz-denovo-nonsyn	8344	HIST1H2BE
SZ_Risk	scz-denovo-nonsyn	342667	STAC2
SZ_Risk	scz-denovo-nonsyn	79672	FN3KRP
SZ_Risk	scz-denovo-nonsyn	27258	LSM3
SZ_Risk	scz-denovo-nonsyn	57644	MYH7B
SZ_Risk	scz-denovo-nonsyn	51162	EGFL7
SZ_Risk	scz-denovo-nonsyn	1496	CTNNA2
SZ_Risk	scz-denovo-nonsyn	51533	PHF7
SZ_Risk	scz-denovo-nonsyn	9947	MAGEC1
SZ_Risk	scz-denovo-nonsyn	92667	MGME1
SZ_Risk	scz-denovo-nonsyn	56890	MDM1
SZ_Risk	scz-denovo-nonsyn	60493	FASTKD5
SZ_Risk	scz-denovo-nonsyn	64174	DPEP2
SZ_Risk	scz-denovo-nonsyn	10845	CLPX
SZ_Risk	scz-denovo-nonsyn	55206	SBNO1
SZ_Risk	scz-denovo-nonsyn	5291	PIK3CB
SZ_Risk	scz-denovo-nonsyn	7200	TRH
SZ_Risk	scz-denovo-nonsyn	22856	CHSY1
SZ_Risk	scz-denovo-nonsyn	10342	TFG
SZ_Risk	scz-denovo-nonsyn	5371	PML
SZ_Risk	scz-denovo-nonsyn	3008	HIST1H1E
SZ_Risk	scz-denovo-nonsyn	22872	SEC31A
SZ_Risk	scz-denovo-nonsyn	84629	TNRC18
SZ_Risk	scz-denovo-nonsyn	285367	RPUSD3
SZ_Risk	scz-denovo-nonsyn	56244	BTNL2
SZ_Risk	scz-denovo-nonsyn	3993	LLGL2
SZ_Risk	scz-denovo-nonsyn	284546	C1orf185
SZ_Risk	scz-denovo-nonsyn	5724	PTAFR
SZ_Risk	scz-denovo-nonsyn	9267	CYTH1
SZ_Risk	scz-denovo-nonsyn	85464	SSH2
SZ_Risk	scz-denovo-nonsyn	7798	LUZP1
SZ_Risk	scz-denovo-nonsyn	221458	KIF6
SZ_Risk	scz-denovo-nonsyn	246	ALOX15
SZ_Risk	scz-denovo-nonsyn	10143	CLEC3A
SZ_Risk	scz-denovo-nonsyn	4035	LRP1
SZ_Risk	scz-denovo-nonsyn	7098	TLR3
SZ_Risk	scz-denovo-nonsyn	1739	DLG1
SZ_Risk	scz-denovo-nonsyn	7100	TLR5
SZ_Risk	scz-denovo-nonsyn	1740	DLG2
SZ_Risk	scz-denovo-nonsyn	4038	LRP4

SZ_Risk	scz-denovo-nonsyn	51284	TLR7
SZ_Risk	scz-denovo-nonsyn	22874	PLEKHA6
SZ_Risk	scz-denovo-nonsyn	5361	PLXNA1
SZ_Risk	scz-denovo-nonsyn	23345	SYNE1
SZ_Risk	scz-denovo-nonsyn	23012	STK38L
SZ_Risk	scz-denovo-nonsyn	27177	IL36B
SZ_Risk	scz-denovo-nonsyn	64137	ABCG4
SZ_Risk	scz-denovo-nonsyn	23145	SSPO
SZ_Risk	scz-denovo-nonsyn	7274	TTPA
SZ_Risk	scz-denovo-nonsyn	201595	STT3B
SZ_Risk	scz-denovo-nonsyn	1464	CSPG4
SZ_Risk	scz-denovo-nonsyn	23065	EMC1
SZ_Risk	scz-denovo-nonsyn	23152	CIC
SZ_Risk	scz-denovo-nonsyn	8720	MBTPS1
SZ_Risk	scz-denovo-nonsyn	1983	EIF5
SZ_Risk	scz-denovo-nonsyn	22861	NLRP1
SZ_Risk	scz-denovo-nonsyn	55536	CDCA7L
SZ_Risk	scz-denovo-nonsyn	83856	FSD1L
SZ_Risk	scz-denovo-nonsyn	7486	WRN
SZ_Risk	scz-denovo-nonsyn	90233	ZNF551
SZ_Risk	scz-denovo-nonsyn	2775	GNAO1
SZ_Risk	scz-denovo-nonsyn	55075	UACA
SZ_Risk	scz-denovo-nonsyn	338821	SLCO1B7
SZ_Risk	scz-denovo-nonsyn	3720	JARID2
SZ_Risk	scz-denovo-nonsyn	11113	CIT
SZ_Risk	scz-denovo-nonsyn	60482	SLC5A7
SZ_Risk	scz-denovo-nonsyn	64753	CCDC136
SZ_Risk	scz-denovo-nonsyn	153090	DAB2IP
SZ_Risk	scz-denovo-nonsyn	339230	CCDC137
SZ_Risk	scz-denovo-nonsyn	23126	POGZ
SZ_Risk	scz-denovo-nonsyn	3455	IFNAR2
SZ_Risk	scz-denovo-nonsyn	129607	CMPK2
SZ_Risk	scz-denovo-nonsyn	6601	SMARCC2
SZ_Risk	scz-denovo-nonsyn	814	CAMK4
SZ_Risk	scz-denovo-nonsyn	57332	CBX8
SZ_Risk	scz-denovo-nonsyn	10585	POMT1
SZ_Risk	scz-denovo-nonsyn	63967	CLSPN
SZ_Risk	scz-denovo-nonsyn	5649	RELN
SZ_Risk	scz-denovo-nonsyn	6568	SLC17A1
SZ_Risk	scz-denovo-nonsyn	9213	XPR1
SZ_Risk	scz-denovo-nonsyn	6326	SCN2A
SZ_Risk	scz-denovo-nonsyn	84343	HPS3
SZ_Risk	scz-denovo-nonsyn	122773	KLHDC1
SZ_Risk	scz-denovo-nonsyn	259232	NALCN
SZ_Risk	scz-denovo-nonsyn	283316	CD163L1
SZ_Risk	scz-denovo-nonsyn	60676	PAPPA2
SZ_Risk	scz-denovo-nonsyn	57721	METTL14
SZ_Risk	scz-denovo-nonsyn	339451	KLHL17
SZ_Risk	scz-denovo-nonsyn	90313	TP53I13
SZ_Risk	scz-denovo-nonsyn	2903	GRIN2A
SZ_Risk	scz-denovo-nonsyn	80314	EPC1
SZ_Risk	scz-denovo-nonsyn	9902	MRC2
SZ_Risk	scz-denovo-nonsyn	10994	ILVBL
SZ_Risk	scz-denovo-nonsyn	50944	SHANK1
SZ_Risk	scz-denovo-nonsyn	23259	DDHD2
SZ_Risk	scz-denovo-nonsyn	154661	RUNDC3B
SZ_Risk	scz-denovo-nonsyn	285513	GPRIN3
SZ_Risk	scz-denovo-nonsyn	8857	FCGBP

SZ_Risk	scz-denovo-nonsyn	147657	ZNF480
SZ_Risk	scz-denovo-nonsyn	84705	GTPBP3
SZ_Risk	scz-denovo-nonsyn	9639	ARHGEF10
SZ_Risk	scz-denovo-nonsyn	9826	ARHGEF11
SZ_Risk	scz-denovo-nonsyn	55174	INTS10
SZ_Risk	scz-denovo-nonsyn	60385	TSKS
SZ_Risk	scz-denovo-nonsyn	353274	ZNF445
SZ_Risk	scz-denovo-nonsyn	51663	ZFR
SZ_Risk	scz-denovo-nonsyn	55628	ZNF407
SZ_Risk	scz-denovo-nonsyn	22899	ARHGEF15
SZ_Risk	scz-denovo-nonsyn	2895	GRID2
SZ_Risk	scz-denovo-nonsyn	128025	WDR64
SZ_Risk	scz-denovo-nonsyn	128853	DUSP15
SZ_Risk	scz-denovo-nonsyn	431705	ASTL
SZ_Risk	scz-denovo-nonsyn	3836	KPNA1
SZ_Risk	scz-denovo-nonsyn	80831	APOL5
SZ_Risk	scz-denovo-nonsyn	3156	HMGCR
SZ_Risk	scz-denovo-nonsyn	8565	YARS
SZ_Risk	scz-denovo-nonsyn	8452	CUL3
SZ_Risk	scz-denovo-nonsyn	2783	GNB2
SZ_Risk	scz-denovo-nonsyn	8994	LIMD1
SZ_Risk	scz-denovo-nonsyn	58492	ZNF77
SZ_Risk	scz-denovo-nonsyn	10681	GNB5
SZ_Risk	scz-denovo-nonsyn	23225	NUP210
SZ_Risk	scz-denovo-nonsyn	8470	SORBS2
SZ_Risk	scz-denovo-nonsyn	8431	NR0B2
SZ_Risk	scz-denovo-nonsyn	3655	ITGA6
SZ_Risk	scz-denovo-nonsyn	1855	DVL1
SZ_Risk	scz-denovo-nonsyn	57575	PCDH10
SZ_Risk	scz-denovo-nonsyn	1327	COX4I1
SZ_Risk	scz-denovo-nonsyn	26468	LHX6
SZ_Risk	scz-denovo-nonsyn	23230	VPS13A
SZ_Risk	scz-denovo-nonsyn	79791	FBXO31
SZ_Risk	scz-denovo-nonsyn	9993	DGCR2
SZ_Risk	scz-denovo-nonsyn	54832	VPS13C
SZ_Risk	scz-denovo-nonsyn	23114	NFASC
SZ_Risk	scz-denovo-nonsyn	116985	ARAP1
SZ_Risk	scz-denovo-nonsyn	162517	FBXO39
SZ_Risk	scz-denovo-nonsyn	729767	CEACAM18
SZ_Risk	scz-denovo-nonsyn	54474	KRT20
SZ_Risk	scz-denovo-nonsyn	2134	EXTL1
SZ_Risk	scz-denovo-nonsyn	147183	KRT25
SZ_Risk	scz-denovo-nonsyn	2035	EPB41
SZ_Risk	scz-denovo-nonsyn	3107	HLA-C
SZ_Risk	scz-denovo-nonsyn	56134	PCDHAC2
SZ_Risk	scz-denovo-nonsyn	4588	MUC6
SZ_Risk	scz-denovo-nonsyn	4627	MYH9
SZ_Risk	scz-denovo-nonsyn	25849	PARM1
SZ_Risk	scz-denovo-nonsyn	399909	PCNXL3
SZ_Risk	scz-denovo-nonsyn	6935	ZEB1
SZ_Risk	scz-denovo-nonsyn	415	ARSE
SZ_Risk	scz-denovo-nonsyn	10458	BAIAP2
SZ_Risk	scz-denovo-nonsyn	7840	ALMS1
SZ_Risk	scz-denovo-nonsyn	9510	ADAMTS1
SZ_Risk	scz-denovo-nonsyn	9508	ADAMTS3
SZ_Risk	scz-denovo-nonsyn	113	ADCY7
SZ_Risk	scz-denovo-nonsyn	1306	COL15A1
SZ_Risk	scz-denovo-nonsyn	57700	FAM160B1

SZ_Risk	scz-denovo-nonsyn	11174	ADAMTS6
SZ_Risk	scz-denovo-nonsyn	64760	FAM160B2
SZ_Risk	scz-denovo-nonsyn	28964	GIT1
SZ_Risk	scz-denovo-nonsyn	9140	ATG12
SZ_Risk	scz-denovo-nonsyn	51150	SDF4
SZ_Risk	scz-denovo-nonsyn	1108	CHD4
SZ_Risk	scz-denovo-nonsyn	84872	ZC3H10
SZ_Risk	scz-denovo-nonsyn	55620	STAP2
SZ_Risk	scz-denovo-nonsyn	23523	CABIN1
SZ_Risk	scz-denovo-nonsyn	92714	ARRDC1
SZ_Risk	scz-denovo-nonsyn	64400	AKTIP
SZ_Risk	scz-denovo-nonsyn	55269	PSPC1
SZ_Risk	scz-denovo-nonsyn	6689	SPIB
SZ_Risk	scz-denovo-nonsyn	1912	PHC2
SZ_Risk	scz-denovo-nonsyn	7862	BRPF1
SZ_Risk	scz-denovo-nonsyn	25836	NIPBL
SZ_Risk	scz-denovo-nonsyn	387778	SPDYC
SZ_Risk	scz-denovo-nonsyn	10290	SPEG
SZ_Risk	scz-denovo-nonsyn	23228	PLCL2
SZ_Risk	scz-denovo-nonsyn	26000	TBC1D10B
SZ_Risk	scz-denovo-nonsyn	10075	HUWE1
SZ_Risk	scz-denovo-nonsyn	114784	CSMD2
SZ_Risk	scz-denovo-nonsyn	115704	EVI5L
SZ_Risk	scz-denovo-nonsyn	2532	DARC
SZ_Risk	scz-denovo-nonsyn	7916	PRRC2A
SZ_Risk	scz-denovo-nonsyn	132884	EVC2
SZ_Risk	scz-denovo-nonsyn	23215	PRRC2C
SZ_Risk	scz-denovo-nonsyn	9256	BZRAP1
SZ_Risk	scz-denovo-nonsyn	57002	YAE1D1
SZ_Risk	scz-denovo-nonsyn	26147	PHF19
SZ_Risk	scz-denovo-nonsyn	28981	IFT81
SZ_Risk	scz-denovo-nonsyn	7772	ZNF229
SZ_Risk	scz-denovo-nonsyn	2193	FARSA
SZ_Risk	scz-denovo-nonsyn	5029	P2RY2
SZ_Risk	scz-denovo-nonsyn	57690	TNRC6C
SZ_Risk	scz-denovo-nonsyn	84910	TMEM87B
SZ_Risk	scz-denovo-nonsyn	158056	MAMDC4
SZ_Risk	scz-denovo-nonsyn	126133	ALDH16A1
SZ_Risk	scz-denovo-nonsyn	79165	LENG1
SZ_Risk	scz-denovo-nonsyn	10002	NR2E3
SZ_Risk	scz-denovo-nonsyn	57185	NIPAL3
SZ_Risk	scz-denovo-nonsyn	8813	DPM1
SZ_Risk	scz-denovo-nonsyn	9874	TLK1
SZ_Risk	scz-denovo-nonsyn	2976	GTF3C2
SZ_Risk	scz-denovo-nonsyn	54567	DLL4
SZ_Risk	scz-denovo-nonsyn	26277	TINF2
SZ_Risk	scz-denovo-nonsyn	22897	CEP164
SZ_Risk	scz-denovo-nonsyn	5774	PTPN3
SZ_Risk	scz-denovo-nonsyn	1806	DPYD
SZ_Risk	scz-denovo-nonsyn	400954	EML6
SZ_Risk	scz-denovo-nonsyn	124944	C17orf49
SZ_Risk	scz-denovo-nonsyn	171023	ASXL1
SZ_Risk	scz-denovo-nonsyn	27343	POLL
SZ_Risk	scz-denovo-nonsyn	5792	PTPRF
SZ_Risk	scz-denovo-nonsyn	5793	PTPRG
SZ_Risk	scz-denovo-nonsyn	6263	RYR3
SZ_Risk	scz-denovo-nonsyn	55183	RIF1
SZ_Risk	scz-denovo-nonsyn	147929	ZNF565

SZ_Risk	scz-denovo-nonsyn	5660	PSAP
SZ_Risk	scz-denovo-nonsyn	5795	PTPRJ
SZ_Risk	scz-denovo-nonsyn	90527	DUOXA1
SZ_Risk	scz-denovo-nonsyn	405753	DUOXA2
SZ_Risk	scz-denovo-nonsyn	56000	NXF3
SZ_Risk	scz-denovo-nonsyn	160428	ALDH1L2
SZ_Risk	scz-denovo-nonsyn	5797	PTPRM
SZ_Risk	scz-denovo-nonsyn	54438	GFOD1
SZ_Risk	scz-denovo-nonsyn	7915	ALDH5A1
SZ_Risk	scz-denovo-nonsyn	768239	PSAPL1
SZ_Risk	scz-denovo-nonsyn	22984	PDCD11
SZ_Risk	scz-denovo-nonsyn	255101	CCDC108
SZ_Risk	scz-denovo-nonsyn	154743	C7orf60
SZ_Risk	scz-denovo-nonsyn	4674	NAP1L2
SZ_Risk	scz-denovo-nonsyn	11278	KLF12
SZ_Risk	scz-denovo-nonsyn	27336	HTATSF1
SZ_Risk	scz-denovo-nonsyn	2064	ERBB2
SZ_Risk	scz-denovo-nonsyn	6002	RGS12
SZ_Risk	scz-denovo-nonsyn	63035	BCORL1
SZ_Risk	scz-denovo-nonsyn	5087	PBX1
SZ_Risk	scz-denovo-nonsyn	6793	STK10
SZ_Risk	scz-denovo-nonsyn	23331	TTC28
SZ_Risk	scz-denovo-nonsyn	6561	SLC13A1
SZ_Risk	scz-denovo-nonsyn	7052	TGM2
SZ_Risk	scz-denovo-nonsyn	2335	FN1
SZ_Risk	scz-denovo-nonsyn	9659	PDE4DIP
SZ_Risk	scz-denovo-nonsyn	131544	CRYBG3
SZ_Risk	scz-denovo-nonsyn	1951	CELSR3
SZ_Risk	scz-denovo-nonsyn	9928	KIF14
SZ_Risk	scz-denovo-nonsyn	343641	TGM6
SZ_Risk	scz-denovo-nonsyn	9600	PITPNM1
SZ_Risk	scz-denovo-nonsyn	8831	SYNGAP1
SZ_Risk	scz-denovo-nonsyn	5287	PIK3C2B
SZ_Risk	scz-denovo-nonsyn	149095	DCST1
SZ_Risk	scz-denovo-nonsyn	23039	XPO7
SZ_Risk	scz-denovo-nonsyn	27252	KLHL20
SZ_Risk	scz-denovo-nonsyn	81544	GDPD5
SZ_Risk	scz-denovo-nonsyn	7257	TSNAX
SZ_Risk	scz-denovo-nonsyn	22999	RIMS1
SZ_Risk	scz-denovo-nonsyn	316	AOX1
SZ_Risk	scz-denovo-nonsyn	547	KIF1A
SZ_Risk	scz-denovo-nonsyn	4308	TRPM1
SZ_Risk	scz-denovo-nonsyn	1379	CR1L
SZ_Risk	scz-denovo-nonsyn	27243	CHMP2A
SZ_Risk	scz-denovo-nonsyn	10641	NPRL2
SZ_Risk	scz-denovo-nonsyn	1662	DDX10
SZ_Risk	scz-denovo-nonsyn	387509	GPR153
SZ_Risk	scz-denovo-nonsyn	199953	TMEM201
SZ_Risk	scz-denovo-nonsyn	4843	NOS2
SZ_Risk	scz-denovo-nonsyn	4846	NOS3
SZ_Risk	scz-denovo-nonsyn	79633	FAT4
SZ_Risk	scz-denovo-nonsyn	221393	GPR115
SZ_Risk	scz-denovo-nonsyn	11260	XPOT
SZ_Risk	scz-denovo-nonsyn	64324	NSD1
SZ_Risk	scz-denovo-nonsyn	7126	TNFAIP1
SZ_Risk	scz-denovo-nonsyn	9498	SLC4A8
SZ_Risk	scz-denovo-nonsyn	56478	EIF4ENIF1
SZ_Risk	scz-denovo-nonsyn	23499	MACF1

SZ_Risk	scz-denovo-nonsyn	390110	ACCSL
SZ_Risk	scz-denovo-nonsyn	79023	NUP37
SZ_Risk	scz-denovo-nonsyn	92799	SHKBP1
SZ_Risk	scz-denovo-nonsyn	113146	AHNAK2
SZ_Risk	scz-denovo-nonsyn	55588	MED29
SZ_Risk	scz-denovo-nonsyn	5189	PEX1
SZ_Risk	scz-denovo-nonsyn	5708	PSMD2
SZ_Risk	scz-denovo-nonsyn	440073	IQSEC3
SZ_Risk	scz-denovo-nonsyn	54866	PPP1R14D
SZ_Risk	scz-denovo-nonsyn	283284	IGSF22
SZ_Risk	scz-denovo-nonsyn	55824	PAG1
SZ_Risk	scz-denovo-nonsyn	57182	ANKRD50
SZ_Risk	scz-denovo-nonsyn	146279	TEKT5
SZ_Risk	scz-denovo-nonsyn	23132	RAD54L2
SZ_Risk	scz-denovo-nonsyn	9923	ZBTB40
SZ_Risk	scz-denovo-nonsyn	51050	PI15
SZ_Risk	scz-denovo-nonsyn	150160	CCT8L2
SZ_Risk	scz-denovo-nonsyn	84878	ZBTB45
SZ_Risk	scz-denovo-nonsyn	23037	PDZD2
SZ_Risk	scz-denovo-nonsyn	286	ANK1
SZ_Risk	scz-denovo-nonsyn	3320	HSP90AA1
SZ_Risk	scz-denovo-nonsyn	64285	RHBDF1
SZ_Risk	scz-denovo-nonsyn	64061	TSPYL2
SZ_Risk	scz-denovo-nonsyn	338440	ANO9
SZ_Risk	scz-denovo-nonsyn	2036	EPB41L1
SZ_Risk	scz-denovo-nonsyn	558	AXL
SZ_Risk	scz-denovo-nonsyn	54617	INO80
SZ_Risk	scz-denovo-nonsyn	25962	KIAA1429
SZ_Risk	scz-denovo-nonsyn	128553	TSHZ2
SZ_Risk	scz-denovo-nonsyn	5863	RGL2
SZ_Risk	scz-denovo-nonsyn	54457	TAF7L
SZ_Risk	scz-denovo-nonsyn	9057	SLC7A6
SZ_Risk	scz-denovo-nonsyn	161	AP2A2
SZ_Risk	scz-denovo-nonsyn	100101267	POM121C
SZ_Risk	scz-denovo-nonsyn	83715	ESPN
SZ_Risk	scz-denovo-nonsyn	10659	CELF2
SZ_Risk	scz-denovo-nonsyn	837	CASP4
SZ_Risk	scz-denovo-nonsyn	4628	MYH10
SZ_Risk	scz-denovo-nonsyn	4629	MYH11
SZ_Risk	scz-denovo-nonsyn	1003	CDH5
SZ_Risk	scz-denovo-nonsyn	221955	DAGLB
SZ_Risk	scz-denovo-nonsyn	5579	PRKCB
SZ_Risk	scz-denovo-nonsyn	10560	SLC19A2
SZ_Risk	scz-denovo-nonsyn	55572	FOXRED1
SZ_Risk	scz-denovo-nonsyn	317703	VN1R4
SZ_Risk	scz-denovo-nonsyn	81794	ADAMTS10
SZ_Risk	scz-denovo-nonsyn	6880	TAF9
SZ_Risk	scz-denovo-nonsyn	1207	CLNS1A
SZ_Risk	scz-denovo-nonsyn	155185	AMZ1
SZ_Risk	scz-denovo-nonsyn	7453	WARS
SZ_Risk	scz-denovo-nonsyn	586	BCAT1
SZ_Risk	scz-denovo-nonsyn	84033	OBSCN
SZ_Risk	scz-denovo-nonsyn	51366	UBR5
SZ_Risk	scz-denovo-nonsyn	728621	CCDC30
SZ_Risk	scz-denovo-nonsyn	29979	UBQLN1
SZ_Risk	scz-denovo-nonsyn	79142	PHF23
SZ_Risk	scz-denovo-nonsyn	9875	URB1
SZ_Risk	scz-denovo-nonsyn	9816	URB2

SZ_Risk	scz-denovo-nonsyn	50807	ASAP1
SZ_Risk	scz-denovo-nonsyn	57523	NYNRIN
SZ_Risk	scz-denovo-nonsyn	7272	TTK
SZ_Risk	scz-denovo-nonsyn	339829	CCDC39
SZ_Risk	scz-denovo-nonsyn	2167	FABP4
SZ_Risk	scz-denovo-nonsyn	7273	TTN
SZ_Risk	scz-denovo-nonsyn	23187	PHLDB1
SZ_Risk	scz-denovo-nonsyn	7175	TPR
SZ_Risk	scz-denovo-nonsyn	85460	ZNF518B
SZ_Risk	scz-denovo-nonsyn	23266	LPHN2
SZ_Risk	scz-denovo-nonsyn	84775	ZNF607
SZ_Risk	scz-denovo-nonsyn	129446	XIRP2
SZ_Risk	scz-denovo-nonsyn	51314	NME8
SZ_Risk	scz-denovo-nonsyn	128876	FAM83C
SZ_Risk	scz-denovo-nonsyn	51778	MYOZ2
SZ_Risk	scz-denovo-nonsyn	158471	PRUNE2
SZ_Risk	scz-denovo-nonsyn	2475	MTOR
SZ_Risk	scz-denovo-nonsyn	2736	GLI2
SZ_Risk	scz-denovo-nonsyn	2737	GLI3
SZ_Risk	scz-denovo-nonsyn	80725	SRCIN1
SZ_Risk	scz-denovo-nonsyn	8604	SLC25A12
SZ_Risk	scz-denovo-nonsyn	4796	TONSL
SZ_Risk	scz-denovo-nonsyn	23025	UNC13A
SZ_Risk	scz-denovo-nonsyn	26505	CNNM3
SZ_Risk	scz-denovo-nonsyn	440279	UNC13C
SZ_Risk	scz-denovo-nonsyn	8242	KDM5C
SZ_Risk	scz-denovo-nonsyn	1281	COL3A1
SZ_Risk	scz-denovo-nonsyn	26090	ABHD12
SZ_Risk	scz-denovo-nonsyn	84446	BRSK1
SZ_Risk	scz-denovo-nonsyn	1659	DHX8
SZ_Risk	scz-denovo-nonsyn	54798	DCHS2
SZ_Risk	scz-denovo-nonsyn	9445	ITM2B
SZ_Risk	scz-denovo-nonsyn	81618	ITM2C
SZ_Risk	scz-denovo-nonsyn	55741	EDEM2
SZ_Risk	scz-denovo-nonsyn	83481	EPPK1
SZ_Risk	scz-denovo-nonsyn	22906	TRAK1
SZ_Risk	scz-denovo-nonsyn	255394	TCP11L2
SZ_Risk	scz-denovo-nonsyn	9742	IFT140
SZ_Risk	scz-denovo-nonsyn	1969	EPHA2
SZ_Risk	scz-denovo-nonsyn	400566	C17orf97
SZ_Risk	scz-denovo-nonsyn	399687	MYO18A
SZ_Risk	scz-denovo-nonsyn	57221	KIAA1244
SZ_Risk	scz-denovo-nonsyn	84700	MYO18B
SZ_Risk	scz-denovo-nonsyn	29965	CDIP1
SZ_Risk	scz-denovo-nonsyn	1180	CLCN1
SZ_Risk	scz-denovo-nonsyn	59	ACTA2
SZ_Risk	scz-denovo-nonsyn	348327	ZNF530
SZ_Risk	scz-denovo-nonsyn	6651	SON
SZ_Risk	scz-denovo-nonsyn	55205	ZNF532
SZ_Risk	scz-denovo-nonsyn	5116	PCNT
SZ_Risk	scz-denovo-nonsyn	9332	CD163
SZ_Risk	scz-denovo-nonsyn	119749	OR4C46
SZ_Risk	scz-denovo-nonsyn	9745	ZNF536
SZ_Risk	scz-denovo-nonsyn	6646	SOAT1
SZ_Risk	scz-denovo-nonsyn	23648	SSBP3
SZ_Risk	scz-denovo-nonsyn	9905	SGSM2
SZ_Risk	scz-denovo-nonsyn	84869	CBR4
SZ_Risk	scz-denovo-nonsyn	27044	SND1

SZ_Risk	scz-denovo-nonsyn	3708	ITPR1
SZ_Risk	scz-denovo-nonsyn	5599	MAPK8
SZ_Risk	scz-denovo-nonsyn	25981	DNAH1
SZ_Risk	scz-denovo-nonsyn	51012	SLMO2
SZ_Risk	scz-denovo-nonsyn	55870	ASH1L
SZ_Risk	scz-denovo-nonsyn	51490	C9orf114
SZ_Risk	scz-denovo-nonsyn	1770	DNAH9
SZ_Risk	scz-denovo-nonsyn	157570	ESCO2
SZ_Risk	scz-denovo-nonsyn	1292	COL6A2
SZ_Risk	scz-denovo-nonsyn	57829	ZP4
SZ_Risk	scz-denovo-nonsyn	3363	HTR7
SZ_Risk	scz-denovo-nonsyn	10125	RASGRP1
SZ_Risk	scz-denovo-nonsyn	55959	SULF2
SZ_Risk	scz-denovo-nonsyn	80028	FBXL18
SZ_Risk	scz-denovo-nonsyn	54620	FBXL19
SZ_Risk	scz-denovo-nonsyn	56154	TEX15
SZ_Risk	scz-denovo-nonsyn	207	AKT1
SZ_Risk	scz-denovo-nonsyn	9592	IER2
SZ_Risk	scz-denovo-nonsyn	343263	MYBPHL
SZ_Risk	scz-denovo-nonsyn	4703	NEB
SZ_Risk	scz-denovo-nonsyn	83593	RASSF5
SZ_Risk	scz-denovo-nonsyn	9415	FADS2
SZ_Risk	scz-denovo-nonsyn	388662	SLC6A17
SZ_Risk	scz-denovo-nonsyn	26512	INTS6
SZ_Risk	scz-denovo-nonsyn	2182	ACSL4
SZ_Risk	scz-denovo-nonsyn	51703	ACSL5
SZ_Risk	scz-denovo-nonsyn	1833	EPYC
SZ_Risk	scz-denovo-nonsyn	81033	KCNH6
SZ_Risk	scz-denovo-nonsyn	79088	ZNF426
SZ_Risk	scz-denovo-nonsyn	6125	RPL5
SZ_Risk	scz-denovo-nonsyn	27284	SULT1B1
SZ_Risk	scz-denovo-nonsyn	124446	TMEM219
SZ_Risk	scz-denovo-nonsyn	54848	ARHGEF38
SZ_Risk	scz-denovo-nonsyn	23405	DICER1
SZ_Risk	scz-denovo-nonsyn	11097	NUPL2
SZ_Risk	scz-denovo-nonsyn	84061	MAGT1
SZ_Risk	scz-denovo-nonsyn	84690	SPATA22
SZ_Risk	scz-denovo-nonsyn	29108	PYCARD
SZ_Risk	scz-denovo-nonsyn	56934	CA10
SZ_Risk	scz-denovo-nonsyn	6453	ITSN1
SZ_Risk	scz-denovo-nonsyn	9378	NRXN1
SZ_Risk	scz-denovo-nonsyn	79628	SH3TC2
SZ_Risk	scz-denovo-nonsyn	9043	SPAG9
SZ_Risk	scz-denovo-nonsyn	5079	PAX5
SZ_Risk	scz-denovo-nonsyn	286046	XKR6
SZ_Risk	scz-denovo-nonsyn	6713	SQLE
SZ_Risk	scz-denovo-nonsyn	118932	ANKRD22
SZ_Risk	scz-denovo-nonsyn	389668	XKR9
SZ_Risk	scz-denovo-nonsyn	64781	CERK
SZ_Risk	scz-denovo-nonsyn	23303	KIF13B
SZ_Risk	scz-denovo-nonsyn	196528	ARID2
SZ_Risk	scz-denovo-nonsyn	83741	TFAP2D
SZ_Risk	scz-denovo-nonsyn	134549	SHROOM1
SZ_Risk	scz-denovo-nonsyn	3888	KRT82
SZ_Risk	scz-denovo-nonsyn	91746	YTHDC1
SZ_Risk	scz-denovo-nonsyn	2077	ERF
SZ_Risk	scz-denovo-nonsyn	4350	MPG
SZ_DE	CM_NO_SVA_UP_FDR_0.05	6335	SCN9A

SZ_DE	CM_NO_SVA_UP_FDR_0.05	389206	BEND4
SZ_DE	CM_NO_SVA_UP_FDR_0.05	80055	PGAP1
SZ_DE	CM_NO_SVA_UP_FDR_0.05	6870	TACR3
SZ_DE	CM_NO_SVA_UP_FDR_0.05	55103	RALGPS2
SZ_DE	CM_NO_SVA_UP_FDR_0.05	22881	ANKRD6
SZ_DE	CM_NO_SVA_UP_FDR_0.05	285513	GPRIN3
SZ_DE	CM_NO_SVA_UP_FDR_0.05	80014	WWC2
SZ_DE	CM_NO_SVA_UP_FDR_0.05	5991	RFX3
SZ_DE	CM_NO_SVA_UP_FDR_0.05	2690	GHR
SZ_DE	CM_NO_SVA_UP_FDR_0.05	79669	C3orf52
SZ_DE	CM_NO_SVA_UP_FDR_0.05	117177	RAB3IP
SZ_DE	CM_NO_SVA_UP_FDR_0.05	330	BIRC3
SZ_DE	CM_NO_SVA_UP_FDR_0.05	9750	FAM65B
SZ_DE	CM_NO_SVA_UP_FDR_0.05	116	ADCYAP1
SZ_DE	CM_NO_SVA_UP_FDR_0.05	84458	LCOR
SZ_DE	CM_NO_SVA_UP_FDR_0.05	10100	TSPAN2
SZ_DE	CM_NO_SVA_UP_FDR_0.05	64283	ARHGEF28
SZ_DE	CM_NO_SVA_UP_FDR_0.05	4160	MC4R
SZ_DE	CM_NO_SVA_UP_FDR_0.05	23200	ATP11B
SZ_DE	CM_NO_SVA_UP_FDR_0.05	23002	DAAM1
SZ_DE	CM_NO_SVA_UP_FDR_0.05	6095	RORA
SZ_DE	CM_NO_SVA_UP_FDR_0.05	793	CALB1
SZ_DE	CM_NO_SVA_UP_FDR_0.05	57684	ZBTB26
SZ_DE	CM_NO_SVA_UP_FDR_0.05	128611	ZNF831
SZ_DE	CM_NO_SVA_UP_FDR_0.05	26973	CHORDC1
SZ_DE	CM_NO_SVA_UP_FDR_0.05	2334	AFF2
SZ_DE	CM_NO_SVA_UP_FDR_0.05	54891	INO80D
SZ_DE	CM_NO_SVA_UP_FDR_0.05	23429	RYBP
SZ_DE	CM_NO_SVA_UP_FDR_0.05	10178	TENM1
SZ_DE	CM_NO_SVA_UP_FDR_0.05	154007	SNRNP48
SZ_DE	CM_NO_SVA_UP_FDR_0.05	55466	DNAJA4
SZ_DE	CM_NO_SVA_UP_FDR_0.05	55033	FKBP14
SZ_DE	CM_NO_SVA_UP_FDR_0.05	9991	PTBP3
SZ_DE	CM_NO_SVA_UP_FDR_0.05	80306	MED28
SZ_DE	CM_NO_SVA_UP_FDR_0.05	79139	DERL1
SZ_DE	CM_NO_SVA_UP_FDR_0.05	389677	RBM12B
SZ_DE	CM_NO_SVA_UP_FDR_0.05	2558	GABRA5
SZ_DE	CM_NO_SVA_UP_FDR_0.05	158399	ZNF483
SZ_DE	CM_NO_SVA_UP_FDR_0.05	25896	INTS7
SZ_DE	CM_NO_SVA_UP_FDR_0.05	400120	SERTM1
SZ_DE	CM_NO_SVA_UP_FDR_0.05	3082	HGF
SZ_DE	CM_NO_SVA_UP_FDR_0.05	51762	RAB8B
SZ_DE	CM_NO_SVA_UP_FDR_0.05	5067	CNTN3
SZ_DE	CM_NO_SVA_UP_FDR_0.05	2149	F2R
SZ_DE	CM_NO_SVA_UP_FDR_0.05	10914	PAPOLA
SZ_DE	CM_NO_SVA_UP_FDR_0.05	54726	OTUD4
SZ_DE	CM_NO_SVA_UP_FDR_0.05	54734	RAB39A
SZ_DE	CM_NO_SVA_UP_FDR_0.05	23089	PEG10
SZ_DE	CM_NO_SVA_UP_FDR_0.05	246175	CNOT6L
SZ_DE	CM_NO_SVA_UP_FDR_0.05	3738	KCNA3
SZ_DE	CM_NO_SVA_UP_FDR_0.05	7762	ZNF215
SZ_DE	CM_NO_SVA_UP_FDR_0.05	8658	TNKS
SZ_DE	CM_NO_SVA_UP_FDR_0.05	83448	PUS7L
SZ_DE	CM_NO_SVA_UP_FDR_0.05	4015	LOX
SZ_DE	CM_NO_SVA_UP_FDR_0.05	219623	TMEM26
SZ_DE	CM_NO_SVA_UP_FDR_0.05	11231	SEC63
SZ_DE	CM_NO_SVA_UP_FDR_0.05	57472	CNOT6
SZ_DE	CM_NO_SVA_UP_FDR_0.05	92126	DSEL

SZ_DE	CM_NO_SVA_UP_FDR_0.05	64853	AIDA
SZ_DE	CM_NO_SVA_UP_FDR_0.05	166336	PRICKLE2
SZ_DE	CM_NO_SVA_UP_FDR_0.05	200576	PIKFYVE
SZ_DE	CM_NO_SVA_UP_FDR_0.05	57600	FNIP2
SZ_DE	CM_NO_SVA_UP_FDR_0.05	90134	KCNH7
SZ_DE	CM_NO_SVA_UP_FDR_0.05	23760	PITPNB
SZ_DE	CM_NO_SVA_UP_FDR_0.05	3189	HNRNPH3
SZ_DE	CM_NO_SVA_UP_FDR_0.05	121536	AEBP2
SZ_DE	CM_NO_SVA_UP_FDR_0.05	100216479	LOC100216479
SZ_DE	CM_NO_SVA_UP_FDR_0.05	54778	RNF111
SZ_DE	CM_NO_SVA_UP_FDR_0.05	161357	MDGA2
SZ_DE	CM_NO_SVA_UP_FDR_0.05	64393	ZMAT3
SZ_DE	CM_NO_SVA_UP_FDR_0.05	116150	NUS1
SZ_DE	CM_NO_SVA_UP_FDR_0.05	285220	EPHA6
SZ_DE	CM_NO_SVA_UP_FDR_0.05	8690	JRKL
SZ_DE	CM_NO_SVA_UP_FDR_0.05	5865	RAB3B
SZ_DE	CM_NO_SVA_UP_FDR_0.05	57708	MIER1
SZ_DE	CM_NO_SVA_UP_FDR_0.05	8001	GLRA3
SZ_DE	CM_NO_SVA_UP_FDR_0.05	8976	WASL
SZ_DE	CM_NO_SVA_UP_FDR_0.05	127253	TYW3
SZ_DE	CM_NO_SVA_UP_FDR_0.05	85465	EPT1
SZ_DE	CM_NO_SVA_UP_FDR_0.05	1452	CSNK1A1
SZ_DE	CM_NO_SVA_UP_FDR_0.05	79836	LONRF3
SZ_DE	CM_NO_SVA_UP_FDR_0.05	2673	GFPT1
SZ_DE	CM_NO_SVA_UP_FDR_0.05	22862	FNDC3A
SZ_DE	CM_NO_SVA_UP_FDR_0.05	54989	ZNF770
SZ_DE	CM_NO_SVA_UP_FDR_0.05	256130	TMEM196
SZ_DE	CM_NO_SVA_UP_FDR_0.05	27	ABL2
SZ_DE	CM_NO_SVA_UP_FDR_0.05	57687	VAT1L
SZ_DE	CM_NO_SVA_UP_FDR_0.05	54733	SLC35F2
SZ_DE	CM_NO_SVA_UP_FDR_0.05	1600	DAB1
SZ_DE	CM_NO_SVA_UP_FDR_0.05	10251	SPRY3
SZ_DE	CM_NO_SVA_UP_FDR_0.05	3479	IGF1
SZ_DE	CM_NO_SVA_UP_FDR_0.05	6120	RPE
SZ_DE	CM_NO_SVA_UP_FDR_0.05	64762	GAREM
SZ_DE	CM_NO_SVA_UP_FDR_0.05	25940	FAM98A
SZ_DE	CM_NO_SVA_UP_FDR_0.05	4147	MATN2
SZ_DE	CM_NO_SVA_UP_FDR_0.05	283464	GXYLT1
SZ_DE	CM_NO_SVA_UP_FDR_0.05	64398	MPP5
SZ_DE	CM_NO_SVA_UP_FDR_0.05	55086	CXorf57
SZ_DE	CM_NO_SVA_UP_FDR_0.05	1012	CDH13
SZ_DE	CM_NO_SVA_UP_FDR_0.05	3181	HNRNPA2B1
SZ_DE	CM_NO_SVA_UP_FDR_0.05	253512	SLC25A30
SZ_DE	CM_NO_SVA_UP_FDR_0.05	7077	TIMP2
SZ_DE	CM_NO_SVA_UP_FDR_0.05	650655	ABCA17P
SZ_DE	CM_NO_SVA_UP_FDR_0.05	9874	TLK1
SZ_DE	CM_NO_SVA_UP_FDR_0.05	54542	RC3H2
SZ_DE	CM_NO_SVA_UP_FDR_0.05	317	APAF1
SZ_DE	CM_NO_SVA_UP_FDR_0.05	57484	RNF150
SZ_DE	CM_NO_SVA_UP_FDR_0.05	219333	USP12
SZ_DE	CM_NO_SVA_UP_FDR_0.05	55279	ZNF654
SZ_DE	CM_NO_SVA_UP_FDR_0.05	10903	MTMR11
SZ_DE	CM_NO_SVA_UP_FDR_0.05	166785	MMAA
SZ_DE	CM_NO_SVA_UP_FDR_0.05	5471	PPAT
SZ_DE	CM_NO_SVA_UP_FDR_0.05	129450	TYW5
SZ_DE	CM_NO_SVA_UP_FDR_0.05	146057	TTBK2
SZ_DE	CM_NO_SVA_UP_FDR_0.05	57532	NUFIP2
SZ_DE	CM_NO_SVA_UP_FDR_0.05	85460	ZNF518B

SZ_DE	CM_NO_SVA_UP_FDR_0.05	3320	HSP90AA1
SZ_DE	CM_NO_SVA_UP_FDR_0.05	149041	RC3H1
SZ_DE	CM_NO_SVA_UP_FDR_0.05	11320	MGAT4A
SZ_DE	CM_NO_SVA_UP_FDR_0.05	399959	MIR100HG
SZ_DE	CM_NO_SVA_UP_FDR_0.05	5610	EIF2AK2
SZ_DE	CM_NO_SVA_UP_FDR_0.05	604	BCL6
SZ_DE	CM_NO_SVA_UP_FDR_0.05	1540	CYLD
SZ_DE	CM_NO_SVA_UP_FDR_0.05	8476	CDC42BPA
SZ_DE	CM_NO_SVA_UP_FDR_0.05	162394	SLFN5
SZ_DE	CM_NO_SVA_UP_FDR_0.05	3598	IL13RA2
SZ_DE	CM_NO_SVA_UP_FDR_0.05	79071	ELOVL6
SZ_DE	CM_NO_SVA_UP_FDR_0.05	140890	SREK1
SZ_DE	CM_NO_SVA_UP_FDR_0.05	56829	ZC3HAV1
SZ_DE	CM_NO_SVA_UP_FDR_0.05	101927193	LOC101927193
SZ_DE	CM_NO_SVA_UP_FDR_0.05	390616	ANKRD34C
SZ_DE	CM_NO_SVA_UP_FDR_0.05	8087	FXR1
SZ_DE	CM_NO_SVA_UP_FDR_0.05	51361	HOOK1
SZ_DE	CM_NO_SVA_UP_FDR_0.05	27324	TOX3
SZ_DE	CM_NO_SVA_UP_FDR_0.05	221895	JAZF1
SZ_DE	CM_NO_SVA_UP_FDR_0.05	50650	ARHGEF3
SZ_DE	CM_NO_SVA_UP_FDR_0.05	205	AK4
SZ_DE	CM_NO_SVA_UP_FDR_0.05	6925	TCF4
SZ_DE	CM_NO_SVA_UP_FDR_0.05	5927	KDM5A
SZ_DE	CM_NO_SVA_UP_FDR_0.05	57182	ANKRD50
SZ_DE	CM_NO_SVA_UP_FDR_0.05	167227	DCP2
SZ_DE	CM_NO_SVA_UP_FDR_0.05	7514	XPO1
SZ_DE	CM_NO_SVA_UP_FDR_0.05	56647	BCCIP
SZ_DE	CM_NO_SVA_UP_FDR_0.05	9320	TRIP12
SZ_DE	CM_NO_SVA_UP_FDR_0.05	9877	ZC3H11A
SZ_DE	CM_NO_SVA_UP_FDR_0.05	27146	FAM184B
SZ_DE	CM_NO_SVA_UP_FDR_0.05	27152	INTU
SZ_DE	CM_NO_SVA_UP_FDR_0.05	23411	SIRT1
SZ_DE	CM_NO_SVA_UP_FDR_0.05	92597	MOB1B
SZ_DE	CM_NO_SVA_UP_FDR_0.05	7273	TTN
SZ_DE	CM_NO_SVA_UP_FDR_0.05	159090	FAM122B
SZ_DE	CM_NO_SVA_UP_FDR_0.05	92	ACVR2A
SZ_DE	CM_NO_SVA_UP_FDR_0.05	10146	G3BP1
SZ_DE	CM_NO_SVA_UP_FDR_0.05	55728	N4BP2
SZ_DE	CM_NO_SVA_UP_FDR_0.05	57223	SMEK2
SZ_DE	CM_NO_SVA_UP_FDR_0.05	11052	CPSF6
SZ_DE	CM_NO_SVA_UP_FDR_0.05	4673	NAP1L1
SZ_DE	CM_NO_SVA_UP_FDR_0.05	10884	MRPS30
SZ_DE	CM_NO_SVA_UP_FDR_0.05	139065	SLITRK4
SZ_DE	CM_NO_SVA_UP_FDR_0.05	29068	ZBTB44
SZ_DE	CM_NO_SVA_UP_FDR_0.05	7073	TIAL1
SZ_DE	CM_NO_SVA_UP_FDR_0.05	153222	CREBRF
SZ_DE	CM_NO_SVA_UP_FDR_0.05	56245	C21orf62
SZ_DE	CM_NO_SVA_UP_FDR_0.05	10116	FEM1B
SZ_DE	CM_NO_SVA_UP_FDR_0.05	3192	HNRNPU
SZ_DE	CM_NO_SVA_UP_FDR_0.05	10285	SMNDC1
SZ_DE	CM_NO_SVA_UP_FDR_0.05	100506606	LOC100506606
SZ_DE	CM_NO_SVA_UP_FDR_0.05	3730	KAL1
SZ_DE	CM_NO_SVA_UP_FDR_0.05	64795	RMND5A
SZ_DE	CM_NO_SVA_UP_FDR_0.05	9001	HAP1
SZ_DE	CM_NO_SVA_UP_FDR_0.05	9931	HELZ
SZ_DE	CM_NO_SVA_UP_FDR_0.05	7803	PTP4A1
SZ_DE	CM_NO_SVA_UP_FDR_0.05	4023	LPL
SZ_DE	CM_NO_SVA_UP_FDR_0.05	65065	NBEAL1

SZ_DE	CM_NO_SVA_UP_FDR_0.05	115827	RAB3C
SZ_DE	CM_NO_SVA_UP_FDR_0.05	1809	DPYSL3
SZ_DE	CM_NO_SVA_UP_FDR_0.05	23603	CORO1C
SZ_DE	CM_NO_SVA_UP_FDR_0.05	4154	MBNL1
SZ_DE	CM_NO_SVA_UP_FDR_0.05	403314	APOBEC4
SZ_DE	CM_NO_SVA_UP_FDR_0.05	84919	PPP1R15B
SZ_DE	CM_NO_SVA_UP_FDR_0.05	91851	CHRDL1
SZ_DE	CM_NO_SVA_UP_FDR_0.05	8539	API5
SZ_DE	CM_NO_SVA_UP_FDR_0.05	5033	P4HA1
SZ_DE	CM_NO_SVA_UP_FDR_0.05	22847	ZNF507
SZ_DE	CM_NO_SVA_UP_FDR_0.05	642273	FAM110C
SZ_DE	CM_NO_SVA_UP_FDR_0.05	113251	LARP4
SZ_DE	CM_NO_SVA_UP_FDR_0.05	56937	PMEPA1
SZ_DE	CM_NO_SVA_UP_FDR_0.05	6526	SLC5A3
SZ_DE	CM_NO_SVA_UP_FDR_0.05	56987	BBX
SZ_DE	CM_NO_SVA_UP_FDR_0.05	25849	PARM1
SZ_DE	CM_NO_SVA_UP_FDR_0.05	114294	LACTB
SZ_DE	CM_NO_SVA_UP_FDR_0.05	51141	INSIG2
SZ_DE	CM_NO_SVA_UP_FDR_0.05	23240	KIAA0922
SZ_DE	CM_NO_SVA_UP_FDR_0.05	492311	IGIP
SZ_DE	CM_NO_SVA_UP_FDR_0.05	80351	TNKS2
SZ_DE	CM_NO_SVA_UP_FDR_0.05	7436	VLDLR
SZ_DE	CM_NO_SVA_UP_FDR_0.05	64376	IKZF5
SZ_DE	CM_NO_SVA_UP_FDR_0.05	7223	TRPC4
SZ_DE	CM_NO_SVA_UP_FDR_0.05	100505532	LOC100505532
SZ_DE	CM_NO_SVA_UP_FDR_0.05	283742	FAM98B
SZ_DE	CM_NO_SVA_UP_FDR_0.05	9317	PTER
SZ_DE	CM_NO_SVA_UP_FDR_0.05	8545	CGGBP1
SZ_DE	CM_NO_SVA_UP_FDR_0.05	2562	GABRB3
SZ_DE	CM_NO_SVA_UP_FDR_0.05	23543	RBFOX2
SZ_DE	CM_NO_SVA_UP_FDR_0.05	10154	PLXNC1
SZ_DE	CM_NO_SVA_UP_FDR_0.05	23394	ADNP
SZ_DE	CM_NO_SVA_UP_FDR_0.05	91404	SESTD1
SZ_DE	CM_NO_SVA_UP_FDR_0.05	57403	RAB22A
SZ_DE	CM_NO_SVA_UP_FDR_0.05	80124	VCPIP1
SZ_DE	CM_NO_SVA_UP_FDR_0.05	23210	JMJD6
SZ_DE	CM_NO_SVA_UP_FDR_0.05	22871	NLGN1
SZ_DE	CM_NO_SVA_UP_FDR_0.05	10168	ZNF197
SZ_DE	CM_NO_SVA_UP_FDR_0.05	284370	ZNF615
SZ_DE	CM_NO_SVA_UP_FDR_0.05	8715	NOL4
SZ_DE	CM_NO_SVA_UP_FDR_0.05	80155	NAA15
SZ_DE	CM_NO_SVA_UP_FDR_0.05	147339	C18orf25
SZ_DE	CM_NO_SVA_UP_FDR_0.05	11260	XPOT
SZ_DE	CM_NO_SVA_UP_FDR_0.05	7693	ZNF134
SZ_DE	CM_NO_SVA_UP_FDR_0.05	56986	DTWD1
SZ_DE	CM_NO_SVA_UP_FDR_0.05	8036	SHOC2
SZ_DE	CM_NO_SVA_UP_FDR_0.05	9646	CTR9
SZ_DE	CM_NO_SVA_UP_FDR_0.05	6049	RNF6
SZ_DE	CM_NO_SVA_UP_FDR_0.05	152185	SPICE1
SZ_DE	CM_NO_SVA_UP_FDR_0.05	10622	POLR3G
SZ_DE	CM_NO_SVA_UP_FDR_0.05	140767	NRSN1
SZ_DE	CM_NO_SVA_UP_FDR_0.05	9559	VPS26A
SZ_DE	CM_NO_SVA_UP_FDR_0.05	10424	PGRMC2
SZ_DE	CM_NO_SVA_UP_FDR_0.05	10228	STX6
SZ_DE	CM_NO_SVA_UP_FDR_0.05	636	BICD1
SZ_DE	CM_NO_SVA_UP_FDR_0.05	26052	DNM3
SZ_DE	CM_NO_SVA_UP_FDR_0.05	10746	MAP3K2
SZ_DE	CM_NO_SVA_UP_FDR_0.05	23269	MGA

SZ_DE	CM_NO_SVA_UP_FDR_0.05	144402	CPNE8
SZ_DE	CM_NO_SVA_UP_FDR_0.05	85464	SSH2
SZ_DE	CM_NO_SVA_UP_FDR_0.05	546	ATRX
SZ_DE	CM_NO_SVA_UP_FDR_0.05	55714	TENM3
SZ_DE	CM_NO_SVA_UP_FDR_0.05	6654	SOS1
SZ_DE	CM_NO_SVA_UP_FDR_0.05	10097	ACTR2
SZ_DE	CM_NO_SVA_UP_FDR_0.05	64854	USP46
SZ_DE	CM_NO_SVA_UP_FDR_0.05	55668	GPATCH2L
SZ_DE	CM_NO_SVA_UP_FDR_0.05	57551	TAOK1
SZ_DE	CM_NO_SVA_UP_FDR_0.05	64770	CCDC14
SZ_DE	CM_NO_SVA_UP_FDR_0.05	388558	ZNF808
SZ_DE	CM_NO_SVA_UP_FDR_0.05	84295	PHF6
SZ_DE	CM_NO_SVA_UP_FDR_0.05	116443	GRIN3A
SZ_DE	CM_NO_SVA_UP_FDR_0.05	100381270	ZBED6
SZ_DE	CM_NO_SVA_UP_FDR_0.05	23039	XPO7
SZ_DE	CM_NO_SVA_UP_FDR_0.05	4289	MKLN1
SZ_DE	CM_NO_SVA_UP_FDR_0.05	157807	CLVS1
SZ_DE	CM_NO_SVA_UP_FDR_0.05	6400	SEL1L
SZ_DE	CM_NO_SVA_UP_FDR_0.05	10794	ZNF460
SZ_DE	CM_NO_SVA_UP_FDR_0.05	56886	UGGT1
SZ_DE	CM_NO_SVA_UP_FDR_0.05	55532	SLC30A10
SZ_DE	CM_NO_SVA_UP_FDR_0.05	3837	KPNB1
SZ_DE	CM_NO_SVA_UP_FDR_0.05	688	KLF5
SZ_DE	CM_NO_SVA_UP_FDR_0.05	80223	RAB11FIP1
SZ_DE	CM_NO_SVA_UP_FDR_0.05	8898	MTMR2
SZ_DE	CM_NO_SVA_UP_FDR_0.05	9069	CLDN12
SZ_DE	CM_NO_SVA_UP_FDR_0.05	4084	MXD1
SZ_DE	CM_NO_SVA_UP_FDR_0.05	133746	JMY
SZ_DE	CM_NO_SVA_UP_FDR_0.05	23049	SMG1
SZ_DE	CM_NO_SVA_UP_FDR_0.05	26019	UPF2
SZ_DE	CM_NO_SVA_UP_FDR_0.05	94122	SYTL5
SZ_DE	CM_NO_SVA_UP_FDR_0.05	65267	WNK3
SZ_DE	CM_NO_SVA_UP_FDR_0.05	2005	ELK4
SZ_DE	CM_NO_SVA_UP_FDR_0.05	22908	SACM1L
SZ_DE	CM_NO_SVA_UP_FDR_0.05	23369	PUM2
SZ_DE	CM_NO_SVA_UP_FDR_0.05	101927455	LOC101927455
SZ_DE	CM_NO_SVA_UP_FDR_0.05	5494	PPM1A
SZ_DE	CM_NO_SVA_UP_FDR_0.05	340719	NANOS1
SZ_DE	CM_NO_SVA_UP_FDR_0.05	54665	RSBN1
SZ_DE	CM_NO_SVA_UP_FDR_0.05	5144	PDE4D
SZ_DE	CM_NO_SVA_UP_FDR_0.05	7110	TMF1
SZ_DE	CM_NO_SVA_UP_FDR_0.05	8650	NUMB
SZ_DE	CM_NO_SVA_UP_FDR_0.05	9734	HDAC9
SZ_DE	CM_NO_SVA_UP_FDR_0.05	3763	KCNJ6
SZ_DE	CM_NO_SVA_UP_FDR_0.05	1106	CHD2
SZ_DE	CM_NO_SVA_UP_FDR_0.05	160760	PPTC7
SZ_DE	CM_NO_SVA_UP_FDR_0.05	10808	HSPH1
SZ_DE	CM_NO_SVA_UP_FDR_0.05	79642	ARSJ
SZ_DE	CM_NO_SVA_UP_FDR_0.05	100507388	LOC100507388
SZ_DE	CM_NO_SVA_UP_FDR_0.05	2332	FMR1
SZ_DE	CM_NO_SVA_UP_FDR_0.05	80319	CXXC4
SZ_DE	CM_NO_SVA_UP_FDR_0.05	100131067	CKMT2-AS1
SZ_DE	CM_NO_SVA_UP_FDR_0.05	654502	IQCJ
SZ_DE	CM_NO_SVA_UP_FDR_0.05	23034	SAMD4A
SZ_DE	CM_NO_SVA_UP_FDR_0.05	8916	HERC3
SZ_DE	CM_NO_SVA_UP_FDR_0.05	64282	PAPD5
SZ_DE	CM_NO_SVA_UP_FDR_0.05	3658	IREB2
SZ_DE	CM_NO_SVA_UP_FDR_0.05	84125	LRRIQ1

SZ_DE	CM_NO_SVA_UP_FDR_0.05	152006	RNF38
SZ_DE	CM_NO_SVA_UP_FDR_0.05	22824	HSPA4L
SZ_DE	CM_NO_SVA_UP_FDR_0.05	8527	DGKD
SZ_DE	CM_NO_SVA_UP_FDR_0.05	1739	DLG1
SZ_DE	CM_NO_SVA_UP_FDR_0.05	79834	PEAK1
SZ_DE	CM_NO_SVA_UP_FDR_0.05	27125	AFF4
SZ_DE	CM_NO_SVA_UP_FDR_0.05	5921	RASA1
SZ_DE	CM_NO_SVA_UP_FDR_0.05	84552	PARD6G
SZ_DE	CM_NO_SVA_UP_FDR_0.05	5954	RCN1
SZ_DE	CM_NO_SVA_UP_FDR_0.05	56929	FEM1C
SZ_DE	CM_NO_SVA_UP_FDR_0.05	285636	C5orf51
SZ_DE	CM_NO_SVA_UP_FDR_0.05	57570	TRMT5
SZ_DE	CM_NO_SVA_UP_FDR_0.05	10087	COL4A3BP
SZ_DE	CM_NO_SVA_UP_FDR_0.05	166929	SGMS2
SZ_DE	CM_NO_SVA_UP_FDR_0.05	2066	ERBB4
SZ_DE	CM_NO_SVA_UP_FDR_0.05	57530	CGN
SZ_DE	CM_NO_SVA_UP_FDR_0.05	55787	TXLNG
SZ_DE	CM_NO_SVA_UP_FDR_0.05	1656	DDX6
SZ_DE	CM_NO_SVA_UP_FDR_0.05	8701	DNAH11
SZ_DE	CM_NO_SVA_UP_FDR_0.05	7188	TRAF5
SZ_DE	CM_NO_SVA_DOWN_FDR_0.05	6578	SLCO2A1
SZ_DE	CM_NO_SVA_DOWN_FDR_0.05	216	ALDH1A1
SZ_DE	CM_NO_SVA_DOWN_FDR_0.05	6988	TCTA
SZ_DE	CM_NO_SVA_DOWN_FDR_0.05	375704	ENHO
SZ_DE	CM_NO_SVA_DOWN_FDR_0.05	92241	RCSD1
SZ_DE	CM_NO_SVA_DOWN_FDR_0.05	63941	NECAB3
SZ_DE	CM_NO_SVA_DOWN_FDR_0.05	1869	E2F1
SZ_DE	CM_NO_SVA_DOWN_FDR_0.05	399474	TMEM200B
SZ_DE	CM_NO_SVA_DOWN_FDR_0.05	60495	HPSE2
SZ_DE	CM_NO_SVA_DOWN_FDR_0.05	55806	HR
SZ_DE	CM_NO_SVA_DOWN_FDR_0.05	84572	GNPTG
SZ_DE	CM_NO_SVA_DOWN_FDR_0.05	10234	LRRC17
SZ_DE	CM_NO_SVA_DOWN_FDR_0.05	11152	WDR45
SZ_DE	CM_NO_SVA_DOWN_FDR_0.05	5192	PEX10
SZ_DE	CM_NO_SVA_DOWN_FDR_0.05	148198	ZNF98
SZ_DE	CM_NO_SVA_DOWN_FDR_0.05	10266	RAMP2
SZ_DE	CM_NO_SVA_DOWN_FDR_0.05	56917	MEIS3
SZ_DE	CM_NO_SVA_DOWN_FDR_0.05	50509	COL5A3
SZ_DE	CM_NO_SVA_DOWN_FDR_0.05	818	CAMK2G
SZ_DE	CM_NO_SVA_DOWN_FDR_0.05	5502	PPP1R1A
SZ_DE	CM_NO_SVA_DOWN_FDR_0.05	3481	IGF2
SZ_DE	CM_NO_SVA_DOWN_FDR_0.05	55317	AP5S1
SZ_DE	CM_NO_SVA_DOWN_FDR_0.05	8912	CACNA1H
SZ_DE	CM_NO_SVA_DOWN_FDR_0.05	762	CA4
SZ_DE	CM_NO_SVA_DOWN_FDR_0.05	79041	TMEM38A
SZ_DE	CM_NO_SVA_DOWN_FDR_0.05	5409	PNMT
SZ_DE	CM_NO_SVA_DOWN_FDR_0.05	6324	SCN1B
SZ_DE	CM_NO_SVA_DOWN_FDR_0.05	100133669	LOC100133669
SZ_DE	CM_NO_SVA_DOWN_FDR_0.05	2038	EPB42
SZ_DE	CM_NO_SVA_DOWN_FDR_0.05	572	BAD
SZ_DE	CM_NO_SVA_DOWN_FDR_0.05	53342	IL17D
SZ_DE	CM_NO_SVA_DOWN_FDR_0.05	79095	C9orf16
SZ_DE	CM_NO_SVA_DOWN_FDR_0.05	50649	ARHGEF4
SZ_DE	CM_NO_SVA_DOWN_FDR_0.05	8482	SEMA7A
SZ_DE	CM_NO_SVA_DOWN_FDR_0.05	255275	MYADML2
SZ_DE	CM_NO_SVA_DOWN_FDR_0.05	170463	SSBP4
SZ_DE	CM_NO_SVA_DOWN_FDR_0.05	79785	RERGL
SZ_DE	CM_NO_SVA_DOWN_FDR_0.05	23541	SEC14L2

SZ_DE	CM_NO_SVA_DOWN_FDR_0.05	65988	ZNF747
SZ_DE	CM_NO_SVA_DOWN_FDR_0.05	152002	XXYLT1
SZ_DE	CM_NO_SVA_DOWN_FDR_0.05	7123	CLEC3B
SZ_DE	CM_NO_SVA_DOWN_FDR_0.05	51257	MARCH2
SZ_DE	CM_NO_SVA_DOWN_FDR_0.05	574036	SERTAD4-AS1
SZ_DE	CM_NO_SVA_DOWN_FDR_0.05	1160	CKMT2
SZ_DE	CM_NO_SVA_DOWN_FDR_0.05	8835	SOCS2
SZ_DE	CM_NO_SVA_DOWN_FDR_0.05	8986	RPS6KA4
SZ_DE	CM_NO_SVA_DOWN_FDR_0.05	79947	DHDDS
SZ_DE	CM_NO_SVA_DOWN_FDR_0.05	89958	SAPCD2
SZ_DE	CM_NO_SVA_DOWN_FDR_0.05	56961	SHD
SZ_DE	CM_NO_SVA_DOWN_FDR_0.05	55152	DALRD3
SZ_DE	CM_NO_SVA_DOWN_FDR_0.05	254295	PHYHD1
SZ_DE	CM_NO_SVA_DOWN_FDR_0.05	84681	HINT2
SZ_DE	CM_NO_SVA_DOWN_FDR_0.05	10023	FRAT1
SZ_DE	CM_NO_SVA_DOWN_FDR_0.05	55588	MED29
SZ_DE	CM_NO_SVA_DOWN_FDR_0.05	56603	CYP26B1
SZ_DE	CM_NO_SVA_DOWN_FDR_0.05	2261	FGFR3
SZ_DE	CM_NO_SVA_DOWN_FDR_0.05	54776	PPP1R12C
SZ_DE	CM_NO_SVA_DOWN_FDR_0.05	2155	F7
SZ_DE	CM_NO_SVA_DOWN_FDR_0.05	126792	B3GALT6
SZ_DE	CM_NO_SVA_DOWN_FDR_0.05	283871	PGP
SZ_DE	CM_NO_SVA_DOWN_FDR_0.05	112464	PRKCDBP
SZ_DE	CM_NO_SVA_DOWN_FDR_0.05	9377	COX5A
SZ_DE	CM_NO_SVA_DOWN_FDR_0.05	10814	CPLX2
SZ_DE	CM_NO_SVA_DOWN_FDR_0.05	768096	HAR1A
SZ_DE	CM_NO_SVA_DOWN_FDR_0.05	253868	C20orf166-AS1
SZ_DE	CM_NO_SVA_DOWN_FDR_0.05	64759	TNS3
SZ_DE	CM_NO_SVA_DOWN_FDR_0.05	100507437	LOC100507437
SZ_DE	CM_NO_SVA_DOWN_FDR_0.05	116835	HSPA12B
SZ_DE	CM_NO_SVA_DOWN_FDR_0.05	25870	SUMF2
SZ_DE	CM_NO_SVA_DOWN_FDR_0.05	284312	ZSCAN1
SZ_DE	CM_NO_SVA_DOWN_FDR_0.05	27077	B9D1
SZ_DE	CM_NO_SVA_DOWN_FDR_0.05	4636	MYL5
SZ_DE	CM_NO_SVA_DOWN_FDR_0.05	2047	EPHB1
SZ_DE	CM_NO_SVA_DOWN_FDR_0.05	80199	FUZ
SZ_DE	CM_NO_SVA_DOWN_FDR_0.05	79006	METRN
SZ_DE	CM_NO_SVA_DOWN_FDR_0.05	283130	SLC25A45
SZ_DE	CM_NO_SVA_DOWN_FDR_0.05	138429	PIP5KL1
SZ_DE	CM_NO_SVA_DOWN_FDR_0.05	375775	PNPLA7
SZ_DE	CM_NO_SVA_DOWN_FDR_0.05	8705	B3GALT4
SZ_DE	CM_NO_SVA_DOWN_FDR_0.05	10120	ACTR1B
SZ_DE	CM_NO_SVA_DOWN_FDR_0.05	7138	TNNT1
SZ_DE	CM_NO_SVA_DOWN_FDR_0.05	10485	C1orf61
SZ_DE	CM_NO_SVA_DOWN_FDR_0.05	129804	FBLN7
SZ_DE	CM_NO_SVA_DOWN_FDR_0.05	6881	TAF10
SZ_DE	CM_NO_SVA_DOWN_FDR_0.05	7264	TSTA3
SZ_DE	CM_NO_SVA_DOWN_FDR_0.05	79180	EFHD2
SZ_DE	CM_NO_SVA_DOWN_FDR_0.05	11331	PHB2
SZ_DE	CM_NO_SVA_DOWN_FDR_0.05	56953	NT5M
SZ_DE	CM_NO_SVA_DOWN_FDR_0.05	1346	COX7A1
SZ_DE	CM_NO_SVA_DOWN_FDR_0.05	10361	NPM2
SZ_DE	CM_NO_SVA_DOWN_FDR_0.05	57348	TTYH1
SZ_DE	CM_NO_SVA_DOWN_FDR_0.05	2067	ERCC1
SZ_DE	CM_NO_SVA_DOWN_FDR_0.05	57447	NDRG2
SZ_DE	CM_NO_SVA_DOWN_FDR_0.05	9088	PKMYT1
SZ_DE	CM_NO_SVA_DOWN_FDR_0.05	755	C21orf2
SZ_DE	CM_NO_SVA_DOWN_FDR_0.05	122616	C14orf79

SZ_DE	CM_NO_SVA_DOWN_FDR_0.05	389084	C2orf82
SZ_DE	CM_NO_SVA_DOWN_FDR_0.05	65078	RTN4R
SZ_DE	CM_NO_SVA_DOWN_FDR_0.05	26268	FBXO9
SZ_DE	CM_NO_SVA_DOWN_FDR_0.05	8303	SNN
SZ_DE	CM_NO_SVA_DOWN_FDR_0.05	100192379	PP12613
SZ_DE	CM_NO_SVA_DOWN_FDR_0.05	391356	PTRHD1
SZ_DE	CM_NO_SVA_DOWN_FDR_0.05	84817	TXNDC17
SZ_DE	CM_NO_SVA_DOWN_FDR_0.05	26873	OPLAH
SZ_DE	CM_NO_SVA_DOWN_FDR_0.05	83547	RILP
SZ_DE	CM_NO_SVA_DOWN_FDR_0.05	25864	ABHD14A
SZ_DE	CM_NO_SVA_DOWN_FDR_0.05	64838	FNDC4
SZ_DE	CM_NO_SVA_DOWN_FDR_0.05	339674	LINC00634
SZ_DE	CM_NO_SVA_DOWN_FDR_0.05	84304	NUDT22
SZ_DE	CM_NO_SVA_DOWN_FDR_0.05	3489	IGFBP6
SZ_DE	CM_NO_SVA_DOWN_FDR_0.05	51550	CINP
SZ_DE	CM_NO_SVA_DOWN_FDR_0.05	56848	SPHK2
SZ_DE	CM_NO_SVA_DOWN_FDR_0.05	79873	NUDT18
SZ_DE	CM_NO_SVA_DOWN_FDR_0.05	124936	CYB5D2
SZ_DE	CM_NO_SVA_DOWN_FDR_0.05	56920	SEMA3G
SZ_DE	CM_NO_SVA_DOWN_FDR_0.05	503693	LOH12CR2
SZ_DE	CM_NO_SVA_DOWN_FDR_0.05	6640	SNTA1
SZ_DE	CM_NO_SVA_DOWN_FDR_0.05	23205	ACSBG1
SZ_DE	CM_NO_SVA_DOWN_FDR_0.05	1351	COX8A
SZ_DE	CM_NO_SVA_DOWN_FDR_0.05	90203	SNX21
SZ_DE	CM_NO_SVA_DOWN_FDR_0.05	55257	MRGBP
SZ_DE	CM_NO_SVA_DOWN_FDR_0.05	347734	SLC35B2
SZ_DE	CM_NO_SVA_DOWN_FDR_0.05	84993	UBL7
SZ_DE	CM_NO_SVA_DOWN_FDR_0.05	9283	GPR37L1
SZ_DE	CM_NO_SVA_DOWN_FDR_0.05	154790	CLEC2L
SZ_DE	CM_NO_SVA_DOWN_FDR_0.05	845	CASQ2
SZ_DE	CM_NO_SVA_DOWN_FDR_0.05	6538	SLC6A11
SZ_DE	CM_NO_SVA_DOWN_FDR_0.05	5264	PHYH
SZ_DE	CM_NO_SVA_DOWN_FDR_0.05	51308	REEP2
SZ_DE	CM_NO_SVA_DOWN_FDR_0.05	6540	SLC6A13
SZ_DE	CM_NO_SVA_DOWN_FDR_0.05	151835	CPNE9
SZ_DE	CM_NO_SVA_DOWN_FDR_0.05	11316	COPE
SZ_DE	CM_NO_SVA_DOWN_FDR_0.05	9715	FAM131B
SZ_DE	CM_NO_SVA_DOWN_FDR_0.05	159371	SLC35G1
SZ_DE	CM_NO_SVA_DOWN_FDR_0.05	51162	EGFL7
SZ_DE	CM_NO_SVA_DOWN_FDR_0.05	79754	ASB13
SZ_DE	CM_NO_SVA_DOWN_FDR_0.05	65094	JMJD4
SZ_DE	CM_NO_SVA_DOWN_FDR_0.05	92305	TMEM129
SZ_DE	CM_NO_SVA_DOWN_FDR_0.05	64320	RNF25
SZ_DE	CM_NO_SVA_DOWN_FDR_0.05	1072	CFL1
SZ_DE	CM_NO_SVA_DOWN_FDR_0.05	5127	CDK16
SZ_DE	CM_NO_SVA_DOWN_FDR_0.05	23138	N4BP3
SZ_DE	CM_NO_SVA_DOWN_FDR_0.05	93058	COQ10A
SZ_DE	CM_NO_SVA_DOWN_FDR_0.05	10815	CPLX1
SZ_DE	CM_NO_SVA_DOWN_FDR_0.05	192668	CYS1
SZ_DE	CM_NO_SVA_DOWN_FDR_0.05	9452	ITM2A
SZ_DE	CM_NO_SVA_DOWN_FDR_0.05	2788	GNG7
SZ_DE	CM_NO_SVA_DOWN_FDR_0.05	10445	MCRS1
SZ_DE	CM_NO_SVA_DOWN_FDR_0.05	114984	FLYWCH2
SZ_DE	CM_NO_SVA_DOWN_FDR_0.05	55004	LAMTOR1
SZ_DE	CM_NO_SVA_DOWN_FDR_0.05	1397	CRIP2
SZ_DE	CM_NO_SVA_DOWN_FDR_0.05	101410534	DLGAP1-AS4
SZ_DE	CM_NO_SVA_DOWN_FDR_0.05	51621	KLF13
SZ_DE	CM_NO_SVA_DOWN_FDR_0.05	284358	MAMSTR

SZ_DE	CM_NO_SVA_DOWN_FDR_0.05	3338	DNAJC4
SZ_DE	CM_NO_SVA_DOWN_FDR_0.05	85452	KIAA1751
SZ_DE	CM_NO_SVA_DOWN_FDR_0.05	284076	TTLL6
SZ_DE	CM_NO_SVA_DOWN_FDR_0.05	64236	PDLIM2
SZ_DE	CM_NO_SVA_DOWN_FDR_0.05	339789	LINC00299
SZ_DE	CM_NO_SVA_DOWN_FDR_0.05	5662	PSD
SZ_DE	CM_NO_SVA_DOWN_FDR_0.05	293	SLC25A6
SZ_DE	CM_NO_SVA_DOWN_FDR_0.05	169270	ZNF596
SZ_DE	CM_NO_SVA_DOWN_FDR_0.05	1135	CHRNA2
SZ_DE	CM_NO_SVA_DOWN_FDR_0.05	100507589	NAGPA-AS1
SZ_DE	CM_NO_SVA_DOWN_FDR_0.05	8525	DGKZ
SZ_DE	CM_NO_SVA_DOWN_FDR_0.05	66004	LYNX1
SZ_DE	CM_NO_SVA_DOWN_FDR_0.05	84276	NICN1
SZ_DE	CM_NO_SVA_DOWN_FDR_0.05	6271	S100A1
SZ_DE	CM_NO_SVA_DOWN_FDR_0.05	1465	CSRP1
SZ_DE	CM_NO_SVA_DOWN_FDR_0.05	66035	SLC2A11
SZ_DE	CM_NO_SVA_DOWN_FDR_0.05	285126	DNAJC5G
SZ_DE	CM_NO_SVA_DOWN_FDR_0.05	9244	CRLF1
SZ_DE	CM_NO_SVA_DOWN_FDR_0.05	387640	SKIDA1
SZ_DE	CM_NO_SVA_DOWN_FDR_0.05	64925	CCDC71
SZ_DE	CM_NO_SVA_DOWN_FDR_0.05	138311	FAM69B
SZ_DE	CM_NO_SVA_DOWN_FDR_0.05	1774	DNASE1L1
SZ_DE	CM_NO_SVA_DOWN_FDR_0.05	162427	FAM134C
SZ_DE	CM_NO_SVA_DOWN_FDR_0.05	6948	TCN2
SZ_DE	CM_NO_SVA_DOWN_FDR_0.05	25854	FAM149A
SZ_DE	CM_NO_SVA_DOWN_FDR_0.05	84275	SLC25A33
SZ_DE	CM_NO_SVA_DOWN_FDR_0.05	5878	RAB5C
SZ_DE	CM_NO_SVA_DOWN_FDR_0.05	79412	KREMEN2
SZ_DE	CM_NO_SVA_DOWN_FDR_0.05	138162	C9orf116
SZ_DE	CM_NO_SVA_DOWN_FDR_0.05	5625	PRODH
SZ_DE	CM_NO_SVA_DOWN_FDR_0.05	128439	SNHG11
SZ_DE	CM_NO_SVA_DOWN_FDR_0.05	85442	KNDC1
SZ_DE	CM_NO_SVA_DOWN_FDR_0.05	54587	MXRA8
SZ_DE	CM_NO_SVA_DOWN_FDR_0.05	150383	CDPF1
SZ_DE	CM_NO_SVA_DOWN_FDR_0.05	6624	FSCN1
SZ_DE	CM_NO_SVA_DOWN_FDR_0.05	64743	WDR13
SZ_DE	CM_NO_SVA_DOWN_FDR_0.05	105	ADARB2
SZ_DE	CM_NO_SVA_DOWN_FDR_0.05	84152	PPP1R1B
SZ_DE	CM_NO_SVA_DOWN_FDR_0.05	29086	BABAM1
SZ_DE	CM_NO_SVA_DOWN_FDR_0.05	129787	TMEM18
SZ_DE	CM_NO_SVA_DOWN_FDR_0.05	79671	NLRX1
SZ_DE	CM_NO_SVA_DOWN_FDR_0.05	10522	DEAF1
SZ_DE	CM_NO_SVA_DOWN_FDR_0.05	10400	PEMT
SZ_DE	CM_NO_SVA_DOWN_FDR_0.05	63905	MANBAL
SZ_DE	CM_NO_SVA_DOWN_FDR_0.05	91252	SLC39A13
SZ_DE	CM_NO_SVA_DOWN_FDR_0.05	122970	ACOT4
SZ_DE	CM_NO_SVA_DOWN_FDR_0.05	5201	PFDN1
SZ_DE	CM_NO_SVA_DOWN_FDR_0.05	30815	ST6GALNAC6
SZ_DE	CM_NO_SVA_DOWN_FDR_0.05	286336	FAM78A
SZ_DE	CM_NO_SVA_DOWN_FDR_0.05	84532	ACSS1
SZ_DE	CM_NO_SVA_DOWN_FDR_0.05	148641	SLC35F3
SZ_DE	CM_NO_SVA_DOWN_FDR_0.05	94160	ABCC12
SZ_DE	CM_NO_SVA_DOWN_FDR_0.05	347862	PDDC1
SZ_DE	CM_NO_SVA_DOWN_FDR_0.05	64792	RABL5
SZ_DE	CM_NO_SVA_DOWN_FDR_0.05	54982	CLN6
SZ_DE	CM_NO_SVA_DOWN_FDR_0.05	54884	RETSAT
SZ_DE	CM_NO_SVA_DOWN_FDR_0.05	285368	PRRT3
SZ_DE	CM_NO_SVA_DOWN_FDR_0.05	5859	QARS

SZ_DE	CM_NO_SVA_DOWN_FDR_0.05	80775	TMEM177
SZ_DE	CM_NO_SVA_DOWN_FDR_0.05	363	AQP6
SZ_DE	CM_NO_SVA_DOWN_FDR_0.05	100507206	LINC00943
SZ_DE	CM_NO_SVA_DOWN_FDR_0.05	10460	TACC3
SZ_DE	CM_NO_SVA_DOWN_FDR_0.05	285193	DUSP28
SZ_DE	CM_NO_SVA_DOWN_FDR_0.05	3698	ITIH2
SZ_DE	CM_NO_SVA_DOWN_FDR_0.05	168391	GALNTL5
SZ_DE	CM_NO_SVA_DOWN_FDR_0.05	9957	HS3ST1
SZ_DE	CM_NO_SVA_DOWN_FDR_0.05	8646	CHRD
SZ_DE	CM_NO_SVA_DOWN_FDR_0.05	6687	SPG7
SZ_DE	CM_NO_SVA_DOWN_FDR_0.05	90485	ZNF835
SZ_DE	CM_NO_SVA_DOWN_FDR_0.05	4974	OMG
SZ_DE	CM_NO_SVA_DOWN_FDR_0.05	441869	ANKRD65
SZ_DE	CM_NO_SVA_DOWN_FDR_0.05	1264	CNN1
SZ_DE	CM_NO_SVA_DOWN_FDR_0.05	8991	SELENBP1
SZ_DE	CM_NO_SVA_DOWN_FDR_0.05	150538	SATB2-AS1
SZ_DE	CM_NO_SVA_DOWN_FDR_0.05	161198	CLEC14A
SZ_DE	CM_NO_SVA_DOWN_FDR_0.05	654429	LRTM2
SZ_DE	CM_NO_SVA_DOWN_FDR_0.05	57799	RAB40C
SZ_DE	CM_NO_SVA_DOWN_FDR_0.05	94097	SFXN5
SZ_DE	CM_NO_SVA_DOWN_FDR_0.05	192286	HIGD2A
SZ_DE	CM_NO_SVA_DOWN_FDR_0.05	83444	INO80B
SZ_DE	CM_NO_SVA_DOWN_FDR_0.05	65996	MGC2752
SZ_DE	CM_NO_SVA_DOWN_FDR_0.05	283999	TMEM235
SZ_DE	CM_NO_SVA_DOWN_FDR_0.05	254359	ZDHHC24
SZ_DE	CM_NO_SVA_DOWN_FDR_0.05	11285	B4GALT7
SZ_DE	CM_NO_SVA_DOWN_FDR_0.05	9638	FEZ1
SZ_DE	CM_NO_SVA_DOWN_FDR_0.05	4185	ADAM11
SZ_DE	CM_NO_SVA_DOWN_FDR_0.05	2647	BLOC1S1
SZ_DE	CM_NO_SVA_DOWN_FDR_0.05	83482	SCRT1
SZ_DE	CM_NO_SVA_DOWN_FDR_0.05	9588	PRDX6
SZ_DE	CM_NO_SVA_DOWN_FDR_0.05	3664	IRF6
SZ_DE	CM_NO_SVA_DOWN_FDR_0.05	51706	CYB5R1
SZ_DE	CM_NO_SVA_DOWN_FDR_0.05	388135	C15orf59
SZ_DE	CM_NO_SVA_DOWN_FDR_0.05	28974	C19orf53
SZ_DE	CM_NO_SVA_DOWN_FDR_0.05	51693	TRAPPC2L
SZ_DE	CM_NO_SVA_DOWN_FDR_0.05	10999	SLC27A4
SZ_DE	CM_NO_SVA_DOWN_FDR_0.05	388341	FAM211A
SZ_DE	CM_NO_SVA_DOWN_FDR_0.05	29882	ANAPC2
SZ_DE	CM_NO_SVA_DOWN_FDR_0.05	25900	IFFO1
SZ_DE	CM_NO_SVA_DOWN_FDR_0.05	1468	SLC25A10
SZ_DE	CM_NO_SVA_DOWN_FDR_0.05	6035	RNASE1
SZ_DE	CM_NO_SVA_DOWN_FDR_0.05	2826	CCR10
SZ_DE	CM_NO_SVA_DOWN_FDR_0.05	100128822	LINC01003
SZ_DE	CM_NO_SVA_DOWN_FDR_0.05	379	ARL4D
SZ_DE	CM_NO_SVA_DOWN_FDR_0.05	5605	MAP2K2
SZ_DE	CM_NO_SVA_DOWN_FDR_0.05	25989	ULK3
SZ_DE	CM_NO_SVA_DOWN_FDR_0.05	2582	GALE
SZ_DE	CM_NO_SVA_DOWN_FDR_0.05	9526	MPDU1
SZ_DE	CM_NO_SVA_DOWN_FDR_0.05	3418	IDH2
SZ_DE	CM_NO_SVA_DOWN_FDR_0.05	1289	COL5A1
SZ_DE	CM_NO_SVA_DOWN_FDR_0.05	56954	NIT2
SZ_DE	CM_NO_SVA_DOWN_FDR_0.05	10268	RAMP3
SZ_DE	CM_NO_SVA_DOWN_FDR_0.05	2879	GPX4
SZ_DE	CM_NO_SVA_DOWN_FDR_0.05	9836	LCMT2
SZ_DE	CM_NO_SVA_DOWN_FDR_0.05	183	AGT
SZ_DE	CM_NO_SVA_DOWN_FDR_0.05	254559	LINC00925
SZ_DE	CM_NO_SVA_DOWN_FDR_0.05	114926	SMIM19

SZ_DE	CM_NO_SVA_DOWN_FDR_0.05	11243	PMF1
SZ_DE	CM_NO_SVA_DOWN_FDR_0.05	65263	PYCRL
SZ_DE	CM_NO_SVA_DOWN_FDR_0.05	6415	SEPW1
SZ_DE	CM_NO_SVA_DOWN_FDR_0.05	2702	GJA5
SZ_DE	CM_NO_SVA_DOWN_FDR_0.05	1298	COL9A2
SZ_DE	CM_NO_SVA_DOWN_FDR_0.05	23457	ABCB9
SZ_DE	CM_NO_SVA_DOWN_FDR_0.05	92745	SLC38A5
SZ_DE	CM_NO_SVA_DOWN_FDR_0.05	51024	FIS1
SZ_DE	CM_NO_SVA_DOWN_FDR_0.05	51281	ANKMY1
SZ_DE	CM_NO_SVA_DOWN_FDR_0.05	128218	TMEM125
SZ_DE	CM_NO_SVA_DOWN_FDR_0.05	4005	LMO2
SZ_DE	CM_NO_SVA_DOWN_FDR_0.05	80148	PQLC1
SZ_DE	CM_NO_SVA_DOWN_FDR_0.05	115992	RNF166
SZ_DE	CM_NO_SVA_DOWN_FDR_0.05	1132	CHRM4
SZ_DE	CM_NO_SVA_DOWN_FDR_0.05	7923	HSD17B8
SZ_DE	CM_NO_SVA_DOWN_FDR_0.05	84279	PRADC1
SZ_DE	CM_NO_SVA_DOWN_FDR_0.05	5871	MAP4K2
SZ_DE	CM_NO_SVA_DOWN_FDR_0.05	1299	COL9A3
SZ_DE	CM_NO_SVA_DOWN_FDR_0.05	25946	ZNF385A
SZ_DE	CM_NO_SVA_DOWN_FDR_0.05	28988	DBNL
SZ_DE	CM_NO_SVA_DOWN_FDR_0.05	2356	FPGS
SZ_DE	CM_NO_SVA_DOWN_FDR_0.05	115708	TRMT61A
SZ_DE	CM_NO_SVA_DOWN_FDR_0.05	249	ALPL
SZ_DE	CM_NO_SVA_DOWN_FDR_0.05	100506779	BZRAP1-AS1
SZ_DE	CM_NO_SVA_DOWN_FDR_0.05	738	VPS51
SZ_DE	CM_NO_SVA_DOWN_FDR_0.05	54681	P4HTM
SZ_DE	CM_NO_SVA_DOWN_FDR_0.05	246330	PELI3
SZ_DE	CM_NO_SVA_DOWN_FDR_0.05	6404	SELPLG
SZ_DE	CM_NO_SVA_DOWN_FDR_0.05	84269	CHCHD5
SZ_DE	CM_NO_SVA_DOWN_FDR_0.05	252995	FNDC5
SZ_DE	CM_NO_SVA_DOWN_FDR_0.05	260434	PYDC1
SZ_DE	CM_NO_SVA_DOWN_FDR_0.05	9127	P2RX6
SZ_DE	CM_NO_SVA_DOWN_FDR_0.05	23770	FKBP8
SZ_DE	CM_NO_SVA_DOWN_FDR_0.05	7965	AIMP2
SZ_DE	CM_NO_SVA_DOWN_FDR_0.05	9905	SGSM2
SZ_DE	CM_NO_SVA_DOWN_FDR_0.05	55663	ZNF446
SZ_DE	CM_NO_SVA_DOWN_FDR_0.05	27089	UQCRQ
SZ_DE	CM_NO_SVA_DOWN_FDR_0.05	90187	EMILIN3
SZ_DE	CM_NO_SVA_DOWN_FDR_0.05	56904	SH3GLB2
SZ_DE	CM_NO_SVA_DOWN_FDR_0.05	79590	MRPL24
SZ_DE	CM_NO_SVA_DOWN_FDR_0.05	90990	KIFC2
SZ_DE	CM_NO_SVA_DOWN_FDR_0.05	85378	TUBGCP6
SZ_DE	CM_NO_SVA_DOWN_FDR_0.05	90024	FLJ20021
SZ_DE	CM_NO_SVA_DOWN_FDR_0.05	1149	CIDEA
SZ_DE	CM_NO_SVA_DOWN_FDR_0.05	149345	SHISA4
SZ_DE	CM_NO_SVA_DOWN_FDR_0.05	64419	MTMR14
SZ_DE	CM_NO_SVA_DOWN_FDR_0.05	80142	PTGES2
SZ_DE	CM_NO_SVA_DOWN_FDR_0.05	55851	PSENEN
SZ_DE	CM_NO_SVA_DOWN_FDR_0.05	8704	B4GALT2
SZ_DE	CM_NO_SVA_DOWN_FDR_0.05	6320	CLEC11A
SZ_DE	CM_NO_SVA_DOWN_FDR_0.05	3671	ISLR
SZ_DE	CM_NO_SVA_DOWN_FDR_0.05	474344	GIMAP6
SZ_DE	CM_NO_SVA_DOWN_FDR_0.05	284111	SLC13A5
SZ_DE	CM_NO_SVA_DOWN_FDR_0.05	116349	C5orf55
SZ_DE	CM_NO_SVA_DOWN_FDR_0.05	5524	PPP2R4
SZ_DE	CM_NO_SVA_DOWN_FDR_0.05	387775	SLC22A10
SZ_DE	CM_NO_SVA_DOWN_FDR_0.05	2987	GUK1
SZ_DE	CM_NO_SVA_DOWN_FDR_0.05	84628	NTNG2

SZ_DE	CM_NO_SVA_DOWN_FDR_0.05	89932	PAPLN
SZ_DE	CM_NO_SVA_DOWN_FDR_0.05	54461	FBXW5
SZ_DE	CM_NO_SVA_DOWN_FDR_0.05	113791	PIK3IP1
SZ_DE	CM_NO_SVA_DOWN_FDR_0.05	631	BFSP1
SZ_DE	CM_NO_SVA_DOWN_FDR_0.05	2134	EXTL1
SZ_DE	CM_NO_SVA_DOWN_FDR_0.05	57795	BRINP2
SZ_DE	CM_NO_SVA_DOWN_FDR_0.05	55652	SLC48A1
SZ_DE	CM_NO_SVA_DOWN_FDR_0.05	253982	ASPHD1
SZ_DE	CM_NO_SVA_DOWN_FDR_0.05	129807	NEU4
SZ_DE	CM_NO_SVA_DOWN_FDR_0.05	25906	ANAPC15
SZ_DE	CM_NO_SVA_DOWN_FDR_0.05	2954	GSTZ1
SZ_DE	CM_NO_SVA_DOWN_FDR_0.05	348751	FTCDNL1
SZ_DE	CM_NO_SVA_DOWN_FDR_0.05	80737	VWA7
SZ_DE	CM_NO_SVA_DOWN_FDR_0.05	22845	DOLK
SZ_DE	CM_NO_SVA_DOWN_FDR_0.05	1307	COL16A1
SZ_DE	CM_NO_SVA_DOWN_FDR_0.05	576	BAI2
SZ_DE	CM_NO_SVA_DOWN_FDR_0.05	57128	LYRM4
SZ_DE	CM_NO_SVA_DOWN_FDR_0.05	128240	APOA1BP
SZ_DE	CM_NO_SVA_DOWN_FDR_0.05	6231	RPS26
SZ_DE	CM_NO_SVA_DOWN_FDR_0.05	55808	ST6GALNAC1
SZ_DE	CM_NO_SVA_DOWN_FDR_0.05	8861	LDB1
SZ_DE	CM_NO_SVA_DE_FDR_0.05	6335	SCN9A
SZ_DE	CM_NO_SVA_DE_FDR_0.05	389206	BEND4
SZ_DE	CM_NO_SVA_DE_FDR_0.05	80055	PGAP1
SZ_DE	CM_NO_SVA_DE_FDR_0.05	6870	TACR3
SZ_DE	CM_NO_SVA_DE_FDR_0.05	55103	RALGPS2
SZ_DE	CM_NO_SVA_DE_FDR_0.05	22881	ANKRD6
SZ_DE	CM_NO_SVA_DE_FDR_0.05	285513	GPRIN3
SZ_DE	CM_NO_SVA_DE_FDR_0.05	80014	WWC2
SZ_DE	CM_NO_SVA_DE_FDR_0.05	5991	RFX3
SZ_DE	CM_NO_SVA_DE_FDR_0.05	2690	GHR
SZ_DE	CM_NO_SVA_DE_FDR_0.05	79669	C3orf52
SZ_DE	CM_NO_SVA_DE_FDR_0.05	117177	RAB3IP
SZ_DE	CM_NO_SVA_DE_FDR_0.05	330	BIRC3
SZ_DE	CM_NO_SVA_DE_FDR_0.05	9750	FAM65B
SZ_DE	CM_NO_SVA_DE_FDR_0.05	116	ADCYAP1
SZ_DE	CM_NO_SVA_DE_FDR_0.05	84458	LCOR
SZ_DE	CM_NO_SVA_DE_FDR_0.05	10100	TSPAN2
SZ_DE	CM_NO_SVA_DE_FDR_0.05	64283	ARHGEF28
SZ_DE	CM_NO_SVA_DE_FDR_0.05	4160	MC4R
SZ_DE	CM_NO_SVA_DE_FDR_0.05	23200	ATP11B
SZ_DE	CM_NO_SVA_DE_FDR_0.05	23002	DAAM1
SZ_DE	CM_NO_SVA_DE_FDR_0.05	6095	RORA
SZ_DE	CM_NO_SVA_DE_FDR_0.05	793	CALB1
SZ_DE	CM_NO_SVA_DE_FDR_0.05	57684	ZBTB26
SZ_DE	CM_NO_SVA_DE_FDR_0.05	128611	ZNF831
SZ_DE	CM_NO_SVA_DE_FDR_0.05	26973	CHORDC1
SZ_DE	CM_NO_SVA_DE_FDR_0.05	2334	AFF2
SZ_DE	CM_NO_SVA_DE_FDR_0.05	54891	INO80D
SZ_DE	CM_NO_SVA_DE_FDR_0.05	23429	RYBP
SZ_DE	CM_NO_SVA_DE_FDR_0.05	10178	TENM1
SZ_DE	CM_NO_SVA_DE_FDR_0.05	154007	SNRNP48
SZ_DE	CM_NO_SVA_DE_FDR_0.05	55466	DNAJA4
SZ_DE	CM_NO_SVA_DE_FDR_0.05	55033	FKBP14
SZ_DE	CM_NO_SVA_DE_FDR_0.05	9991	PTBP3
SZ_DE	CM_NO_SVA_DE_FDR_0.05	80306	MED28
SZ_DE	CM_NO_SVA_DE_FDR_0.05	79139	DERL1
SZ_DE	CM_NO_SVA_DE_FDR_0.05	389677	RBM12B

SZ_DE	CM_NO_SVA_DE_FDR_0.05	2558	GABRA5
SZ_DE	CM_NO_SVA_DE_FDR_0.05	158399	ZNF483
SZ_DE	CM_NO_SVA_DE_FDR_0.05	25896	INTS7
SZ_DE	CM_NO_SVA_DE_FDR_0.05	400120	SERTM1
SZ_DE	CM_NO_SVA_DE_FDR_0.05	3082	HGF
SZ_DE	CM_NO_SVA_DE_FDR_0.05	51762	RAB8B
SZ_DE	CM_NO_SVA_DE_FDR_0.05	5067	CNTN3
SZ_DE	CM_NO_SVA_DE_FDR_0.05	2149	F2R
SZ_DE	CM_NO_SVA_DE_FDR_0.05	10914	PAPOLA
SZ_DE	CM_NO_SVA_DE_FDR_0.05	54726	OTUD4
SZ_DE	CM_NO_SVA_DE_FDR_0.05	54734	RAB39A
SZ_DE	CM_NO_SVA_DE_FDR_0.05	23089	PEG10
SZ_DE	CM_NO_SVA_DE_FDR_0.05	246175	CNOT6L
SZ_DE	CM_NO_SVA_DE_FDR_0.05	3738	KCNA3
SZ_DE	CM_NO_SVA_DE_FDR_0.05	7762	ZNF215
SZ_DE	CM_NO_SVA_DE_FDR_0.05	8658	TNKS
SZ_DE	CM_NO_SVA_DE_FDR_0.05	83448	PUS7L
SZ_DE	CM_NO_SVA_DE_FDR_0.05	4015	LOX
SZ_DE	CM_NO_SVA_DE_FDR_0.05	219623	TMEM26
SZ_DE	CM_NO_SVA_DE_FDR_0.05	11231	SEC63
SZ_DE	CM_NO_SVA_DE_FDR_0.05	57472	CNOT6
SZ_DE	CM_NO_SVA_DE_FDR_0.05	92126	DSEL
SZ_DE	CM_NO_SVA_DE_FDR_0.05	64853	AIDA
SZ_DE	CM_NO_SVA_DE_FDR_0.05	166336	PRICKLE2
SZ_DE	CM_NO_SVA_DE_FDR_0.05	200576	PIKFYVE
SZ_DE	CM_NO_SVA_DE_FDR_0.05	57600	FNIP2
SZ_DE	CM_NO_SVA_DE_FDR_0.05	90134	KCNH7
SZ_DE	CM_NO_SVA_DE_FDR_0.05	23760	PITPNB
SZ_DE	CM_NO_SVA_DE_FDR_0.05	3189	HNRNPH3
SZ_DE	CM_NO_SVA_DE_FDR_0.05	121536	AEBP2
SZ_DE	CM_NO_SVA_DE_FDR_0.05	100216479	LOC100216479
SZ_DE	CM_NO_SVA_DE_FDR_0.05	54778	RNF111
SZ_DE	CM_NO_SVA_DE_FDR_0.05	161357	MDGA2
SZ_DE	CM_NO_SVA_DE_FDR_0.05	64393	ZMAT3
SZ_DE	CM_NO_SVA_DE_FDR_0.05	116150	NUS1
SZ_DE	CM_NO_SVA_DE_FDR_0.05	285220	EPHA6
SZ_DE	CM_NO_SVA_DE_FDR_0.05	8690	JRKL
SZ_DE	CM_NO_SVA_DE_FDR_0.05	5865	RAB3B
SZ_DE	CM_NO_SVA_DE_FDR_0.05	57708	MIER1
SZ_DE	CM_NO_SVA_DE_FDR_0.05	8001	GLRA3
SZ_DE	CM_NO_SVA_DE_FDR_0.05	8976	WASL
SZ_DE	CM_NO_SVA_DE_FDR_0.05	127253	TYW3
SZ_DE	CM_NO_SVA_DE_FDR_0.05	85465	EPT1
SZ_DE	CM_NO_SVA_DE_FDR_0.05	1452	CSNK1A1
SZ_DE	CM_NO_SVA_DE_FDR_0.05	79836	LONRF3
SZ_DE	CM_NO_SVA_DE_FDR_0.05	2673	GFPT1
SZ_DE	CM_NO_SVA_DE_FDR_0.05	22862	FNDC3A
SZ_DE	CM_NO_SVA_DE_FDR_0.05	54989	ZNF770
SZ_DE	CM_NO_SVA_DE_FDR_0.05	256130	TMEM196
SZ_DE	CM_NO_SVA_DE_FDR_0.05	27	ABL2
SZ_DE	CM_NO_SVA_DE_FDR_0.05	57687	VAT1L
SZ_DE	CM_NO_SVA_DE_FDR_0.05	54733	SLC35F2
SZ_DE	CM_NO_SVA_DE_FDR_0.05	1600	DAB1
SZ_DE	CM_NO_SVA_DE_FDR_0.05	10251	SPRY3
SZ_DE	CM_NO_SVA_DE_FDR_0.05	3479	IGF1
SZ_DE	CM_NO_SVA_DE_FDR_0.05	6120	RPE
SZ_DE	CM_NO_SVA_DE_FDR_0.05	64762	GAREM
SZ_DE	CM_NO_SVA_DE_FDR_0.05	25940	FAM98A

SZ_DE	CM_NO_SVA_DE_FDR_0.05	4147	MATN2
SZ_DE	CM_NO_SVA_DE_FDR_0.05	283464	GXYLT1
SZ_DE	CM_NO_SVA_DE_FDR_0.05	64398	MPP5
SZ_DE	CM_NO_SVA_DE_FDR_0.05	55086	CXorf57
SZ_DE	CM_NO_SVA_DE_FDR_0.05	1012	CDH13
SZ_DE	CM_NO_SVA_DE_FDR_0.05	3181	HNRNPA2B1
SZ_DE	CM_NO_SVA_DE_FDR_0.05	253512	SLC25A30
SZ_DE	CM_NO_SVA_DE_FDR_0.05	7077	TIMP2
SZ_DE	CM_NO_SVA_DE_FDR_0.05	650655	ABCA17P
SZ_DE	CM_NO_SVA_DE_FDR_0.05	9874	TLK1
SZ_DE	CM_NO_SVA_DE_FDR_0.05	54542	RC3H2
SZ_DE	CM_NO_SVA_DE_FDR_0.05	317	APAF1
SZ_DE	CM_NO_SVA_DE_FDR_0.05	57484	RNF150
SZ_DE	CM_NO_SVA_DE_FDR_0.05	219333	USP12
SZ_DE	CM_NO_SVA_DE_FDR_0.05	55279	ZNF654
SZ_DE	CM_NO_SVA_DE_FDR_0.05	10903	MTMR11
SZ_DE	CM_NO_SVA_DE_FDR_0.05	166785	MMAA
SZ_DE	CM_NO_SVA_DE_FDR_0.05	5471	PPAT
SZ_DE	CM_NO_SVA_DE_FDR_0.05	129450	TYW5
SZ_DE	CM_NO_SVA_DE_FDR_0.05	146057	TTBK2
SZ_DE	CM_NO_SVA_DE_FDR_0.05	57532	NUFIP2
SZ_DE	CM_NO_SVA_DE_FDR_0.05	85460	ZNF518B
SZ_DE	CM_NO_SVA_DE_FDR_0.05	3320	HSP90AA1
SZ_DE	CM_NO_SVA_DE_FDR_0.05	149041	RC3H1
SZ_DE	CM_NO_SVA_DE_FDR_0.05	11320	MGAT4A
SZ_DE	CM_NO_SVA_DE_FDR_0.05	399959	MIR100HG
SZ_DE	CM_NO_SVA_DE_FDR_0.05	5610	EIF2AK2
SZ_DE	CM_NO_SVA_DE_FDR_0.05	604	BCL6
SZ_DE	CM_NO_SVA_DE_FDR_0.05	1540	CYLD
SZ_DE	CM_NO_SVA_DE_FDR_0.05	8476	CDC42BPA
SZ_DE	CM_NO_SVA_DE_FDR_0.05	162394	SLFN5
SZ_DE	CM_NO_SVA_DE_FDR_0.05	3598	IL13RA2
SZ_DE	CM_NO_SVA_DE_FDR_0.05	79071	ELOVL6
SZ_DE	CM_NO_SVA_DE_FDR_0.05	140890	SREK1
SZ_DE	CM_NO_SVA_DE_FDR_0.05	56829	ZC3HAV1
SZ_DE	CM_NO_SVA_DE_FDR_0.05	101927193	LOC101927193
SZ_DE	CM_NO_SVA_DE_FDR_0.05	390616	ANKRD34C
SZ_DE	CM_NO_SVA_DE_FDR_0.05	8087	FXR1
SZ_DE	CM_NO_SVA_DE_FDR_0.05	51361	HOOK1
SZ_DE	CM_NO_SVA_DE_FDR_0.05	27324	TOX3
SZ_DE	CM_NO_SVA_DE_FDR_0.05	221895	JAZF1
SZ_DE	CM_NO_SVA_DE_FDR_0.05	50650	ARHGEF3
SZ_DE	CM_NO_SVA_DE_FDR_0.05	205	AK4
SZ_DE	CM_NO_SVA_DE_FDR_0.05	6925	TCF4
SZ_DE	CM_NO_SVA_DE_FDR_0.05	5927	KDM5A
SZ_DE	CM_NO_SVA_DE_FDR_0.05	57182	ANKRD50
SZ_DE	CM_NO_SVA_DE_FDR_0.05	167227	DCP2
SZ_DE	CM_NO_SVA_DE_FDR_0.05	7514	XPO1
SZ_DE	CM_NO_SVA_DE_FDR_0.05	56647	BCCIP
SZ_DE	CM_NO_SVA_DE_FDR_0.05	9320	TRIP12
SZ_DE	CM_NO_SVA_DE_FDR_0.05	9877	ZC3H11A
SZ_DE	CM_NO_SVA_DE_FDR_0.05	27146	FAM184B
SZ_DE	CM_NO_SVA_DE_FDR_0.05	27152	INTU
SZ_DE	CM_NO_SVA_DE_FDR_0.05	23411	SIRT1
SZ_DE	CM_NO_SVA_DE_FDR_0.05	92597	MOB1B
SZ_DE	CM_NO_SVA_DE_FDR_0.05	7273	TTN
SZ_DE	CM_NO_SVA_DE_FDR_0.05	159090	FAM122B
SZ_DE	CM_NO_SVA_DE_FDR_0.05	92	ACVR2A

SZ_DE	CM_NO_SVA_DE_FDR_0.05	10146	G3BP1
SZ_DE	CM_NO_SVA_DE_FDR_0.05	55728	N4BP2
SZ_DE	CM_NO_SVA_DE_FDR_0.05	57223	SMEK2
SZ_DE	CM_NO_SVA_DE_FDR_0.05	11052	CPSF6
SZ_DE	CM_NO_SVA_DE_FDR_0.05	4673	NAP1L1
SZ_DE	CM_NO_SVA_DE_FDR_0.05	10884	MRPS30
SZ_DE	CM_NO_SVA_DE_FDR_0.05	139065	SLITRK4
SZ_DE	CM_NO_SVA_DE_FDR_0.05	29068	ZBTB44
SZ_DE	CM_NO_SVA_DE_FDR_0.05	7073	TIAL1
SZ_DE	CM_NO_SVA_DE_FDR_0.05	153222	CREBRF
SZ_DE	CM_NO_SVA_DE_FDR_0.05	56245	C21orf62
SZ_DE	CM_NO_SVA_DE_FDR_0.05	10116	FEM1B
SZ_DE	CM_NO_SVA_DE_FDR_0.05	3192	HNRNPU
SZ_DE	CM_NO_SVA_DE_FDR_0.05	10285	SMNDC1
SZ_DE	CM_NO_SVA_DE_FDR_0.05	100506606	LOC100506606
SZ_DE	CM_NO_SVA_DE_FDR_0.05	3730	KAL1
SZ_DE	CM_NO_SVA_DE_FDR_0.05	64795	RMND5A
SZ_DE	CM_NO_SVA_DE_FDR_0.05	9001	HAP1
SZ_DE	CM_NO_SVA_DE_FDR_0.05	9931	HELZ
SZ_DE	CM_NO_SVA_DE_FDR_0.05	7803	PTP4A1
SZ_DE	CM_NO_SVA_DE_FDR_0.05	4023	LPL
SZ_DE	CM_NO_SVA_DE_FDR_0.05	65065	NBEAL1
SZ_DE	CM_NO_SVA_DE_FDR_0.05	115827	RAB3C
SZ_DE	CM_NO_SVA_DE_FDR_0.05	1809	DPYSL3
SZ_DE	CM_NO_SVA_DE_FDR_0.05	23603	CORO1C
SZ_DE	CM_NO_SVA_DE_FDR_0.05	4154	MBNL1
SZ_DE	CM_NO_SVA_DE_FDR_0.05	403314	APOBEC4
SZ_DE	CM_NO_SVA_DE_FDR_0.05	84919	PPP1R15B
SZ_DE	CM_NO_SVA_DE_FDR_0.05	91851	CHRDL1
SZ_DE	CM_NO_SVA_DE_FDR_0.05	8539	API5
SZ_DE	CM_NO_SVA_DE_FDR_0.05	5033	P4HA1
SZ_DE	CM_NO_SVA_DE_FDR_0.05	22847	ZNF507
SZ_DE	CM_NO_SVA_DE_FDR_0.05	642273	FAM110C
SZ_DE	CM_NO_SVA_DE_FDR_0.05	113251	LARP4
SZ_DE	CM_NO_SVA_DE_FDR_0.05	56937	PMEPA1
SZ_DE	CM_NO_SVA_DE_FDR_0.05	6526	SLC5A3
SZ_DE	CM_NO_SVA_DE_FDR_0.05	56987	BBX
SZ_DE	CM_NO_SVA_DE_FDR_0.05	25849	PARM1
SZ_DE	CM_NO_SVA_DE_FDR_0.05	114294	LACTB
SZ_DE	CM_NO_SVA_DE_FDR_0.05	51141	INSIG2
SZ_DE	CM_NO_SVA_DE_FDR_0.05	23240	KIAA0922
SZ_DE	CM_NO_SVA_DE_FDR_0.05	492311	IGIP
SZ_DE	CM_NO_SVA_DE_FDR_0.05	80351	TNKS2
SZ_DE	CM_NO_SVA_DE_FDR_0.05	7436	VLDLR
SZ_DE	CM_NO_SVA_DE_FDR_0.05	64376	IKZF5
SZ_DE	CM_NO_SVA_DE_FDR_0.05	7223	TRPC4
SZ_DE	CM_NO_SVA_DE_FDR_0.05	100505532	LOC100505532
SZ_DE	CM_NO_SVA_DE_FDR_0.05	283742	FAM98B
SZ_DE	CM_NO_SVA_DE_FDR_0.05	9317	PTER
SZ_DE	CM_NO_SVA_DE_FDR_0.05	8545	CGGBP1
SZ_DE	CM_NO_SVA_DE_FDR_0.05	2562	GABRB3
SZ_DE	CM_NO_SVA_DE_FDR_0.05	23543	RBFOX2
SZ_DE	CM_NO_SVA_DE_FDR_0.05	10154	PLXNC1
SZ_DE	CM_NO_SVA_DE_FDR_0.05	23394	ADNP
SZ_DE	CM_NO_SVA_DE_FDR_0.05	91404	SESTD1
SZ_DE	CM_NO_SVA_DE_FDR_0.05	57403	RAB22A
SZ_DE	CM_NO_SVA_DE_FDR_0.05	80124	VCPIP1
SZ_DE	CM_NO_SVA_DE_FDR_0.05	23210	JMJD6

SZ_DE	CM_NO_SVA_DE_FDR_0.05	22871	NLGN1
SZ_DE	CM_NO_SVA_DE_FDR_0.05	10168	ZNF197
SZ_DE	CM_NO_SVA_DE_FDR_0.05	284370	ZNF615
SZ_DE	CM_NO_SVA_DE_FDR_0.05	8715	NOL4
SZ_DE	CM_NO_SVA_DE_FDR_0.05	80155	NAA15
SZ_DE	CM_NO_SVA_DE_FDR_0.05	147339	C18orf25
SZ_DE	CM_NO_SVA_DE_FDR_0.05	11260	XPOT
SZ_DE	CM_NO_SVA_DE_FDR_0.05	7693	ZNF134
SZ_DE	CM_NO_SVA_DE_FDR_0.05	56986	DTWD1
SZ_DE	CM_NO_SVA_DE_FDR_0.05	8036	SHOC2
SZ_DE	CM_NO_SVA_DE_FDR_0.05	9646	CTR9
SZ_DE	CM_NO_SVA_DE_FDR_0.05	6049	RNF6
SZ_DE	CM_NO_SVA_DE_FDR_0.05	152185	SPICE1
SZ_DE	CM_NO_SVA_DE_FDR_0.05	10622	POLR3G
SZ_DE	CM_NO_SVA_DE_FDR_0.05	140767	NRSN1
SZ_DE	CM_NO_SVA_DE_FDR_0.05	9559	VPS26A
SZ_DE	CM_NO_SVA_DE_FDR_0.05	10424	PGRMC2
SZ_DE	CM_NO_SVA_DE_FDR_0.05	10228	STX6
SZ_DE	CM_NO_SVA_DE_FDR_0.05	636	BICD1
SZ_DE	CM_NO_SVA_DE_FDR_0.05	26052	DNM3
SZ_DE	CM_NO_SVA_DE_FDR_0.05	10746	MAP3K2
SZ_DE	CM_NO_SVA_DE_FDR_0.05	23269	MGA
SZ_DE	CM_NO_SVA_DE_FDR_0.05	144402	CPNE8
SZ_DE	CM_NO_SVA_DE_FDR_0.05	85464	SSH2
SZ_DE	CM_NO_SVA_DE_FDR_0.05	546	ATRX
SZ_DE	CM_NO_SVA_DE_FDR_0.05	55714	TENM3
SZ_DE	CM_NO_SVA_DE_FDR_0.05	6654	SOS1
SZ_DE	CM_NO_SVA_DE_FDR_0.05	10097	ACTR2
SZ_DE	CM_NO_SVA_DE_FDR_0.05	64854	USP46
SZ_DE	CM_NO_SVA_DE_FDR_0.05	55668	GPATCH2L
SZ_DE	CM_NO_SVA_DE_FDR_0.05	57551	TAOK1
SZ_DE	CM_NO_SVA_DE_FDR_0.05	64770	CCDC14
SZ_DE	CM_NO_SVA_DE_FDR_0.05	388558	ZNF808
SZ_DE	CM_NO_SVA_DE_FDR_0.05	84295	PHF6
SZ_DE	CM_NO_SVA_DE_FDR_0.05	116443	GRIN3A
SZ_DE	CM_NO_SVA_DE_FDR_0.05	100381270	ZBED6
SZ_DE	CM_NO_SVA_DE_FDR_0.05	23039	XPO7
SZ_DE	CM_NO_SVA_DE_FDR_0.05	4289	MKLN1
SZ_DE	CM_NO_SVA_DE_FDR_0.05	157807	CLVS1
SZ_DE	CM_NO_SVA_DE_FDR_0.05	6400	SEL1L
SZ_DE	CM_NO_SVA_DE_FDR_0.05	10794	ZNF460
SZ_DE	CM_NO_SVA_DE_FDR_0.05	56886	UGGT1
SZ_DE	CM_NO_SVA_DE_FDR_0.05	55532	SLC30A10
SZ_DE	CM_NO_SVA_DE_FDR_0.05	3837	KPNB1
SZ_DE	CM_NO_SVA_DE_FDR_0.05	688	KLF5
SZ_DE	CM_NO_SVA_DE_FDR_0.05	80223	RAB11FIP1
SZ_DE	CM_NO_SVA_DE_FDR_0.05	8898	MTMR2
SZ_DE	CM_NO_SVA_DE_FDR_0.05	9069	CLDN12
SZ_DE	CM_NO_SVA_DE_FDR_0.05	4084	MXD1
SZ_DE	CM_NO_SVA_DE_FDR_0.05	133746	JMY
SZ_DE	CM_NO_SVA_DE_FDR_0.05	23049	SMG1
SZ_DE	CM_NO_SVA_DE_FDR_0.05	26019	UPF2
SZ_DE	CM_NO_SVA_DE_FDR_0.05	94122	SYTL5
SZ_DE	CM_NO_SVA_DE_FDR_0.05	65267	WNK3
SZ_DE	CM_NO_SVA_DE_FDR_0.05	2005	ELK4
SZ_DE	CM_NO_SVA_DE_FDR_0.05	22908	SACM1L
SZ_DE	CM_NO_SVA_DE_FDR_0.05	23369	PUM2
SZ_DE	CM_NO_SVA_DE_FDR_0.05	101927455	LOC101927455

SZ_DE	CM_NO_SVA_DE_FDR_0.05	5494	PPM1A
SZ_DE	CM_NO_SVA_DE_FDR_0.05	340719	NANOS1
SZ_DE	CM_NO_SVA_DE_FDR_0.05	54665	RSBN1
SZ_DE	CM_NO_SVA_DE_FDR_0.05	5144	PDE4D
SZ_DE	CM_NO_SVA_DE_FDR_0.05	7110	TMF1
SZ_DE	CM_NO_SVA_DE_FDR_0.05	8650	NUMB
SZ_DE	CM_NO_SVA_DE_FDR_0.05	9734	HDAC9
SZ_DE	CM_NO_SVA_DE_FDR_0.05	3763	KCNJ6
SZ_DE	CM_NO_SVA_DE_FDR_0.05	1106	CHD2
SZ_DE	CM_NO_SVA_DE_FDR_0.05	160760	PPTC7
SZ_DE	CM_NO_SVA_DE_FDR_0.05	10808	HSPH1
SZ_DE	CM_NO_SVA_DE_FDR_0.05	79642	ARSJ
SZ_DE	CM_NO_SVA_DE_FDR_0.05	100507388	LOC100507388
SZ_DE	CM_NO_SVA_DE_FDR_0.05	2332	FMR1
SZ_DE	CM_NO_SVA_DE_FDR_0.05	80319	CXXC4
SZ_DE	CM_NO_SVA_DE_FDR_0.05	100131067	CKMT2-AS1
SZ_DE	CM_NO_SVA_DE_FDR_0.05	654502	IQCJ
SZ_DE	CM_NO_SVA_DE_FDR_0.05	23034	SAMD4A
SZ_DE	CM_NO_SVA_DE_FDR_0.05	8916	HERC3
SZ_DE	CM_NO_SVA_DE_FDR_0.05	64282	PAPD5
SZ_DE	CM_NO_SVA_DE_FDR_0.05	3658	IREB2
SZ_DE	CM_NO_SVA_DE_FDR_0.05	84125	LRRIQ1
SZ_DE	CM_NO_SVA_DE_FDR_0.05	152006	RNF38
SZ_DE	CM_NO_SVA_DE_FDR_0.05	22824	HSPA4L
SZ_DE	CM_NO_SVA_DE_FDR_0.05	8527	DGKD
SZ_DE	CM_NO_SVA_DE_FDR_0.05	1739	DLG1
SZ_DE	CM_NO_SVA_DE_FDR_0.05	79834	PEAK1
SZ_DE	CM_NO_SVA_DE_FDR_0.05	27125	AFF4
SZ_DE	CM_NO_SVA_DE_FDR_0.05	5921	RASA1
SZ_DE	CM_NO_SVA_DE_FDR_0.05	84552	PARD6G
SZ_DE	CM_NO_SVA_DE_FDR_0.05	5954	RCN1
SZ_DE	CM_NO_SVA_DE_FDR_0.05	56929	FEM1C
SZ_DE	CM_NO_SVA_DE_FDR_0.05	285636	C5orf51
SZ_DE	CM_NO_SVA_DE_FDR_0.05	57570	TRMT5
SZ_DE	CM_NO_SVA_DE_FDR_0.05	10087	COL4A3BP
SZ_DE	CM_NO_SVA_DE_FDR_0.05	166929	SGMS2
SZ_DE	CM_NO_SVA_DE_FDR_0.05	2066	ERBB4
SZ_DE	CM_NO_SVA_DE_FDR_0.05	57530	CGN
SZ_DE	CM_NO_SVA_DE_FDR_0.05	55787	TXLNG
SZ_DE	CM_NO_SVA_DE_FDR_0.05	1656	DDX6
SZ_DE	CM_NO_SVA_DE_FDR_0.05	8701	DNAH11
SZ_DE	CM_NO_SVA_DE_FDR_0.05	7188	TRAF5
SZ_DE	CM_NO_SVA_DE_FDR_0.05	6578	SLCO2A1
SZ_DE	CM_NO_SVA_DE_FDR_0.05	216	ALDH1A1
SZ_DE	CM_NO_SVA_DE_FDR_0.05	6988	TCTA
SZ_DE	CM_NO_SVA_DE_FDR_0.05	375704	ENHO
SZ_DE	CM_NO_SVA_DE_FDR_0.05	92241	RCSD1
SZ_DE	CM_NO_SVA_DE_FDR_0.05	63941	NECAB3
SZ_DE	CM_NO_SVA_DE_FDR_0.05	1869	E2F1
SZ_DE	CM_NO_SVA_DE_FDR_0.05	399474	TMEM200B
SZ_DE	CM_NO_SVA_DE_FDR_0.05	60495	HPSE2
SZ_DE	CM_NO_SVA_DE_FDR_0.05	55806	HR
SZ_DE	CM_NO_SVA_DE_FDR_0.05	84572	GNPTG
SZ_DE	CM_NO_SVA_DE_FDR_0.05	10234	LRRC17
SZ_DE	CM_NO_SVA_DE_FDR_0.05	11152	WDR45
SZ_DE	CM_NO_SVA_DE_FDR_0.05	5192	PEX10
SZ_DE	CM_NO_SVA_DE_FDR_0.05	148198	ZNF98
SZ_DE	CM_NO_SVA_DE_FDR_0.05	10266	RAMP2

SZ_DE	CM_NO_SVA_DE_FDR_0.05	56917	MEIS3
SZ_DE	CM_NO_SVA_DE_FDR_0.05	50509	COL5A3
SZ_DE	CM_NO_SVA_DE_FDR_0.05	818	CAMK2G
SZ_DE	CM_NO_SVA_DE_FDR_0.05	5502	PPP1R1A
SZ_DE	CM_NO_SVA_DE_FDR_0.05	3481	IGF2
SZ_DE	CM_NO_SVA_DE_FDR_0.05	55317	AP5S1
SZ_DE	CM_NO_SVA_DE_FDR_0.05	8912	CACNA1H
SZ_DE	CM_NO_SVA_DE_FDR_0.05	762	CA4
SZ_DE	CM_NO_SVA_DE_FDR_0.05	79041	TMEM38A
SZ_DE	CM_NO_SVA_DE_FDR_0.05	5409	PNMT
SZ_DE	CM_NO_SVA_DE_FDR_0.05	6324	SCN1B
SZ_DE	CM_NO_SVA_DE_FDR_0.05	100133669	LOC100133669
SZ_DE	CM_NO_SVA_DE_FDR_0.05	2038	EPB42
SZ_DE	CM_NO_SVA_DE_FDR_0.05	572	BAD
SZ_DE	CM_NO_SVA_DE_FDR_0.05	53342	IL17D
SZ_DE	CM_NO_SVA_DE_FDR_0.05	79095	C9orf16
SZ_DE	CM_NO_SVA_DE_FDR_0.05	50649	ARHGEF4
SZ_DE	CM_NO_SVA_DE_FDR_0.05	8482	SEMA7A
SZ_DE	CM_NO_SVA_DE_FDR_0.05	255275	MYADML2
SZ_DE	CM_NO_SVA_DE_FDR_0.05	170463	SSBP4
SZ_DE	CM_NO_SVA_DE_FDR_0.05	79785	RERGL
SZ_DE	CM_NO_SVA_DE_FDR_0.05	23541	SEC14L2
SZ_DE	CM_NO_SVA_DE_FDR_0.05	65988	ZNF747
SZ_DE	CM_NO_SVA_DE_FDR_0.05	152002	XXYLT1
SZ_DE	CM_NO_SVA_DE_FDR_0.05	7123	CLEC3B
SZ_DE	CM_NO_SVA_DE_FDR_0.05	51257	MARCH2
SZ_DE	CM_NO_SVA_DE_FDR_0.05	574036	SERTAD4-AS1
SZ_DE	CM_NO_SVA_DE_FDR_0.05	1160	CKMT2
SZ_DE	CM_NO_SVA_DE_FDR_0.05	8835	SOCS2
SZ_DE	CM_NO_SVA_DE_FDR_0.05	8986	RPS6KA4
SZ_DE	CM_NO_SVA_DE_FDR_0.05	79947	DHDDS
SZ_DE	CM_NO_SVA_DE_FDR_0.05	89958	SAPCD2
SZ_DE	CM_NO_SVA_DE_FDR_0.05	56961	SHD
SZ_DE	CM_NO_SVA_DE_FDR_0.05	55152	DALRD3
SZ_DE	CM_NO_SVA_DE_FDR_0.05	254295	PHYHD1
SZ_DE	CM_NO_SVA_DE_FDR_0.05	84681	HINT2
SZ_DE	CM_NO_SVA_DE_FDR_0.05	10023	FRAT1
SZ_DE	CM_NO_SVA_DE_FDR_0.05	55588	MED29
SZ_DE	CM_NO_SVA_DE_FDR_0.05	56603	CYP26B1
SZ_DE	CM_NO_SVA_DE_FDR_0.05	2261	FGFR3
SZ_DE	CM_NO_SVA_DE_FDR_0.05	54776	PPP1R12C
SZ_DE	CM_NO_SVA_DE_FDR_0.05	2155	F7
SZ_DE	CM_NO_SVA_DE_FDR_0.05	126792	B3GALT6
SZ_DE	CM_NO_SVA_DE_FDR_0.05	283871	PGP
SZ_DE	CM_NO_SVA_DE_FDR_0.05	112464	PRKCDBP
SZ_DE	CM_NO_SVA_DE_FDR_0.05	9377	COX5A
SZ_DE	CM_NO_SVA_DE_FDR_0.05	10814	CPLX2
SZ_DE	CM_NO_SVA_DE_FDR_0.05	768096	HAR1A
SZ_DE	CM_NO_SVA_DE_FDR_0.05	253868	C20orf166-AS1
SZ_DE	CM_NO_SVA_DE_FDR_0.05	64759	TNS3
SZ_DE	CM_NO_SVA_DE_FDR_0.05	100507437	LOC100507437
SZ_DE	CM_NO_SVA_DE_FDR_0.05	116835	HSPA12B
SZ_DE	CM_NO_SVA_DE_FDR_0.05	25870	SUMF2
SZ_DE	CM_NO_SVA_DE_FDR_0.05	284312	ZSCAN1
SZ_DE	CM_NO_SVA_DE_FDR_0.05	27077	B9D1
SZ_DE	CM_NO_SVA_DE_FDR_0.05	4636	MYL5
SZ_DE	CM_NO_SVA_DE_FDR_0.05	2047	EPHB1
SZ_DE	CM_NO_SVA_DE_FDR_0.05	80199	FUZ

SZ_DE	CM_NO_SVA_DE_FDR_0.05	79006	METRN
SZ_DE	CM_NO_SVA_DE_FDR_0.05	283130	SLC25A45
SZ_DE	CM_NO_SVA_DE_FDR_0.05	138429	PIP5KL1
SZ_DE	CM_NO_SVA_DE_FDR_0.05	375775	PNPLA7
SZ_DE	CM_NO_SVA_DE_FDR_0.05	8705	B3GALT4
SZ_DE	CM_NO_SVA_DE_FDR_0.05	10120	ACTR1B
SZ_DE	CM_NO_SVA_DE_FDR_0.05	7138	TNNT1
SZ_DE	CM_NO_SVA_DE_FDR_0.05	10485	C1orf61
SZ_DE	CM_NO_SVA_DE_FDR_0.05	129804	FBLN7
SZ_DE	CM_NO_SVA_DE_FDR_0.05	6881	TAF10
SZ_DE	CM_NO_SVA_DE_FDR_0.05	7264	TSTA3
SZ_DE	CM_NO_SVA_DE_FDR_0.05	79180	EFHD2
SZ_DE	CM_NO_SVA_DE_FDR_0.05	11331	PHB2
SZ_DE	CM_NO_SVA_DE_FDR_0.05	56953	NT5M
SZ_DE	CM_NO_SVA_DE_FDR_0.05	1346	COX7A1
SZ_DE	CM_NO_SVA_DE_FDR_0.05	10361	NPM2
SZ_DE	CM_NO_SVA_DE_FDR_0.05	57348	TTYH1
SZ_DE	CM_NO_SVA_DE_FDR_0.05	2067	ERCC1
SZ_DE	CM_NO_SVA_DE_FDR_0.05	57447	NDRG2
SZ_DE	CM_NO_SVA_DE_FDR_0.05	9088	PKMYT1
SZ_DE	CM_NO_SVA_DE_FDR_0.05	755	C21orf2
SZ_DE	CM_NO_SVA_DE_FDR_0.05	122616	C14orf79
SZ_DE	CM_NO_SVA_DE_FDR_0.05	389084	C2orf82
SZ_DE	CM_NO_SVA_DE_FDR_0.05	65078	RTN4R
SZ_DE	CM_NO_SVA_DE_FDR_0.05	26268	FBXO9
SZ_DE	CM_NO_SVA_DE_FDR_0.05	8303	SNN
SZ_DE	CM_NO_SVA_DE_FDR_0.05	100192379	PP12613
SZ_DE	CM_NO_SVA_DE_FDR_0.05	391356	PTRHD1
SZ_DE	CM_NO_SVA_DE_FDR_0.05	84817	TXNDC17
SZ_DE	CM_NO_SVA_DE_FDR_0.05	26873	OPLAH
SZ_DE	CM_NO_SVA_DE_FDR_0.05	83547	RILP
SZ_DE	CM_NO_SVA_DE_FDR_0.05	25864	ABHD14A
SZ_DE	CM_NO_SVA_DE_FDR_0.05	64838	FNDC4
SZ_DE	CM_NO_SVA_DE_FDR_0.05	339674	LINC00634
SZ_DE	CM_NO_SVA_DE_FDR_0.05	84304	NUDT22
SZ_DE	CM_NO_SVA_DE_FDR_0.05	3489	IGFBP6
SZ_DE	CM_NO_SVA_DE_FDR_0.05	51550	CINP
SZ_DE	CM_NO_SVA_DE_FDR_0.05	56848	SPHK2
SZ_DE	CM_NO_SVA_DE_FDR_0.05	79873	NUDT18
SZ_DE	CM_NO_SVA_DE_FDR_0.05	124936	CYB5D2
SZ_DE	CM_NO_SVA_DE_FDR_0.05	56920	SEMA3G
SZ_DE	CM_NO_SVA_DE_FDR_0.05	503693	LOH12CR2
SZ_DE	CM_NO_SVA_DE_FDR_0.05	6640	SNTA1
SZ_DE	CM_NO_SVA_DE_FDR_0.05	23205	ACSBG1
SZ_DE	CM_NO_SVA_DE_FDR_0.05	1351	COX8A
SZ_DE	CM_NO_SVA_DE_FDR_0.05	90203	SNX21
SZ_DE	CM_NO_SVA_DE_FDR_0.05	55257	MRGBP
SZ_DE	CM_NO_SVA_DE_FDR_0.05	347734	SLC35B2
SZ_DE	CM_NO_SVA_DE_FDR_0.05	84993	UBL7
SZ_DE	CM_NO_SVA_DE_FDR_0.05	9283	GPR37L1
SZ_DE	CM_NO_SVA_DE_FDR_0.05	154790	CLEC2L
SZ_DE	CM_NO_SVA_DE_FDR_0.05	845	CASQ2
SZ_DE	CM_NO_SVA_DE_FDR_0.05	6538	SLC6A11
SZ_DE	CM_NO_SVA_DE_FDR_0.05	5264	PHYH
SZ_DE	CM_NO_SVA_DE_FDR_0.05	51308	REEP2
SZ_DE	CM_NO_SVA_DE_FDR_0.05	6540	SLC6A13
SZ_DE	CM_NO_SVA_DE_FDR_0.05	151835	CPNE9
SZ_DE	CM_NO_SVA_DE_FDR_0.05	11316	COPE

SZ_DE	CM_NO_SVA_DE_FDR_0.05	9715	FAM131B
SZ_DE	CM_NO_SVA_DE_FDR_0.05	159371	SLC35G1
SZ_DE	CM_NO_SVA_DE_FDR_0.05	51162	EGFL7
SZ_DE	CM_NO_SVA_DE_FDR_0.05	79754	ASB13
SZ_DE	CM_NO_SVA_DE_FDR_0.05	65094	JMJD4
SZ_DE	CM_NO_SVA_DE_FDR_0.05	92305	TMEM129
SZ_DE	CM_NO_SVA_DE_FDR_0.05	64320	RNF25
SZ_DE	CM_NO_SVA_DE_FDR_0.05	1072	CFL1
SZ_DE	CM_NO_SVA_DE_FDR_0.05	5127	CDK16
SZ_DE	CM_NO_SVA_DE_FDR_0.05	23138	N4BP3
SZ_DE	CM_NO_SVA_DE_FDR_0.05	93058	COQ10A
SZ_DE	CM_NO_SVA_DE_FDR_0.05	10815	CPLX1
SZ_DE	CM_NO_SVA_DE_FDR_0.05	192668	CYS1
SZ_DE	CM_NO_SVA_DE_FDR_0.05	9452	ITM2A
SZ_DE	CM_NO_SVA_DE_FDR_0.05	2788	GNG7
SZ_DE	CM_NO_SVA_DE_FDR_0.05	10445	MCRS1
SZ_DE	CM_NO_SVA_DE_FDR_0.05	114984	FLYWCH2
SZ_DE	CM_NO_SVA_DE_FDR_0.05	55004	LAMTOR1
SZ_DE	CM_NO_SVA_DE_FDR_0.05	1397	CRIP2
SZ_DE	CM_NO_SVA_DE_FDR_0.05	101410534	DLGAP1-AS4
SZ_DE	CM_NO_SVA_DE_FDR_0.05	51621	KLF13
SZ_DE	CM_NO_SVA_DE_FDR_0.05	284358	MAMSTR
SZ_DE	CM_NO_SVA_DE_FDR_0.05	3338	DNAJC4
SZ_DE	CM_NO_SVA_DE_FDR_0.05	85452	KIAA1751
SZ_DE	CM_NO_SVA_DE_FDR_0.05	284076	TTLL6
SZ_DE	CM_NO_SVA_DE_FDR_0.05	64236	PDLIM2
SZ_DE	CM_NO_SVA_DE_FDR_0.05	339789	LINC00299
SZ_DE	CM_NO_SVA_DE_FDR_0.05	5662	PSD
SZ_DE	CM_NO_SVA_DE_FDR_0.05	293	SLC25A6
SZ_DE	CM_NO_SVA_DE_FDR_0.05	169270	ZNF596
SZ_DE	CM_NO_SVA_DE_FDR_0.05	1135	CHRNA2
SZ_DE	CM_NO_SVA_DE_FDR_0.05	100507589	NAGPA-AS1
SZ_DE	CM_NO_SVA_DE_FDR_0.05	8525	DGKZ
SZ_DE	CM_NO_SVA_DE_FDR_0.05	66004	LYNX1
SZ_DE	CM_NO_SVA_DE_FDR_0.05	84276	NICN1
SZ_DE	CM_NO_SVA_DE_FDR_0.05	6271	S100A1
SZ_DE	CM_NO_SVA_DE_FDR_0.05	1465	CSRP1
SZ_DE	CM_NO_SVA_DE_FDR_0.05	66035	SLC2A11
SZ_DE	CM_NO_SVA_DE_FDR_0.05	285126	DNAJC5G
SZ_DE	CM_NO_SVA_DE_FDR_0.05	9244	CRLF1
SZ_DE	CM_NO_SVA_DE_FDR_0.05	387640	SKIDA1
SZ_DE	CM_NO_SVA_DE_FDR_0.05	64925	CCDC71
SZ_DE	CM_NO_SVA_DE_FDR_0.05	138311	FAM69B
SZ_DE	CM_NO_SVA_DE_FDR_0.05	1774	DNASE1L1
SZ_DE	CM_NO_SVA_DE_FDR_0.05	162427	FAM134C
SZ_DE	CM_NO_SVA_DE_FDR_0.05	6948	TCN2
SZ_DE	CM_NO_SVA_DE_FDR_0.05	25854	FAM149A
SZ_DE	CM_NO_SVA_DE_FDR_0.05	84275	SLC25A33
SZ_DE	CM_NO_SVA_DE_FDR_0.05	5878	RAB5C
SZ_DE	CM_NO_SVA_DE_FDR_0.05	79412	KREMEN2
SZ_DE	CM_NO_SVA_DE_FDR_0.05	138162	C9orf116
SZ_DE	CM_NO_SVA_DE_FDR_0.05	5625	PRODH
SZ_DE	CM_NO_SVA_DE_FDR_0.05	128439	SNHG11
SZ_DE	CM_NO_SVA_DE_FDR_0.05	85442	KNDC1
SZ_DE	CM_NO_SVA_DE_FDR_0.05	54587	MXRA8
SZ_DE	CM_NO_SVA_DE_FDR_0.05	150383	CDPF1
SZ_DE	CM_NO_SVA_DE_FDR_0.05	6624	FSCN1
SZ_DE	CM_NO_SVA_DE_FDR_0.05	64743	WDR13

SZ_DE	CM_NO_SVA_DE_FDR_0.05	105	ADARB2
SZ_DE	CM_NO_SVA_DE_FDR_0.05	84152	PPP1R1B
SZ_DE	CM_NO_SVA_DE_FDR_0.05	29086	BABAM1
SZ_DE	CM_NO_SVA_DE_FDR_0.05	129787	TMEM18
SZ_DE	CM_NO_SVA_DE_FDR_0.05	79671	NLRX1
SZ_DE	CM_NO_SVA_DE_FDR_0.05	10522	DEAF1
SZ_DE	CM_NO_SVA_DE_FDR_0.05	10400	PEMT
SZ_DE	CM_NO_SVA_DE_FDR_0.05	63905	MANBAL
SZ_DE	CM_NO_SVA_DE_FDR_0.05	91252	SLC39A13
SZ_DE	CM_NO_SVA_DE_FDR_0.05	122970	ACOT4
SZ_DE	CM_NO_SVA_DE_FDR_0.05	5201	PFDN1
SZ_DE	CM_NO_SVA_DE_FDR_0.05	30815	ST6GALNAC6
SZ_DE	CM_NO_SVA_DE_FDR_0.05	286336	FAM78A
SZ_DE	CM_NO_SVA_DE_FDR_0.05	84532	ACSS1
SZ_DE	CM_NO_SVA_DE_FDR_0.05	148641	SLC35F3
SZ_DE	CM_NO_SVA_DE_FDR_0.05	94160	ABCC12
SZ_DE	CM_NO_SVA_DE_FDR_0.05	347862	PDDC1
SZ_DE	CM_NO_SVA_DE_FDR_0.05	64792	RABL5
SZ_DE	CM_NO_SVA_DE_FDR_0.05	54982	CLN6
SZ_DE	CM_NO_SVA_DE_FDR_0.05	54884	RETSAT
SZ_DE	CM_NO_SVA_DE_FDR_0.05	285368	PRRT3
SZ_DE	CM_NO_SVA_DE_FDR_0.05	5859	QARS
SZ_DE	CM_NO_SVA_DE_FDR_0.05	80775	TMEM177
SZ_DE	CM_NO_SVA_DE_FDR_0.05	363	AQP6
SZ_DE	CM_NO_SVA_DE_FDR_0.05	100507206	LINC00943
SZ_DE	CM_NO_SVA_DE_FDR_0.05	10460	TACC3
SZ_DE	CM_NO_SVA_DE_FDR_0.05	285193	DUSP28
SZ_DE	CM_NO_SVA_DE_FDR_0.05	3698	ITIH2
SZ_DE	CM_NO_SVA_DE_FDR_0.05	168391	GALNTL5
SZ_DE	CM_NO_SVA_DE_FDR_0.05	9957	HS3ST1
SZ_DE	CM_NO_SVA_DE_FDR_0.05	8646	CHRD
SZ_DE	CM_NO_SVA_DE_FDR_0.05	6687	SPG7
SZ_DE	CM_NO_SVA_DE_FDR_0.05	90485	ZNF835
SZ_DE	CM_NO_SVA_DE_FDR_0.05	4974	OMG
SZ_DE	CM_NO_SVA_DE_FDR_0.05	441869	ANKRD65
SZ_DE	CM_NO_SVA_DE_FDR_0.05	1264	CNN1
SZ_DE	CM_NO_SVA_DE_FDR_0.05	8991	SELENBP1
SZ_DE	CM_NO_SVA_DE_FDR_0.05	150538	SATB2-AS1
SZ_DE	CM_NO_SVA_DE_FDR_0.05	161198	CLEC14A
SZ_DE	CM_NO_SVA_DE_FDR_0.05	654429	LRTM2
SZ_DE	CM_NO_SVA_DE_FDR_0.05	57799	RAB40C
SZ_DE	CM_NO_SVA_DE_FDR_0.05	94097	SFXN5
SZ_DE	CM_NO_SVA_DE_FDR_0.05	192286	HIGD2A
SZ_DE	CM_NO_SVA_DE_FDR_0.05	83444	INO80B
SZ_DE	CM_NO_SVA_DE_FDR_0.05	65996	MGC2752
SZ_DE	CM_NO_SVA_DE_FDR_0.05	283999	TMEM235
SZ_DE	CM_NO_SVA_DE_FDR_0.05	254359	ZDHHC24
SZ_DE	CM_NO_SVA_DE_FDR_0.05	11285	B4GALT7
SZ_DE	CM_NO_SVA_DE_FDR_0.05	9638	FEZ1
SZ_DE	CM_NO_SVA_DE_FDR_0.05	4185	ADAM11
SZ_DE	CM_NO_SVA_DE_FDR_0.05	2647	BLOC1S1
SZ_DE	CM_NO_SVA_DE_FDR_0.05	83482	SCRT1
SZ_DE	CM_NO_SVA_DE_FDR_0.05	9588	PRDX6
SZ_DE	CM_NO_SVA_DE_FDR_0.05	3664	IRF6
SZ_DE	CM_NO_SVA_DE_FDR_0.05	51706	CYB5R1
SZ_DE	CM_NO_SVA_DE_FDR_0.05	388135	C15orf59
SZ_DE	CM_NO_SVA_DE_FDR_0.05	28974	C19orf53
SZ_DE	CM_NO_SVA_DE_FDR_0.05	51693	TRAPPC2L

SZ_DE	CM_NO_SVA_DE_FDR_0.05	10999	SLC27A4
SZ_DE	CM_NO_SVA_DE_FDR_0.05	388341	FAM211A
SZ_DE	CM_NO_SVA_DE_FDR_0.05	29882	ANAPC2
SZ_DE	CM_NO_SVA_DE_FDR_0.05	25900	IFFO1
SZ_DE	CM_NO_SVA_DE_FDR_0.05	1468	SLC25A10
SZ_DE	CM_NO_SVA_DE_FDR_0.05	6035	RNASE1
SZ_DE	CM_NO_SVA_DE_FDR_0.05	2826	CCR10
SZ_DE	CM_NO_SVA_DE_FDR_0.05	100128822	LINC01003
SZ_DE	CM_NO_SVA_DE_FDR_0.05	379	ARL4D
SZ_DE	CM_NO_SVA_DE_FDR_0.05	5605	MAP2K2
SZ_DE	CM_NO_SVA_DE_FDR_0.05	25989	ULK3
SZ_DE	CM_NO_SVA_DE_FDR_0.05	2582	GALE
SZ_DE	CM_NO_SVA_DE_FDR_0.05	9526	MPDU1
SZ_DE	CM_NO_SVA_DE_FDR_0.05	3418	IDH2
SZ_DE	CM_NO_SVA_DE_FDR_0.05	1289	COL5A1
SZ_DE	CM_NO_SVA_DE_FDR_0.05	56954	NIT2
SZ_DE	CM_NO_SVA_DE_FDR_0.05	10268	RAMP3
SZ_DE	CM_NO_SVA_DE_FDR_0.05	2879	GPX4
SZ_DE	CM_NO_SVA_DE_FDR_0.05	9836	LCMT2
SZ_DE	CM_NO_SVA_DE_FDR_0.05	183	AGT
SZ_DE	CM_NO_SVA_DE_FDR_0.05	254559	LINC00925
SZ_DE	CM_NO_SVA_DE_FDR_0.05	114926	SMIM19
SZ_DE	CM_NO_SVA_DE_FDR_0.05	11243	PMF1
SZ_DE	CM_NO_SVA_DE_FDR_0.05	65263	PYCRL
SZ_DE	CM_NO_SVA_DE_FDR_0.05	6415	SEPW1
SZ_DE	CM_NO_SVA_DE_FDR_0.05	2702	GJA5
SZ_DE	CM_NO_SVA_DE_FDR_0.05	1298	COL9A2
SZ_DE	CM_NO_SVA_DE_FDR_0.05	23457	ABCB9
SZ_DE	CM_NO_SVA_DE_FDR_0.05	92745	SLC38A5
SZ_DE	CM_NO_SVA_DE_FDR_0.05	51024	FIS1
SZ_DE	CM_NO_SVA_DE_FDR_0.05	51281	ANKMY1
SZ_DE	CM_NO_SVA_DE_FDR_0.05	128218	TMEM125
SZ_DE	CM_NO_SVA_DE_FDR_0.05	4005	LMO2
SZ_DE	CM_NO_SVA_DE_FDR_0.05	80148	PQLC1
SZ_DE	CM_NO_SVA_DE_FDR_0.05	115992	RNF166
SZ_DE	CM_NO_SVA_DE_FDR_0.05	1132	CHRM4
SZ_DE	CM_NO_SVA_DE_FDR_0.05	7923	HSD17B8
SZ_DE	CM_NO_SVA_DE_FDR_0.05	84279	PRADC1
SZ_DE	CM_NO_SVA_DE_FDR_0.05	5871	MAP4K2
SZ_DE	CM_NO_SVA_DE_FDR_0.05	1299	COL9A3
SZ_DE	CM_NO_SVA_DE_FDR_0.05	25946	ZNF385A
SZ_DE	CM_NO_SVA_DE_FDR_0.05	28988	DBNL
SZ_DE	CM_NO_SVA_DE_FDR_0.05	2356	FPGS
SZ_DE	CM_NO_SVA_DE_FDR_0.05	115708	TRMT61A
SZ_DE	CM_NO_SVA_DE_FDR_0.05	249	ALPL
SZ_DE	CM_NO_SVA_DE_FDR_0.05	100506779	BZRAP1-AS1
SZ_DE	CM_NO_SVA_DE_FDR_0.05	738	VPS51
SZ_DE	CM_NO_SVA_DE_FDR_0.05	54681	P4HTM
SZ_DE	CM_NO_SVA_DE_FDR_0.05	246330	PELI3
SZ_DE	CM_NO_SVA_DE_FDR_0.05	6404	SELPLG
SZ_DE	CM_NO_SVA_DE_FDR_0.05	84269	CHCHD5
SZ_DE	CM_NO_SVA_DE_FDR_0.05	252995	FNDC5
SZ_DE	CM_NO_SVA_DE_FDR_0.05	260434	PYDC1
SZ_DE	CM_NO_SVA_DE_FDR_0.05	9127	P2RX6
SZ_DE	CM_NO_SVA_DE_FDR_0.05	23770	FKBP8
SZ_DE	CM_NO_SVA_DE_FDR_0.05	7965	AIMP2
SZ_DE	CM_NO_SVA_DE_FDR_0.05	9905	SGSM2
SZ_DE	CM_NO_SVA_DE_FDR_0.05	55663	ZNF446

SZ_DE	CM_NO_SVA_DE_FDR_0.05	27089	UQCRQ
SZ_DE	CM_NO_SVA_DE_FDR_0.05	90187	EMILIN3
SZ_DE	CM_NO_SVA_DE_FDR_0.05	56904	SH3GLB2
SZ_DE	CM_NO_SVA_DE_FDR_0.05	79590	MRPL24
SZ_DE	CM_NO_SVA_DE_FDR_0.05	90990	KIFC2
SZ_DE	CM_NO_SVA_DE_FDR_0.05	85378	TUBGCP6
SZ_DE	CM_NO_SVA_DE_FDR_0.05	90024	FLJ20021
SZ_DE	CM_NO_SVA_DE_FDR_0.05	1149	CIDEA
SZ_DE	CM_NO_SVA_DE_FDR_0.05	149345	SHISA4
SZ_DE	CM_NO_SVA_DE_FDR_0.05	64419	MTMR14
SZ_DE	CM_NO_SVA_DE_FDR_0.05	80142	PTGES2
SZ_DE	CM_NO_SVA_DE_FDR_0.05	55851	PSENEN
SZ_DE	CM_NO_SVA_DE_FDR_0.05	8704	B4GALT2
SZ_DE	CM_NO_SVA_DE_FDR_0.05	6320	CLEC11A
SZ_DE	CM_NO_SVA_DE_FDR_0.05	3671	ISLR
SZ_DE	CM_NO_SVA_DE_FDR_0.05	474344	GIMAP6
SZ_DE	CM_NO_SVA_DE_FDR_0.05	284111	SLC13A5
SZ_DE	CM_NO_SVA_DE_FDR_0.05	116349	C5orf55
SZ_DE	CM_NO_SVA_DE_FDR_0.05	5524	PPP2R4
SZ_DE	CM_NO_SVA_DE_FDR_0.05	387775	SLC22A10
SZ_DE	CM_NO_SVA_DE_FDR_0.05	2987	GUK1
SZ_DE	CM_NO_SVA_DE_FDR_0.05	84628	NTNG2
SZ_DE	CM_NO_SVA_DE_FDR_0.05	89932	PAPLN
SZ_DE	CM_NO_SVA_DE_FDR_0.05	54461	FBXW5
SZ_DE	CM_NO_SVA_DE_FDR_0.05	113791	PIK3IP1
SZ_DE	CM_NO_SVA_DE_FDR_0.05	631	BFSP1
SZ_DE	CM_NO_SVA_DE_FDR_0.05	2134	EXTL1
SZ_DE	CM_NO_SVA_DE_FDR_0.05	57795	BRINP2
SZ_DE	CM_NO_SVA_DE_FDR_0.05	55652	SLC48A1
SZ_DE	CM_NO_SVA_DE_FDR_0.05	253982	ASPHD1
SZ_DE	CM_NO_SVA_DE_FDR_0.05	129807	NEU4
SZ_DE	CM_NO_SVA_DE_FDR_0.05	25906	ANAPC15
SZ_DE	CM_NO_SVA_DE_FDR_0.05	2954	GSTZ1
SZ_DE	CM_NO_SVA_DE_FDR_0.05	348751	FTCDNL1
SZ_DE	CM_NO_SVA_DE_FDR_0.05	80737	VWA7
SZ_DE	CM_NO_SVA_DE_FDR_0.05	22845	DOLK
SZ_DE	CM_NO_SVA_DE_FDR_0.05	1307	COL16A1
SZ_DE	CM_NO_SVA_DE_FDR_0.05	576	BAI2
SZ_DE	CM_NO_SVA_DE_FDR_0.05	57128	LYRM4
SZ_DE	CM_NO_SVA_DE_FDR_0.05	128240	APOA1BP
SZ_DE	CM_NO_SVA_DE_FDR_0.05	6231	RPS26
SZ_DE	CM_NO_SVA_DE_FDR_0.05	55808	ST6GALNAC1
SZ_DE	CM_NO_SVA_DE_FDR_0.05	8861	LDB1
NPD_Sets	ASD-candidates	55624	POMGNT1
NPD_Sets	ASD-candidates	6121	RPE65
NPD_Sets	ASD-candidates	1806	DPYD
NPD_Sets	ASD-candidates	9378	NRXN1
NPD_Sets	ASD-candidates	4867	NPHP1
NPD_Sets	ASD-candidates	55777	MBD5
NPD_Sets	ASD-candidates	6323	SCN1A
NPD_Sets	ASD-candidates	23314	SATB2
NPD_Sets	ASD-candidates	686	BTD
NPD_Sets	ASD-candidates	27086	FOXP1
NPD_Sets	ASD-candidates	8492	PRSS12
NPD_Sets	ASD-candidates	25836	NIPBL
NPD_Sets	ASD-candidates	4208	MEF2C
NPD_Sets	ASD-candidates	501	ALDH7A1
NPD_Sets	ASD-candidates	64324	NSD1

NPD_Sets	ASD-candidates	7915	ALDH5A1
NPD_Sets	ASD-candidates	8831	SYNGAP1
NPD_Sets	ASD-candidates	54806	AHI1
NPD_Sets	ASD-candidates	3198	HOXA1
NPD_Sets	ASD-candidates	673	BRAF
NPD_Sets	ASD-candidates	26047	CNTNAP2
NPD_Sets	ASD-candidates	138050	HGSNAT
NPD_Sets	ASD-candidates	55636	CHD7
NPD_Sets	ASD-candidates	157680	VPS13B
NPD_Sets	ASD-candidates	6812	STXBP1
NPD_Sets	ASD-candidates	10585	POMT1
NPD_Sets	ASD-candidates	7248	TSC1
NPD_Sets	ASD-candidates	79813	EHMT1
NPD_Sets	ASD-candidates	5728	PTEN
NPD_Sets	ASD-candidates	2263	FGFR2
NPD_Sets	ASD-candidates	3265	HRAS
NPD_Sets	ASD-candidates	3481	IGF2
NPD_Sets	ASD-candidates	3767	KCNJ11
NPD_Sets	ASD-candidates	22941	SHANK2
NPD_Sets	ASD-candidates	1717	DHCR7
NPD_Sets	ASD-candidates	2348	FOLR1
NPD_Sets	ASD-candidates	220296	HEPACAM
NPD_Sets	ASD-candidates	775	CACNA1C
NPD_Sets	ASD-candidates	2904	GRIN2B
NPD_Sets	ASD-candidates	3845	KRAS
NPD_Sets	ASD-candidates	2799	GNS
NPD_Sets	ASD-candidates	80184	CEP290
NPD_Sets	ASD-candidates	5053	PAH
NPD_Sets	ASD-candidates	5781	PTPN11
NPD_Sets	ASD-candidates	2290	FOXG1
NPD_Sets	ASD-candidates	79944	L2HGDH
NPD_Sets	ASD-candidates	7337	UBE3A
NPD_Sets	ASD-candidates	2628	GATM
NPD_Sets	ASD-candidates	5604	MAP2K1
NPD_Sets	ASD-candidates	7249	TSC2
NPD_Sets	ASD-candidates	1387	CREBBP
NPD_Sets	ASD-candidates	23322	RPGRIP1L
NPD_Sets	ASD-candidates	7531	YWHAE
NPD_Sets	ASD-candidates	5048	PAFAH1B1
NPD_Sets	ASD-candidates	3000	GUCY2D
NPD_Sets	ASD-candidates	10743	RAI1
NPD_Sets	ASD-candidates	84282	RNF135
NPD_Sets	ASD-candidates	4763	NF1
NPD_Sets	ASD-candidates	4669	NAGLU
NPD_Sets	ASD-candidates	6448	SGSH
NPD_Sets	ASD-candidates	2593	GAMT
NPD_Sets	ASD-candidates	4784	NFIX
NPD_Sets	ASD-candidates	1760	DMPK
NPD_Sets	ASD-candidates	8195	MKKS
NPD_Sets	ASD-candidates	6899	TBX1
NPD_Sets	ASD-candidates	158	ADSL
NPD_Sets	ASD-candidates	85358	SHANK3
NPD_Sets	ASD-candidates	57502	NLGN4X
NPD_Sets	ASD-candidates	8905	AP1S2
NPD_Sets	ASD-candidates	4810	NHS
NPD_Sets	ASD-candidates	6792	CDKL5
NPD_Sets	ASD-candidates	139411	PTCHD1
NPD_Sets	ASD-candidates	170302	ARX

NPD_Sets	ASD-candidates	11141	IL1RAPL1
NPD_Sets	ASD-candidates	1756	DMD
NPD_Sets	ASD-candidates	5009	OTC
NPD_Sets	ASD-candidates	8573	CASK
NPD_Sets	ASD-candidates	4693	NDP
NPD_Sets	ASD-candidates	641339	ZNF674
NPD_Sets	ASD-candidates	6853	SYN1
NPD_Sets	ASD-candidates	347344	ZNF81
NPD_Sets	ASD-candidates	24140	FTSJ1
NPD_Sets	ASD-candidates	10084	PQBP1
NPD_Sets	ASD-candidates	778	CACNA1F
NPD_Sets	ASD-candidates	23096	IQSEC2
NPD_Sets	ASD-candidates	8243	SMC1A
NPD_Sets	ASD-candidates	23133	PHF8
NPD_Sets	ASD-candidates	2245	FGD1
NPD_Sets	ASD-candidates	4983	OPHN1
NPD_Sets	ASD-candidates	9968	MED12
NPD_Sets	ASD-candidates	54413	NLGN3
NPD_Sets	ASD-candidates	340533	KIAA2022
NPD_Sets	ASD-candidates	546	ATRX
NPD_Sets	ASD-candidates	57526	PCDH19
NPD_Sets	ASD-candidates	2182	ACSL4
NPD_Sets	ASD-candidates	1641	DCX
NPD_Sets	ASD-candidates	186	AGTR2
NPD_Sets	ASD-candidates	65109	UPF3B
NPD_Sets	ASD-candidates	3920	LAMP2
NPD_Sets	ASD-candidates	2892	GRIA3
NPD_Sets	ASD-candidates	4952	OCRL
NPD_Sets	ASD-candidates	84295	PHF6
NPD_Sets	ASD-candidates	10479	SLC9A6
NPD_Sets	ASD-candidates	9459	ARHGEF6
NPD_Sets	ASD-candidates	2332	FMR1
NPD_Sets	ASD-candidates	2334	AFF2
NPD_Sets	ASD-candidates	6535	SLC6A8
NPD_Sets	ASD-candidates	3897	L1CAM
NPD_Sets	ASD-candidates	4204	MECP2
NPD_Sets	ASD-candidates	116442	RAB39B
NPD_Sets	ASD-denovo-lof	155038	GIMAP8
NPD_Sets	ASD-denovo-lof	667	DST
NPD_Sets	ASD-denovo-lof	5118	PCOLCE
NPD_Sets	ASD-denovo-lof	93100	NAPRT1
NPD_Sets	ASD-denovo-lof	401667	OR51A2
NPD_Sets	ASD-denovo-lof	1767	DNAH5
NPD_Sets	ASD-denovo-lof	9958	USP15
NPD_Sets	ASD-denovo-lof	9056	SLC7A7
NPD_Sets	ASD-denovo-lof	55015	PRPF39
NPD_Sets	ASD-denovo-lof	5649	RELN
NPD_Sets	ASD-denovo-lof	84919	PPP1R15B
NPD_Sets	ASD-denovo-lof	6738	TROVE2
NPD_Sets	ASD-denovo-lof	22999	RIMS1
NPD_Sets	ASD-denovo-lof	54737	MPHOSPH8
NPD_Sets	ASD-denovo-lof	6326	SCN2A
NPD_Sets	ASD-denovo-lof	285175	UNC80
NPD_Sets	ASD-denovo-lof	8029	CUBN
NPD_Sets	ASD-denovo-lof	374786	EFCAB5
NPD_Sets	ASD-denovo-lof	221981	THSD7A
NPD_Sets	ASD-denovo-lof	10788	IQGAP2
NPD_Sets	ASD-denovo-lof	23394	ADNP

NPD_Sets	ASD-denovo-lof	343413	FCRL6
NPD_Sets	ASD-denovo-lof	51107	APH1A
NPD_Sets	ASD-denovo-lof	221178	SPATA13
NPD_Sets	ASD-denovo-lof	55773	TBC1D23
NPD_Sets	ASD-denovo-lof	4036	LRP2
NPD_Sets	ASD-denovo-lof	51111	SUV420H1
NPD_Sets	ASD-denovo-lof	51127	TRIM17
NPD_Sets	ASD-denovo-lof	9320	TRIP12
NPD_Sets	ASD-denovo-lof	8452	CUL3
NPD_Sets	ASD-denovo-lof	23503	ZFYVE26
NPD_Sets	ASD-denovo-lof	374378	GALNT18
NPD_Sets	ASD-denovo-lof	80332	ADAM33
NPD_Sets	ASD-denovo-lof	2109	ETFB
NPD_Sets	ASD-denovo-lof	100534611	TM4SF19-TCTEX1D2
NPD_Sets	ASD-denovo-lof	481	ATP1B1
NPD_Sets	ASD-denovo-lof	728635	DHRS4L1
NPD_Sets	ASD-denovo-lof	23135	KDM6B
NPD_Sets	ASD-denovo-lof	28514	DLL1
NPD_Sets	ASD-denovo-lof	55799	CACNA2D3
NPD_Sets	ASD-denovo-lof	9378	NRXN1
NPD_Sets	ASD-denovo-lof	5253	PHF2
NPD_Sets	ASD-denovo-lof	7812	CSDE1
NPD_Sets	ASD-denovo-lof	5862	RAB2A
NPD_Sets	ASD-denovo-lof	57556	SEMA6A
NPD_Sets	ASD-denovo-lof	157769	FAM91A1
NPD_Sets	ASD-denovo-lof	83473	KATNAL2
NPD_Sets	ASD-denovo-lof	8803	SUCLA2
NPD_Sets	ASD-denovo-lof	8493	PPM1D
NPD_Sets	ASD-denovo-lof	166752	FREM3
NPD_Sets	ASD-denovo-lof	9891	NUAK1
NPD_Sets	ASD-denovo-lof	5079	PAX5
NPD_Sets	ASD-denovo-lof	10716	TBR1
NPD_Sets	ASD-denovo-lof	29850	TRPM5
NPD_Sets	ASD-denovo-lof	56136	PCDHA13
NPD_Sets	ASD-denovo-lof	56624	ASAH2
NPD_Sets	ASD-denovo-lof	23036	ZNF292
NPD_Sets	ASD-denovo-lof	6929	TCF3
NPD_Sets	ASD-denovo-lof	10787	NCKAP1
NPD_Sets	ASD-denovo-lof	23389	MED13L
NPD_Sets	ASD-denovo-lof	84622	ZNF594
NPD_Sets	ASD-denovo-lof	6342	SCP2
NPD_Sets	ASD-denovo-lof	65083	NOL6
NPD_Sets	ASD-denovo-lof	32	ACACB
NPD_Sets	ASD-denovo-lof	254048	UBN2
NPD_Sets	ASD-denovo-lof	9778	KIAA0232
NPD_Sets	ASD-denovo-lof	57680	CHD8
NPD_Sets	ASD-denovo-lof	83552	MFRP
NPD_Sets	ASD-denovo-lof	55777	MBD5
NPD_Sets	ASD-denovo-lof	23126	POGZ
NPD_Sets	ASD-denovo-lof	83992	CTTNBP2
NPD_Sets	ASD-denovo-lof	6840	SVIL
NPD_Sets	ASD-denovo-lof	9703	KIAA0100
NPD_Sets	ASD-denovo-lof	642636	RAD21L1
NPD_Sets	ASD-denovo-lof	3790	KCNS3
NPD_Sets	ASD-denovo-lof	5442	POLRMT
NPD_Sets	ASD-denovo-lof	696	BTN1A1
NPD_Sets	ASD-denovo-lof	23283	CSTF2T
NPD_Sets	ASD-denovo-lof	55739	CARKD

NPD_Sets	ASD-denovo-lof	23181	DIP2A
NPD_Sets	ASD-denovo-lof	4306	NR3C2
NPD_Sets	ASD-denovo-lof	7468	WHSC1
NPD_Sets	ASD-denovo-lof	27332	ZNF638
NPD_Sets	ASD-denovo-lof	22982	DIP2C
NPD_Sets	ASD-denovo-lof	57758	SCUBE2
NPD_Sets	ASD-denovo-lof	116211	TM4SF19
NPD_Sets	ASD-denovo-lof	84570	COL25A1
NPD_Sets	ASD-denovo-lof	10402	ST3GAL6
NPD_Sets	ASD-denovo-lof	7415	VCP
NPD_Sets	ASD-denovo-lof	51439	FAM8A1
NPD_Sets	ASD-denovo-lof	114783	LMTK3
NPD_Sets	ASD-denovo-lof	26046	LTN1
NPD_Sets	ASD-denovo-lof	287	ANK2
NPD_Sets	ASD-denovo-lof	10771	ZMYND11
NPD_Sets	ASD-denovo-lof	3678	ITGA5
NPD_Sets	ASD-denovo-lof	54870	QRICH1
NPD_Sets	ASD-denovo-lof	4774	NFIA
NPD_Sets	ASD-denovo-lof	795	S100G
NPD_Sets	ASD-denovo-lof	6197	RPS6KA3
NPD_Sets	ASD-denovo-lof	51629	SLC25A39
NPD_Sets	ASD-denovo-lof	1842	ECM2
NPD_Sets	ASD-denovo-lof	9578	CDC42BPB
NPD_Sets	ASD-denovo-lof	2312	FLG
NPD_Sets	ASD-denovo-lof	57205	ATP10D
NPD_Sets	ASD-denovo-lof	54014	BRWD1
NPD_Sets	ASD-denovo-lof	22941	SHANK2
NPD_Sets	ASD-denovo-lof	152006	RNF38
NPD_Sets	ASD-denovo-lof	2048	EPHB2
NPD_Sets	ASD-denovo-lof	23001	WDFY3
NPD_Sets	ASD-denovo-lof	54545	MTMR12
NPD_Sets	ASD-denovo-lof	6683	SPAST
NPD_Sets	ASD-denovo-lof	203859	ANO5
NPD_Sets	ASD-denovo-lof	8535	CBX4
NPD_Sets	ASD-denovo-lof	1859	DYRK1A
NPD_Sets	ASD-denovo-lof	6601	SMARCC2
NPD_Sets	ASD-denovo-lof	26262	TSPAN17
NPD_Sets	ASD-denovo-lof	100528021	ST20-MTHFS
NPD_Sets	ASD-denovo-lof	6694	SPP2
NPD_Sets	ASD-denovo-lof	10588	MTHFS
NPD_Sets	ASD-denovo-lof	2904	GRIN2B
NPD_Sets	ASD-denovo-lof	4849	CNOT3
NPD_Sets	ASD-denovo-lof	53335	BCL11A
NPD_Sets	ASD-denovo-lof	29072	SETD2
NPD_Sets	ASD-denovo-lof	5364	PLXNB1
NPD_Sets	ASD-denovo-nonsyn	6569	SLC34A1
NPD_Sets	ASD-denovo-nonsyn	4353	MPO
NPD_Sets	ASD-denovo-nonsyn	6098	ROS1
NPD_Sets	ASD-denovo-nonsyn	282890	ZNF311
NPD_Sets	ASD-denovo-nonsyn	6340	SCNN1G
NPD_Sets	ASD-denovo-nonsyn	8658	TNKS
NPD_Sets	ASD-denovo-nonsyn	8493	PPM1D
NPD_Sets	ASD-denovo-nonsyn	8567	MADD
NPD_Sets	ASD-denovo-nonsyn	64854	USP46
NPD_Sets	ASD-denovo-nonsyn	5587	PRKD1
NPD_Sets	ASD-denovo-nonsyn	84261	FBXW9
NPD_Sets	ASD-denovo-nonsyn	56963	RGMA
NPD_Sets	ASD-denovo-nonsyn	192286	HIGD2A

NPD_Sets	ASD-denovo-nonsyn	4774	NFIA
NPD_Sets	ASD-denovo-nonsyn	7399	USH2A
NPD_Sets	ASD-denovo-nonsyn	23363	OBSL1
NPD_Sets	ASD-denovo-nonsyn	1956	EGFR
NPD_Sets	ASD-denovo-nonsyn	10024	TROAP
NPD_Sets	ASD-denovo-nonsyn	50831	TAS2R3
NPD_Sets	ASD-denovo-nonsyn	9124	PDLIM1
NPD_Sets	ASD-denovo-nonsyn	57758	SCUBE2
NPD_Sets	ASD-denovo-nonsyn	94025	MUC16
NPD_Sets	ASD-denovo-nonsyn	140453	MUC17
NPD_Sets	ASD-denovo-nonsyn	3012	HIST1H2AE
NPD_Sets	ASD-denovo-nonsyn	26013	L3MBTL1
NPD_Sets	ASD-denovo-nonsyn	23189	KANK1
NPD_Sets	ASD-denovo-nonsyn	80312	TET1
NPD_Sets	ASD-denovo-nonsyn	696	BTN1A1
NPD_Sets	ASD-denovo-nonsyn	6389	SDHA
NPD_Sets	ASD-denovo-nonsyn	27006	FGF22
NPD_Sets	ASD-denovo-nonsyn	1788	DNMT3A
NPD_Sets	ASD-denovo-nonsyn	3274	HRH2
NPD_Sets	ASD-denovo-nonsyn	63891	RNF123
NPD_Sets	ASD-denovo-nonsyn	727897	MUC5B
NPD_Sets	ASD-denovo-nonsyn	5296	PIK3R2
NPD_Sets	ASD-denovo-nonsyn	6144	RPL21
NPD_Sets	ASD-denovo-nonsyn	374977	MROH7
NPD_Sets	ASD-denovo-nonsyn	481	ATP1B1
NPD_Sets	ASD-denovo-nonsyn	375611	SLC26A5
NPD_Sets	ASD-denovo-nonsyn	9858	PPP1R26
NPD_Sets	ASD-denovo-nonsyn	776	CACNA1D
NPD_Sets	ASD-denovo-nonsyn	57535	KIAA1324
NPD_Sets	ASD-denovo-nonsyn	777	CACNA1E
NPD_Sets	ASD-denovo-nonsyn	10771	ZMYND11
NPD_Sets	ASD-denovo-nonsyn	374739	TEPP
NPD_Sets	ASD-denovo-nonsyn	5990	RFX2
NPD_Sets	ASD-denovo-nonsyn	55160	ARHGEF10L
NPD_Sets	ASD-denovo-nonsyn	6529	SLC6A1
NPD_Sets	ASD-denovo-nonsyn	479	ATP12A
NPD_Sets	ASD-denovo-nonsyn	728635	DHRS4L1
NPD_Sets	ASD-denovo-nonsyn	6531	SLC6A3
NPD_Sets	ASD-denovo-nonsyn	60626	RIC8A
NPD_Sets	ASD-denovo-nonsyn	23774	BRD1
NPD_Sets	ASD-denovo-nonsyn	731220	RFX8
NPD_Sets	ASD-denovo-nonsyn	23476	BRD4
NPD_Sets	ASD-denovo-nonsyn	779	CACNA1S
NPD_Sets	ASD-denovo-nonsyn	401667	OR51A2
NPD_Sets	ASD-denovo-nonsyn	26873	OPLAH
NPD_Sets	ASD-denovo-nonsyn	79829	NAA40
NPD_Sets	ASD-denovo-nonsyn	2873	GPS1
NPD_Sets	ASD-denovo-nonsyn	149628	PYHIN1
NPD_Sets	ASD-denovo-nonsyn	200162	SPAG17
NPD_Sets	ASD-denovo-nonsyn	4666	NACA
NPD_Sets	ASD-denovo-nonsyn	3308	HSPA4
NPD_Sets	ASD-denovo-nonsyn	8295	TRRAP
NPD_Sets	ASD-denovo-nonsyn	23339	VPS39
NPD_Sets	ASD-denovo-nonsyn	3633	INPP5B
NPD_Sets	ASD-denovo-nonsyn	4486	MST1R
NPD_Sets	ASD-denovo-nonsyn	2262	GPC5
NPD_Sets	ASD-denovo-nonsyn	128822	CST9
NPD_Sets	ASD-denovo-nonsyn	23294	ANKS1A

NPD_Sets	ASD-denovo-nonsyn	155038	GIMAP8
NPD_Sets	ASD-denovo-nonsyn	114791	TUBGCP5
NPD_Sets	ASD-denovo-nonsyn	23181	DIP2A
NPD_Sets	ASD-denovo-nonsyn	4154	MBNL1
NPD_Sets	ASD-denovo-nonsyn	22982	DIP2C
NPD_Sets	ASD-denovo-nonsyn	123096	SLC25A29
NPD_Sets	ASD-denovo-nonsyn	1475	CSTA
NPD_Sets	ASD-denovo-nonsyn	23135	KDM6B
NPD_Sets	ASD-denovo-nonsyn	4648	MYO7B
NPD_Sets	ASD-denovo-nonsyn	23503	ZFYVE26
NPD_Sets	ASD-denovo-nonsyn	7982	ST7
NPD_Sets	ASD-denovo-nonsyn	121457	IKBIP
NPD_Sets	ASD-denovo-nonsyn	4208	MEF2C
NPD_Sets	ASD-denovo-nonsyn	121340	SP7
NPD_Sets	ASD-denovo-nonsyn	25957	PNISR
NPD_Sets	ASD-denovo-nonsyn	9777	TM9SF4
NPD_Sets	ASD-denovo-nonsyn	2015	EMR1
NPD_Sets	ASD-denovo-nonsyn	91752	ZNF804A
NPD_Sets	ASD-denovo-nonsyn	219578	ZNF804B
NPD_Sets	ASD-denovo-nonsyn	2048	EPHB2
NPD_Sets	ASD-denovo-nonsyn	25843	MOB4
NPD_Sets	ASD-denovo-nonsyn	51168	MYO15A
NPD_Sets	ASD-denovo-nonsyn	4288	MKI67
NPD_Sets	ASD-denovo-nonsyn	81	ACTN4
NPD_Sets	ASD-denovo-nonsyn	55079	FEZF2
NPD_Sets	ASD-denovo-nonsyn	2580	GAK
NPD_Sets	ASD-denovo-nonsyn	100885850	PTGES3L-AARSD1
NPD_Sets	ASD-denovo-nonsyn	8532	CPZ
NPD_Sets	ASD-denovo-nonsyn	54439	RBM27
NPD_Sets	ASD-denovo-nonsyn	55262	C7orf43
NPD_Sets	ASD-denovo-nonsyn	5519	PPP2R1B
NPD_Sets	ASD-denovo-nonsyn	253782	CERS6
NPD_Sets	ASD-denovo-nonsyn	6305	SBF1
NPD_Sets	ASD-denovo-nonsyn	81846	SBF2
NPD_Sets	ASD-denovo-nonsyn	10514	MYBBP1A
NPD_Sets	ASD-denovo-nonsyn	26154	ABCA12
NPD_Sets	ASD-denovo-nonsyn	154664	ABCA13
NPD_Sets	ASD-denovo-nonsyn	130733	TMEM178A
NPD_Sets	ASD-denovo-nonsyn	26262	TSPAN17
NPD_Sets	ASD-denovo-nonsyn	60492	CCDC90B
NPD_Sets	ASD-denovo-nonsyn	9901	SRGAP3
NPD_Sets	ASD-denovo-nonsyn	114815	SORCS1
NPD_Sets	ASD-denovo-nonsyn	167410	LIX1
NPD_Sets	ASD-denovo-nonsyn	6323	SCN1A
NPD_Sets	ASD-denovo-nonsyn	90121	TSR2
NPD_Sets	ASD-denovo-nonsyn	8220	DGCR14
NPD_Sets	ASD-denovo-nonsyn	5978	REST
NPD_Sets	ASD-denovo-nonsyn	6929	TCF3
NPD_Sets	ASD-denovo-nonsyn	4763	NF1
NPD_Sets	ASD-denovo-nonsyn	27148	STK36
NPD_Sets	ASD-denovo-nonsyn	5430	POLR2A
NPD_Sets	ASD-denovo-nonsyn	8607	RUVBL1
NPD_Sets	ASD-denovo-nonsyn	23474	ETHE1
NPD_Sets	ASD-denovo-nonsyn	81488	POLR2M
NPD_Sets	ASD-denovo-nonsyn	30846	EHD2
NPD_Sets	ASD-denovo-nonsyn	2312	FLG
NPD_Sets	ASD-denovo-nonsyn	83439	TCF7L1
NPD_Sets	ASD-denovo-nonsyn	84498	FAM120B

NPD_Sets	ASD-denovo-nonsyn	5604	MAP2K1
NPD_Sets	ASD-denovo-nonsyn	5213	PFKM
NPD_Sets	ASD-denovo-nonsyn	3728	JUP
NPD_Sets	ASD-denovo-nonsyn	80310	PDGFD
NPD_Sets	ASD-denovo-nonsyn	23168	RTF1
NPD_Sets	ASD-denovo-nonsyn	124274	GPR139
NPD_Sets	ASD-denovo-nonsyn	23223	RRP12
NPD_Sets	ASD-denovo-nonsyn	54853	WDR55
NPD_Sets	ASD-denovo-nonsyn	8943	AP3D1
NPD_Sets	ASD-denovo-nonsyn	55015	PRPF39
NPD_Sets	ASD-denovo-nonsyn	345	APOC3
NPD_Sets	ASD-denovo-nonsyn	9536	PTGES
NPD_Sets	ASD-denovo-nonsyn	25831	HECTD1
NPD_Sets	ASD-denovo-nonsyn	51127	TRIM17
NPD_Sets	ASD-denovo-nonsyn	8940	TOP3B
NPD_Sets	ASD-denovo-nonsyn	57448	BIRC6
NPD_Sets	ASD-denovo-nonsyn	10330	CNPY2
NPD_Sets	ASD-denovo-nonsyn	51163	DBR1
NPD_Sets	ASD-denovo-nonsyn	5187	PER1
NPD_Sets	ASD-denovo-nonsyn	3185	HNRNPF
NPD_Sets	ASD-denovo-nonsyn	26046	LTN1
NPD_Sets	ASD-denovo-nonsyn	54682	MANSC1
NPD_Sets	ASD-denovo-nonsyn	94239	H2AFV
NPD_Sets	ASD-denovo-nonsyn	26115	TANC2
NPD_Sets	ASD-denovo-nonsyn	81930	KIF18A
NPD_Sets	ASD-denovo-nonsyn	9870	AREL1
NPD_Sets	ASD-denovo-nonsyn	4087	SMAD2
NPD_Sets	ASD-denovo-nonsyn	301	ANXA1
NPD_Sets	ASD-denovo-nonsyn	8749	ADAM18
NPD_Sets	ASD-denovo-nonsyn	79730	NSUN7
NPD_Sets	ASD-denovo-nonsyn	7007	TECTA
NPD_Sets	ASD-denovo-nonsyn	4602	MYB
NPD_Sets	ASD-denovo-nonsyn	84059	GPR98
NPD_Sets	ASD-denovo-nonsyn	2778	GNAS
NPD_Sets	ASD-denovo-nonsyn	4317	MMP8
NPD_Sets	ASD-denovo-nonsyn	100529241	HSPE1-MOB4
NPD_Sets	ASD-denovo-nonsyn	53335	BCL11A
NPD_Sets	ASD-denovo-nonsyn	148811	PM20D1
NPD_Sets	ASD-denovo-nonsyn	7204	TRIO
NPD_Sets	ASD-denovo-nonsyn	5047	PAEP
NPD_Sets	ASD-denovo-nonsyn	342132	ZNF774
NPD_Sets	ASD-denovo-nonsyn	57584	ARHGAP21
NPD_Sets	ASD-denovo-nonsyn	84063	KIRREL2
NPD_Sets	ASD-denovo-nonsyn	64924	SLC30A5
NPD_Sets	ASD-denovo-nonsyn	167465	ZNF366
NPD_Sets	ASD-denovo-nonsyn	84623	KIRREL3
NPD_Sets	ASD-denovo-nonsyn	2	A2M
NPD_Sets	ASD-denovo-nonsyn	5862	RAB2A
NPD_Sets	ASD-denovo-nonsyn	26509	MYOF
NPD_Sets	ASD-denovo-nonsyn	9958	USP15
NPD_Sets	ASD-denovo-nonsyn	54934	KANSL2
NPD_Sets	ASD-denovo-nonsyn	83473	KATNAL2
NPD_Sets	ASD-denovo-nonsyn	23255	SOGA2
NPD_Sets	ASD-denovo-nonsyn	57828	CATSPERG
NPD_Sets	ASD-denovo-nonsyn	387104	SOGA3
NPD_Sets	ASD-denovo-nonsyn	10553	HTATIP2
NPD_Sets	ASD-denovo-nonsyn	26233	FBXL6
NPD_Sets	ASD-denovo-nonsyn	6694	SPP2

NPD_Sets	ASD-denovo-nonsyn	7059	THBS3
NPD_Sets	ASD-denovo-nonsyn	9320	TRIP12
NPD_Sets	ASD-denovo-nonsyn	83959	SLC4A11
NPD_Sets	ASD-denovo-nonsyn	1797	DOM3Z
NPD_Sets	ASD-denovo-nonsyn	80344	DCAF11
NPD_Sets	ASD-denovo-nonsyn	51200	CPA4
NPD_Sets	ASD-denovo-nonsyn	10302	SNAPC5
NPD_Sets	ASD-denovo-nonsyn	55746	NUP133
NPD_Sets	ASD-denovo-nonsyn	10788	IQGAP2
NPD_Sets	ASD-denovo-nonsyn	23019	CNOT1
NPD_Sets	ASD-denovo-nonsyn	128239	IQGAP3
NPD_Sets	ASD-denovo-nonsyn	4849	CNOT3
NPD_Sets	ASD-denovo-nonsyn	4850	CNOT4
NPD_Sets	ASD-denovo-nonsyn	1258	CNGB1
NPD_Sets	ASD-denovo-nonsyn	57472	CNOT6
NPD_Sets	ASD-denovo-nonsyn	5253	PHF2
NPD_Sets	ASD-denovo-nonsyn	23469	PHF3
NPD_Sets	ASD-denovo-nonsyn	147798	TMC4
NPD_Sets	ASD-denovo-nonsyn	9778	KIAA0232
NPD_Sets	ASD-denovo-nonsyn	11224	RPL35
NPD_Sets	ASD-denovo-nonsyn	84952	CGNL1
NPD_Sets	ASD-denovo-nonsyn	6491	STIL
NPD_Sets	ASD-denovo-nonsyn	4193	MDM2
NPD_Sets	ASD-denovo-nonsyn	3382	ICA1
NPD_Sets	ASD-denovo-nonsyn	10130	PDIA6
NPD_Sets	ASD-denovo-nonsyn	51019	CCDC53
NPD_Sets	ASD-denovo-nonsyn	51678	MPP6
NPD_Sets	ASD-denovo-nonsyn	51439	FAM8A1
NPD_Sets	ASD-denovo-nonsyn	64770	CCDC14
NPD_Sets	ASD-denovo-nonsyn	114571	SLC22A9
NPD_Sets	ASD-denovo-nonsyn	22871	NLGN1
NPD_Sets	ASD-denovo-nonsyn	79718	TBL1XR1
NPD_Sets	ASD-denovo-nonsyn	1787	TRDMT1
NPD_Sets	ASD-denovo-nonsyn	23613	ZMYND8
NPD_Sets	ASD-denovo-nonsyn	343099	CCDC18
NPD_Sets	ASD-denovo-nonsyn	23036	ZNF292
NPD_Sets	ASD-denovo-nonsyn	11132	CAPN10
NPD_Sets	ASD-denovo-nonsyn	5728	PTEN
NPD_Sets	ASD-denovo-nonsyn	8562	DENR
NPD_Sets	ASD-denovo-nonsyn	7760	ZNF213
NPD_Sets	ASD-denovo-nonsyn	7762	ZNF215
NPD_Sets	ASD-denovo-nonsyn	128368	OR10Z1
NPD_Sets	ASD-denovo-nonsyn	3996	LLGL1
NPD_Sets	ASD-denovo-nonsyn	10343	PKDREJ
NPD_Sets	ASD-denovo-nonsyn	317	APAF1
NPD_Sets	ASD-denovo-nonsyn	32	ACACB
NPD_Sets	ASD-denovo-nonsyn	64409	WBSCR17
NPD_Sets	ASD-denovo-nonsyn	643338	C15orf62
NPD_Sets	ASD-denovo-nonsyn	93100	NAPRT1
NPD_Sets	ASD-denovo-nonsyn	90407	TMEM41A
NPD_Sets	ASD-denovo-nonsyn	6342	SCP2
NPD_Sets	ASD-denovo-nonsyn	196385	DNAH10
NPD_Sets	ASD-denovo-nonsyn	3316	HSPB2
NPD_Sets	ASD-denovo-nonsyn	8518	IKBKAP
NPD_Sets	ASD-denovo-nonsyn	8701	DNAH11
NPD_Sets	ASD-denovo-nonsyn	9578	CDC42BPB
NPD_Sets	ASD-denovo-nonsyn	114783	LMTK3
NPD_Sets	ASD-denovo-nonsyn	8632	DNAH17

NPD_Sets	ASD-denovo-nonsyn	3913	LAMB2
NPD_Sets	ASD-denovo-nonsyn	3914	LAMB3
NPD_Sets	ASD-denovo-nonsyn	138009	DCAF4L2
NPD_Sets	ASD-denovo-nonsyn	344558	SH3RF3
NPD_Sets	ASD-denovo-nonsyn	27183	VPS4A
NPD_Sets	ASD-denovo-nonsyn	8777	MPDZ
NPD_Sets	ASD-denovo-nonsyn	26003	GORASP2
NPD_Sets	ASD-denovo-nonsyn	51629	SLC25A39
NPD_Sets	ASD-denovo-nonsyn	4035	LRP1
NPD_Sets	ASD-denovo-nonsyn	4036	LRP2
NPD_Sets	ASD-denovo-nonsyn	27010	TPK1
NPD_Sets	ASD-denovo-nonsyn	5118	PCOLCE
NPD_Sets	ASD-denovo-nonsyn	10072	DPP3
NPD_Sets	ASD-denovo-nonsyn	8216	LZTR1
NPD_Sets	ASD-denovo-nonsyn	57664	PLEKHA4
NPD_Sets	ASD-denovo-nonsyn	4041	LRP5
NPD_Sets	ASD-denovo-nonsyn	51284	TLR7
NPD_Sets	ASD-denovo-nonsyn	157769	FAM91A1
NPD_Sets	ASD-denovo-nonsyn	114825	PWWP2A
NPD_Sets	ASD-denovo-nonsyn	83900	KRTAP9-3
NPD_Sets	ASD-denovo-nonsyn	2798	GNRHR
NPD_Sets	ASD-denovo-nonsyn	675	BRCA2
NPD_Sets	ASD-denovo-nonsyn	51380	CSAD
NPD_Sets	ASD-denovo-nonsyn	9734	HDAC9
NPD_Sets	ASD-denovo-nonsyn	84725	PLEKHA8
NPD_Sets	ASD-denovo-nonsyn	23345	SYNE1
NPD_Sets	ASD-denovo-nonsyn	6738	TROVE2
NPD_Sets	ASD-denovo-nonsyn	8925	HERC1
NPD_Sets	ASD-denovo-nonsyn	79782	LRRC31
NPD_Sets	ASD-denovo-nonsyn	79671	NLRX1
NPD_Sets	ASD-denovo-nonsyn	4439	MSH5
NPD_Sets	ASD-denovo-nonsyn	374786	EFCAB5
NPD_Sets	ASD-denovo-nonsyn	57519	STARD9
NPD_Sets	ASD-denovo-nonsyn	51234	EMC4
NPD_Sets	ASD-denovo-nonsyn	790	CAD
NPD_Sets	ASD-denovo-nonsyn	64800	EFCAB6
NPD_Sets	ASD-denovo-nonsyn	2956	MSH6
NPD_Sets	ASD-denovo-nonsyn	55536	CDCA7L
NPD_Sets	ASD-denovo-nonsyn	55773	TBC1D23
NPD_Sets	ASD-denovo-nonsyn	400823	FAM177B
NPD_Sets	ASD-denovo-nonsyn	54212	SNTG1
NPD_Sets	ASD-denovo-nonsyn	171389	NLRP6
NPD_Sets	ASD-denovo-nonsyn	254272	TBC1D28
NPD_Sets	ASD-denovo-nonsyn	221981	THSD7A
NPD_Sets	ASD-denovo-nonsyn	6523	SLC5A1
NPD_Sets	ASD-denovo-nonsyn	100885848	PTGES3L
NPD_Sets	ASD-denovo-nonsyn	27303	RBMS3
NPD_Sets	ASD-denovo-nonsyn	8876	VNN1
NPD_Sets	ASD-denovo-nonsyn	84622	ZNF594
NPD_Sets	ASD-denovo-nonsyn	9871	SEC24D
NPD_Sets	ASD-denovo-nonsyn	795	S100G
NPD_Sets	ASD-denovo-nonsyn	11113	CIT
NPD_Sets	ASD-denovo-nonsyn	56603	CYP26B1
NPD_Sets	ASD-denovo-nonsyn	9891	NUAK1
NPD_Sets	ASD-denovo-nonsyn	23102	TBC1D2B
NPD_Sets	ASD-denovo-nonsyn	259308	FAM205A
NPD_Sets	ASD-denovo-nonsyn	27185	DISC1
NPD_Sets	ASD-denovo-nonsyn	8803	SUCLA2

NPD_Sets	ASD-denovo-nonsyn	23126	POGZ
NPD_Sets	ASD-denovo-nonsyn	55799	CACNA2D3
NPD_Sets	ASD-denovo-nonsyn	1981	EIF4G1
NPD_Sets	ASD-denovo-nonsyn	6599	SMARCC1
NPD_Sets	ASD-denovo-nonsyn	6601	SMARCC2
NPD_Sets	ASD-denovo-nonsyn	8535	CBX4
NPD_Sets	ASD-denovo-nonsyn	340554	ZC3H12B
NPD_Sets	ASD-denovo-nonsyn	57673	BEND3
NPD_Sets	ASD-denovo-nonsyn	54596	L1TD1
NPD_Sets	ASD-denovo-nonsyn	203062	TSNARE1
NPD_Sets	ASD-denovo-nonsyn	56729	RETN
NPD_Sets	ASD-denovo-nonsyn	259215	LY6G6F
NPD_Sets	ASD-denovo-nonsyn	5649	RELN
NPD_Sets	ASD-denovo-nonsyn	146760	RTN4RL1
NPD_Sets	ASD-denovo-nonsyn	51720	UIMC1
NPD_Sets	ASD-denovo-nonsyn	7846	TUBA1A
NPD_Sets	ASD-denovo-nonsyn	6326	SCN2A
NPD_Sets	ASD-denovo-nonsyn	10588	MTHFS
NPD_Sets	ASD-denovo-nonsyn	10786	SLC17A3
NPD_Sets	ASD-denovo-nonsyn	7249	TSC2
NPD_Sets	ASD-denovo-nonsyn	100534599	ISY1-RAB43
NPD_Sets	ASD-denovo-nonsyn	54861	SNRK
NPD_Sets	ASD-denovo-nonsyn	22949	PTGR1
NPD_Sets	ASD-denovo-nonsyn	57721	METTL14
NPD_Sets	ASD-denovo-nonsyn	79066	METTL16
NPD_Sets	ASD-denovo-nonsyn	10210	TOPORS
NPD_Sets	ASD-denovo-nonsyn	285175	UNC80
NPD_Sets	ASD-denovo-nonsyn	6421	SFPQ
NPD_Sets	ASD-denovo-nonsyn	53	ACP2
NPD_Sets	ASD-denovo-nonsyn	163882	CNST
NPD_Sets	ASD-denovo-nonsyn	51527	GSKIP
NPD_Sets	ASD-denovo-nonsyn	2904	GRIN2B
NPD_Sets	ASD-denovo-nonsyn	64855	FAM129B
NPD_Sets	ASD-denovo-nonsyn	22941	SHANK2
NPD_Sets	ASD-denovo-nonsyn	23347	SMCHD1
NPD_Sets	ASD-denovo-nonsyn	414332	LCN10
NPD_Sets	ASD-denovo-nonsyn	285513	GPRIN3
NPD_Sets	ASD-denovo-nonsyn	8857	FCGBP
NPD_Sets	ASD-denovo-nonsyn	58986	TMEM8A
NPD_Sets	ASD-denovo-nonsyn	10451	VAV3
NPD_Sets	ASD-denovo-nonsyn	5547	PRCP
NPD_Sets	ASD-denovo-nonsyn	115330	GPR146
NPD_Sets	ASD-denovo-nonsyn	126017	ZNF813
NPD_Sets	ASD-denovo-nonsyn	342908	ZNF404
NPD_Sets	ASD-denovo-nonsyn	353274	ZNF445
NPD_Sets	ASD-denovo-nonsyn	283375	SLC39A5
NPD_Sets	ASD-denovo-nonsyn	7113	TMPRSS2
NPD_Sets	ASD-denovo-nonsyn	23288	IQCE
NPD_Sets	ASD-denovo-nonsyn	11072	DUSP14
NPD_Sets	ASD-denovo-nonsyn	128853	DUSP15
NPD_Sets	ASD-denovo-nonsyn	54629	FAM63B
NPD_Sets	ASD-denovo-nonsyn	144406	WDR66
NPD_Sets	ASD-denovo-nonsyn	1303	COL12A1
NPD_Sets	ASD-denovo-nonsyn	5168	ENPP2
NPD_Sets	ASD-denovo-nonsyn	9908	G3BP2
NPD_Sets	ASD-denovo-nonsyn	8452	CUL3
NPD_Sets	ASD-denovo-nonsyn	8065	CUL5
NPD_Sets	ASD-denovo-nonsyn	339559	ZFP69

NPD_Sets	ASD-denovo-nonsyn	84465	MEGF11
NPD_Sets	ASD-denovo-nonsyn	23075	SWAP70
NPD_Sets	ASD-denovo-nonsyn	3678	ITGA5
NPD_Sets	ASD-denovo-nonsyn	57556	SEMA6A
NPD_Sets	ASD-denovo-nonsyn	10716	TBR1
NPD_Sets	ASD-denovo-nonsyn	374378	GALNT18
NPD_Sets	ASD-denovo-nonsyn	3679	ITGA7
NPD_Sets	ASD-denovo-nonsyn	389362	PSMG4
NPD_Sets	ASD-denovo-nonsyn	343413	FCRL6
NPD_Sets	ASD-denovo-nonsyn	3140	MR1
NPD_Sets	ASD-denovo-nonsyn	203	AK1
NPD_Sets	ASD-denovo-nonsyn	53616	ADAM22
NPD_Sets	ASD-denovo-nonsyn	8573	CASK
NPD_Sets	ASD-denovo-nonsyn	83667	SESN2
NPD_Sets	ASD-denovo-nonsyn	10787	NCKAP1
NPD_Sets	ASD-denovo-nonsyn	84717	HDGFRP2
NPD_Sets	ASD-denovo-nonsyn	4583	MUC2
NPD_Sets	ASD-denovo-nonsyn	1586	CYP17A1
NPD_Sets	ASD-denovo-nonsyn	4585	MUC4
NPD_Sets	ASD-denovo-nonsyn	55728	N4BP2
NPD_Sets	ASD-denovo-nonsyn	4627	MYH9
NPD_Sets	ASD-denovo-nonsyn	55750	AGK
NPD_Sets	ASD-denovo-nonsyn	257106	ARHGAP30
NPD_Sets	ASD-denovo-nonsyn	4052	LTBP1
NPD_Sets	ASD-denovo-nonsyn	7365	UGT2B10
NPD_Sets	ASD-denovo-nonsyn	63925	ZNF335
NPD_Sets	ASD-denovo-nonsyn	11100	HNRNPUL1
NPD_Sets	ASD-denovo-nonsyn	23358	USP24
NPD_Sets	ASD-denovo-nonsyn	7840	ALMS1
NPD_Sets	ASD-denovo-nonsyn	391189	OR11L1
NPD_Sets	ASD-denovo-nonsyn	5627	PROS1
NPD_Sets	ASD-denovo-nonsyn	111	ADCY5
NPD_Sets	ASD-denovo-nonsyn	152006	RNF38
NPD_Sets	ASD-denovo-nonsyn	3619	INCENP
NPD_Sets	ASD-denovo-nonsyn	11173	ADAMTS7
NPD_Sets	ASD-denovo-nonsyn	221037	JMJD1C
NPD_Sets	ASD-denovo-nonsyn	7581	ZNF33A
NPD_Sets	ASD-denovo-nonsyn	1105	CHD1
NPD_Sets	ASD-denovo-nonsyn	116211	TM4SF19
NPD_Sets	ASD-denovo-nonsyn	1106	CHD2
NPD_Sets	ASD-denovo-nonsyn	1107	CHD3
NPD_Sets	ASD-denovo-nonsyn	971	CD72
NPD_Sets	ASD-denovo-nonsyn	6773	STAT2
NPD_Sets	ASD-denovo-nonsyn	9918	NCAPD2
NPD_Sets	ASD-denovo-nonsyn	55636	CHD7
NPD_Sets	ASD-denovo-nonsyn	10847	SRCAP
NPD_Sets	ASD-denovo-nonsyn	27086	FOXP1
NPD_Sets	ASD-denovo-nonsyn	57680	CHD8
NPD_Sets	ASD-denovo-nonsyn	199974	CYP4Z1
NPD_Sets	ASD-denovo-nonsyn	1601	DAB2
NPD_Sets	ASD-denovo-nonsyn	2996	GYPE
NPD_Sets	ASD-denovo-nonsyn	1499	CTNNB1
NPD_Sets	ASD-denovo-nonsyn	4051	CYP4F3
NPD_Sets	ASD-denovo-nonsyn	200150	PLD5
NPD_Sets	ASD-denovo-nonsyn	64478	CSMD1
NPD_Sets	ASD-denovo-nonsyn	84812	PLCD4
NPD_Sets	ASD-denovo-nonsyn	23195	MDN1
NPD_Sets	ASD-denovo-nonsyn	85452	KIAA1751

NPD_Sets	ASD-denovo-nonsyn	55133	SRBD1
NPD_Sets	ASD-denovo-nonsyn	26147	PHF19
NPD_Sets	ASD-denovo-nonsyn	284827	KRTAP13-4
NPD_Sets	ASD-denovo-nonsyn	667	DST
NPD_Sets	ASD-denovo-nonsyn	57205	ATP10D
NPD_Sets	ASD-denovo-nonsyn	23389	MED13L
NPD_Sets	ASD-denovo-nonsyn	27332	ZNF638
NPD_Sets	ASD-denovo-nonsyn	1845	DUSP3
NPD_Sets	ASD-denovo-nonsyn	27327	TNRC6A
NPD_Sets	ASD-denovo-nonsyn	26155	NOC2L
NPD_Sets	ASD-denovo-nonsyn	84694	GJA10
NPD_Sets	ASD-denovo-nonsyn	404636	FAM45A
NPD_Sets	ASD-denovo-nonsyn	51816	CECR1
NPD_Sets	ASD-denovo-nonsyn	81050	OR5AC2
NPD_Sets	ASD-denovo-nonsyn	79057	PRRG3
NPD_Sets	ASD-denovo-nonsyn	57617	VPS18
NPD_Sets	ASD-denovo-nonsyn	10319	LAMC3
NPD_Sets	ASD-denovo-nonsyn	4921	DDR2
NPD_Sets	ASD-denovo-nonsyn	28514	DLL1
NPD_Sets	ASD-denovo-nonsyn	2975	GTF3C1
NPD_Sets	ASD-denovo-nonsyn	11011	TLK2
NPD_Sets	ASD-denovo-nonsyn	1642	DDB1
NPD_Sets	ASD-denovo-nonsyn	7536	SF1
NPD_Sets	ASD-denovo-nonsyn	4650	MYO9B
NPD_Sets	ASD-denovo-nonsyn	2802	GOLGA3
NPD_Sets	ASD-denovo-nonsyn	2803	GOLGA4
NPD_Sets	ASD-denovo-nonsyn	23077	MYCBP2
NPD_Sets	ASD-denovo-nonsyn	5364	PLXNB1
NPD_Sets	ASD-denovo-nonsyn	23654	PLXNB2
NPD_Sets	ASD-denovo-nonsyn	143689	PIWIL4
NPD_Sets	ASD-denovo-nonsyn	2053	EPHX2
NPD_Sets	ASD-denovo-nonsyn	7812	CSDE1
NPD_Sets	ASD-denovo-nonsyn	23526	HMHA1
NPD_Sets	ASD-denovo-nonsyn	54737	MPHOSPH8
NPD_Sets	ASD-denovo-nonsyn	4173	MCM4
NPD_Sets	ASD-denovo-nonsyn	55631	LRRC40
NPD_Sets	ASD-denovo-nonsyn	81031	SLC2A10
NPD_Sets	ASD-denovo-nonsyn	23258	DENND5A
NPD_Sets	ASD-denovo-nonsyn	160518	DENND5B
NPD_Sets	ASD-denovo-nonsyn	84231	TRAF7
NPD_Sets	ASD-denovo-nonsyn	81567	TXNDC5
NPD_Sets	ASD-denovo-nonsyn	11276	SYNRG
NPD_Sets	ASD-denovo-nonsyn	53919	SLCO1C1
NPD_Sets	ASD-denovo-nonsyn	9635	CLCA2
NPD_Sets	ASD-denovo-nonsyn	6261	RYR1
NPD_Sets	ASD-denovo-nonsyn	649	BMP1
NPD_Sets	ASD-denovo-nonsyn	10721	POLQ
NPD_Sets	ASD-denovo-nonsyn	977	CD151
NPD_Sets	ASD-denovo-nonsyn	577	BAI3
NPD_Sets	ASD-denovo-nonsyn	5796	PTPRK
NPD_Sets	ASD-denovo-nonsyn	9690	UBE3C
NPD_Sets	ASD-denovo-nonsyn	5797	PTPRM
NPD_Sets	ASD-denovo-nonsyn	4162	MCAM
NPD_Sets	ASD-denovo-nonsyn	23394	ADNP
NPD_Sets	ASD-denovo-nonsyn	161725	OTUD7A
NPD_Sets	ASD-denovo-nonsyn	51111	SUV420H1
NPD_Sets	ASD-denovo-nonsyn	23199	GSE1
NPD_Sets	ASD-denovo-nonsyn	140883	ZNF280B

NPD_Sets	ASD-denovo-nonsyn	64648	SPANXD
NPD_Sets	ASD-denovo-nonsyn	171489	SPANXE
NPD_Sets	ASD-denovo-nonsyn	285527	FRYL
NPD_Sets	ASD-denovo-nonsyn	63035	BCORL1
NPD_Sets	ASD-denovo-nonsyn	50839	TAS2R10
NPD_Sets	ASD-denovo-nonsyn	6794	STK11
NPD_Sets	ASD-denovo-nonsyn	10044	SH2D3C
NPD_Sets	ASD-denovo-nonsyn	7051	TGM1
NPD_Sets	ASD-denovo-nonsyn	9737	GPRASP1
NPD_Sets	ASD-denovo-nonsyn	2335	FN1
NPD_Sets	ASD-denovo-nonsyn	7053	TGM3
NPD_Sets	ASD-denovo-nonsyn	57510	XPO5
NPD_Sets	ASD-denovo-nonsyn	83394	PITPNM3
NPD_Sets	ASD-denovo-nonsyn	29072	SETD2
NPD_Sets	ASD-denovo-nonsyn	57685	CACHD1
NPD_Sets	ASD-denovo-nonsyn	22999	RIMS1
NPD_Sets	ASD-denovo-nonsyn	1859	DYRK1A
NPD_Sets	ASD-denovo-nonsyn	6840	SVIL
NPD_Sets	ASD-denovo-nonsyn	9699	RIMS2
NPD_Sets	ASD-denovo-nonsyn	55209	SETD5
NPD_Sets	ASD-denovo-nonsyn	2002	ELK1
NPD_Sets	ASD-denovo-nonsyn	6734	SRPR
NPD_Sets	ASD-denovo-nonsyn	54103	GSAP
NPD_Sets	ASD-denovo-nonsyn	27243	CHMP2A
NPD_Sets	ASD-denovo-nonsyn	55777	MBD5
NPD_Sets	ASD-denovo-nonsyn	29850	TRPM5
NPD_Sets	ASD-denovo-nonsyn	642636	RAD21L1
NPD_Sets	ASD-denovo-nonsyn	54822	TRPM7
NPD_Sets	ASD-denovo-nonsyn	3787	KCNS1
NPD_Sets	ASD-denovo-nonsyn	23241	PACS2
NPD_Sets	ASD-denovo-nonsyn	3790	KCNS3
NPD_Sets	ASD-denovo-nonsyn	3746	KCNC1
NPD_Sets	ASD-denovo-nonsyn	3441	IFNA4
NPD_Sets	ASD-denovo-nonsyn	344787	ZNF860
NPD_Sets	ASD-denovo-nonsyn	2195	FAT1
NPD_Sets	ASD-denovo-nonsyn	9203	ZMYM3
NPD_Sets	ASD-denovo-nonsyn	26036	ZNF451
NPD_Sets	ASD-denovo-nonsyn	3069	HDLBP
NPD_Sets	ASD-denovo-nonsyn	6522	SLC4A2
NPD_Sets	ASD-denovo-nonsyn	57717	PCDHB16
NPD_Sets	ASD-denovo-nonsyn	221188	GPR114
NPD_Sets	ASD-denovo-nonsyn	7468	WHSC1
NPD_Sets	ASD-denovo-nonsyn	50700	RDH8
NPD_Sets	ASD-denovo-nonsyn	64834	ELOVL1
NPD_Sets	ASD-denovo-nonsyn	8120	AP3B2
NPD_Sets	ASD-denovo-nonsyn	166752	FREM3
NPD_Sets	ASD-denovo-nonsyn	84168	ANTXR1
NPD_Sets	ASD-denovo-nonsyn	221178	SPATA13
NPD_Sets	ASD-denovo-nonsyn	81035	COLEC12
NPD_Sets	ASD-denovo-nonsyn	219469	OR8H1
NPD_Sets	ASD-denovo-nonsyn	64327	LMBR1
NPD_Sets	ASD-denovo-nonsyn	113146	AHNAK2
NPD_Sets	ASD-denovo-nonsyn	950	SCARB2
NPD_Sets	ASD-denovo-nonsyn	5663	PSEN1
NPD_Sets	ASD-denovo-nonsyn	3690	ITGB3
NPD_Sets	ASD-denovo-nonsyn	8666	EIF3G
NPD_Sets	ASD-denovo-nonsyn	2562	GABRB3
NPD_Sets	ASD-denovo-nonsyn	83659	TEKT1

NPD_Sets	ASD-denovo-nonsyn	56131	PCDHB4
NPD_Sets	ASD-denovo-nonsyn	9857	CEP350
NPD_Sets	ASD-denovo-nonsyn	9797	TATDN2
NPD_Sets	ASD-denovo-nonsyn	22993	HMGXB3
NPD_Sets	ASD-denovo-nonsyn	6197	RPS6KA3
NPD_Sets	ASD-denovo-nonsyn	284254	DYNAP
NPD_Sets	ASD-denovo-nonsyn	80332	ADAM33
NPD_Sets	ASD-denovo-nonsyn	64207	IRF2BPL
NPD_Sets	ASD-denovo-nonsyn	360023	ZBTB41
NPD_Sets	ASD-denovo-nonsyn	493	ATP2B4
NPD_Sets	ASD-denovo-nonsyn	287	ANK2
NPD_Sets	ASD-denovo-nonsyn	55684	RABL6
NPD_Sets	ASD-denovo-nonsyn	288	ANK3
NPD_Sets	ASD-denovo-nonsyn	203859	ANO5
NPD_Sets	ASD-denovo-nonsyn	339122	RAB43
NPD_Sets	ASD-denovo-nonsyn	57608	KIAA1462
NPD_Sets	ASD-denovo-nonsyn	85453	TSPYL5
NPD_Sets	ASD-denovo-nonsyn	206338	AQPEP
NPD_Sets	ASD-denovo-nonsyn	4854	NOTCH3
NPD_Sets	ASD-denovo-nonsyn	50614	GALNT9
NPD_Sets	ASD-denovo-nonsyn	54962	TIPIN
NPD_Sets	ASD-denovo-nonsyn	142686	ASB14
NPD_Sets	ASD-denovo-nonsyn	79867	TCTN2
NPD_Sets	ASD-denovo-nonsyn	9056	SLC7A7
NPD_Sets	ASD-denovo-nonsyn	196743	PAOX
NPD_Sets	ASD-denovo-nonsyn	84251	SGIP1
NPD_Sets	ASD-denovo-nonsyn	9391	CIAO1
NPD_Sets	ASD-denovo-nonsyn	3283	HSD3B1
NPD_Sets	ASD-denovo-nonsyn	25890	ABI3BP
NPD_Sets	ASD-denovo-nonsyn	344752	AADACL2
NPD_Sets	ASD-denovo-nonsyn	100528021	ST20-MTHFS
NPD_Sets	ASD-denovo-nonsyn	390078	OR52E6
NPD_Sets	ASD-denovo-nonsyn	1045	CDX2
NPD_Sets	ASD-denovo-nonsyn	6487	ST3GAL3
NPD_Sets	ASD-denovo-nonsyn	10402	ST3GAL6
NPD_Sets	ASD-denovo-nonsyn	5049	PAFAH1B2
NPD_Sets	ASD-denovo-nonsyn	4628	MYH10
NPD_Sets	ASD-denovo-nonsyn	590	BCHE
NPD_Sets	ASD-denovo-nonsyn	1003	CDH5
NPD_Sets	ASD-denovo-nonsyn	4306	NR3C2
NPD_Sets	ASD-denovo-nonsyn	23071	ERP44
NPD_Sets	ASD-denovo-nonsyn	84519	ACRBP
NPD_Sets	ASD-denovo-nonsyn	5578	PRKCA
NPD_Sets	ASD-denovo-nonsyn	1605	DAG1
NPD_Sets	ASD-denovo-nonsyn	718	C3
NPD_Sets	ASD-denovo-nonsyn	5456	POU3F4
NPD_Sets	ASD-denovo-nonsyn	5754	PTK7
NPD_Sets	ASD-denovo-nonsyn	26094	DCAF4
NPD_Sets	ASD-denovo-nonsyn	8816	DCAF5
NPD_Sets	ASD-denovo-nonsyn	66002	CYP4F12
NPD_Sets	ASD-denovo-nonsyn	23304	UBR2
NPD_Sets	ASD-denovo-nonsyn	8985	PLOD3
NPD_Sets	ASD-denovo-nonsyn	254048	UBN2
NPD_Sets	ASD-denovo-nonsyn	130507	UBR3
NPD_Sets	ASD-denovo-nonsyn	9656	MDC1
NPD_Sets	ASD-denovo-nonsyn	23283	CSTF2T
NPD_Sets	ASD-denovo-nonsyn	390999	PRAMEF12
NPD_Sets	ASD-denovo-nonsyn	56624	ASAH2

NPD_Sets	ASD-denovo-nonsyn	91614	DEPDC7
NPD_Sets	ASD-denovo-nonsyn	54936	ADPRHL2
NPD_Sets	ASD-denovo-nonsyn	7273	TTN
NPD_Sets	ASD-denovo-nonsyn	8898	MTMR2
NPD_Sets	ASD-denovo-nonsyn	89797	NAV2
NPD_Sets	ASD-denovo-nonsyn	7837	PXDN
NPD_Sets	ASD-denovo-nonsyn	84146	ZNF644
NPD_Sets	ASD-denovo-nonsyn	55159	RFWD3
NPD_Sets	ASD-denovo-nonsyn	23262	PPIP5K2
NPD_Sets	ASD-denovo-nonsyn	23266	LPHN2
NPD_Sets	ASD-denovo-nonsyn	66036	MTMR9
NPD_Sets	ASD-denovo-nonsyn	89846	FGD3
NPD_Sets	ASD-denovo-nonsyn	165904	XIRP1
NPD_Sets	ASD-denovo-nonsyn	100534611	TM4SF19-TCTEX1D2
NPD_Sets	ASD-denovo-nonsyn	55193	PBRM1
NPD_Sets	ASD-denovo-nonsyn	54980	C2orf42
NPD_Sets	ASD-denovo-nonsyn	158471	PRUNE2
NPD_Sets	ASD-denovo-nonsyn	27340	UTP20
NPD_Sets	ASD-denovo-nonsyn	10785	WDR4
NPD_Sets	ASD-denovo-nonsyn	144453	BEST3
NPD_Sets	ASD-denovo-nonsyn	7025	NR2F1
NPD_Sets	ASD-denovo-nonsyn	220001	VWCE
NPD_Sets	ASD-denovo-nonsyn	162394	SLFN5
NPD_Sets	ASD-denovo-nonsyn	84570	COL25A1
NPD_Sets	ASD-denovo-nonsyn	375775	PNPLA7
NPD_Sets	ASD-denovo-nonsyn	26005	C2CD3
NPD_Sets	ASD-denovo-nonsyn	55739	CARKD
NPD_Sets	ASD-denovo-nonsyn	11188	NISCH
NPD_Sets	ASD-denovo-nonsyn	8458	TTF2
NPD_Sets	ASD-denovo-nonsyn	9024	BRSK2
NPD_Sets	ASD-denovo-nonsyn	1660	DHX9
NPD_Sets	ASD-denovo-nonsyn	54014	BRWD1
NPD_Sets	ASD-denovo-nonsyn	9703	KIAA0100
NPD_Sets	ASD-denovo-nonsyn	9611	NCOR1
NPD_Sets	ASD-denovo-nonsyn	64118	DUS1L
NPD_Sets	ASD-denovo-nonsyn	20	ABCA2
NPD_Sets	ASD-denovo-nonsyn	11146	GLMN
NPD_Sets	ASD-denovo-nonsyn	157922	CAMSAP1
NPD_Sets	ASD-denovo-nonsyn	280	AMY2B
NPD_Sets	ASD-denovo-nonsyn	57496	MKL2
NPD_Sets	ASD-denovo-nonsyn	2041	EPHA1
NPD_Sets	ASD-denovo-nonsyn	57634	EP400
NPD_Sets	ASD-denovo-nonsyn	117854	TRIM6
NPD_Sets	ASD-denovo-nonsyn	116986	AGAP2
NPD_Sets	ASD-denovo-nonsyn	56886	UGGT1
NPD_Sets	ASD-denovo-nonsyn	3778	KCNMA1
NPD_Sets	ASD-denovo-nonsyn	2200	FBN1
NPD_Sets	ASD-denovo-nonsyn	27077	B9D1
NPD_Sets	ASD-denovo-nonsyn	1186	CLCN7
NPD_Sets	ASD-denovo-nonsyn	5603	MAPK13
NPD_Sets	ASD-denovo-nonsyn	22990	PCNX
NPD_Sets	ASD-denovo-nonsyn	27352	SGSM3
NPD_Sets	ASD-denovo-nonsyn	57140	RNPEPL1
NPD_Sets	ASD-denovo-nonsyn	1973	EIF4A1
NPD_Sets	ASD-denovo-nonsyn	1144	CHRND
NPD_Sets	ASD-denovo-nonsyn	5595	MAPK3
NPD_Sets	ASD-denovo-nonsyn	27131	SNX5
NPD_Sets	ASD-denovo-nonsyn	27044	SND1

NPD_Sets	ASD-denovo-nonsyn	3708	ITPR1
NPD_Sets	ASD-denovo-nonsyn	26166	RGS22
NPD_Sets	ASD-denovo-nonsyn	9569	GTF2IRD1
NPD_Sets	ASD-denovo-nonsyn	146754	DNAH2
NPD_Sets	ASD-denovo-nonsyn	390162	OR5M9
NPD_Sets	ASD-denovo-nonsyn	6812	STXBP1
NPD_Sets	ASD-denovo-nonsyn	23513	SCRIB
NPD_Sets	ASD-denovo-nonsyn	1767	DNAH5
NPD_Sets	ASD-denovo-nonsyn	6814	STXBP3
NPD_Sets	ASD-denovo-nonsyn	222658	KCTD20
NPD_Sets	ASD-denovo-nonsyn	1770	DNAH9
NPD_Sets	ASD-denovo-nonsyn	7150	TOP1
NPD_Sets	ASD-denovo-nonsyn	51107	APH1A
NPD_Sets	ASD-denovo-nonsyn	65110	UPF3A
NPD_Sets	ASD-denovo-nonsyn	2742	GLRA2
NPD_Sets	ASD-denovo-nonsyn	9899	SV2B
NPD_Sets	ASD-denovo-nonsyn	54545	MTMR12
NPD_Sets	ASD-denovo-nonsyn	5133	PDCD1
NPD_Sets	ASD-denovo-nonsyn	23082	PPRC1
NPD_Sets	ASD-denovo-nonsyn	26523	AGO1
NPD_Sets	ASD-denovo-nonsyn	8408	ULK1
NPD_Sets	ASD-denovo-nonsyn	4987	OPRL1
NPD_Sets	ASD-denovo-nonsyn	56302	TRPV5
NPD_Sets	ASD-denovo-nonsyn	5442	POLRMT
NPD_Sets	ASD-denovo-nonsyn	51665	ASB1
NPD_Sets	ASD-denovo-nonsyn	4703	NEB
NPD_Sets	ASD-denovo-nonsyn	6540	SLC6A13
NPD_Sets	ASD-denovo-nonsyn	26040	SETBP1
NPD_Sets	ASD-denovo-nonsyn	8554	PIAS1
NPD_Sets	ASD-denovo-nonsyn	343450	KCNT2
NPD_Sets	ASD-denovo-nonsyn	11218	DDX20
NPD_Sets	ASD-denovo-nonsyn	23001	WDFY3
NPD_Sets	ASD-denovo-nonsyn	55954	ZMAT5
NPD_Sets	ASD-denovo-nonsyn	57670	KIAA1549
NPD_Sets	ASD-denovo-nonsyn	147923	ZNF420
NPD_Sets	ASD-denovo-nonsyn	154865	IQUB
NPD_Sets	ASD-denovo-nonsyn	9096	TBX18
NPD_Sets	ASD-denovo-nonsyn	23090	ZNF423
NPD_Sets	ASD-denovo-nonsyn	7415	VCP
NPD_Sets	ASD-denovo-nonsyn	2109	ETFB
NPD_Sets	ASD-denovo-nonsyn	65083	NOL6
NPD_Sets	ASD-denovo-nonsyn	11198	SUPT16H
NPD_Sets	ASD-denovo-nonsyn	10985	GCN1L1
NPD_Sets	ASD-denovo-nonsyn	23405	DICER1
NPD_Sets	ASD-denovo-nonsyn	83552	MFRP
NPD_Sets	ASD-denovo-nonsyn	79812	MMRN2
NPD_Sets	ASD-denovo-nonsyn	346389	MACC1
NPD_Sets	ASD-denovo-nonsyn	10886	NPFFR2
NPD_Sets	ASD-denovo-nonsyn	2915	GRM5
NPD_Sets	ASD-denovo-nonsyn	23660	ZKSCAN5
NPD_Sets	ASD-denovo-nonsyn	9126	SMC3
NPD_Sets	ASD-denovo-nonsyn	4189	DNAJB9
NPD_Sets	ASD-denovo-nonsyn	2917	GRM7
NPD_Sets	ASD-denovo-nonsyn	9378	NRXN1
NPD_Sets	ASD-denovo-nonsyn	50618	ITSN2
NPD_Sets	ASD-denovo-nonsyn	9166	EBAG9
NPD_Sets	ASD-denovo-nonsyn	4929	NR4A2
NPD_Sets	ASD-denovo-nonsyn	94137	RP1L1

NPD_Sets	ASD-denovo-nonsyn	51555	PEX5L
NPD_Sets	ASD-denovo-nonsyn	84919	PPP1R15B
NPD_Sets	ASD-denovo-nonsyn	89941	RHOT2
NPD_Sets	ASD-denovo-nonsyn	54870	QRICH1
NPD_Sets	ASD-denovo-nonsyn	5079	PAX5
NPD_Sets	ASD-denovo-nonsyn	55283	MCOLN3
NPD_Sets	ASD-denovo-nonsyn	26267	FBXO10
NPD_Sets	ASD-denovo-nonsyn	57715	SEMA4G
NPD_Sets	ASD-denovo-nonsyn	57125	PLXDC1
NPD_Sets	ASD-denovo-nonsyn	23506	GLTSCR1L
NPD_Sets	ASD-denovo-nonsyn	1154	CISH
NPD_Sets	ASD-denovo-nonsyn	23167	EFR3A
NPD_Sets	ASD-denovo-nonsyn	83992	CTTNBP2
NPD_Sets	ASD-denovo-nonsyn	9221	NOLC1
NPD_Sets	ASD-denovo-nonsyn	84953	MICALCL
NPD_Sets	ASD-denovo-nonsyn	84893	FBXO18
NPD_Sets	ASD-denovo-nonsyn	144501	KRT80
NPD_Sets	ASD-denovo-nonsyn	1842	ECM2
NPD_Sets	ASD-denovo-nonsyn	8029	CUBN
NPD_Sets	ASD-denovo-nonsyn	10152	ABI2
NPD_Sets	ASD-denovo-nonsyn	4134	MAP4
NPD_Sets	ASD-denovo-nonsyn	64848	YTHDC2
NPD_Sets	ASD-denovo-nonsyn	57589	KIAA1432
NPD_Sets	ASD-denovo-nonsyn	64135	IFIH1
NPD_Sets	ASD-denovo-nonsyn	53833	IL20RB
NPD_Sets	ASD-denovo-nonsyn	6683	SPAST
NPD_Sets	ASD-denovo-nonsyn	22854	NTNG1
NPD_Sets	ASD-denovo-nonsyn	56136	PCDHA13
NPD_Sets	FMRP-Ascano	2043	EPHA4
NPD_Sets	FMRP-Ascano	5108	PCM1
NPD_Sets	FMRP-Ascano	57491	AHRR
NPD_Sets	FMRP-Ascano	55717	WDR11
NPD_Sets	FMRP-Ascano	1954	MEGF8
NPD_Sets	FMRP-Ascano	9517	SPTLC2
NPD_Sets	FMRP-Ascano	148156	ZNF558
NPD_Sets	FMRP-Ascano	3607	FOXK2
NPD_Sets	FMRP-Ascano	25902	MTHFD1L
NPD_Sets	FMRP-Ascano	115426	UHRF2
NPD_Sets	FMRP-Ascano	55253	TYW1
NPD_Sets	FMRP-Ascano	10808	HSPH1
NPD_Sets	FMRP-Ascano	3092	HIP1
NPD_Sets	FMRP-Ascano	53349	ZFYVE1
NPD_Sets	FMRP-Ascano	27112	FAM155B
NPD_Sets	FMRP-Ascano	56919	DHX33
NPD_Sets	FMRP-Ascano	9344	TAOK2
NPD_Sets	FMRP-Ascano	10296	MAEA
NPD_Sets	FMRP-Ascano	1000	CDH2
NPD_Sets	FMRP-Ascano	9361	LONP1
NPD_Sets	FMRP-Ascano	51520	LARS
NPD_Sets	FMRP-Ascano	23637	RABGAP1
NPD_Sets	FMRP-Ascano	4241	MFI2
NPD_Sets	FMRP-Ascano	337876	CHSY3
NPD_Sets	FMRP-Ascano	8266	UBL4A
NPD_Sets	FMRP-Ascano	5213	PFKM
NPD_Sets	FMRP-Ascano	4627	MYH9
NPD_Sets	FMRP-Ascano	2768	GNA12
NPD_Sets	FMRP-Ascano	79811	SLTM
NPD_Sets	FMRP-Ascano	51780	KDM3B

NPD_Sets	FMRP-Ascano	63934	ZNF667
NPD_Sets	FMRP-Ascano	9564	BCAR1
NPD_Sets	FMRP-Ascano	9732	DOCK4
NPD_Sets	FMRP-Ascano	9394	HS6ST1
NPD_Sets	FMRP-Ascano	23359	FAM189A1
NPD_Sets	FMRP-Ascano	91746	YTHDC1
NPD_Sets	FMRP-Ascano	80821	DDHD1
NPD_Sets	FMRP-Ascano	5834	PYGB
NPD_Sets	FMRP-Ascano	159195	USP54
NPD_Sets	FMRP-Ascano	5927	KDM5A
NPD_Sets	FMRP-Ascano	6850	SYK
NPD_Sets	FMRP-Ascano	84706	GPT2
NPD_Sets	FMRP-Ascano	126375	ZNF792
NPD_Sets	FMRP-Ascano	6777	STAT5B
NPD_Sets	FMRP-Ascano	22950	SLC4A1AP
NPD_Sets	FMRP-Ascano	114928	GPRASP2
NPD_Sets	FMRP-Ascano	23303	KIF13B
NPD_Sets	FMRP-Ascano	57147	SCYL3
NPD_Sets	FMRP-Ascano	3065	HDAC1
NPD_Sets	FMRP-Ascano	81545	FBXO38
NPD_Sets	FMRP-Ascano	116496	FAM129A
NPD_Sets	FMRP-Ascano	85377	MICALL1
NPD_Sets	FMRP-Ascano	55778	ZNF839
NPD_Sets	FMRP-Ascano	25817	FAM19A5
NPD_Sets	FMRP-Ascano	399687	MYO18A
NPD_Sets	FMRP-Ascano	79714	CCDC51
NPD_Sets	FMRP-Ascano	56980	PRDM10
NPD_Sets	FMRP-Ascano	84342	COG8
NPD_Sets	FMRP-Ascano	284403	WDR62
NPD_Sets	FMRP-Ascano	22995	CEP152
NPD_Sets	FMRP-Ascano	387893	SETD8
NPD_Sets	FMRP-Ascano	4008	LMO7
NPD_Sets	FMRP-Ascano	23233	EXOC6B
NPD_Sets	FMRP-Ascano	253461	ZBTB38
NPD_Sets	FMRP-Ascano	10844	TUBGCP2
NPD_Sets	FMRP-Ascano	3308	HSPA4
NPD_Sets	FMRP-Ascano	55275	VPS53
NPD_Sets	FMRP-Ascano	51593	SRRT
NPD_Sets	FMRP-Ascano	57701	NCKAP5L
NPD_Sets	FMRP-Ascano	80223	RAB11FIP1
NPD_Sets	FMRP-Ascano	9704	DHX34
NPD_Sets	FMRP-Ascano	9989	PPP4R1
NPD_Sets	FMRP-Ascano	125950	RAVER1
NPD_Sets	FMRP-Ascano	339175	METTL2A
NPD_Sets	FMRP-Ascano	57679	ALS2
NPD_Sets	FMRP-Ascano	4054	LTBP3
NPD_Sets	FMRP-Ascano	91	ACVR1B
NPD_Sets	FMRP-Ascano	10155	TRIM28
NPD_Sets	FMRP-Ascano	5598	MAPK7
NPD_Sets	FMRP-Ascano	2621	GAS6
NPD_Sets	FMRP-Ascano	4298	MLLT1
NPD_Sets	FMRP-Ascano	51230	PHF20
NPD_Sets	FMRP-Ascano	64425	POLR1E
NPD_Sets	FMRP-Ascano	23152	CIC
NPD_Sets	FMRP-Ascano	2017	CTTN
NPD_Sets	FMRP-Ascano	80124	VCPIP1
NPD_Sets	FMRP-Ascano	7874	USP7
NPD_Sets	FMRP-Ascano	3642	INSM1

NPD_Sets	FMRP-Ascano	10483	SEC23B
NPD_Sets	FMRP-Ascano	23426	GRIP1
NPD_Sets	FMRP-Ascano	257218	SHPRH
NPD_Sets	FMRP-Ascano	30827	CXXC1
NPD_Sets	FMRP-Ascano	547	KIF1A
NPD_Sets	FMRP-Ascano	57646	USP28
NPD_Sets	FMRP-Ascano	83852	SETDB2
NPD_Sets	FMRP-Ascano	2176	FANCC
NPD_Sets	FMRP-Ascano	57448	BIRC6
NPD_Sets	FMRP-Ascano	80179	MYO19
NPD_Sets	FMRP-Ascano	3676	ITGA4
NPD_Sets	FMRP-Ascano	11319	ECD
NPD_Sets	FMRP-Ascano	7443	VRK1
NPD_Sets	FMRP-Ascano	8438	RAD54L
NPD_Sets	FMRP-Ascano	10564	ARFGEF2
NPD_Sets	FMRP-Ascano	64151	NCAPG
NPD_Sets	FMRP-Ascano	9646	CTR9
NPD_Sets	FMRP-Ascano	7267	TTC3
NPD_Sets	FMRP-Ascano	9716	AQR
NPD_Sets	FMRP-Ascano	84138	SLC7A6OS
NPD_Sets	FMRP-Ascano	83540	NUF2
NPD_Sets	FMRP-Ascano	7156	TOP3A
NPD_Sets	FMRP-Ascano	54806	AHI1
NPD_Sets	FMRP-Ascano	9448	MAP4K4
NPD_Sets	FMRP-Ascano	1981	EIF4G1
NPD_Sets	FMRP-Ascano	10999	SLC27A4
NPD_Sets	FMRP-Ascano	56254	RNF20
NPD_Sets	FMRP-Ascano	11169	WDHD1
NPD_Sets	FMRP-Ascano	9314	KLF4
NPD_Sets	FMRP-Ascano	4301	MLLT4
NPD_Sets	FMRP-Ascano	23670	TMEM2
NPD_Sets	FMRP-Ascano	6909	TBX2
NPD_Sets	FMRP-Ascano	56926	NCLN
NPD_Sets	FMRP-Ascano	4591	TRIM37
NPD_Sets	FMRP-Ascano	10994	ILVBL
NPD_Sets	FMRP-Ascano	161582	DYX1C1
NPD_Sets	FMRP-Ascano	60625	DHX35
NPD_Sets	FMRP-Ascano	5116	PCNT
NPD_Sets	FMRP-Ascano	57508	INTS2
NPD_Sets	FMRP-Ascano	641	BLM
NPD_Sets	FMRP-Ascano	57616	TSHZ3
NPD_Sets	FMRP-Ascano	80230	RUFY1
NPD_Sets	FMRP-Ascano	5894	RAF1
NPD_Sets	FMRP-Ascano	80331	DNAJC5
NPD_Sets	FMRP-Ascano	23240	KIAA0922
NPD_Sets	FMRP-Ascano	10970	CKAP4
NPD_Sets	FMRP-Ascano	84034	EMILIN2
NPD_Sets	FMRP-Ascano	103	ADAR
NPD_Sets	FMRP-Ascano	7596	ZNF45
NPD_Sets	FMRP-Ascano	2177	FANCD2
NPD_Sets	FMRP-Ascano	53	ACP2
NPD_Sets	FMRP-Ascano	3980	LIG3
NPD_Sets	FMRP-Ascano	5705	PSMC5
NPD_Sets	FMRP-Ascano	8622	PDE8B
NPD_Sets	FMRP-Ascano	374654	KIF7
NPD_Sets	FMRP-Ascano	57685	CACHD1
NPD_Sets	FMRP-Ascano	58506	SCAF1
NPD_Sets	FMRP-Ascano	374655	ZNF710

NPD_Sets	FMRP-Ascano	4756	NEO1
NPD_Sets	FMRP-Ascano	4810	NHS
NPD_Sets	FMRP-Ascano	158158	RASEF
NPD_Sets	FMRP-Ascano	116987	AGAP1
NPD_Sets	FMRP-Ascano	3313	HSPA9
NPD_Sets	FMRP-Ascano	54862	CC2D1A
NPD_Sets	FMRP-Ascano	5660	PSAP
NPD_Sets	FMRP-Ascano	6448	SGSH
NPD_Sets	FMRP-Ascano	55893	ZNF395
NPD_Sets	FMRP-Ascano	9625	AATK
NPD_Sets	FMRP-Ascano	23211	ZC3H4
NPD_Sets	FMRP-Ascano	9759	HDAC4
NPD_Sets	FMRP-Ascano	4926	NUMA1
NPD_Sets	FMRP-Ascano	79954	NOL10
NPD_Sets	FMRP-Ascano	2774	GNAL
NPD_Sets	FMRP-Ascano	23438	HARS2
NPD_Sets	FMRP-Ascano	51283	BFAR
NPD_Sets	FMRP-Ascano	4605	MYBL2
NPD_Sets	FMRP-Ascano	1362	CPD
NPD_Sets	FMRP-Ascano	64399	HHIP
NPD_Sets	FMRP-Ascano	9128	PRPF4
NPD_Sets	FMRP-Ascano	22856	CHSY1
NPD_Sets	FMRP-Ascano	3202	HOXA5
NPD_Sets	FMRP-Ascano	85450	ITPRIP
NPD_Sets	FMRP-Ascano	9092	SART1
NPD_Sets	FMRP-Ascano	8664	EIF3D
NPD_Sets	FMRP-Ascano	351	APP
NPD_Sets	FMRP-Ascano	8527	DGKD
NPD_Sets	FMRP-Ascano	5422	POLA1
NPD_Sets	FMRP-Ascano	9462	RASAL2
NPD_Sets	FMRP-Ascano	3480	IGF1R
NPD_Sets	FMRP-Ascano	9640	ZNF592
NPD_Sets	FMRP-Ascano	64132	XYLT2
NPD_Sets	FMRP-Ascano	23351	KHNYN
NPD_Sets	FMRP-Ascano	10781	ZNF266
NPD_Sets	FMRP-Ascano	9353	SLIT2
NPD_Sets	FMRP-Ascano	9679	FAM53B
NPD_Sets	FMRP-Ascano	23659	PLA2G15
NPD_Sets	FMRP-Ascano	134957	STXBP5
NPD_Sets	FMRP-Ascano	26133	TRPC4AP
NPD_Sets	FMRP-Ascano	4686	NCBP1
NPD_Sets	FMRP-Ascano	9569	GTF2IRD1
NPD_Sets	FMRP-Ascano	23524	SRRM2
NPD_Sets	FMRP-Ascano	23179	RGL1
NPD_Sets	FMRP-Ascano	1785	DNM2
NPD_Sets	FMRP-Ascano	8602	NOP14
NPD_Sets	FMRP-Ascano	256364	EML3
NPD_Sets	FMRP-Ascano	79633	FAT4
NPD_Sets	FMRP-Ascano	51088	KLHL5
NPD_Sets	FMRP-Ascano	23258	DENND5A
NPD_Sets	FMRP-Ascano	1809	DPYSL3
NPD_Sets	FMRP-Ascano	7049	TGFBR3
NPD_Sets	FMRP-Ascano	27044	SND1
NPD_Sets	FMRP-Ascano	58499	ZNF462
NPD_Sets	FMRP-Ascano	5236	PGM1
NPD_Sets	FMRP-Ascano	125058	TBC1D16
NPD_Sets	FMRP-Ascano	79414	LRFN3
NPD_Sets	FMRP-Ascano	10111	RAD50

NPD_Sets	FMRP-Ascano	4245	MGAT1
NPD_Sets	FMRP-Ascano	650	BMP2
NPD_Sets	FMRP-Ascano	55760	DHX32
NPD_Sets	FMRP-Ascano	57479	PRR12
NPD_Sets	FMRP-Ascano	23301	EHBP1
NPD_Sets	FMRP-Ascano	29781	NCAPH2
NPD_Sets	FMRP-Ascano	197131	UBR1
NPD_Sets	FMRP-Ascano	115950	ZNF653
NPD_Sets	FMRP-Ascano	56886	UGGT1
NPD_Sets	FMRP-Ascano	56931	DUS3L
NPD_Sets	FMRP-Ascano	10144	FAM13A
NPD_Sets	FMRP-Ascano	7692	ZNF133
NPD_Sets	FMRP-Ascano	5792	PTPRF
NPD_Sets	FMRP-Ascano	55568	GALNT10
NPD_Sets	FMRP-Ascano	148398	SAMD11
NPD_Sets	FMRP-Ascano	55718	POLR3E
NPD_Sets	FMRP-Ascano	23042	PDXDC1
NPD_Sets	FMRP-Ascano	23139	MAST2
NPD_Sets	FMRP-Ascano	51366	UBR5
NPD_Sets	FMRP-Ascano	79754	ASB13
NPD_Sets	FMRP-Ascano	51202	DDX47
NPD_Sets	FMRP-Ascano	7128	TNFAIP3
NPD_Sets	FMRP-Ascano	8792	TNFRSF11A
NPD_Sets	FMRP-Ascano	4851	NOTCH1
NPD_Sets	FMRP-Ascano	23132	RAD54L2
NPD_Sets	FMRP-Ascano	9882	TBC1D4
NPD_Sets	FMRP-Ascano	6624	FSCN1
NPD_Sets	FMRP-Ascano	4215	MAP3K3
NPD_Sets	FMRP-Ascano	1039	CDR2
NPD_Sets	FMRP-Ascano	2064	ERBB2
NPD_Sets	FMRP-Ascano	2534	FYN
NPD_Sets	FMRP-Ascano	63893	UBE2O
NPD_Sets	FMRP-Ascano	79147	FKRP
NPD_Sets	FMRP-Ascano	6305	SBF1
NPD_Sets	FMRP-Ascano	6749	SSRP1
NPD_Sets	FMRP-Ascano	23338	PHF15
NPD_Sets	FMRP-Ascano	9785	DHX38
NPD_Sets	FMRP-Ascano	54476	RNF216
NPD_Sets	FMRP-Ascano	9927	MFN2
NPD_Sets	FMRP-Ascano	8467	SMARCA5
NPD_Sets	FMRP-Ascano	476	ATP1A1
NPD_Sets	FMRP-Ascano	211	ALAS1
NPD_Sets	FMRP-Ascano	55677	IWS1
NPD_Sets	FMRP-Ascano	26005	C2CD3
NPD_Sets	FMRP-Ascano	2720	GLB1
NPD_Sets	FMRP-Ascano	7375	USP4
NPD_Sets	FMRP-Ascano	23646	PLD3
NPD_Sets	FMRP-Ascano	404734	ANKHD1-EIF4EBP3
NPD_Sets	FMRP-Ascano	5738	PTGFRN
NPD_Sets	FMRP-Ascano	8567	MADD
NPD_Sets	FMRP-Ascano	54887	UHRF1BP1
NPD_Sets	FMRP-Ascano	64852	TUT1
NPD_Sets	FMRP-Ascano	4691	NCL
NPD_Sets	FMRP-Ascano	84134	TOMM40L
NPD_Sets	FMRP-Ascano	79659	DYNC2H1
NPD_Sets	FMRP-Ascano	8573	CASK
NPD_Sets	FMRP-Ascano	64780	MICAL1
NPD_Sets	FMRP-Ascano	10194	TSHZ1

NPD_Sets	FMRP-Ascano	23028	KDM1A
NPD_Sets	FMRP-Ascano	150737	TTC30B
NPD_Sets	FMRP-Ascano	472	ATM
NPD_Sets	FMRP-Ascano	22937	SCAP
NPD_Sets	FMRP-Ascano	79605	PGBD5
NPD_Sets	FMRP-Ascano	51427	ZNF107
NPD_Sets	FMRP-Ascano	5774	PTPN3
NPD_Sets	FMRP-Ascano	79893	GGNBP2
NPD_Sets	FMRP-Ascano	65095	KRI1
NPD_Sets	FMRP-Ascano	123169	LEO1
NPD_Sets	FMRP-Ascano	8516	ITGA8
NPD_Sets	FMRP-Ascano	2673	GFPT1
NPD_Sets	FMRP-Ascano	9601	PDIA4
NPD_Sets	FMRP-Ascano	2033	EP300
NPD_Sets	FMRP-Ascano	3035	HARS
NPD_Sets	FMRP-Ascano	3550	IK
NPD_Sets	FMRP-Ascano	7090	TLE3
NPD_Sets	FMRP-Ascano	8425	LTBP4
NPD_Sets	FMRP-Ascano	9423	NTN1
NPD_Sets	FMRP-Ascano	24137	KIF4A
NPD_Sets	FMRP-Ascano	2590	GALNT2
NPD_Sets	FMRP-Ascano	399909	PCNXL3
NPD_Sets	FMRP-Ascano	440193	CCDC88C
NPD_Sets	FMRP-Ascano	4522	MTHFD1
NPD_Sets	FMRP-Ascano	26015	RPAP1
NPD_Sets	FMRP-Ascano	9382	COG1
NPD_Sets	FMRP-Ascano	993	CDC25A
NPD_Sets	FMRP-Ascano	9442	MED27
NPD_Sets	FMRP-Ascano	8940	TOP3B
NPD_Sets	FMRP-Ascano	54549	SDK2
NPD_Sets	FMRP-Ascano	6597	SMARCA4
NPD_Sets	FMRP-Ascano	7407	VARS
NPD_Sets	FMRP-Ascano	53616	ADAM22
NPD_Sets	FMRP-Ascano	8828	NRP2
NPD_Sets	FMRP-Ascano	2186	BPTF
NPD_Sets	FMRP-Ascano	146923	RUNDC1
NPD_Sets	FMRP-Ascano	4548	MTR
NPD_Sets	FMRP-Ascano	55178	RNMTL1
NPD_Sets	FMRP-Ascano	23499	MACF1
NPD_Sets	FMRP-Ascano	9826	ARHGEF11
NPD_Sets	FMRP-Ascano	4131	MAP1B
NPD_Sets	FMRP-Ascano	9469	CHST3
NPD_Sets	FMRP-Ascano	5603	MAPK13
NPD_Sets	FMRP-Ascano	22884	WDR37
NPD_Sets	FMRP-Ascano	5361	PLXNA1
NPD_Sets	FMRP-Ascano	8661	EIF3A
NPD_Sets	FMRP-Ascano	23235	SIK2
NPD_Sets	FMRP-Ascano	9771	RAPGEF5
NPD_Sets	FMRP-Ascano	10153	CEBPZ
NPD_Sets	FMRP-Ascano	11124	FAF1
NPD_Sets	FMRP-Ascano	11120	BTN2A1
NPD_Sets	FMRP-Ascano	118	ADD1
NPD_Sets	FMRP-Ascano	57521	RPTOR
NPD_Sets	FMRP-Ascano	64794	DDX31
NPD_Sets	FMRP-Ascano	23432	GPR161
NPD_Sets	FMRP-Ascano	3667	IRS1
NPD_Sets	FMRP-Ascano	163	AP2B1
NPD_Sets	FMRP-Ascano	9644	SH3PXD2A

NPD_Sets	FMRP-Ascano	6872	TAF1
NPD_Sets	FMRP-Ascano	23216	TBC1D1
NPD_Sets	FMRP-Ascano	24145	PANX1
NPD_Sets	FMRP-Ascano	10574	CCT7
NPD_Sets	FMRP-Ascano	5318	PKP2
NPD_Sets	FMRP-Ascano	27245	AHDC1
NPD_Sets	FMRP-Ascano	8777	MPDZ
NPD_Sets	FMRP-Ascano	6091	ROBO1
NPD_Sets	FMRP-Ascano	26153	KIF26A
NPD_Sets	FMRP-Ascano	5588	PRKCQ
NPD_Sets	FMRP-Ascano	11044	PAPD7
NPD_Sets	FMRP-Ascano	8535	CBX4
NPD_Sets	FMRP-Ascano	2009	EML1
NPD_Sets	FMRP-Ascano	10131	TRAP1
NPD_Sets	FMRP-Ascano	25	ABL1
NPD_Sets	FMRP-Ascano	85440	DOCK7
NPD_Sets	FMRP-Ascano	55610	CCDC132
NPD_Sets	FMRP-Ascano	10072	DPP3
NPD_Sets	FMRP-Ascano	2058	EPRS
NPD_Sets	FMRP-Ascano	26267	FBXO10
NPD_Sets	FMRP-Ascano	114789	SLC25A25
NPD_Sets	FMRP-Ascano	23207	PLEKHM2
NPD_Sets	FMRP-Ascano	10765	KDM5B
NPD_Sets	FMRP-Ascano	6839	SUV39H1
NPD_Sets	FMRP-Ascano	117246	FTSJ3
NPD_Sets	FMRP-Ascano	5830	PEX5
NPD_Sets	FMRP-Ascano	5428	POLG
NPD_Sets	FMRP-Ascano	9110	MTMR4
NPD_Sets	FMRP-Ascano	22980	TCF25
NPD_Sets	FMRP-Ascano	57522	SRGAP1
NPD_Sets	FMRP-Ascano	84629	TNRC18
NPD_Sets	FMRP-Ascano	55125	CEP192
NPD_Sets	FMRP-Ascano	5310	PKD1
NPD_Sets	FMRP-Ascano	10181	RBM5
NPD_Sets	FMRP-Ascano	9328	GTF3C5
NPD_Sets	FMRP-Ascano	9183	ZW10
NPD_Sets	FMRP-Ascano	23108	RAP1GAP2
NPD_Sets	FMRP-Ascano	29123	ANKRD11
NPD_Sets	FMRP-Ascano	55898	UNC45A
NPD_Sets	FMRP-Ascano	9733	SART3
NPD_Sets	FMRP-Ascano	84656	GLYR1
NPD_Sets	FMRP-Ascano	79960	PHF17
NPD_Sets	FMRP-Ascano	9793	CKAP5
NPD_Sets	FMRP-Ascano	9219	MTA2
NPD_Sets	FMRP-Ascano	9747	FAM115A
NPD_Sets	FMRP-Ascano	4651	MYO10
NPD_Sets	FMRP-Ascano	3141	HLCS
NPD_Sets	FMRP-Ascano	1612	DAPK1
NPD_Sets	FMRP-Ascano	50	ACO2
NPD_Sets	FMRP-Ascano	4171	MCM2
NPD_Sets	FMRP-Ascano	9510	ADAMTS1
NPD_Sets	FMRP-Ascano	23223	RRP12
NPD_Sets	FMRP-Ascano	255252	LRRC57
NPD_Sets	FMRP-Ascano	8829	NRP1
NPD_Sets	FMRP-Ascano	831	CAST
NPD_Sets	FMRP-Ascano	7203	CCT3
NPD_Sets	FMRP-Ascano	583	BBS2
NPD_Sets	FMRP-Ascano	7186	TRAF2

NPD_Sets	FMRP-Ascano	347902	AMIGO2
NPD_Sets	FMRP-Ascano	25896	INTS7
NPD_Sets	FMRP-Ascano	84167	C19orf44
NPD_Sets	FMRP-Ascano	51001	MTERFD1
NPD_Sets	FMRP-Ascano	23061	TBC1D9B
NPD_Sets	FMRP-Ascano	137964	AGPAT6
NPD_Sets	FMRP-Ascano	79677	SMC6
NPD_Sets	FMRP-Ascano	4176	MCM7
NPD_Sets	FMRP-Ascano	7088	TLE1
NPD_Sets	FMRP-Ascano	9862	MED24
NPD_Sets	FMRP-Ascano	55722	CEP72
NPD_Sets	FMRP-Ascano	80208	SPG11
NPD_Sets	FMRP-Ascano	288	ANK3
NPD_Sets	FMRP-Ascano	84851	TRIM52
NPD_Sets	FMRP-Ascano	25925	ZNF521
NPD_Sets	FMRP-Ascano	93323	HAUS8
NPD_Sets	FMRP-Ascano	9794	MAML1
NPD_Sets	FMRP-Ascano	64411	ARAP3
NPD_Sets	FMRP-Ascano	84444	DOT1L
NPD_Sets	FMRP-Ascano	116985	ARAP1
NPD_Sets	FMRP-Ascano	6786	STIM1
NPD_Sets	FMRP-Ascano	23175	LPIN1
NPD_Sets	FMRP-Ascano	221061	FAM171A1
NPD_Sets	FMRP-Ascano	8427	ZNF282
NPD_Sets	FMRP-Ascano	64118	DUS1L
NPD_Sets	FMRP-Ascano	5802	PTPRS
NPD_Sets	FMRP-Ascano	114826	SMYD4
NPD_Sets	FMRP-Ascano	8660	IRS2
NPD_Sets	FMRP-Ascano	6301	SARS
NPD_Sets	FMRP-Ascano	9258	MFHAS1
NPD_Sets	FMRP-Ascano	53615	MBD3
NPD_Sets	FMRP-Ascano	56905	C15orf39
NPD_Sets	FMRP-Ascano	23361	ZNF629
NPD_Sets	FMRP-Ascano	81570	CLPB
NPD_Sets	FMRP-Ascano	3673	ITGA2
NPD_Sets	FMRP-Ascano	26205	GMEB2
NPD_Sets	FMRP-Ascano	6741	SSB
NPD_Sets	FMRP-Ascano	140730	RIMS4
NPD_Sets	FMRP-Ascano	109	ADCY3
NPD_Sets	FMRP-Ascano	2110	ETFDH
NPD_Sets	FMRP-Ascano	9678	PHF14
NPD_Sets	FMRP-Ascano	6711	SPTBN1
NPD_Sets	FMRP-Ascano	26504	CNNM4
NPD_Sets	FMRP-Ascano	253650	ANKRD18A
NPD_Sets	FMRP-Ascano	5903	RANBP2
NPD_Sets	FMRP-Ascano	23	ABCF1
NPD_Sets	FMRP-Ascano	84162	KIAA1109
NPD_Sets	FMRP-Ascano	54480	CHPF2
NPD_Sets	FMRP-Ascano	1523	CUX1
NPD_Sets	FMRP-Ascano	57704	GBA2
NPD_Sets	FMRP-Ascano	23339	VPS39
NPD_Sets	FMRP-Ascano	2137	EXTL3
NPD_Sets	FMRP-Ascano	25799	ZNF324
NPD_Sets	FMRP-Ascano	4077	NBR1
NPD_Sets	FMRP-Ascano	23332	CLASP1
NPD_Sets	FMRP-Ascano	51602	NOP58
NPD_Sets	FMRP-Ascano	9215	LARGE
NPD_Sets	FMRP-Ascano	340252	ZNF680

NPD_Sets	FMRP-Ascano	2733	GLE1
NPD_Sets	FMRP-Ascano	89781	HPS4
NPD_Sets	FMRP-Ascano	55187	VPS13D
NPD_Sets	FMRP-Ascano	23386	NUDCD3
NPD_Sets	FMRP-Ascano	150465	TTL
NPD_Sets	FMRP-Ascano	644815	FAM83G
NPD_Sets	FMRP-Ascano	9980	DOPEY2
NPD_Sets	FMRP-Ascano	4175	MCM6
NPD_Sets	FMRP-Ascano	9397	NMT2
NPD_Sets	FMRP-Ascano	51429	SNX9
NPD_Sets	FMRP-Ascano	6840	SVIL
NPD_Sets	FMRP-Ascano	55813	UTP6
NPD_Sets	FMRP-Ascano	8720	MBTPS1
NPD_Sets	FMRP-Ascano	9817	KEAP1
NPD_Sets	FMRP-Ascano	79803	HPS6
NPD_Sets	FMRP-Ascano	107	ADCY1
NPD_Sets	FMRP-Ascano	4898	NRD1
NPD_Sets	FMRP-Ascano	22993	HMGXB3
NPD_Sets	FMRP-Ascano	136051	ZNF786
NPD_Sets	FMRP-Ascano	57634	EP400
NPD_Sets	FMRP-Ascano	22920	KIFAP3
NPD_Sets	FMRP-Ascano	55215	FANCI
NPD_Sets	FMRP-Ascano	1314	COPA
NPD_Sets	FMRP-Ascano	11336	EXOC3
NPD_Sets	FMRP-Ascano	8874	ARHGEF7
NPD_Sets	FMRP-Ascano	3978	LIG1
NPD_Sets	FMRP-Ascano	613	BCR
NPD_Sets	FMRP-Ascano	54620	FBXL19
NPD_Sets	FMRP-Ascano	23451	SF3B1
NPD_Sets	FMRP-Ascano	5797	PTPRM
NPD_Sets	FMRP-Ascano	1455	CSNK1G2
NPD_Sets	FMRP-Ascano	7753	ZNF202
NPD_Sets	FMRP-Ascano	8826	IQGAP1
NPD_Sets	FMRP-Ascano	54487	DGCR8
NPD_Sets	FMRP-Ascano	85379	KIAA1671
NPD_Sets	FMRP-Ascano	987	LRBA
NPD_Sets	FMRP-Ascano	120114	FAT3
NPD_Sets	FMRP-Ascano	9820	CUL7
NPD_Sets	FMRP-Ascano	285527	FRYL
NPD_Sets	FMRP-Ascano	9578	CDC42BPB
NPD_Sets	FMRP-Ascano	9181	ARHGEF2
NPD_Sets	FMRP-Ascano	166647	GPR125
NPD_Sets	FMRP-Ascano	121551	BTBD11
NPD_Sets	FMRP-Ascano	1477	CSTF1
NPD_Sets	FMRP-Ascano	23224	SYNE2
NPD_Sets	FMRP-Ascano	4216	MAP3K4
NPD_Sets	FMRP-Ascano	23180	RFTN1
NPD_Sets	FMRP-Ascano	4836	NMT1
NPD_Sets	FMRP-Ascano	5351	PLOD1
NPD_Sets	FMRP-Ascano	3609	ILF3
NPD_Sets	FMRP-Ascano	7074	TIAM1
NPD_Sets	FMRP-Ascano	2314	FLII
NPD_Sets	FMRP-Ascano	79868	ALG13
NPD_Sets	FMRP-Ascano	2887	GRB10
NPD_Sets	FMRP-Ascano	113146	AHNAK2
NPD_Sets	FMRP-Ascano	10403	NDC80
NPD_Sets	FMRP-Ascano	5253	PHF2
NPD_Sets	FMRP-Ascano	7586	ZKSCAN1

NPD_Sets	FMRP-Ascano	55153	SDAD1
NPD_Sets	FMRP-Ascano	26960	NBEA
NPD_Sets	FMRP-Ascano	5099	PCDH7
NPD_Sets	FMRP-Ascano	7436	VLDLR
NPD_Sets	FMRP-Ascano	54625	PARP14
NPD_Sets	FMRP-Ascano	23228	PLCL2
NPD_Sets	FMRP-Ascano	22982	DIP2C
NPD_Sets	FMRP-Ascano	24147	FJX1
NPD_Sets	FMRP-Ascano	63916	ELMO2
NPD_Sets	FMRP-Ascano	57703	CWC22
NPD_Sets	FMRP-Ascano	1850	DUSP8
NPD_Sets	FMRP-Ascano	10868	USP20
NPD_Sets	FMRP-Ascano	9922	IQSEC1
NPD_Sets	FMRP-Ascano	26019	UPF2
NPD_Sets	FMRP-Ascano	90075	ZNF30
NPD_Sets	FMRP-Ascano	23294	ANKS1A
NPD_Sets	FMRP-Ascano	302	ANXA2
NPD_Sets	FMRP-Ascano	6238	RRBP1
NPD_Sets	FMRP-Ascano	10112	KIF20A
NPD_Sets	FMRP-Ascano	3326	HSP90AB1
NPD_Sets	FMRP-Ascano	55552	ZNF823
NPD_Sets	FMRP-Ascano	87	ACTN1
NPD_Sets	FMRP-Ascano	27152	INTU
NPD_Sets	FMRP-Ascano	10073	SNUPN
NPD_Sets	FMRP-Ascano	1952	CELSR2
NPD_Sets	FMRP-Ascano	79631	EFTUD1
NPD_Sets	FMRP-Ascano	29888	STRN4
NPD_Sets	FMRP-Ascano	160518	DENND5B
NPD_Sets	FMRP-Ascano	84132	USP42
NPD_Sets	FMRP-Ascano	1642	DDB1
NPD_Sets	FMRP-Ascano	955	ENTPD6
NPD_Sets	FMRP-Ascano	55628	ZNF407
NPD_Sets	FMRP-Ascano	5514	PPP1R10
NPD_Sets	FMRP-Ascano	9632	SEC24C
NPD_Sets	FMRP-Ascano	182	JAG1
NPD_Sets	FMRP-Ascano	55746	NUP133
NPD_Sets	FMRP-Ascano	2132	EXT2
NPD_Sets	FMRP-Ascano	7737	RNF113A
NPD_Sets	FMRP-Ascano	90627	STARD13
NPD_Sets	FMRP-Ascano	9321	TRIP11
NPD_Sets	FMRP-Ascano	160851	DGKH
NPD_Sets	FMRP-Ascano	23355	VPS8
NPD_Sets	FMRP-Ascano	23210	JMJD6
NPD_Sets	FMRP-Ascano	27340	UTP20
NPD_Sets	FMRP-Ascano	9891	NUAK1
NPD_Sets	FMRP-Ascano	59277	NTN4
NPD_Sets	FMRP-Ascano	57118	CAMK1D
NPD_Sets	FMRP-Ascano	3837	KPNB1
NPD_Sets	FMRP-Ascano	84893	FBXO18
NPD_Sets	FMRP-Ascano	9670	IPO13
NPD_Sets	FMRP-Ascano	84895	FAM73B
NPD_Sets	FMRP-Ascano	7750	ZMYM2
NPD_Sets	FMRP-Ascano	667	DST
NPD_Sets	FMRP-Ascano	23203	PMPCA
NPD_Sets	FMRP-Ascano	545	ATR
NPD_Sets	FMRP-Ascano	9146	HGS
NPD_Sets	FMRP-Ascano	80206	FHOD3
NPD_Sets	FMRP-Ascano	140809	SRXN1

NPD_Sets	FMRP-Ascano	7511	XPNPEP1
NPD_Sets	FMRP-Ascano	5859	QARS
NPD_Sets	FMRP-Ascano	57647	DHX37
NPD_Sets	FMRP-Ascano	5255	PHKA1
NPD_Sets	FMRP-Ascano	23309	SIN3B
NPD_Sets	FMRP-Ascano	8927	BSN
NPD_Sets	FMRP-Ascano	4676	NAP1L4
NPD_Sets	FMRP-Ascano	23534	TNPO3
NPD_Sets	FMRP-Ascano	10514	MYBBP1A
NPD_Sets	FMRP-Ascano	976	CD97
NPD_Sets	FMRP-Ascano	9920	KBTBD11
NPD_Sets	FMRP-Ascano	833	CARS
NPD_Sets	FMRP-Ascano	10869	USP19
NPD_Sets	FMRP-Ascano	55636	CHD7
NPD_Sets	FMRP-Ascano	3069	HDLBP
NPD_Sets	FMRP-Ascano	4790	NFKB1
NPD_Sets	FMRP-Ascano	9330	GTF3C3
NPD_Sets	FMRP-Ascano	57062	DDX24
NPD_Sets	FMRP-Ascano	30811	HUNK
NPD_Sets	FMRP-Ascano	4041	LRP5
NPD_Sets	FMRP-Ascano	57505	AARS2
NPD_Sets	FMRP-Ascano	9968	MED12
NPD_Sets	FMRP-Ascano	23344	ESYT1
NPD_Sets	FMRP-Ascano	23654	PLXNB2
NPD_Sets	FMRP-Ascano	58504	ARHGAP22
NPD_Sets	FMRP-Ascano	23251	KIAA1024
NPD_Sets	FMRP-Ascano	54856	GON4L
NPD_Sets	FMRP-Ascano	81559	TRIM11
NPD_Sets	FMRP-Ascano	54496	PRMT7
NPD_Sets	FMRP-Ascano	147923	ZNF420
NPD_Sets	FMRP-Ascano	162	AP1B1
NPD_Sets	FMRP-Ascano	7840	ALMS1
NPD_Sets	FMRP-Ascano	23269	MGA
NPD_Sets	FMRP-Ascano	3949	LDLR
NPD_Sets	FMRP-Ascano	1616	DAXX
NPD_Sets	FMRP-Ascano	10127	ZNF263
NPD_Sets	FMRP-Ascano	23066	CAND2
NPD_Sets	FMRP-Ascano	57410	SCYL1
NPD_Sets	FMRP-Ascano	3833	KIFC1
NPD_Sets	FMRP-Ascano	9730	VPRBP
NPD_Sets	FMRP-Ascano	7175	TPR
NPD_Sets	FMRP-Ascano	8943	AP3D1
NPD_Sets	FMRP-Ascano	23510	KCTD2
NPD_Sets	FMRP-Ascano	80005	DOCK5
NPD_Sets	FMRP-Ascano	10592	SMC2
NPD_Sets	FMRP-Ascano	22883	CLSTN1
NPD_Sets	FMRP-Ascano	344558	SH3RF3
NPD_Sets	FMRP-Ascano	2580	GAK
NPD_Sets	FMRP-Ascano	4172	MCM3
NPD_Sets	FMRP-Ascano	10940	POP1
NPD_Sets	FMRP-Ascano	126070	ZNF440
NPD_Sets	FMRP-Ascano	55770	EXOC2
NPD_Sets	FMRP-Ascano	4285	MIPEP
NPD_Sets	FMRP-Ascano	1793	DOCK1
NPD_Sets	FMRP-Ascano	9895	TECPR2
NPD_Sets	FMRP-Ascano	89796	NAV1
NPD_Sets	FMRP-Ascano	321	APBA2
NPD_Sets	FMRP-Ascano	4641	MYO1C

NPD_Sets	FMRP-Ascano	7222	TRPC3
NPD_Sets	FMRP-Ascano	375449	MAST4
NPD_Sets	FMRP-Ascano	80347	COASY
NPD_Sets	FMRP-Ascano	10579	TACC2
NPD_Sets	FMRP-Ascano	55388	MCM10
NPD_Sets	FMRP-Ascano	56897	WRNIP1
NPD_Sets	FMRP-Ascano	51761	ATP8A2
NPD_Sets	FMRP-Ascano	7158	TP53BP1
NPD_Sets	FMRP-Ascano	3099	HK2
NPD_Sets	FMRP-Ascano	3915	LAMC1
NPD_Sets	FMRP-Ascano	25879	DCAF13
NPD_Sets	FMRP-Ascano	2261	FGFR3
NPD_Sets	FMRP-Ascano	91151	TIGD7
NPD_Sets	FMRP-Ascano	5209	PFKFB3
NPD_Sets	FMRP-Ascano	9905	SGSM2
NPD_Sets	FMRP-Ascano	23481	PES1
NPD_Sets	FMRP-Ascano	8458	TTF2
NPD_Sets	FMRP-Ascano	155435	RBM33
NPD_Sets	FMRP-Ascano	3309	HSPA5
NPD_Sets	FMRP-Ascano	8189	SYMPK
NPD_Sets	FMRP-Ascano	57674	RNF213
NPD_Sets	FMRP-Ascano	23236	PLCB1
NPD_Sets	FMRP-Ascano	23450	SF3B3
NPD_Sets	FMRP-Ascano	79932	KIAA0319L
NPD_Sets	FMRP-Ascano	79813	EHMT1
NPD_Sets	FMRP-Ascano	3954	LETM1
NPD_Sets	FMRP-Ascano	51479	ANKFY1
NPD_Sets	FMRP-Ascano	55226	NAT10
NPD_Sets	FMRP-Ascano	91768	CABLES1
NPD_Sets	FMRP-Ascano	55660	PRPF40A
NPD_Sets	FMRP-Ascano	4670	HNRNPM
NPD_Sets	FMRP-Ascano	51340	CRNKL1
NPD_Sets	FMRP-Ascano	8729	GBF1
NPD_Sets	FMRP-Ascano	7150	TOP1
NPD_Sets	FMRP-Ascano	7109	TRAPPC10
NPD_Sets	FMRP-Ascano	79711	IPO4
NPD_Sets	FMRP-Ascano	4236	MFAP1
NPD_Sets	FMRP-Ascano	23247	KIAA0556
NPD_Sets	FMRP-Ascano	51603	METTL13
NPD_Sets	FMRP-Ascano	6461	SHB
NPD_Sets	FMRP-Ascano	3064	HTT
NPD_Sets	FMRP-Ascano	23030	KDM4B
NPD_Sets	FMRP-Ascano	22893	BAHD1
NPD_Sets	FMRP-Ascano	23020	SNRNP200
NPD_Sets	FMRP-Ascano	26502	NARF
NPD_Sets	FMRP-Ascano	57585	CRAMP1L
NPD_Sets	FMRP-Ascano	8914	TIMELESS
NPD_Sets	FMRP-Ascano	23380	SRGAP2
NPD_Sets	FMRP-Ascano	7837	PXDN
NPD_Sets	FMRP-Ascano	83903	GSG2
NPD_Sets	FMRP-Ascano	23286	WWC1
NPD_Sets	FMRP-Ascano	10061	ABCF2
NPD_Sets	FMRP-Ascano	1376	CPT2
NPD_Sets	FMRP-Ascano	23279	NUP160
NPD_Sets	FMRP-Ascano	2975	GTF3C1
NPD_Sets	FMRP-Ascano	79026	AHNAK
NPD_Sets	FMRP-Ascano	309	ANXA6
NPD_Sets	FMRP-Ascano	9700	ESPL1

NPD_Sets	FMRP-Ascano	6830	SUPT6H
NPD_Sets	FMRP-Ascano	3708	ITPR1
NPD_Sets	FMRP-Ascano	54535	CCHCR1
NPD_Sets	FMRP-Ascano	23348	DOCK9
NPD_Sets	FMRP-Ascano	84154	RPF2
NPD_Sets	FMRP-Ascano	7184	HSP90B1
NPD_Sets	FMRP-Ascano	23151	GRAMD4
NPD_Sets	FMRP-Ascano	11188	NISCH
NPD_Sets	FMRP-Ascano	7174	TPP2
NPD_Sets	FMRP-Ascano	7517	XRCC3
NPD_Sets	FMRP-Ascano	23239	PHLPP1
NPD_Sets	FMRP-Ascano	47	ACLY
NPD_Sets	FMRP-Ascano	9414	TJP2
NPD_Sets	FMRP-Ascano	1778	DYNC1H1
NPD_Sets	FMRP-Ascano	661	POLR3D
NPD_Sets	FMRP-Ascano	5783	PTPN13
NPD_Sets	FMRP-Ascano	10482	NXF1
NPD_Sets	FMRP-Ascano	57568	SIPA1L2
NPD_Sets	FMRP-Ascano	5297	PI4KA
NPD_Sets	FMRP-Ascano	4920	ROR2
NPD_Sets	FMRP-Ascano	154796	AMOT
NPD_Sets	FMRP-Ascano	4645	MYO5B
NPD_Sets	FMRP-Ascano	9924	PAN2
NPD_Sets	FMRP-Ascano	8518	IKBKAP
NPD_Sets	FMRP-Ascano	84059	GPR98
NPD_Sets	FMRP-Ascano	10497	UNC13B
NPD_Sets	FMRP-Ascano	57713	SFMBT2
NPD_Sets	FMRP-Ascano	64215	DNAJC1
NPD_Sets	FMRP-Ascano	57619	SHROOM3
NPD_Sets	FMRP-Ascano	57221	KIAA1244
NPD_Sets	FMRP-Ascano	83746	L3MBTL2
NPD_Sets	FMRP-Ascano	7566	ZNF18
NPD_Sets	FMRP-Ascano	57513	CASKIN2
NPD_Sets	FMRP-Ascano	4038	LRP4
NPD_Sets	FMRP-Ascano	23400	ATP13A2
NPD_Sets	FMRP-Ascano	9875	URB1
NPD_Sets	FMRP-Ascano	10939	AFG3L2
NPD_Sets	FMRP-Ascano	51196	PLCE1
NPD_Sets	FMRP-Ascano	55661	DDX27
NPD_Sets	FMRP-Ascano	161	AP2A2
NPD_Sets	FMRP-Ascano	1462	VCAN
NPD_Sets	FMRP-Ascano	9682	KDM4A
NPD_Sets	FMRP-Ascano	7153	TOP2A
NPD_Sets	FMRP-Ascano	80222	TARS2
NPD_Sets	FMRP-Ascano	4217	MAP3K5
NPD_Sets	FMRP-Ascano	5190	PEX6
NPD_Sets	FMRP-Ascano	8295	TRRAP
NPD_Sets	FMRP-Ascano	64718	UNKL
NPD_Sets	FMRP-Ascano	8632	DNAH17
NPD_Sets	FMRP-Ascano	23049	SMG1
NPD_Sets	FMRP-Ascano	57169	ZNFX1
NPD_Sets	FMRP-Ascano	84079	ANKRD27
NPD_Sets	FMRP-Ascano	3643	INSR
NPD_Sets	FMRP-Ascano	27000	DNAJC2
NPD_Sets	FMRP-Ascano	5394	EXOSC10
NPD_Sets	FMRP-Ascano	121227	LRIG3
NPD_Sets	FMRP-Ascano	91133	L3MBTL4
NPD_Sets	FMRP-Ascano	55837	EAPP

NPD_Sets	FMRP-Ascano	7249	TSC2
NPD_Sets	FMRP-Ascano	7543	ZFX
NPD_Sets	FMRP-Ascano	9508	ADAMTS3
NPD_Sets	FMRP-Ascano	79980	DSN1
NPD_Sets	FMRP-Ascano	8886	DDX18
NPD_Sets	FMRP-Ascano	54520	CCDC93
NPD_Sets	FMRP-Ascano	30836	DNTTIP2
NPD_Sets	FMRP-Ascano	10594	PRPF8
NPD_Sets	FMRP-Ascano	90441	ZNF622
NPD_Sets	FMRP-Ascano	1786	DNMT1
NPD_Sets	FMRP-Ascano	55665	URGCP
NPD_Sets	FMRP-Ascano	23302	WSCD1
NPD_Sets	FMRP-Ascano	51512	GTSE1
NPD_Sets	FMRP-Ascano	11004	KIF2C
NPD_Sets	FMRP-Ascano	8662	EIF3B
NPD_Sets	FMRP-Ascano	8897	MTMR3
NPD_Sets	FMRP-Ascano	5707	PSMD1
NPD_Sets	FMRP-Ascano	25778	DSTYK
NPD_Sets	FMRP-Ascano	10395	DLC1
NPD_Sets	FMRP-Ascano	56288	PARD3
NPD_Sets	FMRP-Ascano	56949	XAB2
NPD_Sets	FMRP-Ascano	56259	CTNNBL1
NPD_Sets	FMRP-Ascano	29803	REPIN1
NPD_Sets	FMRP-Ascano	334	APLP2
NPD_Sets	FMRP-Ascano	57617	VPS18
NPD_Sets	FMRP-Ascano	26999	CYFIP2
NPD_Sets	FMRP-Ascano	63977	PRDM15
NPD_Sets	FMRP-Ascano	29894	CPSF1
NPD_Sets	FMRP-Ascano	56967	C14orf132
NPD_Sets	FMRP-Ascano	55201	MAP1S
NPD_Sets	FMRP-Ascano	55703	POLR3B
NPD_Sets	FMRP-Ascano	63892	THADA
NPD_Sets	FMRP-Ascano	3716	JAK1
NPD_Sets	FMRP-Ascano	30849	PIK3R4
NPD_Sets	FMRP-Ascano	5426	POLE
NPD_Sets	FMRP-Ascano	8242	KDM5C
NPD_Sets	FMRP-Ascano	9371	KIF3B
NPD_Sets	FMRP-Ascano	4967	OGDH
NPD_Sets	FMRP-Ascano	1495	CTNNA1
NPD_Sets	FMRP-Ascano	6117	RPA1
NPD_Sets	FMRP-Ascano	23370	ARHGEF18
NPD_Sets	FMRP-Ascano	23140	ZZEF1
NPD_Sets	FMRP-Ascano	54497	HEATR5B
NPD_Sets	FMRP-Ascano	51594	NBAS
NPD_Sets	FMRP-Ascano	7415	VCP
NPD_Sets	FMRP-Ascano	23395	LARS2
NPD_Sets	FMRP-Ascano	2734	GLG1
NPD_Sets	FMRP-Ascano	3912	LAMB1
NPD_Sets	FMRP-Ascano	25894	PLEKHG4
NPD_Sets	FMRP-Ascano	8894	EIF2S2
NPD_Sets	FMRP-Ascano	84181	CHD6
NPD_Sets	FMRP-Ascano	7343	UBTF
NPD_Sets	FMRP-Ascano	26230	TIAM2
NPD_Sets	FMRP-Ascano	22853	LMTK2
NPD_Sets	FMRP-Ascano	4650	MYO9B
NPD_Sets	FMRP-Ascano	9265	CYTH3
NPD_Sets	FMRP-Ascano	145567	TTC7B
NPD_Sets	FMRP-Ascano	64135	IFIH1

NPD_Sets	FMRP-Ascano	3678	ITGA5
NPD_Sets	FMRP-Ascano	89887	ZNF628
NPD_Sets	FMRP-Ascano	29072	SETD2
NPD_Sets	FMRP-Ascano	1951	CELSR3
NPD_Sets	FMRP-Ascano	26018	LRIG1
NPD_Sets	FMRP-Ascano	84733	CBX2
NPD_Sets	FMRP-Ascano	55698	RADIL
NPD_Sets	FMRP-Ascano	23005	MAPKBP1
NPD_Sets	FMRP-Ascano	25973	PARS2
NPD_Sets	FMRP-Ascano	5256	PHKA2
NPD_Sets	FMRP-Ascano	10460	TACC3
NPD_Sets	FMRP-Ascano	8312	AXIN1
NPD_Sets	FMRP-Ascano	9869	SETDB1
NPD_Sets	FMRP-Ascano	25929	GEMIN5
NPD_Sets	FMRP-Ascano	23677	SH3BP4
NPD_Sets	FMRP-Ascano	9688	NUP93
NPD_Sets	FMRP-Ascano	3710	ITPR3
NPD_Sets	FMRP-Ascano	23158	TBC1D9
NPD_Sets	FMRP-Ascano	135114	HINT3
NPD_Sets	FMRP-Ascano	23523	CABIN1
NPD_Sets	FMRP-Ascano	124842	TMEM132E
NPD_Sets	FMRP-Ascano	24148	PRPF6
NPD_Sets	FMRP-Ascano	5708	PSMD2
NPD_Sets	FMRP-Ascano	55341	LSG1
NPD_Sets	FMRP-Ascano	5836	PYGL
NPD_Sets	FMRP-Ascano	221002	RASGEF1A
NPD_Sets	FMRP-Ascano	23644	EDC4
NPD_Sets	FMRP-Ascano	157922	CAMSAP1
NPD_Sets	FMRP-Ascano	63898	SH2D4A
NPD_Sets	FMRP-Ascano	25885	POLR1A
NPD_Sets	FMRP-Ascano	9129	PRPF3
NPD_Sets	FMRP-Ascano	2195	FAT1
NPD_Sets	FMRP-Ascano	114787	GPRIN1
NPD_Sets	FMRP-Ascano	22880	MORC2
NPD_Sets	FMRP-Ascano	7862	BRPF1
NPD_Sets	FMRP-Ascano	6712	SPTBN2
NPD_Sets	FMRP-Ascano	79149	ZSCAN5A
NPD_Sets	FMRP-Ascano	63976	PRDM16
NPD_Sets	FMRP-Ascano	10615	SPAG5
NPD_Sets	FMRP-Ascano	9967	THRAP3
NPD_Sets	FMRP-Ascano	23178	PASK
NPD_Sets	FMRP-Ascano	8559	PRPF18
NPD_Sets	FMRP-Ascano	8563	THOC5
NPD_Sets	FMRP-Ascano	8925	HERC1
NPD_Sets	FMRP-Ascano	26524	LATS2
NPD_Sets	FMRP-Ascano	3709	ITPR2
NPD_Sets	FMRP-Ascano	144100	PLEKHA7
NPD_Sets	FMRP-Ascano	5976	UPF1
NPD_Sets	FMRP-Ascano	27079	RPUSD2
NPD_Sets	FMRP-Ascano	5335	PLCG1
NPD_Sets	FMRP-Ascano	29890	RBM15B
NPD_Sets	FMRP-Ascano	790	CAD
NPD_Sets	FMRP-Ascano	84164	ASCC2
NPD_Sets	FMRP-Ascano	3482	IGF2R
NPD_Sets	FMRP-Ascano	93624	TADA2B
NPD_Sets	FMRP-Ascano	7701	ZNF142
NPD_Sets	FMRP-Ascano	23195	MDN1
NPD_Sets	FMRP-Ascano	2316	FLNA

NPD_Sets	FMRP-Ascano	4288	MKI67
NPD_Sets	FMRP-Ascano	9918	NCAPD2
NPD_Sets	FMRP-Ascano	55229	PANK4
NPD_Sets	FMRP-Ascano	5795	PTPRJ
NPD_Sets	FMRP-Ascano	1659	DHX8
NPD_Sets	FMRP-Ascano	9816	URB2
NPD_Sets	FMRP-Ascano	9513	FXR2
NPD_Sets	FMRP-Ascano	16	AARS
NPD_Sets	FMRP-Ascano	57683	ZDBF2
NPD_Sets	FMRP-Ascano	287	ANK2
NPD_Sets	FMRP-Ascano	65123	INTS3
NPD_Sets	FMRP-Ascano	3831	KLC1
NPD_Sets	FMRP-Ascano	23242	COBL
NPD_Sets	FMRP-Ascano	57680	CHD8
NPD_Sets	FMRP-Ascano	23503	ZFYVE26
NPD_Sets	FMRP-Ascano	50628	GEMIN4
NPD_Sets	FMRP-Ascano	4642	MYO1D
NPD_Sets	FMRP-Ascano	839	CASP6
NPD_Sets	FMRP-Ascano	9620	CELSR1
NPD_Sets	FMRP-Ascano	161725	OTUD7A
NPD_Sets	FMRP-Ascano	4628	MYH10
NPD_Sets	FMRP-Ascano	5550	PREP
NPD_Sets	FMRP-Ascano	57510	XPO5
NPD_Sets	FMRP-Ascano	9343	EFTUD2
NPD_Sets	FMRP-Ascano	80271	ITPKC
NPD_Sets	FMRP-Ascano	55755	CDK5RAP2
NPD_Sets	FMRP-Ascano	55526	DHTKD1
NPD_Sets	FMRP-Ascano	1662	DDX10
NPD_Sets	FMRP-Ascano	8241	RBM10
NPD_Sets	FMRP-Ascano	8924	HERC2
NPD_Sets	FMRP-Ascano	23513	SCRIB
NPD_Sets	FMRP-Ascano	4036	LRP2
NPD_Sets	FMRP-Ascano	24144	TFIP11
NPD_Sets	FMRP-Ascano	9903	KLHL21
NPD_Sets	FMRP-Ascano	341640	FREM2
NPD_Sets	FMRP-Ascano	57661	PHRF1
NPD_Sets	FMRP-Ascano	26958	COPG2
NPD_Sets	FMRP-Ascano	23774	BRD1
NPD_Sets	FMRP-Ascano	23328	SASH1
NPD_Sets	FMRP-Ascano	63967	CLSPN
NPD_Sets	FMRP-Ascano	6594	SMARCA1
NPD_Sets	FMRP-Ascano	9416	DDX23
NPD_Sets	FMRP-Ascano	2201	FBN2
NPD_Sets	FMRP-Ascano	10908	PNPLA6
NPD_Sets	FMRP-Ascano	2200	FBN1
NPD_Sets	FMRP-Ascano	9790	BMS1
NPD_Sets	FMRP-Ascano	50485	SMARCAL1
NPD_Sets	FMRP-Ascano	51191	HERC5
NPD_Sets	FMRP-Ascano	1108	CHD4
NPD_Sets	FMRP-Ascano	26024	PTCD1
NPD_Sets	FMRP-Ascano	11128	POLR3A
NPD_Sets	FMRP-Ascano	7204	TRIO
NPD_Sets	FMRP-Ascano	6262	RYR2
NPD_Sets	FMRP-Ascano	23392	KIAA0368
NPD_Sets	FMRP-Ascano	79925	SPEF2
NPD_Sets	FMRP-Ascano	23130	ATG2A
NPD_Sets	FMRP-Ascano	57728	WDR19
NPD_Sets	FMRP-Ascano	29889	GNL2

NPD_Sets	FMRP-Ascano	9919	SEC16A
NPD_Sets	FMRP-Ascano	8642	DCHS1
NPD_Sets	FMRP-Ascano	2317	FLNB
NPD_Sets	FMRP-Ascano	1660	DHX9
NPD_Sets	FMRP-Ascano	10985	GCN1L1
NPD_Sets	FMRP-Ascano	116236	ABHD15
NPD_Sets	FMRP-Ascano	6709	SPTAN1
NPD_Sets	FMRP-Ascano	11325	DDX42
NPD_Sets	FMRP-Ascano	9231	DLG5
NPD_Sets	FMRP-Ascano	22984	PDCD11
NPD_Sets	FMRP-Ascano	2071	ERCC3
NPD_Sets	FMRP-Ascano	7094	TLN1
NPD_Sets	FMRP-Ascano	7290	HIRA
NPD_Sets	FMRP-Ascano	2475	MTOR
NPD_Sets	FMRP-Ascano	83481	EPPK1
NPD_Sets	FMRP-Ascano	7644	ZNF91
NPD_Sets	FMRP-Darnell	8927	BSN
NPD_Sets	FMRP-Darnell	547	KIF1A
NPD_Sets	FMRP-Darnell	4130	MAP1A
NPD_Sets	FMRP-Darnell	324	APC
NPD_Sets	FMRP-Darnell	4131	MAP1B
NPD_Sets	FMRP-Darnell	116986	AGAP2
NPD_Sets	FMRP-Darnell	107	ADCY1
NPD_Sets	FMRP-Darnell	22859	LPHN1
NPD_Sets	FMRP-Darnell	26999	CYFIP2
NPD_Sets	FMRP-Darnell	491	ATP2B2
NPD_Sets	FMRP-Darnell	11188	NISCH
NPD_Sets	FMRP-Darnell	23095	KIF1B
NPD_Sets	FMRP-Darnell	5802	PTPRS
NPD_Sets	FMRP-Darnell	50944	SHANK1
NPD_Sets	FMRP-Darnell	10075	HUWE1
NPD_Sets	FMRP-Darnell	3798	KIF5A
NPD_Sets	FMRP-Darnell	576	BAI2
NPD_Sets	FMRP-Darnell	8831	SYNGAP1
NPD_Sets	FMRP-Darnell	478	ATP1A3
NPD_Sets	FMRP-Darnell	2194	FASN
NPD_Sets	FMRP-Darnell	23001	WDFY3
NPD_Sets	FMRP-Darnell	5138	PDE2A
NPD_Sets	FMRP-Darnell	1107	CHD3
NPD_Sets	FMRP-Darnell	287	ANK2
NPD_Sets	FMRP-Darnell	3708	ITPR1
NPD_Sets	FMRP-Darnell	23154	NCDN
NPD_Sets	FMRP-Darnell	3097	HIVEP2
NPD_Sets	FMRP-Darnell	7267	TTC3
NPD_Sets	FMRP-Darnell	3064	HTT
NPD_Sets	FMRP-Darnell	9625	AATK
NPD_Sets	FMRP-Darnell	9743	ARHGAP32
NPD_Sets	FMRP-Darnell	22853	LMTK2
NPD_Sets	FMRP-Darnell	1952	CELSR2
NPD_Sets	FMRP-Darnell	815	CAMK2A
NPD_Sets	FMRP-Darnell	1501	CTNND2
NPD_Sets	FMRP-Darnell	1938	EEF2
NPD_Sets	FMRP-Darnell	1759	DNM1
NPD_Sets	FMRP-Darnell	57555	NLGN2
NPD_Sets	FMRP-Darnell	6712	SPTBN2
NPD_Sets	FMRP-Darnell	27445	PCLO
NPD_Sets	FMRP-Darnell	2904	GRIN2B
NPD_Sets	FMRP-Darnell	89795	NAV3

NPD_Sets	FMRP-Darnell	4035	LRP1
NPD_Sets	FMRP-Darnell	57580	PREX1
NPD_Sets	FMRP-Darnell	9379	NRXN2
NPD_Sets	FMRP-Darnell	10297	APC2
NPD_Sets	FMRP-Darnell	1742	DLG4
NPD_Sets	FMRP-Darnell	4763	NF1
NPD_Sets	FMRP-Darnell	23125	CAMTA2
NPD_Sets	FMRP-Darnell	288	ANK3
NPD_Sets	FMRP-Darnell	29	ABR
NPD_Sets	FMRP-Darnell	92154	MTSS1L
NPD_Sets	FMRP-Darnell	84894	LINGO1
NPD_Sets	FMRP-Darnell	2901	GRIK5
NPD_Sets	FMRP-Darnell	23077	MYCBP2
NPD_Sets	FMRP-Darnell	10129	FRY
NPD_Sets	FMRP-Darnell	4133	MAP2
NPD_Sets	FMRP-Darnell	9612	NCOR2
NPD_Sets	FMRP-Darnell	20	ABCA2
NPD_Sets	FMRP-Darnell	6305	SBF1
NPD_Sets	FMRP-Darnell	8567	MADD
NPD_Sets	FMRP-Darnell	8911	CACNA1I
NPD_Sets	FMRP-Darnell	22990	PCNX
NPD_Sets	FMRP-Darnell	3800	KIF5C
NPD_Sets	FMRP-Darnell	4644	MYO5A
NPD_Sets	FMRP-Darnell	9472	AKAP6
NPD_Sets	FMRP-Darnell	57030	SLC17A7
NPD_Sets	FMRP-Darnell	8525	DGKZ
NPD_Sets	FMRP-Darnell	23258	DENND5A
NPD_Sets	FMRP-Darnell	8924	HERC2
NPD_Sets	FMRP-Darnell	1778	DYNC1H1
NPD_Sets	FMRP-Darnell	8502	PKP4
NPD_Sets	FMRP-Darnell	667	DST
NPD_Sets	FMRP-Darnell	575	BAI1
NPD_Sets	FMRP-Darnell	57605	PITPNM2
NPD_Sets	FMRP-Darnell	5297	PI4KA
NPD_Sets	FMRP-Darnell	6709	SPTAN1
NPD_Sets	FMRP-Darnell	23524	SRRM2
NPD_Sets	FMRP-Darnell	1387	CREBBP
NPD_Sets	FMRP-Darnell	26960	NBEA
NPD_Sets	FMRP-Darnell	506	ATP5B
NPD_Sets	FMRP-Darnell	80725	SRCIN1
NPD_Sets	FMRP-Darnell	4967	OGDH
NPD_Sets	FMRP-Darnell	9229	DLGAP1
NPD_Sets	FMRP-Darnell	23046	KIF21B
NPD_Sets	FMRP-Darnell	23400	ATP13A2
NPD_Sets	FMRP-Darnell	6334	SCN8A
NPD_Sets	FMRP-Darnell	23162	MAPK8IP3
NPD_Sets	FMRP-Darnell	65268	WNK2
NPD_Sets	FMRP-Darnell	83637	ZMIZ2
NPD_Sets	FMRP-Darnell	146330	FBXL16
NPD_Sets	FMRP-Darnell	8295	TRRAP
NPD_Sets	FMRP-Darnell	6711	SPTBN1
NPD_Sets	FMRP-Darnell	23061	TBC1D9B
NPD_Sets	FMRP-Darnell	6651	SON
NPD_Sets	FMRP-Darnell	5361	PLXNA1
NPD_Sets	FMRP-Darnell	58512	DLGAP3
NPD_Sets	FMRP-Darnell	10188	TNK2
NPD_Sets	FMRP-Darnell	2909	ARHGAP35
NPD_Sets	FMRP-Darnell	23164	MPRIP

NPD_Sets	FMRP-Darnell	6721	SREBF2
NPD_Sets	FMRP-Darnell	26038	CHD5
NPD_Sets	FMRP-Darnell	23129	PLXND1
NPD_Sets	FMRP-Darnell	9479	MAPK8IP1
NPD_Sets	FMRP-Darnell	9645	MICAL2
NPD_Sets	FMRP-Darnell	5581	PRKCE
NPD_Sets	FMRP-Darnell	6497	SKI
NPD_Sets	FMRP-Darnell	65125	WNK1
NPD_Sets	FMRP-Darnell	23025	UNC13A
NPD_Sets	FMRP-Darnell	22980	TCF25
NPD_Sets	FMRP-Darnell	1982	EIF4G2
NPD_Sets	FMRP-Darnell	389813	C9orf172
NPD_Sets	FMRP-Darnell	5522	PPP2R2C
NPD_Sets	FMRP-Darnell	9901	SRGAP3
NPD_Sets	FMRP-Darnell	2903	GRIN2A
NPD_Sets	FMRP-Darnell	23152	CIC
NPD_Sets	FMRP-Darnell	9568	GABBR2
NPD_Sets	FMRP-Darnell	6812	STXBP1
NPD_Sets	FMRP-Darnell	9611	NCOR1
NPD_Sets	FMRP-Darnell	23261	CAMTA1
NPD_Sets	FMRP-Darnell	5923	RASGRF1
NPD_Sets	FMRP-Darnell	5127	CDK16
NPD_Sets	FMRP-Darnell	3054	HCFC1
NPD_Sets	FMRP-Darnell	22895	RPH3A
NPD_Sets	FMRP-Darnell	773	CACNA1A
NPD_Sets	FMRP-Darnell	10079	ATP9A
NPD_Sets	FMRP-Darnell	9980	DOPEY2
NPD_Sets	FMRP-Darnell	8997	KALRN
NPD_Sets	FMRP-Darnell	23122	CLASP2
NPD_Sets	FMRP-Darnell	115703	ARHGAP33
NPD_Sets	FMRP-Darnell	8925	HERC1
NPD_Sets	FMRP-Darnell	5178	PEG3
NPD_Sets	FMRP-Darnell	80309	SPHKAP
NPD_Sets	FMRP-Darnell	85358	SHANK3
NPD_Sets	FMRP-Darnell	1740	DLG2
NPD_Sets	FMRP-Darnell	55187	VPS13D
NPD_Sets	FMRP-Darnell	9828	ARHGEF17
NPD_Sets	FMRP-Darnell	9659	PDE4DIP
NPD_Sets	FMRP-Darnell	1499	CTNNB1
NPD_Sets	FMRP-Darnell	7204	TRIO
NPD_Sets	FMRP-Darnell	83660	TLN2
NPD_Sets	FMRP-Darnell	50488	MINK1
NPD_Sets	FMRP-Darnell	9892	SNAP91
NPD_Sets	FMRP-Darnell	23389	MED13L
NPD_Sets	FMRP-Darnell	9600	PITPNM1
NPD_Sets	FMRP-Darnell	23395	LARS2
NPD_Sets	FMRP-Darnell	64324	NSD1
NPD_Sets	FMRP-Darnell	2782	GNB1
NPD_Sets	FMRP-Darnell	9578	CDC42BPB
NPD_Sets	FMRP-Darnell	2196	FAT2
NPD_Sets	FMRP-Darnell	440279	UNC13C
NPD_Sets	FMRP-Darnell	9736	USP34
NPD_Sets	FMRP-Darnell	488	ATP2A2
NPD_Sets	FMRP-Darnell	23040	MYT1L
NPD_Sets	FMRP-Darnell	84446	BRSK1
NPD_Sets	FMRP-Darnell	6252	RTN1
NPD_Sets	FMRP-Darnell	399687	MYO18A
NPD_Sets	FMRP-Darnell	57584	ARHGAP21

NPD_Sets	FMRP-Darnell	816	CAMK2B
NPD_Sets	FMRP-Darnell	23348	DOCK9
NPD_Sets	FMRP-Darnell	204851	HIPK1
NPD_Sets	FMRP-Darnell	8941	CDK5R2
NPD_Sets	FMRP-Darnell	84461	NEURL4
NPD_Sets	FMRP-Darnell	5799	PTPRN2
NPD_Sets	FMRP-Darnell	5792	PTPRF
NPD_Sets	FMRP-Darnell	89796	NAV1
NPD_Sets	FMRP-Darnell	1639	DCTN1
NPD_Sets	FMRP-Darnell	161	AP2A2
NPD_Sets	FMRP-Darnell	1808	DPYSL2
NPD_Sets	FMRP-Darnell	23109	DDN
NPD_Sets	FMRP-Darnell	4591	TRIM37
NPD_Sets	FMRP-Darnell	23312	DMXL2
NPD_Sets	FMRP-Darnell	51195	RAPGEFL1
NPD_Sets	FMRP-Darnell	8239	USP9X
NPD_Sets	FMRP-Darnell	130507	UBR3
NPD_Sets	FMRP-Darnell	22883	CLSTN1
NPD_Sets	FMRP-Darnell	23196	FAM120A
NPD_Sets	FMRP-Darnell	22937	SCAP
NPD_Sets	FMRP-Darnell	58506	SCAF1
NPD_Sets	FMRP-Darnell	23096	IQSEC2
NPD_Sets	FMRP-Darnell	23396	PIP5K1C
NPD_Sets	FMRP-Darnell	6597	SMARCA4
NPD_Sets	FMRP-Darnell	57575	PCDH10
NPD_Sets	FMRP-Darnell	65009	NDRG4
NPD_Sets	FMRP-Darnell	10125	RASGRP1
NPD_Sets	FMRP-Darnell	114088	TRIM9
NPD_Sets	FMRP-Darnell	111	ADCY5
NPD_Sets	FMRP-Darnell	5579	PRKCB
NPD_Sets	FMRP-Darnell	7074	TIAM1
NPD_Sets	FMRP-Darnell	747	DAGLA
NPD_Sets	FMRP-Darnell	25999	CLIP3
NPD_Sets	FMRP-Darnell	6653	SORL1
NPD_Sets	FMRP-Darnell	30000	TNPO2
NPD_Sets	FMRP-Darnell	23365	ARHGEF12
NPD_Sets	FMRP-Darnell	23274	CLEC16A
NPD_Sets	FMRP-Darnell	22839	DLGAP4
NPD_Sets	FMRP-Darnell	333	APLP1
NPD_Sets	FMRP-Darnell	9853	RUSC2
NPD_Sets	FMRP-Darnell	7067	THRA
NPD_Sets	FMRP-Darnell	56977	STOX2
NPD_Sets	FMRP-Darnell	10439	OLFM1
NPD_Sets	FMRP-Darnell	160	AP2A1
NPD_Sets	FMRP-Darnell	26037	SIPA1L1
NPD_Sets	FMRP-Darnell	399909	PCNXL3
NPD_Sets	FMRP-Darnell	7109	TRAPPC10
NPD_Sets	FMRP-Darnell	477	ATP1A2
NPD_Sets	FMRP-Darnell	23158	TBC1D9
NPD_Sets	FMRP-Darnell	57524	CASKIN1
NPD_Sets	FMRP-Darnell	5362	PLXNA2
NPD_Sets	FMRP-Darnell	84630	TTBK1
NPD_Sets	FMRP-Darnell	120114	FAT3
NPD_Sets	FMRP-Darnell	3326	HSP90AB1
NPD_Sets	FMRP-Darnell	777	CACNA1E
NPD_Sets	FMRP-Darnell	50	ACO2
NPD_Sets	FMRP-Darnell	57479	PRR12
NPD_Sets	FMRP-Darnell	26115	TANC2

NPD_Sets	FMRP-Darnell	23345	SYNE1
NPD_Sets	FMRP-Darnell	23130	ATG2A
NPD_Sets	FMRP-Darnell	22983	MAST1
NPD_Sets	FMRP-Darnell	23214	XPO6
NPD_Sets	FMRP-Darnell	55700	MAP7D1
NPD_Sets	FMRP-Darnell	60	ACTB
NPD_Sets	FMRP-Darnell	11122	PTPRT
NPD_Sets	FMRP-Darnell	8913	CACNA1G
NPD_Sets	FMRP-Darnell	1795	DOCK3
NPD_Sets	FMRP-Darnell	9728	SECISBP2L
NPD_Sets	FMRP-Darnell	6601	SMARCC2
NPD_Sets	FMRP-Darnell	28964	GIT1
NPD_Sets	FMRP-Darnell	9829	DNAJC6
NPD_Sets	FMRP-Darnell	27245	AHDC1
NPD_Sets	FMRP-Darnell	1213	CLTC
NPD_Sets	FMRP-Darnell	23139	MAST2
NPD_Sets	FMRP-Darnell	10594	PRPF8
NPD_Sets	FMRP-Darnell	23466	CBX6
NPD_Sets	FMRP-Darnell	89910	UBE3B
NPD_Sets	FMRP-Darnell	22848	AAK1
NPD_Sets	FMRP-Darnell	57178	ZMIZ1
NPD_Sets	FMRP-Darnell	9201	DCLK1
NPD_Sets	FMRP-Darnell	23287	AGTPBP1
NPD_Sets	FMRP-Darnell	3831	KLC1
NPD_Sets	FMRP-Darnell	23332	CLASP1
NPD_Sets	FMRP-Darnell	3745	KCNB1
NPD_Sets	FMRP-Darnell	1108	CHD4
NPD_Sets	FMRP-Darnell	6595	SMARCA2
NPD_Sets	FMRP-Darnell	6853	SYN1
NPD_Sets	FMRP-Darnell	57062	DDX24
NPD_Sets	FMRP-Darnell	23504	RIMBP2
NPD_Sets	FMRP-Darnell	23499	MACF1
NPD_Sets	FMRP-Darnell	66008	TRAK2
NPD_Sets	FMRP-Darnell	22906	TRAK1
NPD_Sets	FMRP-Darnell	29123	ANKRD11
NPD_Sets	FMRP-Darnell	6453	ITSN1
NPD_Sets	FMRP-Darnell	2137	EXTL3
NPD_Sets	FMRP-Darnell	85455	DISP2
NPD_Sets	FMRP-Darnell	2915	GRM5
NPD_Sets	FMRP-Darnell	1822	ATN1
NPD_Sets	FMRP-Darnell	613	BCR
NPD_Sets	FMRP-Darnell	56134	PCDHAC2
NPD_Sets	FMRP-Darnell	57348	TTYH1
NPD_Sets	FMRP-Darnell	84629	TNRC18
NPD_Sets	FMRP-Darnell	3778	KCNMA1
NPD_Sets	FMRP-Darnell	9969	MED13
NPD_Sets	FMRP-Darnell	51663	ZFR
NPD_Sets	FMRP-Darnell	9670	IPO13
NPD_Sets	FMRP-Darnell	4915	NTRK2
NPD_Sets	FMRP-Darnell	6857	SYT1
NPD_Sets	FMRP-Darnell	9435	CHST2
NPD_Sets	FMRP-Darnell	23518	R3HDM1
NPD_Sets	FMRP-Darnell	6262	RYR2
NPD_Sets	FMRP-Darnell	57448	BIRC6
NPD_Sets	FMRP-Darnell	23181	DIP2A
NPD_Sets	FMRP-Darnell	22997	IGSF9B
NPD_Sets	FMRP-Darnell	8874	ARHGEF7
NPD_Sets	FMRP-Darnell	2775	GNAO1

NPD_Sets	FMRP-Darnell	146057	TTBK2
NPD_Sets	FMRP-Darnell	5662	PSD
NPD_Sets	FMRP-Darnell	8476	CDC42BPA
NPD_Sets	FMRP-Darnell	4122	MAN2A2
NPD_Sets	FMRP-Darnell	4734	NEDD4
NPD_Sets	FMRP-Darnell	1981	EIF4G1
NPD_Sets	FMRP-Darnell	56924	PAK6
NPD_Sets	FMRP-Darnell	5413	SEPT5
NPD_Sets	FMRP-Darnell	7317	UBA1
NPD_Sets	FMRP-Darnell	57568	SIPA1L2
NPD_Sets	FMRP-Darnell	23037	PDZD2
NPD_Sets	FMRP-Darnell	25791	NGEF
NPD_Sets	FMRP-Darnell	6319	SCD
NPD_Sets	FMRP-Darnell	57476	GRAMD1B
NPD_Sets	FMRP-Darnell	2774	GNAL
NPD_Sets	FMRP-Darnell	3631	INPP4A
NPD_Sets	FMRP-Darnell	153090	DAB2IP
NPD_Sets	FMRP-Darnell	94030	LRRC4B
NPD_Sets	FMRP-Darnell	4134	MAP4
NPD_Sets	FMRP-Darnell	9500	MAGED1
NPD_Sets	FMRP-Darnell	23507	LRRC8B
NPD_Sets	FMRP-Darnell	283373	ANKRD52
NPD_Sets	FMRP-Darnell	9732	DOCK4
NPD_Sets	FMRP-Darnell	9066	SYT7
NPD_Sets	FMRP-Darnell	2195	FAT1
NPD_Sets	FMRP-Darnell	85442	KNDC1
NPD_Sets	FMRP-Darnell	26173	INTS1
NPD_Sets	FMRP-Darnell	26053	AUTS2
NPD_Sets	FMRP-Darnell	8848	TSC22D1
NPD_Sets	FMRP-Darnell	23174	ZCCHC14
NPD_Sets	FMRP-Darnell	25769	SLC24A2
NPD_Sets	FMRP-Darnell	2778	GNAS
NPD_Sets	FMRP-Darnell	3340	NDST1
NPD_Sets	FMRP-Darnell	57142	RTN4
NPD_Sets	FMRP-Darnell	1463	NCAN
NPD_Sets	FMRP-Darnell	2550	GABBR1
NPD_Sets	FMRP-Darnell	3756	KCNH1
NPD_Sets	FMRP-Darnell	2475	MTOR
NPD_Sets	FMRP-Darnell	9448	MAP4K4
NPD_Sets	FMRP-Darnell	79739	TTLL7
NPD_Sets	FMRP-Darnell	9899	SV2B
NPD_Sets	FMRP-Darnell	8507	ENC1
NPD_Sets	FMRP-Darnell	29924	EPN1
NPD_Sets	FMRP-Darnell	8408	ULK1
NPD_Sets	FMRP-Darnell	4784	NFIX
NPD_Sets	FMRP-Darnell	9344	TAOK2
NPD_Sets	FMRP-Darnell	59269	HIVEP3
NPD_Sets	FMRP-Darnell	57496	MKL2
NPD_Sets	FMRP-Darnell	10777	ARPP21
NPD_Sets	FMRP-Darnell	55870	ASH1L
NPD_Sets	FMRP-Darnell	9256	BZRAP1
NPD_Sets	FMRP-Darnell	80727	TTYH3
NPD_Sets	FMRP-Darnell	2752	GLUL
NPD_Sets	FMRP-Darnell	10985	GCN1L1
NPD_Sets	FMRP-Darnell	1850	DUSP8
NPD_Sets	FMRP-Darnell	8729	GBF1
NPD_Sets	FMRP-Darnell	23049	SMG1
NPD_Sets	FMRP-Darnell	2185	PTK2B

NPD_Sets	FMRP-Darnell	9378	NRXN1
NPD_Sets	FMRP-Darnell	1951	CELSR3
NPD_Sets	FMRP-Darnell	8404	SPARCL1
NPD_Sets	FMRP-Darnell	6508	SLC4A3
NPD_Sets	FMRP-Darnell	23623	RUSC1
NPD_Sets	FMRP-Darnell	10142	AKAP9
NPD_Sets	FMRP-Darnell	21	ABCA3
NPD_Sets	FMRP-Darnell	8648	NCOA1
NPD_Sets	FMRP-Darnell	23026	MYO16
NPD_Sets	FMRP-Darnell	6543	SLC8A2
NPD_Sets	FMRP-Darnell	28996	HIPK2
NPD_Sets	FMRP-Darnell	9758	FRMPD4
NPD_Sets	FMRP-Darnell	54542	RC3H2
NPD_Sets	FMRP-Darnell	166336	PRICKLE2
NPD_Sets	FMRP-Darnell	163	AP2B1
NPD_Sets	FMRP-Darnell	6506	SLC1A2
NPD_Sets	FMRP-Darnell	9043	SPAG9
NPD_Sets	FMRP-Darnell	57609	DIP2B
NPD_Sets	FMRP-Darnell	23316	CUX2
NPD_Sets	FMRP-Darnell	11180	WDR6
NPD_Sets	FMRP-Darnell	5339	PLEC
NPD_Sets	FMRP-Darnell	29978	UBQLN2
NPD_Sets	FMRP-Darnell	10444	ZER1
NPD_Sets	FMRP-Darnell	23113	CUL9
NPD_Sets	FMRP-Darnell	7216	TRO
NPD_Sets	FMRP-Darnell	55102	ATG2B
NPD_Sets	FMRP-Darnell	5098	PCDHGC3
NPD_Sets	FMRP-Darnell	2889	RAPGEF1
NPD_Sets	FMRP-Darnell	9320	TRIP12
NPD_Sets	FMRP-Darnell	29888	STRN4
NPD_Sets	FMRP-Darnell	23369	PUM2
NPD_Sets	FMRP-Darnell	27125	AFF4
NPD_Sets	FMRP-Darnell	23264	ZC3H7B
NPD_Sets	FMRP-Darnell	9690	UBE3C
NPD_Sets	FMRP-Darnell	2309	FOXO3
NPD_Sets	FMRP-Darnell	5900	RALGDS
NPD_Sets	FMRP-Darnell	65018	PINK1
NPD_Sets	FMRP-Darnell	114787	GPRIN1
NPD_Sets	FMRP-Darnell	9706	ULK2
NPD_Sets	FMRP-Darnell	22982	DIP2C
NPD_Sets	FMRP-Darnell	375449	MAST4
NPD_Sets	FMRP-Darnell	9145	SYNGR1
NPD_Sets	FMRP-Darnell	1612	DAPK1
NPD_Sets	FMRP-Darnell	114783	LMTK3
NPD_Sets	FMRP-Darnell	23248	RPRD2
NPD_Sets	FMRP-Darnell	6616	SNAP25
NPD_Sets	FMRP-Darnell	57554	LRRC7
NPD_Sets	FMRP-Darnell	2033	EP300
NPD_Sets	FMRP-Darnell	320	APBA1
NPD_Sets	FMRP-Darnell	8189	SYMPK
NPD_Sets	FMRP-Darnell	8867	SYNJ1
NPD_Sets	FMRP-Darnell	8618	CADPS
NPD_Sets	FMRP-Darnell	85446	ZFHX2
NPD_Sets	FMRP-Darnell	23094	SIPA1L3
NPD_Sets	FMRP-Darnell	10014	HDAC5
NPD_Sets	FMRP-Darnell	7249	TSC2
NPD_Sets	FMRP-Darnell	1152	CKB
NPD_Sets	FMRP-Darnell	8671	SLC4A4

NPD_Sets	FMRP-Darnell	9793	CKAP5
NPD_Sets	FMRP-Darnell	10787	NCKAP1
NPD_Sets	FMRP-Darnell	8943	AP3D1
NPD_Sets	FMRP-Darnell	1826	DSCAM
NPD_Sets	FMRP-Darnell	56995	TULP4
NPD_Sets	FMRP-Darnell	23043	TNIK
NPD_Sets	FMRP-Darnell	273	AMPH
NPD_Sets	FMRP-Darnell	9228	DLGAP2
NPD_Sets	FMRP-Darnell	9110	MTMR4
NPD_Sets	FMRP-Darnell	5518	PPP2R1A
NPD_Sets	FMRP-Darnell	3096	HIVEP1
NPD_Sets	FMRP-Darnell	23284	LPHN3
NPD_Sets	FMRP-Darnell	90990	KIFC2
NPD_Sets	FMRP-Darnell	10194	TSHZ1
NPD_Sets	FMRP-Darnell	63916	ELMO2
NPD_Sets	FMRP-Darnell	3797	KIF3C
NPD_Sets	FMRP-Darnell	57634	EP400
NPD_Sets	FMRP-Darnell	388662	SLC6A17
NPD_Sets	FMRP-Darnell	80003	PCNXL2
NPD_Sets	FMRP-Darnell	283209	PGM2L1
NPD_Sets	FMRP-Darnell	146664	MGAT5B
NPD_Sets	FMRP-Darnell	26057	ANKRD17
NPD_Sets	FMRP-Darnell	23054	NCOA6
NPD_Sets	FMRP-Darnell	659	BMPR2
NPD_Sets	FMRP-Darnell	801	CALM1
NPD_Sets	FMRP-Darnell	5795	PTPRJ
NPD_Sets	FMRP-Darnell	8851	CDK5R1
NPD_Sets	FMRP-Darnell	6792	CDKL5
NPD_Sets	FMRP-Darnell	134957	STXBP5
NPD_Sets	FMRP-Darnell	7786	MAP3K12
NPD_Sets	FMRP-Darnell	6925	TCF4
NPD_Sets	FMRP-Darnell	3751	KCND2
NPD_Sets	FMRP-Darnell	9905	SGSM2
NPD_Sets	FMRP-Darnell	23074	UHRF1BP1L
NPD_Sets	FMRP-Darnell	57679	ALS2
NPD_Sets	FMRP-Darnell	64743	WDR13
NPD_Sets	FMRP-Darnell	808	CALM3
NPD_Sets	FMRP-Darnell	22993	HMGXB3
NPD_Sets	FMRP-Darnell	498	ATP5A1
NPD_Sets	FMRP-Darnell	9369	NRXN3
NPD_Sets	FMRP-Darnell	9698	PUM1
NPD_Sets	FMRP-Darnell	1363	CPE
NPD_Sets	FMRP-Darnell	57338	JPH3
NPD_Sets	FMRP-Darnell	10919	EHMT2
NPD_Sets	FMRP-Darnell	22864	R3HDM2
NPD_Sets	FMRP-Darnell	23394	ADNP
NPD_Sets	FMRP-Darnell	57148	RALGAPB
NPD_Sets	FMRP-Darnell	610	HCN2
NPD_Sets	FMRP-Darnell	1840	DTX1
NPD_Sets	FMRP-Darnell	23416	KCNH3
NPD_Sets	FMRP-Darnell	11113	CIT
NPD_Sets	FMRP-Darnell	9867	PJA2
NPD_Sets	FMRP-Darnell	23326	USP22
NPD_Sets	FMRP-Darnell	10446	LRRN2
NPD_Sets	FMRP-Darnell	5354	PLP1
NPD_Sets	FMRP-Darnell	5530	PPP3CA
NPD_Sets	FMRP-Darnell	79567	FAM65A
NPD_Sets	FMRP-Darnell	9839	ZEB2

NPD_Sets	FMRP-Darnell	375790	AGRN
NPD_Sets	FMRP-Darnell	57512	GPR158
NPD_Sets	FMRP-Darnell	3996	LLGL1
NPD_Sets	FMRP-Darnell	57680	CHD8
NPD_Sets	FMRP-Darnell	54497	HEATR5B
NPD_Sets	FMRP-Darnell	476	ATP1A1
NPD_Sets	FMRP-Darnell	80243	PREX2
NPD_Sets	FMRP-Darnell	253959	RALGAPA1
NPD_Sets	FMRP-Darnell	4150	MAZ
NPD_Sets	FMRP-Darnell	440073	IQSEC3
NPD_Sets	FMRP-Darnell	9747	FAM115A
NPD_Sets	FMRP-Darnell	22941	SHANK2
NPD_Sets	FMRP-Darnell	55690	PACS1
NPD_Sets	FMRP-Darnell	230	ALDOC
NPD_Sets	FMRP-Darnell	1400	CRMP1
NPD_Sets	FMRP-Darnell	503542	SPRN
NPD_Sets	FMRP-Darnell	29767	TMOD2
NPD_Sets	FMRP-Darnell	11033	ADAP1
NPD_Sets	FMRP-Darnell	84687	PPP1R9B
NPD_Sets	FMRP-Darnell	256472	TMEM151A
NPD_Sets	FMRP-Darnell	286	ANK1
NPD_Sets	FMRP-Darnell	118987	PDZD8
NPD_Sets	FMRP-Darnell	23286	WWC1
NPD_Sets	FMRP-Darnell	55832	CAND1
NPD_Sets	FMRP-Darnell	351	APP
NPD_Sets	FMRP-Darnell	79633	FAT4
NPD_Sets	FMRP-Darnell	162	AP1B1
NPD_Sets	FMRP-Darnell	1958	EGR1
NPD_Sets	FMRP-Darnell	23476	BRD4
NPD_Sets	FMRP-Darnell	149041	RC3H1
NPD_Sets	FMRP-Darnell	57447	NDRG2
NPD_Sets	FMRP-Darnell	2902	GRIN1
NPD_Sets	FMRP-Darnell	23523	CABIN1
NPD_Sets	FMRP-Darnell	8242	KDM5C
NPD_Sets	FMRP-Darnell	22843	PPM1E
NPD_Sets	FMRP-Darnell	104	ADARB1
NPD_Sets	FMRP-Darnell	5582	PRKCG
NPD_Sets	FMRP-Darnell	7102	TSPAN7
NPD_Sets	FMRP-Darnell	9282	MED14
NPD_Sets	FMRP-Darnell	9024	BRSK2
NPD_Sets	FMRP-Darnell	8936	WASF1
NPD_Sets	FMRP-Darnell	1917	EEF1A2
NPD_Sets	FMRP-Darnell	10612	TRIM3
NPD_Sets	FMRP-Darnell	54878	DPP8
NPD_Sets	FMRP-Darnell	23646	PLD3
NPD_Sets	FMRP-Darnell	10499	NCOA2
NPD_Sets	FMRP-Darnell	784	CACNB3
NPD_Sets	FMRP-Darnell	3748	KCNC3
NPD_Sets	FMRP-Darnell	3098	HK1
NPD_Sets	FMRP-Darnell	9826	ARHGEF11
NPD_Sets	FMRP-Darnell	79699	ZYG11B
NPD_Sets	FMRP-Darnell	5728	PTEN
NPD_Sets	FMRP-Darnell	283149	BCL9L
NPD_Sets	FMRP-Darnell	5101	PCDH9
NPD_Sets	FMRP-Darnell	5364	PLXNB1
NPD_Sets	FMRP-Darnell	23321	TRIM2
NPD_Sets	FMRP-Darnell	5310	PKD1
NPD_Sets	FMRP-Darnell	9258	MFHAS1

NPD_Sets	FMRP-Darnell	26050	SLITRK5
NPD_Sets	FMRP-Darnell	2899	GRIK3
NPD_Sets	FMRP-Darnell	116987	AGAP1
NPD_Sets	FMRP-Darnell	55095	SAMD4B
NPD_Sets	FMRP-Darnell	10114	HIPK3
NPD_Sets	FMRP-Darnell	3843	IPO5
NPD_Sets	FMRP-Darnell	2186	BPTF
NPD_Sets	FMRP-Darnell	481	ATP1B1
NPD_Sets	FMRP-Darnell	4862	NPAS2
NPD_Sets	FMRP-Darnell	9215	LARGE
NPD_Sets	FMRP-Darnell	4037	LRP3
NPD_Sets	FMRP-Darnell	23041	MON2
NPD_Sets	FMRP-Darnell	9919	SEC16A
NPD_Sets	FMRP-Darnell	57224	NHSL1
NPD_Sets	FMRP-Darnell	23013	SPEN
NPD_Sets	FMRP-Darnell	57551	TAOK1
NPD_Sets	FMRP-Darnell	57468	SLC12A5
NPD_Sets	FMRP-Darnell	473	RERE
NPD_Sets	FMRP-Darnell	5099	PCDH7
NPD_Sets	FMRP-Darnell	156	ADRBK1
NPD_Sets	FMRP-Darnell	8470	SORBS2
NPD_Sets	FMRP-Darnell	4900	NRGN
NPD_Sets	FMRP-Darnell	57521	RPTOR
NPD_Sets	FMRP-Darnell	55084	SOBP
NPD_Sets	FMRP-Darnell	3899	AFF3
NPD_Sets	FMRP-Darnell	84067	FAM160A2
NPD_Sets	FMRP-Darnell	116984	ARAP2
NPD_Sets	FMRP-Darnell	535	ATP6V0A1
NPD_Sets	FMRP-Darnell	5793	PTPRG
NPD_Sets	FMRP-Darnell	5649	RELN
NPD_Sets	FMRP-Darnell	84181	CHD6
NPD_Sets	FMRP-Darnell	1487	CTBP1
NPD_Sets	FMRP-Darnell	6844	VAMP2
NPD_Sets	FMRP-Darnell	51310	SLC22A17
NPD_Sets	FMRP-Darnell	9900	SV2A
NPD_Sets	FMRP-Darnell	57569	ARHGAP20
NPD_Sets	FMRP-Darnell	55729	ATF7IP
NPD_Sets	FMRP-Darnell	22907	DHX30
NPD_Sets	FMRP-Darnell	5980	REV3L
NPD_Sets	FMRP-Darnell	9498	SLC4A8
NPD_Sets	FMRP-Darnell	11069	RAPGEF4
NPD_Sets	FMRP-Darnell	79711	IPO4
NPD_Sets	FMRP-Darnell	55777	MBD5
NPD_Sets	FMRP-Darnell	114794	ELFN2
NPD_Sets	FMRP-Darnell	23187	PHLDB1
NPD_Sets	FMRP-Darnell	5789	PTPRD
NPD_Sets	FMRP-Darnell	9990	SLC12A6
NPD_Sets	FMRP-Darnell	23467	NPTXR
NPD_Sets	FMRP-Darnell	10891	PPARGC1A
NPD_Sets	FMRP-Darnell	7025	NR2F1
NPD_Sets	FMRP-Darnell	8660	IRS2
NPD_Sets	FMRP-Darnell	9181	ARHGEF2
NPD_Sets	FMRP-Darnell	9663	LPIN2
NPD_Sets	FMRP-Darnell	9898	UBAP2L
NPD_Sets	FMRP-Darnell	10313	RTN3
NPD_Sets	FMRP-Darnell	441151	TMEM151B
NPD_Sets	FMRP-Darnell	6942	TCF20
NPD_Sets	FMRP-Darnell	150726	FBXO41

NPD_Sets	FMRP-Darnell	26133	TRPC4AP
NPD_Sets	FMRP-Darnell	2823	GPM6A
NPD_Sets	FMRP-Darnell	5526	PPP2R5B
NPD_Sets	FMRP-Darnell	23039	XPO7
NPD_Sets	FMRP-Darnell	54972	TMEM132A
NPD_Sets	FMRP-Darnell	4155	MBP
NPD_Sets	FMRP-Darnell	26470	SEZ6L2
NPD_Sets	FMRP-Darnell	57582	KCNT1
NPD_Sets	FMRP-Darnell	116988	AGAP3
NPD_Sets	FMRP-Darnell	1523	CUX1
NPD_Sets	FMRP-Darnell	57636	ARHGAP23
NPD_Sets	FMRP-Darnell	161742	SPRED1
NPD_Sets	FMRP-Darnell	23116	FAM179B
NPD_Sets	FMRP-Darnell	339983	NAT8L
NPD_Sets	FMRP-Darnell	57678	GPAM
NPD_Sets	FMRP-Darnell	7532	YWHAG
NPD_Sets	FMRP-Darnell	10025	MED16
NPD_Sets	FMRP-Darnell	89797	NAV2
NPD_Sets	FMRP-Darnell	165215	FAM171B
NPD_Sets	FMRP-Darnell	10565	ARFGEF1
NPD_Sets	FMRP-Darnell	11076	TPPP
NPD_Sets	FMRP-Darnell	23405	DICER1
NPD_Sets	FMRP-Darnell	3716	JAK1
NPD_Sets	FMRP-Darnell	526	ATP6V1B2
NPD_Sets	FMRP-Darnell	51754	TMEM8B
NPD_Sets	FMRP-Darnell	4782	NFIC
NPD_Sets	FMRP-Darnell	54413	NLGN3
NPD_Sets	FMRP-Darnell	4651	MYO10
NPD_Sets	FMRP-Darnell	6710	SPTB
NPD_Sets	FMRP-Darnell	10814	CPLX2
NPD_Sets	FMRP-Darnell	221692	PHACTR1
NPD_Sets	FMRP-Darnell	84251	SGIP1
NPD_Sets	FMRP-Darnell	377	ARF3
NPD_Sets	FMRP-Darnell	8672	EIF4G3
NPD_Sets	FMRP-Darnell	375323	LHFPL4
NPD_Sets	FMRP-Darnell	23373	CRTC1
NPD_Sets	FMRP-Darnell	200576	PIKFYVE
NPD_Sets	FMRP-Darnell	23242	COBL
NPD_Sets	FMRP-Darnell	2781	GNAZ
NPD_Sets	FMRP-Darnell	51366	UBR5
NPD_Sets	FMRP-Darnell	157922	CAMSAP1
NPD_Sets	FMRP-Darnell	57169	ZNFX1
NPD_Sets	FMRP-Darnell	145567	TTC7B
NPD_Sets	FMRP-Darnell	80036	TRPM3
NPD_Sets	FMRP-Darnell	23112	TNRC6B
NPD_Sets	FMRP-Darnell	6624	FSCN1
NPD_Sets	FMRP-Darnell	10395	DLC1
NPD_Sets	FMRP-Darnell	118	ADD1
NPD_Sets	FMRP-Darnell	2534	FYN
NPD_Sets	FMRP-Darnell	9796	PHYHIP
NPD_Sets	FMRP-Darnell	63893	UBE2O
NPD_Sets	FMRP-Darnell	84861	KLHL22
NPD_Sets	FMRP-Darnell	9759	HDAC4
NPD_Sets	FMRP-Darnell	1762	DMWD
NPD_Sets	FMRP-Darnell	10494	STK25
NPD_Sets	FMRP-Darnell	8658	TNKS
NPD_Sets	FMRP-Darnell	9854	C2CD2L
NPD_Sets	FMRP-Darnell	9651	PLCH2

NPD_Sets	FMRP-Darnell	51230	PHF20
NPD_Sets	FMRP-Darnell	9751	SNPH
NPD_Sets	FMRP-Darnell	23328	SASH1
NPD_Sets	FMRP-Darnell	22820	COPG1
NPD_Sets	FMRP-Darnell	774	CACNA1B
NPD_Sets	FMRP-Darnell	1123	CHN1
NPD_Sets	FMRP-Darnell	9915	ARNT2
NPD_Sets	FMRP-Darnell	5128	CDK17
NPD_Sets	FMRP-Darnell	2043	EPHA4
NPD_Sets	FMRP-Darnell	7384	UQCRC1
NPD_Sets	FMRP-Darnell	6529	SLC6A1
NPD_Sets	FMRP-Darnell	8522	GAS7
NPD_Sets	FMRP-Darnell	64759	TNS3
NPD_Sets	FMRP-Darnell	56907	SPIRE1
NPD_Sets	FMRP-Darnell	6272	SORT1
NPD_Sets	FMRP-Darnell	10000	AKT3
NPD_Sets	FMRP-Darnell	11100	HNRNPUL1
NPD_Sets	FMRP-Darnell	5727	PTCH1
NPD_Sets	FMRP-Darnell	126129	CPT1C
NPD_Sets	FMRP-Darnell	1267	CNP
NPD_Sets	FMRP-Darnell	22954	TRIM32
NPD_Sets	FMRP-Darnell	4684	NCAM1
NPD_Sets	FMRP-Darnell	6397	SEC14L1
NPD_Sets	FMRP-Darnell	57453	DSCAML1
NPD_Sets	FMRP-Darnell	55964	SEPT3
NPD_Sets	FMRP-Darnell	4916	NTRK3
NPD_Sets	FMRP-Darnell	23420	NOMO1
NPD_Sets	FMRP-Darnell	23677	SH3BP4
NPD_Sets	FMRP-Darnell	782	CACNB1
NPD_Sets	FMRP-Darnell	8622	PDE8B
NPD_Sets	FMRP-Darnell	5142	PDE4B
NPD_Sets	FMRP-Darnell	6297	SALL2
NPD_Sets	FMRP-Darnell	4905	NSF
NPD_Sets	FMRP-Darnell	9231	DLG5
NPD_Sets	FMRP-Darnell	23135	KDM6B
NPD_Sets	FMRP-Darnell	388	RHOB
NPD_Sets	FMRP-Darnell	84444	DOT1L
NPD_Sets	FMRP-Darnell	9619	ABCG1
NPD_Sets	FMRP-Darnell	7804	LRP8
NPD_Sets	FMRP-Darnell	84669	USP32
NPD_Sets	FMRP-Darnell	54769	DIRAS2
NPD_Sets	FMRP-Darnell	26025	PCDHGA12
NPD_Sets	FMRP-Darnell	10381	TUBB3
NPD_Sets	FMRP-Darnell	50649	ARHGEF4
NPD_Sets	FMRP-Darnell	10645	CAMKK2
NPD_Sets	FMRP-Darnell	401190	RGS7BP
NPD_Sets	FMRP-Darnell	84867	PTPN5
NPD_Sets	FMRP-Darnell	8204	NRIP1
NPD_Sets	FMRP-Darnell	23108	RAP1GAP2
NPD_Sets	FMRP-Darnell	2975	GTF3C1
NPD_Sets	FMRP-Darnell	3607	FOXK2
NPD_Sets	FMRP-Darnell	79065	ATG9A
NPD_Sets	FMRP-Darnell	2976	GTF3C2
NPD_Sets	FMRP-Darnell	9863	MAGI2
NPD_Sets	FMRP-Darnell	8541	PPFIA3
NPD_Sets	FMRP-Darnell	23064	SETX
NPD_Sets	FMRP-Darnell	23241	PACS2
NPD_Sets	FMRP-Darnell	3785	KCNQ2

NPD_Sets	FMRP-Darnell	54620	FBXL19
NPD_Sets	FMRP-Darnell	5430	POLR2A
NPD_Sets	FMRP-Darnell	4209	MEF2D
NPD_Sets	FMRP-Darnell	9865	TRIL
NPD_Sets	FMRP-Darnell	11176	BAZ2A
NPD_Sets	FMRP-Darnell	7090	TLE3
NPD_Sets	FMRP-Darnell	9091	PIGQ
NPD_Sets	FMRP-Darnell	84253	GARNL3
NPD_Sets	FMRP-Darnell	79085	SLC25A23
NPD_Sets	FMRP-Darnell	5594	MAPK1
NPD_Sets	FMRP-Darnell	27087	B3GAT1
NPD_Sets	FMRP-Darnell	55512	SMPD3
NPD_Sets	FMRP-Darnell	23270	TSPYL4
NPD_Sets	FMRP-Darnell	322	APBB1
NPD_Sets	FMRP-Darnell	10893	MMP24
NPD_Sets	FMRP-Darnell	493	ATP2B4
NPD_Sets	FMRP-Darnell	60680	CELF5
NPD_Sets	FMRP-Darnell	10290	SPEG
NPD_Sets	FMRP-Darnell	23413	NCS1
NPD_Sets	FMRP-Darnell	5596	MAPK4
NPD_Sets	FMRP-Darnell	6546	SLC8A1
NPD_Sets	FMRP-Darnell	9693	RAPGEF2
NPD_Sets	FMRP-Darnell	56894	AGPAT3
NPD_Sets	FMRP-Darnell	27239	GPR162
NPD_Sets	FMRP-Darnell	5747	PTK2
NPD_Sets	FMRP-Darnell	11346	SYNPO
NPD_Sets	FMRP-Darnell	4928	NUP98
NPD_Sets	FMRP-Darnell	9114	ATP6V0D1
NPD_Sets	FMRP-Darnell	23221	RHOBTB2
NPD_Sets	FMRP-Darnell	55362	TMEM63B
NPD_Sets	FMRP-Darnell	23770	FKBP8
NPD_Sets	FMRP-Darnell	10815	CPLX1
NPD_Sets	FMRP-Darnell	1182	CLCN3
NPD_Sets	FMRP-Darnell	2914	GRM4
NPD_Sets	FMRP-Darnell	90249	UNC5A
NPD_Sets	FMRP-Darnell	10484	SEC23A
NPD_Sets	FMRP-Darnell	55074	OXR1
NPD_Sets	FMRP-Darnell	4628	MYH10
NPD_Sets	FMRP-Darnell	23051	ZHX3
NPD_Sets	FMRP-Darnell	23149	FCHO1
NPD_Sets	FMRP-Darnell	23005	MAPKBP1
NPD_Sets	FMRP-Darnell	57534	MIB1
NPD_Sets	FMRP-Darnell	7008	TEF
NPD_Sets	FMRP-Darnell	84502	JPH4
NPD_Sets	FMRP-Darnell	29979	UBQLN1
NPD_Sets	FMRP-Darnell	8314	BAP1
NPD_Sets	FMRP-Darnell	55450	CAMK2N1
NPD_Sets	FMRP-Darnell	55209	SETD5
NPD_Sets	FMRP-Darnell	79595	SAP130
NPD_Sets	FMRP-Darnell	2932	GSK3B
NPD_Sets	FMRP-Darnell	23300	ATMIN
NPD_Sets	FMRP-Darnell	5187	PER1
NPD_Sets	FMRP-Darnell	23047	PDS5B
NPD_Sets	FMRP-Darnell	226	ALDOA
NPD_Sets	FMRP-Darnell	5909	RAP1GAP
NPD_Sets	FMRP-Darnell	55827	DCAF6
NPD_Sets	FMRP-Darnell	90134	KCNH7
NPD_Sets	FMRP-Darnell	1124	CHN2

NPD_Sets	FMRP-Darnell	8078	USP5
NPD_Sets	FMRP-Darnell	66004	LYNX1
NPD_Sets	FMRP-Darnell	23148	NACAD
NPD_Sets	FMRP-Darnell	421	ARVCF
NPD_Sets	FMRP-Darnell	10489	LRRC41
NPD_Sets	FMRP-Darnell	3737	KCNA2
NPD_Sets	FMRP-Darnell	56144	PCDHA4
NPD_Sets	FMRP-Darnell	55605	KIF21A
NPD_Sets	FMRP-Darnell	1837	DTNA
NPD_Sets	FMRP-Darnell	57649	PHF12
NPD_Sets	FMRP-Darnell	5213	PFKM
NPD_Sets	FMRP-Darnell	5097	PCDH1
NPD_Sets	FMRP-Darnell	23236	PLCB1
NPD_Sets	FMRP-Darnell	53349	ZFYVE1
NPD_Sets	FMRP-Darnell	408	ARRB1
NPD_Sets	FMRP-Darnell	284434	NWD1
NPD_Sets	FMRP-Darnell	23030	KDM4B
NPD_Sets	FMRP-Darnell	65078	RTN4R
NPD_Sets	FMRP-Darnell	5781	PTPN11
NPD_Sets	FMRP-Darnell	23211	ZC3H4
NPD_Sets	FMRP-Darnell	64682	ANAPC1
NPD_Sets	FMRP-Darnell	348	APOE
NPD_Sets	FMRP-Darnell	6310	ATXN1
NPD_Sets	FMRP-Darnell	5567	PRKACB
NPD_Sets	FMRP-Darnell	23033	DOPEY1
NPD_Sets	FMRP-Darnell	3786	KCNQ3
NPD_Sets	FMRP-Darnell	47	ACLY
NPD_Sets	FMRP-Darnell	63827	BCAN
NPD_Sets	FMRP-Darnell	3069	HDLBP
NPD_Sets	FMRP-Darnell	9993	DGCR2
NPD_Sets	FMRP-Darnell	5660	PSAP
NPD_Sets	FMRP-Darnell	27072	VPS41
NPD_Sets	FMRP-Darnell	79813	EHMT1
NPD_Sets	FMRP-Darnell	24139	EML2
NPD_Sets	ID-candidates	2517	FUCA1
NPD_Sets	ID-candidates	6513	SLC2A1
NPD_Sets	ID-candidates	6491	STIL
NPD_Sets	ID-candidates	29929	ALG6
NPD_Sets	ID-candidates	1629	DBT
NPD_Sets	ID-candidates	4893	NRAS
NPD_Sets	ID-candidates	3766	KCNJ10
NPD_Sets	ID-candidates	259266	ASPM
NPD_Sets	ID-candidates	23418	CRB1
NPD_Sets	ID-candidates	343035	RD3
NPD_Sets	ID-candidates	6905	TBCE
NPD_Sets	ID-candidates	2271	FH
NPD_Sets	ID-candidates	4613	MYCN
NPD_Sets	ID-candidates	6654	SOS1
NPD_Sets	ID-candidates	2071	ERCC3
NPD_Sets	ID-candidates	22930	RAB3GAP1
NPD_Sets	ID-candidates	9839	ZEB2
NPD_Sets	ID-candidates	129880	BBS5
NPD_Sets	ID-candidates	2571	GAD1
NPD_Sets	ID-candidates	9759	HDAC4
NPD_Sets	ID-candidates	51185	CRBN
NPD_Sets	ID-candidates	285362	SUMF1
NPD_Sets	ID-candidates	80746	TSEN2
NPD_Sets	ID-candidates	5894	RAF1

NPD_Sets	ID-candidates	7048	TGFBR2
NPD_Sets	ID-candidates	2720	GLB1
NPD_Sets	ID-candidates	200894	ARL13B
NPD_Sets	ID-candidates	84100	ARL6
NPD_Sets	ID-candidates	545	ATR
NPD_Sets	ID-candidates	10195	ALG3
NPD_Sets	ID-candidates	9711	KIAA0226
NPD_Sets	ID-candidates	57545	CC2D2A
NPD_Sets	ID-candidates	5860	QDPR
NPD_Sets	ID-candidates	79644	SRD5A3
NPD_Sets	ID-candidates	8671	SLC4A4
NPD_Sets	ID-candidates	55212	BBS7
NPD_Sets	ID-candidates	166379	BBS12
NPD_Sets	ID-candidates	9227	LRAT
NPD_Sets	ID-candidates	175	AGA
NPD_Sets	ID-candidates	56172	ANKH
NPD_Sets	ID-candidates	4338	MOCS2
NPD_Sets	ID-candidates	1161	ERCC8
NPD_Sets	ID-candidates	347733	TUBB2B
NPD_Sets	ID-candidates	4758	NEU1
NPD_Sets	ID-candidates	4337	MOCS1
NPD_Sets	ID-candidates	26503	SLC17A5
NPD_Sets	ID-candidates	167691	LCA5
NPD_Sets	ID-candidates	594	BCKDHB
NPD_Sets	ID-candidates	2898	GRIK2
NPD_Sets	ID-candidates	55084	SOBP
NPD_Sets	ID-candidates	3908	LAMA2
NPD_Sets	ID-candidates	383	ARG1
NPD_Sets	ID-candidates	5191	PEX7
NPD_Sets	ID-candidates	404672	GTF2H5
NPD_Sets	ID-candidates	27241	BBS9
NPD_Sets	ID-candidates	2990	GUSB
NPD_Sets	ID-candidates	9179	AP4M1
NPD_Sets	ID-candidates	5649	RELN
NPD_Sets	ID-candidates	1738	DLD
NPD_Sets	ID-candidates	3614	IMPDH1
NPD_Sets	ID-candidates	79648	MCPH1
NPD_Sets	ID-candidates	7991	TUSC3
NPD_Sets	ID-candidates	91147	TMEM67
NPD_Sets	ID-candidates	51305	KCNK9
NPD_Sets	ID-candidates	83696	TRAPPC9
NPD_Sets	ID-candidates	7436	VLDLR
NPD_Sets	ID-candidates	7046	TGFBR1
NPD_Sets	ID-candidates	2218	FKTN
NPD_Sets	ID-candidates	22954	TRIM32
NPD_Sets	ID-candidates	55755	CDK5RAP2
NPD_Sets	ID-candidates	6709	SPTAN1
NPD_Sets	ID-candidates	56623	INPP5E
NPD_Sets	ID-candidates	2074	ERCC6
NPD_Sets	ID-candidates	26128	KIAA1279
NPD_Sets	ID-candidates	9126	SMC3
NPD_Sets	ID-candidates	8036	SHOC2
NPD_Sets	ID-candidates	79751	SLC25A22
NPD_Sets	ID-candidates	5080	PAX6
NPD_Sets	ID-candidates	55343	SLC35C1
NPD_Sets	ID-candidates	51259	TMEM216
NPD_Sets	ID-candidates	582	BBS1
NPD_Sets	ID-candidates	79053	ALG8

NPD_Sets	ID-candidates	9440	MED17
NPD_Sets	ID-candidates	79796	ALG9
NPD_Sets	ID-candidates	867	CBL
NPD_Sets	ID-candidates	5818	PVRL1
NPD_Sets	ID-candidates	84623	KIRREL3
NPD_Sets	ID-candidates	7846	TUBA1A
NPD_Sets	ID-candidates	57609	DIP2B
NPD_Sets	ID-candidates	6821	SUOX
NPD_Sets	ID-candidates	79738	BBS10
NPD_Sets	ID-candidates	79158	GNPTAB
NPD_Sets	ID-candidates	3479	IGF1
NPD_Sets	ID-candidates	23545	ATP6V0A2
NPD_Sets	ID-candidates	55835	CENPJ
NPD_Sets	ID-candidates	10166	SLC25A15
NPD_Sets	ID-candidates	2073	ERCC5
NPD_Sets	ID-candidates	1282	COL4A1
NPD_Sets	ID-candidates	57096	RPGRIP1
NPD_Sets	ID-candidates	4247	MGAT2
NPD_Sets	ID-candidates	145226	RDH12
NPD_Sets	ID-candidates	29954	POMT2
NPD_Sets	ID-candidates	2581	GALC
NPD_Sets	ID-candidates	55812	SPATA7
NPD_Sets	ID-candidates	123016	TTC8
NPD_Sets	ID-candidates	7443	VRK1
NPD_Sets	ID-candidates	161742	SPRED1
NPD_Sets	ID-candidates	22995	CEP152
NPD_Sets	ID-candidates	23431	AP4E1
NPD_Sets	ID-candidates	585	BBS4
NPD_Sets	ID-candidates	84572	GNPTG
NPD_Sets	ID-candidates	57465	TBC1D24
NPD_Sets	ID-candidates	5373	PMM2
NPD_Sets	ID-candidates	583	BBS2
NPD_Sets	ID-candidates	9289	GPR56
NPD_Sets	ID-candidates	84342	COG8
NPD_Sets	ID-candidates	1013	CDH15
NPD_Sets	ID-candidates	23746	AIPL1
NPD_Sets	ID-candidates	9526	MPDU1
NPD_Sets	ID-candidates	113235	SLC46A1
NPD_Sets	ID-candidates	2670	GFAP
NPD_Sets	ID-candidates	54903	MKS1
NPD_Sets	ID-candidates	9382	COG1
NPD_Sets	ID-candidates	283989	TSEN54
NPD_Sets	ID-candidates	26040	SETBP1
NPD_Sets	ID-candidates	6925	TCF4
NPD_Sets	ID-candidates	5605	MAP2K2
NPD_Sets	ID-candidates	57192	MCOLN1
NPD_Sets	ID-candidates	54862	CC2D1A
NPD_Sets	ID-candidates	284403	WDR62
NPD_Sets	ID-candidates	593	BCKDHA
NPD_Sets	ID-candidates	2068	ERCC2
NPD_Sets	ID-candidates	2067	ERCC1
NPD_Sets	ID-candidates	79147	FKRP
NPD_Sets	ID-candidates	1406	CRX
NPD_Sets	ID-candidates	11284	PNKP
NPD_Sets	ID-candidates	1789	DNMT3B
NPD_Sets	ID-candidates	9342	SNAP29
NPD_Sets	ID-candidates	9215	LARGE
NPD_Sets	ID-candidates	2033	EP300

NPD_Sets	ID-candidates	79087	ALG12
NPD_Sets	ID-candidates	5476	CTSA
NPD_Sets	ID-candidates	10564	ARFGEF2
NPD_Sets	ID-candidates	8813	DPM1
NPD_Sets	ID-candidates	875	CBS
NPD_Sets	ID-candidates	5116	PCNT
NPD_Sets	ID-candidates	3052	HCCS
NPD_Sets	ID-candidates	8481	OFD1
NPD_Sets	ID-candidates	2187	FANCB
NPD_Sets	ID-candidates	5160	PDHA1
NPD_Sets	ID-candidates	6197	RPS6KA3
NPD_Sets	ID-candidates	6611	SMS
NPD_Sets	ID-candidates	2710	GK
NPD_Sets	ID-candidates	7102	TSPAN7
NPD_Sets	ID-candidates	54880	BCOR
NPD_Sets	ID-candidates	10159	ATP6AP2
NPD_Sets	ID-candidates	4128	MAOA
NPD_Sets	ID-candidates	7592	ZNF41
NPD_Sets	ID-candidates	64840	PORCN
NPD_Sets	ID-candidates	6855	SYP
NPD_Sets	ID-candidates	57477	SHROOM4
NPD_Sets	ID-candidates	3028	HSD17B10
NPD_Sets	ID-candidates	10075	HUWE1
NPD_Sets	ID-candidates	11279	KLF8
NPD_Sets	ID-candidates	23229	ARHGEF9
NPD_Sets	ID-candidates	3476	IGBP1
NPD_Sets	ID-candidates	1741	DLG3
NPD_Sets	ID-candidates	6567	SLC16A2
NPD_Sets	ID-candidates	84061	MAGT1
NPD_Sets	ID-candidates	538	ATP7A
NPD_Sets	ID-candidates	5230	PGK1
NPD_Sets	ID-candidates	254065	BRWD3
NPD_Sets	ID-candidates	7552	ZNF711
NPD_Sets	ID-candidates	27286	SRPX2
NPD_Sets	ID-candidates	1678	TIMM8A
NPD_Sets	ID-candidates	5354	PLP1
NPD_Sets	ID-candidates	5631	PRPS1
NPD_Sets	ID-candidates	5063	PAK3
NPD_Sets	ID-candidates	7319	UBE2A
NPD_Sets	ID-candidates	4694	NDUFA1
NPD_Sets	ID-candidates	8450	CUL4B
NPD_Sets	ID-candidates	51114	ZDHHC9
NPD_Sets	ID-candidates	2719	GPC3
NPD_Sets	ID-candidates	3251	HPRT1
NPD_Sets	ID-candidates	6658	SOX3
NPD_Sets	ID-candidates	50814	NSDHL
NPD_Sets	ID-candidates	215	ABCD1
NPD_Sets	ID-candidates	554	AVPR2
NPD_Sets	ID-candidates	2316	FLNA
NPD_Sets	ID-candidates	2664	GDI1
NPD_Sets	ID-candidates	8517	IKBKG
NPD_Sets	ID-candidates	1736	DKC1
NPD_Sets	ID-denovo-lof	9204	ZMYM6
NPD_Sets	ID-denovo-lof	23040	MYT1L
NPD_Sets	ID-denovo-lof	1499	CTNNB1
NPD_Sets	ID-denovo-lof	10492	SYNCRIP
NPD_Sets	ID-denovo-lof	4036	LRP2
NPD_Sets	ID-denovo-lof	7846	TUBA1A

NPD_Sets	ID-denovo-lof	5160	PDHA1
NPD_Sets	ID-denovo-lof	2888	GRB14
NPD_Sets	ID-denovo-lof	6326	SCN2A
NPD_Sets	ID-denovo-lof	25820	ARIH1
NPD_Sets	ID-denovo-lof	1654	DDX3X
NPD_Sets	ID-denovo-lof	8831	SYNGAP1
NPD_Sets	ID-denovo-lof	23036	ZNF292
NPD_Sets	ID-denovo-lof	6529	SLC6A1
NPD_Sets	ID-denovo-lof	4214	MAP3K1
NPD_Sets	ID-denovo-lof	55209	SETD5
NPD_Sets	ID-denovo-lof	283471	TMPRSS12
NPD_Sets	ID-denovo-lof	55023	PHIP
NPD_Sets	ID-denovo-lof	4520	MTF1
NPD_Sets	ID-denovo-lof	23096	IQSEC2
NPD_Sets	ID-denovo-lof	3097	HIVEP2
NPD_Sets	ID-denovo-lof	4204	MECP2
NPD_Sets	ID-denovo-lof	57459	GATAD2B
NPD_Sets	ID-denovo-lof	1106	CHD2
NPD_Sets	ID-denovo-lof	6812	STXBP1
NPD_Sets	ID-denovo-lof	26040	SETBP1
NPD_Sets	ID-denovo-lof	51322	WAC
NPD_Sets	ID-denovo-lof	8546	AP3B1
NPD_Sets	ID-denovo-lof	83852	SETDB2
NPD_Sets	ID-denovo-lof	283489	CHAMP1
NPD_Sets	ID-denovo-nonsyn	1496	CTNNA2
NPD_Sets	ID-denovo-nonsyn	10472	ZBTB18
NPD_Sets	ID-denovo-nonsyn	6812	STXBP1
NPD_Sets	ID-denovo-nonsyn	55209	SETD5
NPD_Sets	ID-denovo-nonsyn	10522	DEAF1
NPD_Sets	ID-denovo-nonsyn	83852	SETDB2
NPD_Sets	ID-denovo-nonsyn	23040	MYT1L
NPD_Sets	ID-denovo-nonsyn	283489	CHAMP1
NPD_Sets	ID-denovo-nonsyn	4204	MECP2
NPD_Sets	ID-denovo-nonsyn	1654	DDX3X
NPD_Sets	ID-denovo-nonsyn	4292	MLH1
NPD_Sets	ID-denovo-nonsyn	23314	SATB2
NPD_Sets	ID-denovo-nonsyn	48	ACO1
NPD_Sets	ID-denovo-nonsyn	55023	PHIP
NPD_Sets	ID-denovo-nonsyn	6326	SCN2A
NPD_Sets	ID-denovo-nonsyn	25791	NGEF
NPD_Sets	ID-denovo-nonsyn	10564	ARFGEF2
NPD_Sets	ID-denovo-nonsyn	4035	LRP1
NPD_Sets	ID-denovo-nonsyn	4036	LRP2
NPD_Sets	ID-denovo-nonsyn	1261	CNGA3
NPD_Sets	ID-denovo-nonsyn	10274	STAG1
NPD_Sets	ID-denovo-nonsyn	10492	SYNCRIP
NPD_Sets	ID-denovo-nonsyn	283471	TMPRSS12
NPD_Sets	ID-denovo-nonsyn	160851	DGKH
NPD_Sets	ID-denovo-nonsyn	56660	KCNK12
NPD_Sets	ID-denovo-nonsyn	238	ALK
NPD_Sets	ID-denovo-nonsyn	100137047	JMJD7
NPD_Sets	ID-denovo-nonsyn	221692	PHACTR1
NPD_Sets	ID-denovo-nonsyn	4214	MAP3K1
NPD_Sets	ID-denovo-nonsyn	401285	TCP10L2
NPD_Sets	ID-denovo-nonsyn	23096	IQSEC2
NPD_Sets	ID-denovo-nonsyn	6721	SREBF2
NPD_Sets	ID-denovo-nonsyn	1778	DYNC1H1
NPD_Sets	ID-denovo-nonsyn	6513	SLC2A1

NPD_Sets	ID-denovo-nonsyn	904	CCNT1
NPD_Sets	ID-denovo-nonsyn	4643	MYO1E
NPD_Sets	ID-denovo-nonsyn	6334	SCN8A
NPD_Sets	ID-denovo-nonsyn	128611	ZNF831
NPD_Sets	ID-denovo-nonsyn	7846	TUBA1A
NPD_Sets	ID-denovo-nonsyn	4705	NDUFA10
NPD_Sets	ID-denovo-nonsyn	5688	PSMA7
NPD_Sets	ID-denovo-nonsyn	9940	DLEC1
NPD_Sets	ID-denovo-nonsyn	4225	MEP1B
NPD_Sets	ID-denovo-nonsyn	57459	GATAD2B
NPD_Sets	ID-denovo-nonsyn	5160	PDHA1
NPD_Sets	ID-denovo-nonsyn	10487	CAP1
NPD_Sets	ID-denovo-nonsyn	146664	MGAT5B
NPD_Sets	ID-denovo-nonsyn	90592	ZNF700
NPD_Sets	ID-denovo-nonsyn	8260	NAA10
NPD_Sets	ID-denovo-nonsyn	3800	KIF5C
NPD_Sets	ID-denovo-nonsyn	23036	ZNF292
NPD_Sets	ID-denovo-nonsyn	7204	TRIO
NPD_Sets	ID-denovo-nonsyn	6925	TCF4
NPD_Sets	ID-denovo-nonsyn	119467	CLRN3
NPD_Sets	ID-denovo-nonsyn	155054	ZNF425
NPD_Sets	ID-denovo-nonsyn	9724	UTP14C
NPD_Sets	ID-denovo-nonsyn	10753	CAPN9
NPD_Sets	ID-denovo-nonsyn	4750	NEK1
NPD_Sets	ID-denovo-nonsyn	1917	EEF1A2
NPD_Sets	ID-denovo-nonsyn	1106	CHD2
NPD_Sets	ID-denovo-nonsyn	342574	KRT27
NPD_Sets	ID-denovo-nonsyn	26523	AGO1
NPD_Sets	ID-denovo-nonsyn	29083	GTPBP8
NPD_Sets	ID-denovo-nonsyn	23316	CUX2
NPD_Sets	ID-denovo-nonsyn	1499	CTNNB1
NPD_Sets	ID-denovo-nonsyn	3786	KCNQ3
NPD_Sets	ID-denovo-nonsyn	26985	AP3M1
NPD_Sets	ID-denovo-nonsyn	2889	RAPGEF1
NPD_Sets	ID-denovo-nonsyn	51429	SNX9
NPD_Sets	ID-denovo-nonsyn	30000	TNPO2
NPD_Sets	ID-denovo-nonsyn	23348	DOCK9
NPD_Sets	ID-denovo-nonsyn	83992	CTTNBP2
NPD_Sets	ID-denovo-nonsyn	10087	COL4A3BP
NPD_Sets	ID-denovo-nonsyn	53831	GPR84
NPD_Sets	ID-denovo-nonsyn	85462	FHDC1
NPD_Sets	ID-denovo-nonsyn	55230	USP40
NPD_Sets	ID-denovo-nonsyn	4238	MFAP3
NPD_Sets	ID-denovo-nonsyn	3097	HIVEP2
NPD_Sets	ID-denovo-nonsyn	9737	GPRASP1
NPD_Sets	ID-denovo-nonsyn	8831	SYNGAP1
NPD_Sets	ID-denovo-nonsyn	114883	OSBPL9
NPD_Sets	ID-denovo-nonsyn	5925	RB1
NPD_Sets	ID-denovo-nonsyn	2890	GRIA1
NPD_Sets	ID-denovo-nonsyn	23028	KDM1A
NPD_Sets	ID-denovo-nonsyn	221656	KDM1B
NPD_Sets	ID-denovo-nonsyn	123207	C15orf40
NPD_Sets	ID-denovo-nonsyn	4627	MYH9
NPD_Sets	ID-denovo-nonsyn	6529	SLC6A1
NPD_Sets	ID-denovo-nonsyn	8503	PIK3R3
NPD_Sets	ID-denovo-nonsyn	222	ALDH3B2
NPD_Sets	ID-denovo-nonsyn	1742	DLG4
NPD_Sets	ID-denovo-nonsyn	818	CAMK2G

NPD_Sets	ID-denovo-nonsyn	440854	CAPN14
NPD_Sets	ID-denovo-nonsyn	9765	ZFYVE16
NPD_Sets	ID-denovo-nonsyn	6535	SLC6A8
NPD_Sets	ID-denovo-nonsyn	8546	AP3B1
NPD_Sets	ID-denovo-nonsyn	57489	ODF2L
NPD_Sets	ID-denovo-nonsyn	26040	SETBP1
NPD_Sets	ID-denovo-nonsyn	5528	PPP2R5D
NPD_Sets	ID-denovo-nonsyn	80210	ARMC9
NPD_Sets	ID-denovo-nonsyn	7991	TUSC3
NPD_Sets	ID-denovo-nonsyn	80071	CCDC15
NPD_Sets	ID-denovo-nonsyn	26115	TANC2
NPD_Sets	ID-denovo-nonsyn	3756	KCNH1
NPD_Sets	ID-denovo-nonsyn	8681	JMJD7-PLA2G4B
NPD_Sets	ID-denovo-nonsyn	2888	GRB14
NPD_Sets	ID-denovo-nonsyn	163255	ZNF540
NPD_Sets	ID-denovo-nonsyn	285093	CXXC11
NPD_Sets	ID-denovo-nonsyn	115019	SLC26A9
NPD_Sets	ID-denovo-nonsyn	4520	MTF1
NPD_Sets	ID-denovo-nonsyn	5934	RBL2
NPD_Sets	ID-denovo-nonsyn	64901	RANBP17
NPD_Sets	ID-denovo-nonsyn	9204	ZMYM6
NPD_Sets	ID-denovo-nonsyn	7343	UBTF
NPD_Sets	ID-denovo-nonsyn	9585	KIF20B
NPD_Sets	ID-denovo-nonsyn	10188	TNK2
NPD_Sets	ID-denovo-nonsyn	84217	ZMYND12
NPD_Sets	ID-denovo-nonsyn	390168	OR5M1
NPD_Sets	ID-denovo-nonsyn	79868	ALG13
NPD_Sets	ID-denovo-nonsyn	57534	MIB1
NPD_Sets	ID-denovo-nonsyn	4143	MAT1A
NPD_Sets	ID-denovo-nonsyn	55870	ASH1L
NPD_Sets	ID-denovo-nonsyn	283571	PROX2
NPD_Sets	ID-denovo-nonsyn	26074	C20orf26
NPD_Sets	ID-denovo-nonsyn	80351	TNKS2
NPD_Sets	ID-denovo-nonsyn	25820	ARIH1
NPD_Sets	ID-denovo-nonsyn	50863	NTM
NPD_Sets	ID-denovo-nonsyn	375612	LHFPL3
NPD_Sets	ID-denovo-nonsyn	2903	GRIN2A
NPD_Sets	ID-denovo-nonsyn	51322	WAC
NPD_Sets	ID-denovo-nonsyn	3882	KRT32
NPD_Sets	ID-denovo-nonsyn	2904	GRIN2B
NPD_Sets	miR-137	64137	ABCG4
NPD_Sets	miR-137	84945	ABHD13
NPD_Sets	miR-137	11057	ABHD2
NPD_Sets	miR-137	57406	ABHD6
NPD_Sets	miR-137	107	ADCY1
NPD_Sets	miR-137	108	ADCY2
NPD_Sets	miR-137	84890	ADO
NPD_Sets	miR-137	153	ADRB1
NPD_Sets	miR-137	56894	AGPAT3
NPD_Sets	miR-137	23382	AHCYL2
NPD_Sets	miR-137	84883	AIFM2
NPD_Sets	miR-137	11217	AKAP2
NPD_Sets	miR-137	208	AKT2
NPD_Sets	miR-137	8854	ALDH1A2
NPD_Sets	miR-137	57538	ALPK3
NPD_Sets	miR-137	23452	ANGPTL2
NPD_Sets	miR-137	23253	ANKRD12
NPD_Sets	miR-137	23243	ANKRD28

NPD_Sets	miR-137	91526	ANKRD44
NPD_Sets	miR-137	283373	ANKRD52
NPD_Sets	miR-137	57719	ANO8
NPD_Sets	miR-137	130340	AP1S3
NPD_Sets	miR-137	359	AQP2
NPD_Sets	miR-137	143872	ARHGAP42
NPD_Sets	miR-137	9912	ARHGAP44
NPD_Sets	miR-137	394	ARHGAP5
NPD_Sets	miR-137	23370	ARHGEF18
NPD_Sets	miR-137	55156	ARMC1
NPD_Sets	miR-137	444	ASPH
NPD_Sets	miR-137	22863	ATG14
NPD_Sets	miR-137	23250	ATP11A
NPD_Sets	miR-137	64756	ATPAF1
NPD_Sets	miR-137	342371	ATXN1L
NPD_Sets	miR-137	8707	B3GALT2
NPD_Sets	miR-137	9334	B4GALT5
NPD_Sets	miR-137	10018	BCL2L11
NPD_Sets	miR-137	63035	BCORL1
NPD_Sets	miR-137	8927	BSN
NPD_Sets	miR-137	399947	C11orf87
NPD_Sets	miR-137	147339	C18orf25
NPD_Sets	miR-137	54149	C21orf91
NPD_Sets	miR-137	126567	C2CD4C
NPD_Sets	miR-137	766	CA7
NPD_Sets	miR-137	775	CACNA1C
NPD_Sets	miR-137	8912	CACNA1H
NPD_Sets	miR-137	8911	CACNA1I
NPD_Sets	miR-137	93664	CADPS2
NPD_Sets	miR-137	808	CALM3
NPD_Sets	miR-137	83698	CALN1
NPD_Sets	miR-137	815	CAMK2A
NPD_Sets	miR-137	10645	CAMKK2
NPD_Sets	miR-137	154467	CCDC167
NPD_Sets	miR-137	317762	CCDC85C
NPD_Sets	miR-137	901	CCNG2
NPD_Sets	miR-137	23607	CD2AP
NPD_Sets	miR-137	8476	CDC42BPA
NPD_Sets	miR-137	10435	CDC42EP2
NPD_Sets	miR-137	55536	CDCA7L
NPD_Sets	miR-137	80205	CHD9
NPD_Sets	miR-137	1113	CHGA
NPD_Sets	miR-137	1121	CHM
NPD_Sets	miR-137	23122	CLASP2
NPD_Sets	miR-137	23274	CLEC16A
NPD_Sets	miR-137	246175	CNOT6L
NPD_Sets	miR-137	1310	COL19A1
NPD_Sets	miR-137	1289	COL5A1
NPD_Sets	miR-137	9318	COPS2
NPD_Sets	miR-137	9586	CREB5
NPD_Sets	miR-137	1399	CRKL
NPD_Sets	miR-137	27254	CSDC2
NPD_Sets	miR-137	64478	CSMD1
NPD_Sets	miR-137	10106	CTDSP2
NPD_Sets	miR-137	83992	CTTNBP2
NPD_Sets	miR-137	55917	CTTNBP2NL
NPD_Sets	miR-137	8452	CUL3
NPD_Sets	miR-137	1523	CUX1

NPD_Sets	miR-137	1525	CXADR
NPD_Sets	miR-137	6387	CXCL12
NPD_Sets	miR-137	116159	CYYR1
NPD_Sets	miR-137	50717	DCAF8
NPD_Sets	miR-137	51473	DCDC2
NPD_Sets	miR-137	166614	DCLK2
NPD_Sets	miR-137	163486	DENND1B
NPD_Sets	miR-137	9909	DENND4B
NPD_Sets	miR-137	148252	DIRAS1
NPD_Sets	miR-137	54769	DIRAS2
NPD_Sets	miR-137	54788	DNAJB12
NPD_Sets	miR-137	220164	DOK6
NPD_Sets	miR-137	1832	DSP
NPD_Sets	miR-137	25778	DSTYK
NPD_Sets	miR-137	113878	DTX2
NPD_Sets	miR-137	1846	DUSP4
NPD_Sets	miR-137	1847	DUSP5
NPD_Sets	miR-137	1943	EFNA2
NPD_Sets	miR-137	23167	EFR3A
NPD_Sets	miR-137	1995	ELAVL3
NPD_Sets	miR-137	114794	ELFN2
NPD_Sets	miR-137	256364	EML3
NPD_Sets	miR-137	2020	EN2
NPD_Sets	miR-137	2045	EPHA7
NPD_Sets	miR-137	2046	EPHA8
NPD_Sets	miR-137	2059	EPS8
NPD_Sets	miR-137	2101	ESRRA
NPD_Sets	miR-137	2104	ESRRG
NPD_Sets	miR-137	150864	FAM117B
NPD_Sets	miR-137	285172	FAM126B
NPD_Sets	miR-137	162427	FAM134C
NPD_Sets	miR-137	51059	FAM135B
NPD_Sets	miR-137	56975	FAM20C
NPD_Sets	miR-137	143684	FAM76B
NPD_Sets	miR-137	286336	FAM78A
NPD_Sets	miR-137	151354	FAM84A
NPD_Sets	miR-137	120114	FAT3
NPD_Sets	miR-137	114907	FBXO32
NPD_Sets	miR-137	121512	FGD4
NPD_Sets	miR-137	2256	FGF11
NPD_Sets	miR-137	2275	FHL3
NPD_Sets	miR-137	2288	FKBP4
NPD_Sets	miR-137	252995	FNDC5
NPD_Sets	miR-137	96459	FNIP1
NPD_Sets	miR-137	1112	FOXN3
NPD_Sets	miR-137	10818	FRS2
NPD_Sets	miR-137	5045	FURIN
NPD_Sets	miR-137	9568	GABBR2
NPD_Sets	miR-137	2554	GABRA1
NPD_Sets	miR-137	2589	GALNT1
NPD_Sets	miR-137	8139	GAN
NPD_Sets	miR-137	64599	GIGYF1
NPD_Sets	miR-137	26035	GLCE
NPD_Sets	miR-137	84662	GLIS2
NPD_Sets	miR-137	10672	GNA13
NPD_Sets	miR-137	2779	GNAT1
NPD_Sets	miR-137	2802	GOLGA3
NPD_Sets	miR-137	26003	GORASP2

NPD_Sets	miR-137	23131	GPATCH8
NPD_Sets	miR-137	56261	GPCPD1
NPD_Sets	miR-137	160897	GPR180
NPD_Sets	miR-137	2849	GPR26
NPD_Sets	miR-137	65983	GRAMD3
NPD_Sets	miR-137	23151	GRAMD4
NPD_Sets	miR-137	2890	GRIA1
NPD_Sets	miR-137	2893	GRIA4
NPD_Sets	miR-137	2932	GSK3B
NPD_Sets	miR-137	2977	GUCY1A2
NPD_Sets	miR-137	283464	GXYLT1
NPD_Sets	miR-137	3021	H3F3B
NPD_Sets	miR-137	610	HCN2
NPD_Sets	miR-137	139324	HDX
NPD_Sets	miR-137	57493	HEG1
NPD_Sets	miR-137	8916	HERC3
NPD_Sets	miR-137	3090	HIC1
NPD_Sets	miR-137	28996	HIPK2
NPD_Sets	miR-137	3101	HK3
NPD_Sets	miR-137	3131	HLF
NPD_Sets	miR-137	6596	HLTF
NPD_Sets	miR-137	220988	HNRNPA3
NPD_Sets	miR-137	3192	HNRNPU
NPD_Sets	miR-137	3224	HOXC8
NPD_Sets	miR-137	3358	HTR2C
NPD_Sets	miR-137	3416	IDE
NPD_Sets	miR-137	3417	IDH1
NPD_Sets	miR-137	64375	IKZF4
NPD_Sets	miR-137	3613	IMPA2
NPD_Sets	miR-137	3632	INPP5A
NPD_Sets	miR-137	8660	IRS2
NPD_Sets	miR-137	145501	ISM2
NPD_Sets	miR-137	80853	JHDM1D
NPD_Sets	miR-137	3736	KCNA1
NPD_Sets	miR-137	3756	KCNH1
NPD_Sets	miR-137	3760	KCNJ3
NPD_Sets	miR-137	10242	KCNMB2
NPD_Sets	miR-137	115207	KCTD12
NPD_Sets	miR-137	9682	KDM4A
NPD_Sets	miR-137	10765	KDM5B
NPD_Sets	miR-137	22889	KIAA0907
NPD_Sets	miR-137	85379	KIAA1671
NPD_Sets	miR-137	3815	KIT
NPD_Sets	miR-137	8462	KLF11
NPD_Sets	miR-137	11278	KLF12
NPD_Sets	miR-137	81606	LBH
NPD_Sets	miR-137	203190	LGI3
NPD_Sets	miR-137	10184	LHFPL2
NPD_Sets	miR-137	22998	LIMCH1
NPD_Sets	miR-137	22853	LMTK2
NPD_Sets	miR-137	23143	LRCH1
NPD_Sets	miR-137	390205	LRRC10B
NPD_Sets	miR-137	55222	LRRC20
NPD_Sets	miR-137	64101	LRRC4
NPD_Sets	miR-137	338645	LUZP2
NPD_Sets	miR-137	4094	MAF
NPD_Sets	miR-137	9794	MAML1
NPD_Sets	miR-137	6416	MAP2K4

NPD_Sets	miR-137	9175	MAP3K13
NPD_Sets	miR-137	5871	MAP4K2
NPD_Sets	miR-137	5602	MAPK10
NPD_Sets	miR-137	9261	MAPKAPK2
NPD_Sets	miR-137	64844	MARCH7
NPD_Sets	miR-137	8932	MBD2
NPD_Sets	miR-137	129642	MBOAT2
NPD_Sets	miR-137	51360	MBTPS2
NPD_Sets	miR-137	51108	METTL9
NPD_Sets	miR-137	11343	MGLL
NPD_Sets	miR-137	375056	MIA3
NPD_Sets	miR-137	166968	MIER3
NPD_Sets	miR-137	4286	MITF
NPD_Sets	miR-137	23609	MKRN2
NPD_Sets	miR-137	4354	MPP1
NPD_Sets	miR-137	4440	MSI1
NPD_Sets	miR-137	253827	MSRB3
NPD_Sets	miR-137	92140	MTDH
NPD_Sets	miR-137	4520	MTF1
NPD_Sets	miR-137	4084	MXD1
NPD_Sets	miR-137	4642	MYO1D
NPD_Sets	miR-137	4665	NAB2
NPD_Sets	miR-137	138151	NACC2
NPD_Sets	miR-137	339983	NAT8L
NPD_Sets	miR-137	8648	NCOA1
NPD_Sets	miR-137	8202	NCOA3
NPD_Sets	miR-137	63941	NECAB3
NPD_Sets	miR-137	4747	NEFL
NPD_Sets	miR-137	81832	NETO1
NPD_Sets	miR-137	4761	NEUROD2
NPD_Sets	miR-137	58158	NEUROD4
NPD_Sets	miR-137	4763	NF1
NPD_Sets	miR-137	23114	NFASC
NPD_Sets	miR-137	4772	NFATC1
NPD_Sets	miR-137	4781	NFIB
NPD_Sets	miR-137	4801	NFYB
NPD_Sets	miR-137	25836	NIPBL
NPD_Sets	miR-137	284353	NKPD1
NPD_Sets	miR-137	4851	NOTCH1
NPD_Sets	miR-137	4864	NPC1
NPD_Sets	miR-137	9542	NRG2
NPD_Sets	miR-137	4905	NSF
NPD_Sets	miR-137	23386	NUDCD3
NPD_Sets	miR-137	55916	NXT2
NPD_Sets	miR-137	23762	OSBP2
NPD_Sets	miR-137	56957	OTUD7B
NPD_Sets	miR-137	10605	PAIP1
NPD_Sets	miR-137	445815	PALM2-AKAP2
NPD_Sets	miR-137	11044	PAPD7
NPD_Sets	miR-137	79668	PARP8
NPD_Sets	miR-137	115294	PCMTD1
NPD_Sets	miR-137	10015	PDCD6IP
NPD_Sets	miR-137	10846	PDE10A
NPD_Sets	miR-137	5141	PDE4A
NPD_Sets	miR-137	23244	PDS5A
NPD_Sets	miR-137	23037	PDZD2
NPD_Sets	miR-137	8504	PEX3
NPD_Sets	miR-137	5210	PFKFB4

NPD_Sets	miR-137	79960	PHF17
NPD_Sets	miR-137	90102	PHLDB2
NPD_Sets	miR-137	8503	PIK3R3
NPD_Sets	miR-137	79837	PIP4K2C
NPD_Sets	miR-137	5306	PITPNA
NPD_Sets	miR-137	57605	PITPNM2
NPD_Sets	miR-137	23659	PLA2G15
NPD_Sets	miR-137	55041	PLEKHB2
NPD_Sets	miR-137	26030	PLEKHG3
NPD_Sets	miR-137	80301	PLEKHO2
NPD_Sets	miR-137	56937	PMEPA1
NPD_Sets	miR-137	5432	POLR2C
NPD_Sets	miR-137	10891	PPARGC1A
NPD_Sets	miR-137	133522	PPARGC1B
NPD_Sets	miR-137	22843	PPM1E
NPD_Sets	miR-137	5500	PPP1CB
NPD_Sets	miR-137	26051	PPP1R16B
NPD_Sets	miR-137	55607	PPP1R9A
NPD_Sets	miR-137	9701	PPP6R2
NPD_Sets	miR-137	84279	PRADC1
NPD_Sets	miR-137	63976	PRDM16
NPD_Sets	miR-137	5562	PRKAA1
NPD_Sets	miR-137	5564	PRKAB1
NPD_Sets	miR-137	5577	PRKAR2B
NPD_Sets	miR-137	51334	PRR16
NPD_Sets	miR-137	5738	PTGFRN
NPD_Sets	miR-137	11156	PTP4A3
NPD_Sets	miR-137	5784	PTPN14
NPD_Sets	miR-137	84867	PTPN5
NPD_Sets	miR-137	5829	PXN
NPD_Sets	miR-137	9444	QKI
NPD_Sets	miR-137	9771	RAPGEF5
NPD_Sets	miR-137	65059	RAPH1
NPD_Sets	miR-137	11228	RASSF8
NPD_Sets	miR-137	55225	RAVER2
NPD_Sets	miR-137	55285	RBM41
NPD_Sets	miR-137	283248	RCOR2
NPD_Sets	miR-137	57455	REXO1
NPD_Sets	miR-137	23180	RFTN1
NPD_Sets	miR-137	9628	RGS6
NPD_Sets	miR-137	55188	RIC8B
NPD_Sets	miR-137	253260	RICTOR
NPD_Sets	miR-137	57494	RIMKLB
NPD_Sets	miR-137	140730	RIMS4
NPD_Sets	miR-137	64795	RMND5A
NPD_Sets	miR-137	50862	RNF141
NPD_Sets	miR-137	57484	RNF150
NPD_Sets	miR-137	285671	RNF180
NPD_Sets	miR-137	80352	RNF39
NPD_Sets	miR-137	6047	RNF4
NPD_Sets	miR-137	6095	RORA
NPD_Sets	miR-137	6138	RPL15
NPD_Sets	miR-137	6158	RPL28
NPD_Sets	miR-137	58528	RRAGD
NPD_Sets	miR-137	6239	RREB1
NPD_Sets	miR-137	6263	RYR3
NPD_Sets	miR-137	51128	SAR1B
NPD_Sets	miR-137	388228	SBK1

NPD_Sets	miR-137	55206	SBNO1
NPD_Sets	miR-137	83482	SCRT1
NPD_Sets	miR-137	85508	SCRT2
NPD_Sets	miR-137	51714	SELT
NPD_Sets	miR-137	55964	SEPT3
NPD_Sets	miR-137	27230	SERP1
NPD_Sets	miR-137	91404	SESTD1
NPD_Sets	miR-137	23067	SETD1B
NPD_Sets	miR-137	57713	SFMBT2
NPD_Sets	miR-137	6445	SGCG
NPD_Sets	miR-137	8879	SGPL1
NPD_Sets	miR-137	54557	SGTB
NPD_Sets	miR-137	53358	SHC3
NPD_Sets	miR-137	729993	SHISA9
NPD_Sets	miR-137	357	SHROOM2
NPD_Sets	miR-137	150094	SIK1
NPD_Sets	miR-137	57568	SIPA1L2
NPD_Sets	miR-137	6497	SKI
NPD_Sets	miR-137	6558	SLC12A2
NPD_Sets	miR-137	57468	SLC12A5
NPD_Sets	miR-137	63027	SLC22A23
NPD_Sets	miR-137	25769	SLC24A2
NPD_Sets	miR-137	7782	SLC30A4
NPD_Sets	miR-137	10559	SLC35A1
NPD_Sets	miR-137	113829	SLC35A4
NPD_Sets	miR-137	9906	SLC35E2
NPD_Sets	miR-137	728661	SLC35E2B
NPD_Sets	miR-137	124935	SLC43A2
NPD_Sets	miR-137	85414	SLC45A3
NPD_Sets	miR-137	9497	SLC4A7
NPD_Sets	miR-137	60482	SLC5A7
NPD_Sets	miR-137	6529	SLC6A1
NPD_Sets	miR-137	6535	SLC6A8
NPD_Sets	miR-137	6536	SLC6A9
NPD_Sets	miR-137	6546	SLC8A1
NPD_Sets	miR-137	8467	SMARCA5
NPD_Sets	miR-137	57154	SMURF1
NPD_Sets	miR-137	8303	SNN
NPD_Sets	miR-137	54861	SNRK
NPD_Sets	miR-137	6667	SP1
NPD_Sets	miR-137	56848	SPHK2
NPD_Sets	miR-137	9806	SPOCK2
NPD_Sets	miR-137	144108	SPTY2D1
NPD_Sets	miR-137	6714	SRC
NPD_Sets	miR-137	6431	SRSF6
NPD_Sets	miR-137	23648	SSBP3
NPD_Sets	miR-137	117178	SSX2IP
NPD_Sets	miR-137	6767	ST13
NPD_Sets	miR-137	6487	ST3GAL3
NPD_Sets	miR-137	84620	ST6GAL2
NPD_Sets	miR-137	140901	STK35
NPD_Sets	miR-137	11329	STK38
NPD_Sets	miR-137	83931	STK40
NPD_Sets	miR-137	6801	STRN
NPD_Sets	miR-137	9515	STXBP5L
NPD_Sets	miR-137	6815	STYX
NPD_Sets	miR-137	9900	SV2A
NPD_Sets	miR-137	6854	SYN2

NPD_Sets	miR-137	79953	SYNDIG1
NPD_Sets	miR-137	8867	SYNJ1
NPD_Sets	miR-137	54457	TAF7L
NPD_Sets	miR-137	23216	TBC1D1
NPD_Sets	miR-137	23232	TBC1D12
NPD_Sets	miR-137	6913	TBX15
NPD_Sets	miR-137	6926	TBX3
NPD_Sets	miR-137	6925	TCF4
NPD_Sets	miR-137	7003	TEAD1
NPD_Sets	miR-137	9895	TECPR2
NPD_Sets	miR-137	7020	TFAP2A
NPD_Sets	miR-137	7068	THRB
NPD_Sets	miR-137	7082	TJP1
NPD_Sets	miR-137	50999	TMED5
NPD_Sets	miR-137	114795	TMEM132B
NPD_Sets	miR-137	100113407	TMEM170B
NPD_Sets	miR-137	202915	TMEM184A
NPD_Sets	miR-137	161145	TMEM229B
NPD_Sets	miR-137	55161	TMEM33
NPD_Sets	miR-137	148534	TMEM56
NPD_Sets	miR-137	81542	TMX1
NPD_Sets	miR-137	23112	TNRC6B
NPD_Sets	miR-137	27348	TOR1B
NPD_Sets	miR-137	53373	TPCN1
NPD_Sets	miR-137	9697	TRAM2
NPD_Sets	miR-137	7222	TRPC3
NPD_Sets	miR-137	7257	TSNAX
NPD_Sets	miR-137	340348	TSPAN33
NPD_Sets	miR-137	23331	TTC28
NPD_Sets	miR-137	23508	TTC9
NPD_Sets	miR-137	56995	TULP4
NPD_Sets	miR-137	7326	UBE2G1
NPD_Sets	miR-137	55284	UBE2W
NPD_Sets	miR-137	65264	UBE2Z
NPD_Sets	miR-137	254048	UBN2
NPD_Sets	miR-137	92181	UBTD2
NPD_Sets	miR-137	9706	ULK2
NPD_Sets	miR-137	57646	USP28
NPD_Sets	miR-137	84749	USP30
NPD_Sets	miR-137	79805	VASH2
NPD_Sets	miR-137	79001	VKORC1
NPD_Sets	miR-137	90113	VWA5B2
NPD_Sets	miR-137	23286	WWC1
NPD_Sets	miR-137	11060	WWP2
NPD_Sets	miR-137	64328	XPO4
NPD_Sets	miR-137	54464	XRN1
NPD_Sets	miR-137	91746	YTHDC1
NPD_Sets	miR-137	51776	ZAK
NPD_Sets	miR-137	51341	ZBTB7A
NPD_Sets	miR-137	51043	ZBTB7B
NPD_Sets	miR-137	23144	ZC3H3
NPD_Sets	miR-137	340481	ZDHHC21
NPD_Sets	miR-137	51304	ZDHHC3
NPD_Sets	miR-137	678	ZFP36L2
NPD_Sets	miR-137	9765	ZFYVE16
NPD_Sets	miR-137	7750	ZMYM2
NPD_Sets	miR-137	7707	ZNF148
NPD_Sets	miR-137	25946	ZNF385A

NPD_Sets	miR-137	203523	ZNF449
NPD_Sets	miR-137	55279	ZNF654
NPD_Sets	miR-137	374655	ZNF710
NPD_Sets	miR-137	7629	ZNF76
NPD_Sets	mGluR5	989	SEPT7
NPD_Sets	mGluR5	9456	HOMER1
NPD_Sets	mGluR5	1785	DNM2
NPD_Sets	mGluR5	11346	SYNPO
NPD_Sets	mGluR5	55690	PACS1
NPD_Sets	mGluR5	230	ALDOC
NPD_Sets	mGluR5	490	ATP2B1
NPD_Sets	mGluR5	118	ADD1
NPD_Sets	mGluR5	8867	SYNJ1
NPD_Sets	mGluR5	11034	DSTN
NPD_Sets	mGluR5	5413	SEPT5
NPD_Sets	mGluR5	119	ADD2
NPD_Sets	mGluR5	11113	CIT
NPD_Sets	mGluR5	4905	NSF
NPD_Sets	mGluR5	8927	BSN
NPD_Sets	mGluR5	2664	GDI1
NPD_Sets	mGluR5	1759	DNM1
NPD_Sets	mGluR5	5864	RAB3A
NPD_Sets	mGluR5	163	AP2B1
NPD_Sets	mGluR5	3897	L1CAM
NPD_Sets	mGluR5	7533	YWHAH
NPD_Sets	mGluR5	7532	YWHAG
NPD_Sets	mGluR5	1213	CLTC
NPD_Sets	mGluR5	10971	YWHAQ
NPD_Sets	mGluR5	50944	SHANK1
NPD_Sets	mGluR5	2775	GNAO1
NPD_Sets	mGluR5	7534	YWHAZ
NPD_Sets	mGluR5	226	ALDOA
NPD_Sets	mGluR5	481	ATP1B1
NPD_Sets	mGluR5	23236	PLCB1
NPD_Sets	mGluR5	7529	YWHAB
NPD_Sets	mGluR5	4130	MAP1A
NPD_Sets	mGluR5	4131	MAP1B
NPD_Sets	mGluR5	4133	MAP2
NPD_Sets	mGluR5	476	ATP1A1
NPD_Sets	mGluR5	477	ATP1A2
NPD_Sets	mGluR5	478	ATP1A3
NPD_Sets	mGluR5	22895	RPH3A

SI Table 3. Cell line description and available clinical information for all experiments.

Pilot	Phase 1 Plate Number	Phase 2 Plate Number	RNAseq (loxapine or methylparaben)	Cell Type	Cohort	Source	Patient ID	Somatic Cell	Reprogramming Method	NPC Differentiation	NPC Line ID for L1000	NPC Passage	L1000 Phase	NPC Line IDs for loxapine RNAseq	NPC Line IDs for methylparaben RNAseq	Sex	Dx	Ethnicity	Age of Onset	IQ	Developmental History	Clozapine Response	Family History	SZ-related CNV Burden
na	1	1	na	hiPSC NPC	SZ1	ATCC	CNTL-2522	fibroblast	tet-inducible lentivirus	EB	BJ-2-E (1), BJ-2-A (2)	p7-p10	1 & 2	na	na	M	Control	Causasian	n/a	unknown	unknown	unknown	unknown	-
na	3	1	na	hiPSC NPC	SZ1	Coriell	GM03440	fibroblast	tet-inducible lentivirus	EB	3440-5-A	p7-p10	1 & 2	na	na	M	Control	Causasian	n/a	unknown	unknown	unknown	unknown	-
1	2	1	na	hiPSC NPC	SZ1	Coriell	GM03651	fibroblast	tet-inducible lentivirus	EB	3651-A-A	p7-p10	1 & 2	na	na	F	Control	Causasian	n/a	unknown	unknown	unknown	unknown	-
na	4	2	na	hiPSC NPC	SZ1	Coriell	GM04506	fibroblast	tet-inducible lentivirus	EB	4506-B-A	p7-p10	1 & 2	na	na	F	Control	Causasian	n/a	unknown	unknown	unknown	unknown	-
na	4	na	na	hiPSC NPC	SZ1	Coriell	AG09319	fibroblast	tet-inducible lentivirus	EB	9319-2-A	p7-p10	1	na	na	F	Control	Causasian	n/a	unknown	unknown	unknown	unknown	-
na	2	3	L	hiPSC NPC	SZ2	NIMH	NSB2607	fibroblast	sendai virus	EB + dual-SMAD	2607-3-1	p5	1 & 2	2607-3-1, 2607-4-1	na	M	Control	Caucasian	n/a	126	clean	n/a	-	-
na	3	4	L,M	hiPSC NPC	SZ2	NIMH	NSB3084	fibroblast	sendai virus	EB + dual-SMAD	3084-2-2	p7	1 & 2	3084-2-2, 3084-1-1	3084-1-1	M	Control	Black, non-Hispanic	n/a	87	clean	n/a	-	-
na	1	2	na	hiPSC NPC	SZ2	NIMH	NSB3113	fibroblast	sendai virus	EB + dual-SMAD	3113-6-3	p5	1 & 2	na	na	F	Control	Caucasian	n/a	123	clean	n/a	-	-
na	1	4	M	hiPSC NPC	SZ2	NIMH	NSB3121	fibroblast	sendai virus	EB + dual-SMAD	3121-2-1	p5	1 & 2	na	3121-2-1, 3121-2-2	F	Control	Caucasian	n/a	134	clean	n/a	-	-
na	na	3	M	hiPSC NPC	SZ2	NIMH	NSB3182	fibroblast	sendai virus	EB + dual-SMAD	3182-2-4	p5	2	na	3182-2-4, 3182-3-1	F	Control	Caucasian	n/a	119	clean	n/a	-	-
na	4	4	na	hiPSC NPC	SZ2	NIMH	NSB3234	fibroblast	sendai virus	EB + dual-SMAD	3234-2-4	p5	1 & 2	na	na	M	Control	Indian	n/a	94	clean	n/a	-	-
na	4	3	L	hiPSC NPC	SZ2	NIMH	NSB553	fibroblast	sendai virus	EB + dual-SMAD	553-3-1	p5	1 & 2	553-3-1, 553-S1-C	na	M	Control	Caucasian	n/a	127	clean	n/a	-	-
na	2	2	na	hiPSC NPC	SZ2	NIMH	NSB690	fibroblast	sendai virus	EB + dual-SMAD	690-2-1	p5	1 & 2	na	na	M	Control	Caucasian	n/a	115	clean	n/a	-	-
na	3	1	na	hiPSC NPC	SZ1	Coriell	GM02038	fibroblast	tet-inducible lentivirus	EB	2038-1-A	p7-p10	1 & 2	na	na	M	Schizophrenia	Causasian	6	unknown	unknown	unknown	unknown	-
na	3	1	na	hiPSC NPC	SZ1	Coriell	GM01792	fibroblast	tet-inducible lentivirus	EB	1792-1-E	p7-p10	1 & 2	na	na	M	Schizophrenia	Causasian Jewish /Scandanavian	unknown	unknown	unknown	unknown	SZ, SA, ASD	-
1	2	1	na	hiPSC NPC	SZ1	Coriell	GM01835	fibroblast	tet-inducible lentivirus	EB	1835-1-3 (P, 2), 1835-1-5 (1)	p7-p10	1 & 2	na	na	F	Schizo-affective	Causasian Jewish	unknown	unknown	unknown	unknown	SZ, ASD	-
na	4	2	na	hiPSC NPC	SZ1	Coriell	GM02497	fibroblast	tet-inducible lentivirus	EB	2497-1-C	p7-p10	1 & 2	na	na	M	Schizophrenia	Causasian Jewish	15	unknown	unknown	unknown	SZ, SPD	-
na	1	na	na	hiPSC NPC	SZ2	NIMH	NSB1275	fibroblast	sendai virus	EB + dual-SMAD	1275-B-3	p5	1	na	na	F	COS	Caucasian	10	67	MDD	NA	SPD	22q11.2
na	1	3	M	hiPSC NPC	SZ2	NIMH	NSB2011	fibroblast	sendai virus	EB + dual-SMAD	2011-3-12	p5	1 & 2	na	2011-3-12, 2011-4-4	F	COS	Caucasian	8	69	MDD	Y	SPD; PPD	16p11.2
na	2	4	L	hiPSC NPC	SZ2	NIMH	NSB2476	fibroblast	sendai virus	EB + dual-SMAD	2476-D1-2	p7	1 & 2	2476-D1-2, 2476-D4-4	na	F	COS	Caucasian-Hispanic	8	82	clean	Y	APD	-
na	3	4	L	hiPSC NPC	SZ2	NIMH	NSB2513	fibroblast	sendai virus	EB + dual-SMAD	2513-2-4	p5	1 & 2	2513-2-4, 2513-1-1	na	M	COS	Caucasian	4	55	ASD	N	BD	-
na	na	2	M	hiPSC NPC	SZ2	NIMH	NSB2962	fibroblast	sendai virus	EB + dual-SMAD	2962-2-1	p5	2	na	2962-2-1, 2962-1-3	M	COS	Caucasian-Hispanic	8	78	clean	Y	SZ/ASD	-
na	4	3	na	hiPSC NPC	SZ2	NIMH	NSB499	fibroblast	sendai virus	EB + dual-SMAD	499-2-12	p5	1 & 2	na	na	M	COS	Caucasian	12	84	clean	N	SPD	3p25.3
na	3	4	na	hiPSC NPC	SZ2	NIMH	NSB581	fibroblast	sendai virus	EB + dual-SMAD	581-2-1	p5	1 & 2	na	na	M	COS	Caucasian	7	74	MDD	N	SA	2p16.3 del
na	2	2	L,M	hiPSC NPC	SZ2	NIMH	NSB676	fibroblast	sendai virus	EB + dual-SMAD	676-2-3	p5	1 & 2	676-1-2	676-2-3, 676-1-2	F	COS	Caucasian	10	79	MDD	N	PPD	16p11.2
na	1	3	na	hiPSC NPC	SZ2	NIMH	NSB2484	fibroblast	sendai virus	EB + dual-SMAD	2484-2-A	p6	1 & 2	na	na	F	COS	Caucasian	10	98	MDD	N	PPD	-
1	1	1	na	CCL	SH-SY5Y	ATCC	CRL-2266	na	na	na	na	na	1 & 2	na	na	F	neuroblastoma, bone marrow	unknown	unknown	unknown	unknown	unknown	unknown	unknown
na	1	1	na	CCL	MCF7	ATCC	HTB-22	na	na	na	na	na	1 & 2	na	na	F	adenocarcinoma, metastic mammary glad	Caucasian	unknown	unknown	unknown	unknown	unknown	unknown
na	2	2	na	CCL	A673	ATCC	CRL-1598	na	na	na	na	na	1 & 2	na	na	F	Ewing's sarcoma, muscle	unknown	unknown	unknown	unknown	unknown	unknown	unknown
na	2	2	na	CCL	AGS	ATCC	CRL-1739	na	na	na	na	na	1 & 2	na	na	F	gastric adenocarcinoma, stomach	Caucasian	unknown	unknown	unknown	unknown	unknown	unknown
1	3	4	na	CCL	A549	ATCC	CCL-185	na	na	na	na	na	1 & 2	na	na	M	carcinoma, lung	Caucasian	unknown	unknown	unknown	unknown	unknown	unknown
na	3	4	na	CCL	HepG2	ATCC	HB-8065	na	na	na	na	na	1 & 2	na	na	M	hepatocellular carcinoma, liver	Caucasian	unknown	unknown	unknown	unknown	unknown	unknown
na	4	3	na	CCL	VCaP	ATCC	CRL-2876	na	na	na	na	na	1 & 2	na	na	M	cancer, prostate	Caucasian	unknown	unknown	unknown	unknown	unknown	unknown
na	4	3	na	CCL	HT29	ATCC	HTB-38	na	na	na	na	na	1 & 2	na	na	F	colorectal adenocarcinoma, colon	Caucasian	unknown	unknown	unknown	unknown	unknown	unknown

Abbreviations: tetracycline (tet), embryoid body (EB), childhood-onset schizophrenia (COS), miscellaneous developmental delay (MDD), autism spectrum disorder (ASD), apparently neurotypical (clean), schizoid personality disorder (SPD), paranoid personality disorder (PPD), bipolar disorder (BD), antisocial personality disorder (APD)

SI Table 4. Drug concentrations used in L1000 screening experiments.

Pilot	Concentration (µM)	Time (h)	Batch 1	Concentration (µM)	Time (h)	Batch 2	Concentration (µM)	Time (h)
Amisulpride (D2R antagonist)	0.01, 0.1	6, 18	3-Acetamidocoumarin	10.00	6	3-Acetamidocoumarin	0.01	6
BAY K8644 (Ca(V)1 agonist)	1, 30	6, 18	4-Hydroxyphenazone	10.00	6	Androsterone	0.03	6
LY341495 (mGluR2/3 antagonist)	0.01, 0.1	6, 18	Alpha-Ergocryptine	7.00	6	Anisomycin (A 00010) 3-Acetoxy-2-(4-Methoxybenzyl)-4-Pyrrolidinol	0.10	6
LY3792628 (mGluR2/3 agonist)	0.01, 0.1	6, 18	Amoxapine	0.10	6	Aripiprazole	0.03	6
MDL 100907 (5HT2A antagonist)	0.5, 0.05	6, 18	Anisomycin	10.00	6	Bay K8644 100 Mm	1.34	6
MK801 (NMDAR antagonist)	10, 100	6, 18	Arachidonyltrifluoromethane	10.00	6	Benzyloxycarbonyl-L-Glycyl-L-Phenylalanyl-L-Phenylalanyl-L-Tyrosinebenzyl Ester	1.00	6
NMDA (NMDAR agonist)	10, 100	6, 18	Arcaine	10.00	6	Bms-250904	0.30	6
Nimodipine (Ca(V)1 antagonist)	1, 10	6, 18	Aripiprazole	1.00	6	Bosutinib	0.01	6
Quinpirole (D2R agonist)	0.01, 0.1	6, 18	Bas-012416453	10.00	6	Buflomedil Hydrochloride	1.00	6
TCB-2 (5HT2A agonist)	0.01, 0.1	6, 18	Bay K8644	1.00	6	Bupropion Hydrochloride	0.30	6
			Bergenin	1.00	6	Celastrol	10.00	6
			Bucladesine	10.00	6	Cetocycline' 'A-13428'	0.30	6
			Budesonide	9.20	6	Chenodeoxycholic Acid	10.00	6
			Bumetanide	10.00	6	Chlorpromazine Hydrochloride	0.03	6
			Carbimazole	10.00	6	Clozapine	0.10	6
			Celastrol	2.50	6	Cm Za 0576	1.00	6
			Cephaeline	6.00	6	Conessine	0.30	6
			Chenodeoxycholic Acid	10.00	6	Corticosterone	0.01	6
			Chlorcyclizine	1.00	6	Dihydrostilbestrol	0.00	6
			Chlorpromazine	0.10	6	Diltiazem Hydrochloride	0.30	6
			Diclofenamide	10.00	6	Diphenylaminotriazine	3.00	6
			Diflorasone	8.00	6	Dl-Thiorphan	0.30	6
			Dihydroergocristine	1.00	6	Dorzolamide Hydrochloride	0.10	6
			Diltiazem	3.00	6	Eplerenone	3.00	6
			Diphemanil Metilsulfate	10.00	6	Equilenin	0.03	6
			Dl-Thiorphan	1.00	6	Estriol	0.03	6
			Emetine	7.20	6	Estrone	0.03	6
			Ethionamide	10.00	6	Ethoxzolamide	0.00	6
			Fendiline	10.00	6	Etifenin	1.00	6
			Flecainide	8.40	6	Gabazine	1.00	6
			Furosemide	10.00	6	Glaxo:Gr247538	1.00	6
			Haloperidol	0.10	6	Glipizide	0.00	6
			Hydroquinine	1.00	6	Gt-2394 Rac	10.00	6
			Isoniazid	10.00	6	Harmine Hydrochloride	0.30	6
			Lansoprazole	10.00	6	Iloperidone	0.01	6
			Levothyroxine Sodium	1.00	6	Iofetamine Hydrochloride	10.00	6
			Liothyronine	3.20	6	Kanpaullone	0.10	6
			Loxapine	1.00	6	Lamivudine	3.00	6
			Ly379268	0.10	6	Ly379268	0.13	6
			Lycorine	10.00	6	Lycorine	3.00	6
			Mdl100907	0.50	6	Mafenide Hydrochloride	0.00	6
			Mebendazole	10.00	6	Mdl 29951	3.00	6
			Melatonin	1.00	6	Mdl100907	0.67	6
			Methylbenzethonium Chloride	8.60	6	Mebendazole	0.30	6
			Moxonidine	1.00	6	Memantine Hydrochloride	0.01	6
			Myeloperoxidase Inhibitor-I	1.10	6	Methylparaben	10.00	6
			Nadolol	10.00	6	Moclobemide	10.00	6
			Naringenin	10.00	6	N6-Cyclopentyladenosine	1.00	6
			Nefopam	1.38	6	Naloxone Hydrochloride	0.00	6
			Niclosamide	10.00	6	Naltrexone Hydrochloride	0.00	6
			Niflumic Acid	10.00	6	Nefopam	0.30	6
			Nimodipine	1.00	6	Niclosamide	0.03	6
			Nordihydroguaiaretic Acid	1.00	6	Nimodipine	1.34	6
			Phenazone	1.00	6	Nogestrel	0.01	6
			Phenelzine	10.00	6	Norethindrone	0.01	6
			Phenyl Biguanide	10.00	6	Norfloxacin	10.00	6
			Pizotifen	1.00	6	Perceptin	0.30	6
			Pk11195	1.00	6	Phenelzine Sulfate	0.00	6
			Prenylamine	9.60	6	Podophyllotoxin	0.01	6
			Prestwick-675	10.00	6	Potassium Estrone Sulfate	0.03	6
			Primidone	10.00	6	Quetiapine	0.30	6
			Quetiapine	10.00	6	Quinpirole	0.13	6
			Quinpirole	0.10	6	Quipazine, N-Methyl-, Dimaleate	0.03	6
			Repaglinide	8.80	6	Risperidone	0.03	6
			Ronidazole	1.00	6	Salsolidin	10.00	6
			Salsolidin	1.00	6	Sb 43152	1.00	6
			Sotalol	10.00	6	Sb-242235	0.30	6
			Spiradoline	0.10	6	Sorafenib	0.03	6
			Sulconazole	8.60	6	Spironolactone	0.10	6
			Suloctidil	10.00	6	Suloctidil	1.00	6
			Tanespimycin	1.00	6	Tanespimycin	1.34	6
			Tcb-2 10 Mm	0.10	6	Tcb-2	0.13	6
			Trazodone	1.00	6	Tiagabine	1.00	6
			Trichostatin A	0.10	6	Tranylcypromine Hydrochloride	3.00	6
			Trimethadione	10.00	6	Trichostatin A	0.13	6
			Trimethobenzamide	1.00	6	Unc0638	0.10	6
			Tyloxapol	4.00	6	Urapidil, 5-Methyl-	0.10	6
			Vigabatrin	10.00	6	Vandetanib	0.30	6
			Vorinostat	1.00	6	Vatalanib	1.00	6
			Zardaverine	10.00	6	Wb-4101 Hydrochloride	0.00	6
			Ziprasidone	0.10	6	Ziprasidone	0.03	6
