# Supplementary material for: Expression-based drug screening of neural progenitor cells from individuals with schizophrenia
Source: Nat Commun. 2018 Oct 24;9:4412. doi: 10.1038/s41467-018-06515-4 (PMC6200740; doi:10.1038/s41467-018-06515-4)
Supplement: Supplementary file 2 — Description of Additional Supplementary Files [file 41467_2018_6515_MOESM2_ESM.pdf]

Description of Additional Supplementary Files

File Name: Supplementary Data 1
Description: Differentially expressed genes, SZ-sets and chemogenomic enrichments between hiPSC NPCs and CCLs for each drug.

File Name: Supplementary Data 2
Description: Summary of drug induced perturbations that normalize post-mortem SZ DE genes.
